# Microanatomy of the Human Atherosclerotic Plaque by Single-Cell Transcriptomics

**DOI:** 10.1161/CIRCRESAHA.120.316770

**Published:** 2020-09-28

**Authors:** Marie A.C. Depuydt, Koen H.M. Prange, Lotte Slenders, Tiit Örd, Danny Elbersen, Arjan Boltjes, Saskia C.A. de Jager, Folkert W. Asselbergs, Gert J. de Borst, Einari Aavik, Tapio Lönnberg, Esther Lutgens, Christopher K. Glass, Hester M. den Ruijter, Minna U. Kaikkonen, Ilze Bot, Bram Slütter, Sander W. van der Laan, Seppo Yla-Herttuala, Michal Mokry, Johan Kuiper, Menno P.J. de Winther, Gerard Pasterkamp

**Affiliations:** 1 Leiden Academic Centre for Drug Research, Division of Biotherapeutics, Leiden University, Einsteinweg 55, Leiden, the Netherlands (M.A.C.D., I.B., B.S., J.K.).; 2 Amsterdam University Medical Centers–Location AMC, University of Amsterdam, Experimental Vascular Biology, Department of Medical Biochemistry, Amsterdam Cardiovascular Sciences, Amsterdam Infection and Immunity, Meibergdreef 9, the Netherlands (K.H.M.P., M.P.J.d.W.).; 3 Laboratory of Clinical Chemistry and Haematology, University Medical Center, Heidelberglaan 100, Utrecht, the Netherlands (L.S., A.B., F.W.A., S.W.v.d.L., M.M., G.P.).; 4 A.I.Virtanen Institute for Molecular Sciences, University of Eastern Finland, Kuopio, Finland (T.O., E.A., M.U.K., S.Y.-H.).; 5 Laboratory for Experimental Cardiology (D.E., S.C.A.d.J), University Medical Center Utrecht, Heidelberglaan 100, the Netherlands.; 6 Vascular Surgery (G.J.d.B.), University Medical Center Utrecht, Heidelberglaan 100, the Netherlands.; 7 Cardiology (H.M.d.R., M.M.), University Medical Center Utrecht, Heidelberglaan 100, the Netherlands.; 8 Turku Bioscience Centre, University of Turku and Åbo Akademi University, Finland (T.L.).; 9 Institute for Cardiovascular Prevention (IPEK), Munich, Germany (E.L., M.P.J.d.W.).; 10 German Center for Cardiovascular Research (DZHK), partner site Munich Heart Alliance, Munich, Germany (E.L., M.P.J.d.W.).; 11 Cell and Molecular Medicine (C.K.G.), University of California San Diego, CA.; 12 School of Medicine (C.K.G.), University of California San Diego, CA.

**Keywords:** atherosclerosis, cardiovascular disease, genome-wide association study, single-cell analysis

## Abstract

**Rationale:**

Atherosclerotic lesions are known for their cellular heterogeneity, yet the molecular complexity within the cells of human plaques has not been fully assessed.

**Objective:**

Using single-cell transcriptomics and chromatin accessibility, we gained a better understanding of the pathophysiology underlying human atherosclerosis.

**Methods and Results:**

We performed single-cell RNA and single-cell ATAC sequencing on human carotid atherosclerotic plaques to define the cells at play and determine their transcriptomic and epigenomic characteristics. We identified 14 distinct cell populations including endothelial cells, smooth muscle cells, mast cells, B cells, myeloid cells, and T cells and identified multiple cellular activation states and suggested cellular interconversions. Within the endothelial cell population, we defined subsets with angiogenic capacity plus clear signs of endothelial to mesenchymal transition. CD4^+^ and CD8^+^ T cells showed activation-based subclasses, each with a gradual decline from a cytotoxic to a more quiescent phenotype. Myeloid cells included 2 populations of proinflammatory macrophages showing IL (interleukin) 1B or TNF (tumor necrosis factor) expression as well as a foam cell-like population expressing TREM2 (triggering receptor expressed on myeloid cells 2) and displaying a fibrosis-promoting phenotype. ATACseq data identified specific transcription factors associated with the myeloid subpopulation and T cell cytokine profiles underlying mutual activation between both cell types. Finally, cardiovascular disease susceptibility genes identified using public genome-wide association studies data were particularly enriched in lesional macrophages, endothelial, and smooth muscle cells.

**Conclusions:**

This study provides a transcriptome-based cellular landscape of human atherosclerotic plaques and highlights cellular plasticity and intercellular communication at the site of disease. This detailed definition of cell communities at play in atherosclerosis will facilitate cell-based mapping of novel interventional targets with direct functional relevance for the treatment of human disease.

## Abstract 2

Supplemental Digital Content is available in the text.

**Meet the First Author, see p 1346**

Atherosclerosis is characterized by chronic, lipid-driven vascular inflammation and is the main underlying cause of cardiovascular disease (CVD).^1^ Many studies have defined cellular profiles of human atherosclerosis based on single or several marker proteins, but detailed description of the cells involved in the pathophysiology of atherogenesis is lacking. Moreover, genome-wide association studies (GWAS) have identified many loci associated with increased risk for CVD, but the translation of these findings into new therapies^2^ has been hampered by the lack of information on specific cell communities in atherosclerotic plaques and the cell-specific expression patterns of druggable candidate genes at the site of disease. Recently, the immune cell composition of murine and human aortic atherosclerotic plaques has been described using cytometry by time of flight and single-cell RNA sequencing (scRNA-seq).^3–7^ Yet, the full cellular composition of human carotid plaques, including nonimmune cells, remains elusive. Therefore, we performed scRNA-seq and single-cell ATAC sequencing (scATAC-seq) on advanced human atherosclerotic plaques obtained during carotid endarterectomy and report a comprehensive overview of the various cell types in plaques and their activation status, which reveals an active, ongoing inflammation and multiple cellular interactions as well as cellular plasticity with respect to endothelial cells (EC) and macrophages. In addition, we identified cell type–specific expression of GWAS risk loci for CVD.

## Methods

In silico data analysis was performed using custom R Scripts (R version 3.5.3) designed especially for this research or based on the recommended pipelines from the preexisting packages listed in the individual segments above. R scripts are available on GitHub [https://github.com/AtheroExpress/MicroanatomyHumanPlaque_scRNAseq]. Other data is available from the corresponding authors upon reasonable request.

Please see the Data Supplement for detailed methods.

## Results

### Single-Cell RNA Sequencing Identifies 14 Distinct Cell Populations in Human Atherosclerotic Plaques

**Figure 1.**
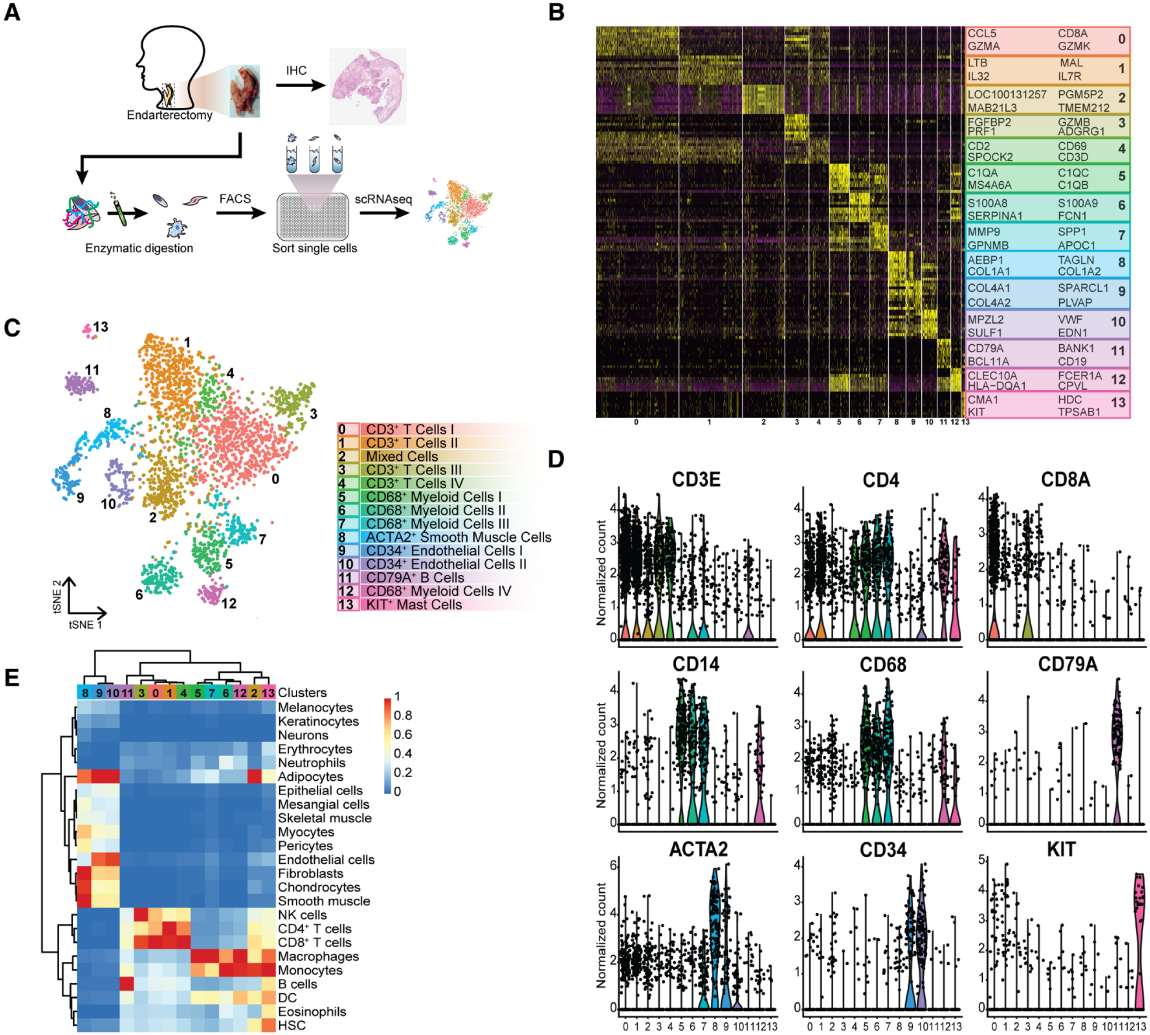
**CCA clustering and tSNE visualization revealed 14 distinct populations.** **A**, Experimental setup: plaque samples obtained from endarterectomy procedures were digested, single viable cells were fluorescence-activated cell sorting (FACS) sorted in a polymerase chain reaction plate, and CEL-seq2 was performed. **B**, Heatmap of top marker genes per cluster. **C**, tSNE visualization of clustering revealed 14 cell populations. Population identities were determined based on marker gene expression. **D**, Violin plots of signature genes confirmed population identities, as well as (**E**) by similarity to known cell type in reference RNA sequencing (RNA-seq) data sets. ACTA2 indicates alpha actin 2, smooth muscle; DC, dendritic cell; HSC, hematopoietic stem cell; IHC, immunohistochemistry; KIT, c-KIT; NK, natural killer; scRNAseq, single-cell RNA sequencing; and tSNE, t-distributed stochastic neighbor embedding.

To examine the transcriptome of human atherosclerotic plaques, carotid endarterectomy tissue from 18 patients (77% male sex) was enzymatically digested, viable nucleated cells were isolated by fluorescence-activated cell sorting (FACS; Figure 1A, Figure IA, Table I in the Data Supplement), and scRNA-seq libraries were prepared. After filtering cells based on the number of reported genes (see Methods in the Data Supplement), we applied unbiased clustering on 3282 cells, identifying 14 cell populations (Figure 1B and 1C, Table II in the Data Supplement). Correlation of our scRNA-seq data with bulk RNA-seq data (Figure IB in the Data Supplement) and examining interpatient variation of cluster distribution (Figure IC in the Data Supplement) and size (Figure ID in the Data Supplement) confirmed uniformity of the data except for patient 1. We assigned a cell type to each cluster based on differential expression of established lineage markers (Figure 1B and 1D). Cluster composition did not differ between sexes (Figure IE in the Data Supplement) and cluster identities were confirmed by correlation with bulk RNA-seq datasets (Figure 1E).^8^ We observed 3 nonimmune cell clusters (clusters 8, 9, and 10; expressing *CD34* and *ACTA2* [actin alpha 2, smooth muscle])^9,10^ and 11 leukocyte clusters (Figure 1B and 1D). The latter included 5 lymphocyte clusters (clusters 0, 1, 3, 4, and 11; expressing *CD3E*, *CD4*, *CD8*, *CD79A*),^11,12^ 5 myeloid clusters (clusters 5, 6, 7, 12 and 13; expressing *CD14*, *CD68*, *KIT*),^13–16^ and 1 cluster containing a mixture of cells (cluster 2), which did not show a clear cell type–defining expression profile but had similar gene expression levels as other clusters and seemed to mainly contain apoptotic myeloid and T cells (Figure 1B and 1D; Figure II A-D in the Data Supplement). T cells appeared to be the most abundant population in our data set, encompassing 52.4% of all analyzed cells, whereas the myeloid populations represented 18.5% of all cells (Figure IIE in the Data Supplement). Histological analysis of matched samples confirmed that CD3^+^ T cells indeed outnumbered the CD68^+^ cells, which represent macrophages and to a limited extend smooth muscle cells (SMCs),^17^ in the studied samples (number of CD3^+^ T cells: 1880±449 versus number of CD68^+^ cells: 870±135; Figure IIF in the Data Supplement).

### ECs Exhibited a Gene Expression Profile Indicative of Activation and Potential Transdifferentiation

**Figure 2.**
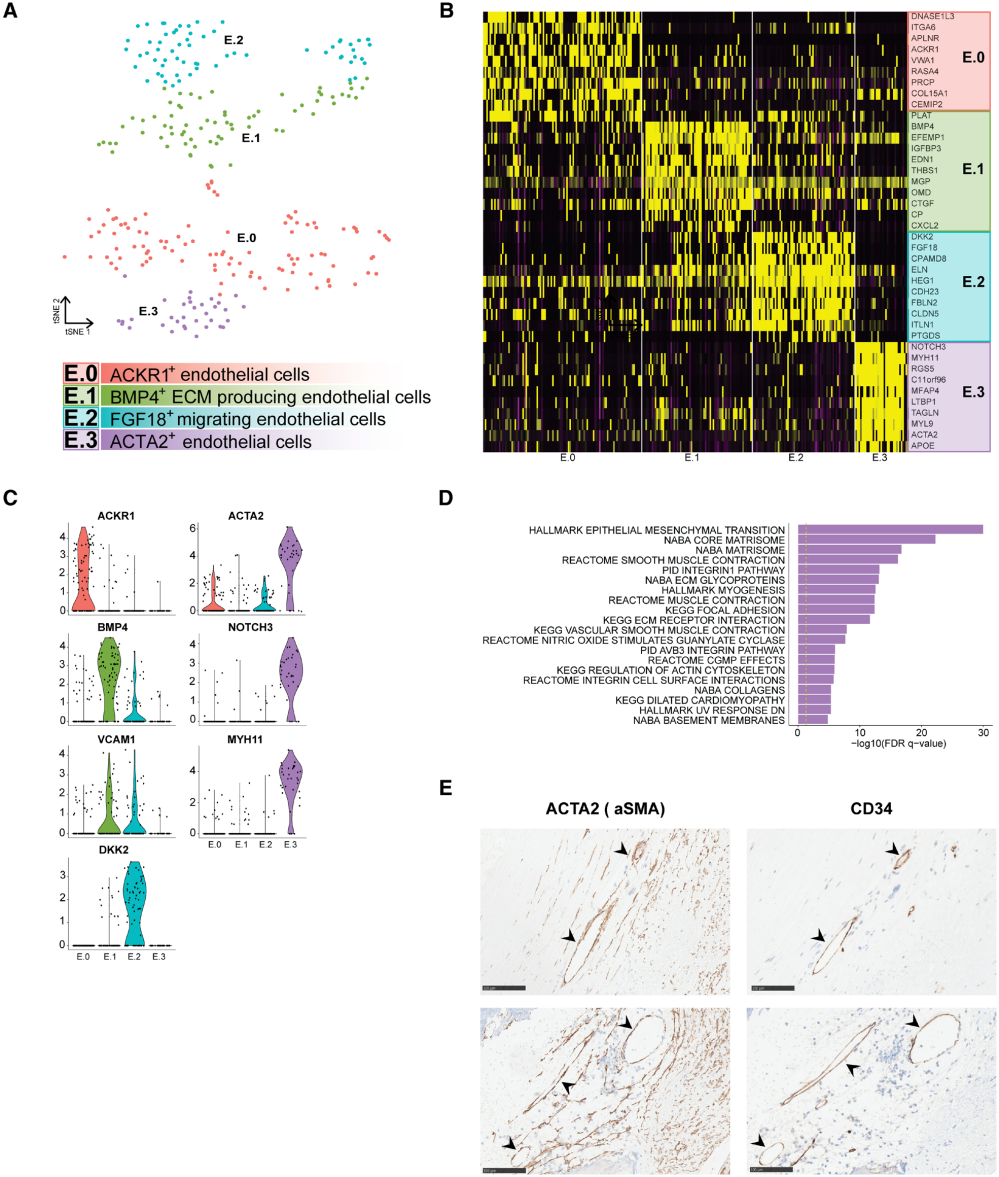
**Subclustering of endothelial cells revealed 4 distinct populations.** **A**, tSNE visualization of clustering revealed 4 distinct endothelial cell populations. **B**, Heatmap of top marker genes per cluster. **C**, Violin plots of endothelial cell-specific markers, genes involved in endothelial cell angiogenesis and activation, and genes that are associated with endothelial to mesenchymal transition. **D**, Top pathways associated with cluster E.3. **E**, Examples of ACTA2 (actin alpha 2, smooth muscle) and CD34 expression in a monolayer of cells lining intraplaque vasculature on sequential histological slides of 2 different patients. Scale bars represent 100 µm. ACKR1 indicates atypical chemokine receptor 1; ACTA2, actin alpha 2, smooth muscle; aSMA, actin alpha 2, smooth muscle; BMP4, bone morphogenetic protein 4; DKK2, dickkopf-related protein 2; E, endothelial cell; FDR, false discovery rate; FGF18, fibroblast growth factor 18; KEGG, Kyoto encyclopedia of genes and genomes; MYH11, myosin heavy chain 11; NOTCH3, notch receptor 3; tSNE, t-distributed stochastic neighbor embedding; and VCAM1, vascular cell adhesion molecule 1.

ECs were represented by cluster 9; expressing *COL4A1*, (collagen type IV alpha 1 chain) COL4A2, *SPARCL1*, and *PLVAP*, and cluster 10; expressing *MPZL2*, *SULF1*, *VWF*, and *EDN1* (Figure 1B and 1C, Table II in the Data Supplement). Isolating and reclustering these clusters revealed 4 distinct subclasses (E.0-E.3, E indicates EC, Figure 2A, Table II in the Data Supplement). We could assign EC phenotypes to the subclasses by assessing marker genes (Figure 2B). E.0, E.1, and E.2 displayed classical endothelial markers *CD34* and *PECAM1*, and the vascular endothelial marker *TIE1*. E.0 showed distinct expression of *ACKR1*, which has been associated with venous ECs and the vasa vasorum in mice^18,19^ and *PRCP*,^20^ involved in angiogenesis and regeneration of damaged endothelium (Figure 2B and 2C). E.1 and E.2 separated on expression of extracellular matrix genes in E.1 and cell mobility markers *FGF18* and *HEG1* in E.2. Both populations expressed *VCAM1* (Figure 2C), which is expressed by activated endothelium and facilitates adhesion and transmigration of leukocytes, such as monocytes and T cells.^21^ Together, this suggests that E.0, E.1, and E.2 represent activated endothelium which actively aggravates inflammation in the advanced lesion by cell adhesion and neovascularization and mediating leukocyte extravasation.^22^ Of note, subclass E.3 expressed typical SMC markers, such as *ACTA2*, *NOTCH3*, and *MYH11*, next to the aforementioned endothelial markers (Figure 2C). This, combined with its clustering among the EC clusters and enrichment of transitory and SMC-related pathways (Figure 2D), indicated that this subset may be undergoing endothelial to mesenchymal transition or vice-versa. To validate these findings, we looked into the expression of ACTA2 and CD34 on sequential histological slides. Figure 2E shows cells lining the intraplaque vasculature that shows overlapping expression.

### Synthetic Phenotype Dominates in Plaque Smooth Muscle Cells

SMCs were represented by cluster 8, expressing *MYH11*, *PDGFRB*, *NOTCH3*, and *MFAP4*^23–25^ (Figure 1B and 1C, Table II in the Data Supplement), which separated into 2 subclasses (Figure IIIA in the Data Supplement): a cluster of SMCs with contractile characteristics (cluster S.1; expressing *MYH11*, *ACTA2* and *TAGLN*) and a cluster of synthetic-like SMCs (cluster S.0; expressing *COL1A1*, *MGP* and *COL3A1*)^26^ (Figure IIIB and IIIC and Table II in the Data Supplement). The low expression of typical SMC markers in cluster S.0 and upregulation of extracellular matrix genes suggested that a subset of these cells were derived from the established cap portion of the plaque. A limited number of cells within this cluster was *KLF4*^+^ (Figure IIID in the Data Supplement), indicative of differentiation from vascular smooth muscle cells into either a synthetic or macrophage-like phenotype.

### Intraplaque T Cells Are Defined by Activation Status

**Figure 3.**
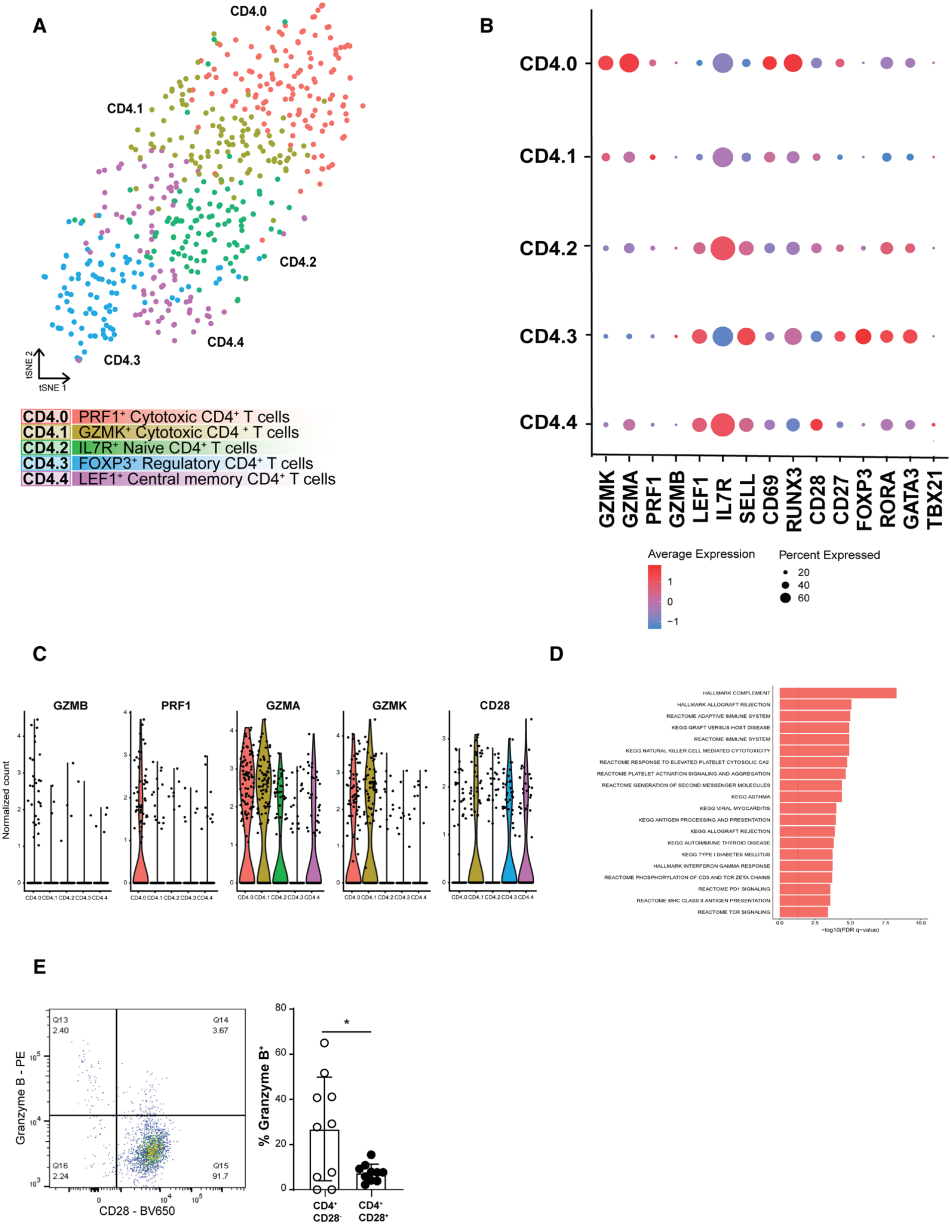
**Subclustering of CD4^+^ T cells revealed 5 distinct populations.** **A**, tSNE visualization of clustering revealed 5 distinct CD4^+^ T-cell populations. **B**, Dot plot of cluster-identifying genes and T-cell transcription factors. **C**, Violin plots of CD4.0 characterizing cytotoxic genes. **D**, Flow cytometry analysis of Granzyme B production by CD4^+^CD28^−^ cells on defrosted plaque samples. **E**, Top pathways associated with cluster CD4.0. Data shown as mean±SD (n=10; obtained from cohort 1 and 2). *P<0.05. FDR indicates false discovery rate; FOXP3, forkhead box P3; GZMK, granzyme K; IL7R, interleukin 7 receptor; KEGG, Kyoto encyclopedia of genes and genomes; LEF1, lymphoid enhancer-binding factor 1; PD1, programmed cell death protein 1;PRF1, perforin 1; TCR, T cell receptor; and tSNE, t-distributed stochastic neighbor embedding.

Lymphocyte clusters consisted of one small, but homogenous cluster of B cells (cluster 11; expressing *CD79A*, *FCER2*, *CD22*, and *CD79B*)^27–29^ (Figure 1B and 1C, Table II in the Data Supplement), and 4 T-cell clusters. To define the T cells in more detail, we assessed the CD4^+^ T cells (expression *CD4*>*CD8*) and the CD8^+^ T cells (expression *CD8*>*CD4*) from *CD3* enriched clusters 0, 1, 3, and 4. Isolating and reclustering the CD4^+^ T cells revealed 5 subclasses (CD4.0–CD4.4, Figure 3A, Table II in the Data Supplement) of which the primary difference was their activation state rather than the transcription factors and cytokines commonly used to define CD4^+^ T-helper (T_H_) subsets (Figure 3B and 3C). CD4.0 and CD4.1 exerted a cytotoxic gene expression profile exemplified by expression of *GZMA*, *GZMK*, and *PRF1*. Apart from these cytotoxic transcripts, cells in CD4.0 also showed very little *CD28* expression and some *GZMB* expression, suggesting that these cells are cytotoxic CD4^+^CD28^null^ cells that have previously been correlated with unstable angina and increased risk of Major Adverse Cardiovascular Events.^30,31^ In addition, gene expression in this cluster confirmed an enrichment in proinflammatory pathways associated with adaptive immune responses (Figure 3D). Using flow cytometry, we confirmed the cytotoxic character of the CD4^+^CD28^null^ cells, which showed that significantly more CD4^+^CD28^−^ cells contained granzyme B as compared to the CD4^+^CD28^+^ cells (Figure 3E, Figure IVA in the Data Supplement). CD4.2 and CD4.4 were characterized by expression of *IL7R* (interleukin 7 receptor), *LEF1*, and *SELL*, associated with a naïve and central-memory phenotype. The final CD4^+^ subclass (CD4.3) was identified as a regulatory T-cell cluster based on the expression of the classical markers *FOXP3* (forkhead box P3), *IL2RA* (CD25), and *CTLA4*^32^ (Figure 3B, Table II in the Data Supplement). Interestingly, we also found some coexpression of FOXP3 with transcription factors RORA (RAR Related Orphan Receptor A) and GATA3 (GATA binding protein 3) in this cluster (Figure IVB in the Data Supplement), which has, respectively, been associated with the enhanced immunosuppressive function of regulatory T cells^33^ and with the prevention of polarization towards other T_H_ subsets.^34^ Expression of the T_H_ cell subset–specific transcription factors *TBX21* (Tbet [T-box transcription factor 21]; Th1), *GATA3* (Th2), and *RORC* (RORγT; Th17) was not linked to a specific cluster (Figure IVC in the Data Supplement), which seems to be a common phenomenon when dealing with T-cell scRNA-seq data.^35,36^ By analyzing the CD4^+^ T cells in a clustering-independent method by selecting all cells that have the expression of both CD3E and CD4 and subsequently analyzing the expression of single T_H_-specific transcription factors, we find that a large population of T cells did not express a clear signal of the transcription factors (Figure IVD in the Data Supplement).

Analysis of CD8^+^ T cells revealed 3 subclasses (Figure VA and VB in the Data Supplement), which were similar to CD4^+^ T cells defined by differences in activation state. CD8.0 was identified as an effector-memory subset, characterized by expression of *GZMK*, *GZMA*, and *CD69*, indicating recent T-cell receptor activity (Figure VC in the Data Supplement). A clear, terminally differentiated, cytotoxic CD8^+^ T-cell profile was observed in CD8.1, which showed expression of *GZMB*, *TBX21*, *NKG7*, *GNLY*, *ZNF683*, and *CX3CR1*, and in line, this subclass lacked *CD69* expression. Finally, CD8.2 displayed a quiescent, central-memory CD8^+^ T-cell phenotype with expression of *LEF1*, *SELL*, *IL7R*, and *LTB*. In contrast to Fernandez et al^7^ and previous scRNA-seq data obtained from various cancers, we did not detect a clear exhausted phenotype in the CD8^+^ T cells.^35–37^ The CD8 clusters with reduced cytotoxic potential show expression of *CD69*, suggesting recent T cell receptor (TCR) activation and it will be of future interest to examine how these CD8^+^ populations were activated and how they affect the pathogenesis of atherosclerosis. This could indicate that not the cytotoxic but the more quiescent CD8^+^ T-cell subsets are responding to plaque-specific antigens and may be more relevant in the pathogenesis of atherosclerosis. Using experimental mouse models of atherosclerosis it has been shown that the majority of CD8^+^ T cells in the plaque are antigen-specific,^38^ but so far little is known regarding the plaque-antigen(s) they respond to. Whereas CD4^+^ T cells have been shown to respond to (ox)LDL ([oxidized] low-density lipoprotein) and its related apo B_100_ peptide, plaque-antigen(s) for CD8^+^ remain mostly indefinable.^39^ Therefore, we are unable to define which antigens have activated the T cells in the atherosclerotic lesion.

### Both Proinflammatory and Anti-inflammatory Macrophage Populations Reside in the Plaque

**Figure 4.**
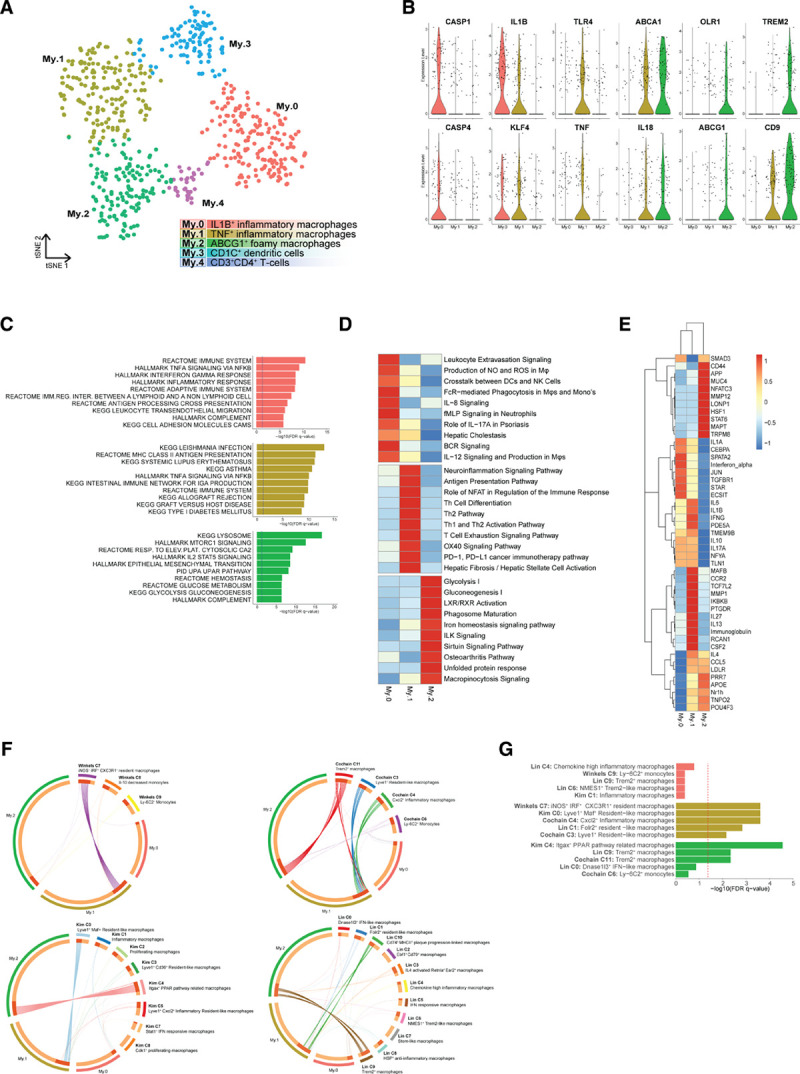
**Subclustering of myeloid cells revealed 5 distinct populations.** **A**, tSNE visualization of clustering revealed 5 distinct myeloid populations. **B**, Violin plots of macrophage-specific activation genes and foam cell markers. **C**, Top pathways associated with the macrophage clusters. **D**, Unique pathways per macrophage cluster. **E**, Ingenuity Pathway Analysis of upstream regulators of the macrophage subsets. Both **D** and **E** depict data as *Z* score. **F**, Circos plots displaying overlap of macrophage clusters with macrophage clusters of murine single-cell RNA sequencing papers. Dotted lines indicate no significant overlap, and solid lines indicate significant overlap. **G**, Bar graph with top 5 overlapping clusters of human and murine macrophage clusters. ABCG1 indicates ATP-binding cassette sub-family G member 1; BCR, B cell receptor; CXCL2, C-X-C motif chemokine ligand 2; DC, dendritic cell; FcR, Fc receptor; FDR, false discovery rate; IL, interleukin; ILK, integrin-linked kinase; iNOS, inducible nitric oxide synthase; KEGG, Kyoto encyclopedia of genes and genomes; LXR, liver X receptor; My, myeloid cells; NK, natural killer; PD1, programmed cell death protein 1; PPAR, peroxisome proliferator-activated receptor; ROS, reactive oxygen species; TH, T helper; TNF, tumor necrosis factor; TREM2, triggering receptor expressed on myeloid cells 2; tSNE, t-distributed stochastic neighbor embedding; and RXR, retinoid X receptor.

Atherosclerotic myeloid cells were represented by 5 clusters. A small, distinct mast cell population was defined by expression of *HDC*, *KIT*, *CMA1*, and *TPSAB1*.^40^ The remaining myeloid clusters, cluster 6, 7, 8, and 12, expressed *CD14* and *CD68* (Figure 1B and 1C) and isolating and reclustering of these cells revealed 5 distinct phenotypes (My.0–My.4 [myeloid cell], Figure 4A, Figure VIA and Table II in the Data Supplement).

My.0, My.1, and My.2 most likely represented different macrophage activation states. Enrichment of proinflammatory marker genes (Figure 4B) and immune and inflammatory pathways (Figure 4C) indicated that subclasses My.0 and My.1 consisted of proinflammatory macrophages. My.0 showed characteristics of recently recruited macrophages (leukocyte transendothelial migration, Figure 4C and leukocyte extravasation signaling, Figure 4D) displaying inflammasome activation based on coexpression of *IL1B*, *CASP1*, and *CASP4* (Figure 4B, Figure VIB in the Data Supplement). My.1 represented macrophages that differentially expressed tumor necrosis factor (*TNF*) and toll-like receptors (Figure 4B). Interestingly, both My.0 and My.1 expressed *KLF4* (kruppel like factor 4), albeit at a low level, which is known to drive macrophages towards an anti-inflammatory phenotype by repressing the NF-κB (nuclear factor κB) gene program.^41^ Our data may suggest that an inhibitory feedback loop in the proinflammatory macrophage populations is actively mediated by KLF4 expression.

In contrast to My.0 and My.1, My.2 showed absence of clear proinflammatory markers and showed signs of macrophages and foam cells. It expressed foam cell marker genes *ABCA1*,^42^ *ABCG1*, *MMP9*, and *OLR1*^43^ (Figure 4B, Figure VIA in the Data Supplement), profibrotic markers, such as *TREM2* (triggering receptor expressed on myeloid cells 2) and *CD9*,^44,45^ and the enrichment of metabolic pathways hinted at a shift in metabolism (Figure 4C, bottom). Interestingly, My.2 cells expressed smooth muscle actin (*ACTA2*), a hallmark of smooth muscle cells, in combination with macrophage markers such as *LGALS3* and *CD68* (Figure VIC and VID in the Data Supplement). Expression of myeloid lineage TFs (transcription factors) PU.1 (SPI1) and C/EBPß (CEBPB [CCAAT enhancer binding protein beta])^46^ and absence of SMC lineage TFs MYOCD (myocardin) and MRTF-A (MRTFA [myocardin related transcription factor A])^47^ in specifically the ACTA2^+^ cells of My.2 suggests that part of the My.2 myeloid cells gained characteristics of SMCs rather than that it originated from SMCs^17^ (Figure VIE in the Data Supplement).

To further characterize the 3 subclasses, we next examined pathways differentially enriched per population (Figure 4D) as well as the upstream regulators that possibly govern these populations by Ingenuity Pathway Analysis (Figure 4E). My.0 and My.1 showed enrichment for classical inflammatory and immune pathways clearly suggesting cellular activation, recruitment, and immune cell interactions driving their phenotype. In line, Ingenuity Pathway Analysis predicted that My.0 and My.1 are mainly controlled by proinflammatory factors, such as IL1A, IFN (interferon) A, IFNG, and IL1B.

My.2 was enriched for metabolic pathways and LXR/RXR (liver X receptor/retinoid X receptor) activation, consistent with a foamy phenotype. Hence, this cluster was uniquely driven by anti-inflammatory pathways such as STAT6 (signal transducer and activator of transcription 6) and had typical foam cell-related factors including APOE and the LXR family (Nr1h [nuclear receptor subfamily 1 group H]: NR1H2,3,4), which interestingly showed some overlap with My.1. The latter may indicate that unlike the more recently recruited My.0, My.1 cells are gaining foamy characteristics.

My.3 is characterized by dendritic cell markers, such as *CD1C*, *CLEC10A*, and *FCER1A* (Figure VIA in the Data Supplement) and this population most likely represents CD1c^+^ dendritic cells.^13,48,49^ In line with their dendritic cell phenotype, this cluster showed the highest expression of multiple class II HLA genes indicative of their enhanced activation status as a consequence of antigen-specific interaction with plaque T cells (Figure VIF in the Data Supplement). Cluster My.4 expressed *CD3E*, *GNLY*, *FOXP3*, and *CD2*, suggesting that My.4 potentially contains regulatory T cells. This misclustering might be a result of comparable CD4 expression levels in T cells and macrophages because myeloid cells frequently expressed CD4 (Figure IE in the Data Supplement).

Finally, we compared our macrophage subclasses with monocyte and macrophage populations from 4 recent articles on scRNA-seq analysis of atherosclerotic plaques in mice.^3–6^ Eight mouse populations showed significant overlap with our human subclasses (Figure 4F). My.0 showed no statistically significant overlap, but most resembled inflammatory mouse macrophages (Figure 4G). My.1 resembled inflammatory, resident-like mouse macrophages, and My.2 overlapped with foamy, anti-inflammatory, Trem2^+^ macrophages. Together, this confirms the recently migrated and embedded inflammatory phenotypes we defined, respectively, for My.0 and My.1 and matches the foamy phenotype we saw in My.2. It also showcases a decent concordance between human patients and mouse models in relation to cell type diversity.

### Intercellular Communication Drives Inflammation Within the Plaque

**Figure 5.**
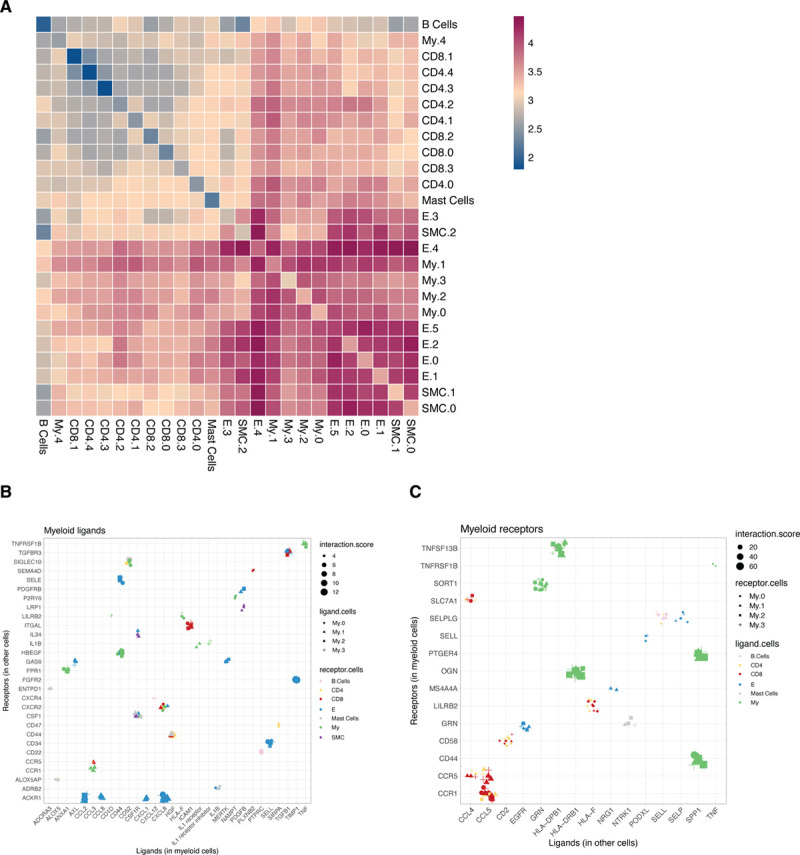
**Ligand-receptor interaction analyses to assess intracellular communication in the plaque.** **A**, Heatmap showing logarithmic interaction scores between all cell subsets. Top quartile of unique ligand-receptor interactions between all cells and myeloid cells for both (**B**) ligands expressed by myeloid cells and (**C**) receptors expressed by myeloid cells. E indicates endothelial cells; My, myeloid cells; and SMC, smooth muscle cells.

We next examined potential ligand-receptor interactions between cell types to predict intercellular communication within the lesion based on CellPhone DB v2.0.^50^ Lymphocytes and mast cells showed the lowest absolute numbers of potential interactions while myeloid, endothelial, and SMCs displayed higher numbers of interactions (Figure 5A). The low interaction between myeloid and T cells may be a consequence of the apparent lack of detection of TCR-related genes (*TRA, TRB, TRG*) in our scRNA-seq dataset and the fact that CD4-class II and CD8-class I interactions are not included in this database.

Subsequently, we specifically examined the top unique interactions within the myeloid populations, split by myeloid ligands (Figure 5B) and receptors (Figure 5C). We found multiple chemotactic interactions, including endothelial *ACKR1*^51^ with myeloid-derived *CCL2*, CXCL8, *CCL8*, and *CXCL1*, of which the last 2 ligands were specifically expressed in My.1. We also observed an interaction between *CSF1R* on all myeloid subsets and *CSF1* on ECs, smooth muscle cells, mast cells, and myeloid cells. *CCR1* (C-C motif chemokine receptor 1) and *CCR5* interacted with CCL5 from both CD4^+^ and CD8^+^ T cells and *CXCR4* on B cells interacted with *CXCL12* on My.1 cells. In addition, we identified communication patterns that are potentially involved in extravasation of myeloid cells, including *CD44* (My)–*SELE* (EC), *SELL* (My)–*CD34* (EC), *SELPLG* (My)–*SELP*, and *SELL* (both EC). Myeloid cells showed potential capability to attract other leukocytes, for example CCR5^+^ T cells through expression of *CCL3* (My.1). Moreover, myeloid cells were also predicted to interact with T cells leading to mutual activation, through *SIRPA* (My)–*CD47* (T)^52^ *ICAM1* (My)–*ITGAL* (CD8), inducing cytotoxicity and multiple interactions involved in antigen presentation. Lastly, interaction of *PDGFB* on myeloid subsets with *PDGFBR* on ECs suggest a possible myeloid-driven induction of angiogenesis, which has been associated with plaque destabilization.^53,54^

### Chromatin Accessibility of Myeloid and T-Cell Populations Reveals Transcription Factors Involved in Gene Regulation

**Figure 6.**
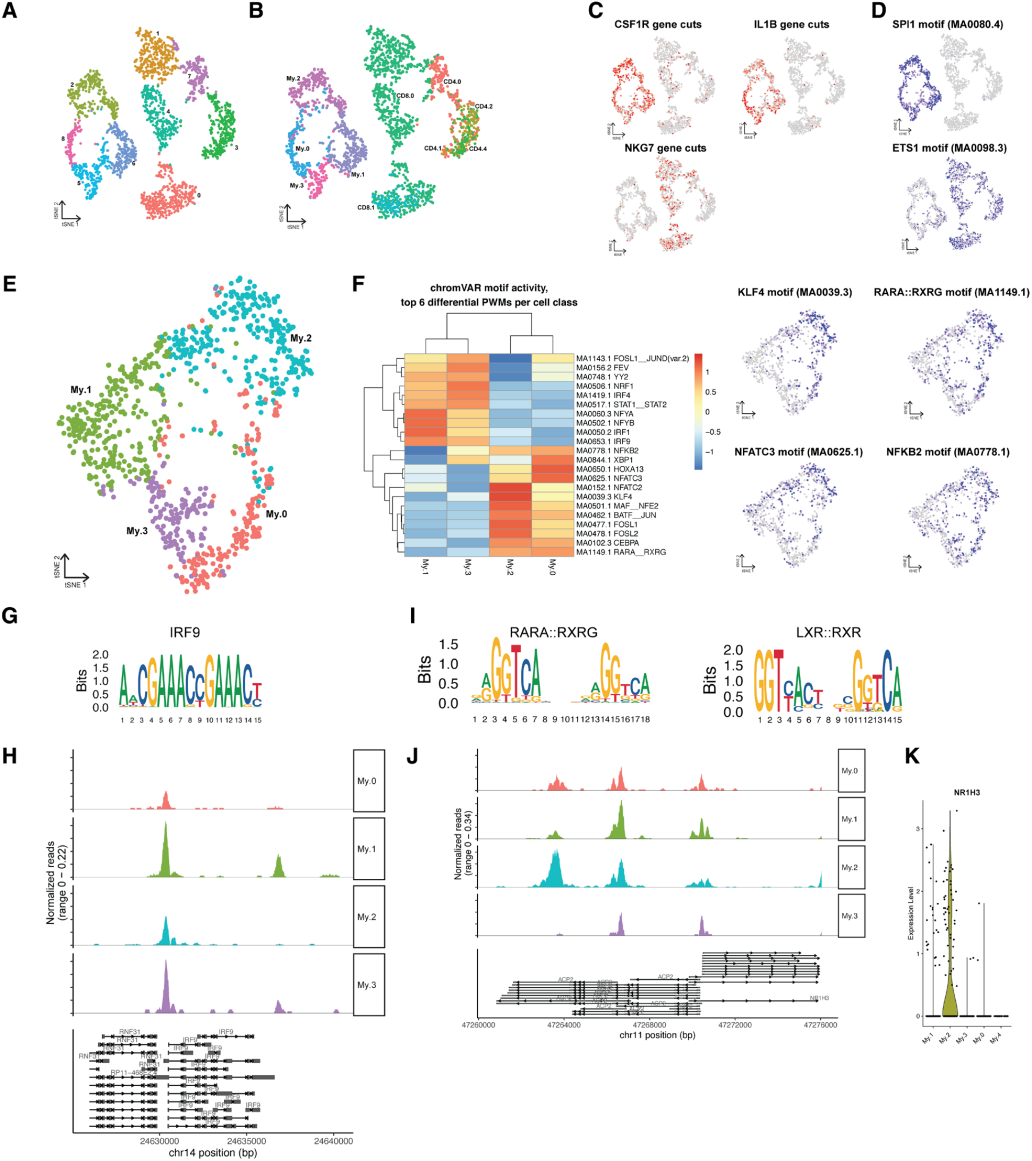
**Chromatin accessibility of myeloid cells in human atherosclerotic plaques analyzed using single-cell ATAC sequencing (scATAC-seq).** **A**, tSNE visualization of myeloid and T-cell clusters based on scATAC-seq. **B**, Projection of single-cell RNA sequencing (scRNA-seq) myeloid and T-cell labels over the scATAC-seq clusters. **C**, tSNE visualization of cell type–specific accessible gene loci. **D**, tSNE visualization of cell type–specific transcription factor motifs enriched in open chromatin regions. **E**, tSNE visualization of subclustered scATAC-seq myeloid clusters. **F**, Heatmap showing the top differential open chromatin TF motifs by chromVAR, with subcluster specific accessible TF motifs visualized as tSNE. **G**, IRF9 motif. **H**, Pseudobulk genome browser visualization identifying the open chromatin regions of *IRF9* in different myeloid subsets. **I**, RARA:RXRG, and LXR (liver X receptor): RXR (retinoid X receptor) motifs. **J**, Pseudobulk genome browser visualization identifying open chromatin regions of *NRIH3* (encoding LXRα) in different myeloid subsets. **K**, Violin plot of *NR1H3* (nuclear receptor subfamily 1 group H) gene expression from myeloid scRNA-seq data. ETS1 indicates ETS proto-oncogene 1; IRF, interferon regulatory factor; KLF, kruppel like factor 4; NFATC3, nuclear factor of activated T cells 3; NR1H3, nuclear receptor subfamily 1 group H member 3; PWM, position weight matrix; tSNE, t-distributed stochastic neighbor embedding; RARA, retinoic acid receptor alpha; and RXRG, retinoid X receptor gamma.

Next, we aimed to further define the genomic landscape that accounts for the obtained cluster-specific patterns of gene expression and potentially uncover disease driving transcription factors. Using scATAC-seq, we examined the open chromatin promoter and enhancer landscape of myeloid and T cells in human plaques. We identified 4 myeloid and 5 T cell clusters by scATAC-seq. Population label transfer from scRNA-seq to scATAC-seq populations showed good agreement with the native scATAC-seq cluster borders and retrieved the majority of the scRNA-seq populations (Figure 6A and 6B). Open chromatin at macrophage (*CSF1R*, *IL1B*) and T cell–specific genes (*NKG7*), as well as enrichment of motifs of cell type TFs for macrophages and T cells (*SPI1*^55^ and *ETS1*^56^), confirmed the delineation between cell types (Figure 6C and 6D). Transferred myeloid populations were reclustered analogous to the scRNA-seq clusters (Figure 6E).

IRF4 has been shown to be a CD1c^+^ dendritic cell-specific transcriptional regulator^57^ and its motif was indeed enriched in My.3 (Figure 6F). In line, we found specific open chromatin at the promoter region of *IL12A*, the subunit that is specific for the cytokine IL12, in all myeloid populations and an enhancer specifically in My.3 dendritic cells (Figure VIIE in the Data Supplement). IL12 is required to induce a proinflammatory, T_H_1-like cytotoxic phenotype of T cells and actively induces atherosclerosis.^58,59^ Potentially, as a result of the My.3-specific IL12, we observed open chromatin at the *IFNG* and *TNF* loci in CD4.0, confirming its activated, cytotoxic phenotype and suggesting that this cluster has T_H_1-like properties (Figure VIIG and VIIH in the Data Supplement). Additionally enriched accessible motifs within the T cells (Figure VIIA and VIIB in the Data Supplement) were observed for the *RUNX3* motif in CD4.0, normally a CD8^+^ T-cell linage specific TF that is also known to induce expression of cytotoxic genes in CD4^+^ T cells,^60–63^ as well as the *STAT3* motif, which is downstream of IL6 and IL2 signaling. The BATF_JUN motif (Figure VIIC in the Data Supplement) that is known to be critical for effector function in T cells was also enriched in this cluster.^64^ The effector function could be further confirmed by differential open chromatin of the *GZMB* and *GZMH* loci in both CD4.0 and all CD8 clusters (Figure VIID in the Data Supplement) and an open locus at *IL2* in CD4.0, CD4.1, and CD8.0.

In line with the scRNA-seq data, My.1 showed enrichment of proinflammatory TF motifs (Figure 6F), which matches the proinflammatory gene expression seen in these cells. This cluster was especially enriched in INF signaling induced TFs including IRF1 (interferon regulatory factor 1), IRF9, STAT1, and STAT2. The STAT1-STAT2 complex is known to interact with IRF9 upon IFNγ stimulation and hence induces the upregulation of proinflammatory cytokines as TNF, indicating an IFNγ pathway driven activation, possibly secreted by the T cells.^65^ Indeed, the IRF9 motif was accessible and the *IRF9* locus was open predominantly in My.1 cells (Figure 6G and 6H). Moreover, these IRF and STAT TFs are also key mediators of type I IFN responses which have previously been shown to associate with atherosclerotic disease as well.^66^

My.0 cells were specifically enriched for the NFATC3 (nuclear factor of activated T cells 3) motif (Figure 6F), a TF that has previously been linked to activated TLR-pathway signaling and has been shown to partially regulate subsequent TNFα and IL-1ß secretion.^67,68^ Finally, My.2 cells were enriched for anti-inflammatory, foam cell–associated TFs in the scATAC-seq data similar as in the scRNA-seq data. We observed increased chromatin accessibility at loci harboring the KLF4 motif, which next to repressing proinflammatory programs was shown to implement an anti-inflammatory macrophage activation state and is also known to be involved in the transformation of vascular SMCs to macrophages^41,46^ (Figure 6F). This is in contrast with the scRNA-seq data where KLF4 was expressed at a low level, indicating that while the KLF4 locus is poised, its associated gene program is not necessarily executed in all foamy macrophages. Furthermore, My.2 was enriched for the de novo motif MA1149.1, which was annotated to RAR_RXR, a motif with high similarity to the LXR_RXR motif (Figure 6I). Moreover, LXR_RXR motif accessibility is enriched in My.2 cells and the *NR1H3* (LXRα) locus is opened specifically in the My.2 population (Figure 6J). In line, the scRNA-seq data likewise shows *NR1H3* upregulation specifically in My.2 (Figure 6K).

We could not map the regulatory T-cell cluster CD4.3 to a scATAC-seq cluster. The *FOXP3* locus hardly showed open chromatin in any population in the scATAC-seq data set and neither did the Treg-associated cytokine gene *IL10* (Figure VIII and VIIJ in the Data Supplement).

### Cell Type–Specific Enrichment of Genes in GWAS Loci.

**Figure 7.**
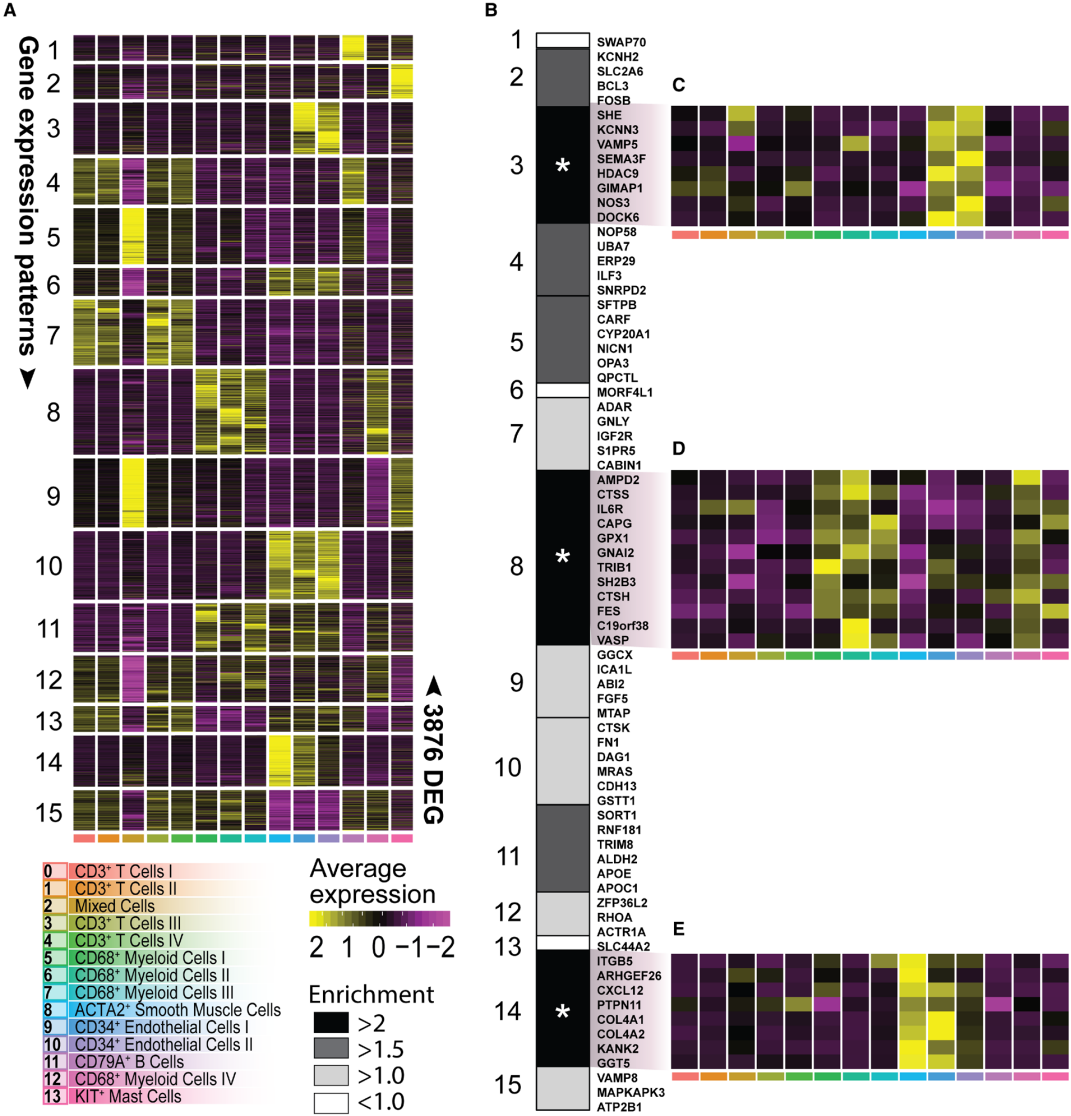
**Projection of coronary artery disease (CAD) genome-wide association studies (GWAS)–associated genes.** **A**, Heatmap of average expression of 3876 differentially expressed genes (DEGs) divided into 15 gene expression patters that best-matched cluster or cell type identity (for DEG selection, see Figure VIII in the Data Supplement). **B**, Enrichment of 74 CAD GWAS associated genes across the 15 gene expression patterns. **C**, **D**, and **E**, Heatmap of average relative expression of significantly enriched CAD genes from gene expression pattern no. 3, no. 8, and no. 14, respectively. Asterisk indicates significant enrichment. *P<0.05. ACTA2 indicates actin alpha 2, smooth muscle; and KIT, c-KIT.

GWAS have discovered 163 genetic susceptibility loci linked to coronary artery disease (CAD) through literature search and effects on expression.^69^ However, the challenge remains in identifying the potential causal genes linked to these loci for functional testing as novel therapeutic targets. In part, this is due to the underlying genetic architecture where multiple causative variants in a gene might be involved and variants in linkage disequilibrium only show marginal significance in a GWAS. Another reason is that many of the risk variants are not causal and ambiguously linked to genes. A gene-centric analysis considers all variants in a gene and solves these issues, yet such analyses fail to identify the cells potentially involved. Here, we aimed to (1) identify genes associated with CAD that are (2) also highly expressed in specific cell types, effectively identifying tangible candidates for functional follow-up. To this end, we mapped genes near GWAS loci associated with CAD and assessed expression of these genes across our scRNA-seq cell populations to investigate their expression in disease-relevant tissue. We prioritized 317 protein-coding genes based on the summary statistics of a recent CAD GWAS^70^ (see Methods and Table III in the Data Supplement). Next, we selected the genes that would best represent each individual cell population. To achieve this, we determined differentially expressed genes (Figure VIII and Methods in the Data Supplement). Three thousand eight hundred seventy-six genes were differentially expressed and differentially expressed genes were grouped into 15 gene expression patterns that best matched the scRNA-seq populations (Figure 7A). We overlapped the 317 CAD-associated genes with the 3876 differentially expressed genes, resulting in a significant overlap (permutation over random data *P*=2.67×10^-5^) of 74 genes. These genes are distributed over multiple individual CAD loci (Table III in the Data Supplement), indicating that our methods are robust. We observed a significant accumulation of GWAS linked genes in patterns 3, 8, and 14 (permutation over random data *P*=0.015, *P*=0.006, and *P*=0.015, respectively; Figure 7B). Genes in pattern 3 are associated with higher expression in the EC clusters 9 and 10 (Figure 7C) and consisted of *SHE*, *KCNN3*, *VAMP5*, *SEMA3F*, *HDAC9*, *GIMAP1*, *NOS3*, and *DOCK6*. Pattern 8 is hallmarked by gene expression associated with all 4 macrophage populations (Figure 7A) and contained *AMPD2*, *CTSS*, *IL6R*, *CAPG*, *GPX1*, *GNAI2*, *TRIB1*, *SH2B3*, *FES*, *C19orf38*, and *VASP* (Figure 7B and 7D). Genes in pattern 14 were predominantly associated with higher expression in both the smooth muscle cell population 8 and the *CD34*^+^ EC population 10 (Figure 7E). This pattern contained *ITGB*, *ARHGEF26*, *CXCL12*, *PTPN11*, *COL4A1*, *COL4A2*, *KANK2*, and *GGT5*. Our results suggest that macrophages, smooth muscle cells, and ECs are of particular interest as a starting point for functional testing.

Furthermore, given that for many of the genes previously mapped to the 163 CAD loci the mechanisms and cellular expression are still unknown,^69^ we examine whether these genes show cell type–specific expression in carotid plaques. We found that 24 of the 75 genes previously classified as unknown by Erdmann et al.^69^ were differentially expressed between cell populations in carotid plaques, and included 3 genes that also showed association with CAD (*CHD13*, *SNRPD2*, and *ARHGEF26*; Table III in the Data Supplement) in our analysis.

## Discussion

In the past 2 years, single-cell technologies have advanced our knowledge of atherosclerosis tremendously. scRNA-seq has been applied to specifically describe the immune cell landscape of murine and human atherosclerotic lesions.^3–7^ The recent study by Fernandez et al^7^ gave a first overview of the human immune cell landscape during atherosclerosis by showing a data set based on extensive cytometry by time of flight analyses and by comparing RNA expression profiles of T cells and macrophages in plaque and blood of symptomatic and asymptomatic patients.^7^ They provide insight into which immune cells reside in the plaque and described their different activation states. Yet, both the mouse and human studies lack coverage of nonimmune cell types in the plaque and so far only a limited number of patients have been included in the scRNA-seq studies. Here, we applied scRNA-seq to all live cells in advanced human atherosclerotic plaques of 18 patients and revealed a highly diverse cellular landscape consisting of 14 main cell populations.

We detected a predominance of T cells in the leukocyte population of the human lesions. In contrast, murine scRNA-seq studies describe a more prominent presence of myeloid cells, which may be caused by the previously described declining myeloid content upon progression of human atherosclerotic plaques, whereas T cells reciprocally increase in human atherosclerosis.^71,72^ Both CD4^+^ and CD8^+^ T-cell subsets were characterized by their activation state, rather than classical T_H_ or T_C_ subclasses. We could confirm the presence of activated T cells that in the plaque could especially be characterized by the expression of multiple granzymes.^7^ In addition, we show that these granzymes are not only expressed by CD8^+^ T cells but also by a substantial number of CD4^+^ T cells in the plaque. The CD4^+^ T cells showed a dominant cytotoxic T-cell pool, characterized by expression of *PRF1* and multiple granzymes, with granzyme B production confirmed by flow cytometry. The lack of *CD28* expression in these cells indicates that this pool constitutes most likely a subset of cytotoxic CD4^+^CD28^null^ T cells, which has previously been associated with atherosclerosis as they have been detected in peripheral blood of patients with CAD.^30,73^ Although the presence of a similar TCR clone as observed in peripheral CD4^+^CD28^null^ cells was found in bulk coronary artery tissue,^30,73^ we can now confirm the presence of these cells on a single-cell level suggesting a functional role in patients with CVD. As cytokine expression could not be retrieved from the scRNA-seq data, but we were able to detect open chromatin at various cytokine gene loci within the T-cell populations using scATAC-seq suggesting active cytokine genes. Among others, *IFNG* showed open chromatin in the cytotoxic and effector T-cell subclasses. Apart from confirming the cytotoxic, T_H_1-like phenotype within the plaque, this also suggests that the proinflammatory macrophage subclasses we observe in our dataset may be primed for classical activation by secretion of IFNγ by the T cells.^74^ These T_H_1 cells acting on macrophages may, in turn, be driven by activated CD1c^+^ dendritic cells that were characterized by an active *IL12* gene (ie, open enhancer), which has previously been found on protein level in plaque lysates^75,76^ and the enrichment of HLA-DR (major histocompability complex, class II, DR beta 1) subtypes.^49,77^

Each of the macrophage clusters seemed to have been activated differently, one expressing *TNF* and *TLR4*, which can be activated by oxLDL and IFNγ,^78^ as well as IL1B, and the other more selectively expressing *IL1B*, which correlated with caspase expression suggesting inflammasome activation.^79^ The recent CANTOS trial (Canakinumab Anti-Inflammatory Thrombosis Outcome Trial), which targeted IL-1β,^80^ might thus have been effective through impacting on the proinflammatory capacity of the My.0 and My.1 populations within the plaque. Chromatin accessibility confirmed the proinflammatory phenotype of these cells by showing open regions linked to inflammatory transcription factors. Particularly the TNF enriched macrophage cluster My.1 was enriched for motifs from IFN induced TFs (eg, STATs, IRFs), which correlated with our upstream regulator analysis that suggested IFN as drivers of the macrophage phenotype and may be a result of the local IFNγ production by T cells. ln line, the My.0 and My.1 populations correlated with inflammatory and resident-like macrophages as detected in murine lesions.^3–6^

The IL12-IFNγ axis, as found in our scRNA-seq, data may form an important feature of T-cell activation in the plaque, and subsequent activation of myeloid cells contributes to the inflammation profile within the plaque. This is in line with several experimental studies that show the proatherogenic role of both IL12 and IFNγ in cardiovascular disease.^59,81,82^

The more anti-inflammatory foam cell–like cluster was characterized by expression of ABC cholesterol efflux transporters and lipid-related genes whose expression is most likely driven by intracellular lipid accumulation.^83^ The lipid-phenotype was confirmed by the enriched LXR_RXR TF motifs in the scATAC-seq data. LXR is a well-known nuclear receptor, active in foam cells and inducing ABC transporters.^46,84^ The notice that foam cell formation per se is not proinflammatory is a recent ongoing paradigm shift in the field. Several studies have previously shown clear proinflammatory characteristics of foam cell formation either through engagement of TLRs by oxLDL,^85–87^ induction of oxidative responses,^88^ or through other pathways.^89–91^ However, recent data studying foam cells in in vivo model systems^92^ or isolating foam cells from murine plaques^5^ clearly demonstrate that foam cells do not necessarily show proinflammatory characteristics and even may be considered anti-inflammatory.^92,93^ In line, our data show that cells exhibiting the foam cell–driven LXR activation program do not express high levels of *IL1B* and *TNF*. This further confirms that lipid accumulation leads to *LXR* activation and induces an anti-inflammatory phenotype. We also observed *TREM2* and *CD9* expression within this cluster, resembling the TREM2^+^ macrophages found in murine atherosclerosis.^4,6^ In other tissues, these TREM2^+^CD9^+^ macrophages have been described as either lipid-associated macrophages^45^ in obesity, or as scar associated macrophages^44^ in liver cirrhosis. Key phenotypes of these cells were shown to involve profibrotic characteristics and this is also of high relevance for human atherosclerosis as it may indicate a plaque stabilizing macrophage population.

Our study provides further supports the notion that trans-differentiation of cells is likely to occur in human atherosclerosis. About a quarter of the My.2 macrophages expressed smooth muscle cell actin, which may indicate derivation from SMCs, or conversely macrophages showing an SMC-like fibrotic phenotype.^17,94^ Presence of myeloid lineage-specific TF expression in these cells (eg, SPI and CEBPB) and absence of SMC TFs (eg, MYOCD and MRTFA) suggests that the latter is more likely. This is in line with previous reports applying SMC lineage tracing that showed that unidentified SMC-derived cells in atherosclerotic lesions exhibit phenotypes of other cell lineages, including macrophages and mesenchymal stem cells.^17,95^ Also EC cluster E.3 was characterized by expression of smooth muscle cell markers, such as *ACTA2*, *MYH11*, and *NOTCH3*, suggesting that these cells could be in endothelial to mesenchymal transition. Mature ECs can exhibit considerable heterogeneity and can transdifferentiate into mesenchymal-like cells, a biological process called endothelial to mesenchymal transition.^95^ There is accumulating evidence that endothelial to mesenchymal transition plays a role in atherosclerotic lesion progression and which has been linked with inflammatory stress and endothelial dysfunction.^96,97^ Our study shows that distinct EC clusters are present within atherosclerotic lesions and the gene signatures identify a cluster that shares both SMC and EC characteristics further providing human supportive evidence that endothelial to mesenchymal transition may occur in advanced human atherosclerotic plaques.

Apart from cellular plasticity within the endothelial and macrophage population, our study also provides new insights regarding intercellular communication within the plaque and its role in progression of atherosclerosis. We have shown that the within the plaque this was predicted to be most prevalent between myeloid, endothelial, and smooth muscle cells. In addition to previous studies predicting interactions between macrophages and T cells in human lesions,^7^ we were also able to predict interactions between ECs and SMCs, which were mainly involved with chemotaxis and extravasation of myeloid cells. We also show activation and recruitment of other immune cells, in particular T cells. Future development of therapeutics may benefit from detailing these interactions, providing specific pathways to target.

One of the significant post-GWAS challenges is the identification of candidate genes and pathways with clinical potential.^98^ Here, we mapped genes based on common variants (minor allele frequency >1%) in susceptibility loci and used single-cell resolution expression in disease-relevant tissue to identify putative targets for future functional follow-up. Our analysis showed enriched expression of CAD-associated genes in myeloid, endothelial, and smooth muscle cells. Furthermore, some of these genes are involved in cell-cell interactions, such as *SORT1* and *CXCL12*. Interestingly, the candidate genes did not show a significant overlap with T-cell–specific transcriptional signatures. Our approach is pragmatic in that we explicitly focus on (1) common variants in risk loci associated with CAD, (2) map protein-coding genes that are associated with CAD to these risk loci, and (3) select CAD-associated genes that are also differentially expressed between cell populations. This identifies tangible potential targets as starting points for future functional testing in macrophages, endothelial, and smooth muscle cells. However, we note that rare loss-of-function variants and underrepresented genes may have significant effects on these and other cells. Future studies focusing on loss-of-function variants and under-expressed genes could identify potentially other cell-specific targets.

There are several limitations that come with the use of human plaque endarterectomy samples. The vast majority of carotid endarterectomy samples also contain an inevitable small medial smooth muscle cell layer that potentially has contributed to the contractile smooth muscle cell cluster. There is a fine line between increasing digestion time to isolate more cells and generating a pure sample containing a high number of viable cells. We, therefore, did not exclude that the ratio of cell types that we detected in the plaques based on gene expression profiles was affected by the digestion procedure.

In summary, we provide an in-depth characterization of the highly diverse cellular communities in advanced human atherosclerotic plaques. Based on RNA expression and chromatin accessibility profiles of individual cells, we uncover among others the presence of proinflammatory, cytotoxic T-cell populations, multiple activation states of macrophages and their interactions, and functionally distinct EC populations that all can be considered modulators of human disease development. Furthermore, we show that by incorporating GWAS data, scRNA-seq data can be applied to map CVD susceptibility loci to specific cell populations and define potential patient-driven relevant targets for drug intervention of specific cell types. Our approach thus provides a powerful tool to aid research into the mechanisms underlying human disease and discover novel drug targets for intervention.

## Acknowledgments

Corresponding authors: Johan Kuiper Leiden Academic Centre for Drug Research, Division of Biotherapeutics, Leiden University, Einsteinweg 55, 2333 CC Leiden, The Netherlands. Email: j.kuiper@lacdr.leidenuniv.nl; or Menno P.J. de Winther: Amsterdam University Medical Centers–location AMC, University of Amsterdam, Experimental Vascular Biology, Department of Medical Biochemistry, Amsterdam Cardiovascular Sciences, Amsterdam Infection and Immunity, Meibergdreef 9, Amsterdam, The Netherlands. Email: m.dewinther@amsterdamumc.nl; or Michal Mokry: Laboratory of Clinical Chemistry and Haematology, University Medical Center, Heidelberglaan 100, Utrecht, The Netherlands Email: m.mokry@umcutrecht.nl; or Gerard Pasterkamp: Laboratory of Clinical Chemistry and Haematology, University Medical Center, Heidelberglaan 100, Utrecht, The Netherlands. Email: g.pasterkamp@umcutrecht.nl.

We thank Judith Vivié and Dr Mauro Muraro of Single Cell Discoveries for processing the plates for sequencing. We thank Dr Anouk Wezel and Dr Harm Smeets for sample collection at the Haaglanden Medisch Centrum. We acknowledge Biocenter Finland for infrastructure support. We are grateful to Kimmo Mäkinen for enabling sample acquisition at Kuopio University Hospital. M.A.C. Depuydt, K.H.M. Prange, and L. Slenders drafted the article and designed the figures. G.J. de Borst performed carotid endarterectomy procedures. M.A.C. Depuydt, D. Elbersen, I. Bot, B. Slütter, S.C.A. de Jager, and M. Mokry provided the pilot experiments. D. Elbersen and M.A.C. Depuydt executed the human plaque processing, fluorescence-activated cell sorting (FACS) and flow cytometry. K.H.M. Prange performed the clustering analyses. L. Slenders performed the genome-wide association study (GWAS) analysis with help from M. Mokry, S.W. van der Laan, A. Boltjes, and F.W. Asselbergs, and S.W. van der Laan executed the FUMA data collection. M.A.C. Depuydt, K.H.M. Prange, L. Slenders, A. Boltjes, I. Bot, B. Slütter, and S.W. van der Laan participated in conceptualization, data interpretation, and provided critical feedback on the article. S.C.A. de Jager and E. Lutgens provided critical feedback on the article. H.M. den Ruijter and C.K. Glass provided funding and critical feedback on the article. T. Örd, E. Aavik, and T. Lönnberg processed the human plaque samples and carried out the experimental work for single-cell ATAC sequencing (scATAC-seq), analyzed the data, and prepared the corresponding figures. M.U. Kaikkonen and S. Yla-Herttuala participated in the conceptualization, funding, and supervision of the scATAC-seq experiments and analysis. M. Mokry, J. Kuiper, M.P.J de Winther, and G. Pasterkamp participated in the conceptualization, funding, and supervision of the single-cell RNA sequencing (scRNA-seq) experiments and analysis and finalization of the article. All authors provided feedback on the research, analyses, and article.

## Sources of Funding

This work was supported by The Dutch Heart Foundation (CVON2017-20: Generating the best evidence-based pharmaceutical targets and drugs for atherosclerosis [GENIUS II] to J. Kuiper, M.P.J de Winther, G. Pasterkamp, S.W. van der Laan, M.A.C. Depuydt, I. Bot, B. Slütter); Spark-Holding BV (grant number 2015B002 to M.P.J de Winther); NWO-ZonMW (Dutch Research Council - The Netherlands Organization for Health Research and Development; Programma Translationeel Onderzoek [PTO] program Inhibition of mast cell activation in atherosclerotic lesions using an anti-IgE antibody approach (grant number 95105013 to M.A.C. Depuydt, I. Bot, and J. Kuiper); the European Union (Innovative Training Networks [ITN]-grant EPIMAC to M.P.J de Winther); Fondation Leducq (Transatlantic Network Grants to M.P.J de Winther, C.K. Glass, S. Yla-Herttuala, and G. Pasterkamp); EU 755320 Taxinomisis grant (G.J. de Borst, A. Boltjes, G. Pasterkamp). We acknowledge the European Research Area Network on Cardiovascular Diseases (ERA-CVD, grant number 01KL1802 to S.W. van der Laan, G. Pasterkamp); the ERA-Endless consortium (Dutch Heart Foundation, grant number 2017/T099 to H.M. den Ruijter and G. Pasterkamp), European Research Council (ERC) consolidator grant (grant number 866478 UCARE to H.M. den Ruijter). M.U. Kaikkonen was supported by the ERC under the European Union’s Horizon 2020 research and innovation programme (grant number 802825 to M.U. Kaikkonen), the Academy of Finland (Decisions 287478 and 319324), the Finnish Foundation for Cardiovascular Research, and the Sigrid Jusélius Foundation. T. Örd and M.U. Kaikkonen were supported by the Health from Science Academy Programme of Academy of Finland (TERVA) Programme of the Academy of Finland (Decision 314554). T. Lönnberg was supported by the Academy of Finland (Decisions 311081 and 314557).

## Disclosures

None.

## Supplemental Materials

Expanded Material & Methods

Online Figures I–VIII

Online Table I–IV

References^99–112^

## Supplementary Material



## Novelty and Significance

### What Is Known?

- Atherosclerotic lesions show a complex cellular composition that has mainly been studied using selected marker molecules.
- The benefit of using single-cell RNA sequencing as unbiased method has been shown for immune cells in both murine and human atherosclerosis.
