# Supplementary figures and images for: Microanatomy of the Human Atherosclerotic Plaque by Single-Cell Transcriptomics
Source: Circ Res. 2020 Sep 28;127(11):1437–55. doi: 10.1161/CIRCRESAHA.120.316770 (PMC7641189; doi:10.1161/CIRCRESAHA.120.316770)

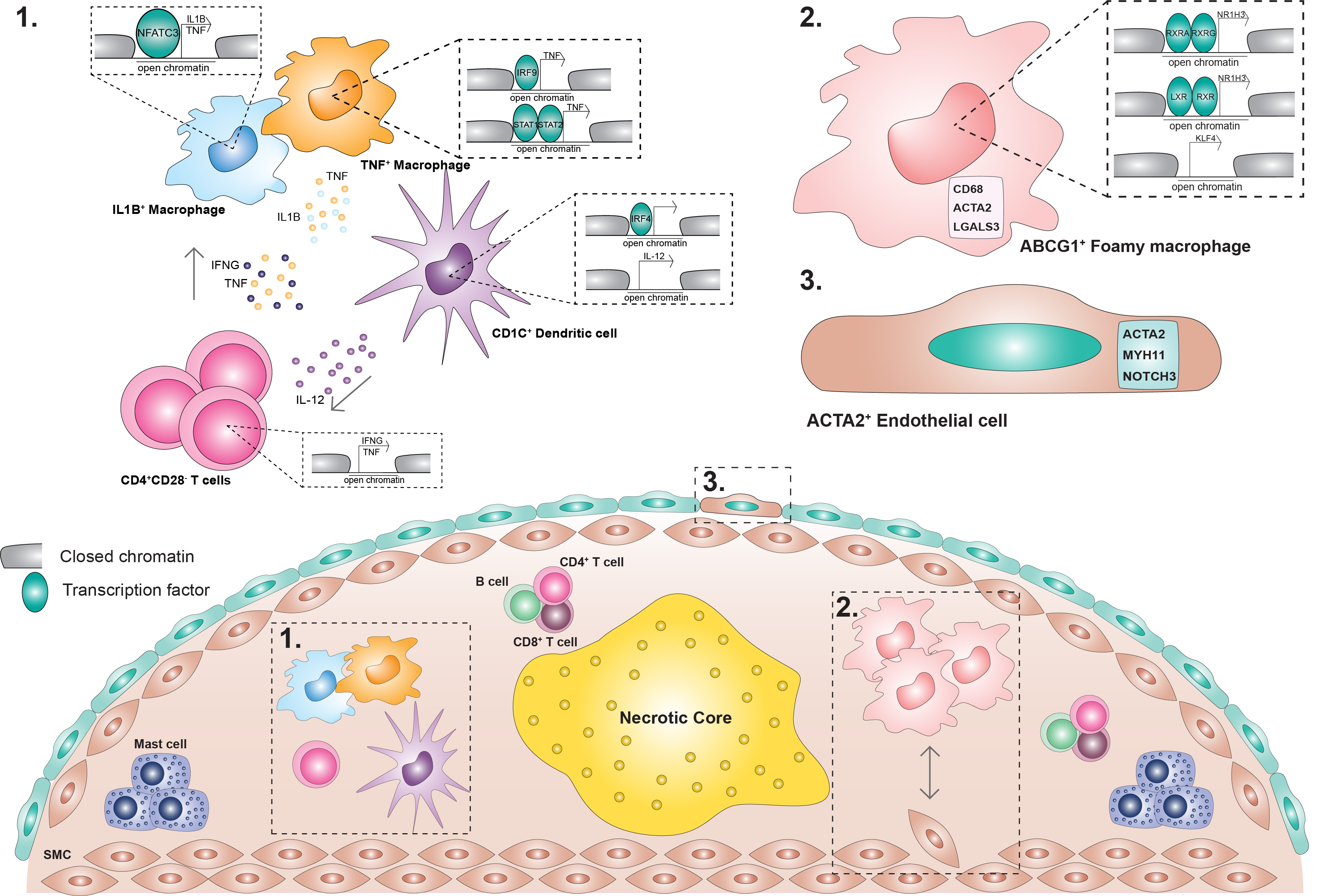

Supplement: Supplementary file 1 [file res-127-1437-s001.jpg]
