# Supplementary material for: Microanatomy of the Human Atherosclerotic Plaque by Single-Cell Transcriptomics
Source: Circ Res. 2020 Sep 28;127(11):1437–55. doi: 10.1161/CIRCRESAHA.120.316770 (PMC7641189; doi:10.1161/CIRCRESAHA.120.316770)
Supplement: Supplementary file 2 [file res-127-1437-s002.pdf]

Major Resources Table

In order to allow validation and replication of experiments, all essential research materials listed in the Methods should be included in the Major Resources Table below. Authors are encouraged to use public repositories for protocols, data, code, and other materials and provide persistent identifiers and/or links to repositories when available. Authors may add or delete rows as needed.

Antibodies

Target antigen	Vendor or Source	Catalog #	Working concentration	Lot # (preferred but not required)	Persistent ID / URL
CD3	DAKO		1:100	N/A	www.agilent.com/dako-products
CD68	Novocastra	NCL-CD68-KP1	1:3200	N/A	www.shop.leicabiosystems.com/us/ihc-ish/ihc-primary-antibodies/
CD34	Ventana Medical Systems	790-2927	Manufacturers instructions	N/A	www.diagnostics.roche.com
α-SMA	Sigma	A2547	1:20000	N/A	www.sigmaaldrich.com
Calcein AM	Invitrogen	C3099	1:20	N/A	www.thermofisher.com/invitrogen
Hoechst 33342	Invitrogen	H3570	1:100		www.thermofisher.com/invitrogen
Fixable viability dye – eFluor 450	eBioscience	65-0865-18	1:1000	N/A	www.thermofisher.com/ebioscience
CD45 – PECy7	eBioscience	25-9459-42	1:20	N/A	www.thermofisher.com/ebioscience
CD3 - BV421	Biolegend	317343	1:20	N/A	www.biolegend.com
CD4 – PETR	Life technologies	MHC50417	1:20	N/A	www.thermofisher.com
CD28 - BV650	Biolegend	302946	1:20	N/A	www.biolegend.com
Granzyme B – PE	eBioscience	12-8899-41	1:20	N/A	www.thermofisher.com/ebioscience
TruStain FcX	Biolegend	422302	1:250	N/A	www.biolegend.com

Data & Code Availability

Description	Source / Repository	Persistent ID / URL
Sequencing data	Upon request	

Other

Description	Source / Repository	Persistent ID / URL
RPMI 1640	Gibco	www.thermofisher.com
Collagenase IV	ThermoFisher Scientific	www.thermofisher.com
DNAse I	Sigma	www.sigmaaldrich.com
Human Albumin Fraction V	MP Biomedicals	www.mpbio.com
Flavopiridol (Alvocidib)	Selleckchem	www.selleckchem.com
Phorbol 12-myristate 13-acetate	Sigma	www.sigmaaldrich.com
Ionomycin	Sigma	www.sigmaaldrich.com
Multi-Tissue Dissociation kit	Miltenyi Biotec	www.miltenyibiotec.com
Dead cell removal kit	Miltenyi Biotec	www.miltenyibiotec.com
Iodixanol	OptiPrep, Sigma	www.sigmaaldrich.com

DOI [to be added]

DOI [to be added]
