# Supplementary material for: Microanatomy of the Human Atherosclerotic Plaque by Single-Cell Transcriptomics
Source: Circ Res. 2020 Sep 28;127(11):1437–55. doi: 10.1161/CIRCRESAHA.120.316770 (PMC7641189; doi:10.1161/CIRCRESAHA.120.316770)
Supplement: Supplementary file 3 [file res-127-1437-s003.pdf]

SUPPLEMENTAL MATERIAL

MICROANATOMY OF THE HUMAN ATHEROSCLEROTIC PLAQUE BY SINGLE-CELL TRANSCRIPTOMICS

Marie AC Depuydt, MSc.[1#], Koen HM Prange, PhD[2#], Lotte Slenders, MSc.[3#], Tiit Örd, PhD[4], Danny Elbersen[5], Arjan Boltjes, PhD[3], Saskia CA de Jager, PhD[5], Folkert W Asselbergs, Prof. MD PhD[3], Gert J. de Borst, Prof. MD PhD[6], Einari Aavik, PhD[4], Tapio Lönnberg, PhD[7], Esther Lutgens, Prof. MD PhD[2,8], Christopher K Glass, Prof. MD PhD[9,10], Hester M. den Ruijter, PhD[11], Minna U Kaikkonen, PhD[4], Ilze Bot, PhD[1], Bram Slütter, PhD[1], Sander W van der Laan, PhD[3], Seppo Yla-Herttuala, Prof. MD PhD[4], Michal Mokry, MD PhD[3,11*], Johan Kuiper, Prof. PhD[1*], Menno PJ de Winther, Prof. PhD[2,8*], Gerard Pasterkamp, Prof. MD PhD[3*]

Affiliations

1 Leiden Academic Centre for Drug Research, Division of Biotherapeutics, Leiden University, Einsteinweg 55, 2333 CC Leiden, The Netherlands
2 Amsterdam University Medical Centers – location AMC, University of Amsterdam, Experimental Vascular Biology, Department of Medical Biochemistry, Amsterdam Cardiovascular Sciences, Amsterdam Infection and Immunity, Meibergdreef 9, Amsterdam, The Netherlands
3 Laboratory of Clinical Chemistry and Haematology, University Medical Center, Heidelberglaan 100, Utrecht, The Netherlands
4 A.I.Virtanen Institute for Molecular Sciences, University of Eastern Finland, 70210 Kuopio, Finland
5 Laboratory for Experimental Cardiology, University Medical Center Utrecht, Heidelberglaan 100, Utrecht, The Netherlands
6 Department of Vascular Surgery, University Medical Centre Utrecht, Heidelberglaan 100, Utrecht, The Netherlands
7 Turku Bioscience Centre, University of Turku and Åbo Akademi University, 20520 Turku, Finland
8 Institute for Cardiovascular Prevention (IPEK), Munich, Germany & German Center for Cardiovascular Research (DZHK), partner site Munich Heart Alliance, Munich, Germany
9 Cell and Molecular Medicine, University of California San Diego, San Diego, CA, USA.
10 School of Medicine, University of California San Diego, San Diego, CA, USA.
11 Department of Cardiology, University Medical Center Utrecht, Heidelberglaan 100, Utrecht, The Netherlands

CONTENT

Expanded Material & Methods
Online figures I-VIII
Online table I-IV
References 100-113

EXPANDED MATERIALS AND METHODS

Patient population

Atherosclerotic plaques were obtained from 14 male and 4 female patients undergoing a carotid endarterectomy (CEA) procedure. All plaque specimens were included in the Athero-Express Biobank Study (AE, www.atheroexpress.nl), an ongoing biobank study at the University Medical Centre Utrecht (UMCU)[100] (Cohort 1). Only primary CEAs were included, restenotic plaques were excluded due to their difference in composition compared to primary atherosclerotic plaques[101]. The study was approved by the Medical Ethical Committee of the UMCU. For flow cytometry, seven plaques (5 male, 2 female) were used that were obtained from patients undergoing CEA procedure at Haaglanden Medical Center Westeinde (The Hague, The Netherlands) (Cohort 2). The study was approved by the Medical Ethical Committee of the HMC. For scATAC-Seq, atherosclerotic plaque samples were obtained from 3 patients undergoing CEA procedure at Kuopio University Hospital, Kuopio, Finland (Cohort 3). The studies were approved by Local Ethical Committee of Kuopio University Hospital. All studies were performed in accordance with the declaration of Helsinki and all patients gave written informed consent before start of the study.

Human plaque processing (Single Cell)

Human carotid plaques of Cohort 1 were collected during CEA; the culprit segment (5 mm) was used for histology and embedded in paraffin as described elsewhere[100]. Time between surgical removal and plaque processing did not exceed 10 minutes. The inclusion of a small medial layer in the dissected tissue could not be excluded during the surgical procedure. The plaques were characterized as fibrous (n=6), fibro-atheromatous (n=6) or atheromatous (n=6). Characterization was in concordance with the Athero-Express Biobank Study protocols. The remainder of the plaque washed in RPMI and minced into small pieces with a razor blade. The tissue was then digested in RPMI 1640 containing 2.5 mg/mL Collagenase IV (ThermoFisher Scientific), 0.25 mg/mL DNAse I (Sigma), 2.5 mg/mL Human Albumin Fraction V (MP Biomedicals) and 1 mM Flavopiridol (Selleckchem) at 37°C for 30 minutes. Subsequently, the plaque cell suspension was filtered through a 70 µm cell strainer and washed with RPMI 1640. Cells were kept in RPMI 1640 with 1% Fetal Calf Serum until subsequent staining for fluorescence-activated cell sorting. Remaining, unstained cells were cryostored in liquid nitrogen.

Immunohistochemistry

Immunohistochemical staining of CD3, CD34, αSMA (ACTA2) and CD68 was performed fully automated (Benchmark, Ventana Medical Systems, Yuscon AZ). Stainings were performed on matched samples of patients from cohort 1 (n=3). The CD3 antibody was purchased from DAKO and used at a dilution of 1:100. The CD68 antibody was purchased from Novocastra (cat. No. NCL-CD68-KP1) and used at a dilution of 1:3200. The CD34 antibody was purchased from Ventana Medical Systems (cat. No. 790-2927) and used at the dilution recommended by manufacturer. The αSMA antibody was purchased from Sigma (cat. No. A2547) and used at a dilution of 1:20000.

Human plaque processing (whole tissue)

Whole plaque tissue RNA-seq was obtained from two male Athero-Express biobank samples. RNA-seq library preparation was performed using the CEL-Seq2 Sample Preparation Protocol[102] and sequenced as 2 x 75bp paired-end on a Illumina NextSeq 500 (Utrecht Sequencing Facility). The reads were de-multiplexed and aligned to human cDNA reference using the BWA (v0.7.13)[103]. Downstream analysis was performed using custom R scripts.

Fluorescent activated cell sorting of viable cells

Single cell suspensions were stained with Calcein AM and Hoechst (ThermoFisher Scientific) in PBS supplemented with 5% Fetal Bovine Serum (FBS) and 0.2% ethylenediaminetetraacetic acid (EDTA) for 30 minutes at 37ºC. After staining, cells were washed and filtered through a 70 µm FlowMi cell strainer (SP Scienceware). Viable cells, positive for both Calcein AM and Hoechst, were sorted using the Beckman Coulter MoFlo Astrios EQ.

Flow cytometry

Flow cytometry was performed on defrosted single cell suspensions of plaques from three patients of cohort 1 and on seven plaques single cell suspensions from cohort 2 (characterized as advanced plaques that showed luminal thrombosis, regions of intraplaque hemorrhage, large macrophage regions and a large necrotic core). All samples were digested according to the same protocol as described above for the scRNA-seq samples. The single plaque cells were stimulated with phorbol 12-myristate 13-acetate (PMA, 50 ng/mL, Sigma-Aldrich), ionomycin (500 ng/mL, Sigma-Aldrich) and Brefeldin A (ThermoFisher Scientific) for 3.5h in complete RPMI at 37°C and 5% CO_2. Subsequently, cells were stained with extracellular antibodies, fixated, permeabilized and stained with intracellular antibodies (Online Table 4). The fluorescently labeled plaque cells were measured using a Cytoflex S (Beckman and Coulter) and analysed with FlowJo software. Statistical analysis was performed using Graphpad Prism 8 software. Data passed the Shapiro-Wilk normality test and an unpaired T-test was performed to determine significance.

Single cell RNA-sequencing

Using a Mosquito® HTS (TTP Labtech) 384 wells plates were filled with 50nL lysis buffer containing CELseq2-primers, spike-ins and dinucleotide triphosphates (dNTPs) and overlaid with mineral oil to prevent evaporation. Viable cells were sorted one cell per well into these 384 wells plates, fixed by lysis to preserve expression status, and immediately frozen at -80ºC until further processing. First, cDNA was constructed using the SORT-seq protocol[104]. In short, cells were lysed for 5 min at 65ºC and subsequently reverse transcription and second strand mixes were added using the Nanodrop II liquid handling platform (GC biotech). Next, cells were pooled in one library and the aqueous phase was separated from the oil phase, followed by in vitro transcription (IVT). A library was formed using the CEL-Seq2 protocol[102]. Primers consisted of a 24 bp polyT stretch, a 64bp random molecular barcode (UMI), a cell-specific 8bp barcode, the 5′ Illumina TruSeq small RNA kit adaptor and a T7 promoter. For sequencing, TruSeq small RNA primers (Illumina) were added to the libraries and sequenced paired end at 75 bp read length using Illumina NextSeq 500.

scRNA-seq data processing and clustering

Single-cell sequencing data were processed as described previously[104]. Analyses were performed in an R 3.5 environment[105] using Seurat (version 2.3.4 and 3.0)[106]. Prior to processing, reads were filtered for mitochondrial and ribosomal genes, MALAT1, KCNQ1OT1, UGDH-AS1, and EEF1A. In order to omit doublets and low-quality cells, only cells expressing between 500 and 10.000 genes and genes expressed in at least 3 cells were used for further analysis. The following steps were performed using Seurat (version 2.3.4): data was log-normalized and scaled with the exclusion of unique molecular identifiers (UMIs). Top variable genes for all samples were used to combine samples into one object using Seurat function RunMultiCCA(), after which samples were aligned using AlignSubspace() with reduction.type=CCA and grouping.var=”plate”. Subsequently, canonical correlation analysis (CCA) reduction was performed with a resolution of 1.2 for 15 dimensions to identify clusters and to perform t-distributed stochastic neighbor embedding (tSNE). Cell types were assigned to cell clusters by evaluating gene expression of individual cell clusters using differential gene expression (Wilcoxon rank sum test) and analysis with SingleR[8] against BLUEPRINT[107] reference data. Sub-clustering of identified cell clusters was performed using CCA with a resolution of 0.9 or 1.5 for 15 dimensions (Seurat version

2.3.4). Downstream analysis of initial and sub-clusters was performed in a similar manner. Pathway analysis were performed using the EGSEA (version 1.16.0) with Canonical pathway and hallmark gene set collections from broad institute GSEA (version 1.17.0) and FDR for multiple testing correction. tSNE plots, dot plots and violin plots were made with Seurat (version 2.3.4 and 3.0). Figure 1E and S2C were made using SingleR (version 1.2.4). Barplots were made using ggplot2. For full details, see **Data access.**

Comparison whole- tissue and single-cell RNA-seq

Ilumina cell sequencing data was processed as described previously[104]. In summary, prior to processing reads were filtered for mitochondrial and ribosomal genes, MALAT1, KCNQ1OT1, UGDH-AS1 and EEF1A, and duplicate or unannotated entries. A "pseudobulk" patient was created by combining reads from all patients. Individual patients and the pseudobulk patient were compared to bulk RNA-seq as described above. Correlation coefficient was determined through Pearson correlation (CI 0.95). Scatterplot was made using ggplot2. All processing was performed using custom R scripts. For full details, see **Data access**.

Integration of mouse datasets

Top 20 marker genes from all myeloid cell clusters in the 4 mouse datasets[3–6] were converted to human symbols using the ENSEMBL biomaRt R package (v2.42.0) in an R v3.5.3 environment. Subsequently, the overlap of genes in the mouse clusters with significant ($p.adj < 0.1$) marker genes from My.0, My.1, and My.2 was determined and significance calculated by hypergeometric test using standard R function phyper() with parameters q-1 and lower.tail = F. Finally, bar graphs were generated with ggplot2 and circos plots of overlaps were generated on http://metascape.org by uploading lists of marker genes per cluster, starting a custom analysis, and downloading the corresponding 'gene circos.svg' file from the 'ID conversion' tab. For full details, see **Data access**.

Ligand-receptor interaction analysis

Ligand-receptor interactions were calculated using cellphonedb [v2.11 – database version 2.0.0][50] with default settings. Heatmap was made with cellphonedb. Top significant ($p < 0.05$) interactions were defined as > 3rd quartile and plotted using ggplot2. For full details, see **Data access**.

Human plaque processing for scATAC-Seq

The tissue samples from cohort 3 (characterized as fibro-atheromatous) were minced with a scalpel and enzymatically dissociated using Miltenyi Biotec Multi-Tissue Dissociation Kit supplemented with 0.5% BSA and 20 mM HEPES buffer (pH 7.2-7.5) for 60 min at 37 °C with end-over-end rotation. The suspension was passed through a 30 µm strainer and viable cells were purified magnetically using the Dead Cell Removal Kit (Miltenyi Biotec). For the isolation of nuclei from cells, the lysis buffer formulation recommended by 10x Genomics for scATAC-Seq nuclei preparation was used. The nuclei were purified by iodixanol (OptiPrep; Sigma) density gradient centrifugation at 3300g for 20 min at 4 °C. The nuclei were recovered at the interface of 29% and 35% iodixanol. The nuclei were resuspended in ice-cold 10x Genomics Nuclei Buffer and counted using fluorescence staining (DAPI). Approximately 5000-6000 nuclei per sample were processed on the 10x Genomics Chromium Controller instrument, followed by scATAC-Seq library preparation according to the manufacturer's protocol. The libraries were sequenced on an Illumina NextSeq sequencer using the standard protocol recommended by 10x Genomics for scATAC-Seq libraries.

scATAC-Seq data analysis

Sequencing data preprocessing, library quality control, cell calling and peak calling were done with the Cell Ranger ATAC pipeline (10x Genomics, version 1.1). Integration of scATAC-Seq samples, clustering of cells, and visualization of chromatin accessibility and pseudobulk coverage tracks was performed with Seurat (version 3.0)[108] and its extension Signac (version 0.15; https://github.com/timoast/signac). scATAC-Seq cells were matched to scRNA-Seq cell types using the scRNA-Seq as the reference data set in a cross-modal label transfer procedure, as described in Stuart et al.(2019).[108] Differential motif accessibility was calculated using chromVAR (version 1.5)[109] using the human motifs from the JASPAR CORE motif collection (2018 release)[110]. First, at the level of individual cells, the relative accessibility of each motif genome-wide ('motif activity') was computed as the deviation in motif occurrence in the accessible peaks compared to a set of background peaks matched for GC content, using the default parameters of the chromVAR method.[109] Subsequently, cell clusters were tested for differences (both positive and negative direction) in motif activity using a logistic regression test with the total peak count as a latent variable, as suggested for differential motif analysis by the authors of the Signac package109. $P < 0.05$ after correction for multiple testing using the Benjamini–Hochberg procedure was considered significant. Heatmaps were made using pheatmap (version 1.0.12), genome plots were made using Signac. For full details, see **Data access**.

Differential gene expression analysis and mapping of GWAS loci

For the analysis and mapping of CAD associated gene to the single-cell RNAseq data, we applied three steps: 1) we prioritized genes based on the CAD GWAS summary statistics, 2) we determined the genes differentially expressed between cell type and therefore specific to these cell types, and 3) we overlapped the GWAS-derived genes and differentially expressed genes to identify genes associated to CAD and specific to a cell type.

Step 1: We used the summary statistics from the GWAS on CAD by Nelson *et al.* which included CARDIoGRAMplusC4D and UK Biobank data[71]. This data was annotated with a the Functional Mapping and Annotation of Genome-Wide Association Studies (FUMA v1.3.3b, https://fuma.ctglab.nl) platform.[111] FUMA can be used to annotate, prioritize, visualize and interpret GWAS results. In short, FUMA takes GWAS summary statistics as an input, it will map and match the results to a reference (in this case the 1000G EUR population, https://www.internationalgenome.org[112]), and provides extensive functional annotation for all SNPs (present in the summary statistics and the reference used) in genomic areas identified by lead SNPs. Various filters can be applied. Here, we applied genome-wide significance ($p = 5x10^{-8}$), a linkage disequilibrium $r^2 = 0.05$ for clumping of independent loci within 1000 kb of the lead variant (PLINK version 1.9), and included only variants with minor allele frequency (MAF) > 1%. Annotation and mapping of genes were based on 1) position (within 1000kb of the lead variant), 2) limited to blood and vascular bed derived data from the Genotype-Tissue Expression (GTEx version 7) Project only. This resulted in a list of 644 genes that mapped to CAD loci. To further prioritize these 644 genes, we calculated per-gene p-values based on the per-SNP p-values from the CAD GWAS summary statistics. To this end FUMA uses MAGMA (https://ctg.cncr.nl/software/magma, v1.06[113]). MAGMA first analyses the individual SNPs in a gene and combines the resulting SNP p-values into a gene test-statistic while taking into account the underlying LD structure. Using permutations based on a randomly generated test-statistic drawn from the standard normal distribution, a per-gene empirical (permuted) p-value is derived. This results in a per-gene p-value of association with CAD for all genes in a reference (here ENSEMBL v92 was used and we only included protein-coding genes). We further prioritized the 644 genes mapped to CAD loci by selecting genes with a MAGMA p-value < 0.05. This selection resulted in 317 CAD associated genes (mapped to CAD associated loci) which were used for the downstream single-cell analysis and mapping.

Step 2: For the single-cell analysis and mapping, we have focused only on genes that 1) are specific for the different cell types in plaque, and that 2) have a genetic association with CAD. The purpose of the analysis is to find those genes in each cell type to provide tangible starting points for functional testing. Genes that are highly specific (i.e., highly expressed) are more likely to have a specific function within the cells, making them interesting targets when these genes are also genetically correlated with the

disease. To this end we performed a differential gene expression analysis, resulting in 3876 differentially expressed genes (DEG) listed in figure 7A, the second pillar of this analysis. For better interpretation and visualization, we sorted these genes into 15 gene expression patterns, which highlight the expression of genes in specific (related) cell types. Differential expression was tested between clusters by Wilcoxon sum rank testing in both a “One cluster vs. One cluster” and “One cluster vs. Remaining clusters” fashion. Genes were deemed differentially expressed if they met 2 criteria: 1) >= 10% of cells within one cell cluster express the gene with log2 fold change of >= 0.6, and 2) the gene passes the Bonferroni adjusted significance threshold of p.adjusted < 0.05 for this test. The 3876 differentially expressed genes (DEG) were sorted into 15 gene expression patterns by K-means clustering based on average expression per cell type (Online Table 3).

Step 3: After this, the 317 CAD associated genes derived from the GWAS summary statistics and the 3876 DEGs derived from the scRNAseq data are overlapped. This resulted in 74 genes that are both highly expressed in scRNAseq data (a DEG), and are associated to CAD based on GWAS summary statistics. We calculated enrichment of the 74 DEGs in the 15 gene expression patterns using permutation analysis. We sampled 75000 random sets of 317 genes from 26180 genes that were possible to map. We then calculated positive enrichment for each gene expression pattern for the mapped GWAS genes compared to the random sets and determined the enrichment of DEGs. Downstream processing was performed using custom R scripts. Heatmap was made with Seurat (version 2.3.4) and bar graph with ggplot2. For full details, see **Data access**.

Data access

In silico data analysis was performed using custom R scripts (R version 3.5.3) designed especially for this research and/or based on the recommended pipelines from the pre-existing packages listed in the individual segments above. R scripts are available on GitHub [https://github.com/AtheroExpress/MicroanatomyHumanPlaque_scRNAseq] Other data is available upon personal request to the corresponding authors (m.mokry@umcutrecht.nl; j.kuiper@lacdr.leidenuniv.nl; m.dewinther@amsterdamumc.nl; g.pasterkamp@umcutrecht.nl).

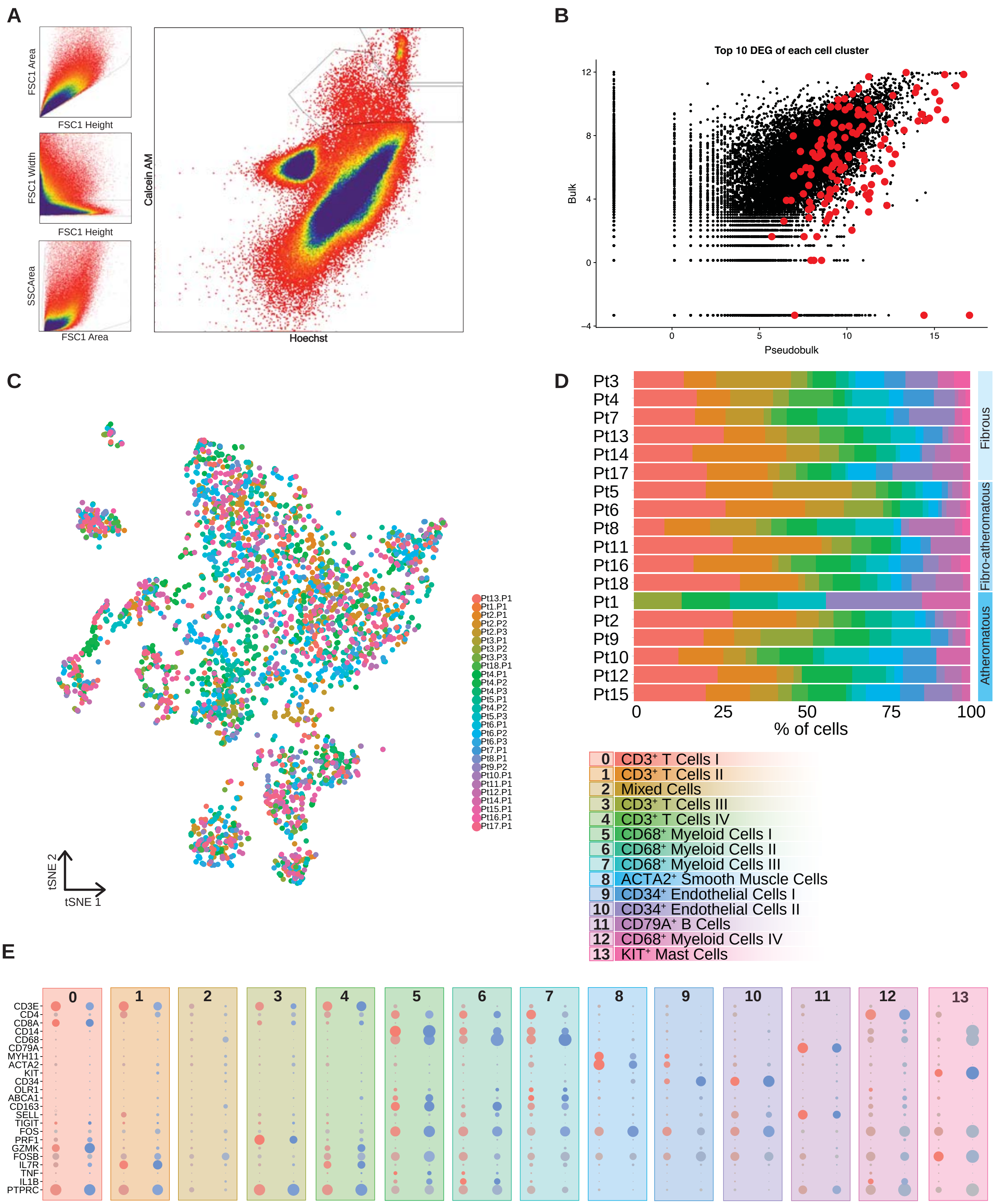

Online Figure I. CCA clustering and tSNE visualization revealed 14 distinct populations. A) FACS plots showing gating directions for viable cells, positive for both Calcein AM and Hoechst B) Single cell CEL-seq2 libraries showed good correlation with plaque bulk RNA-seq libraries. Correlation 0.643 p < 2.2e-10. C) Clusters were not biased towards individual PCR plates or patients. D) Relative cluster sizes were comparable between patients. E) Dotplot of marker genes confirmed population identities and low inter-sex variance.

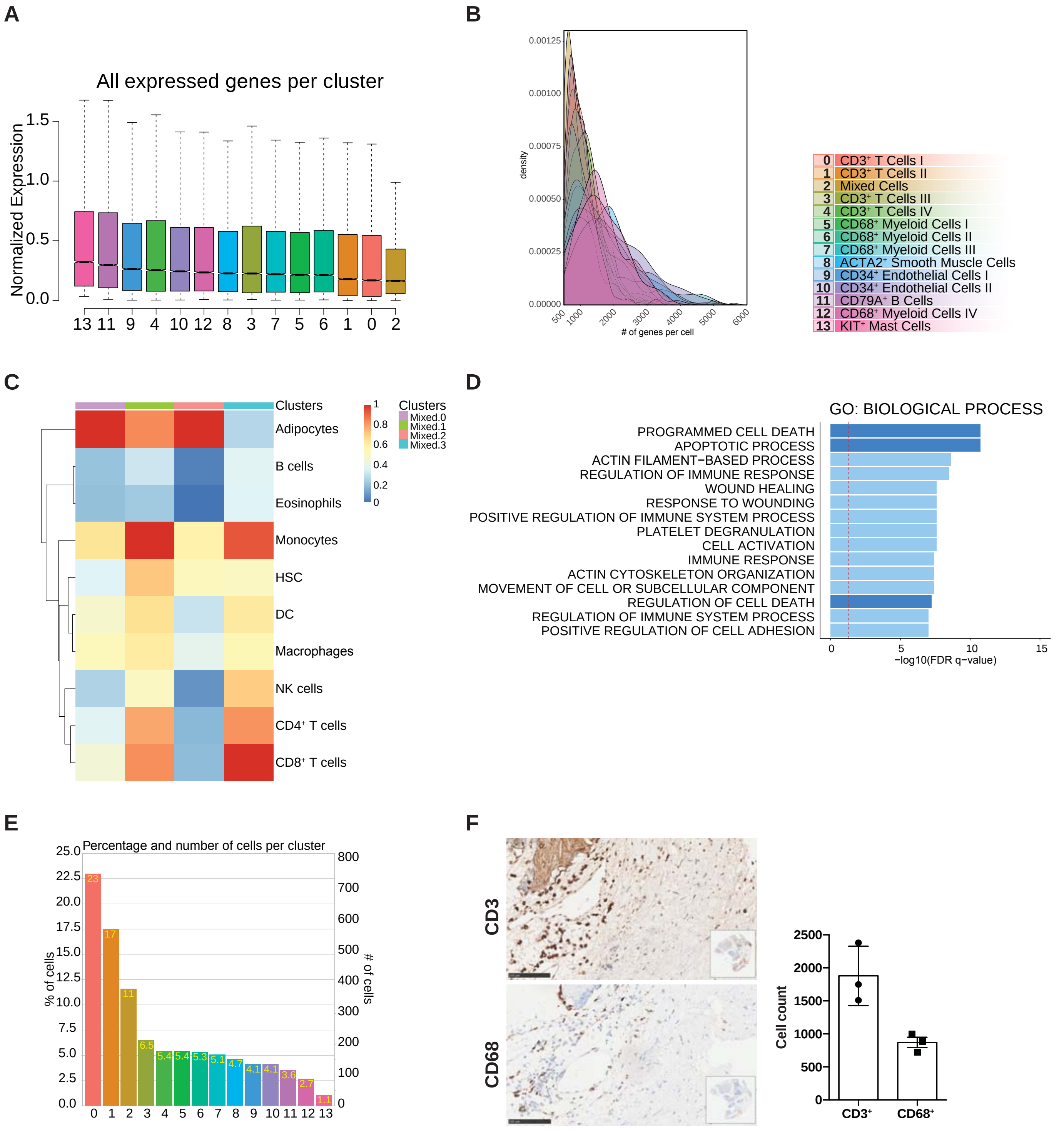

Online Figure II. Refinement of population identities. A) Boxplots of normalized gene expression per cluster. B) Per cluster density plots of number of detected genes per cell. C) Heatmap of subclusters of unknown cluster 2's similarity to reference datasets of known cell types. D) Top pathways associated with cluster 2. E) Distribution of number of cells per cluster. F) Immunostaining of 3 representative endarterectomy samples. Top left: $CD3^+$ T cells. Bottom Left: $CD68^+$ macrophages. Right: Barplots of positive cell counts, data shown as mean±SD (n=3). Scale bars represent 100µm.

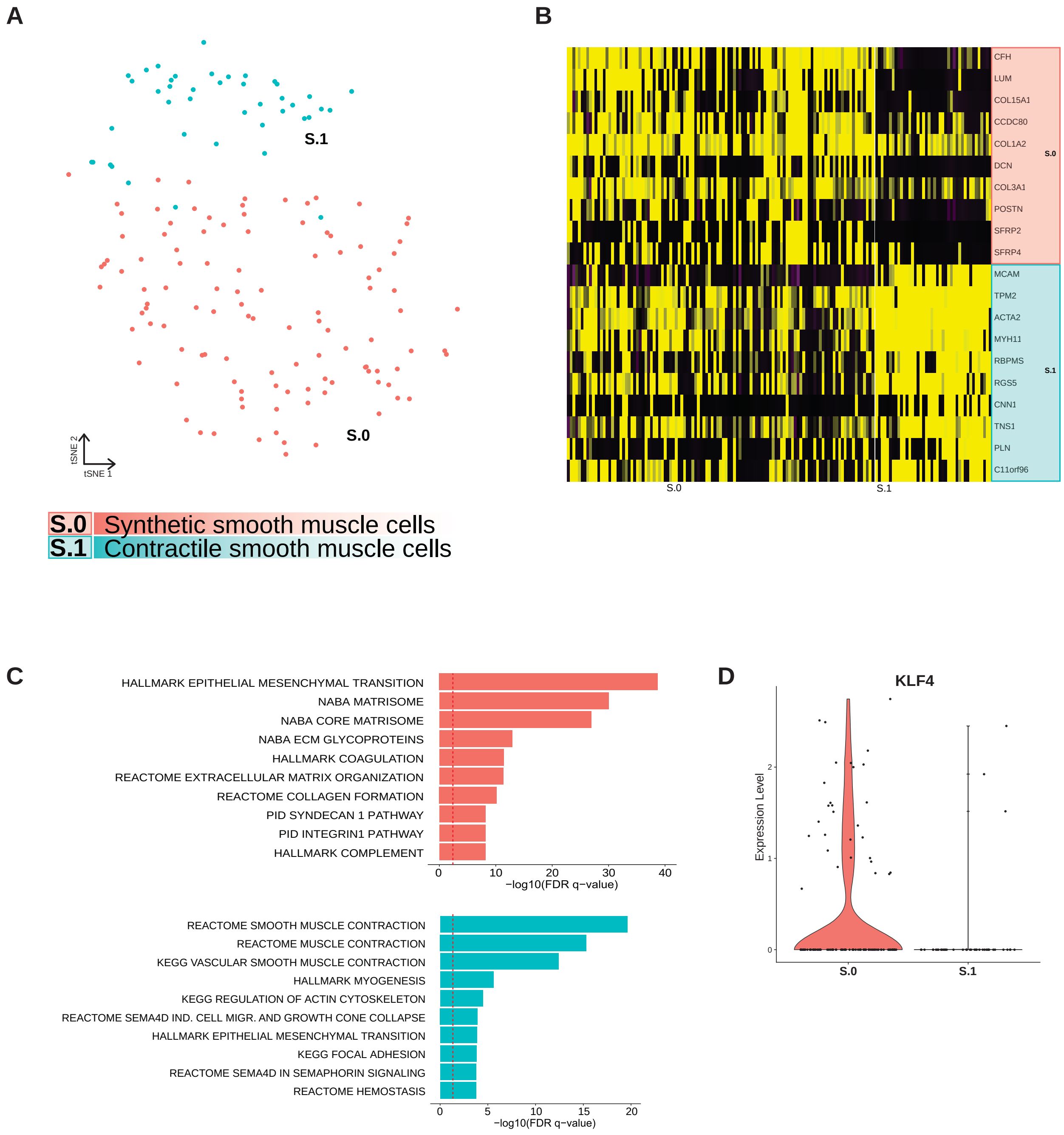

Online Figure III. Subclustering of smooth muscle cells revealed 2 distinct populations. A) tSNE visualization of clustering revealed 2 distinct smooth muscle cell populations. B) Heatmap of top marker genes per cluster. C) Top pathways associated with each population.

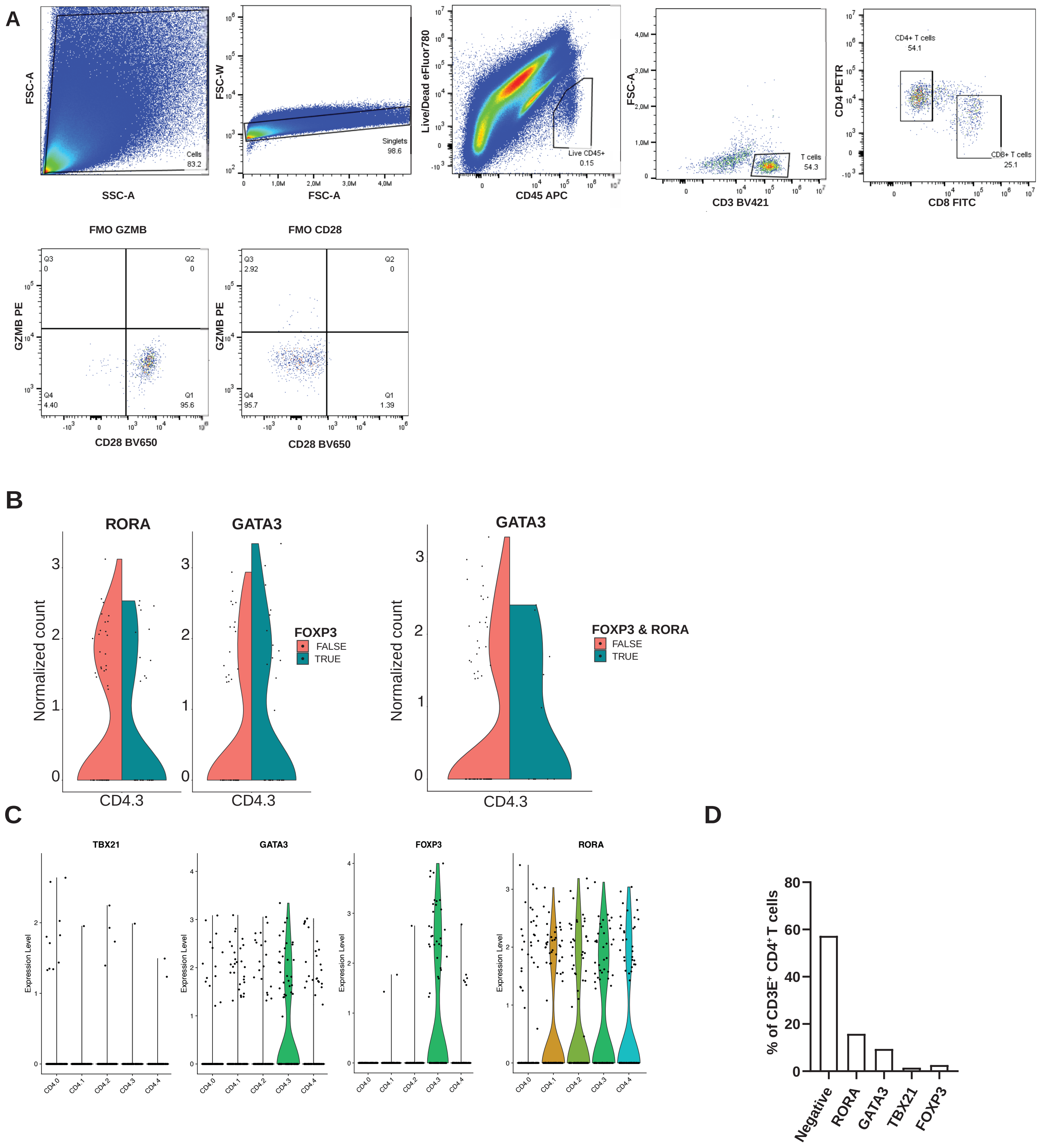

Online Figure IV. Representation of potential T_H-subsets in the human plaque. A) Gating strategy for *GZMB* staining within $CD4^+CD28^{null}$ T cells. B) Split-violins showing a correlation between expression of *FOXP3* and respectively *RORA* and *GATA3*; and cells that express all three transcription factors. C) Violin plots showing expression of helper T cell subset-related transcription factors within the $CD4^+$clusters. D) Independent analysis of all $CD3E^+CD4^+$ T cells. Negative $CD4^+$ T cells are negative for all transcription factors. Other bars represent the percentage of $CD4^+$ T cells that specifically express the noted transcription factor. *RORC* was not detected in our dataset.

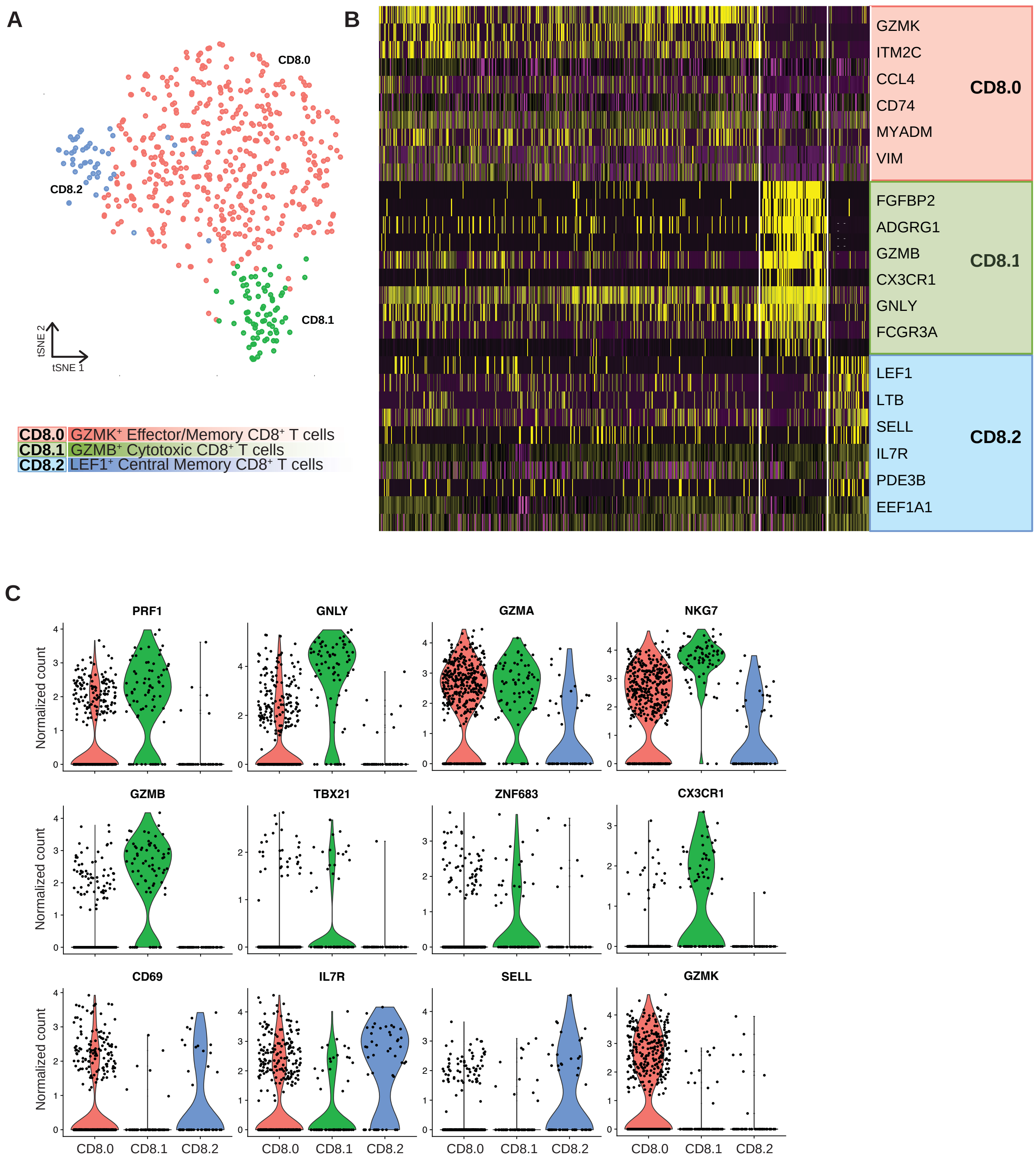

Online Figure V. Subclustering of CD8⁺ T cells revealed 3 distinct populations. A) tSNE visualization of clustering revealed 4 distinct CD8⁺ T cell populations. B) Heatmap of top marker genes per cluster. C) Violin plots of marker genes associated with CD8⁺ T cell cytotoxicity and quiescence.

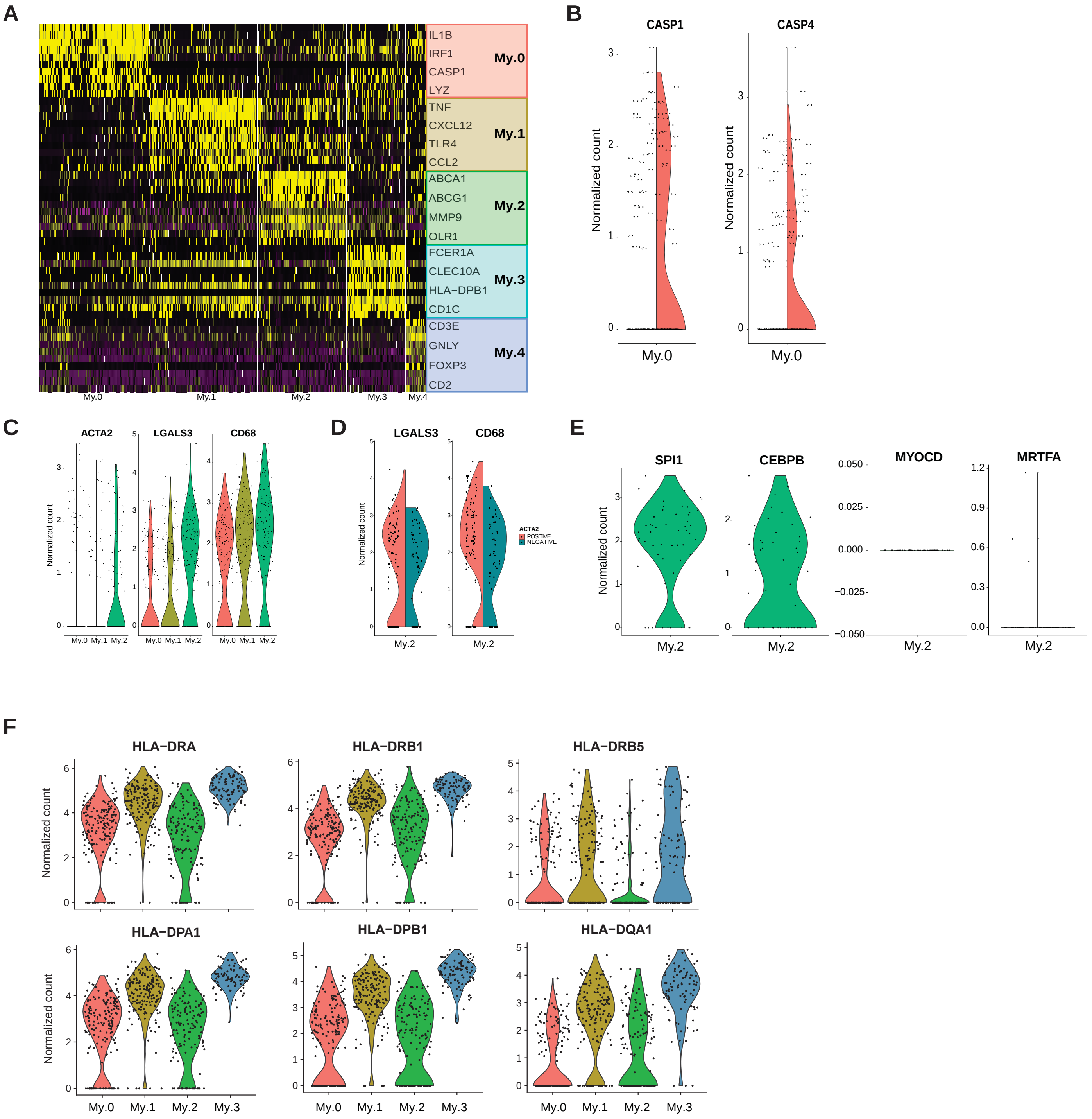

Online Figure VI. Myeloid population marker genes and SMC related gene expression. A) Heatmap of top marker genes per cluster. B) Split violin plot showing *CASP1* and *CASP4* expression in *IL1B*⁻ (left side of violins) and *IL1B*⁺ cells (right side of violins). C) Violin plot showing genes associated with the transition of smooth muscle cell to macrophage. D) Correlation of *ACTA2* expression with *CD68* and *LGALS3* levels in cluster My.2. E) Violin plots of *ACTA2*⁺ cells in cluster My.2 showing expression of myeloid lineage transcription factors *SPI1* and *CEBPB* and smooth muscle cell lineage transcription factors *MYOCD* and *MRTFA*. F) Violin plots of class II HLA subtypes expressed by cluster My.0 – My.4.

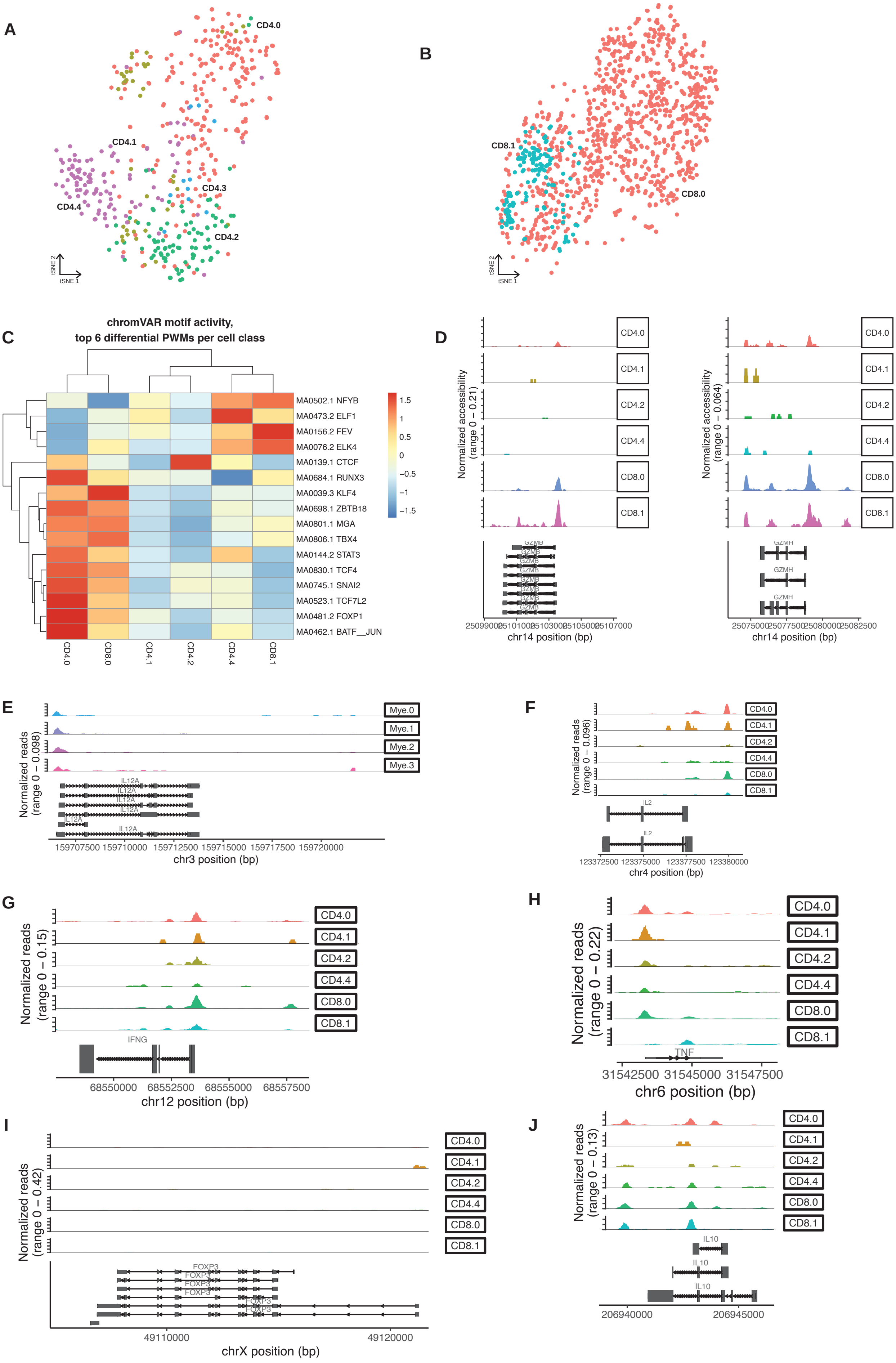
A
CD4.0
CD4.1
CD4.3
CD4.4
CD4.2
tSNE 2
tSNE 1
B
CD8.1
CD8.0
tSNE 2
tSNE 1
C
chromVAR motif activity,
top 6 differential PWMs per cell class
MA0502.1 NFYB
MA0473.2 ELF1
MA0156.2 FEV
MA0076.2 ELK4
MA0139.1 CTCF
MA0684.1 RUNX3
MA0039.3 KLF4
MA0698.1 ZBTB18
MA0801.1 MGA
MA0806.1 TBX4
MA0144.2 STAT3
MA0830.1 TCF4
MA0745.1 SNAI2
MA0523.1 TCF7L2
MA0481.2 FOXP1
MA0462.1 BATF__JUN
1.5
1
0.5
0
−0.5
−1
−1.5
CD4.0
CD8.0
CD4.1
CD4.2
CD4.4
CD8.1
D
Normalized accessibility
(range 0 − 0.21)
CD4.0
CD4.1
CD4.2
CD4.4
CD8.0
CD8.1
GZMB
chr14 position (bp)
Normalized accessibility
(range 0 − 0.064)
GZMH
25075000
25077500
25080000
25082500
chr14 position (bp)
E
Normalized reads
(range 0 − 0.098)
Mye.0
Mye.1
Mye.2
Mye.3
IL12A
159707500 159710000 159712500 159715000 159717500 159720000
chr3 position (bp)
F
Normalized reads
(range 0 − 0.096)
CD4.0
CD4.1
CD4.2
CD4.4
CD8.0
CD8.1
IL2
123372500 123375000 123377500 123380000
chr4 position (bp)
G
Normalized reads
(range 0 − 0.15)
CD4.0
CD4.1
CD4.2
CD4.4
CD8.0
CD8.1
IFNG
68550000 68552500 68555000 68557500
chr12 position (bp)
H
Normalized reads
(range 0 − 0.22)
CD4.0
CD4.1
CD4.2
CD4.4
CD8.0
CD8.1
TNF
31542500 31545000 31547500
chr6 position (bp)
I
Normalized reads
(range 0 − 0.42)
CD4.0
CD4.1
CD4.2
CD4.4
CD8.0
CD8.1
FOXP3
49110000
49120000
chrX position (bp)
J
Normalized reads
(range 0 − 0.13)
CD4.0
CD4.1
CD4.2
CD4.4
CD8.0
CD8.1
IL10
206940000
206945000

Online Figure VII. Chromatin accessibility of T cells in human atherosclerotic plaques analyzed using scATAC-seq. A) tSNE visualization of $CD4^+$ T cell subclusters. B) tSNE visualization of $CD8^+$ T cell subclusters. C) Heatmap showing the top differential TF motifs based on chromVAR. Pseudobulk genome browser visualization identifying the open chromatin regions of E) *IL12*, F) *IL2*, G) *IFNG*, H) *TNF*, I) *FOXP3* and J) *IL10*.

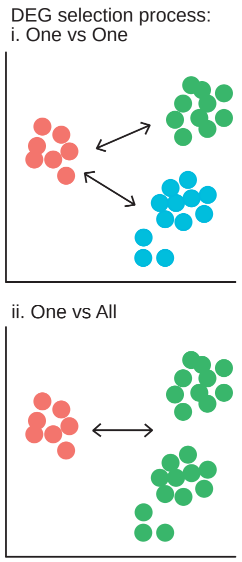

Online Figure VIII. Projection of CAD GWAS associated genes: DEG selection process. Schematic overview of differentially expressed gene (DEG) selection. Top: DEG were called comparing all clusters against one another. Bottom: DEG were called comparing one cluster against all others aggregated.

Online Table I

Baseline characteristics of patients of AtheroExpress (AE) cohort (cohort 1)

	**Cohort 1 (n = 18)**
Sex, male, N (%)	14 (77.9)
Age, y (SD)	71.6 (9.08)
**History, N (%)**	
Cerebrovascular disease	9 (50)
Coronary artery disease	7 (38.8)
Peripheral arterial occlusive disease	3 (16.7)
**Risk factors**	
BMI, kg/m2 (SD)	25.7 (3.38)
Hypercholesterolemia, N (%)	13 (72.2)
Hypertension, N (%)	13 (72.2)
Current smokers, N (%)	7 (38.8)
Type 2 diabetes mellitus	6 (33.3)
Total cholesterol, mmol/L (SD)	4.13 (0.86)
LDL, mmol/L (SD)	2.36 (0.74)
HDL, mmol/L (SD)	1.10 (0.18)
Triglyceride, mmol/L (SD)	1.75 (1.25)
GFR, MDRD, ml/min/1.73 cm2 (SD)	79.37 (30.5)
**Medication, N (%)**	
Statins	13 (72.2)
Lipid-lowering drugs	3 (16.7)
Anti-platelet drugs	13 (72.2)
**Pre-operative symptoms, N (%)**	
Asymptomatic	2 (11.1)
Ocular	5 (27.8)
TIA	1 (5.6)
Stroke	9 (50)
Major cardiovascular events during follow up	2 (11.1)
**Surgery, N (%)**	
De novo	18 (100)
Restenosis	-

Missing values	
Sex, N (%)	-
Age, N (%)	-
**History, N (%)**	
Cerebrovascular disease	-
Coronary artery disease	-
Peripheral arterial occlusive disease	3 (16.7)
**Risk factors, N(%)**	
BMI	2 (11.1)
Hypercholesterolemia	2 (11.1)

Hypertension	1 (5.6)
Current smokers	1 (5.6)
Type 2 diabetes mellitus	-
Total cholesterol	5 (27.8)
LDL	6 (33.3)
HDL	5 (27.8)
Triglyceride	6 (33.3)
GFR	2 (11.1)
**Medication, N (%)**	
Statins	1 (5.6)
Lipid-lowering drugs	1 (5.6)
Anti-platelet drugs	1 (5.6)
**Pre-operative symptoms, N (%)**	
Asymptomatic	-
Ocular	-
TIA	-
Stroke	-
Major cardiovascular events during follow up	14 (77.9)
**Surgery, N (%)**	
De novo	-
Restenosis	-

Online Table II

Cluster 0 marker genes

gene	p_val	avg_logFC	pct.1	pct.2	p_val_adj
CCL5	1,53E-199	1,443909202	0,886	0,392	2,91E-195
CD8A	6,88E-173	1,647518237	0,568	0,113	1,30E-168
GZMK	3,24E-170	1,662596045	0,595	0,131	6,13E-166
GZMA	6,95E-138	1,250003309	0,691	0,233	1,32E-133
NKG7	1,60E-119	0,994623949	0,639	0,21	3,03E-115
HLA-B	4,11E-98	0,579518923	0,989	0,897	7,78E-94
CD8B	4,32E-93	1,211515518	0,378	0,083	8,19E-89
CST7	2,46E-90	1,076679745	0,509	0,167	4,66E-86
CCL4	8,10E-86	1,585357684	0,552	0,225	1,53E-81
HLA-C	4,67E-83	0,571180857	0,962	0,833	8,85E-79
GZMH	2,62E-82	0,99042891	0,34	0,07	4,97E-78
B2M	9,52E-82	0,510983591	0,989	0,923	1,80E-77
CD3D	2,30E-79	0,824790524	0,598	0,242	4,37E-75
PTPRC	6,32E-76	0,675454277	0,862	0,581	1,20E-71
HLA-A	1,57E-74	0,572678986	0,96	0,848	2,98E-70
CTSW	5,27E-73	0,848279524	0,556	0,227	9,99E-69
APOBEC3G	6,17E-73	0,963173958	0,442	0,152	1,17E-68
CD2	1,33E-71	0,842092876	0,633	0,299	2,52E-67
CD3E	4,48E-66	0,719657045	0,745	0,429	8,48E-62
IL2RB	6,27E-64	0,7786013	0,464	0,169	1,19E-59
IL32	6,67E-62	0,631358369	0,761	0,46	1,26E-57
ITM2C	5,16E-61	1,044877345	0,386	0,134	9,77E-57
TARP	1,05E-59	1,159130335	0,382	0,132	1,98E-55
SH2D1A	2,41E-56	0,833147648	0,29	0,077	4,57E-52
CD44	2,09E-53	0,63120927	0,761	0,518	3,97E-49
FYN	5,24E-52	0,736056757	0,593	0,326	9,93E-48
RAC2	9,19E-52	0,605528841	0,728	0,483	1,74E-47
PTPRCAP	1,63E-51	0,667396614	0,687	0,427	3,09E-47
RUNX3	2,81E-51	0,700689213	0,582	0,309	5,33E-47
HCST	2,72E-48	0,676651535	0,647	0,399	5,15E-44
BCL11B	5,50E-47	0,719208662	0,507	0,243	1,04E-42
LCP1	1,15E-46	0,508873735	0,847	0,661	2,17E-42
CD96	2,40E-45	0,727435154	0,422	0,183	4,55E-41
SAMD3	1,01E-41	0,731817717	0,272	0,087	1,92E-37
TC2N	8,45E-38	0,682172472	0,367	0,158	1,60E-33
TMSB4X	1,06E-37	0,32410458	0,988	0,92	2,02E-33
ARHGDIB	2,75E-37	0,414001647	0,887	0,757	5,21E-33
SLC38A1	5,36E-37	0,67332262	0,466	0,246	1,02E-32
WIPF1	1,95E-36	0,647194843	0,584	0,381	3,70E-32
CD48	1,73E-35	0,547143999	0,635	0,414	3,28E-31
ARAP2	4,54E-35	0,663922304	0,285	0,108	8,61E-31
LCK	2,48E-33	0,619088862	0,456	0,239	4,70E-29
CD53	2,63E-33	0,535174482	0,666	0,457	4,99E-29
PIP4K2A	1,05E-32	0,655657973	0,477	0,28	1,98E-28
MIAT	1,05E-32	0,557758892	0,374	0,172	1,99E-28
HLA-F	1,72E-32	0,628245728	0,556	0,363	3,25E-28
PRKCH	2,19E-32	0,676728273	0,399	0,206	4,14E-28
ITM2A	7,98E-32	0,728557822	0,407	0,213	1,51E-27
STK17A	1,30E-31	0,671952687	0,365	0,176	2,46E-27
PARP8	2,65E-31	0,649606945	0,37	0,181	5,03E-27

ITGAL	3,81E-31	0,602766644	0,519	0,32	7,22E-27
ACAP1	9,83E-31	0,608411287	0,378	0,187	1,86E-26
AKNA	1,43E-30	0,57875505	0,52	0,32	2,71E-26
RARRES3	3,62E-30	0,585368902	0,389	0,197	6,86E-26
ARHGEF1	1,23E-29	0,582337492	0,5	0,313	2,32E-25
PRF1	1,91E-29	0,356272192	0,293	0,118	3,62E-25
NLRC5	2,54E-29	0,604248049	0,341	0,165	4,81E-25
IL2RG	3,53E-29	0,499479137	0,741	0,581	6,69E-25
NFATC2	1,23E-28	0,663435759	0,385	0,205	2,33E-24
FAM102A	2,40E-28	0,62073805	0,338	0,164	4,56E-24
ETS1	2,75E-28	0,406431375	0,631	0,418	5,22E-24
GNG2	3,85E-28	0,624234473	0,333	0,161	7,30E-24
RASGRP1	7,00E-28	0,530300273	0,316	0,142	1,33E-23
EMB	1,40E-27	0,55746651	0,416	0,231	2,65E-23
SYNE2	6,87E-26	0,490706972	0,531	0,343	1,30E-21
SLC9A3R1	9,38E-26	0,570098139	0,35	0,179	1,78E-21
SLAMF7	1,00E-25	0,599579233	0,26	0,112	1,89E-21
CYFIP2	1,18E-25	0,557283022	0,443	0,26	2,24E-21
RAB27A	1,68E-25	0,681825798	0,434	0,269	3,19E-21
CDC42SE2	7,12E-25	0,516872297	0,487	0,31	1,35E-20
CD69	8,52E-25	0,670874426	0,292	0,137	1,61E-20
FYB1	1,10E-24	0,36168508	0,721	0,533	2,09E-20
TMA7	4,32E-24	0,44027723	0,702	0,583	8,19E-20
SARAF	4,84E-24	0,368326926	0,671	0,527	9,17E-20
STK17B	7,02E-24	0,516505598	0,558	0,383	1,33E-19
TBCD	7,41E-24	0,69598393	0,28	0,138	1,40E-19
BTG1	1,45E-23	0,430184446	0,687	0,552	2,74E-19
FNBP1	1,47E-23	0,516885336	0,54	0,379	2,78E-19
DDX5	7,15E-23	0,393756389	0,788	0,669	1,36E-18
sep-09	1,92E-22	0,510858639	0,57	0,422	3,65E-18
BTN3A2	1,95E-22	0,58523917	0,382	0,225	3,70E-18
CD7	1,51E-21	0,518839631	0,31	0,159	2,85E-17
CALM1	2,23E-21	0,27081268	0,91	0,824	4,22E-17
PFN1	2,98E-21	0,282272446	0,935	0,853	5,64E-17
STAT4	4,88E-21	0,483471511	0,26	0,123	9,24E-17
SPOCK2	9,73E-21	0,422958787	0,497	0,318	1,84E-16
sep-01	5,01E-20	0,55715855	0,306	0,165	9,50E-16
EVL	9,82E-20	0,486582443	0,492	0,34	1,86E-15
CXCR4	1,32E-19	0,593881571	0,405	0,251	2,50E-15
ARHGAP9	1,53E-19	0,51511481	0,308	0,167	2,89E-15
IKZF3	1,56E-19	0,415951037	0,549	0,398	2,95E-15
RASAL3	2,17E-19	0,506222761	0,281	0,144	4,12E-15
TMC8	2,67E-19	0,458428827	0,382	0,227	5,06E-15
SRGN	3,10E-19	0,255642841	0,833	0,695	5,87E-15
CD52	3,53E-19	0,33262024	0,634	0,444	6,69E-15
GLCCI1	4,00E-19	0,502153347	0,281	0,143	7,59E-15
CD247	6,60E-19	0,438139634	0,292	0,148	1,25E-14
NEK7	2,54E-18	0,531583771	0,28	0,156	4,81E-14
KIAA1551	4,17E-18	0,483004583	0,442	0,303	7,91E-14
ZAP70	5,38E-18	0,421053677	0,268	0,136	1,02E-13
IFITM1	5,57E-18	0,342398376	0,7	0,538	1,06E-13
PTP4A2	1,58E-17	0,421873141	0,684	0,597	2,99E-13
BTN3A1	2,06E-17	0,591113314	0,316	0,188	3,90E-13
PDCD4	3,41E-17	0,597543699	0,366	0,237	6,46E-13
NFATC3	6,56E-17	0,485417375	0,314	0,186	1,24E-12
SYNE1	7,52E-17	0,573234139	0,377	0,251	1,42E-12

SEMA4D	1,31E-16	0,527478296	0,37	0,236	2,48E-12
YWHAZ	1,56E-16	0,328909992	0,781	0,719	2,96E-12
CLEC2D	1,95E-16	0,425419724	0,5	0,358	3,70E-12
WNK1	3,85E-16	0,479024338	0,487	0,368	7,29E-12
ORC3	7,67E-16	0,436440179	0,296	0,167	1,45E-11
CNOT6L	1,93E-15	0,476711349	0,363	0,237	3,66E-11
MBNL1	2,48E-15	0,371718968	0,639	0,545	4,69E-11
TNFAIP3	2,52E-15	0,629314415	0,32	0,2	4,77E-11
ZFP36L2	4,36E-15	0,392534295	0,687	0,588	8,26E-11
CELF2	9,53E-15	0,430507192	0,565	0,459	1,81E-10
CCSER2	3,79E-14	0,533844061	0,321	0,207	7,18E-10
LPIN1	5,74E-14	0,499275849	0,318	0,204	1,09E-09
MYL12A	1,52E-13	0,326011499	0,676	0,595	2,88E-09
PPP2R5C	1,90E-13	0,46041382	0,381	0,266	3,60E-09
IKZF1	2,24E-13	0,423440767	0,411	0,284	4,24E-09
RASSF5	2,28E-13	0,408829143	0,423	0,302	4,33E-09
TBC1D10C	2,71E-13	0,33958094	0,252	0,14	5,14E-09
ABHD17A	3,99E-13	0,467704422	0,329	0,222	7,56E-09
ARL4C	4,85E-13	0,342312746	0,52	0,389	9,18E-09
PDE7A	7,25E-13	0,473231972	0,31	0,199	1,37E-08
OGT	1,14E-12	0,431708642	0,428	0,322	2,15E-08
ATM	2,12E-12	0,414215048	0,493	0,396	4,02E-08
PSME1	6,63E-12	0,384082583	0,533	0,443	1,26E-07
SPATA13	9,53E-12	0,430753715	0,272	0,17	1,81E-07
NKTR	1,06E-11	0,371150201	0,491	0,402	2,01E-07
ALOX5AP	2,75E-11	0,44560173	0,381	0,27	5,21E-07
OGA	5,63E-11	0,414238112	0,415	0,315	1,07E-06
ICAM3	7,91E-11	0,353230066	0,272	0,17	1,50E-06
NBEAL2	8,98E-11	0,429241632	0,282	0,183	1,70E-06
GBP5	9,56E-11	0,437210617	0,28	0,18	1,81E-06
CLDND1	1,84E-10	0,658195583	0,351	0,265	3,48E-06
SIGIRR	2,10E-10	0,398003601	0,312	0,215	3,98E-06
CCND2	2,21E-10	0,431185276	0,373	0,277	4,18E-06
APMAP	2,25E-10	0,408548317	0,259	0,169	4,26E-06
UTRN	3,52E-10	0,448252401	0,44	0,354	6,66E-06
BIN2	3,90E-10	0,323916662	0,337	0,233	7,39E-06
UBB	4,00E-10	0,311968424	0,638	0,578	7,57E-06
PSMB9	5,92E-10	0,334383864	0,29	0,197	1,12E-05
HNRNPH1	6,38E-10	0,342455824	0,603	0,551	1,21E-05
PLP2	6,91E-10	0,489312658	0,405	0,324	1,31E-05
DOCK8	1,16E-09	0,390317428	0,312	0,219	2,20E-05
VAMP2	1,47E-09	0,283856991	0,61	0,532	2,78E-05
FCMR	1,55E-09	0,279946358	0,309	0,204	2,93E-05
TMC6	2,00E-09	0,323898235	0,282	0,19	3,79E-05
CLEC2B	2,33E-09	0,466904521	0,317	0,233	4,42E-05
LEPROTL1	2,64E-09	0,385295006	0,312	0,222	5,01E-05
SH3KBP1	3,46E-09	0,380515517	0,41	0,323	6,56E-05
PREX1	4,14E-09	0,452639337	0,345	0,262	7,85E-05
PPDPF	4,45E-09	0,330542805	0,552	0,485	8,44E-05
JAK1	5,14E-09	0,329680348	0,487	0,405	9,74E-05
LCP2	5,30E-09	0,405352168	0,312	0,223	0,0001005
SUB1	8,72E-09	0,441616746	0,43	0,367	0,000165211
SUN2	8,88E-09	0,318642495	0,492	0,412	0,000168352
SLA	9,70E-09	0,36603064	0,28	0,193	0,000183744
STK10	1,06E-08	0,40988673	0,328	0,243	0,000200096
SET	1,26E-08	0,253968175	0,595	0,549	0,000239616

ANXA6	1,57E-08	0,309826669	0,435	0,352	0,000297159
SLAMF6	2,05E-08	0,306090406	0,312	0,218	0,000389005
PRKACB	2,25E-08	0,352606344	0,282	0,196	0,000425912
KDM5A	2,31E-08	0,423906267	0,332	0,252	0,000436983
MYL12B	3,52E-08	0,299846236	0,576	0,524	0,000667954
MYCBP2	3,69E-08	0,345101417	0,459	0,382	0,000699939
ID2	4,17E-08	0,372051055	0,414	0,333	0,000789518
HNRNPDL	4,24E-08	0,269591183	0,499	0,427	0,000802545
OPTN	6,34E-08	0,349526873	0,296	0,214	0,001201439
YPEL5	7,95E-08	0,416706939	0,347	0,27	0,001506222
AHNAK	1,13E-07	0,25110814	0,755	0,691	0,002138309
F2R	1,19E-07	0,347273555	0,265	0,185	0,002256693
UBL3	1,24E-07	0,447749487	0,255	0,184	0,002346692
RBMS1	1,74E-07	0,308683766	0,462	0,388	0,003292464
HNRNPA3	1,99E-07	0,359286771	0,454	0,396	0,00377272
RORA	2,01E-07	0,369593758	0,261	0,184	0,003803977
ADGRE5	2,23E-07	0,419938584	0,313	0,238	0,004234731
ANKRD44	2,69E-07	0,287671658	0,337	0,256	0,005090368
sep-06	2,82E-07	0,341292621	0,406	0,328	0,005340879
STK4	3,09E-07	0,382574142	0,454	0,39	0,005859794
IDS	3,71E-07	0,301609092	0,554	0,508	0,007024455
EML4	4,92E-07	0,335437449	0,366	0,288	0,009321371
AKAP13	5,07E-07	0,328531826	0,491	0,433	0,009605614
MACF1	5,70E-07	0,298782651	0,448	0,381	0,010796761
TNRC6B	5,75E-07	0,316599533	0,442	0,382	0,010900981
KMT2A	6,29E-07	0,361328645	0,426	0,364	0,011921413
PIN1	7,61E-07	0,297130672	0,493	0,441	0,014428629
MLLT6	7,85E-07	0,310592148	0,48	0,416	0,014873222
SF3B1	8,08E-07	0,285825658	0,548	0,51	0,015307752
ELOVL5	9,31E-07	0,300625953	0,265	0,191	0,017633046
SLFN5	9,74E-07	0,292473716	0,289	0,213	0,018462215
OST4	1,01E-06	0,267993586	0,455	0,391	0,019094654
ZC3HAV1	1,11E-06	0,325901008	0,452	0,39	0,02099108
ERBIN	1,13E-06	0,336829939	0,28	0,209	0,021460944
TAPBP	1,14E-06	0,313506558	0,524	0,483	0,021630889
NCOR1	1,82E-06	0,3160757	0,476	0,426	0,034482835
PTBP3	1,88E-06	0,345443099	0,386	0,324	0,035546951
CCND3	2,44E-06	0,283743783	0,285	0,215	0,046321305
MAPK1	2,83E-06	0,367943737	0,317	0,256	0,053648367
RAD21	2,95E-06	0,356481292	0,334	0,275	0,055918805
DEFA6	3,60E-06	0,319221366	0,265	0,196	0,068249131
NIN	3,92E-06	0,384747278	0,279	0,216	0,074359939
CAPZA1	3,94E-06	0,271305566	0,517	0,466	0,074691233
INPP5D	5,56E-06	0,303878738	0,288	0,22	0,105420152
RNF44	5,85E-06	0,287331275	0,288	0,221	0,110928151
IQGAP1	6,17E-06	0,283841471	0,574	0,553	0,116833802
PHIP	6,24E-06	0,373171906	0,416	0,369	0,118236345
CHD2	6,39E-06	0,265855648	0,33	0,263	0,121096906
SSR4	8,57E-06	0,296373976	0,405	0,357	0,162351526
GMFG	1,10E-05	0,268015077	0,406	0,339	0,208418616
DDX3Y	1,13E-05	0,371113304	0,255	0,192	0,213443299
FMNL1	1,22E-05	0,290804681	0,305	0,24	0,231540292
VPS13C	1,39E-05	0,338481209	0,411	0,37	0,262530561
ARHGAP30	1,80E-05	0,29699849	0,261	0,198	0,341554641
PIK3R1	1,84E-05	0,389146104	0,297	0,239	0,347905799
sep-07	2,11E-05	0,262569564	0,439	0,387	0,400554326

IL10RA	2,65E-05	0,267016711	0,26	0,194	0,501591617
KCNAB2	2,68E-05	0,284735511	0,325	0,259	0,508767415
DAZAP2	3,57E-05	0,258659749	0,532	0,498	0,676908632
C12orf57	3,84E-05	0,277969773	0,334	0,274	0,72729906
SNRPD2	4,12E-05	0,255421193	0,406	0,353	0,78000651
BIRC6	4,80E-05	0,343047626	0,29	0,238	0,909231381
PTK2B	4,90E-05	0,32234618	0,394	0,336	0,929154574
KLF13	5,34E-05	0,274605172	0,342	0,285	1
DIAPH1	6,49E-05	0,275377659	0,338	0,289	1
KLF5	6,80E-05	0,291852518	0,285	0,231	1
MCM2	6,81E-05	0,295718701	0,322	0,271	1
KMT2E	8,60E-05	0,269640215	0,412	0,368	1
TRIM22	0,000112382	0,32543532	0,321	0,273	1
ATP2B4	0,000155193	0,355295485	0,316	0,267	1
CNN2	0,000160183	0,276299167	0,439	0,411	1
SNHG6	0,000190012	0,304678801	0,381	0,338	1
CAPN2	0,000190773	0,303393312	0,408	0,373	1
TERF2IP	0,000207619	0,2976211	0,33	0,28	1
HECA	0,000225192	0,28360745	0,314	0,267	1
GNPTAB	0,0002617	0,298733707	0,285	0,237	1
PPP1R18	0,000279728	0,331041354	0,408	0,376	1
SLC4A7	0,000344402	0,279538835	0,261	0,213	1
AKAP9	0,000349863	0,290075451	0,408	0,375	1
SYNRG	0,000350141	0,26866394	0,321	0,273	1
DDX6	0,000399604	0,271633757	0,305	0,261	1
ASH1L	0,000446651	0,288480824	0,3	0,255	1
WAS	0,000477943	0,267306171	0,344	0,295	1
ATP5F1EP2	0,000517091	0,30676173	0,434	0,404	1
ANKRD11	0,000525319	0,295279118	0,366	0,326	1
LY6E	0,000607507	0,264610901	0,362	0,322	1
TRAM1	0,000636791	0,314088353	0,371	0,337	1
TNRC6C	0,000685128	0,262345227	0,261	0,215	1
RAP1A	0,000783094	0,316584986	0,386	0,354	1
ATRX	0,000993961	0,26984253	0,459	0,441	1
KLRD1	0,001236872	0,252674172	0,293	0,244	1
ZNF655	0,001326626	0,293989452	0,277	0,237	1
LPXN	0,001350533	0,280775061	0,279	0,233	1
SURF4	0,001524697	0,271715537	0,255	0,218	1
MBD2	0,001685756	0,284657821	0,333	0,297	1
USP34	0,001986901	0,279146183	0,312	0,276	1
STOM	0,002021871	0,265418586	0,411	0,379	1
ATP5MG	0,002029932	0,297233297	0,382	0,356	1
C9orf78	0,002049932	0,252331957	0,375	0,343	1
SRSF2	0,002116754	0,258796548	0,39	0,36	1
EVI2A	0,002260454	0,285065101	0,251	0,211	1
SPCS3	0,002422116	0,289281011	0,267	0,229	1
TRIM33	0,002558097	0,332667195	0,275	0,239	1
LIMD2	0,002746292	0,284013937	0,297	0,258	1
GCC2	0,003622826	0,296160687	0,251	0,215	1
PSME2	0,003933144	0,284330057	0,351	0,328	1
PSMA5	0,0047249	0,321877149	0,255	0,222	1
TSC22D3	0,005503163	0,261374537	0,386	0,364	1
KAT6A	0,005928392	0,257523302	0,318	0,294	1
RBM26	0,007610079	0,27979025	0,257	0,23	1

Cluster 1 marker genes

gene	p_val	avg_logFC	pct.1	pct.2	p_val_adj
LTB	2,12E-126	157.144.041.071.028	0.629	0.165	4,02E-122
MAL	8,25E-107	125.392.674.394.625	0.274	0.017	1,56E-102
EEF1A1	1,80E-100	0.698095998732645	0.995	0.956	3,41E-96
IL7R	2,56E-95	138.451.302.067.287	0.657	0.232	4,84E-91
TPT1	1,23E-72	0.80562218509916	0.984	0.903	2,34E-68
IL32	4,67E-57	0.85616148117416	0.81	0.47	8,84E-53
LEF1	1,87E-51	107.509.163.532.902	0.31	0.069	3,54E-47
TCF7	1,27E-48	106.486.977.591.449	0.49	0.186	2,42E-44
PABPC1	3,72E-48	0.677236432515811	0.913	0.808	7,05E-44
UBA52	1,70E-43	0.552834841777747	0.937	0.833	3,23E-39
LDHB	3,81E-43	0.920549564308936	0.571	0.289	7,22E-39
SPOCK2	4,77E-43	0.839153459744087	0.62	0.304	9,04E-39
FAU	4,44E-40	0.569355783135492	0.929	0.795	8,42E-36
B2M	1,34E-39	0.444519598316268	0.99	0.927	2,53E-35
DGKA	3,54E-39	0.958947469842925	0.517	0.237	6,71E-35
EEF1G	6,86E-38	0.690270615662846	0.834	0.691	1,30E-33
RACK1	2,92E-36	0.597947283440392	0.895	0.766	5,53E-32
NPM3	3,47E-35	0.891955615940496	0.584	0.327	6,58E-31
EEF1B2	6,43E-34	0.750758411440551	0.815	0.645	1,22E-29
IFITM1	8,04E-34	0.63801220608809	0.779	0.532	1,52E-29
NOP53	3,06E-32	0.712700410189171	0.728	0.526	5,80E-28
ETS1	4,69E-32	0.729955370459341	0.692	0.419	8,89E-28
BCL11B	4,00E-31	0.701966612965507	0.537	0.254	7,57E-27
SARAF	4,33E-31	0.734741067332902	0.733	0.523	8,20E-27
FLT3LG	1,58E-30	0.845934373958874	0.369	0.136	2,99E-27
CD3E	2,30E-30	0.555758009328496	0.744	0.45	4,36E-25
TOMM7	5,74E-25	0.627205258333034	0.751	0.564	1,09E-20
FYB1	8,38E-25	0.638696952637242	0.758	0.538	1,59E-20
CD52	1,18E-25	0.560651265114728	0.709	0.441	2,24E-20
CD3D	4,99E-24	0.634609002205933	0.551	0.276	9,45E-20
BIRC3	3,54E-21	0.753531284772392	0.394	0.179	6,70E-17
ITK	1,03E-20	0.722198365136875	0.324	0.123	1,95E-16
CD48	2,07E-20	0.587274530530593	0.664	0.423	3,92E-16
LCK	9,87E-20	0.591730804968257	0.488	0.246	1,87E-15
CYLD	5,06E-18	0.690182120619434	0.477	0.263	9,59E-14
FXYD5	5,39E-18	0.584510195124097	0.688	0.501	1,02E-13
ADD3	7,41E-18	0.685197538884553	0.531	0.316	1,40E-13
CD2	1,24E-17	0.538745416016253	0.587	0.331	2,36E-13
GOLGA8A	1,51E-17	0.670092169378183	0.432	0.218	2,86E-13
BTF3	4,14E-17	0.555586271210596	0.716	0.541	7,85E-13
HLA-B	1,03E-16	0.293984209252693	0.981	0.905	1,96E-12
NAP1L1	1,54E-17	0.509328266418636	0.761	0.607	2,91E-12
CDC14A	8,02E-16	0.628780745177581	0.26	0.092	1,52E-11
PAG1	5,17E-15	0.721871430577827	0.474	0.276	9,80E-11
TMSB10	1,09E-13	0.364924830860681	0.927	0.856	2,07E-09
SYNE2	1,40E-13	0.617098101322966	0.554	0.351	2,66E-09
FCMR	3,73E-12	0.538236988531943	0.394	0.193	7,08E-09
COX7C	1,00E-11	0.498944678451461	0.74	0.602	1,90E-07
GATA3	1,46E-11	0.62279506322086	0.258	0.099	2,76E-07
RAC2	3,93E-11	0.458510616792661	0.713	0.503	7,44E-07
CD6	7,83E-10	0.547229404471187	0.272	0.114	1,48E-05
AES	1,40E-09	0.530240528179756	0.672	0.53	2,64E-05
ARHGDIB	2,99E-09	0.392799577850185	0.859	0.771	5,67E-05

EMB	6,20E-09	0.511912632694333	0.432	0.24	1,17E-04
ZC3HAV1	7,30E-09	0.609498323944162	0.544	0.375	1,38E-04
IKZF1	2,16E-08	0.489477436215042	0.469	0.28	4,09E-05
NACA	2,78E-08	0.534187957223636	0.674	0.56	5,27E-04
PIK3IP1	5,68E-08	0.623144243353168	0.338	0.177	1,08E-03
PBXIP1	6,42E-08	0.539985141573451	0.476	0.308	1,22E-03
GSTK1	1,87E-08	0.588654067923213	0.495	0.333	3,54E-03
PTPRC	4,32E-07	0.348636573445034	0.791	0.615	8,19E-03
CD4	7,05E-07	0.485093653450563	0.537	0.35	1,34E-02
BCL2	7,13E-07	0.66155304216868	0.293	0.141	1,35E-02
OCIAD2	1,33E-06	0.548094243623815	0.284	0.134	2,52E-02
SORL1	2,17E-06	0.601963060156092	0.479	0.32	4,12E-02
FAM102A	9,65E-06	0.491447630985552	0.34	0.175	1,83E-01
SELL	2,82E-05	0.842285361991076	0.376	0.225	5,35E-01
HLA-A	3,39E-05	0.263410423749277	0.939	0.86	6,43E-01
SLC38A1	3,63E-05	0.464659145393682	0.444	0.266	6,88E-01
ORC3	4,16E-06	0.504920114136401	0.326	0.169	7,89E-01
PTPRCAP	5,11E-06	0.307174023428134	0.66	0.45	9,68E-01
COMMD6	5,72E-05	0.543743072173291	0.488	0.34	1,08E+00
HINT1	6,26E-05	0.55848562588284	0.517	0.371	1,19E+00
EML4	9,85E-05	0.522155769744632	0.441	0.278	1,87E+00
SNHG8	1,10E-04	0.708711874727615	0.361	0.219	2,09E+00
MYL12A	1,12E-04	0.394915321262673	0.716	0.592	2,12E+00
HSPA8	1,21E-04	0.428386873063158	0.69	0.571	2,29E+00
S100A4	1,45E-04	0.333057261238722	0.815	0.665	2,74E+00
sep-09	1,99E-04	0.420952793633241	0.589	0.428	3,76E+00
EIF3E	2,11E-04	0.570066522791854	0.533	0.394	4,00E+00
CD44	2,67E-04	0.373201424219831	0.699	0.548	5,06E+00
sep-06	4,92E-04	0.479300024006533	0.483	0.317	9,32E+00
EEF1D	1,13E-03	0.465487500798445	0.618	0.506	2,15E+01
CDC42SE2	1,36E-03	0.47885418431748	0.484	0.322	2,59E+01
EIF4B	3,49E-03	0.417580519810215	0.733	0.634	6,61E+01
TESPA1	6,81E-03	0.471176821431875	0.282	0.142	1,29E+01
EVL	9,28E-03	0.448366408645917	0.503	0.347	1,76E+02
TRAF3IP3	1,04E-02	0.430698784534575	0.254	0.123	1,98E+02
CYTIP	2,69E-02	0.494816144174948	0.336	0.191	5,10E+02
ZAP70	8,79E-02	0.431327472140638	0.28	0.142	1,67E+03
RAPGEF6	9,07E-02	0.556083065426687	0.27	0.143	1,72E+03
RORA	9,08E-02	0.472120777713154	0.315	0.177	1,72E+02
STK17B	9,72E-02	0.4547288654999	0.542	0.398	1,84E+03
ANKRD12	1,43E-01	0.44015430568146	0.666	0.562	2,70E+03
CD247	1,46E-01	0.333387804398844	0.3	0.156	2,77E+03
SNHG6	1,48E-01	0.556606864787785	0.463	0.323	2,81E+02
CALM1	1,68E-02	0.282815212329496	0.92	0.828	3,19E+03
RASGRP1	1,74E-01	0.427708043949446	0.296	0.158	3,30E+03
SLC2A3	1,99E-01	0.468034818387963	0.43	0.281	3,77E+03
PGGHG	3,80E-01	0.523573389523619	0.258	0.134	7,19E+03
EIF1	7,34E-01	0.301981791410304	0.833	0.775	1,39E+04
TMA7	1,10E+00	0.338316799218215	0.697	0.592	2,09E+04
NCK2	1,96E+00	0.564834783491658	0.289	0.167	3,71E+04
GAS5	2,08E+00	0.60175293511036	0.39	0.263	3,95E+04
LEPROTL1	4,35E+00	0.501208852663758	0.35	0.22	8,25E+04
MYCBP2	7,86E+00	0.451856960634516	0.502	0.378	1,49E+05
IL2RG	8,51E+00	0.303278811929494	0.706	0.6	1,61E+05
GMFG	1,15E+01	0.440455011028767	0.463	0.331	2,19E+05
CD53	1,33E+00	0.368700646863109	0.613	0.482	2,53E+05

EIF3H	7,27E+01	0.449178132684984	0.491	0.38	1,38E+06
MIAT	1,32E+02	0.474536003966222	0.324	0.196	2,50E+06
CD28	1,34E+02	0.466441203616746	0.27	0.154	2,53E+06
DEFA6	1,49E+02	0.429925547924406	0.314	0.19	2,82E+06
STMN3	1,51E+02	0.518119797730768	0.26	0.148	2,86E+06
LIMD2	1,62E+02	0.427621711833666	0.371	0.244	3,06E+06
SATB1	1,86E+02	0.558393244278967	0.308	0.192	3,52E+06
ZFAS1	2,20E+02	0.4987542302699	0.481	0.373	4,16E+06
TMC8	2,96E+02	0.425318912998725	0.371	0.24	5,60E+06
BTG1	3,90E+02	0.305918372849975	0.667	0.565	7,38E+05
HNRNPH1	5,21E+02	0.287001956404472	0.645	0.546	9,86E+05
TC2N	6,04E+02	0.458479471555945	0.303	0.185	1,14E+07
MBNL1	9,44E+02	0.350460484819275	0.636	0.552	1,79E+07
HNRNPA1	1,38E+03	0.394664092583542	0.551	0.446	2,62E+07
EEF2	3,41E+03	0.324637161761923	0.725	0.657	6,46E+07
ANKRD44	3,77E+03	0.406480278596833	0.375	0.253	7,14E+07
NPM1	4,20E+03	0.415153088335536	0.61	0.541	7,96E+07
SMCHD1	5,68E+02	0.463857830011387	0.378	0.267	1,08E+07
HECA	1,11E+04	0.443744795614307	0.368	0.258	2,10E+08
SNHG5	1,15E+04	0.472177632997831	0.491	0.388	2,18E+07
RGCC	1,28E+04	0.464048054020023	0.284	0.175	2,42E+08
PFDN5	1,31E+04	0.332496466590868	0.615	0.532	2,48E+08
NKTR	1,87E+04	0.475150551165358	0.497	0.406	3,54E+08
CD69	2,06E+04	0.397677255428901	0.263	0.153	3,91E+08
TBC1D10C	2,39E+04	0.34586891977919	0.254	0.147	4,53E+08
CCND2	2,45E+04	0.423530825093809	0.39	0.279	4,65E+08
ATXN7	2,51E+04	0.423546483539898	0.287	0.182	4,75E+08
ICAM3	3,49E+04	0.383812878764554	0.282	0.175	6,61E+08
SEMA4D	4,02E+04	0.345756216401031	0.362	0.247	7,61E+08
FAM107B	4,21E+03	0.467171602453152	0.375	0.271	7,97E+07
JAK3	4,52E+03	0.409966912645609	0.286	0.181	8,56E+08
SIGIRR	5,76E+04	0.371225738250478	0.326	0.219	1,09E+09
PRKY	9,35E+04	0.452351508822367	0.298	0.197	1,77E+09
YWHAB	9,99E+04	0.271585894629688	0.803	0.764	1,89E+09
CYTH1	1,09E+05	0.441971662471439	0.329	0.226	2,07E+09
OST4	1,22E+05	0.418394583446248	0.479	0.39	2,32E+09
CRYBG1	1,96E+05	0.449090095797816	0.26	0.163	3,72E+09
SELPLG	2,06E+05	0.355760539067292	0.31	0.206	3,90E+08
PLP2	3,42E+04	0.338372532574007	0.429	0.325	6,48E+08
SOD1	4,16E+04	0.469169608572608	0.443	0.358	7,88E+09
FOXO1	4,18E+05	0.476333729988345	0.329	0.228	7,92E+09
SAMSN1	4,42E+05	0.359076472500375	0.253	0.155	8,38E+09
GTF3A	6,17E+05	0.415733620607188	0.277	0.184	0.000116978704738027
FYN	8,27E+05	0.254162883629942	0.49	0.365	0.000156780283194657
FOXP1	1,18E+06	0.350581384323359	0.573	0.497	0.00022320365350844
RASSF5	1,87E+06	0.319590122245318	0.416	0.312	0.000353685567867987
CYFIP2	1,91E+06	0.269272888814407	0.397	0.282	0.00036111488151454
SF3B1	2,13E+06	0.356163859479549	0.566	0.509	0.000403842554909944
SUN2	2,15E+06	0.311026694735081	0.514	0.413	0.000407076487997507
YWHAZ	2,25E+06	0.250381192510238	0.753	0.729	0.000426734048791885
NOP58	2,57E+06	0.421481168060631	0.319	0.227	0.000487075945556837
PSIP1	3,43E+06	0.44577688380503	0.296	0.209	0.000649682564158443
ARL6IP5	3,74E+06	0.376886461709066	0.549	0.472	0.000709583829713064
SSR2	3,84E+06	0.368487621976242	0.434	0.353	0.000727060043148597
CD96	4,01E+06	0.322556692923641	0.324	0.22	0.000760076028899714
RBL2	4,03E+06	0.328292274335195	0.256	0.166	0.000764297731662133

HLA-F	4,36E+06	0.25473288467734	0.493	0.389	0.000825822802559878
UBALD2	4,74E+05	0.479355946617677	0.28	0.199	0.000897986632056214
CLEC2D	6,05E+06	0.273924835050281	0.477	0.372	0.00114613848750754
ATP5MC2	7,16E+06	0.310295122785921	0.589	0.518	0.00135758509481689
RIPOR2	1,14E+07	0.334500074913899	0.387	0.292	0.00216297993140014
RAP1A	1,16E+07	0.330647284207586	0.43	0.346	0.0022027323158621
MAP3K1	1,18E+07	0.408387835094845	0.258	0.175	0.00223471236670987
ANP32B	1,34E+07	0.378861447413657	0.43	0.343	0.00254000697596196
LRRC75A-A	1,55E+07	0.445301942543439	0.409	0.331	0.00294348933904111
ACAP1	1,62E+07	0.310954104430938	0.31	0.214	0.00307280451815461
NAP1L4	1,92E+07	0.339552690028481	0.275	0.19	0.00363430648451956
FKBP5	1,98E+07	0.362468765819638	0.368	0.282	0.00374634274234577
sep-01	2,17E+07	0.286306160820727	0.274	0.182	0.00411526100034923
ATM	2,23E+07	0.367235962291174	0.484	0.404	0.00423385876144866
EIF3K	2,35E+07	0.371631165732713	0.413	0.34	0.00444586910814867
OGT	2,60E+07	0.361820974037281	0.418	0.331	0.00492181316733143
DDX17	4,40E+07	0.27013441749307	0.617	0.557	0.00833618717346435
HNRNPDL	5,12E+07	0.338839243325837	0.505	0.43	0.0096979177089466
KIAA1551	6,37E+07	0.330513710977769	0.406	0.32	0.0120628490061638
APRT	6,89E+07	0.370002561094745	0.3	0.222	0.0130489360571844
RNF44	1,03E+08	0.339099054860867	0.303	0.222	0.0194855684908802
PDE7A	1,80E+08	0.284436509622574	0.298	0.209	0.0341043722468261
ANXA1	1,82E+08	0.349507120949263	0.542	0.497	0.0345213065133528
STAT1	2,33E+08	0.480874886812681	0.334	0.264	0.0441288796052391
DIAPH1	2,66E+08	0.270261900849448	0.369	0.285	0.0504093962902421
TSC22D3	2,69E+07	0.284582010307892	0.434	0.355	0.0510052695221152
TMC6	2,97E+08	0.354305915294831	0.274	0.198	0.0562931323206366
EIF3F	3,29E+08	0.334993826845206	0.409	0.342	0.062406027907497
ARHGEF1	3,40E+08	0.289002491447319	0.422	0.342	0.064380765215146
PSME1	3,75E+08	0.286283710262087	0.51	0.453	0.071086151495388
TRIM22	3,86E+08	0.318787579386535	0.345	0.271	0.0731104839417794
PIK3CD	3,86E+08	0.329728823896171	0.265	0.188	0.0731804441869697
RARRES3	4,24E+08	0.271707052520853	0.31	0.226	0.0803986637076254
EIF3L	6,10E+08	0.351783687336044	0.47	0.412	0.115576245161559
STK4	6,96E+08	0.253985541904189	0.465	0.392	0.131924786413093
UBE2D2	7,03E+07	0.266668371895341	0.34	0.265	0.133185308187987
STX16	9,85E+08	0.308213758508826	0.329	0.261	0.186748309575642
LPXN	1,17E+09	0.312202429533254	0.307	0.23	0.221113743488813
KMT2A	1,19E+09	0.296579185540785	0.436	0.366	0.225580074206013
ATP5MG	1,21E+09	0.313813349540911	0.411	0.351	0.229172042804472
MAF	1,26E+09	0.290059118640187	0.294	0.22	0.238066550748141
UXT	1,65E+08	0.381469995967559	0.314	0.25	0.311829040239687
NR3C1	2,07E+09	0.295759369036547	0.441	0.375	0.392231090296397
C6orf48	2,09E+08	0.374604870181079	0.326	0.264	0.396440532433142
GPSM3	2,15E+09	0.298829700090323	0.368	0.294	0.408170209412714
STK38	2,36E+09	0.287774312703465	0.26	0.187	0.446623996544359
HIGD2A	2,47E+09	0.344860786518133	0.298	0.235	0.468777982158811
ZNF292	2,65E+09	0.299780605776313	0.301	0.234	0.501596714796372
ATP5PD	2,74E+09	0.353728675348962	0.319	0.257	0.519839256543202
UQCRB	2,88E+09	0.363278931634577	0.397	0.347	0.545346654078006
CCND3	2,89E+09	0.330670298268303	0.287	0.219	0.547849483062959
CNOT6L	2,90E+09	0.260268386345459	0.326	0.253	0.548839497800052
TMEM173	3,17E+09	0.355324915069249	0.315	0.253	0.601442039944915
SH3KBP1	4,54E+09	0.28340843503366	0.401	0.331	0.859932110230531
TNFAIP3	4,65E+09	0.330661127917284	0.284	0.216	0.881096329366418
ABLIM1	6,01E+09	0.312844183809927	0.26	0.192	1

CIB1	6,13E+09	0.307928817945177	0.329	0.268	1
EZR	9,07E+09	0.25680761612483	0.324	0.257	1
ERAP2	0.0001052	0.35420128572351	0.294	0.236	1
IL6R	0.0001218	0.300932523644206	0.27	0.21	1
GCC2	0.0001372	0.304837929016699	0.272	0.213	1
RAN	0.0001447	0.31241593499505	0.331	0.279	1
SNRNP70	0.0001614	0.333356593195442	0.378	0.33	1
SNRPD2	0.0002007	0.294977472443685	0.401	0.357	1
C12orf57	0.0003241	0.262041827051947	0.336	0.278	1
ARHGAP4	0.0003402	0.280817318021938	0.256	0.199	1
CCT8	0.0003467	0.355590135812694	0.254	0.206	1
SYNRG	0.0003592	0.257567329626936	0.328	0.274	1
ARHGEF6	0.0003930	0.270533643052372	0.268	0.214	1
MCM2	0.0004091	0.291218996843865	0.328	0.273	1
NSA2	0.0004246	0.350509390124223	0.319	0.273	1
CD47	0.0004547	0.282116189000849	0.404	0.364	1
USP15	0.0004680	0.275498081277832	0.254	0.199	1
ITGB1	0.0004986	0.277471610202887	0.535	0.493	1
ANAPC16	0.0006487	0.266469483374073	0.422	0.379	1
OXA1L	0.0007788	0.313516977727452	0.253	0.203	1
NDUFA4	0.0013184	0.257137018710211	0.408	0.371	1
COX6C	0.0013666	0.256160829868171	0.404	0.367	1
ATP5F1A	0.0015637	0.283280594806656	0.376	0.343	1
CNN2	0.0016881	0.268293730950485	0.448	0.411	1
CBFB	0.0016923	0.313241893061812	0.274	0.23	1
KIAA0319L	0.0018012	0.278996392800784	0.265	0.217	1
ARHGAP45	0.0020405	0.281518116076076	0.258	0.213	1
KIAA1109	0.0023298	0.274420726237599	0.265	0.217	1
XPO6	0.0023749	0.265410100455939	0.268	0.226	1
LCP2	0.0024989	0.288248355051665	0.28	0.236	1
PNN	0.0028135	0.303850634122341	0.268	0.228	1
SKP1	0.0029776	0.257158776112381	0.418	0.394	1
PRPF8	0.0030755	0.263958481210918	0.307	0.268	1
EIF2S3	0.0045852	0.251788902404644	0.355	0.326	1
ANXA6	0.0055157	0.288357101245249	0.395	0.366	1
EIF3G	0.0062748	0.290733258770209	0.263	0.23	1
CAB39	0.0076989	0.259094620778089	0.277	0.246	1
FNBP4	0.0084969	0.271022375354383	0.268	0.23	1
TOMM20	0.0090086	0.251642851831249	0.321	0.294	1
GBP2	0.0090375	0.328754092387976	0.254	0.222	1

Cluster 2 marker genes

gene	p_val	avg_logFC	pct.1	pct.2	p_val_adj
LOC10013	6,18E-142	179.661.645.343.455	1	0.965	1,17E-137
PGM5P2	3,09E-130	187.647.326.929.577	0.979	0.765	5,85E-126
LOC64340	5,15E-130	15.446.547.100.906	0.958	0.688	9,76E-126
TMEM212	6,12E-130	168.353.830.919.794	0.963	0.726	1,16E-124
ODF2L	4,06E-127	164.844.355.657.339	0.958	0.767	7,69E-123
MAB21L3	5,16E-127	173.591.743.005.674	0.982	0.895	9,77E-123
CFLAR	5,88E-126	155.566.913.681.664	0.966	0.83	1,11E-121
CCDC144B	4,74E-124	179.417.962.700.851	0.937	0.645	8,98E-120
XAF1	1,71E-123	160.439.954.681.549	0.953	0.745	3,24E-119
ZNF471	3,03E-121	171.194.304.644.229	0.929	0.651	5,75E-117
ESRG	2,37E-120	166.209.031.050.408	0.863	0.385	4,50E-115
TSIX	3,10E-119	163.197.741.953.424	0.934	0.62	5,88E-115
DDHD1	6,37E-118	151.450.831.850.316	0.916	0.55	1,21E-113
ZNF480	2,90E-113	154.083.696.217.484	0.908	0.577	5,50E-109
F5	1,61E-109	169.208.714.730.205	0.926	0.639	3,05E-105
SP100	2,52E-103	138.140.669.861.817	0.945	0.721	4,77E-99
ANKRD20A	1,86E-102	138.834.527.368.835	0.932	0.676	3,53E-98
ASTN2	2,88E-102	183.143.014.923.343	0.866	0.466	5,47E-98
USP33	7,44E-98	167.732.423.636.282	0.897	0.597	1,41E-92
ACADSB	1,22E-94	151.771.560.511.959	0.821	0.426	2,30E-89
HCG18	1,37E-92	150.293.458.200.414	0.829	0.449	2,59E-88
TPTE2P1	2,16E-92	145.353.299.435.587	0.774	0.357	4,09E-88
NXNL2	1,77E-91	154.726.655.102.991	0.718	0.283	3,35E-87
SAA1	3,53E-91	15.195.824.577.724	0.75	0.311	6,68E-87
POTEM	2,49E-90	163.342.982.557.401	0.766	0.377	4,73E-87
LINC00550	3,34E-87	145.305.270.714.744	0.616	0.187	6,33E-84
ZNF37A	3,48E-87	138.673.001.929.002	0.766	0.364	6,59E-83
SBF2-AS1	1,90E-84	143.942.972.521.474	0.721	0.303	3,60E-80
LINC00670	3,12E-84	153.003.811.418.737	0.682	0.266	5,92E-80
ZNF37BP	1,06E-82	143.444.032.400.175	0.8	0.439	2,01E-78
RAD21-AS	3,01E-80	147.160.119.733.212	0.737	0.346	5,70E-76
XRCC2	6,67E-80	143.375.502.830.042	0.729	0.337	1,26E-75
NFX1	7,26E-76	128.132.370.796.834	0.779	0.415	1,38E-71
HYDIN	2,18E-75	131.254.829.096.608	0.787	0.428	4,12E-71
ENTPD1	1,52E-74	131.422.286.884.456	0.779	0.415	2,88E-70
TIGD1	4,58E-74	131.584.386.760.234	0.771	0.402	8,67E-70
ZNF829	2,00E-72	136.648.102.540.106	0.621	0.225	3,79E-68
NKAIN3-IT	6,25E-72	128.579.876.914.318	0.671	0.276	1,18E-67
HEXA-AS1	3,32E-71	117.095.815.799.522	0.45	0.098	6,30E-67
LOC28643	2,18E-70	141.595.669.650.921	0.747	0.387	4,13E-66
LOC10050	7,61E-70	132.581.246.807.411	0.589	0.203	1,44E-64
FAM111B	3,49E-67	130.810.257.796.255	0.545	0.17	6,62E-63
ADH4	1,34E-63	124.699.703.356.454	0.634	0.254	2,54E-59
ALPK1	2,85E-63	125.414.152.847.443	0.597	0.227	5,40E-59
ARHGEF26	6,15E-63	141.874.692.339.749	0.682	0.333	1,17E-58
MCFD2	1,00E-61	125.635.514.391.481	0.737	0.406	1,90E-57
LINC00547	1,11E-59	127.759.917.305.789	0.621	0.251	2,10E-55
KLHL5	1,74E-59	113.172.261.667.326	0.761	0.448	3,30E-55
ORC4	3,82E-59	124.905.732.701.376	0.663	0.306	7,24E-55
ASB4	4,71E-59	11.820.672.819.726	0.613	0.235	8,92E-55
CTSC	1,10E-58	121.149.184.901.886	0.789	0.541	2,09E-54
SCD5	2,22E-58	140.400.417.874.309	0.568	0.212	4,20E-54
HTRA4	4,52E-58	123.889.013.489.592	0.582	0.216	8,56E-54

LINC-ROR	2,77E-57	112.835.423.200.868	0.384	0.083	5,24E-53
ARRDC3-A!	5,85E-56	121.443.665.873.137	0.645	0.277	1,11E-51
LOC10013(	3,62E-55	129.389.102.999.609	0.571	0.214	6,87E-51
UTS2B	1,03E-54	124.997.283.910.193	0.379	0.082	1,96E-50
ACBD7	3,89E-54	110.132.922.848.387	0.492	0.149	7,36E-50
RAB3IP	2,60E-53	10.967.679.319.147	0.621	0.268	4,92E-49
LOC64621	5,41E-53	119.909.338.118.797	0.574	0.224	1,03E-48
PTCSC3	1,15E-52	121.085.596.893.566	0.495	0.157	2,17E-48
PLEKHA5	1,99E-52	123.301.095.891.206	0.584	0.244	3,78E-49
ADGRG4	7,10E-52	110.735.810.695.223	0.468	0.134	1,35E-47
ULBP1	7,48E-52	11.003.987.109.642	0.416	0.107	1,42E-47
LOC28458:	1,29E-51	114.962.866.218.257	0.461	0.136	2,45E-47
SPART	1,92E-51	114.768.030.815.045	0.539	0.197	3,63E-47
LINC00842	9,00E-50	120.443.873.385.311	0.361	0.08	1,70E-45
REXO1L1P	4,18E-49	104.768.785.426.701	0.405	0.104	7,92E-45
MC2R	9,05E-48	119.580.023.640.404	0.376	0.092	1,72E-43
CD300E	1,01E-47	119.891.543.025.011	0.382	0.094	1,91E-43
SHISA9	2,17E-47	122.645.609.854.376	0.561	0.234	4,12E-43
ANKLE1	3,43E-45	0.982664561396965	0.424	0.121	6,50E-41
TLR8-AS1	9,58E-45	121.157.495.367.924	0.392	0.106	1,82E-40
ABCA9	2,63E-44	103.792.493.719.672	0.442	0.134	4,99E-40
ZNF441	3,36E-45	106.090.310.222.305	0.45	0.141	6,36E-40
CDC27	5,68E-44	134.115.005.083.419	0.561	0.238	1,08E-39
C2orf91	9,74E-44	102.095.407.178.861	0.484	0.164	1,85E-39
MRPL36	2,97E-43	11.925.889.324.906	0.521	0.207	5,62E-39
LOC10037!	4,02E-43	113.019.077.420.938	0.484	0.174	7,61E-39
APOL4	2,42E-43	115.642.236.749.029	0.482	0.172	4,58E-38
LOC22072!	1,61E-40	113.064.137.606.663	0.437	0.144	3,04E-37
ANKRD36E	2,28E-40	0.887641339052492	0.311	0.067	4,32E-37
LINC00346	3,01E-40	116.842.793.272.905	0.5	0.196	5,70E-36
ANKRD30E	3,14E-40	0.949265699166354	0.258	0.045	5,95E-36
LINC00652	4,10E-40	112.020.889.583.909	0.403	0.123	7,77E-36
BAAT	4,19E-40	100.394.411.900.264	0.403	0.117	7,93E-36
UGT2B15	1,30E-41	0.933621304235905	0.371	0.098	2,46E-35
RAB12	1,45E-40	0.801060425688632	0.916	0.833	2,76E-35
COX10-AS:	6,37E-39	100.578.091.078.291	0.35	0.09	1,21E-34
SPDYE7P	8,38E-39	112.900.921.315.593	0.453	0.153	1,59E-34
GPLD1	1,15E-38	100.035.518.304.833	0.397	0.117	2,17E-34
PCA3	1,54E-38	101.617.106.957.092	0.374	0.103	2,91E-34
LOC10050(	1,56E-38	0.79413121398361	0.266	0.05	2,96E-34
DYNAP	5,56E-38	102.329.288.182.765	0.368	0.101	1,05E-33
NUDT9	6,96E-37	100.944.144.691.072	0.518	0.213	1,32E-33
TECRL	1,34E-36	0.918792810971141	0.345	0.09	2,53E-32
RNF217-AS	4,28E-36	0.830894257640131	0.279	0.058	8,11E-32
LEPR	9,97E-36	132.603.255.240.855	0.75	0.478	1,89E-32
TSG1	1,24E-35	103.228.670.194.863	0.395	0.123	2,35E-31
EPPIN	1,52E-35	111.490.066.441.672	0.397	0.126	2,89E-31
APOL6	2,59E-35	0.988394170078353	0.708	0.474	4,91E-31
SLC6A20	3,18E-35	0.993784867642744	0.429	0.144	6,03E-31
LINC00410	7,59E-35	0.78556680271687	0.276	0.058	1,44E-30
ZNF667	9,58E-35	0.931594984815901	0.447	0.155	1,82E-30
RNF213	9,94E-35	0.820452299419895	0.821	0.658	1,88E-30
LRRC27	1,41E-34	0.980139710344378	0.479	0.181	2,67E-31
FGF5	1,73E-34	0.790803878509434	0.263	0.052	3,28E-30
ZNF662	4,81E-34	103.020.051.704.539	0.5	0.204	9,12E-30
HLA-L	1,70E-33	0.84255475166441	0.287	0.065	3,23E-29

LPAL2	1,72E-33	100.246.009.088.934	0.458	0.169	3,25E-29
FOXL2NB	5,86E-33	0.857648122798916	0.332	0.088	1,11E-28
ABCC9	5,69E-32	0.86192811059919	0.316	0.081	1,08E-27
ZNF445	1,80E-31	0.97322982022619	0.429	0.156	3,41E-27
LOC15727:	2,64E-31	0.792607839094628	0.255	0.053	5,00E-27
TRMT9B	3,69E-31	0.919928797851716	0.418	0.143	7,00E-27
MGC2734!	6,05E-31	0.985562637004093	0.403	0.138	1,15E-26
FAM227A	9,14E-31	0.96457817919384	0.529	0.233	1,73E-27
LOC72973:	2,17E-30	0.995951835661355	0.434	0.161	4,11E-26
FECH	2,45E-30	0.989038233797482	0.458	0.18	4,64E-26
DZIP3	6,08E-30	0.922515442914052	0.453	0.173	1,15E-25
CTCFL	1,08E-30	0.937286105434639	0.326	0.091	2,05E-25
LINC0064!	1,13E-28	0.842927009530652	0.326	0.093	2,15E-24
PRR11	3,14E-28	0.916773056327827	0.576	0.285	5,96E-25
LSAMP	4,05E-29	0.918175613383304	0.345	0.103	7,68E-24
LINC00663	1,02E-27	0.85737734515907	0.363	0.114	1,94E-23
LOC10028!	2,52E-27	0.902788465396388	0.337	0.102	4,78E-23
CEP126	4,94E-27	0.931616262569059	0.45	0.179	9,35E-23
LOC44030(	6,41E-27	0.919304292473036	0.368	0.123	1,21E-22
POLH	6,71E-27	0.93015546203393	0.542	0.257	1,27E-22
HSD3BP4	8,92E-27	0.971907061430445	0.432	0.164	1,69E-22
ZNF551	9,81E-27	0.903445780221273	0.353	0.112	1,86E-22
KLF15	4,08E-26	0.80047690153099	0.718	0.531	7,74E-22
HHLA2	5,72E-26	0.971802546231797	0.326	0.1	1,08E-22
LINC00507	7,79E-28	0.785557635326932	0.255	0.06	1,48E-21
ZNF793	8,65E-26	0.89880826896951	0.516	0.243	1,64E-21
SLC7A14	9,15E-26	0.819401507931544	0.329	0.1	1,73E-21
SFTPB	9,32E-26	0.788337901008026	0.329	0.098	1,77E-21
CLEC19A	1,28E-25	0.771861272397535	0.279	0.072	2,42E-21
AP4S1	3,03E-25	0.85202283356419	0.482	0.211	5,75E-21
NCMAP	5,48E-25	0.872684037893	0.389	0.139	1,04E-21
ORAI2	7,57E-25	0.834582682592052	0.663	0.415	1,43E-20
CENPN	2,75E-24	0.882309088600398	0.45	0.185	5,22E-20
CARMN	3,83E-24	0.870217399008156	0.432	0.168	7,26E-20
CYP20A1	5,63E-24	0.833542192436186	0.689	0.462	1,07E-19
L2HGDH	7,60E-24	0.801431352336357	0.371	0.129	1,44E-19
ZNRF3-AS1	1,77E-23	0.923557172660886	0.403	0.151	3,35E-19
MCTP2	1,80E-24	0.829340068877557	0.392	0.147	3,40E-19
PASD1	2,01E-23	0.790589664663635	0.282	0.077	3,82E-19
MYLK3	4,95E-24	0.836555518922459	0.368	0.13	9,37E-19
GNRHR2	5,33E-23	0.755088700283046	0.303	0.088	1,01E-18
DNAAF4	5,57E-23	0.700377636924315	0.263	0.068	1,06E-18
ZNF582-AS	8,96E-23	0.748851932439061	0.303	0.09	1,70E-18
TRAPPC8	1,22E-24	0.887765583075661	0.395	0.151	2,32E-18
ZFP14	4,39E-22	0.842332543218981	0.442	0.185	8,31E-18
LOC40132	5,78E-22	0.788672708500995	0.266	0.072	1,09E-17
FAM120C	6,12E-22	0.872093359098622	0.316	0.099	1,16E-18
AP1S3	8,56E-22	0.887817774594039	0.395	0.153	1,62E-17
ALG1L9P	9,37E-22	0.811382827491282	0.334	0.109	1,78E-17
CHML	1,09E-21	0.772616368980753	0.311	0.097	2,07E-18
ADIPOQ-A!	1,33E-21	0.811483748938592	0.287	0.084	2,53E-17
ZNF716	1,36E-21	0.778580490192041	0.266	0.071	2,58E-17
HUNK	1,54E-21	0.732486175444519	0.268	0.072	2,91E-17
CEACAMP:	1,66E-21	0.784828128785793	0.411	0.16	3,14E-17
PNPLA3	2,20E-21	0.832883334179609	0.311	0.096	4,17E-17
ATCAY	3,23E-21	0.899145290639982	0.476	0.216	6,11E-17

CC2D2A	4,90E-21	0.848906904000239	0.347	0.123	9,29E-17
LECT2	9,54E-21	0.734596516919189	0.253	0.066	1,81E-16
HTATSF1P2	1,08E-20	0.777268306576333	0.361	0.13	2,05E-16
CRYBB2P1	1,51E-20	0.895398621465703	0.376	0.144	2,86E-16
SEC23IP	1,65E-20	0.794561731852733	0.4	0.161	3,13E-16
NPAP1	1,79E-20	0.734696023386232	0.289	0.085	3,40E-16
RASSF8	2,14E-21	0.759185967429265	0.345	0.12	4,06E-16
DPY19L2P2	6,02E-20	0.681526782110861	0.253	0.067	1,14E-15
LOC10050(	8,48E-20	0.924083703727614	0.45	0.207	1,61E-15
ZNF737	2,10E-19	0.777213833011766	0.432	0.185	3,97E-15
PIN4P1	2,18E-19	0.769533689717513	0.345	0.121	4,12E-15
LHFPL5	3,36E-19	0.870943056053013	0.411	0.174	6,37E-15
RAB3B	3,79E-19	0.759031017358869	0.468	0.209	7,19E-15
ZNF696	4,77E-19	0.784022450626306	0.337	0.118	9,03E-15
KCNJ5	8,13E-19	0.771950376033927	0.321	0.108	1,54E-14
ZNF738	9,50E-19	0.78472974453519	0.332	0.115	1,80E-14
TLCD2	1,02E-18	0.852792971190872	0.387	0.155	1,94E-14
NXPE3	1,23E-18	0.771322175370925	0.547	0.293	2,34E-14
POM121L1	2,00E-18	0.793394169419775	0.311	0.103	3,79E-14
LINC00476	2,16E-18	0.800131809165965	0.321	0.11	4,09E-14
SHROOM4	2,27E-18	0.862582779875716	0.371	0.146	4,31E-14
GJC1	3,59E-18	0.835321725356712	0.463	0.219	6,80E-14
STAT5A	4,75E-18	114.985.158.575.573	0.405	0.176	9,01E-14
DNAL1	5,05E-19	0.836253858642019	0.411	0.178	9,58E-14
NOS1	8,10E-18	0.797546333627313	0.374	0.146	1,53E-14
C1orf140	1,63E-17	0.775772897013044	0.292	0.094	3,09E-13
PTGER3	1,85E-17	0.726486441072178	0.253	0.072	3,50E-13
FKBP7	2,29E-17	0.695504297285842	0.261	0.077	4,35E-13
DUXA	2,46E-17	0.780413544198537	0.311	0.107	4,67E-13
TNFRSF9	3,00E-17	0.716255731549902	0.424	0.182	5,68E-13
IL10	4,69E-17	0.789020267747975	0.334	0.123	8,89E-13
CMBL	1,97E-16	0.739821289217193	0.284	0.091	3,74E-12
TSC22D1-A	3,18E-16	0.738627543364232	0.3	0.101	6,03E-12
PEX5L	3,21E-16	0.735800185775312	0.263	0.08	6,09E-12
METTL21A	4,00E-16	0.797164525915053	0.416	0.185	7,58E-12
METTL8	4,14E-16	0.818879657978882	0.511	0.274	7,84E-12
EPHA1-AS1	4,27E-16	0.736630030251264	0.339	0.125	8,09E-12
ZNF621	8,08E-16	0.750292607228296	0.566	0.345	1,53E-11
DIPK2B	1,33E-15	0.780845789959772	0.416	0.187	2,51E-11
BCYRN1	2,45E-15	0.732669692580772	0.408	0.18	4,64E-11
ZBTB8A	2,63E-15	0.716332122310366	0.397	0.171	4,99E-11
KLHL7-DT	2,69E-15	0.621322735997531	0.261	0.079	5,10E-11
HTR3B	3,12E-15	0.766858985751405	0.279	0.091	5,91E-11
TRIM4	4,77E-15	0.887484778270244	0.384	0.173	9,05E-11
DNMBP-AS	4,85E-15	0.708714289398153	0.276	0.09	9,20E-12
SLC9A3R2	5,15E-15	0.906672114311664	0.342	0.134	9,76E-12
FOXRED2	6,74E-15	0.689334729739156	0.276	0.09	1,28E-11
RBSN	6,81E-15	0.831046300487667	0.466	0.231	1,29E-10
URB1	8,87E-15	0.669235765782605	0.324	0.118	1,68E-10
LOC10013	8,89E-15	0.729651197431585	0.266	0.084	1,69E-10
CASP10	1,07E-14	0.845204253827628	0.392	0.177	2,04E-10
COG6	1,18E-15	0.77718759444954	0.334	0.13	2,25E-10
DBT	1,41E-15	0.768485874791824	0.476	0.247	2,67E-10
BSN-DT	2,67E-14	0.79904786130605	0.363	0.152	5,07E-10
LINC00578	2,69E-14	0.631506686704842	0.279	0.091	5,11E-10
LOC10050(	3,31E-14	0.873684003624902	0.421	0.197	6,27E-10

FXN	3,36E-15	0.773767184836384	0.403	0.182	6,37E-11
ZNF470	3,50E-14	0.664752240297362	0.274	0.089	6,63E-10
PPARA	3,54E-14	0.739475939254611	0.455	0.224	6,70E-10
ICA1L	4,85E-14	0.770247842936108	0.361	0.149	9,19E-10
LOC33980	5,10E-14	0.709422465150017	0.274	0.093	9,66E-10
LOC10012	7,51E-14	0.672877293798997	0.321	0.118	1,42E-09
AIPL1	7,87E-14	0.703883516816699	0.276	0.092	1,49E-09
CHURC1	2,20E-13	0.700109253792757	0.613	0.395	4,16E-09
CA5B	2,66E-13	0.69731755342157	0.35	0.142	5,04E-09
FZD3	2,91E-13	0.747224474729151	0.282	0.097	5,51E-09
FDPSP2	3,02E-13	0.80245085140172	0.353	0.147	5,73E-10
MYO10	5,59E-13	0.693315934084655	0.276	0.096	1,06E-08
ARGFX	5,67E-13	0.676503047335184	0.326	0.125	1,07E-08
LOC10012	6,52E-13	0.655045636330837	0.276	0.094	1,24E-08
SMA5	7,35E-13	0.73602330699439	0.326	0.126	1,39E-08
FKBP14	8,36E-14	0.778537827599767	0.411	0.197	1,58E-08
VSTM4	8,70E-13	0.746934042004928	0.326	0.13	1,65E-08
ELMOD1	9,14E-14	0.626990867551127	0.263	0.085	1,73E-08
PABPC1P2	1,07E-12	0.69353860557402	0.271	0.092	2,03E-10
PDP2	1,10E-12	0.7840574957761	0.345	0.141	2,08E-08
NLRP12	1,30E-12	0.737953490024754	0.421	0.199	2,46E-08
HHLA3	1,68E-12	0.688080987696555	0.279	0.099	3,18E-08
CABP4	1,99E-12	0.767106297462201	0.424	0.205	3,76E-08
SCN11A	2,84E-12	0.695943178763733	0.266	0.09	5,38E-08
TMEM213	2,84E-12	0.764547507126529	0.387	0.17	5,39E-09
ZNF714	3,10E-12	0.725209359635565	0.376	0.166	5,88E-08
GTF2H5	3,52E-12	0.761089260167232	0.447	0.231	6,67E-08
RAMP2-AS	4,04E-12	0.727014693175976	0.358	0.154	7,66E-08
ZNF114	4,48E-12	0.820168541233684	0.342	0.144	8,50E-08
CDHR3	5,28E-12	0.59022703391676	0.263	0.088	1,00E-07
FBXL18	7,62E-13	0.75506387551281	0.363	0.162	1,44E-07
SEC14L4	8,40E-12	0.686715456808329	0.274	0.095	1,59E-07
LRRC2	8,40E-12	0.694241506398993	0.413	0.194	1,59E-07
SLC15A2	9,22E-12	0.719203814414385	0.313	0.12	1,75E-07
BHMT2	2,66E-11	0.765311339328173	0.408	0.198	5,05E-07
SLC35E2A	2,96E-11	0.60804537811928	0.271	0.094	5,61E-07
GGT6	4,13E-12	0.696614199805552	0.276	0.1	7,82E-07
CYP27C1	4,56E-11	0.703492555190884	0.326	0.131	8,65E-07
QPCTL	5,59E-11	0.722515257328371	0.255	0.088	1,06E-06
SCML4	1,35E-10	0.710206217399648	0.447	0.235	2,56E-06
VSIG1	1,64E-10	0.661691118791096	0.261	0.09	3,11E-06
FLJ42627	1,75E-10	0.782763786340978	0.355	0.158	3,32E-06
ZNF529	1,76E-10	0.723385403044381	0.353	0.156	3,34E-06
GRM6	2,95E-10	0.777874366635287	0.345	0.148	5,59E-06
KCNK6	3,20E-10	0.750524631825391	0.353	0.156	6,07E-06
ITIH5	3,22E-10	0.662643062946198	0.324	0.131	6,11E-06
SLC4A4	4,54E-10	0.68448865956866	0.318	0.131	8,61E-06
IRGQ	4,95E-10	0.746318997364689	0.371	0.174	9,38E-06
TYW3	5,22E-10	0.74571277682115	0.505	0.307	9,88E-06
GK5	5,45E-10	0.715582204018966	0.487	0.277	1,03E-05
KREMEN1	8,90E-10	0.711311474754605	0.503	0.294	1,69E-05
EMX2OS	1,51E-09	0.701652807934326	0.276	0.103	2,86E-05
SGSM1	1,75E-10	0.690565701044089	0.334	0.141	3,32E-05
LINC00574	2,45E-09	0.678819914374232	0.268	0.1	4,64E-05
HAUS2	3,76E-09	0.747489204193943	0.426	0.22	7,12E-05
JRK	5,90E-10	0.614229377968822	0.261	0.094	1,12E-04

NMNAT1	6,29E-09	0.76243747797441	0.4	0.202	1,19E-05
KCNK3	6,66E-09	0.630318050681234	0.263	0.097	1,26E-04
ZNF818P	6,87E-09	0.702870281281385	0.284	0.111	1,30E-04
PDE6A	7,13E-09	0.802815699861412	0.495	0.293	1,35E-04
FBLIM1	9,02E-09	0.718988853193526	0.497	0.29	1,71E-04
PALM2	9,05E-09	0.656367936335396	0.297	0.121	1,71E-04
XPNPEP3	9,10E-09	0.690957735757366	0.395	0.193	1,72E-04
SETMAR	1,05E-08	0.673351868066251	0.276	0.105	2,00E-04
TMC7	1,13E-08	0.697075806834035	0.287	0.114	2,14E-04
NUBPL	1,26E-08	0.751587732164357	0.413	0.21	2,38E-04
ZNF665	1,49E-09	0.738990756764208	0.363	0.168	2,83E-04
TSTD3	1,62E-08	0.604108089034682	0.276	0.105	3,08E-05
FAM106A	1,72E-08	0.688362795896536	0.318	0.136	3,25E-05
ZNF483	2,02E-08	0.687876969699928	0.308	0.13	3,82E-04
GLIPR1L2	2,49E-08	0.650965881751726	0.392	0.193	4,72E-04
EFHC1	3,00E-08	0.623404618653579	0.313	0.131	5,69E-04
KIAA1549	4,70E-08	0.606875421550533	0.266	0.1	8,90E-05
CHST6	7,61E-08	0.685249230858076	0.279	0.111	1,44E-04
THAP6	8,05E-08	0.604305736811101	0.274	0.106	1,53E-03
ALG1	8,58E-08	0.629935073840374	0.303	0.125	1,63E-04
JHY	9,22E-08	0.731492190987382	0.282	0.111	1,75E-03
THEM4	9,55E-08	0.723051967639906	0.403	0.209	1,81E-03
AASS	1,15E-08	0.593716819066371	0.279	0.11	2,18E-03
PXMP4	1,18E-07	0.707453963088973	0.347	0.162	2,23E-03
KBTBD6	1,28E-07	0.639894905941925	0.274	0.106	2,43E-03
MTRNR2L4	1,33E-07	0.74878954247697	0.266	0.103	2,52E-03
FBXL19-AS	1,65E-07	0.646691996648071	0.297	0.123	3,12E-03
GPR155	2,22E-07	0.661757525808228	0.532	0.325	4,21E-04
TRAF3IP2	2,46E-07	0.722718009251269	0.292	0.122	4,66E-03
ZNF557	3,57E-07	0.63933783868573	0.308	0.129	6,77E-03
KRT18	4,28E-07	0.667932671081088	0.253	0.096	8,11E-03
TMEM192	5,27E-08	0.655675068999472	0.426	0.228	9,99E-03
TOR1AIP2	5,96E-07	0.710356013081809	0.426	0.242	1,13E-02
GOSR1	7,11E-07	0.676565073908662	0.387	0.196	1,35E-02
MARVELD3	8,59E-07	0.645867945456955	0.287	0.117	1,63E-02
TFDP2	1,00E-06	0.71229475179571	0.426	0.232	1,90E-02
COA1	1,25E-06	0.627921142834971	0.392	0.201	2,37E-02
FLVCR1	1,28E-06	0.614021269203453	0.313	0.136	2,42E-02
LINC00311	1,49E-06	0.701365852799205	0.363	0.175	2,83E-03
MTG2	2,11E-06	0.687489282331208	0.308	0.137	4,00E-02
OPA3	2,27E-06	0.651894666121737	0.497	0.301	4,30E-02
LMOD3	2,51E-06	0.692890560020285	0.263	0.104	4,76E-02
LAX1	3,21E-06	0.563869074798507	0.268	0.106	6,07E-02
C21orf62	3,55E-06	0.633961898451423	0.274	0.111	6,73E-02
YAF2	4,88E-06	0.70462089095546	0.426	0.242	9,25E-02
ACP7	6,21E-06	0.547317629579931	0.263	0.104	1,18E-01
ABHD11	7,45E-06	0.685120418692305	0.287	0.122	1,41E-01
IBA57	7,93E-06	0.606268188182105	0.289	0.123	1,50E-01
PRDM7	8,74E-06	0.721098645233517	0.289	0.125	1,66E-01
MDM4	9,45E-07	0.496103589721339	0.739	0.627	1,79E-01
DSG2	1,25E-05	0.671856072954352	0.292	0.126	2,37E-01
VPS53	1,42E-05	0.675675408633949	0.458	0.267	2,70E-01
NLN	1,47E-06	0.619525518942023	0.287	0.123	2,79E-01
GFOD2	1,50E-06	0.638707087100604	0.334	0.157	2,85E-01
PLA2G12A	1,52E-05	0.636450522976339	0.342	0.165	2,88E-01
KIF1C	1,57E-05	0.642785069875912	0.384	0.202	2,98E-01

IVD	1,99E-05	0.673903743873209	0.461	0.274	3,77E-02
METTL2B	2,07E-05	0.675864141234343	0.303	0.135	3,93E-01
TTTY15	2,30E-05	0.55743545701776	0.303	0.132	4,36E-02
ERCC4	2,32E-05	0.633404940932953	0.358	0.177	4,40E-01
WDR17	3,13E-05	0.568901145139795	0.253	0.1	5,93E-01
INF2	4,73E-06	0.64246566045831	0.297	0.135	8,95E-01
SPATA5	5,43E-05	0.604876089925245	0.253	0.101	1,03E+00
MMRN2	7,65E-05	0.446916817379782	0.297	0.124	1,45E+00
GBP4	8,55E-05	0.681048840901856	0.461	0.283	1,62E-01
PRKXP1	9,35E-05	0.57671132358728	0.274	0.116	1,77E+00
ZNF320	1,24E-04	0.569671537157266	0.274	0.115	2,34E+00
LINC02210	1,99E-04	0.660730224021879	0.268	0.114	3,77E+00
RINL	2,04E-05	0.625355967298984	0.297	0.132	3,86E+00
DHTKD1	2,61E-05	0.617690633299038	0.368	0.188	4,94E+00
SPIB	2,88E-04	0.610589905644992	0.326	0.155	5,45E+00
ZHX3	5,24E-06	0.660952267542451	0.363	0.188	9,92E+00
COQ10B	6,72E-04	0.721299233108788	0.439	0.257	1,27E+01
KIAA1143	6,76E-04	0.626054826288401	0.376	0.203	1,28E+01
ZNF865	6,98E-04	0.578057205295884	0.3	0.136	1,32E+01
PATJ	8,03E-04	0.639465708887709	0.408	0.225	1,52E+01
GPR82	8,66E-04	0.547465848414984	0.339	0.165	1,64E+01
ARMC9	9,23E-04	0.65310299452131	0.297	0.138	1,75E+01
SWAP70	9,31E-04	0.682104400887845	0.411	0.236	1,76E+01
MR1	1,12E-03	0.636264841224294	0.366	0.194	2,12E+01
LINC00672	1,48E-03	0.531145588104432	0.261	0.108	2,81E+00
CACNG8	1,54E-03	0.600669185758189	0.297	0.137	2,91E+01
C9orf64	1,61E-03	0.622145116562401	0.253	0.106	3,04E+01
MIGA1	3,15E-03	0.654000268277153	0.379	0.209	5,97E+01
EMP2	3,64E-03	0.582799605210606	0.418	0.244	6,90E+01
ZMYM5	4,91E-03	0.667909827156758	0.387	0.215	9,30E+01
RAB11FIP4	5,54E-03	0.590916627526465	0.379	0.202	1,05E+02
CXorf38	6,54E-05	0.583522303349814	0.361	0.19	1,24E+02
PDE4C	1,23E-02	0.579097666827053	0.279	0.126	2,32E+02
IPO9	1,24E-02	0.635858140061684	0.466	0.299	2,35E+02
LYRM7	1,43E-03	0.608168426962049	0.453	0.283	2,71E+02
IKZF3	1,51E-03	0.428872976448188	0.605	0.41	2,86E+02
SLC9A7	1,55E-02	0.577146378147112	0.271	0.123	2,93E+02
GSTM3	1,61E-02	0.597983452923505	0.3	0.144	3,05E+01
HCG11	1,63E-02	0.65290917964347	0.358	0.189	3,09E+02
C17orf75	2,19E-02	0.531083413842257	0.271	0.121	4,15E+02
SIGLEC10	3,80E-02	0.543923345016022	0.347	0.181	7,21E+02
LPP	4,14E-02	0.494399320549752	0.584	0.442	7,84E+02
CPM	5,56E-02	0.527895798055991	0.366	0.198	1,05E+03
ADGRE2	6,90E-02	0.561341505386809	0.261	0.116	1,31E+03
CLSPN	7,21E-02	0.535390591726023	0.284	0.131	1,37E+03
ST3GAL6	9,15E-02	0.606987137058828	0.268	0.123	1,73E+03
DNASE1	1,07E-01	0.552011968595536	0.271	0.124	2,04E+03
KLRD1	1,42E-01	0.470190072875177	0.413	0.235	2,69E+03
ARMC10	2,02E-03	0.58013864273666	0.258	0.119	3,83E+03
CLUAP1	3,18E-01	0.547621806806469	0.305	0.153	6,03E+03
LRRC57	3,35E-01	0.495957066919354	0.266	0.123	6,36E+03
BLZF1	6,21E-01	0.57734099020688	0.313	0.162	1,18E+04
PGPEP1	6,51E-01	0.570604728018306	0.411	0.247	1,23E+04
FLT1	6,90E-01	0.547375405993652	0.287	0.139	1,31E+04
CPSF2	6,92E-01	0.554482166989753	0.287	0.144	1,31E+04
TPM3P9	8,19E-02	0.480490794210151	0.258	0.118	1,55E+04

C3orf62	1,06E+00	0.612543766591531	0.274	0.133	2,01E+04
PNPT1	1,10E+00	0.684916175900136	0.368	0.214	2,08E+04
WDR92	2,02E+00	0.579583431136356	0.303	0.155	3,84E+03
LMLN	2,31E+00	0.525892079398748	0.253	0.118	4,37E+04
PPARD	4,05E+00	0.616604831007741	0.276	0.139	7,68E+04
NICN1	4,24E+00	0.599885915052063	0.287	0.145	8,04E+04
DCAF10	5,29E+00	0.566232083212823	0.405	0.247	1,00E+05
IRF1	6,14E+00	0.474240447118359	0.553	0.391	1,16E+05
DFFA	6,19E+00	0.651393026588537	0.308	0.164	1,17E+05
TET2	6,48E+00	0.59543966747027	0.35	0.202	1,23E+05
XIAP	6,63E-01	0.544430180054543	0.479	0.336	1,26E+05
PGM2L1	6,90E+00	0.562785638088146	0.442	0.285	1,31E+05
FBXL20	8,44E+00	0.559057147905878	0.371	0.216	1,60E+04
DIXDC1	8,90E+00	0.564639555890337	0.263	0.127	1,69E+05
TTN	1,06E+01	0.502855649571268	0.303	0.151	2,01E+05
NFAM1	1,98E+01	0.572688460263052	0.318	0.173	3,76E+04
EXOSC2	2,18E+01	0.583278086822332	0.279	0.142	4,13E+05
LRRC58	2,68E+01	0.586622603746965	0.376	0.228	5,08E+05
MAPK13	2,79E+01	0.507355394565969	0.266	0.132	5,29E+05
RNF14	2,87E+01	0.563835486462663	0.305	0.163	5,44E+05
TPMT	2,92E+01	0.534197553710218	0.263	0.131	5,53E+05
LOC44108	2,96E+01	0.560557760049119	0.258	0.127	5,62E+05
DIS3	3,84E+01	0.559056519824421	0.297	0.16	7,27E+04
SMYD4	4,82E+01	0.538465093081929	0.287	0.149	9,13E+05
DRAM1	5,13E+01	0.597669144580083	0.279	0.147	9,72E+05
AARS2	5,82E+01	0.563624804320224	0.253	0.124	1,10E+05
SLC31A1	9,01E+01	0.606323440257191	0.318	0.182	1,71E+06
TMEM181	1,16E+02	0.51911965985994	0.437	0.289	2,20E+06
TIGAR	1,27E+02	0.46013337379087	0.297	0.159	2,40E+06
SLC35A3	1,32E+02	0.598545651815922	0.347	0.203	2,50E+06
TERF1	1,34E+02	0.582730317769324	0.329	0.187	2,54E+06
LRPAP1	1,92E+02	0.535746680002519	0.484	0.344	3,64E+06
CCDC142	2,06E+02	0.542447428110614	0.292	0.156	3,91E+06
KIF1B	2,14E+02	0.574705735333586	0.329	0.19	4,05E+06
GALNT6	2,30E+02	0.512095819940022	0.295	0.159	4,35E+06
SHANK2-A	3,11E+00	0.471161438250414	0.271	0.139	5,89E+06
ZNF542P	3,51E+02	0.513664238518859	0.261	0.133	6,65E+06
TRIM65	3,85E+02	0.519439664167958	0.284	0.152	7,29E+06
EMC1	4,19E+02	0.586745861664797	0.332	0.2	7,95E+06
CPPED1	4,63E+02	0.542393046810228	0.313	0.181	8,78E+06
TRPM7	7,85E+02	0.500505697361585	0.392	0.247	1,49E+07
LINC00294	9,77E+02	0.515136304360359	0.326	0.186	1,85E+07
LOC10019	1,09E+03	0.515663857677007	0.516	0.404	2,06E+07
ABI2	1,22E+03	0.516499542161769	0.329	0.192	2,31E+07
RRP7A	1,23E+03	0.530735357189726	0.371	0.231	2,34E+07
SERPINE1	1,32E+03	0.436617191300069	0.261	0.134	2,51E+07
ASPH	1,59E+03	0.470058489963554	0.292	0.162	3,02E+07
DCAF11	1,93E+02	0.475302600946709	0.279	0.15	3,65E+06
MAVS	2,57E+02	0.466867483217486	0.453	0.318	4,88E+07
MCTS1	3,67E+03	0.528547272863762	0.311	0.18	6,95E+07
GTPBP10	4,10E+03	0.471034605651409	0.268	0.144	7,77E+07
PHAX	4,72E+03	0.528520848235827	0.345	0.211	8,94E+07
C5orf24	5,65E+03	0.536857774222924	0.321	0.192	1,07E+08
GUSBP3	7,61E+03	0.48164285597622	0.363	0.227	1,44E+08
LOC72968	1,02E+04	0.603777846892139	0.261	0.143	1,93E+08
RNF141	1,27E+04	0.537124763566423	0.279	0.16	2,41E+06

ABL2	1,64E+04	0.562489795866032	0.337	0.215	3,10E+08
TTPAL	1,66E+04	0.442637558620466	0.313	0.182	3,15E+08
MINDY2	2,05E+04	0.407928863092209	0.305	0.174	3,88E+07
UTP14C	2,71E+04	0.444816258352373	0.263	0.143	5,13E+08
UGGT1	2,84E+03	0.466608713559534	0.361	0.234	5,38E+08
SPN	2,88E+04	0.461831849020373	0.463	0.33	5,46E+08
UBXN7	3,33E+03	0.555725019202846	0.292	0.173	6,30E+06
HSH2D	3,47E+04	0.371687506960717	0.274	0.148	6,58E+08
TESPA1	3,53E+04	0.373684508173975	0.276	0.152	6,68E+08
ZYG11B	5,39E+04	0.549726246072369	0.345	0.223	1,02E+08
DCUN1D5	6,28E+04	0.420079327727758	0.289	0.168	1,19E+09
GNL3L	6,44E+04	0.499161732960528	0.329	0.203	1,22E+09
TNFAIP8L1	7,73E+04	0.42304788262377	0.258	0.14	1,46E+09
CORO2A	7,94E+04	0.441331624437811	0.295	0.171	1,50E+09
GRIPAP1	2,27E+05	0.510766656756113	0.326	0.209	4,29E+08
SRCAP	2,37E+05	0.416199287723314	0.258	0.144	4,50E+09
ZNF264	3,97E+05	0.471697169906815	0.313	0.196	7,52E+08
CWF19L1	4,47E+05	0.487411694003674	0.318	0.203	8,47E+09
SLAMF6	6,50E+05	0.442452182621959	0.35	0.225	0.000123219994876443
PTPN2	7,75E+05	0.399626474975991	0.397	0.275	0.000146811480818188
ARSA	1,06E+06	0.433478732626678	0.266	0.157	0.000201152202643902
RHOBTB3	1,24E+05	0.511637787183139	0.266	0.157	0.000234883431945944
ATXN3	1,25E+06	0.467308874622507	0.321	0.206	0.000236428540392099
NOL9	1,40E+06	0.469526251704042	0.284	0.174	0.000264873323417784
ENAH	1,62E+06	0.533015773659794	0.263	0.159	0.000306728972625398
IL6R	2,07E+04	0.408778685620485	0.324	0.207	0.000392164800000276
FCF1	2,14E+06	0.424255734785789	0.276	0.167	0.000405526928303533
AAK1	2,38E+06	0.300975260800666	0.626	0.541	0.000450828452666135
TM7SF3	2,77E+04	0.443701206017897	0.339	0.227	0.000524619385776908
CTBP2	3,81E+06	0.457868807022098	0.303	0.197	0.000722452008346408
UTP23	3,94E+06	0.57031590687983	0.295	0.19	0.000745735462361341
RSL1D1	5,05E+06	0.426440087293728	0.455	0.346	0.000957095539504727
NCBP1	5,17E+06	0.399360389494026	0.255	0.152	0.00097957585412094
HOOK3	5,88E+06	0.472869032055023	0.432	0.328	0.00111411962137463
NCOA7	1,33E+07	0.429523040931008	0.334	0.226	0.002524699342265
CLN8	1,44E+07	0.429996723467915	0.258	0.158	0.00273810799544308
WAC-AS1	1,46E+07	0.447735151708101	0.474	0.394	0.00276130368597161
SMAD5	2,12E+07	0.373324941346789	0.271	0.169	0.0040157765914949
GATAD1	2,29E+07	0.468451636366661	0.376	0.274	0.00433519029293032
CD28	2,64E+06	0.302972549235903	0.268	0.162	0.00500633676607747
NMT2	2,88E+07	0.422848858258095	0.308	0.203	0.00546184671398909
GNB5	3,62E+07	0.375300990930286	0.321	0.215	0.00685395025184682
NUDC	3,67E+06	0.472553864513524	0.374	0.281	0.0069551204886762
CEP135	4,26E+07	0.406268785430741	0.263	0.162	0.00806932865446266
CCBE1	5,88E+07	0.470935688663579	0.3	0.204	0.0111473787702369
COX19	5,90E+07	0.420045080639253	0.271	0.173	0.0111723245736086
PHACTR4	6,99E+07	0.403100267576201	0.376	0.278	0.0132506666983279
GCLM	1,43E+08	0.39125516146163	0.263	0.17	0.0271358466322528
TCAF1	1,45E+08	0.388054450425771	0.268	0.175	0.0274903392977233
GAS7	1,87E+08	0.366003502691548	0.297	0.2	0.0354086244871439
SLC16A7	2,12E+08	0.317209345451154	0.292	0.192	0.0402109138575151
NSL1	2,30E+08	0.417467023171022	0.295	0.201	0.0435263333551674
TRMT10B	2,75E+08	0.39837061695004	0.289	0.196	0.0520615877805259
DSTYK	3,36E+07	0.348932946399394	0.274	0.182	0.0636037609725817
SMN1	3,91E+08	0.398231101075266	0.263	0.174	0.0741727350782295
GOLGA2	4,26E+08	0.358325813572727	0.261	0.171	0.0807212808765294

RBBP4	4,97E+08	0.377176592081814	0.345	0.252	0.0941684576730716
PIK3C2A	6,46E+08	0.403553124705074	0.308	0.219	0.122352337051875
CAV2	1,08E+09	0.306094629894137	0.263	0.172	0.204369994888157
PCYOX1	1,26E+09	0.333354542087298	0.292	0.202	0.238650817311013
NLRP6	2,06E+08	0.465007195080903	0.342	0.271	0.389786818584108
MTHFR	3,62E+09	0.307518068071031	0.284	0.201	0.686852640702182
SPTLC2	4,48E+09	0.417100260954597	0.276	0.202	0.848676781610469
SLC12A6	5,70E+09	0.296091584600486	0.255	0.175	1
PHC3	5,74E+09	0.34063295528915	0.339	0.256	1
TMEM167,	7,61E+09	0.337242718538655	0.263	0.186	1
RNF125	9,79E+09	0.266471463017639	0.318	0.233	1
ELK4	0.0001315	0.351043519584602	0.313	0.237	1
RAB11FIP1	0.0002027	0.274796391062627	0.313	0.237	1
ANKRD36E	0.0003134	0.258196064907301	0.289	0.215	1
ILF3-DT	0.0004154	0.276719439028501	0.258	0.188	1
MAGT1	0.0007604	0.299610645069126	0.329	0.262	1
MYO5A	0.0009080	0.296450231252677	0.326	0.261	1
DENR	0.0010537	0.398837291566998	0.282	0.219	1
KIAA0355	0.0011378	0.291543748684884	0.263	0.201	1
HIP1	0.0014779	0.28291432476213	0.261	0.198	1
CREB1	0.0014941	0.345316177820904	0.261	0.2	1
DIP2A	0.0016253	0.282258602319494	0.329	0.265	1
NFATC2IP	0.0023355	0.307439438419462	0.334	0.279	1
CYCS	0.0035924	0.253710744725248	0.405	0.36	1
CBX5	0.0060389	0.261125762094704	0.3	0.247	1
RABGAP1L	0.0067619	0.257764363820113	0.303	0.248	1

Cluster 3 marker genes

gene	p_val	avg_logFC	pct.1	pct.2	p_val_adj
FGFBP2	2,04E-254	2,339888827	0,573	0,021	3,86E-250
GZMB	1,56E-212	2,337431106	0,681	0,054	2,96E-208
ADGRG1	1,72E-167	1,725867998	0,559	0,045	3,26E-163
PRF1	3,75E-155	2,03273578	0,761	0,116	7,11E-151
KLRF1	1,49E-146	1,732286078	0,484	0,036	2,83E-142
NKG7	6,75E-140	2,035173244	0,939	0,265	1,28E-135
SPON2	4,85E-127	1,472361056	0,315	0,013	9,20E-123
GNLY	1,28E-105	2,68095321	0,822	0,269	2,42E-101
HOPX	5,65E-83	1,286375967	0,554	0,101	1,07E-78
CX3CR1	7,46E-76	1,348291293	0,394	0,055	1,41E-71
CTSW	2,06E-73	1,32019375	0,784	0,269	3,91E-69
GZMH	1,42E-71	1,404893927	0,526	0,105	2,69E-67
GZMA	2,59E-60	1,022238925	0,831	0,304	4,90E-56
CCL5	6,17E-59	1,061654328	0,93	0,476	1,17E-54
KLRD1	6,72E-56	1,198419977	0,667	0,227	1,27E-51
IL2RB	3,06E-54	1,386337912	0,638	0,209	5,80E-50
TXK	9,26E-54	1,017248719	0,286	0,039	1,75E-49
CST7	5,40E-52	1,169144613	0,648	0,217	1,02E-47
FCRL6	6,36E-49	1,12580042	0,376	0,076	1,21E-44
CD247	6,35E-48	1,036951587	0,545	0,156	1,20E-43
MYBL1	7,86E-44	0,841804516	0,385	0,083	1,49E-39
LGALS13	2,62E-41	0,959973481	0,399	0,098	4,97E-37
IFITM1	3,19E-41	0,85684759	0,906	0,552	6,05E-37
KLRB1	1,01E-40	1,139597671	0,46	0,131	1,91E-36
RUNX3	4,99E-39	0,964992317	0,732	0,346	9,46E-35
HLA-C	8,95E-38	0,719932321	0,967	0,856	1,70E-33
BIN2	1,69E-37	0,913080971	0,606	0,232	3,21E-33
HLA-B	2,38E-37	0,543836067	0,991	0,913	4,51E-33
FCGR3A	2,96E-36	0,89648294	0,333	0,077	5,61E-32
CD7	9,71E-35	1,146067415	0,502	0,172	1,84E-30
TSPAN32	8,93E-33	0,787038395	0,319	0,077	1,69E-28
EFHD2	1,25E-32	0,95475936	0,606	0,271	2,36E-28
ITGAL	8,07E-32	0,843965662	0,69	0,343	1,53E-27
TGFBR3	6,73E-31	1,083848553	0,479	0,18	1,27E-26
PYHIN1	1,19E-26	0,738854428	0,282	0,072	2,26E-22
ARL4C	2,18E-26	0,781591759	0,7	0,4	4,13E-22
ZAP70	7,93E-26	0,759895999	0,423	0,148	1,50E-21
B2M	2,28E-25	0,485480241	1	0,934	4,32E-21
SAMD3	9,44E-24	0,653703449	0,357	0,114	1,79E-19
HCST	1,02E-23	0,687357418	0,728	0,437	1,93E-19
ITGB2	1,21E-23	0,735332622	0,714	0,426	2,30E-19
MATK	1,27E-23	0,570760504	0,268	0,071	2,41E-19
APMAP	2,95E-23	0,841237822	0,427	0,173	5,59E-19
PLEK	3,67E-23	0,800401368	0,488	0,216	6,96E-19
CHST12	4,11E-23	0,659835917	0,296	0,089	7,80E-19
LPCAT1	1,33E-22	0,64310333	0,263	0,073	2,52E-18
ABHD17A	1,40E-22	0,705425561	0,502	0,229	2,66E-18
SLC9A3R1	3,00E-22	0,738783839	0,469	0,201	5,69E-18
SLAMF7	3,10E-22	0,734427142	0,366	0,131	5,87E-18
GOLGA8S	3,72E-22	0,854867269	0,319	0,107	7,05E-18
GNAQ	5,33E-22	0,878160872	0,291	0,09	1,01E-17
FGR	7,46E-22	0,763627131	0,39	0,149	1,41E-17
HLA-F	2,77E-21	0,580934488	0,685	0,388	5,25E-17

LCP1	3,59E-21	0,483502304	0,948	0,687	6,80E-17
PRKCH	8,93E-21	0,636070801	0,507	0,233	1,69E-16
RARRES3	1,61E-20	0,698840582	0,488	0,224	3,04E-16
PTPRC	1,81E-20	0,533472057	0,869	0,63	3,42E-16
USP28	4,29E-20	0,626864883	0,254	0,074	8,12E-16
FLNA	6,02E-20	0,58651824	0,751	0,507	1,14E-15
SYNE1	6,73E-20	0,759563899	0,516	0,263	1,28E-15
BTN3A2	9,60E-20	0,548918509	0,521	0,243	1,82E-15
HLA-E	4,81E-19	0,384060516	0,972	0,845	9,11E-15
SUN2	1,89E-18	0,615595032	0,667	0,414	3,57E-14
GNPTAB	3,92E-18	0,715468446	0,474	0,232	7,44E-14
PTPN4	3,99E-18	0,694083467	0,319	0,117	7,57E-14
FYN	4,14E-18	0,575370436	0,653	0,369	7,85E-14
ITGAM	4,20E-18	0,655908124	0,315	0,115	7,95E-14
SYTL1	1,27E-17	0,55880855	0,371	0,146	2,41E-13
LITAF	4,01E-17	0,610646219	0,723	0,495	7,59E-13
ADD3	7,56E-17	0,564724291	0,601	0,337	1,43E-12
HLA-A	1,21E-16	0,417467173	0,977	0,867	2,30E-12
RASSF1	2,39E-16	0,58510727	0,296	0,111	4,53E-12
ITGB7	4,65E-16	0,532586178	0,319	0,122	8,81E-12
APOBEC3G	4,95E-16	0,578104452	0,441	0,203	9,37E-12
TXNIP	1,31E-15	0,644921425	0,69	0,457	2,48E-11
SYNE2	2,51E-15	0,613946116	0,615	0,37	4,75E-11
CYFIP2	9,08E-15	0,593586133	0,535	0,286	1,72E-10
AKNA	9,79E-15	0,554637403	0,582	0,351	1,86E-10
BTN3A1	1,10E-14	0,53707457	0,427	0,202	2,08E-10
AOAH	3,55E-14	0,595174455	0,282	0,111	6,73E-10
RAP1B	6,97E-14	0,57366378	0,559	0,348	1,32E-09
MAPK1	8,87E-14	0,619466964	0,465	0,256	1,68E-09
CCND3	1,09E-13	0,502008912	0,427	0,218	2,07E-09
RAC2	1,98E-13	0,406831046	0,751	0,525	3,76E-09
ADAM8	2,52E-13	0,520811448	0,338	0,151	4,77E-09
TPST2	2,71E-13	0,620407816	0,258	0,101	5,14E-09
ETS1	3,66E-13	0,415490433	0,714	0,45	6,93E-09
GBP5	4,80E-13	0,492391003	0,399	0,189	9,10E-09
MYL12A	5,48E-13	0,425441773	0,775	0,602	1,04E-08
MBL2	9,76E-13	0,609073247	0,38	0,192	1,85E-08
JAK1	9,97E-13	0,52227604	0,62	0,411	1,89E-08
ARHGEF3	1,33E-12	0,50739519	0,277	0,112	2,52E-08
KLRG1	1,43E-12	0,589176657	0,347	0,165	2,72E-08
PRKACB	1,47E-12	0,538787869	0,399	0,203	2,78E-08
GNG2	1,69E-12	0,551416432	0,38	0,188	3,20E-08
STAT4	2,86E-12	0,442217216	0,324	0,142	5,42E-08
LCK	3,68E-12	0,375847958	0,516	0,273	6,97E-08
SRGN	4,52E-12	0,339678946	0,901	0,715	8,57E-08
C12orf75	5,07E-12	0,424812009	0,277	0,114	9,61E-08
IL2RG	5,74E-12	0,363790753	0,826	0,604	1,09E-07
MYO1F	5,91E-12	0,565946808	0,418	0,22	1,12E-07
RAB27A	6,14E-12	0,494395406	0,498	0,294	1,16E-07
LSP1	9,67E-12	0,389794317	0,592	0,358	1,83E-07
SORL1	9,74E-12	0,550396975	0,54	0,334	1,85E-07
SH2D2A	1,25E-11	0,367647333	0,272	0,112	2,36E-07
NLRC5	1,28E-11	0,531114317	0,385	0,193	2,42E-07
STK38	1,79E-11	0,593626178	0,366	0,188	3,39E-07
DENND2D	2,14E-11	0,488501219	0,254	0,103	4,05E-07
PXN	2,53E-11	0,545476241	0,315	0,15	4,79E-07

IQGAP2	3,90E-11	0,429511107	0,418	0,221	7,39E-07
PARP8	3,94E-11	0,417043078	0,413	0,211	7,47E-07
PPP2R5C	6,54E-11	0,557625945	0,46	0,281	1,24E-06
SSBP3	6,77E-11	0,481025677	0,291	0,134	1,28E-06
KLF13	9,10E-11	0,580907753	0,469	0,286	1,72E-06
PIP4K2A	1,02E-10	0,468888688	0,521	0,312	1,93E-06
EVL	1,39E-10	0,434346446	0,563	0,362	2,64E-06
ADGRE5	3,08E-10	0,414473425	0,427	0,243	5,83E-06
ATM	4,32E-10	0,413975281	0,596	0,406	8,19E-06
RNF166	4,91E-10	0,48694459	0,352	0,181	9,31E-06
GLCCI1	5,19E-10	0,430342296	0,329	0,164	9,84E-06
INPP4A	5,99E-10	0,521805891	0,263	0,121	1,14E-05
CDC42SE2	6,54E-10	0,418973284	0,535	0,338	1,24E-05
RNF213	7,69E-10	0,300422924	0,826	0,666	1,46E-05
MLLT6	1,09E-09	0,40996773	0,601	0,419	2,06E-05
IKZF1	1,14E-09	0,368978713	0,507	0,299	2,16E-05
HELZ	1,30E-09	0,463907726	0,394	0,226	2,46E-05
TARP	1,98E-09	0,43488848	0,343	0,179	3,75E-05
PTPN12	4,08E-09	0,559459979	0,333	0,188	7,73E-05
UHMK1	4,27E-09	0,527795101	0,418	0,247	8,09E-05
UBB	6,26E-09	0,359292145	0,737	0,582	0,000118596
SELPLG	1,26E-08	0,491976384	0,371	0,214	0,000238199
CORO1A	1,27E-08	0,471762254	0,319	0,168	0,000240619
TBC1D10C	1,35E-08	0,443675804	0,305	0,156	0,000255626
CALM1	1,53E-08	0,259387273	0,911	0,839	0,000289
ARAP2	1,62E-08	0,438049863	0,282	0,14	0,000306186
AES	1,89E-08	0,432110214	0,685	0,546	0,000358138
SPATA13	2,24E-08	0,364114818	0,338	0,183	0,000425194
NFATC2	2,73E-08	0,382693272	0,404	0,236	0,000516741
ZBTB44	3,01E-08	0,490375716	0,329	0,185	0,000570538
PTP4A2	3,13E-08	0,342951529	0,728	0,61	0,000592908
GSE1	3,46E-08	0,449013279	0,277	0,144	0,00065638
EIF4EBP2	3,56E-08	0,491697087	0,441	0,277	0,000674865
NFATC3	3,92E-08	0,401883387	0,357	0,205	0,00074366
CCDC69	4,70E-08	0,465903711	0,408	0,252	0,000889973
RIPOR2	5,16E-08	0,411280301	0,479	0,297	0,000976962
PTPRCAP	5,66E-08	0,355439072	0,662	0,474	0,001072786
DHRS7	6,61E-08	0,401465007	0,324	0,181	0,00125268
STK17A	8,74E-08	0,443268057	0,362	0,21	0,001656055
CNOT6L	9,04E-08	0,452549146	0,413	0,256	0,001713495
OGT	1,03E-07	0,369900008	0,502	0,335	0,001949384
PPP1CA	1,09E-07	0,522579836	0,263	0,138	0,00207328
RNF125	1,25E-07	0,356539915	0,39	0,232	0,002363168
ERBIN	1,55E-07	0,487603228	0,352	0,216	0,002946395
CCL4	1,56E-07	0,409202853	0,46	0,289	0,002951042
ID2	1,65E-07	0,389301214	0,512	0,341	0,00311755
TAPBP	1,91E-07	0,377439591	0,638	0,482	0,003619261
IQGAP1	1,99E-07	0,434635804	0,681	0,55	0,003770564
ANXA6	2,26E-07	0,321920277	0,526	0,36	0,004286555
LBH	2,74E-07	0,390387412	0,286	0,151	0,005190662
sep-07	2,79E-07	0,387207421	0,545	0,389	0,005287628
IGF2R	3,02E-07	0,518139919	0,254	0,135	0,005730208
PSME1	3,35E-07	0,328464042	0,606	0,454	0,006346427
MYO1G	3,49E-07	0,356750962	0,3	0,166	0,006615037
ARPC2	3,55E-07	0,259231607	0,85	0,772	0,006729064
CCND2	3,93E-07	0,392134276	0,441	0,289	0,007448282

CELF2	4,00E-07	0,284609703	0,643	0,472	0,007576825
PSMB9	4,53E-07	0,401338695	0,352	0,209	0,008588606
CYTH1	4,93E-07	0,488948329	0,376	0,235	0,009342958
RASSF5	5,62E-07	0,329914957	0,488	0,319	0,010646211
MBNL1	6,32E-07	0,296036849	0,695	0,558	0,011968705
ARHGAP9	6,33E-07	0,384877588	0,329	0,19	0,011992336
ITGB1	6,63E-07	0,385490064	0,62	0,492	0,012555007
IKZF3	6,75E-07	0,259389193	0,606	0,421	0,012792362
UTRN	6,79E-07	0,362380076	0,516	0,364	0,012857752
TOP2B	7,94E-07	0,386437535	0,352	0,215	0,015050945
STK10	8,06E-07	0,342492358	0,399	0,253	0,015282517
DENND4B	8,10E-07	0,445645546	0,296	0,17	0,015352638
FNBP1	1,10E-06	0,281569732	0,573	0,405	0,020914448
MSN	1,11E-06	0,361835672	0,615	0,487	0,020988346
CD53	1,32E-06	0,270770333	0,662	0,494	0,024969796
PTPRA	1,43E-06	0,524876455	0,3	0,181	0,027023846
ANKRD44	1,56E-06	0,330207955	0,408	0,265	0,029468777
NUP210	1,68E-06	0,292803572	0,272	0,144	0,031770606
PTPN22	2,14E-06	0,406284163	0,254	0,138	0,040606431
CD8A	2,21E-06	0,373941844	0,347	0,209	0,041803011
BIN1	2,35E-06	0,33023838	0,272	0,151	0,044510867
LIMD2	2,47E-06	0,336375749	0,399	0,257	0,046770911
TAP1	2,73E-06	0,397132372	0,268	0,153	0,051825904
LY6E	2,75E-06	0,42607121	0,451	0,323	0,052116548
ADCY7	2,87E-06	0,374973208	0,319	0,193	0,054364103
TMC8	3,05E-06	0,281705872	0,404	0,253	0,057764587
PREX1	3,13E-06	0,328598435	0,413	0,272	0,059223149
NLRC3	3,22E-06	0,308917423	0,282	0,155	0,061062646
UCP2	3,88E-06	0,260578792	0,563	0,395	0,073433138
FMNL1	4,75E-06	0,354924101	0,38	0,246	0,090077288
CDC25B	5,17E-06	0,347127734	0,254	0,142	0,097987281
CLEC2D	5,67E-06	0,336498603	0,526	0,381	0,107449305
YWHAQ	5,76E-06	0,325694369	0,512	0,377	0,109176411
TES	6,32E-06	0,374901992	0,343	0,221	0,119763865
SEMA4D	8,02E-06	0,285803655	0,399	0,258	0,151970058
BZW1	9,57E-06	0,367413389	0,408	0,285	0,181400524
HCLS1	1,07E-05	0,326280358	0,592	0,451	0,203605727
CLEC2B	1,25E-05	0,310501696	0,371	0,244	0,236939573
STK4	1,62E-05	0,273236792	0,549	0,395	0,306766997
PSMB10	1,78E-05	0,292707597	0,343	0,221	0,33796817
TSC22D4	1,82E-05	0,394431573	0,263	0,156	0,344872294
MACF1	2,08E-05	0,355639143	0,512	0,388	0,394555792
ARHGAP30	2,16E-05	0,394878446	0,324	0,205	0,408418937
MCTP2	2,26E-05	0,258675013	0,286	0,168	0,429030406
CD3E	2,50E-05	0,272085599	0,638	0,492	0,473665971
PPP1R18	2,55E-05	0,341889705	0,507	0,375	0,483100555
RORA	3,03E-05	0,370115967	0,31	0,194	0,573307533
DIP2A	3,63E-05	0,37881176	0,385	0,265	0,688197534
GNA13	3,87E-05	0,411887787	0,296	0,192	0,732585097
MAPRE2	3,91E-05	0,393958793	0,362	0,253	0,74037953
DIAPH1	4,31E-05	0,391325354	0,408	0,293	0,816562836
SLAMF6	4,50E-05	0,259297231	0,357	0,232	0,852316975
PRKCB	6,44E-05	0,287655423	0,493	0,365	1
IVNS1ABP	7,26E-05	0,469790878	0,286	0,186	1
KIAA2026	7,53E-05	0,432395206	0,376	0,263	1
CASP4	8,38E-05	0,31267496	0,291	0,186	1

KCNAB2	9,00E-05	0,361591316	0,371	0,268	1
EMB	9,00E-05	0,251039845	0,399	0,265	1
HERPUD2	0,000108235	0,324500721	0,305	0,203	1
DDX6	0,000127799	0,338173694	0,371	0,264	1
TMED2	0,000130092	0,386329507	0,338	0,242	1
SIGIRR	0,000148972	0,274089967	0,343	0,23	1
CTDSP1	0,000154786	0,316487323	0,31	0,209	1
SLA	0,000156453	0,320207608	0,315	0,206	1
SSR2	0,000171628	0,269977497	0,465	0,36	1
GRK2	0,000173422	0,294844121	0,498	0,388	1
RAB29	0,000175479	0,295278878	0,376	0,274	1
VAMP2	0,000185323	0,280432274	0,634	0,544	1
SPCS3	0,000209863	0,274460138	0,333	0,231	1
BCLAF1	0,00023129	0,307260867	0,432	0,326	1
CSK	0,000260251	0,260561233	0,362	0,249	1
TFDP2	0,000288669	0,271702711	0,352	0,248	1
LUC7L3	0,000293761	0,263621667	0,451	0,344	1
ACTN4	0,000325632	0,253583717	0,615	0,514	1
PIK3R1	0,000428539	0,307423053	0,343	0,246	1
PIK3CD	0,000471172	0,255336814	0,296	0,195	1
HMGN3	0,000489934	0,349501213	0,268	0,18	1
TCIRG1	0,000528746	0,251162138	0,31	0,217	1
RNF44	0,000635053	0,405440535	0,329	0,23	1
CIAO1	0,000658677	0,317117494	0,385	0,295	1
ABHD2	0,000661785	0,323736215	0,357	0,263	1
CD52	0,000681383	0,292274433	0,587	0,481	1
RABGAP1L	0,000688151	0,269829673	0,347	0,248	1
FAM49B	0,000765881	0,275941998	0,362	0,258	1
CDC37	0,000837395	0,282113847	0,347	0,255	1
LCP2	0,00085537	0,279789585	0,338	0,237	1
NIPBL	0,000985018	0,280348399	0,404	0,308	1
LMAN2	0,001103967	0,278067645	0,277	0,196	1
NONO	0,00110604	0,280932887	0,394	0,299	1
PTBP3	0,001111869	0,28804222	0,432	0,332	1
PAFAH1B1	0,001125892	0,276991263	0,3	0,218	1
SPN	0,001149237	0,307831441	0,437	0,339	1
SELENOF	0,001336388	0,278612508	0,352	0,264	1
TCF25	0,001390321	0,278926386	0,347	0,256	1
WDR26	0,001472352	0,261297371	0,305	0,219	1
CNOT2	0,001496851	0,397666681	0,347	0,264	1
CGGBP1	0,001608119	0,297425222	0,268	0,19	1
FCER1G	0,001742711	0,43671098	0,404	0,33	1
FOXN3	0,001871963	0,259401954	0,46	0,364	1
TRIM33	0,0018908	0,266524329	0,324	0,242	1
ZNF148	0,001902514	0,264499654	0,268	0,186	1
ARF6	0,00202622	0,265172906	0,404	0,312	1
CAPN15	0,002105576	0,456751865	0,272	0,195	1
BUB3	0,002340422	0,347090824	0,277	0,202	1
ADAR	0,003015495	0,296439674	0,399	0,323	1
SHISA5	0,003384492	0,278631858	0,39	0,313	1
SELENOT	0,00365862	0,283639227	0,343	0,263	1
CSNK1G2	0,003963555	0,305103589	0,305	0,227	1
PAIP2	0,00412224	0,292607493	0,319	0,24	1
NCOA1	0,004612699	0,305715418	0,277	0,203	1
CAST	0,004692873	0,277580663	0,418	0,346	1
BIRC6	0,004878972	0,251074194	0,324	0,245	1

EIF3G	0,005239264	0,252032666	0,305	0,231	1
PSMB8	0,007589675	0,25540176	0,263	0,195	1
MAN1A2	0,007744377	0,282625095	0,272	0,202	1

Cluster 4 marker genes

gene	p_val	avg_logFC	pct.1	pct.2	p_val_adj
CD2	7,03E-18	0,670745401	0,652	0,36	1,33E-13
IL7R	9,89E-18	0,776748766	0,573	0,291	1,87E-13
CD69	1,82E-16	0,748762749	0,393	0,16	3,45E-12
CD3D	3,93E-15	0,543901192	0,601	0,308	7,45E-11
RAC2	1,65E-14	0,523107344	0,798	0,524	3,12E-10
IL32	2,51E-14	0,524102073	0,781	0,515	4,76E-10
SPOCK2	6,80E-14	0,611704587	0,601	0,345	1,29E-09
CD3E	5,97E-12	0,409907852	0,73	0,488	1,13E-07
CD6	6,61E-12	0,563702062	0,315	0,131	1,25E-07
CD52	7,46E-12	0,466112579	0,73	0,474	1,41E-07
B2M	1,55E-11	0,335608475	0,978	0,936	2,94E-07
PTPRC	5,72E-11	0,397908107	0,826	0,635	1,08E-06
ACAP1	1,35E-10	0,514607847	0,421	0,22	2,56E-06
PBXIP1	1,59E-10	0,731802833	0,517	0,327	3,00E-06
SAMSN1	8,41E-10	0,429116564	0,337	0,163	1,59E-05
LTB	9,91E-10	0,501547441	0,438	0,236	1,88E-05
GZMA	1,10E-09	0,37945825	0,556	0,326	2,09E-05
EMB	1,16E-09	0,51856368	0,455	0,263	2,19E-05
FAM102A	1,16E-09	0,502849019	0,382	0,194	2,20E-05
CYFIP2	1,21E-09	0,497196253	0,489	0,292	2,29E-05
MIAT	3,12E-09	0,542273157	0,393	0,208	5,92E-05
TXLNGY	3,35E-09	0,494720089	0,287	0,133	6,35E-05
PARP8	3,55E-09	0,544979091	0,393	0,215	6,72E-05
RASGRP1	3,63E-09	0,518948295	0,343	0,173	6,88E-05
LCK	4,26E-09	0,398104137	0,483	0,277	8,08E-05
GZMK	6,51E-09	0,45055576	0,416	0,227	0,000123399
FLT3LG	6,92E-09	0,542857977	0,331	0,168	0,000131152
ETS1	1,04E-08	0,390269747	0,657	0,456	0,000197927
EML4	1,92E-08	0,473039383	0,472	0,297	0,000363579
PTPRCAP	2,10E-08	0,439520672	0,669	0,476	0,00039759
FYB1	3,02E-08	0,349698464	0,747	0,567	0,000572499
CD53	3,77E-08	0,349573899	0,68	0,495	0,000714383
CCL5	3,87E-08	0,273888361	0,708	0,494	0,000732964
TBC1D10C	5,18E-08	0,451337002	0,309	0,157	0,000980972
KLRB1	6,49E-08	0,650334421	0,287	0,144	0,001230318
CD44	6,87E-08	0,335724541	0,736	0,565	0,001302092
TC2N	8,24E-08	0,395412397	0,365	0,197	0,001561004
SLC38A1	8,32E-08	0,404333134	0,472	0,287	0,001576395
TCF7	9,38E-08	0,339519546	0,41	0,229	0,001778349
HLA-B	1,11E-07	0,254222343	0,961	0,916	0,00209906
GOLGA8A	1,33E-07	0,43232908	0,416	0,246	0,002516679
HLA-C	1,48E-07	0,253317058	0,921	0,86	0,002799472
CCND2	1,53E-07	0,434669308	0,455	0,29	0,002906793
CRIP1	1,70E-07	0,427359257	0,618	0,458	0,003223331
IL2RG	1,95E-07	0,300132133	0,792	0,608	0,003694655
DOCK8	1,97E-07	0,491052969	0,388	0,232	0,003727936
ARL4C	2,13E-07	0,372766059	0,596	0,409	0,004032945
KIAA1551	2,32E-07	0,499357285	0,489	0,326	0,004396214
SUN2	2,45E-07	0,331287547	0,596	0,421	0,004650741
HLA-A	2,68E-07	0,265893987	0,927	0,871	0,005083493
ATXN7	3,53E-07	0,468025305	0,343	0,193	0,006697693
DDX5	4,94E-07	0,329746353	0,815	0,689	0,009369064
TNRC6C	5,34E-07	0,516151144	0,371	0,217	0,010117311

CLEC2D	6,62E-07	0,415725393	0,539	0,382	0,012551516
MYL12A	7,00E-07	0,301186239	0,742	0,606	0,013268977
AAK1	7,64E-07	0,329897729	0,685	0,543	0,014478052
ZC3HAV1	8,11E-07	0,372156914	0,551	0,396	0,015370342
CD96	1,16E-06	0,342195858	0,393	0,229	0,021920568
NFATC2	1,20E-06	0,485596445	0,382	0,239	0,022696933
ITM2A	1,24E-06	0,522264089	0,399	0,249	0,023528212
DGKA	1,29E-06	0,405946903	0,433	0,277	0,024499963
SYNE2	1,48E-06	0,43843543	0,545	0,377	0,02798752
RUNX3	1,49E-06	0,406929634	0,522	0,363	0,028211557
SARAF	1,55E-06	0,380770105	0,691	0,552	0,029388386
HLA-F	1,61E-06	0,432326663	0,551	0,399	0,030577992
AKNA	2,44E-06	0,373362175	0,517	0,357	0,046260819
sep-01	2,84E-06	0,296145835	0,337	0,19	0,053753881
FOXO1	3,59E-06	0,411621875	0,376	0,238	0,068056897
TPT1	5,21E-06	0,266922723	0,955	0,915	0,098796318
FYN	5,32E-06	0,415912189	0,517	0,38	0,100796366
ORC3	5,49E-06	0,449601918	0,32	0,189	0,103995622
CD48	6,39E-06	0,31680101	0,607	0,457	0,121146607
PDE7A	7,13E-06	0,391420135	0,36	0,217	0,135203647
WNK1	8,17E-06	0,333895345	0,528	0,388	0,154745904
AHNAK	8,55E-06	0,298532331	0,809	0,7	0,161963767
GBP5	1,17E-05	0,439418317	0,32	0,196	0,221387571
IFITM1	1,46E-05	0,264173475	0,713	0,567	0,275845733
ARHGEF1	1,67E-05	0,361221793	0,483	0,349	0,315895551
TNFAIP3	1,87E-05	0,37646921	0,354	0,22	0,355229307
CEP85L	2,12E-05	0,310940577	0,264	0,147	0,401983122
TMC8	2,92E-05	0,495459746	0,382	0,256	0,552675742
SYTL1	3,23E-05	0,384567746	0,27	0,154	0,611295528
ZAP70	3,39E-05	0,291025542	0,281	0,159	0,641901408
ANKRD12	4,69E-05	0,316186056	0,657	0,575	0,889607569
DIAPH1	4,70E-05	0,38968269	0,416	0,293	0,890533143
EVL	4,90E-05	0,424868824	0,494	0,368	0,928088709
DEFA6	4,95E-05	0,380940696	0,326	0,205	0,937478022
RGCC	5,30E-05	0,520134543	0,303	0,188	1
BCL11B	7,94E-05	0,250466004	0,438	0,296	1
TSC22D3	8,03E-05	0,393612711	0,478	0,363	1
LBH	8,18E-05	0,303783841	0,264	0,154	1
KLF5	9,37E-05	0,473604571	0,348	0,237	1
RASAL3	9,75E-05	0,338429854	0,281	0,17	1
sep-06	9,83E-05	0,411011327	0,461	0,34	1
MDFIC	0,000101379	0,34581689	0,399	0,282	1
PAG1	0,000101963	0,303996591	0,433	0,304	1
CALM1	0,000114608	0,259594421	0,871	0,842	1
ZFP36L2	0,000131682	0,3549615	0,685	0,607	1
LPIN1	0,000141093	0,478872462	0,331	0,225	1
YPEL5	0,000143638	0,395864986	0,393	0,282	1
RORA	0,000158658	0,305930843	0,309	0,195	1
NCK2	0,000165756	0,322454746	0,292	0,183	1
FXYD5	0,000170962	0,271770773	0,64	0,528	1
ITGAL	0,00017209	0,316551394	0,494	0,358	1
STK17B	0,000185375	0,399930048	0,511	0,418	1
BTG1	0,00018566	0,338128735	0,663	0,578	1
MBNL1	0,000198222	0,304397468	0,646	0,562	1
GNG2	0,000199355	0,323358683	0,309	0,194	1
EIF4G2	0,000203246	0,256957115	0,708	0,583	1

ITK	0,000219774	0,271676735	0,258	0,153	1
H2AFV	0,000234989	0,317592696	0,371	0,263	1
TMC6	0,000272444	0,300532323	0,315	0,205	1
PAXX	0,000276332	0,363073969	0,27	0,171	1
DDX24	0,000355567	0,28047596	0,41	0,299	1
ELOVL5	0,000375351	0,366427201	0,303	0,203	1
HUWE1	0,000420723	0,450523621	0,309	0,219	1
ANTXR2	0,000586028	0,36376218	0,27	0,175	1
RNF44	0,000601383	0,334336971	0,331	0,231	1
OGT	0,000607623	0,263678761	0,455	0,34	1
LDHB	0,000617466	0,266118342	0,444	0,332	1
NKTR	0,000618721	0,301430735	0,517	0,417	1
PIK3CD	0,000783496	0,319529533	0,292	0,197	1
PIK3IP1	0,000853245	0,385350229	0,292	0,2	1
SRSF11	0,000859415	0,330779684	0,612	0,525	1
UBE2G2	0,000924048	0,316496392	0,404	0,312	1
OGA	0,000933185	0,368798817	0,427	0,333	1
CD28	0,000968699	0,277076983	0,264	0,169	1
LPXN	0,000984506	0,35604823	0,331	0,239	1
NBEAL2	0,001053813	0,304258311	0,298	0,201	1
SMCHD1	0,001108435	0,277672701	0,388	0,28	1
C12orf57	0,001115019	0,356560257	0,393	0,282	1
KMT2C	0,001119266	0,299687179	0,41	0,311	1
IKZF1	0,001138334	0,349662938	0,416	0,307	1
ARID1A	0,001165715	0,311869618	0,41	0,31	1
GMFG	0,001255033	0,27208219	0,449	0,349	1
SNRPD2	0,001312999	0,280423569	0,455	0,36	1
PPP2R5C	0,001326379	0,306401031	0,388	0,287	1
TMEM109	0,001382809	0,388222079	0,32	0,233	1
SSR2	0,001412654	0,275057548	0,455	0,362	1
CYTH1	0,001464932	0,258064655	0,337	0,239	1
AKAP9	0,001475873	0,318957049	0,466	0,378	1
RFLNB	0,00160434	0,257315051	0,253	0,165	1
ICAM3	0,001842722	0,263455799	0,281	0,189	1
SF3B1	0,001903647	0,255766854	0,612	0,514	1
AKIRIN1	0,001919274	0,278544908	0,281	0,195	1
ANXA1	0,002175204	0,392659622	0,584	0,5	1
RABAC1	0,002434229	0,354808277	0,264	0,187	1
JAK1	0,002520786	0,252448615	0,511	0,419	1
CCNI	0,002646367	0,272827178	0,584	0,493	1
HECA	0,002896339	0,326974813	0,36	0,273	1
IDS	0,003693909	0,260489011	0,573	0,515	1
BIRC6	0,003914087	0,401572996	0,32	0,246	1
UBE2D3	0,003985295	0,25519348	0,455	0,37	1
CELF2	0,004310863	0,250635144	0,551	0,48	1
HINT1	0,004363823	0,276727742	0,461	0,393	1
LEPROTL1	0,00445665	0,284248706	0,32	0,238	1
CRYBG1	0,004608628	0,30660434	0,253	0,176	1
MBD1	0,004765896	0,293793328	0,371	0,294	1
NR3C1	0,004822841	0,321976484	0,449	0,383	1
CYTIP	0,005089856	0,288998476	0,292	0,212	1
ATRX	0,005454914	0,299250518	0,517	0,441	1
ARHGAP9	0,005618699	0,315627802	0,275	0,195	1
RNF19A	0,006355276	0,402144325	0,264	0,194	1
EIF3F	0,006622238	0,266023622	0,433	0,35	1
GAS5	0,006793017	0,30406927	0,36	0,281	1

FNBP1	0,006987591	0,282091866	0,478	0,413	1
PRRC2B	0,006997168	0,323953124	0,365	0,293	1
TIAL1	0,007429892	0,272377113	0,275	0,2	1
HIPK1	0,008652685	0,421729681	0,264	0,201	1
OPTN	0,009133322	0,333012174	0,298	0,229	1

Cluster 5 marker genes

gene	p_val	avg_logFC	pct.1	pct.2	p_val_adj
C1QA	2,85E-259	2,814906932	0,899	0,075	5,39E-255
C1QC	6,56E-255	2,616653872	0,876	0,074	1,24E-250
FOLR2	1,89E-203	1,616254044	0,483	0,016	3,58E-199
C1QB	6,75E-200	2,873122495	0,871	0,11	1,28E-195
MS4A6A	1,12E-168	1,967129432	0,798	0,097	2,12E-164
CD14	9,33E-166	2,000567341	0,865	0,122	1,77E-161
SLCO2B1	1,71E-159	1,579584896	0,556	0,041	3,24E-155
CSF1R	2,74E-146	1,68408435	0,747	0,096	5,19E-142
CD163	1,71E-145	1,972690214	0,691	0,081	3,24E-141
MAFB	4,14E-144	1,906838862	0,831	0,131	7,84E-140
MSR1	2,14E-143	1,757110463	0,725	0,091	4,05E-139
F13A1	1,37E-136	1,768068293	0,5	0,039	2,59E-132
VSIG4	4,79E-125	1,58958088	0,567	0,06	9,07E-121
MARCKS	3,14E-120	1,786648457	0,882	0,199	5,95E-116
SIGLEC1	2,50E-119	1,164042201	0,376	0,022	4,73E-115
FCGR2A	2,36E-118	1,651381838	0,534	0,056	4,47E-114
DAB2	5,41E-118	1,683158797	0,708	0,112	1,03E-113
MS4A4A	5,23E-112	1,914864197	0,803	0,172	9,92E-108
FCGR3A	3,20E-108	1,425342424	0,556	0,067	6,07E-104
STAB1	1,50E-106	1,44719789	0,545	0,066	2,84E-102
CCL3	1,09E-103	1,570116975	0,517	0,06	2,06E-99
AIF1	5,15E-103	1,329900623	0,73	0,126	9,76E-99
LGMN	2,28E-102	1,860078329	0,567	0,08	4,33E-98
IGSF21	1,78E-98	1,049379556	0,315	0,019	3,38E-94
HLA-DMB	8,06E-97	1,389097339	0,742	0,146	1,53E-92
HLA-DRB1	9,95E-95	1,878973196	0,983	0,529	1,89E-90
C3AR1	1,52E-94	1,110376659	0,5	0,061	2,89E-90
SLC40A1	3,66E-94	1,666859026	0,567	0,086	6,94E-90
CTSB	6,04E-90	1,770145117	0,972	0,489	1,14E-85
HLA-DMA	7,43E-90	1,438622351	0,747	0,17	1,41E-85
MPEG1	2,60E-87	1,304711254	0,607	0,101	4,93E-83
GPR34	7,35E-87	0,885306198	0,287	0,018	1,39E-82
HLA-DPB1	2,15E-86	1,654866258	0,955	0,44	4,07E-82
HLA-DRA	2,41E-86	1,807410121	0,983	0,54	4,56E-82
GNAO1	4,50E-86	1,553525629	0,854	0,258	8,53E-82
HLA-DRB6	4,57E-86	1,742135781	0,742	0,187	8,65E-82
HLA-DQA1	1,54E-85	1,54287678	0,831	0,236	2,92E-81
CPVL	1,82E-85	1,16267109	0,534	0,076	3,44E-81
CYBB	2,49E-85	1,348414508	0,697	0,141	4,72E-81
CD74	5,37E-84	1,708370264	1	0,869	1,02E-79
SPI1	8,43E-84	1,230300595	0,713	0,146	1,60E-79
ADAP2	2,26E-83	1,387844595	0,545	0,086	4,28E-79
CD68	3,61E-82	1,460368956	0,758	0,188	6,84E-78
TGFBI	8,00E-81	1,172105004	0,652	0,126	1,52E-76
HLA-DPA1	9,52E-80	1,690581511	0,966	0,604	1,80E-75
FCGRT	2,41E-79	1,387671097	0,848	0,268	4,57E-75
SLC15A3	1,36E-78	0,980290363	0,444	0,057	2,58E-74
TMEM176B	3,30E-76	1,517011707	0,59	0,115	6,26E-72
PSAP	1,50E-75	1,518312888	0,961	0,537	2,85E-71
FTL	5,45E-74	1,498038644	0,989	0,736	1,03E-69
IER3	7,89E-74	1,609782754	0,652	0,151	1,50E-69
NPC2	2,13E-73	1,375202152	0,837	0,296	4,03E-69
GGTA1P	1,06E-72	0,931191123	0,404	0,052	2,02E-68

TYROBP	8,39E-71	1,246045702	0,972	0,43	1,59E-66
HMOX1	2,68E-70	0,970688297	0,354	0,039	5,07E-66
NCF4	2,71E-70	0,875907488	0,449	0,064	5,14E-66
CXCL16	5,69E-70	1,362584437	0,697	0,179	1,08E-65
PLTP	4,16E-69	1,648376591	0,68	0,181	7,88E-65
CST3	5,59E-69	1,254045352	0,927	0,394	1,06E-64
GRN	6,25E-69	1,284344021	0,893	0,358	1,18E-64
CXCL8	1,76E-67	1,655414055	0,427	0,064	3,34E-63
C2	1,91E-66	1,055750418	0,303	0,03	3,61E-62
RNASE6	8,17E-65	1,046274956	0,455	0,073	1,55E-60
LILRB4	3,63E-64	1,058921237	0,382	0,052	6,88E-60
SAT1	7,05E-64	1,372699105	0,854	0,341	1,34E-59
CTSH	2,24E-63	0,930325273	0,59	0,122	4,24E-59
ME1	1,15E-62	0,644163589	0,275	0,026	2,18E-58
CREG1	5,31E-62	1,174165588	0,573	0,132	1,01E-57
SGK1	9,84E-62	1,272203289	0,517	0,103	1,86E-57
LILRB2	2,08E-61	0,952366309	0,522	0,102	3,94E-57
CLEC7A	1,39E-60	0,927487521	0,534	0,107	2,62E-56
CXCL3	1,81E-60	1,389653585	0,331	0,042	3,43E-56
KCTD12	1,08E-59	1,171648914	0,663	0,18	2,04E-55
TMEM176A	1,14E-59	1,069356307	0,433	0,073	2,16E-55
GLUL	2,05E-59	1,251490339	0,77	0,267	3,88E-55
SLAMF8	7,60E-59	0,792821044	0,32	0,039	1,44E-54
AP1B1	2,14E-58	1,012111889	0,562	0,128	4,05E-54
OLFML2B	5,67E-58	0,877276566	0,348	0,047	1,08E-53
TMEM51	1,25E-57	0,815935573	0,292	0,033	2,36E-53
CXCL2	1,73E-57	1,280649861	0,404	0,066	3,27E-53
CD93	7,88E-57	1,013557556	0,534	0,113	1,49E-52
CEBPD	3,87E-56	0,987536483	0,522	0,113	7,33E-52
PLAU	6,90E-56	1,072172956	0,343	0,048	1,31E-51
LRP1	4,50E-55	0,947577626	0,562	0,129	8,53E-51
TNFAIP2	1,70E-54	1,028024459	0,545	0,124	3,22E-50
GPX1	1,05E-53	1,227145308	0,764	0,286	2,00E-49
FPR3	3,43E-53	0,74960045	0,326	0,044	6,50E-49
C5AR1	5,54E-53	1,166391431	0,596	0,158	1,05E-48
FUCA1	7,07E-53	0,926487505	0,36	0,056	1,34E-48
LY86	7,40E-53	0,71916012	0,399	0,067	1,40E-48
RAB32	9,15E-53	0,786984459	0,354	0,054	1,73E-48
TBXAS1	2,31E-52	0,86950574	0,399	0,069	4,38E-48
IGSF6	5,71E-52	0,841783938	0,331	0,048	1,08E-47
SIRPA	6,26E-52	0,985868518	0,489	0,104	1,19E-47
RNF130	8,74E-52	0,940387237	0,567	0,143	1,66E-47
CCR1	9,71E-52	0,92219135	0,416	0,076	1,84E-47
PILRA	1,08E-51	0,807864375	0,348	0,053	2,05E-47
TLR2	2,36E-51	0,727924268	0,371	0,06	4,47E-47
LYZ	5,11E-51	0,78233653	0,736	0,24	9,69E-47
CD86	8,32E-51	0,781144146	0,376	0,062	1,58E-46
FCER1G	2,87E-50	1,040881953	0,792	0,309	5,45E-46
RAB31	3,45E-50	1,106522605	0,579	0,157	6,54E-46
NPL	1,14E-49	0,719050942	0,287	0,037	2,16E-45
TTYH3	3,07E-49	0,901957792	0,478	0,105	5,81E-45
IGF1	4,32E-49	1,07506969	0,27	0,034	8,18E-45
NELL1	1,72E-48	1,020431815	0,455	0,099	3,27E-44
mrt-01	2,57E-48	0,814141466	0,416	0,079	4,87E-44
CTSS	3,69E-48	0,860206653	0,86	0,367	7,00E-44
EGR1	6,67E-48	1,180952338	0,708	0,243	1,26E-43

MNDA	2,75E-47	0,682811747	0,382	0,066	5,20E-43
PLD3	1,81E-46	1,030808061	0,567	0,156	3,43E-42
ASAH1	1,99E-46	1,080548574	0,629	0,201	3,77E-42
GPNMB	4,06E-46	1,620114172	0,522	0,139	7,69E-42
BLVRB	1,50E-45	0,751260926	0,36	0,065	2,83E-41
PDK4	3,20E-45	0,993039678	0,371	0,07	6,07E-41
CTSZ	3,40E-45	1,22932306	0,438	0,097	6,43E-41
HLA-DRB5	5,62E-45	1,489149785	0,534	0,147	1,06E-40
MFSD1	9,55E-45	0,879960358	0,534	0,144	1,81E-40
LAIR1	9,98E-45	0,92580275	0,579	0,162	1,89E-40
RIN2	4,76E-44	1,104272792	0,399	0,084	9,02E-40
THEMIS2	1,34E-43	0,642070774	0,393	0,076	2,54E-39
CTSD	1,68E-43	1,497883312	0,77	0,365	3,18E-39
HLA-DQB2	2,41E-42	0,68130498	0,27	0,038	4,57E-38
RBM47	6,39E-42	0,926924213	0,539	0,149	1,21E-37
FOS	1,07E-41	1,188679752	0,787	0,363	2,03E-37
ZFP36L1	7,33E-41	1,062720213	0,899	0,541	1,39E-36
RGL1	8,34E-41	0,93033174	0,264	0,039	1,58E-36
CFD	1,08E-40	0,828845836	0,36	0,071	2,04E-36
IFI30	2,56E-40	1,019574867	0,848	0,437	4,84E-36
MRAP	5,72E-40	0,4994849	0,287	0,045	1,08E-35
DUSP3	8,66E-40	0,844602863	0,416	0,098	1,64E-35
NFKBIA	9,91E-40	1,240285947	0,713	0,305	1,88E-35
EPB41L3	1,25E-39	0,706322722	0,337	0,064	2,37E-35
ADAM28	1,61E-39	0,637548088	0,309	0,053	3,04E-35
SDC3	4,07E-39	0,981529633	0,404	0,094	7,71E-35
ADAM9	8,27E-39	0,705806251	0,348	0,07	1,57E-34
TNFSF13B	1,14E-38	0,735553465	0,433	0,102	2,16E-34
HNMT	2,24E-38	0,858541437	0,399	0,092	4,24E-34
HCK	3,51E-38	0,709472892	0,438	0,106	6,65E-34
MAF	4,89E-38	1,077557266	0,618	0,211	9,27E-34
ABCA1	2,20E-37	0,862315892	0,416	0,099	4,18E-33
TYMP	3,82E-37	0,759748048	0,584	0,181	7,23E-33
ITGAX	6,39E-37	0,874680548	0,438	0,109	1,21E-32
NINJ1	6,95E-37	0,701862708	0,382	0,086	1,32E-32
CTSL	8,29E-37	0,954813458	0,455	0,124	1,57E-32
ALOX5	1,11E-36	0,712797889	0,41	0,096	2,11E-32
ARRB2	1,30E-36	0,866246209	0,691	0,267	2,46E-32
LRRC25	1,37E-36	0,670158446	0,371	0,081	2,59E-32
HLA-DOA	1,69E-36	0,724809413	0,421	0,103	3,20E-32
CEL	3,32E-36	1,193960079	0,567	0,2	6,29E-32
PLXNB2	1,52E-35	0,719313129	0,444	0,116	2,87E-31
ZFHX3	1,57E-35	0,998011676	0,539	0,171	2,97E-31
CMKLR1	3,40E-35	0,769429988	0,292	0,055	6,45E-31
DSE	3,55E-35	0,602296197	0,331	0,068	6,73E-31
CYFIP1	9,84E-35	0,911369706	0,556	0,185	1,86E-30
RNASE1	1,13E-34	1,694041992	0,567	0,208	2,14E-30
FRMD4A	1,40E-34	0,674955664	0,264	0,046	2,66E-30
FPR1	2,06E-34	0,544992225	0,315	0,061	3,91E-30
SHTN1	2,08E-34	0,665556575	0,303	0,059	3,94E-30
LYN	2,37E-34	0,675843766	0,483	0,136	4,48E-30
CD83	3,96E-34	0,793468775	0,404	0,101	7,51E-30
FCGR2B	4,33E-34	0,791207384	0,303	0,06	8,20E-30
EMILIN2	5,45E-34	0,619229508	0,292	0,055	1,03E-29
KCNMA1	6,17E-34	0,640690934	0,292	0,055	1,17E-29
SPRED1	7,31E-34	0,658358381	0,371	0,088	1,38E-29

GAA	7,61E-34	0,702116695	0,399	0,099	1,44E-29
SECTM1	9,70E-34	0,589777257	0,298	0,057	1,84E-29
TIMP2	2,02E-33	0,793700558	0,601	0,213	3,83E-29
DMXL2	2,38E-33	0,675246241	0,348	0,077	4,52E-29
P2RY13	5,11E-33	0,752825774	0,264	0,047	9,67E-29
SERPINA1	1,13E-32	0,502135117	0,5	0,142	2,14E-28
RUNX1	1,74E-32	0,835318985	0,416	0,113	3,30E-28
LST1	3,82E-32	0,543070665	0,41	0,103	7,25E-28
UNC93B1	4,26E-32	0,673509614	0,416	0,109	8,07E-28
SYK	6,66E-32	0,696415855	0,534	0,171	1,26E-27
LPAR6	7,26E-32	0,746929398	0,444	0,128	1,38E-27
PTAFR	3,53E-31	0,882263117	0,506	0,16	6,70E-27
HLA-DQA2	5,61E-31	0,649530892	0,287	0,056	1,06E-26
PLAUR	1,02E-30	0,7293083	0,472	0,143	1,94E-26
MGAT1	1,53E-30	0,822061468	0,545	0,184	2,90E-26
IRF8	1,67E-30	0,408910278	0,275	0,052	3,17E-26
PLBD1	3,68E-30	0,557020782	0,287	0,059	6,98E-26
ARHGAP18	1,13E-29	0,618780742	0,41	0,112	2,14E-25
GM2A	1,42E-29	0,642440919	0,41	0,116	2,68E-25
TLR7	1,54E-29	0,680433534	0,303	0,067	2,91E-25
LY96	3,71E-29	0,703423675	0,281	0,059	7,04E-25
RAC1	6,14E-29	0,726368081	0,826	0,489	1,16E-24
TNF	9,85E-29	0,768785629	0,258	0,052	1,87E-24
YWHAH	1,07E-28	0,774514112	0,483	0,163	2,03E-24
MAN2B1	1,33E-28	0,723654299	0,466	0,151	2,53E-24
CD63	1,43E-28	0,870932458	0,747	0,398	2,71E-24
COLGALT1	1,55E-28	0,632523031	0,393	0,109	2,94E-24
PLXND1	1,57E-28	0,741551678	0,393	0,112	2,97E-24
TLR4	2,07E-28	0,650213744	0,36	0,095	3,92E-24
LAPTM5	2,72E-28	0,674901238	0,904	0,603	5,16E-24
GLIPR1L2	2,85E-28	1,220965831	0,528	0,198	5,40E-24
SLC7A7	4,05E-28	0,458845925	0,258	0,051	7,68E-24
CCDC88A	4,52E-28	0,672356578	0,556	0,201	8,56E-24
APOE	5,57E-28	1,410899119	0,427	0,139	1,06E-23
PEA15	6,50E-28	0,742255081	0,612	0,242	1,23E-23
FTH1	7,13E-28	0,736684553	0,955	0,807	1,35E-23
SDCBP	1,55E-27	0,797331392	0,674	0,317	2,93E-23
PLEKHO2	1,62E-27	0,640155981	0,292	0,066	3,08E-23
IL1B	1,64E-27	0,835994915	0,303	0,071	3,11E-23
HEXB	2,45E-27	0,601565323	0,427	0,132	4,64E-23
RGS1	2,61E-27	0,730795382	0,343	0,088	4,94E-23
ZEB2	3,61E-27	0,682641025	0,528	0,182	6,85E-23
TSPAN4	5,55E-27	0,657648553	0,258	0,054	1,05E-22
ATP6V0B	8,37E-27	0,703035208	0,618	0,251	1,59E-22
NAGK	9,10E-27	0,689740296	0,511	0,183	1,73E-22
CSF3R	1,24E-26	0,429207455	0,27	0,057	2,35E-22
APOC1	1,64E-26	0,97566764	0,275	0,062	3,11E-22
RB1	4,50E-26	0,651273639	0,382	0,113	8,53E-22
PLXDC2	6,74E-26	0,466647085	0,253	0,054	1,28E-21
HEXA	8,06E-26	0,540358827	0,371	0,105	1,53E-21
MEF2C	9,61E-26	0,535808766	0,427	0,133	1,82E-21
PPT1	1,20E-25	0,784317858	0,596	0,267	2,26E-21
BCAT1	1,57E-25	0,702985067	0,449	0,148	2,98E-21
ATP6AP1	2,11E-25	0,699528332	0,444	0,148	4,00E-21
ZFP36	7,93E-25	0,739086102	0,59	0,255	1,50E-20
IDH1	1,90E-24	0,621430275	0,281	0,069	3,60E-20

IFNGR1	2,77E-24	0,629781688	0,466	0,162	5,25E-20
RGS2	3,45E-24	0,67441412	0,36	0,106	6,54E-20
TUBA1B	5,15E-24	0,752632621	0,719	0,399	9,76E-20
DUSP1	5,96E-24	0,721526709	0,545	0,214	1,13E-19
TNFSF15	9,73E-24	0,906090373	0,275	0,069	1,84E-19
MKNK1	1,03E-23	0,60037459	0,337	0,095	1,95E-19
CPM	1,58E-23	0,917604106	0,511	0,2	3,00E-19
MPP1	2,14E-23	0,626752961	0,331	0,094	4,06E-19
ABHD12	2,27E-23	0,66836743	0,331	0,095	4,30E-19
RGS10	3,19E-23	0,575790343	0,483	0,172	6,04E-19
LHFPL2	3,88E-23	0,519622115	0,281	0,07	7,35E-19
AKR1A1	7,14E-23	0,58971596	0,421	0,141	1,35E-18
TPP1	7,20E-23	0,686561282	0,713	0,403	1,37E-18
NCF2	1,49E-22	0,54422302	0,309	0,083	2,82E-18
MEF2A	1,64E-22	0,626008029	0,438	0,153	3,10E-18
PRKCD	1,94E-22	0,480237032	0,303	0,081	3,67E-18
CEP170	2,00E-22	0,592382872	0,382	0,124	3,79E-18
ITGAM	2,07E-22	0,555580374	0,371	0,114	3,92E-18
PDGFB	3,21E-22	0,55394083	0,264	0,064	6,09E-18
JUN	3,46E-22	0,608151207	0,455	0,163	6,55E-18
CCL2	3,75E-22	1,058237542	0,315	0,09	7,10E-18
KLF6	3,80E-22	0,688286481	0,843	0,559	7,20E-18
PLEKHO1	5,77E-22	0,55840537	0,404	0,134	1,09E-17
RNASET2	6,66E-22	0,687031356	0,68	0,342	1,26E-17
BTG2	7,94E-22	0,519092611	0,365	0,114	1,50E-17
AKR1B1	7,95E-22	0,711139657	0,427	0,152	1,51E-17
HERPUD1	8,30E-22	0,741049931	0,438	0,162	1,57E-17
CAPG	1,13E-21	0,667028793	0,433	0,152	2,13E-17
PYCARD	1,63E-21	0,559571822	0,449	0,159	3,09E-17
BRI3	1,97E-21	0,664367015	0,466	0,186	3,73E-17
CREBL2	2,27E-21	0,607106247	0,421	0,151	4,30E-17
RCBTB2	2,69E-21	0,479509846	0,281	0,074	5,10E-17
ACSL1	2,71E-21	0,452086511	0,264	0,066	5,14E-17
IL18	4,02E-21	0,635693382	0,309	0,088	7,61E-17
ATP6V1B2	5,49E-21	0,582127091	0,427	0,15	1,04E-16
SOD2	5,64E-21	0,633583151	0,517	0,21	1,07E-16
NAGA	6,66E-21	0,634637134	0,298	0,086	1,26E-16
DUSP6	6,74E-21	0,77096434	0,404	0,147	1,28E-16
ADA2	8,37E-21	0,718714753	0,511	0,218	1,59E-16
FAM49B	8,55E-21	0,550780629	0,584	0,246	1,62E-16
CYTH4	1,13E-20	0,565560808	0,365	0,118	2,13E-16
NAIP	1,16E-20	0,522179137	0,393	0,131	2,19E-16
ELL2	1,19E-20	0,598555545	0,303	0,087	2,25E-16
GNS	1,24E-20	0,62070712	0,539	0,231	2,36E-16
GAS6	2,19E-20	0,476016895	0,382	0,126	4,16E-16
HBEGF	2,21E-20	0,845484324	0,292	0,084	4,19E-16
GRINA	2,36E-20	0,622629731	0,365	0,123	4,48E-16
GNB4	2,54E-20	0,554519089	0,444	0,17	4,81E-16
NAMPT	3,61E-20	0,624260555	0,466	0,188	6,83E-16
DRAM2	4,12E-20	0,449281372	0,36	0,116	7,81E-16
NCKAP1L	6,56E-20	0,525859642	0,32	0,097	1,24E-15
C20orf194	8,99E-20	0,473421133	0,27	0,074	1,70E-15
SLC16A3	1,09E-19	0,456035102	0,303	0,088	2,07E-15
LGALS9	1,28E-19	0,59646104	0,309	0,092	2,43E-15
FGL2	1,32E-19	0,638701842	0,517	0,224	2,51E-15
ST8SIA4	2,24E-19	0,449709014	0,343	0,109	4,24E-15

PMP22	2,41E-19	0,523454423	0,298	0,09	4,57E-15
QKI	4,20E-19	0,672924254	0,562	0,268	7,96E-15
AP2A2	4,73E-19	0,602103764	0,365	0,127	8,97E-15
SYNGR2	5,98E-19	0,568593648	0,472	0,196	1,13E-14
ALDH2	9,10E-19	0,555299186	0,331	0,111	1,73E-14
BNIP3L	1,37E-18	0,547059907	0,489	0,203	2,59E-14
TNFRSF1A	2,31E-18	0,506384354	0,337	0,113	4,37E-14
NCOA4	4,58E-18	0,618969321	0,522	0,239	8,68E-14
SLC37A2	7,54E-18	0,545096271	0,32	0,104	1,43E-13
DPYSL2	1,28E-17	0,641957654	0,472	0,209	2,42E-13
DUSP2	1,91E-17	0,70698628	0,287	0,091	3,62E-13
PLIN2	2,39E-17	0,757044545	0,292	0,092	4,53E-13
IL10RA	2,51E-17	0,485322127	0,472	0,194	4,76E-13
NEAT1	3,28E-17	0,612021891	0,702	0,398	6,22E-13
TPCN1	3,84E-17	0,385113984	0,27	0,079	7,28E-13
MTRNR2L1	3,98E-17	0,755540281	0,736	0,467	7,54E-13
VAMP8	4,05E-17	0,479443959	0,455	0,187	7,68E-13
TPD52L2	4,21E-17	0,460425876	0,32	0,109	7,98E-13
S100A11	4,49E-17	0,554696821	0,815	0,583	8,50E-13
GRK3	4,51E-17	0,402335028	0,315	0,102	8,55E-13
LOC441081	4,53E-17	0,612432468	0,36	0,13	8,59E-13
AOAH	4,71E-17	0,429479371	0,331	0,11	8,93E-13
CREB5	5,45E-17	0,641373847	0,253	0,075	1,03E-12
SKAP2	7,95E-17	0,468957634	0,478	0,2	1,51E-12
IFNGR2	9,71E-17	0,469844762	0,36	0,13	1,84E-12
OAZ2	1,32E-16	0,42785309	0,264	0,081	2,50E-12
LPCAT2	1,33E-16	0,504498152	0,292	0,096	2,52E-12
CLTA	1,61E-16	0,540588689	0,376	0,144	3,05E-12
IL13RA1	1,81E-16	0,530888875	0,427	0,176	3,43E-12
AP2S1	2,14E-16	0,516573362	0,494	0,221	4,06E-12
KLF4	2,72E-16	0,538986117	0,309	0,104	5,16E-12
GRB2	2,80E-16	0,489977274	0,624	0,318	5,30E-12
NFKBIZ	2,81E-16	0,6091864	0,449	0,194	5,32E-12
SLC18A1	3,00E-16	0,578496185	0,382	0,15	5,69E-12
ATP6V0C	3,18E-16	0,520830308	0,758	0,476	6,02E-12
CXCL12	3,22E-16	0,522256641	0,253	0,075	6,10E-12
MCL1	4,07E-16	0,537342898	0,68	0,402	7,72E-12
CEBPB	6,62E-16	0,501865633	0,343	0,126	1,25E-11
LILRB1	1,06E-15	0,560954423	0,309	0,108	2,02E-11
LILRB3	1,18E-15	0,456526317	0,331	0,12	2,23E-11
APLP2	1,37E-15	0,437901726	0,697	0,386	2,59E-11
GPR183	2,11E-15	0,588989472	0,331	0,122	3,99E-11
VASH1	2,17E-15	0,513471612	0,281	0,094	4,11E-11
TNFRSF1B	2,36E-15	0,427435318	0,635	0,318	4,46E-11
SPINT2	2,48E-15	0,497068006	0,461	0,199	4,70E-11
PFKFB3	3,00E-15	0,481333412	0,337	0,125	5,69E-11
SCARB2	4,44E-15	0,37970464	0,371	0,144	8,41E-11
RNF13	4,92E-15	0,54072157	0,416	0,178	9,33E-11
MYO9B	5,40E-15	0,410125655	0,433	0,178	1,02E-10
RIN3	6,11E-15	0,422203487	0,264	0,084	1,16E-10
SNX2	6,19E-15	0,47814833	0,354	0,138	1,17E-10
WASHC4	6,49E-15	0,44668185	0,331	0,121	1,23E-10
FABP5	6,86E-15	0,550651828	0,393	0,156	1,30E-10
SNX6	7,03E-15	0,453166972	0,41	0,173	1,33E-10
LAPTM4A	8,04E-15	0,36301263	0,545	0,251	1,52E-10
H2AFY	8,15E-15	0,465933394	0,579	0,293	1,55E-10

RBPJ	8,60E-15	0,505872843	0,629	0,346	1,63E-10
C1orf162	9,79E-15	0,358764016	0,427	0,175	1,86E-10
CD4	1,35E-14	0,53127926	0,64	0,368	2,56E-10
BASP1	1,73E-14	0,398783576	0,287	0,098	3,27E-10
IER2	2,77E-14	0,507374332	0,449	0,205	5,26E-10
SLC43A3	3,61E-14	0,372843203	0,287	0,099	6,85E-10
RAB5C	3,71E-14	0,494870076	0,376	0,156	7,04E-10
TMBIM6	5,43E-14	0,460548963	0,871	0,645	1,03E-09
GDE1	6,65E-14	0,463191772	0,348	0,141	1,26E-09
ZNF385A	6,71E-14	0,389426069	0,315	0,117	1,27E-09
PABPC4	7,54E-14	0,466568317	0,596	0,32	1,43E-09
LAMP1	1,09E-13	0,473168271	0,584	0,303	2,07E-09
SCAMP2	1,15E-13	0,586648442	0,275	0,096	2,18E-09
RHOQ	1,52E-13	0,506451022	0,371	0,158	2,89E-09
SERPING1	1,86E-13	0,343549605	0,337	0,13	3,53E-09
RNF149	2,00E-13	0,442252054	0,444	0,204	3,80E-09
IRS2	3,80E-13	0,359138638	0,275	0,096	7,20E-09
RAP2B	4,00E-13	0,429593463	0,399	0,172	7,58E-09
LGALS3BP	4,02E-13	0,379193914	0,253	0,085	7,63E-09
HSBP1	4,96E-13	0,490791779	0,331	0,136	9,39E-09
HSPA1A	4,99E-13	0,57300696	0,32	0,129	9,46E-09
METTL7A	6,23E-13	0,523601825	0,466	0,229	1,18E-08
NR4A2	6,32E-13	0,593720101	0,438	0,201	1,20E-08
SLC39A1	6,80E-13	0,359653905	0,253	0,087	1,29E-08
ANXA5	8,19E-13	0,461475569	0,691	0,413	1,55E-08
ATP2B1	8,99E-13	0,412879312	0,567	0,29	1,70E-08
SRRM4	1,03E-12	0,631459715	0,253	0,088	1,95E-08
ATP6V1C1	1,18E-12	0,283964121	0,258	0,091	2,24E-08
RHOG	1,59E-12	0,437706911	0,444	0,209	3,01E-08
ARAP1	1,82E-12	0,421248242	0,275	0,101	3,45E-08
TUBA1A	1,87E-12	0,449566323	0,455	0,219	3,55E-08
DDAH2	1,98E-12	0,430408996	0,298	0,115	3,75E-08
GABARAP	2,24E-12	0,50500087	0,77	0,536	4,24E-08
CD300A	2,63E-12	0,284508591	0,253	0,088	4,99E-08
PIK3AP1	3,08E-12	0,267191072	0,309	0,118	5,84E-08
ARL8B	3,14E-12	0,443559106	0,331	0,138	5,96E-08
JUNB	3,18E-12	0,446321156	0,708	0,436	6,03E-08
PPP1R15A	3,24E-12	0,48180018	0,354	0,151	6,14E-08
OS9	4,53E-12	0,447959939	0,522	0,268	8,59E-08
CMTM6	6,06E-12	0,416619456	0,618	0,336	1,15E-07
FRMD4B	6,84E-12	0,34803753	0,287	0,11	1,30E-07
ITM2B	7,36E-12	0,410757377	0,904	0,794	1,40E-07
PEPD	7,44E-12	0,422588273	0,331	0,139	1,41E-07
SLC35F6	8,52E-12	0,351426888	0,258	0,095	1,61E-07
RRBP1	8,91E-12	0,435210299	0,298	0,121	1,69E-07
VPS35	9,23E-12	0,362346198	0,427	0,199	1,75E-07
GADD45B	9,26E-12	0,485732832	0,393	0,181	1,75E-07
RHOB	1,25E-11	0,293516783	0,27	0,1	2,38E-07
MTSS1	1,39E-11	0,461694683	0,275	0,107	2,63E-07
ST8SIA1	1,48E-11	0,526307586	0,292	0,118	2,81E-07
LITAF	2,18E-11	0,46789031	0,725	0,497	4,14E-07
SH2B3	2,19E-11	0,503569628	0,309	0,132	4,15E-07
CMTM3	2,58E-11	0,375248844	0,399	0,184	4,88E-07
HSP90AA1	2,99E-11	0,453219087	0,865	0,662	5,66E-07
TUBA1C	4,24E-11	0,286765609	0,309	0,126	8,03E-07
RTN4	5,51E-11	0,398818406	0,708	0,45	1,04E-06

PDXK	6,37E-11	0,369154179	0,264	0,103	1,21E-06
ATP1B3	6,59E-11	0,403643589	0,522	0,285	1,25E-06
CD84	6,78E-11	0,494804028	0,669	0,416	1,28E-06
ITPRIPL2	6,92E-11	0,496661359	0,388	0,188	1,31E-06
MGAT4A	7,55E-11	0,296622042	0,433	0,207	1,43E-06
FGD4	1,20E-10	0,448154743	0,253	0,098	2,27E-06
CYBA	1,23E-10	0,44197358	0,685	0,435	2,34E-06
RPN2	1,36E-10	0,366252475	0,567	0,317	2,58E-06
MAN1A1	1,39E-10	0,388737601	0,326	0,142	2,64E-06
GAS7	1,42E-10	0,354243808	0,416	0,199	2,69E-06
PTPRE	1,45E-10	0,406212998	0,376	0,173	2,75E-06
LAMP2	1,60E-10	0,468948377	0,489	0,267	3,04E-06
GPX3	1,83E-10	0,401029221	0,258	0,103	3,46E-06
LAMTOR2	2,22E-10	0,354407474	0,258	0,103	4,20E-06
PRKACA	2,54E-10	0,341593325	0,331	0,149	4,81E-06
DNASE2	2,62E-10	0,387960377	0,253	0,099	4,97E-06
KIAA0930	3,76E-10	0,341084969	0,337	0,152	7,12E-06
BAZ2B	4,34E-10	0,382361033	0,393	0,19	8,22E-06
NIPSNAP2	5,16E-10	0,294741726	0,264	0,106	9,77E-06
VMP1	6,92E-10	0,348732779	0,382	0,183	1,31E-05
PLEK	7,03E-10	0,374356727	0,438	0,222	1,33E-05
GNAI2	7,22E-10	0,365295295	0,837	0,649	1,37E-05
TALDO1	7,78E-10	0,295746596	0,376	0,177	1,47E-05
PICALM	9,54E-10	0,271919046	0,433	0,215	1,81E-05
TMEM107	1,02E-09	0,387808074	0,258	0,106	1,93E-05
KDELR1	1,02E-09	0,40077752	0,354	0,168	1,93E-05
ETS2	1,29E-09	0,387203319	0,27	0,112	2,44E-05
EPN1	1,84E-09	0,461029799	0,258	0,109	3,49E-05
YBX3	1,87E-09	0,254532636	0,388	0,187	3,55E-05
SNX10	2,02E-09	0,376896593	0,258	0,109	3,82E-05
VAMP3	2,11E-09	0,338581984	0,309	0,142	4,00E-05
GALC	2,13E-09	0,351209247	0,264	0,11	4,03E-05
FUCA2	2,14E-09	0,342881946	0,253	0,104	4,06E-05
SLC11A2	2,33E-09	0,41949709	0,281	0,122	4,41E-05
CACUL1	2,44E-09	0,361212389	0,315	0,144	4,62E-05
BST2	3,67E-09	0,359079442	0,36	0,175	6,96E-05
IQGAP2	5,36E-09	0,310248243	0,433	0,222	0,000101494
DNAJC5	5,76E-09	0,323157592	0,27	0,118	0,000109187
ITPR2	6,04E-09	0,450976599	0,365	0,186	0,000114479
AHR	6,46E-09	0,439349031	0,365	0,181	0,00012242
LGALS3	6,57E-09	0,563850597	0,421	0,236	0,000124511
P2RX7	7,48E-09	0,384995735	0,258	0,11	0,000141744
CIAO2A	8,90E-09	0,254665151	0,281	0,123	0,000168635
BCAP31	1,26E-08	0,48154459	0,393	0,214	0,000238902
TMED9	1,35E-08	0,349934893	0,315	0,151	0,000255884
CLTC	1,90E-08	0,377671856	0,522	0,315	0,000360654
SERPINB6	1,95E-08	0,445905478	0,41	0,218	0,000370017
GNB2	2,18E-08	0,287592302	0,393	0,203	0,000412499
CORO1B	2,36E-08	0,334394099	0,309	0,147	0,000446635
TXN2	2,44E-08	0,289705144	0,298	0,139	0,000462667
PLEKHB2	2,75E-08	0,384705938	0,461	0,264	0,000520187
COMT	2,78E-08	0,290044007	0,287	0,133	0,000526344
PRDX1	3,18E-08	0,319408446	0,466	0,267	0,000603413
CANX	3,27E-08	0,34329583	0,663	0,434	0,000619125
POU2F2	3,38E-08	0,329887858	0,433	0,237	0,000641149
TNRC18P2	3,49E-08	0,280373964	0,258	0,115	0,000661946

ALCAM	3,79E-08	0,311377351	0,281	0,131	0,000717615
ATP6V1A	3,79E-08	0,336428324	0,36	0,187	0,00071878
TXNIP	4,40E-08	0,390233308	0,652	0,462	0,000834738
PLCB2	5,06E-08	0,259100219	0,326	0,153	0,000958686
CDKN1A	5,37E-08	0,255507939	0,298	0,141	0,001017062
HOOK3	5,51E-08	0,347830108	0,545	0,329	0,001044585
TGFBR1	5,64E-08	0,344988477	0,303	0,146	0,001068438
PSMD2	6,17E-08	0,275165372	0,393	0,207	0,001169937
ARPC5	6,31E-08	0,296460707	0,584	0,372	0,001195551
ALDH3B1	6,67E-08	0,304996829	0,27	0,124	0,001263425
ITGB2	6,92E-08	0,250427191	0,663	0,432	0,00131058
ATP6V0D1	6,95E-08	0,30100051	0,275	0,127	0,001317877
PTTG1IP	8,30E-08	0,290214286	0,449	0,245	0,001572098
SPOP	8,32E-08	0,326818408	0,399	0,215	0,001576969
NFIC	8,59E-08	0,270351336	0,517	0,293	0,001627266
C6orf62	8,90E-08	0,362726181	0,478	0,279	0,001685688
JAML	9,46E-08	0,274383504	0,281	0,129	0,001792731
NCOR2	1,08E-07	0,298376093	0,32	0,159	0,002042067
YWHAE	1,13E-07	0,274327173	0,466	0,264	0,002134983
SLC4A7	1,28E-07	0,323601527	0,393	0,214	0,002433851
IFI16	1,62E-07	0,288636799	0,511	0,301	0,003071553
VOPP1	1,75E-07	0,286376883	0,438	0,252	0,003320501
CAT	1,75E-07	0,268031613	0,315	0,156	0,003322356
BRD8	1,75E-07	0,308690493	0,511	0,299	0,003322689
CTSC	1,95E-07	0,270342702	0,758	0,559	0,003694566
UCP2	1,95E-07	0,42565605	0,596	0,395	0,003696176
TPI1	2,25E-07	0,26579348	0,506	0,299	0,004260545
LGALS1	2,79E-07	0,28734745	0,567	0,357	0,005282003
CNPY3	2,96E-07	0,27626626	0,36	0,191	0,005607857
SPG21	3,30E-07	0,277483809	0,27	0,127	0,006246721
HCLS1	4,29E-07	0,258938041	0,68	0,447	0,008124803
THBD	4,42E-07	0,332104335	0,253	0,121	0,00837117
SPP1	4,73E-07	0,537291575	0,298	0,153	0,00895466
HTRA1	4,76E-07	0,426645862	0,258	0,126	0,009029567
TMEM14C	4,86E-07	0,251988954	0,287	0,143	0,009210208
ARPC3	4,87E-07	0,379997522	0,596	0,426	0,009219899
CHMP1B	5,58E-07	0,419535815	0,264	0,131	0,010573715
MAP3K3	7,21E-07	0,291900246	0,298	0,15	0,013666637
ATP6AP2	7,50E-07	0,311481201	0,506	0,321	0,014220616
CREM	1,01E-06	0,267149072	0,287	0,142	0,01922542
MLEC	1,41E-06	0,250845486	0,303	0,157	0,026796027
ATF6	1,43E-06	0,346160658	0,315	0,169	0,027192072
RTN3	1,67E-06	0,265865375	0,365	0,202	0,031582759
CALR	1,84E-06	0,336460973	0,579	0,387	0,034942006
ARHGEF2	2,23E-06	0,332640503	0,298	0,157	0,04223351
CAPZB	2,32E-06	0,289258796	0,674	0,491	0,043963393
RAB8A	2,55E-06	0,338032195	0,281	0,142	0,048326928
PBRM1	2,79E-06	0,323048246	0,32	0,177	0,052951937
WSB1	3,19E-06	0,261070251	0,59	0,382	0,060423374
GDI2	3,52E-06	0,279697017	0,478	0,299	0,066705473
GSTO1	3,80E-06	0,314610812	0,343	0,195	0,072050837
SUSD6	5,13E-06	0,270165936	0,281	0,15	0,097244804
SOCS3	5,14E-06	0,330934026	0,348	0,198	0,097345875
STAT2	5,22E-06	0,2783954	0,281	0,148	0,09890674
APPL1	5,60E-06	0,316364009	0,27	0,142	0,106177968
MED13L	8,25E-06	0,27058768	0,36	0,205	0,156284499

PLIN3	8,48E-06	0,338911867	0,298	0,166	0,160603971
FOXN3	9,79E-06	0,269825386	0,539	0,361	0,185527494
RIPOR1	1,00E-05	0,262161544	0,258	0,137	0,190369838
WASF2	1,04E-05	0,25109566	0,567	0,371	0,197377676
GNAI3	1,23E-05	0,348766718	0,303	0,17	0,233993149
PEBP1	1,24E-05	0,334077567	0,607	0,407	0,235609376
RNU4ATAC	1,60E-05	0,338340934	0,298	0,168	0,302565073
TGOLN2	1,66E-05	0,292757535	0,612	0,42	0,313763398
ASPH	1,69E-05	0,256473898	0,303	0,17	0,320706287
PRSS8	1,85E-05	0,30523401	0,787	0,639	0,351500437
HDLBP	1,94E-05	0,289000166	0,376	0,229	0,368238141
SLC31A1	3,00E-05	0,262333672	0,326	0,19	0,568934684
CD81	3,02E-05	0,354322835	0,674	0,521	0,572299071
ARPC1B	3,96E-05	0,25882332	0,551	0,378	0,749719955
HM13	4,29E-05	0,257064822	0,309	0,179	0,813555668
TOP1	8,95E-05	0,305254845	0,292	0,172	1
ROCK1	9,13E-05	0,278789288	0,41	0,264	1
SIGLEC10	0,000154482	0,309733816	0,315	0,194	1
MIDN	0,004140072	0,296990279	0,365	0,259	1

Cluster 6 marker genes

gene	p_val	avg_logFC	pct.1	pct.2	p_val_adj
S100A8	1,25E-271	3,445832089	0,783	0,046	2,38E-267
S100A9	4,58E-248	3,441412618	0,823	0,063	8,67E-244
FCN1	1,18E-174	2,378743203	0,794	0,097	2,23E-170
SERPINA1	1,07E-165	2,324698928	0,834	0,123	2,03E-161
S100A12	1,17E-157	1,736810434	0,32	0,007	2,21E-153
CFP	7,65E-150	1,604355684	0,52	0,037	1,45E-145
FPR1	5,97E-149	1,677782337	0,566	0,047	1,13E-144
AIF1	1,90E-118	1,785370701	0,743	0,126	3,61E-114
CSTA	4,27E-115	1,282310372	0,44	0,036	8,09E-111
CYBB	1,07E-111	1,726103267	0,754	0,139	2,03E-107
LST1	2,51E-104	1,713105291	0,611	0,092	4,76E-100
LYZ	2,90E-96	2,264027441	0,834	0,235	5,49E-92
SLC11A1	1,77E-93	1,321196075	0,429	0,044	3,36E-89
TYMP	1,91E-92	1,710103541	0,737	0,173	3,61E-88
NCF2	1,17E-88	1,364609547	0,514	0,072	2,21E-84
MNDA	4,98E-88	1,585400457	0,474	0,061	9,44E-84
IL1B	5,69E-86	1,691508984	0,474	0,062	1,08E-81
MPEG1	8,93E-84	1,371689523	0,594	0,103	1,69E-79
VCAN	5,12E-83	1,664038743	0,714	0,166	9,71E-79
CTSS	1,10E-82	1,877176847	0,874	0,366	2,08E-78
PRAM1	3,33E-82	0,803828202	0,251	0,014	6,31E-78
CSF3R	2,54E-80	1,565339878	0,411	0,049	4,82E-76
SECTM1	2,46E-76	1,169294745	0,411	0,051	4,67E-72
HCK	5,59E-74	1,540060208	0,537	0,1	1,06E-69
LILRB2	8,11E-73	1,516227459	0,543	0,101	1,54E-68
IFI30	2,78E-67	1,495914215	0,909	0,434	5,27E-63
SPI1	4,24E-66	1,346340803	0,634	0,151	8,04E-62
PSAP	2,00E-64	1,428341175	0,914	0,54	3,79E-60
TYROBP	5,38E-64	1,409408089	0,891	0,435	1,02E-59
CLEC12A	1,61E-63	0,92705515	0,291	0,029	3,06E-59
CD14	3,70E-59	1,36409745	0,589	0,138	7,01E-55
CXCL8	5,32E-59	1,582428949	0,406	0,066	1,01E-54
IER3	1,66E-58	1,343051899	0,6	0,155	3,14E-54
FCER1G	3,83E-57	1,35067045	0,806	0,308	7,25E-53
FTL	9,35E-57	1,05502379	0,977	0,737	1,77E-52
SAT1	5,61E-56	1,380621878	0,806	0,344	1,06E-51
HK3	8,89E-56	0,781169749	0,28	0,031	1,68E-51
BCL2A1	7,90E-55	1,1040852	0,383	0,062	1,50E-50
CD68	8,41E-55	0,978842526	0,68	0,193	1,59E-50
COTL1	5,28E-53	1,343064422	0,834	0,42	1,00E-48
MS4A6A	2,48E-52	0,98847774	0,52	0,113	4,70E-48
FTH1	8,27E-52	1,178080509	0,971	0,807	1,57E-47
NAMPT	1,12E-51	1,422895238	0,606	0,181	2,13E-47
CFD	4,88E-51	1,111094298	0,389	0,069	9,25E-47
TGFBI	2,43E-50	1,229570244	0,537	0,133	4,60E-46
CST3	5,88E-50	1,076144138	0,851	0,399	1,11E-45
FGR	7,44E-49	1,219196498	0,543	0,143	1,41E-44
G0S2	1,62E-48	1,185618026	0,269	0,033	3,07E-44
DDR2	2,08E-47	1,289212878	0,48	0,119	3,94E-43
TNFSF13B	5,93E-45	1,112789054	0,446	0,101	1,12E-40
HBEGF	5,91E-43	1,207868455	0,383	0,079	1,12E-38
ACTB	6,27E-43	0,754939962	1	0,976	1,19E-38
PILRA	7,74E-43	0,858697153	0,32	0,055	1,47E-38

TNFAIP2	1,13E-42	1,154516511	0,486	0,128	2,13E-38
SLC7A7	4,35E-41	0,77355792	0,297	0,049	8,25E-37
C5AR1	6,31E-41	1,24370664	0,537	0,161	1,20E-36
RAB31	1,25E-40	1,063889273	0,537	0,159	2,36E-36
MAFB	8,00E-40	0,981159426	0,531	0,148	1,52E-35
DMXL2	6,38E-39	0,835052178	0,366	0,077	1,21E-34
RGS2	9,12E-39	1,159265012	0,423	0,103	1,73E-34
CPVL	2,50E-38	0,786172314	0,389	0,084	4,74E-34
CLEC7A	3,90E-38	1,094681537	0,434	0,113	7,40E-34
CXCL2	7,04E-38	1,113701442	0,343	0,07	1,33E-33
PLBD1	1,49E-37	0,904501083	0,309	0,058	2,83E-33
GRN	7,84E-37	1,009670047	0,754	0,367	1,49E-32
HLA-DRA	9,02E-37	0,686284885	0,903	0,545	1,71E-32
FGL2	1,11E-36	1,098194252	0,594	0,22	2,11E-32
GPX1	1,63E-36	1,034876147	0,68	0,292	3,09E-32
GCA	3,45E-36	0,937576062	0,32	0,065	6,54E-32
LILRB3	3,52E-36	0,945674616	0,434	0,114	6,66E-32
JAML	2,98E-34	0,932256745	0,44	0,12	5,64E-30
WARS	6,12E-34	1,243069957	0,411	0,116	1,16E-29
ZNF385A	7,28E-33	0,914923275	0,411	0,111	1,38E-28
CEBPD	9,63E-33	0,965162936	0,429	0,119	1,83E-28
LYN	1,79E-32	1,117206155	0,451	0,138	3,40E-28
TNFRSF1B	2,15E-32	1,016690457	0,674	0,316	4,07E-28
S100A4	2,45E-32	0,861420421	0,914	0,679	4,64E-28
CD36	3,56E-32	0,907261099	0,366	0,091	6,75E-28
VSIR	5,77E-32	0,937511031	0,457	0,142	1,09E-27
GABARAP	1,55E-31	0,902465325	0,829	0,533	2,95E-27
GNAI2	2,28E-31	0,835421391	0,874	0,647	4,32E-27
PLAUR	3,46E-31	1,102890437	0,457	0,145	6,57E-27
NFKBIA	3,48E-31	1,227859497	0,651	0,309	6,60E-27
AP1S2	5,91E-31	0,98837795	0,486	0,166	1,12E-26
SRGN	1,36E-30	0,837683181	0,92	0,716	2,59E-26
CD74	2,28E-30	0,435675223	1	0,869	4,32E-26
SOD2	2,63E-30	1,071942559	0,537	0,209	4,99E-26
TLR2	1,24E-29	0,96196226	0,291	0,064	2,34E-25
C1orf162	1,24E-29	1,089429648	0,491	0,171	2,34E-25
FOS	3,41E-29	0,809275872	0,749	0,366	6,46E-25
PGD	3,79E-29	0,885715287	0,326	0,08	7,18E-25
ZFP36	3,81E-29	0,941295944	0,6	0,255	7,22E-25
STXBP2	6,69E-29	0,906693552	0,383	0,109	1,27E-24
RXRA	1,01E-28	0,787525982	0,366	0,099	1,91E-24
CSF1R	4,02E-28	0,811908626	0,406	0,116	7,62E-24
LRRC25	4,28E-28	0,942671952	0,326	0,083	8,12E-24
ITGAX	3,11E-27	0,958280552	0,389	0,112	5,89E-23
VMP1	3,69E-27	0,81528824	0,497	0,176	7,00E-23
VASP	4,30E-27	1,008185569	0,491	0,189	8,16E-23
CNPY3	1,04E-26	0,876773513	0,497	0,183	1,97E-22
UNC93B1	1,04E-26	0,878962903	0,377	0,111	1,97E-22
NFKBIZ	2,87E-26	0,94314936	0,509	0,191	5,45E-22
PYCARD	3,09E-26	0,883564288	0,457	0,159	5,85E-22
SRRM4	1,12E-25	0,932410697	0,32	0,084	2,13E-21
ATP6V0C	1,33E-25	0,824000141	0,783	0,475	2,51E-21
OSCAR	1,60E-25	0,676880223	0,257	0,056	3,04E-21
S100A11	1,97E-25	0,799420488	0,823	0,583	3,73E-21
FKBP1A	2,23E-25	0,702397298	0,777	0,447	4,22E-21
PLXNC1	2,65E-25	0,712959091	0,314	0,082	5,02E-21

ANPEP	2,83E-25	0,800758235	0,286	0,069	5,35E-21
ARRB2	3,58E-25	0,868713488	0,6	0,273	6,79E-21
ALOX5	4,05E-25	0,740457812	0,354	0,099	7,68E-21
GLUL	7,84E-25	0,9782994	0,589	0,277	1,49E-20
ATP6V0B	8,53E-25	0,849902149	0,583	0,253	1,62E-20
HLA-DRB1	8,56E-25	0,433778546	0,846	0,537	1,62E-20
PTGS2	1,50E-24	0,731052323	0,303	0,078	2,84E-20
FCGRT	1,82E-24	0,769383202	0,623	0,281	3,44E-20
SLC16A3	4,85E-24	0,711544249	0,32	0,087	9,18E-20
CTSB	6,86E-24	0,356609304	0,817	0,498	1,30E-19
RHOG	4,16E-23	0,77914751	0,503	0,206	7,89E-19
JUNB	4,32E-23	0,743157749	0,749	0,434	8,18E-19
MYO1F	5,20E-23	0,785485971	0,52	0,217	9,86E-19
EGR1	6,49E-23	0,823539187	0,571	0,251	1,23E-18
ADAP2	9,55E-23	0,635028496	0,337	0,098	1,81E-18
POU2F2	1,45E-22	0,982940654	0,526	0,232	2,75E-18
CD300E	2,81E-22	0,650129119	0,366	0,114	5,32E-18
OAZ1	4,53E-22	0,73656722	0,794	0,603	8,58E-18
ATP6V0D1	9,74E-22	0,686917139	0,371	0,122	1,85E-17
LY86	1,05E-21	0,640450151	0,28	0,074	2,00E-17
SYK	2,87E-21	0,701332271	0,457	0,175	5,44E-17
MS4A4A	3,59E-21	0,81430755	0,474	0,191	6,80E-17
SKAP2	3,88E-21	0,701620025	0,491	0,2	7,34E-17
CYBA	1,58E-20	0,776216801	0,691	0,435	3,00E-16
EFHD2	2,65E-20	0,714857366	0,589	0,276	5,02E-16
TBXAS1	2,79E-20	0,643992097	0,274	0,076	5,29E-16
ACSL1	9,13E-20	0,764766383	0,251	0,067	1,73E-15
CD93	9,54E-20	0,780373425	0,36	0,124	1,81E-15
TALDO1	1,22E-19	0,857528287	0,429	0,174	2,30E-15
CEBPB	1,40E-19	0,720800141	0,36	0,125	2,65E-15
PTPRE	1,78E-19	0,746273151	0,429	0,171	3,37E-15
LILRB1	2,80E-19	0,603376078	0,331	0,107	5,31E-15
LGALS9	3,12E-19	0,610746646	0,303	0,093	5,91E-15
IRAK3	6,59E-19	0,596304044	0,257	0,072	1,25E-14
AGTRAP	8,71E-19	0,757576795	0,28	0,084	1,65E-14
HLA-DPA1	1,35E-18	0,401881106	0,84	0,612	2,56E-14
ATP6V1B2	1,45E-18	0,91573362	0,389	0,153	2,76E-14
GRINA	1,54E-18	0,721654346	0,349	0,125	2,93E-14
ARPC1B	1,56E-18	0,738863593	0,634	0,373	2,95E-14
ARPC4	1,71E-18	0,750787955	0,651	0,359	3,25E-14
AOAH	2,15E-18	0,503022545	0,337	0,11	4,07E-14
TIMP1	4,38E-18	0,971029341	0,766	0,508	8,30E-14
CCR1	4,66E-18	0,60920772	0,28	0,084	8,84E-14
LAPTM5	6,36E-18	0,555733946	0,834	0,607	1,20E-13
NEAT1	8,49E-18	0,733900424	0,646	0,401	1,61E-13
MARCKS	1,09E-17	0,664108426	0,497	0,222	2,06E-13
FCGR3A	1,23E-17	0,76611524	0,274	0,083	2,33E-13
CTSH	1,75E-17	0,654173233	0,366	0,136	3,31E-13
BRI3	2,87E-17	0,709164785	0,434	0,188	5,45E-13
ZEB2	4,58E-17	0,695258131	0,434	0,188	8,68E-13
LAT2	5,11E-17	0,521690019	0,257	0,075	9,69E-13
NAGA	5,95E-17	0,660047746	0,28	0,088	1,13E-12
HLA-DMB	1,75E-16	0,548715652	0,417	0,165	3,32E-12
MYO1G	2,50E-16	0,701090425	0,4	0,162	4,73E-12
TMEM176B	3,19E-16	0,621618783	0,343	0,129	6,04E-12
ARPC5	3,88E-16	0,709560861	0,611	0,37	7,35E-12

SCIMP	4,27E-16	0,624943701	0,251	0,077	8,08E-12
FAM49A	4,45E-16	0,627598664	0,257	0,08	8,44E-12
RAC1	4,72E-16	0,556819992	0,766	0,493	8,94E-12
THEMIS2	5,49E-16	0,656642548	0,263	0,084	1,04E-11
CASP1	5,62E-16	0,638806508	0,343	0,13	1,07E-11
SH3BGRL3	6,33E-16	0,490068779	0,943	0,847	1,20E-11
S100A6	7,80E-16	0,49398569	0,846	0,672	1,48E-11
CMTM6	8,10E-16	0,640771298	0,589	0,338	1,53E-11
CXCL16	8,34E-16	0,670562186	0,451	0,194	1,58E-11
LTA4H	8,89E-16	0,948524175	0,389	0,171	1,68E-11
NPC2	1,15E-15	0,587253949	0,583	0,311	2,18E-11
BASP1	1,37E-15	0,789061823	0,286	0,098	2,60E-11
mrt-01	1,38E-15	0,481940255	0,274	0,088	2,61E-11
CPPED1	1,73E-15	0,61865628	0,423	0,183	3,29E-11
CTSD	4,19E-15	0,422698597	0,64	0,372	7,94E-11
AP2S1	4,41E-15	0,612522088	0,469	0,222	8,37E-11
GRB2	4,62E-15	0,725287993	0,566	0,322	8,76E-11
TMSB10	4,65E-15	0,432167849	0,937	0,865	8,81E-11
KCTD12	5,25E-15	0,561542236	0,434	0,193	9,96E-11
SULF2	7,79E-15	0,580139514	0,257	0,083	1,48E-10
CD163	8,23E-15	0,673987081	0,297	0,104	1,56E-10
EHBP1L1	9,13E-15	0,597627186	0,371	0,156	1,73E-10
ZYX	1,11E-14	0,645632696	0,64	0,395	2,10E-10
DUSP2	1,31E-14	0,73744046	0,269	0,092	2,48E-10
ZFP36L1	1,32E-14	0,61171712	0,749	0,55	2,51E-10
RNASE6	1,47E-14	0,522025951	0,257	0,084	2,79E-10
PECAM1	1,49E-14	0,520016886	0,566	0,294	2,82E-10
PLIN3	2,29E-14	0,646078812	0,377	0,162	4,35E-10
PRKCD	2,87E-14	0,633682528	0,251	0,084	5,43E-10
DUSP6	3,61E-14	0,581759582	0,36	0,15	6,84E-10
ASAH1	5,84E-14	0,5975173	0,446	0,211	1,11E-09
SAMHD1	7,94E-14	0,590635905	0,286	0,103	1,50E-09
CARD16	9,02E-14	0,540186849	0,28	0,101	1,71E-09
SDCBP	9,84E-14	0,568966019	0,589	0,322	1,86E-09
PFN1	1,06E-13	0,424821136	0,931	0,869	2,01E-09
EVI2B	1,69E-13	0,587669705	0,594	0,352	3,20E-09
DUSP1	1,75E-13	0,578449834	0,457	0,219	3,31E-09
CD4	2,12E-13	0,620486028	0,606	0,37	4,01E-09
RGS19	2,77E-13	0,546591488	0,343	0,141	5,26E-09
RBM47	2,97E-13	0,650718267	0,366	0,16	5,62E-09
KLF4	3,47E-13	0,502130205	0,286	0,106	6,57E-09
RIN2	3,48E-13	0,499734391	0,263	0,092	6,60E-09
PRSS8	4,42E-13	0,479044813	0,806	0,638	8,37E-09
HLA-DRB5	4,56E-13	0,60502253	0,36	0,157	8,65E-09
NFAM1	5,96E-13	0,613389	0,389	0,179	1,13E-08
SIRPA	6,47E-13	0,535220526	0,297	0,116	1,23E-08
GNS	1,03E-12	0,584058817	0,463	0,236	1,95E-08
FAM49B	1,06E-12	0,577990775	0,486	0,252	2,01E-08
ALDH2	1,09E-12	0,602680184	0,286	0,113	2,06E-08
ADGRE2	1,86E-12	0,503032854	0,309	0,123	3,52E-08
IL17RA	1,99E-12	0,708463698	0,474	0,258	3,78E-08
UQCRC1	2,80E-12	0,495957106	0,32	0,133	5,31E-08
PLEK	2,93E-12	0,742791671	0,434	0,222	5,55E-08
HLA-DPB1	3,85E-12	0,310343304	0,691	0,455	7,29E-08
RNF130	3,95E-12	0,543669348	0,349	0,155	7,48E-08
SH3BP2	5,32E-12	0,61792359	0,457	0,24	1,01E-07

HLA-DMA	5,47E-12	0,451717815	0,411	0,19	1,04E-07
FOSL2	8,05E-12	0,531254073	0,251	0,092	1,53E-07
LSP1	8,10E-12	0,538192647	0,594	0,361	1,53E-07
EMP3	9,33E-12	0,55229861	0,674	0,44	1,77E-07
ATP2B1	1,08E-11	0,638346777	0,52	0,293	2,05E-07
RNF149	1,23E-11	0,62699519	0,411	0,206	2,32E-07
PIK3AP1	1,40E-11	0,494627629	0,297	0,119	2,65E-07
TUBA1A	1,45E-11	0,83668247	0,411	0,221	2,75E-07
DBNL	1,46E-11	0,601241898	0,337	0,152	2,77E-07
SHKBP1	1,53E-11	0,607713559	0,366	0,173	2,90E-07
MBOAT7	1,83E-11	0,509487914	0,263	0,102	3,47E-07
GSTP1	2,25E-11	0,608289293	0,491	0,283	4,26E-07
LRP1	2,28E-11	0,460032257	0,331	0,142	4,31E-07
STX10	2,93E-11	0,498779463	0,251	0,096	5,55E-07
COLGALT1	3,18E-11	0,512791028	0,28	0,116	6,02E-07
IRS2	3,87E-11	0,457941563	0,257	0,098	7,33E-07
ALDOA	4,39E-11	0,6089316	0,6	0,413	8,31E-07
MEGF9	5,59E-11	0,646975957	0,366	0,179	1,06E-06
UBE2R2	5,70E-11	0,715599313	0,383	0,191	1,08E-06
ADA2	6,09E-11	0,615757143	0,423	0,224	1,15E-06
MYD88	6,42E-11	0,501676433	0,297	0,127	1,22E-06
PLXNB2	7,37E-11	0,491124573	0,291	0,125	1,40E-06
GRK3	7,86E-11	0,488947089	0,263	0,105	1,49E-06
IL10RB	1,14E-10	0,551937303	0,4	0,207	2,16E-06
CFL1	1,32E-10	0,369973947	0,931	0,872	2,50E-06
GRK2	1,42E-10	0,589097098	0,577	0,385	2,69E-06
LCP1	1,61E-10	0,331891127	0,874	0,694	3,04E-06
RASSF2	2,03E-10	0,514257128	0,303	0,134	3,85E-06
MCL1	3,40E-10	0,635301053	0,6	0,407	6,45E-06
PABPC4	3,91E-10	0,542665992	0,526	0,324	7,41E-06
ATP1B3	4,08E-10	0,609769177	0,48	0,288	7,74E-06
MYL6	4,24E-10	0,40410947	0,846	0,715	8,04E-06
HPSE	4,58E-10	0,579478522	0,326	0,155	8,67E-06
PABPC1	5,07E-10	0,313479095	0,897	0,823	9,62E-06
MAN2B1	6,87E-10	0,543191645	0,331	0,159	1,30E-05
SOCS3	7,96E-10	0,514660897	0,383	0,196	1,51E-05
TTYH3	8,34E-10	0,459743728	0,274	0,117	1,58E-05
IFNGR2	8,71E-10	0,534877542	0,291	0,134	1,65E-05
RNASET2	9,60E-10	0,45754379	0,554	0,35	1,82E-05
CAPNS1	1,03E-09	0,490945348	0,623	0,41	1,94E-05
CD83	1,21E-09	0,381110386	0,263	0,109	2,29E-05
GADD45B	1,22E-09	0,483193922	0,366	0,183	2,31E-05
GLIPR2	1,24E-09	0,513669295	0,36	0,179	2,35E-05
APLP2	1,63E-09	0,499186755	0,611	0,391	3,09E-05
ANXA5	1,65E-09	0,440688992	0,629	0,417	3,13E-05
H2AFY	1,87E-09	0,440545443	0,509	0,297	3,55E-05
ITGB2	1,99E-09	0,41342131	0,634	0,434	3,77E-05
MGAT1	2,05E-09	0,494093504	0,371	0,194	3,89E-05
CDKN1A	2,52E-09	0,529526978	0,303	0,141	4,77E-05
TPI1	2,79E-09	0,547471713	0,486	0,3	5,29E-05
GPCPD1	3,17E-09	0,585162441	0,36	0,186	6,01E-05
RNU4ATAC	3,27E-09	0,50291229	0,337	0,166	6,19E-05
ITGAM	4,00E-09	0,610089118	0,269	0,12	7,58E-05
GPSM3	4,34E-09	0,440853018	0,497	0,296	8,23E-05
GSTO1	4,99E-09	0,554009323	0,366	0,193	9,45E-05
RTN4	6,30E-09	0,439007575	0,64	0,454	0,00011943

ACTR2	7,00E-09	0,478346623	0,674	0,515	0,000132642
ARPC2	7,64E-09	0,343792391	0,863	0,772	0,000144811
TCIRG1	8,49E-09	0,571741248	0,383	0,214	0,000160888
PRR13	8,53E-09	0,490279141	0,497	0,312	0,00016162
GMFG	8,63E-09	0,379698568	0,56	0,342	0,000163552
MAP3K3	9,43E-09	0,581111106	0,303	0,15	0,000178668
METTL7A	9,63E-09	0,469356112	0,411	0,233	0,000182522
PKM	1,02E-08	0,453551249	0,726	0,597	0,000192618
FYB1	1,14E-08	0,350498548	0,771	0,565	0,000216505
PPT1	1,51E-08	0,462597978	0,463	0,275	0,000285347
VAMP8	1,68E-08	0,593076036	0,354	0,192	0,000318381
GLIPR1L2	1,72E-08	0,562604697	0,377	0,207	0,000325018
CRTAP	1,75E-08	0,417786564	0,537	0,342	0,0003319
NAGK	1,76E-08	0,431859437	0,36	0,192	0,000334094
PTAFR	2,14E-08	0,49255417	0,331	0,171	0,000406302
LFNG	2,38E-08	0,544021559	0,263	0,12	0,000450718
RHOA	2,82E-08	0,384747541	0,726	0,595	0,000535124
NR4A1	2,86E-08	0,496592551	0,257	0,118	0,000542543
TGOLN2	3,73E-08	0,406714279	0,594	0,421	0,00070743
TPP1	4,24E-08	0,378261829	0,606	0,409	0,000804083
CDC42	4,32E-08	0,437605136	0,68	0,537	0,000818005
PPP1R15A	4,91E-08	0,500477464	0,303	0,154	0,000929523
HCLS1	5,00E-08	0,415625502	0,634	0,45	0,000946603
ZFAND5	6,00E-08	0,513453446	0,429	0,255	0,00113693
BLOC1S1	6,51E-08	0,519378824	0,411	0,238	0,001232948
SIGLEC10	6,51E-08	0,600994268	0,343	0,192	0,001234341
NAIP	8,21E-08	0,474621568	0,28	0,138	0,001554849
IER2	8,32E-08	0,483937929	0,371	0,21	0,001576532
METRNL	8,62E-08	0,358608019	0,274	0,13	0,00163292
PFDN5	9,28E-08	0,399432614	0,691	0,538	0,001758938
BRD8	9,39E-08	0,332579577	0,497	0,3	0,001778778
LAIR1	1,21E-07	0,463579511	0,331	0,176	0,002296282
ETV6	1,45E-07	0,484015524	0,32	0,169	0,002751932
CYFIP1	2,04E-07	0,485816988	0,349	0,197	0,0038635
PLCB2	2,10E-07	0,453429792	0,303	0,154	0,003988564
LGALS1	2,14E-07	0,363242333	0,554	0,358	0,004061699
CUX1	2,16E-07	0,456468588	0,343	0,192	0,004100107
HSBP1	2,48E-07	0,442604769	0,274	0,139	0,004702272
MYADM	2,50E-07	0,425890852	0,566	0,38	0,004734641
KIAA0930	2,52E-07	0,416372317	0,297	0,154	0,004771159
DICER1	2,70E-07	0,411280621	0,36	0,199	0,005121646
RTN3	2,89E-07	0,450315723	0,354	0,202	0,005479493
LRRFIP1	3,18E-07	0,355980874	0,783	0,657	0,006025492
PARVG	3,73E-07	0,326486076	0,28	0,136	0,007062859
ALDH3B1	3,90E-07	0,432806857	0,257	0,125	0,007394617
FOSB	4,46E-07	0,346975742	0,657	0,487	0,008451861
JPT1	5,11E-07	0,478754183	0,257	0,125	0,009683385
TNFSF10	5,94E-07	0,409557338	0,32	0,172	0,011251764
PRKACA	6,91E-07	0,481339945	0,286	0,151	0,013093475
WAS	7,28E-07	0,446773687	0,463	0,297	0,013791102
ARPC3	7,28E-07	0,389977422	0,6	0,425	0,013798174
WSB1	8,29E-07	0,420193263	0,537	0,386	0,01571124
SERPINB1	8,33E-07	0,504491498	0,383	0,236	0,015786311
UCP2	8,90E-07	0,424159844	0,554	0,397	0,016860391
MEF2C	9,07E-07	0,368310216	0,274	0,142	0,01718729
IMPDH1	1,05E-06	0,396080065	0,263	0,134	0,019821135

SERP1	1,25E-06	0,486645025	0,503	0,351	0,023666491
DPYSL2	1,98E-06	0,407121574	0,36	0,215	0,037540631
LAMTOR1	2,10E-06	0,422508832	0,269	0,14	0,039730289
GNB2	2,16E-06	0,437738215	0,349	0,205	0,040991818
RNF13	2,30E-06	0,349136882	0,326	0,183	0,043617001
PSMB3	2,54E-06	0,399050905	0,354	0,21	0,048073511
BAG6	2,82E-06	0,338751114	0,349	0,201	0,053497038
PKN1	3,48E-06	0,390365068	0,291	0,16	0,065919144
GAS7	4,56E-06	0,541678886	0,337	0,204	0,086447087
MOB3A	5,09E-06	0,514415623	0,337	0,196	0,096380595
ENO1	5,29E-06	0,449129144	0,629	0,486	0,100286092
CAPG	5,78E-06	0,370694628	0,291	0,16	0,109613058
MSN	6,31E-06	0,349384388	0,629	0,488	0,119495794
ADGRE5	6,46E-06	0,36548949	0,4	0,247	0,122481469
QKI	6,80E-06	0,420516575	0,411	0,277	0,1288028
ANXA2	7,45E-06	0,268672628	0,577	0,4	0,141088008
MAP3K20	8,05E-06	0,480865487	0,251	0,139	0,152465641
PRKCB	8,12E-06	0,390099785	0,514	0,365	0,153850322
CCDC69	1,03E-05	0,394825279	0,4	0,254	0,194438754
RHOQ	1,20E-05	0,29749887	0,291	0,163	0,227818039
ATP6V1A	1,22E-05	0,280784318	0,326	0,189	0,231240517
SF3B5	1,34E-05	0,314302878	0,309	0,178	0,254230816
GAPDH	1,37E-05	0,372663067	0,817	0,745	0,260395998
LAMTOR4	1,50E-05	0,322845684	0,303	0,174	0,284917208
IL6R	1,52E-05	0,336673716	0,354	0,213	0,288982472
IL13RA1	1,64E-05	0,454254901	0,303	0,183	0,310938563
AP1B1	1,69E-05	0,305143606	0,269	0,145	0,320409309
ZNF106	1,85E-05	0,409899597	0,423	0,293	0,349934681
SERF2	1,94E-05	0,261167563	0,8	0,701	0,368466872
RNF181	2,04E-05	0,323930123	0,269	0,15	0,386355159
DDX21	2,31E-05	0,312332827	0,451	0,302	0,43684561
LYST	2,55E-05	0,33039865	0,331	0,201	0,482884115
PLEKHO1	2,68E-05	0,455050712	0,257	0,143	0,50740699
LGALS3	2,69E-05	0,284390761	0,377	0,238	0,510436546
CCDC88A	2,71E-05	0,350361566	0,343	0,214	0,513172137
ATG3	2,76E-05	0,349305633	0,274	0,156	0,522183446
CEL	2,83E-05	0,278121052	0,349	0,212	0,536909774
RAB10	2,85E-05	0,31074228	0,343	0,21	0,539756918
RAB5C	3,27E-05	0,351262654	0,28	0,162	0,618975517
SYNGR2	3,37E-05	0,394900595	0,331	0,204	0,639226649
BAZ1A	3,56E-05	0,301771369	0,411	0,283	0,674569189
ATP5MPL	4,13E-05	0,342877931	0,377	0,235	0,783555328
CACUL1	5,86E-05	0,285052358	0,257	0,147	1
PICALM	6,07E-05	0,302877513	0,343	0,22	1
SLC2A3	6,34E-05	0,517229633	0,423	0,301	1
ZNF652	6,55E-05	0,282024084	0,326	0,197	1
CLIC1	6,89E-05	0,263980419	0,6	0,444	1
WASF2	7,10E-05	0,291937175	0,514	0,375	1
OS9	7,18E-05	0,420763744	0,4	0,276	1
PEA15	7,25E-05	0,329250575	0,383	0,256	1
ANP32A	0,000101054	0,34129808	0,331	0,217	1
CREG1	0,000103329	0,37243782	0,257	0,15	1
CHMP2A	0,000106247	0,286541409	0,309	0,191	1
DAZAP2	0,000118494	0,328043075	0,611	0,5	1
ATP5F1EP2	0,000150283	0,260936742	0,537	0,404	1
YBX3	0,000150662	0,294749667	0,309	0,192	1

CSK	0,000158007	0,298870161	0,371	0,25	1
JUND	0,000164192	0,342903919	0,771	0,678	1
NCOA4	0,000169881	0,34290254	0,366	0,248	1
SH3BGRL	0,000172907	0,322731578	0,469	0,344	1
IFITM2	0,000178969	0,416122294	0,874	0,818	1
ITPK1	0,000179151	0,351386997	0,257	0,155	1
CAST	0,000185181	0,254677507	0,474	0,344	1
SYAP1	0,00019926	0,394395454	0,331	0,221	1
VPS35	0,000224955	0,4633147	0,314	0,206	1
LY6E	0,000230044	0,346392473	0,446	0,325	1
BAZ2B	0,000232242	0,378484209	0,303	0,195	1
ITPRIPL2	0,000242442	0,276140496	0,303	0,193	1
TMEM167A	0,000271491	0,346994867	0,291	0,19	1
IL10RA	0,000276698	0,254091252	0,32	0,203	1
PSMD2	0,00039105	0,407759574	0,314	0,211	1
HIPK2	0,000419015	0,307559155	0,291	0,185	1
GDI2	0,000449763	0,282432487	0,423	0,302	1
CAPZB	0,000453991	0,251171923	0,611	0,495	1
PSMA6	0,000467675	0,270836828	0,32	0,21	1
CHP1	0,000470518	0,401130114	0,28	0,182	1
SLC7A5P2	0,000475024	0,412751497	0,257	0,157	1
PSMA4	0,000487629	0,255386561	0,297	0,187	1
TGFB1	0,000494822	0,282384431	0,371	0,25	1
CMIP	0,000496744	0,269448241	0,48	0,353	1
ELOB	0,000534442	0,388210744	0,44	0,328	1
MIDN	0,000684741	0,518102398	0,354	0,26	1
JARID2	0,000773567	0,376946059	0,28	0,182	1
ICAM3	0,000859829	0,381087621	0,286	0,189	1
NBEAL2	0,001029946	0,350173408	0,303	0,201	1
CAT	0,00114508	0,307099049	0,251	0,16	1
PTK2B	0,001196355	0,346642634	0,446	0,344	1
RNF141	0,001236827	0,278051314	0,263	0,168	1
JMJD1C	0,001352029	0,268651988	0,377	0,269	1
SERPINB6	0,001496757	0,422020389	0,314	0,224	1
COPE	0,001611861	0,305136243	0,377	0,279	1
TRAPPC1	0,001705049	0,310701837	0,383	0,282	1
HADHA	0,00172835	0,25323594	0,326	0,23	1
TPM3	0,002147906	0,255067589	0,709	0,653	1
NDUFB10	0,002330376	0,349454011	0,257	0,173	1
BNIP3L	0,002545073	0,313415847	0,303	0,214	1
PSMB10	0,002593301	0,270728417	0,326	0,223	1
NAAA	0,002960193	0,315044921	0,28	0,189	1
CALM2	0,003003982	0,299960114	0,583	0,504	1
UQCRC2	0,003231394	0,269786577	0,274	0,186	1
RAB1B	0,003944643	0,266748363	0,303	0,219	1
GNB4	0,005055525	0,255886181	0,263	0,181	1
HMGA1	0,005262892	0,269798301	0,291	0,203	1
STAT6	0,005835331	0,286864851	0,36	0,279	1
LMAN2	0,006671886	0,260345441	0,28	0,197	1
TSPO	0,006841705	0,30377256	0,371	0,279	1
ARL6IP4	0,007856314	0,298976582	0,331	0,251	1
TRIR	0,009956154	0,278762906	0,52	0,42	1

Cluster 7 marker genes

gene	p_val	avg_logFC	pct.1	pct.2	p_val_adj
MMP9	9,17E-156	2,61358195	0,503	0,031	1,74E-151
SPP1	2,69E-149	3,673919882	0,814	0,126	5,11E-145
APOC1	2,82E-147	2,30986683	0,569	0,047	5,35E-143
GPNMB	2,27E-107	1,766839249	0,731	0,129	4,29E-103
APOE	2,02E-102	2,37772544	0,695	0,125	3,83E-98
MMP19	2,77E-91	1,334164607	0,311	0,02	5,25E-87
FBP1	1,20E-85	1,423278487	0,491	0,064	2,28E-81
TREM2	6,01E-81	0,946414243	0,293	0,02	1,14E-76
ANPEP	3,69E-77	1,256053892	0,455	0,061	7,00E-73
LILRB4	7,78E-75	0,978073667	0,419	0,051	1,47E-70
CTSB	1,00E-74	2,075733104	0,928	0,493	1,90E-70
IL4I1	2,18E-74	0,767474967	0,251	0,015	4,13E-70
CD68	4,55E-73	1,590590129	0,737	0,191	8,63E-69
CYP27A1	3,24E-72	0,931999822	0,323	0,03	6,14E-68
GRN	1,09E-71	1,417165146	0,91	0,359	2,07E-67
CCR1	1,85E-70	0,965968562	0,491	0,074	3,51E-66
IFI30	1,15E-69	1,609621793	0,934	0,434	2,18E-65
CAPG	2,23E-69	1,356332687	0,635	0,142	4,23E-65
RNASE1	3,43E-69	1,54768053	0,754	0,199	6,49E-65
CD163	1,69E-68	1,101869359	0,533	0,091	3,21E-64
MSR1	2,70E-68	1,336685003	0,551	0,103	5,11E-64
CD14	4,88E-68	1,263984213	0,641	0,136	9,24E-64
SPI1	2,26E-67	1,158141997	0,671	0,15	4,29E-63
FTL	1,62E-65	1,863685145	0,988	0,737	3,07E-61
FABP5	1,51E-64	1,845871578	0,611	0,145	2,86E-60
MAFB	8,67E-63	1,232171544	0,629	0,144	1,64E-58
VSIG4	1,34E-62	1,083346673	0,443	0,068	2,55E-58
LHFPL2	5,19E-61	0,995911283	0,419	0,063	9,83E-57
SCD	3,33E-60	1,007946422	0,389	0,055	6,30E-56
CSTB	8,24E-60	1,926393637	0,772	0,298	1,56E-55
NR1H3	2,05E-59	0,797602309	0,269	0,025	3,88E-55
PSAP	5,42E-59	1,336794064	0,946	0,54	1,03E-54
C5AR1	8,04E-58	1,2374408	0,629	0,157	1,52E-53
TYROBP	1,13E-57	1,322997558	0,91	0,435	2,13E-53
SLC11A1	1,55E-56	0,962290307	0,359	0,049	2,93E-52
MITF	1,21E-55	0,875881839	0,365	0,052	2,28E-51
PLD3	1,45E-55	1,472429843	0,587	0,156	2,75E-51
GPX1	2,86E-55	1,296060474	0,772	0,288	5,42E-51
CSF1R	2,12E-54	0,947621317	0,533	0,11	4,02E-50
FAM20C	2,74E-53	0,742566319	0,281	0,032	5,19E-49
CTSL	2,80E-53	1,497320001	0,527	0,121	5,30E-49
TYMP	4,11E-53	1,123129598	0,659	0,178	7,79E-49
NPC2	7,85E-53	1,278622413	0,778	0,301	1,49E-48
CCDC88A	8,86E-53	1,236664877	0,659	0,197	1,68E-48
PLTP	1,39E-52	1,289839453	0,641	0,185	2,64E-48
ACP5	2,66E-52	1,176087366	0,347	0,05	5,04E-48
PKM	2,97E-52	1,124050064	0,934	0,587	5,64E-48
TMEM51	7,73E-52	0,758519914	0,287	0,034	1,46E-47
CLEC5A	2,85E-51	0,881027298	0,293	0,036	5,41E-47
FPR3	5,57E-51	0,678548488	0,329	0,045	1,05E-46
CTSD	4,33E-49	1,670173148	0,802	0,364	8,21E-45
FN1	5,18E-49	1,548646938	0,743	0,275	9,82E-45
ADAP2	1,98E-48	0,914744042	0,461	0,092	3,75E-44

ITGAX	5,28E-48	0,928829586	0,491	0,107	1,00E-43
C1QA	6,78E-48	0,863609966	0,485	0,1	1,29E-43
SLC16A3	9,19E-48	0,970943727	0,425	0,082	1,74E-43
CTSZ	9,37E-48	1,237786811	0,455	0,098	1,77E-43
TREM1	1,38E-47	0,799925125	0,263	0,031	2,62E-43
S100A11	1,93E-47	1,115364486	0,916	0,578	3,66E-43
FTH1	1,09E-46	1,339462546	0,994	0,806	2,06E-42
SLC43A3	1,42E-46	0,866802009	0,443	0,091	2,69E-42
C1QC	1,45E-46	0,855474144	0,473	0,099	2,74E-42
SIRPA	2,38E-46	0,936520837	0,473	0,107	4,51E-42
OLR1	7,81E-46	0,996866742	0,323	0,05	1,48E-41
TGFBI	9,26E-46	1,251471657	0,533	0,135	1,75E-41
MPP1	1,56E-45	1,036705475	0,431	0,09	2,96E-41
ST14	2,93E-44	0,589403077	0,281	0,038	5,55E-40
LYZ	2,50E-43	0,855250389	0,719	0,242	4,73E-39
TTYH3	3,03E-43	1,170512789	0,455	0,108	5,74E-39
ZNF385A	3,70E-43	0,913733108	0,473	0,109	7,00E-39
FCGRT	4,68E-43	0,99493919	0,743	0,276	8,87E-39
SLCO2B1	7,72E-43	0,606446634	0,335	0,054	1,46E-38
TUBA1C	1,22E-42	1,145687579	0,479	0,118	2,32E-38
AIF1	1,26E-42	0,740312497	0,557	0,138	2,38E-38
FCER1G	1,68E-42	0,966491951	0,79	0,31	3,19E-38
ABCA1	1,72E-42	1,109427739	0,437	0,099	3,26E-38
EMILIN2	2,81E-42	0,623034356	0,329	0,054	5,32E-38
PLAU	2,85E-42	1,058388125	0,311	0,05	5,41E-38
CYFIP1	1,18E-41	0,903310228	0,599	0,184	2,23E-37
CST3	1,30E-40	0,747027585	0,88	0,399	2,46E-36
GLUL	2,85E-40	1,112541056	0,713	0,272	5,40E-36
NPL	6,12E-40	0,522572791	0,269	0,039	1,16E-35
MARCKS	6,93E-40	1,103998293	0,641	0,215	1,31E-35
ACTB	9,44E-40	0,802340756	0,994	0,977	1,79E-35
GSN	1,65E-39	0,774314362	0,749	0,299	3,13E-35
PLAUR	1,71E-39	0,920139929	0,521	0,142	3,24E-35
SLAMF8	1,98E-39	0,545871362	0,281	0,042	3,74E-35
ANXA5	1,67E-38	1,040806257	0,814	0,408	3,17E-34
ATP6V0B	2,52E-38	1,007088419	0,665	0,25	4,78E-34
CTSH	2,57E-38	0,925506984	0,491	0,129	4,86E-34
LRP1	4,34E-38	1,005561288	0,497	0,134	8,23E-34
CXCL16	1,30E-37	0,957095202	0,587	0,187	2,46E-33
DMXL2	1,40E-37	0,834491553	0,371	0,077	2,65E-33
CD36	7,79E-37	1,411676836	0,389	0,091	1,48E-32
ATP6V0C	2,00E-36	0,87885561	0,868	0,471	3,79E-32
SGK1	4,82E-36	1,031371739	0,431	0,109	9,14E-32
SLC7A7	7,50E-36	0,550603077	0,293	0,05	1,42E-31
SHTN1	7,74E-36	0,665955734	0,317	0,059	1,47E-31
LGALS3	1,81E-35	1,167770026	0,605	0,227	3,44E-31
NELL1	2,24E-35	0,764445366	0,419	0,102	4,24E-31
CREG1	4,17E-35	0,837110148	0,485	0,138	7,90E-31
SDC2	4,30E-35	1,011473042	0,359	0,079	8,15E-31
CYBB	4,57E-35	0,832701852	0,533	0,152	8,66E-31
LGALS1	1,48E-34	1,112496115	0,737	0,349	2,80E-30
LRRC25	2,02E-34	0,700052503	0,371	0,082	3,83E-30
HLA-DRB1	2,35E-34	0,804570833	0,916	0,534	4,45E-30
VIM	2,79E-34	0,868906541	0,958	0,823	5,29E-30
CEBPB	1,10E-33	0,972165961	0,443	0,121	2,08E-29
EIF4EBP1	3,34E-33	0,608200209	0,311	0,061	6,32E-29

CD63	3,45E-32	1,059547376	0,766	0,398	6,53E-28
C3AR1	3,95E-32	0,635828673	0,335	0,071	7,49E-28
UNC93B1	5,33E-32	0,729133415	0,425	0,109	1,01E-27
BCAT1	7,50E-32	0,890977552	0,479	0,147	1,42E-27
ADAM9	9,15E-32	0,652063797	0,329	0,072	1,73E-27
PDXK	1,21E-31	0,753925344	0,389	0,097	2,29E-27
CORO1C	1,52E-31	0,593386365	0,341	0,076	2,87E-27
ITGAM	2,53E-31	0,689666477	0,425	0,112	4,79E-27
ALDOA	3,96E-31	1,035057753	0,749	0,405	7,51E-27
LAPTM5	1,01E-30	0,901984689	0,868	0,606	1,91E-26
HAVCR2	1,29E-30	0,509662663	0,293	0,057	2,45E-26
HSD3B7	1,60E-30	0,539387834	0,251	0,044	3,04E-26
ACTN1	5,44E-30	0,715820491	0,683	0,27	1,03E-25
C1QB	5,51E-30	0,776549936	0,461	0,134	1,04E-25
TNFAIP2	1,03E-29	0,853150885	0,449	0,131	1,94E-25
S100A10	1,62E-29	1,240403716	0,772	0,517	3,06E-25
HNMT	1,91E-29	0,59850866	0,377	0,094	3,62E-25
LGALS9	3,10E-29	0,577565708	0,365	0,09	5,87E-25
ITGB5	3,24E-29	0,68141783	0,341	0,081	6,14E-25
PLIN2	7,07E-29	1,037419354	0,353	0,09	1,34E-24
SLC37A2	1,02E-28	0,704573628	0,389	0,101	1,93E-24
ANXA2	1,39E-28	0,963708069	0,725	0,393	2,64E-24
SDCBP	1,53E-28	0,742453588	0,701	0,317	2,90E-24
LMNA	2,08E-28	0,811056007	0,665	0,282	3,94E-24
HLA-DRA	2,87E-28	0,598152769	0,904	0,546	5,44E-24
SLC18A1	3,36E-28	0,826125565	0,461	0,147	6,37E-24
HCK	3,85E-28	0,51800799	0,407	0,109	7,29E-24
GSTO1	4,21E-28	0,809420739	0,521	0,186	7,97E-24
RIN2	4,34E-28	0,739244537	0,353	0,088	8,22E-24
PLXNB2	1,02E-27	0,749409018	0,407	0,119	1,93E-23
FAM129B	1,04E-27	0,661569448	0,383	0,103	1,98E-23
H2AFY	1,46E-27	0,778680841	0,653	0,29	2,76E-23
APLP2	1,96E-27	0,865903288	0,737	0,385	3,72E-23
LGMN	2,64E-27	1,095246472	0,353	0,093	5,01E-23
RAB31	3,38E-27	0,738025709	0,491	0,163	6,41E-23
ATP6V1B2	7,52E-27	0,678623157	0,467	0,149	1,43E-22
RAB13	1,32E-26	0,66036215	0,359	0,096	2,50E-22
LILRB2	1,53E-26	0,547534125	0,401	0,11	2,89E-22
MTSS1	2,01E-26	0,668639243	0,377	0,102	3,81E-22
PEA15	2,43E-26	0,741805865	0,605	0,244	4,60E-22
CD74	2,43E-26	0,639631061	0,976	0,871	4,61E-22
CD86	1,56E-25	0,633562707	0,293	0,067	2,96E-21
EMP1	3,19E-25	0,67134004	0,365	0,101	6,05E-21
GNS	6,93E-25	0,76302916	0,569	0,23	1,31E-20
YWHAG	9,00E-25	0,723597401	0,455	0,158	1,71E-20
TLR2	2,08E-24	0,509768131	0,281	0,065	3,93E-20
RNASE6	3,25E-24	0,684487597	0,317	0,082	6,16E-20
TUBA1B	3,53E-24	0,77343111	0,749	0,399	6,70E-20
FGR	3,73E-24	0,6025795	0,455	0,149	7,06E-20
GAA	4,65E-24	0,620839199	0,359	0,102	8,81E-20
AP2S1	5,18E-24	0,724469822	0,563	0,218	9,81E-20
SLC15A3	5,23E-24	0,601202711	0,281	0,067	9,90E-20
DAB2	5,56E-24	0,76001315	0,413	0,13	1,05E-19
CEL	6,16E-24	1,233067086	0,509	0,204	1,17E-19
RAC1	6,69E-24	0,737124939	0,778	0,493	1,27E-19
ATP6V1F	7,40E-24	0,757602574	0,563	0,239	1,40E-19

THEMIS2	2,24E-23	0,515357425	0,317	0,082	4,25E-19
CD4	4,40E-23	0,743307775	0,713	0,365	8,33E-19
PAPSS1	4,92E-23	0,571469197	0,377	0,115	9,33E-19
ABHD12	6,71E-23	0,654028788	0,341	0,096	1,27E-18
COLGALT1	6,75E-23	0,565673218	0,377	0,111	1,28E-18
ASAH1	1,47E-22	0,790869476	0,521	0,208	2,79E-18
NME1	1,67E-22	0,564713292	0,281	0,069	3,17E-18
ATF5	2,98E-22	0,731107071	0,263	0,064	5,64E-18
MFSD1	3,39E-22	0,736954607	0,431	0,151	6,42E-18
LAIR1	6,31E-22	0,633292468	0,473	0,169	1,20E-17
SH3BGRL3	8,94E-22	0,61321098	0,97	0,846	1,69E-17
CD9	1,23E-21	0,784565658	0,509	0,201	2,32E-17
PLBD1	1,62E-21	0,446516286	0,257	0,062	3,06E-17
KCNMA1	2,01E-21	0,46604752	0,251	0,058	3,80E-17
IFNGR2	2,32E-21	0,669380201	0,389	0,129	4,40E-17
CTSS	2,90E-21	0,689319201	0,707	0,377	5,49E-17
ENO1	4,47E-21	0,844181838	0,743	0,481	8,47E-17
BLVRB	5,08E-21	0,573410373	0,275	0,071	9,62E-17
RGS1	5,22E-21	0,71503643	0,317	0,09	9,90E-17
ALCAM	1,22E-20	0,836962105	0,377	0,126	2,31E-16
PLEKHO2	1,47E-20	0,477435617	0,269	0,068	2,79E-16
MGLL	2,67E-20	0,640992404	0,257	0,065	5,06E-16
IL18	3,48E-20	0,506804156	0,311	0,089	6,59E-16
PTAFR	4,52E-20	0,607496068	0,455	0,164	8,57E-16
RNF130	4,66E-20	0,638794295	0,419	0,152	8,83E-16
NINJ1	5,20E-20	0,441567152	0,317	0,09	9,86E-16
S100A6	5,45E-20	0,716061163	0,874	0,671	1,03E-15
RNH1	6,66E-20	0,669144635	0,503	0,201	1,26E-15
HLA-DPA1	9,06E-20	0,495113017	0,88	0,61	1,72E-15
PGD	1,13E-19	0,419939974	0,299	0,082	2,15E-15
PRKCD	1,25E-19	0,345987553	0,299	0,082	2,36E-15
PARVB	1,34E-19	0,419083629	0,269	0,071	2,55E-15
SERF2	1,36E-19	0,627825037	0,898	0,696	2,58E-15
TUBB6	1,44E-19	0,499184855	0,275	0,074	2,73E-15
MYOF	2,21E-19	0,487364403	0,305	0,088	4,18E-15
CEP170	4,03E-19	0,558480743	0,371	0,126	7,63E-15
GNAI2	4,83E-19	0,554068169	0,874	0,648	9,16E-15
ACSL1	5,26E-19	0,551393859	0,257	0,067	9,97E-15
PHLDA1	5,74E-19	0,590944622	0,263	0,07	1,09E-14
TMEM176B	6,82E-19	0,715038905	0,377	0,128	1,29E-14
SPRED1	6,97E-19	0,566599951	0,305	0,093	1,32E-14
GM2A	9,31E-19	0,730116697	0,359	0,119	1,76E-14
PLXND1	1,01E-18	0,512744479	0,353	0,115	1,91E-14
SAT1	1,02E-18	0,688681454	0,683	0,352	1,93E-14
TWF2	1,12E-18	0,526912543	0,329	0,104	2,11E-14
PLIN3	1,27E-18	0,679067537	0,425	0,16	2,41E-14
MFSD12	1,31E-18	0,463299399	0,275	0,075	2,48E-14
ALOX5	1,63E-18	0,465267808	0,329	0,101	3,10E-14
EMP3	2,35E-18	0,643936678	0,743	0,437	4,45E-14
PRDX1	2,67E-18	0,743138001	0,551	0,264	5,06E-14
DDR2	3,03E-18	0,471629372	0,371	0,126	5,74E-14
PLOD1	3,06E-18	0,545032445	0,251	0,067	5,80E-14
GNAO1	3,14E-18	0,491431262	0,605	0,273	5,96E-14
SYNGR2	3,83E-18	0,591361561	0,479	0,196	7,25E-14
TPD52L2	4,68E-18	0,410959435	0,335	0,109	8,86E-14
MGAT1	5,36E-18	0,657097764	0,461	0,19	1,02E-13

EPB41L3	6,07E-18	0,510896903	0,257	0,07	1,15E-13
CCL3	6,28E-18	0,514608758	0,269	0,075	1,19E-13
NCF2	6,77E-18	0,370445875	0,293	0,085	1,28E-13
TCIRG1	7,53E-18	0,574181244	0,503	0,208	1,43E-13
LY86	8,02E-18	0,423760757	0,269	0,075	1,52E-13
ATP6AP2	8,19E-18	0,707977259	0,605	0,317	1,55E-13
SORT1	8,81E-18	0,544909617	0,287	0,086	1,67E-13
SOX4	8,83E-18	0,544978688	0,347	0,117	1,67E-13
STAB1	1,07E-17	0,485996622	0,281	0,082	2,02E-13
VAMP8	1,14E-17	0,558879481	0,467	0,187	2,16E-13
SLC39A8	1,43E-17	0,68798891	0,275	0,081	2,72E-13
TIMP2	2,29E-17	0,567027833	0,515	0,219	4,33E-13
RBM47	3,22E-17	0,543022494	0,419	0,157	6,11E-13
KIAA0930	4,17E-17	0,602456171	0,395	0,15	7,90E-13
ARHGAP18	4,40E-17	0,462356391	0,347	0,116	8,34E-13
ENG	5,24E-17	0,322194628	0,437	0,167	9,94E-13
FNDC3B	5,32E-17	0,563796122	0,389	0,147	1,01E-12
KCTD12	5,89E-17	0,562353474	0,473	0,192	1,12E-12
BRI3	6,05E-17	0,736150826	0,443	0,188	1,15E-12
PIK3AP1	6,10E-17	0,48084776	0,347	0,117	1,16E-12
ATP6V1A	6,61E-17	0,467005414	0,455	0,182	1,25E-12
AKR1A1	1,08E-16	0,475605171	0,389	0,143	2,04E-12
GABARAP	1,18E-16	0,544196973	0,82	0,534	2,24E-12
CD83	2,69E-16	0,431764945	0,323	0,106	5,09E-12
TUBB2A	3,09E-16	0,6957934	0,665	0,394	5,86E-12
HEXB	3,76E-16	0,626478387	0,365	0,136	7,12E-12
ARL8B	3,81E-16	0,454620178	0,371	0,136	7,23E-12
MAN2B1	4,82E-16	0,553403625	0,401	0,155	9,12E-12
IFI6	4,94E-16	0,800737987	0,515	0,242	9,36E-12
EFHD2	5,23E-16	0,549118357	0,575	0,277	9,91E-12
PLEKHO1	6,39E-16	0,565352256	0,371	0,137	1,21E-11
ARRB2	7,59E-16	0,449324881	0,587	0,274	1,44E-11
HLA-DQA1	7,69E-16	0,605134595	0,539	0,254	1,46E-11
GPI	7,74E-16	0,586152709	0,509	0,234	1,47E-11
SQOR	9,25E-16	0,349739554	0,299	0,095	1,75E-11
RTN3	9,29E-16	0,520098402	0,461	0,197	1,76E-11
CAPZA2	1,04E-15	0,536606872	0,497	0,224	1,98E-11
SMS	1,07E-15	0,593433256	0,359	0,133	2,02E-11
YWHAH	1,16E-15	0,594038372	0,407	0,168	2,20E-11
PPT1	1,27E-15	0,690794174	0,539	0,271	2,40E-11
HTRA1	1,44E-15	0,710390533	0,335	0,122	2,73E-11
BCAP31	2,75E-15	0,55235563	0,473	0,211	5,21E-11
GPX4	3,15E-15	0,612833925	0,533	0,268	5,96E-11
PYCARD	3,43E-15	0,637537714	0,401	0,162	6,50E-11
CLEC7A	4,74E-15	0,435714759	0,335	0,119	8,98E-11
ICAM1	8,50E-15	0,416067585	0,251	0,077	1,61E-10
SCARB2	1,21E-14	0,500272459	0,371	0,145	2,29E-10
TPP1	1,41E-14	0,583953237	0,677	0,406	2,67E-10
COMT	1,60E-14	0,398442858	0,347	0,131	3,03E-10
SERPINA1	1,63E-14	0,405865791	0,383	0,149	3,09E-10
ATP1B3	1,99E-14	0,487087603	0,575	0,283	3,77E-10
CFD	2,03E-14	0,496173636	0,251	0,077	3,85E-10
TMED9	2,13E-14	0,477938823	0,371	0,149	4,04E-10
RNASET2	3,54E-14	0,582356803	0,647	0,345	6,71E-10
HSPB1	4,49E-14	0,456257227	0,713	0,414	8,51E-10
CLIC1	4,72E-14	0,563054235	0,677	0,44	8,94E-10

VASP	5,29E-14	0,454483111	0,443	0,193	1,00E-09
AP1B1	8,51E-14	0,455789236	0,359	0,14	1,61E-09
PLEK	1,07E-13	0,443731098	0,485	0,22	2,02E-09
MYL6	1,15E-13	0,480614345	0,91	0,712	2,18E-09
ALDH3B1	1,15E-13	0,418721903	0,329	0,122	2,19E-09
ARAP1	1,55E-13	0,314311892	0,293	0,101	2,93E-09
ARPC4	1,55E-13	0,598350253	0,647	0,36	2,94E-09
CCL2	1,60E-13	0,91531664	0,269	0,093	3,03E-09
LAPTM4A	1,62E-13	0,458673086	0,527	0,253	3,08E-09
RTN4	1,77E-13	0,601263085	0,683	0,452	3,35E-09
TNS1	2,20E-13	0,356487278	0,335	0,126	4,17E-09
ATP6AP1	2,35E-13	0,406407333	0,377	0,152	4,45E-09
ZYX	2,52E-13	0,605468133	0,647	0,395	4,77E-09
RALA	2,64E-13	0,5147613	0,347	0,138	5,01E-09
ABCG1	2,75E-13	0,489649912	0,251	0,083	5,22E-09
MS4A4A	2,77E-13	0,404792305	0,437	0,194	5,25E-09
GRINA	2,80E-13	0,603834257	0,323	0,126	5,30E-09
FCN1	3,29E-13	0,652275326	0,323	0,124	6,23E-09
ITGB2	3,61E-13	0,580873393	0,677	0,432	6,84E-09
AP1S2	4,77E-13	0,46882083	0,401	0,171	9,04E-09
CNPY3	5,87E-13	0,434680944	0,431	0,188	1,11E-08
ITGA5	6,48E-13	0,401682221	0,431	0,189	1,23E-08
NAGA	6,52E-13	0,326911013	0,263	0,089	1,24E-08
CD109	6,81E-13	0,373807164	0,311	0,115	1,29E-08
FERMT3	6,86E-13	0,31862116	0,383	0,154	1,30E-08
SYK	7,47E-13	0,473457101	0,413	0,178	1,41E-08
MTRNR2L1	7,91E-13	0,919265736	0,707	0,47	1,50E-08
PABPC4	1,14E-12	0,510188254	0,587	0,321	2,15E-08
TSPO	1,18E-12	0,549513355	0,503	0,272	2,23E-08
SFXN3	1,25E-12	0,377674288	0,299	0,112	2,36E-08
PTPN6	1,43E-12	0,299142681	0,365	0,144	2,71E-08
TGFBR1	1,87E-12	0,399541566	0,353	0,144	3,55E-08
GAPDH	1,92E-12	0,535604466	0,892	0,742	3,64E-08
LYN	1,98E-12	0,320579845	0,359	0,144	3,76E-08
SDC3	2,45E-12	0,564489401	0,275	0,102	4,65E-08
HLA-DRB6	2,82E-12	0,502678221	0,443	0,205	5,34E-08
RAP2B	2,92E-12	0,517475986	0,389	0,173	5,53E-08
IRAK1	2,96E-12	0,468785401	0,323	0,127	5,61E-08
HMGA1	3,06E-12	0,420828334	0,437	0,195	5,79E-08
UQCRC1	3,23E-12	0,447459345	0,329	0,133	6,12E-08
ZFP36L1	3,26E-12	0,449130655	0,796	0,548	6,18E-08
PFN1	6,08E-12	0,410989485	0,958	0,867	1,15E-07
TP53I11	6,53E-12	0,450169453	0,251	0,088	1,24E-07
PTPRE	6,97E-12	0,343321955	0,395	0,173	1,32E-07
VDAC1	7,08E-12	0,467522476	0,521	0,288	1,34E-07
mrt-01	7,28E-12	0,288242737	0,257	0,089	1,38E-07
DDAH2	7,86E-12	0,390737158	0,299	0,116	1,49E-07
PSENEN	8,07E-12	0,352229152	0,269	0,098	1,53E-07
HNRNPAB	8,23E-12	0,429022181	0,431	0,201	1,56E-07
CYBA	1,13E-11	0,448145579	0,701	0,435	2,14E-07
HLA-DPB1	1,28E-11	0,28448833	0,713	0,455	2,43E-07
GNAS	1,56E-11	0,352176279	0,94	0,851	2,96E-07
RNF13	1,68E-11	0,374651224	0,401	0,179	3,17E-07
RHOQ	1,88E-11	0,411589554	0,365	0,159	3,56E-07
HSD17B4	2,43E-11	0,294469494	0,257	0,093	4,61E-07
LSM4	2,66E-11	0,509857934	0,359	0,156	5,05E-07

PFKL	2,92E-11	0,326836073	0,353	0,148	5,54E-07
LPCAT2	3,09E-11	0,433306062	0,263	0,099	5,85E-07
PLEC	3,44E-11	0,353755539	0,557	0,295	6,52E-07
PGK1	3,82E-11	0,493642703	0,647	0,415	7,25E-07
LDHA	3,86E-11	0,605544216	0,647	0,432	7,31E-07
CLTA	3,93E-11	0,37099033	0,341	0,146	7,44E-07
PTMS	4,05E-11	0,483846463	0,461	0,231	7,67E-07
FKBP1A	4,55E-11	0,458702614	0,701	0,452	8,61E-07
RXRA	4,84E-11	0,361529444	0,275	0,104	9,17E-07
YWHAE	5,56E-11	0,414212067	0,503	0,263	1,05E-06
RHOG	5,95E-11	0,505145882	0,431	0,211	1,13E-06
TPM4	6,08E-11	0,474309719	0,665	0,418	1,15E-06
SERPINB6	6,15E-11	0,345420349	0,449	0,217	1,16E-06
ZFHX3	6,49E-11	0,359318474	0,395	0,18	1,23E-06
CD151	7,27E-11	0,407246679	0,401	0,187	1,38E-06
DUSP3	7,70E-11	0,33117155	0,275	0,107	1,46E-06
BTF3L4	7,90E-11	0,373379654	0,311	0,126	1,50E-06
GLUD1	7,99E-11	0,372740481	0,389	0,178	1,51E-06
SF3B4	8,10E-11	0,566503837	0,401	0,194	1,54E-06
ALDH2	8,38E-11	0,342111316	0,287	0,114	1,59E-06
RNPEP	8,79E-11	0,292699232	0,275	0,105	1,67E-06
KDELR1	8,84E-11	0,317499433	0,377	0,167	1,68E-06
ECHS1	1,06E-10	0,366073394	0,263	0,099	2,01E-06
LIMS1	1,08E-10	0,485977039	0,467	0,25	2,05E-06
NSFL1C	1,20E-10	0,36713799	0,317	0,133	2,27E-06
CEBPD	1,27E-10	0,373292084	0,305	0,126	2,41E-06
SKAP2	1,34E-10	0,421533885	0,425	0,204	2,55E-06
LSM12	1,49E-10	0,311371843	0,263	0,1	2,83E-06
METRNL	1,54E-10	0,414508995	0,305	0,129	2,92E-06
VKORC1	2,04E-10	0,466726914	0,329	0,144	3,87E-06
SLC11A2	2,07E-10	0,278922484	0,299	0,121	3,93E-06
TNFRSF1A	2,09E-10	0,362356376	0,287	0,116	3,96E-06
TALDO1	2,09E-10	0,501368109	0,377	0,178	3,96E-06
FOS	2,09E-10	0,265603494	0,653	0,372	3,97E-06
ARHGDIA	2,13E-10	0,502025767	0,629	0,389	4,03E-06
LTA4H	2,47E-10	0,356396402	0,377	0,172	4,67E-06
ZMIZ1	2,60E-10	0,298600601	0,293	0,118	4,92E-06
CALU	2,89E-10	0,3916895	0,407	0,195	5,48E-06
MLEC	3,49E-10	0,354234291	0,347	0,155	6,61E-06
ARPC2	3,71E-10	0,308755129	0,928	0,769	7,04E-06
NUCB1	3,81E-10	0,454873935	0,503	0,267	7,22E-06
NUP62	3,84E-10	0,416529228	0,401	0,196	7,28E-06
OS9	3,86E-10	0,407660163	0,503	0,27	7,31E-06
PPP4C	4,31E-10	0,333876046	0,311	0,132	8,16E-06
MAF	4,41E-10	0,365089746	0,449	0,221	8,36E-06
NFKBIA	4,58E-10	0,449809623	0,557	0,315	8,67E-06
S100A4	5,02E-10	0,457655749	0,874	0,682	9,51E-06
BNIP3L	5,40E-10	0,44166705	0,413	0,208	1,02E-05
SOD2	5,60E-10	0,637190686	0,425	0,216	1,06E-05
DPYSL2	5,63E-10	0,338255346	0,431	0,212	1,07E-05
CALR	6,38E-10	0,629609017	0,605	0,387	1,21E-05
GGA1	6,40E-10	0,395842835	0,335	0,148	1,21E-05
MRPL23	6,42E-10	0,340466134	0,251	0,097	1,22E-05
FUCA2	8,30E-10	0,299724319	0,263	0,104	1,57E-05
CHCHD10	8,42E-10	0,398815576	0,383	0,183	1,60E-05
OTUB1	1,16E-09	0,390603821	0,329	0,145	2,19E-05

PPP1R9B	1,23E-09	0,304366571	0,269	0,108	2,32E-05
SNX2	1,46E-09	0,347269806	0,317	0,141	2,77E-05
ANXA4	1,64E-09	0,266662382	0,269	0,11	3,12E-05
SH3BP2	1,70E-09	0,382302138	0,461	0,24	3,22E-05
PGAM1	1,80E-09	0,321971979	0,371	0,176	3,41E-05
BANF1	2,09E-09	0,361683495	0,467	0,253	3,95E-05
CPM	2,13E-09	0,552140657	0,413	0,207	4,03E-05
MYO9B	2,33E-09	0,395794458	0,371	0,183	4,41E-05
TMSB10	2,51E-09	0,304966942	0,952	0,864	4,75E-05
CFL1	2,97E-09	0,431771973	0,952	0,871	5,62E-05
CRTAP	3,01E-09	0,381243621	0,575	0,341	5,71E-05
RASSF2	3,17E-09	0,320530621	0,305	0,134	6,00E-05
COMMD9	3,18E-09	0,301111852	0,281	0,117	6,02E-05
TUBA1A	3,20E-09	0,407673581	0,431	0,221	6,07E-05
MYADM	3,24E-09	0,466301921	0,587	0,379	6,14E-05
HLA-DMA	3,67E-09	0,283573231	0,395	0,191	6,96E-05
TMBIM6	3,86E-09	0,380678136	0,802	0,649	7,31E-05
STX12	3,87E-09	0,316532276	0,263	0,108	7,33E-05
SOCS3	3,94E-09	0,392396687	0,389	0,196	7,46E-05
HSPD1	4,93E-09	0,408627458	0,413	0,209	9,34E-05
PTTG1IP	5,26E-09	0,38524646	0,455	0,246	9,96E-05
GPX3	5,30E-09	0,416707594	0,251	0,104	0,000100438
GSTP1	5,40E-09	0,40674464	0,497	0,283	0,000102345
GALM	5,58E-09	0,304650521	0,365	0,173	0,000105654
ACTR1A	6,00E-09	0,448384193	0,287	0,126	0,000113695
COL6A1	6,25E-09	0,380708313	0,305	0,139	0,000118508
TSPAN3	6,36E-09	0,378561356	0,299	0,133	0,000120428
ATP6V0D1	6,79E-09	0,407517618	0,287	0,127	0,000128661
BLOC1S1	7,28E-09	0,397803451	0,449	0,237	0,000137993
LSP1	7,83E-09	0,489476939	0,581	0,362	0,000148466
NAGK	8,19E-09	0,433146606	0,377	0,191	0,00015529
CAPZB	8,83E-09	0,457434438	0,695	0,491	0,000167358
ARPC5	9,40E-09	0,365003015	0,593	0,372	0,000178182
OAZ1	9,75E-09	0,336235389	0,784	0,604	0,000184742
MPV17	9,96E-09	0,359051833	0,347	0,164	0,000188685
ARL6IP1	1,04E-08	0,348867907	0,479	0,26	0,000197679
ARL8A	1,24E-08	0,399886003	0,281	0,123	0,000234094
TIMP1	1,26E-08	0,30442233	0,754	0,509	0,000238363
P2RX7	1,51E-08	0,255064615	0,263	0,11	0,000286129
ZEB2	1,63E-08	0,307966959	0,383	0,191	0,000308983
C1orf43	1,71E-08	0,298087666	0,431	0,227	0,000324824
ARHGEF2	1,76E-08	0,322534916	0,329	0,156	0,000333078
CIAO2A	1,92E-08	0,319790867	0,281	0,123	0,000363477
PSMA4	1,97E-08	0,321873422	0,365	0,184	0,000372807
HPCAL1	2,12E-08	0,336683215	0,359	0,177	0,000401415
GAS7	2,12E-08	0,39768868	0,389	0,202	0,00040184
GNG5	2,23E-08	0,296256971	0,485	0,273	0,000422971
CD81	2,53E-08	0,437482575	0,707	0,519	0,000480228
DBNL	2,80E-08	0,294300488	0,323	0,153	0,000531054
TAGLN2	2,85E-08	0,353488172	0,569	0,36	0,000539482
SSR3	2,87E-08	0,363241306	0,455	0,252	0,000544269
ANP32A	3,13E-08	0,250661002	0,413	0,213	0,000593366
GPS2	3,26E-08	0,503569984	0,461	0,264	0,000618294
RHOC	3,31E-08	0,334887594	0,311	0,147	0,000626971
UBE2R2	3,39E-08	0,263492269	0,383	0,192	0,000642801
TRIM28	3,46E-08	0,387903228	0,455	0,254	0,000655704

PICALM	3,46E-08	0,324204702	0,407	0,217	0,000656023
SNX10	3,50E-08	0,315434826	0,257	0,109	0,000664101
AP2M1	3,58E-08	0,310249624	0,479	0,267	0,000677795
HSP90AB1	4,19E-08	0,389686993	0,766	0,574	0,000794144
AUP1	4,20E-08	0,350462013	0,395	0,208	0,000795376
CSK	4,20E-08	0,31915647	0,461	0,245	0,000796611
TPI1	4,54E-08	0,469809786	0,497	0,3	0,000859901
AURKAIP1	4,78E-08	0,328000038	0,269	0,121	0,000906701
UBA1	5,44E-08	0,331778529	0,593	0,373	0,001031807
NTAN1	5,50E-08	0,281793113	0,269	0,12	0,001042686
TMEM219	5,57E-08	0,309649328	0,437	0,237	0,001055153
EIF4G2	5,87E-08	0,405508103	0,754	0,581	0,001112744
TRMT112	5,98E-08	0,391689004	0,557	0,339	0,001132973
VCAN	6,11E-08	0,323851265	0,365	0,187	0,001157541
PSMD2	7,80E-08	0,286888937	0,389	0,207	0,001477196
PTK2B	7,87E-08	0,71979846	0,533	0,34	0,001492022
DEGS1	7,96E-08	0,307639307	0,365	0,188	0,001507556
HLA-DMB	8,31E-08	0,372183199	0,341	0,17	0,001575648
GRB2	8,39E-08	0,390159514	0,527	0,324	0,001590758
RAB10	8,97E-08	0,406753755	0,383	0,209	0,001700671
KHSRP	9,43E-08	0,360286168	0,431	0,233	0,001787534
LOC644936	1,63E-07	0,285970361	0,269	0,124	0,003089446
LAMP1	1,94E-07	0,406765509	0,497	0,309	0,00368274
HDGF	2,28E-07	0,293903002	0,425	0,234	0,004325643
NAPA	2,35E-07	0,334834848	0,377	0,204	0,00444426
VPS29	2,47E-07	0,280449978	0,389	0,207	0,004688982
QKI	2,55E-07	0,289110255	0,473	0,274	0,00483776
TXN	2,89E-07	0,368773709	0,449	0,261	0,005473304
PFKFB3	3,05E-07	0,300205333	0,275	0,129	0,005775612
ERGIC3	3,27E-07	0,35217915	0,449	0,26	0,006195051
CAPNS1	3,37E-07	0,315128634	0,629	0,411	0,006393265
CANX	3,60E-07	0,30847265	0,641	0,436	0,006819234
PTPN12	3,69E-07	0,278275442	0,359	0,189	0,006996891
METTL7A	3,71E-07	0,441080079	0,413	0,233	0,007024913
HSPA9	3,94E-07	0,266557326	0,365	0,188	0,007459246
RAD23A	5,87E-07	0,267392801	0,443	0,252	0,011126618
LILRB3	5,88E-07	0,297939929	0,263	0,124	0,011137565
SQSTM1	6,01E-07	0,259675864	0,497	0,295	0,011381588
RHOA	6,47E-07	0,335175713	0,743	0,595	0,012253406
HM13	8,44E-07	0,278964741	0,335	0,178	0,015989837
PRSS8	8,98E-07	0,351845421	0,772	0,64	0,017010681
PCBP1	9,26E-07	0,252922362	0,683	0,482	0,017552119
CMTM6	1,01E-06	0,271707747	0,551	0,341	0,019092934
ASPH	1,02E-06	0,317063648	0,323	0,169	0,019339354
COX8A	1,08E-06	0,295818389	0,569	0,375	0,020465471
COX6B1	1,15E-06	0,307054747	0,557	0,37	0,021834469
CTNNB1	1,20E-06	0,268846237	0,389	0,217	0,022689824
COL6A2	1,20E-06	0,367071277	0,263	0,129	0,022830481
RNF181	1,21E-06	0,323913322	0,293	0,149	0,022882984
HSBP1	1,42E-06	0,349754966	0,275	0,139	0,026947191
CCT2	1,44E-06	0,29411515	0,329	0,178	0,027370141
HGSNAT	1,48E-06	0,290974646	0,263	0,128	0,028054463
ATP6V1G1	1,58E-06	0,286415751	0,425	0,252	0,029902175
CDKN1A	1,63E-06	0,35857863	0,281	0,142	0,030804616
CCT5	1,72E-06	0,261141068	0,335	0,177	0,0325861
RNF145	1,77E-06	0,403620143	0,479	0,308	0,033574467

GLIPR2	1,79E-06	0,271465001	0,341	0,18	0,033977616
SLC3A2	2,40E-06	0,296315845	0,299	0,155	0,045397886
EIF5B	2,54E-06	0,290582762	0,515	0,328	0,048169316
SLC31A1	2,59E-06	0,339017163	0,347	0,189	0,049007899
ILF2	2,69E-06	0,289710858	0,323	0,171	0,051054942
UCP2	2,72E-06	0,275897491	0,611	0,395	0,051488734
VAPA	2,73E-06	0,302839973	0,425	0,248	0,051648058
SH3BGRL	2,79E-06	0,345009676	0,521	0,341	0,052905224
GHITM	2,96E-06	0,291788949	0,407	0,232	0,056161493
RPN2	3,01E-06	0,278369839	0,509	0,321	0,057024588
TBC1D9B	3,19E-06	0,294792562	0,305	0,16	0,060489252
YBX3	3,38E-06	0,27359747	0,347	0,19	0,064100566
ITPRIPL2	3,77E-06	0,362146986	0,341	0,191	0,071514526
LARP1	4,20E-06	0,330397159	0,575	0,395	0,079579716
WAS	4,39E-06	0,367255657	0,485	0,296	0,083097919
P4HB	4,70E-06	0,384768277	0,491	0,321	0,089093249
PDIA3	5,56E-06	0,267451841	0,365	0,204	0,105410818
FLOT1	5,79E-06	0,386807938	0,413	0,247	0,109808849
SRGN	6,21E-06	0,291963053	0,856	0,72	0,11766292
MAPK13	7,00E-06	0,288439139	0,275	0,141	0,132692548
ARF3	7,51E-06	0,273057543	0,407	0,234	0,142397051
SPINT2	7,65E-06	0,321477049	0,359	0,205	0,144964471
CALM2	7,68E-06	0,352516886	0,635	0,501	0,145549837
KLHDC3	1,04E-05	0,300374719	0,257	0,134	0,19644882
RTL8C	1,08E-05	0,315586534	0,281	0,152	0,205259371
PTPA	1,10E-05	0,257561492	0,269	0,14	0,207813068
SEC61A1	1,30E-05	0,342458086	0,311	0,171	0,246784924
PTMA	1,41E-05	0,271535868	0,934	0,878	0,266767181
TLN1	1,47E-05	0,259920593	0,635	0,453	0,278283526
BST2	1,51E-05	0,25273494	0,323	0,178	0,286255539
NDUFB2	1,58E-05	0,265820453	0,401	0,235	0,300175104
TXN2	2,01E-05	0,269227936	0,263	0,142	0,381420603
CSRP1	2,21E-05	0,31055187	0,341	0,199	0,418342599
CDC42	2,23E-05	0,25968388	0,713	0,536	0,422576095
RDH11	2,29E-05	0,261279865	0,263	0,137	0,434293085
PSMB3	2,44E-05	0,323635426	0,353	0,211	0,463162785
STAT6	2,58E-05	0,25363178	0,449	0,274	0,488068392
IFNGR1	2,77E-05	0,262139426	0,305	0,172	0,524802247
HSPA1A	2,97E-05	0,377949942	0,251	0,133	0,563639922
YTHDF2	3,17E-05	0,274041898	0,293	0,162	0,600394091
RBPJ	3,97E-05	0,314567461	0,509	0,353	0,752616382
NFE2L2	4,54E-05	0,2602564	0,311	0,181	0,859599793
CD84	4,55E-05	0,292719647	0,587	0,422	0,861931576
HSP90AA1	8,27E-05	0,478986802	0,76	0,668	1
PNPLA2	0,000121622	0,288985476	0,383	0,242	1
HSP90B1	0,000133028	0,544815593	0,581	0,417	1
SNRPB	0,00013516	0,268803128	0,431	0,282	1
PLCB2	0,000155576	0,261284862	0,275	0,156	1
ALOX5AP	0,000167741	0,268368348	0,443	0,287	1
IL17RA	0,000226358	0,313944633	0,401	0,263	1
EHBP1L1	0,000289348	0,282828712	0,275	0,161	1
CALM3	0,000322849	0,354354813	0,455	0,327	1
PSMD7	0,000344838	0,25992906	0,293	0,179	1
SLC25A39	0,000355036	0,365557696	0,281	0,17	1
JUND	0,000388418	0,25411841	0,79	0,677	1
CTBP2	0,000410013	0,297942658	0,329	0,203	1

RASA4	0,000814095	0,264577301	0,275	0,168	1
PDLIM1	0,005916743	0,304550869	0,341	0,238	1
HSPA5	0,008602195	0,541820352	0,413	0,318	1

Cluster 8 marker genes

gene	p_val	avg_logFC	pct.1	pct.2	p_val_adj
SOD3	7,35E-251	2,177822868	0,732	0,036	1,39E-246
NOTCH3	5,89E-250	1,992856518	0,739	0,036	1,12E-245
PDGFRB	6,29E-234	1,671788326	0,667	0,029	1,19E-229
MYLK	4,12E-231	1,831043392	0,712	0,037	7,82E-227
AEBP1	4,32E-226	2,618849042	0,824	0,063	8,19E-222
MXRA8	2,02E-218	1,466575084	0,562	0,02	3,83E-214
ANTXR1	9,10E-218	1,737970867	0,686	0,037	1,72E-213
LTBP1	1,01E-212	2,192638517	0,83	0,07	1,91E-208
FRZB	7,80E-210	1,956517424	0,562	0,022	1,48E-205
GUCY1A1	8,68E-210	2,104534865	0,758	0,053	1,65E-205
MFAP4	2,34E-207	2,03616264	0,588	0,026	4,43E-203
THBS2	8,41E-206	2,373339658	0,778	0,062	1,59E-201
MAP1B	1,19E-200	2,253309982	0,83	0,075	2,26E-196
PRRX1	1,28E-196	1,432508852	0,588	0,028	2,43E-192
MFGE8	1,34E-196	1,84763256	0,725	0,053	2,54E-192
NOV	2,24E-193	2,028196104	0,614	0,034	4,24E-189
COL14A1	7,24E-189	2,137655589	0,771	0,067	1,37E-184
PDE5A	3,71E-188	1,711303124	0,575	0,029	7,04E-184
MYL9	1,23E-186	2,450697792	0,843	0,089	2,32E-182
ID4	2,35E-186	1,545778107	0,536	0,024	4,45E-182
CRYAB	5,49E-182	1,845819251	0,66	0,046	1,04E-177
IGFBP2	5,91E-182	1,903567493	0,68	0,05	1,12E-177
MYH10	1,46E-178	2,166489847	0,739	0,065	2,76E-174
TPM2	1,09E-175	2,210580869	0,797	0,081	2,07E-171
PLS3	7,72E-175	1,77573255	0,745	0,067	1,46E-170
PDGFRA	2,55E-174	1,050836316	0,458	0,017	4,83E-170
ASPN	4,01E-174	1,711532329	0,542	0,028	7,61E-170
C1S	3,05E-173	1,895012505	0,765	0,073	5,79E-169
ADIRF	1,25E-171	2,195126464	0,876	0,11	2,37E-167
CALD1	1,36E-171	2,386500982	0,935	0,136	2,57E-167
PCOLCE	3,17E-169	1,697604551	0,549	0,031	6,00E-165
C1R	9,17E-169	2,015409291	0,778	0,079	1,74E-164
FSTL1	7,01E-168	1,826361632	0,797	0,084	1,33E-163
TAGLN	2,62E-166	2,84777546	0,869	0,12	4,96E-162
FXYD1	6,21E-166	1,186409378	0,542	0,029	1,18E-161
ECM2	6,80E-165	1,288847625	0,529	0,028	1,29E-160
DKK3	1,62E-163	1,986899042	0,752	0,077	3,06E-159
NDUFA4L2	5,94E-163	1,342313429	0,392	0,012	1,13E-158
COL6A1	3,53E-162	2,09563066	0,869	0,112	6,69E-158
PLAC9	5,45E-161	1,192768503	0,471	0,021	1,03E-156
LMOD1	2,60E-160	1,354538284	0,471	0,021	4,93E-156
COL6A2	2,93E-159	2,073362118	0,83	0,102	5,55E-155
COL6A3	2,24E-158	1,606128936	0,444	0,019	4,24E-154
COL1A2	4,20E-158	3,008835257	0,856	0,12	7,95E-154
ADH1B	2,54E-157	2,181348481	0,569	0,039	4,82E-153
COL1A1	7,61E-152	2,656108203	0,725	0,074	1,44E-147
TMEM47	9,59E-151	1,49664617	0,621	0,05	1,82E-146
PDLIM3	9,82E-151	1,239099744	0,51	0,03	1,86E-146
SFRP4	2,93E-149	1,593646094	0,379	0,013	5,55E-145
OGN	3,45E-147	2,473259881	0,83	0,117	6,54E-143
RCAN2	8,82E-146	1,434394075	0,516	0,033	1,67E-141
SMOC2	8,14E-145	1,316180818	0,412	0,018	1,54E-140
PCOLCE2	4,76E-143	1,374334656	0,386	0,015	9,03E-139

ANO1	5,01E-141	0,929630098	0,386	0,015	9,49E-137
SSPN	1,33E-139	1,252565765	0,529	0,036	2,53E-135
NR2F2	2,75E-139	1,72438402	0,771	0,093	5,22E-135
CDH13	2,14E-138	1,402777294	0,634	0,059	4,05E-134
GEM	2,22E-136	1,368743913	0,438	0,023	4,20E-132
ISLR	5,03E-136	1,577664782	0,497	0,034	9,54E-132
PPP1R14A	1,13E-134	1,46127034	0,523	0,038	2,14E-130
C2orf40	2,88E-134	1,233983234	0,346	0,012	5,46E-130
SERPING1	1,77E-133	1,791240582	0,778	0,11	3,36E-129
ITGBL1	1,89E-133	1,546209075	0,523	0,04	3,59E-129
SLIT1	6,61E-133	1,412858793	0,529	0,04	1,25E-128
TPM1	4,77E-132	1,844466832	0,739	0,095	9,04E-128
LTBP2	1,43E-131	2,26774736	0,758	0,106	2,71E-127
PALLD	1,66E-130	1,542112993	0,686	0,079	3,15E-126
LUM	3,08E-130	2,309709727	0,471	0,032	5,84E-126
FMOD	4,33E-130	1,341767042	0,464	0,03	8,21E-126
EFHD1	1,48E-129	1,107184436	0,399	0,019	2,81E-125
IGFBP6	1,07E-127	1,7100559	0,477	0,033	2,02E-123
EFEMP1	4,16E-127	2,116036854	0,824	0,134	7,88E-123
FHL1	2,68E-126	2,063727868	0,712	0,093	5,08E-122
C1QTNF1	3,02E-126	1,541771263	0,647	0,071	5,73E-122
SPARC	1,47E-123	2,265096333	0,915	0,195	2,78E-119
CYBRD1	2,16E-122	1,507431285	0,699	0,089	4,10E-118
HSPB6	2,86E-122	1,477931538	0,588	0,058	5,42E-118
DPYSL3	3,64E-122	1,297156706	0,575	0,054	6,90E-118
SDC2	1,16E-120	1,281051812	0,627	0,067	2,20E-116
C1orf198	6,22E-120	1,19656247	0,556	0,051	1,18E-115
EPDR1	7,27E-120	0,777348528	0,333	0,013	1,38E-115
LHFPL6	1,88E-119	1,47698334	0,621	0,069	3,57E-115
NFIX	1,88E-119	1,273287201	0,627	0,069	3,57E-115
IGFBP5	2,23E-119	2,369791347	0,837	0,153	4,22E-115
MRVI1	8,30E-119	1,153915006	0,386	0,021	1,57E-114
AQP1	1,79E-118	1,655732398	0,81	0,129	3,40E-114
C11orf96	7,57E-118	1,829133734	0,556	0,053	1,43E-113
BTF3P11	1,30E-115	1,495969986	0,346	0,016	2,46E-111
BGN	2,63E-114	2,46639013	0,928	0,242	4,99E-110
PRKG1	2,97E-114	0,855332349	0,366	0,019	5,63E-110
SPARCL1	3,11E-114	1,872346064	0,778	0,124	5,89E-110
FBLN5	1,07E-113	1,753026511	0,699	0,1	2,03E-109
TBX2	2,88E-113	1,006125948	0,333	0,015	5,46E-109
COL3A1	1,16E-112	2,609399472	0,758	0,121	2,19E-108
CTGF	1,44E-112	2,190125866	0,66	0,085	2,72E-108
VCAN	1,10E-111	2,097060513	0,843	0,164	2,09E-107
COL12A1	3,03E-111	0,977549794	0,392	0,024	5,75E-107
MYH11	5,73E-111	2,456773762	0,667	0,096	1,09E-106
HEYL	8,94E-111	1,052470368	0,34	0,016	1,69E-106
PFN2	2,29E-109	1,203555255	0,431	0,032	4,34E-105
ANGPT1	1,90E-108	0,799966968	0,333	0,016	3,60E-104
NFIB	4,19E-108	1,349387669	0,797	0,127	7,94E-104
SELENOM	2,17E-107	1,214078287	0,693	0,095	4,11E-103
NEXN	2,24E-107	1,031062087	0,438	0,033	4,25E-103
CXCL12	2,97E-107	1,47195413	0,562	0,061	5,63E-103
COL21A1	4,26E-107	0,880372051	0,294	0,012	8,08E-103
EMILIN1	1,44E-106	0,883492425	0,418	0,03	2,72E-102
CNN3	4,58E-106	0,995308656	0,529	0,052	8,68E-102
CCDC80	5,12E-106	2,401382071	0,621	0,081	9,69E-102

TGFB1I1	5,62E-105	1,121354971	0,484	0,044	1,06E-100
FIBIN	6,67E-105	0,719900737	0,261	0,008	1,26E-100
FN1	1,72E-103	2,1528603	0,941	0,268	3,26E-99
TNS1	3,24E-103	1,620728616	0,706	0,109	6,14E-99
EBF1	6,14E-103	1,055010772	0,49	0,045	1,16E-98
DPT	2,02E-102	1,318826067	0,294	0,013	3,84E-98
A2M	3,79E-102	1,662537058	0,765	0,135	7,19E-98
LOXL1	2,16E-101	0,823252716	0,314	0,015	4,08E-97
COL4A2	2,32E-101	1,47963045	0,673	0,099	4,40E-97
JAM3	3,41E-101	0,795200669	0,392	0,027	6,47E-97
LAMA2	2,55E-100	0,947357442	0,373	0,025	4,83E-96
ARHGEF17	5,39E-99	0,700794701	0,307	0,015	1,02E-94
OMD	9,58E-99	1,523661813	0,536	0,06	1,82E-94
KANK2	1,50E-98	1,218942408	0,536	0,061	2,85E-94
AGT	6,83E-98	1,555456151	0,438	0,039	1,29E-93
AOC3	1,53E-97	0,756046734	0,307	0,015	2,90E-93
RGS5	3,34E-97	2,484832402	0,608	0,088	6,32E-93
FBN1	6,09E-97	1,110872837	0,484	0,048	1,15E-92
CPE	1,69E-96	1,479927121	0,477	0,047	3,20E-92
GAP43	1,64E-95	1,007451523	0,288	0,013	3,11E-91
JAG1	3,26E-95	1,081199103	0,536	0,062	6,17E-91
MRC2	7,45E-95	1,007405706	0,451	0,042	1,41E-90
CFH	1,75E-94	2,110698264	0,641	0,105	3,31E-90
TMEM30B	4,97E-94	0,724426796	0,281	0,013	9,41E-90
COL18A1	2,09E-93	1,47258331	0,569	0,074	3,97E-89
LAMB2	5,80E-92	1,043390079	0,542	0,067	1,10E-87
IGFBP7	8,40E-92	2,385623558	0,935	0,371	1,59E-87
SYNPO	1,17E-91	1,200266592	0,647	0,097	2,21E-87
EFEMP2	2,89E-91	0,766874805	0,353	0,025	5,48E-87
ID3	4,83E-91	1,284239965	0,536	0,065	9,16E-87
GPC6	5,99E-91	0,792356707	0,281	0,014	1,13E-86
MIR100HG	7,63E-91	0,830992918	0,34	0,022	1,45E-86
CAVIN1	1,59E-90	1,385501941	0,81	0,163	3,02E-86
PPP1R3C	4,23E-90	0,703158494	0,275	0,013	8,02E-86
FOXC1	5,22E-90	1,293221956	0,556	0,072	9,89E-86
LRRC17	3,24E-89	0,972751928	0,333	0,022	6,14E-85
ANG	1,24E-88	0,900702283	0,379	0,031	2,36E-84
DSTN	2,10E-88	1,82201246	0,83	0,214	3,98E-84
LRRC32	2,21E-88	0,975901353	0,392	0,034	4,19E-84
HTRA1	2,80E-88	1,30798847	0,654	0,107	5,30E-84
CHPF	3,67E-88	0,884868136	0,327	0,021	6,95E-84
PRELP	5,00E-88	2,205314704	0,889	0,287	9,47E-84
SULF1	7,91E-88	1,413478516	0,673	0,113	1,50E-83
ACTA2	8,35E-88	2,695458003	0,804	0,209	1,58E-83
RGS4	1,69E-87	0,726903341	0,255	0,011	3,20E-83
LIMCH1	1,86E-87	1,142436501	0,536	0,068	3,52E-83
CLIC4	9,07E-87	1,271593795	0,706	0,13	1,72E-82
INHBA	1,21E-86	0,918136467	0,333	0,023	2,29E-82
GPX3	2,73E-86	1,387880032	0,588	0,088	5,17E-82
SYDE1	5,44E-86	0,651645926	0,255	0,012	1,03E-81
CX3CL1	1,61E-85	0,787554789	0,359	0,028	3,06E-81
MYO1D	6,18E-85	1,013977774	0,516	0,065	1,17E-80
PTPRS	8,81E-85	0,712094026	0,34	0,025	1,67E-80
FMO2	4,62E-84	1,338064776	0,366	0,031	8,76E-80
RBPMS	1,78E-83	1,038666336	0,49	0,058	3,37E-79
FHL2	2,12E-83	0,764967344	0,307	0,02	4,01E-79

GNG12	2,53E-83	1,06671936	0,523	0,068	4,80E-79
TFPI	8,05E-83	1,006192512	0,477	0,055	1,53E-78
CYR61	9,81E-83	1,384555608	0,497	0,061	1,86E-78
PAM	1,37E-82	1,122246592	0,621	0,101	2,59E-78
GULP1	1,20E-81	0,615494915	0,275	0,015	2,27E-77
FKBP10	4,61E-81	0,687846849	0,288	0,018	8,74E-77
LGALS3BP	7,57E-81	1,109202376	0,536	0,073	1,43E-76
NFIA	9,20E-81	1,502677881	0,758	0,164	1,74E-76
NNMT	1,40E-80	0,93576496	0,464	0,053	2,65E-76
PLN	3,94E-80	1,177189117	0,333	0,026	7,46E-76
COL5A2	2,35E-79	1,021063862	0,412	0,043	4,46E-75
COL5A1	3,47E-79	1,133752506	0,373	0,034	6,58E-75
AKAP12	1,30E-78	1,158419477	0,418	0,045	2,47E-74
COL4A1	3,36E-78	1,134559633	0,503	0,066	6,37E-74
DMD	4,55E-78	0,854587214	0,366	0,033	8,61E-74
PRSS23	5,48E-78	1,428990344	0,542	0,081	1,04E-73
GSN	1,22E-77	2,096384754	0,889	0,294	2,32E-73
LRP1	5,25E-77	1,27899268	0,667	0,127	9,94E-73
ITGA11	5,90E-77	0,607979453	0,275	0,017	1,12E-72
MGP	1,61E-76	2,547985476	0,98	0,548	3,06E-72
NUPR1	2,94E-76	0,75918504	0,386	0,038	5,58E-72
RRAS	3,23E-76	1,079361323	0,529	0,077	6,13E-72
UNC5B	3,27E-76	1,081419413	0,418	0,047	6,19E-72
SORBS1	6,26E-76	0,889436285	0,32	0,025	1,19E-71
PDGFD	3,61E-75	0,977717543	0,412	0,045	6,84E-71
TIMP2	4,10E-75	1,602180622	0,784	0,207	7,76E-71
GAS6	6,82E-75	1,22658726	0,634	0,115	1,29E-70
COL16A1	1,43E-74	0,682327823	0,294	0,021	2,72E-70
HEY2	1,54E-74	0,612523892	0,255	0,015	2,91E-70
CAVIN3	3,87E-73	1,100648196	0,51	0,074	7,33E-69
EPS8	3,21E-72	0,855888824	0,418	0,049	6,08E-68
IL33	5,08E-72	0,950742079	0,497	0,069	9,63E-68
ITGA7	1,10E-71	0,798640749	0,261	0,017	2,09E-67
GATA6	1,63E-71	0,799840127	0,353	0,034	3,08E-67
SVIL	3,97E-71	0,938662245	0,49	0,069	7,52E-67
ITGB5	8,74E-71	1,176433702	0,497	0,074	1,66E-66
CCDC3	1,27E-70	1,035164808	0,359	0,036	2,41E-66
KCNMB1	5,41E-70	0,883198897	0,34	0,032	1,02E-65
YAP1	6,53E-70	0,772593812	0,386	0,043	1,24E-65
PLPP3	8,00E-70	1,068861868	0,484	0,068	1,52E-65
CFB	1,25E-69	0,743184764	0,294	0,023	2,37E-65
PXDN	1,39E-69	0,926040725	0,366	0,038	2,63E-65
PARM1	3,70E-69	0,683552187	0,307	0,025	7,02E-65
TWSG1	8,73E-69	0,734535306	0,418	0,051	1,65E-64
WWTR1	1,56E-68	0,95819768	0,529	0,082	2,95E-64
PHLDB1	1,86E-68	0,928598048	0,359	0,037	3,52E-64
RBMS3	4,39E-67	0,911298879	0,471	0,067	8,31E-63
LTBP4	1,04E-66	1,204171023	0,66	0,143	1,97E-62
PCDH7	1,48E-66	1,210981848	0,444	0,062	2,81E-62
MXRA7	1,52E-66	1,063226378	0,66	0,14	2,88E-62
VCL	1,65E-66	1,083989161	0,66	0,137	3,13E-62
TCEAL9	1,93E-66	0,783289508	0,451	0,063	3,65E-62
MYO1B	2,27E-66	0,616628515	0,288	0,023	4,30E-62
CRIP2	1,29E-65	1,086947334	0,693	0,152	2,45E-61
COL8A1	2,50E-65	1,228862887	0,412	0,054	4,73E-61
SYNPO2	5,86E-64	1,12406566	0,444	0,064	1,11E-59

GLT8D2	1,05E-63	0,715921888	0,294	0,026	2,00E-59
NCKAP1	2,65E-63	0,785051874	0,49	0,075	5,02E-59
EPAS1	7,83E-63	1,4419912	0,745	0,2	1,48E-58
SPRY1	1,72E-62	0,920877224	0,366	0,043	3,26E-58
LMO7	4,49E-62	0,872683115	0,366	0,043	8,51E-58
COX7A1	4,59E-62	0,635393597	0,333	0,035	8,69E-58
SGCB	1,56E-61	0,734584025	0,359	0,042	2,96E-57
ASAP2	3,65E-61	0,888292932	0,386	0,05	6,92E-57
GGT5	4,03E-61	0,795562995	0,268	0,022	7,63E-57
MT1E	7,84E-61	0,661297508	0,333	0,036	1,49E-56
TCF4	2,68E-60	1,15221317	0,784	0,224	5,09E-56
TCEAL4	2,82E-60	0,96933062	0,503	0,086	5,35E-56
MAP1A	4,80E-60	0,630351273	0,255	0,02	9,10E-56
CTSK	5,77E-60	0,912814452	0,471	0,076	1,09E-55
SORBS3	1,88E-59	0,968777882	0,529	0,098	3,56E-55
CTTN	2,48E-59	0,76646925	0,399	0,054	4,69E-55
PTGIS	2,64E-59	1,202103012	0,595	0,127	5,01E-55
DST	3,27E-59	0,921757046	0,549	0,105	6,20E-55
FBLN1	5,36E-59	1,4587029	0,49	0,087	1,02E-54
SSC5D	9,27E-59	0,81782215	0,268	0,023	1,76E-54
THRB	2,01E-58	0,667096869	0,268	0,023	3,82E-54
SERPINH1	2,83E-58	0,813346756	0,412	0,06	5,37E-54
TM4SF1	3,87E-58	0,841854736	0,608	0,121	7,34E-54
NDN	1,09E-57	0,680845878	0,261	0,023	2,06E-53
MMP2	1,44E-57	1,051741913	0,471	0,078	2,74E-53
CD9	1,51E-57	1,19587848	0,725	0,192	2,87E-53
CAV1	2,49E-57	0,889166083	0,621	0,133	4,72E-53
MICAL2	2,22E-56	1,278187715	0,627	0,151	4,21E-52
TSC22D1	2,39E-56	0,994465742	0,477	0,083	4,53E-52
ELN	4,59E-56	1,126947577	0,562	0,115	8,70E-52
DCN	7,05E-56	1,754532034	0,301	0,033	1,34E-51
CYB5R3	8,33E-56	1,212299514	0,778	0,258	1,58E-51
PMEPA1	2,18E-55	1,030046358	0,536	0,107	4,14E-51
AXL	2,86E-55	0,592507894	0,353	0,044	5,43E-51
CTSF	4,21E-55	0,884348678	0,373	0,052	7,98E-51
TINAGL1	9,41E-55	0,94340793	0,51	0,098	1,78E-50
CD151	1,06E-54	1,086928148	0,68	0,174	2,00E-50
OSMR	1,07E-54	0,635891836	0,288	0,03	2,04E-50
FAT4	1,42E-54	0,637105291	0,255	0,023	2,69E-50
FBLN2	1,92E-54	1,106736169	0,399	0,06	3,63E-50
HSPB1	3,23E-53	1,286455645	0,889	0,407	6,13E-49
SPTBN1	4,06E-53	1,159897719	0,745	0,221	7,69E-49
ECM1	6,27E-53	0,635334871	0,288	0,031	1,19E-48
PDGFA	7,24E-53	0,85878948	0,405	0,063	1,37E-48
RBFOX2	1,05E-52	0,74825031	0,379	0,055	1,98E-48
PLSCR4	1,22E-52	0,905527933	0,386	0,058	2,32E-48
ZNF503	1,22E-52	0,974824315	0,379	0,057	2,32E-48
TGM2	1,24E-52	1,126386371	0,471	0,086	2,36E-48
MSRB3	1,37E-52	1,004614872	0,562	0,125	2,59E-48
VIM	2,28E-52	1,134836266	0,961	0,823	4,32E-48
CPED1	2,63E-52	0,615017596	0,301	0,034	4,98E-48
CTDSPL	1,81E-51	0,587272905	0,275	0,029	3,43E-47
LAMC1	3,18E-51	0,88863782	0,484	0,091	6,03E-47
BEX3	8,89E-51	0,883664856	0,477	0,091	1,69E-46
GLIS2	6,47E-50	0,58357301	0,261	0,027	1,23E-45
NFIC	7,40E-50	1,143169411	0,791	0,282	1,40E-45

FGFR1	2,20E-49	0,839151064	0,497	0,1	4,18E-45
CPQ	2,44E-49	0,62276857	0,405	0,066	4,62E-45
SYNM	2,61E-49	0,727704586	0,294	0,035	4,94E-45
COL15A1	2,92E-49	0,939502144	0,392	0,063	5,54E-45
PDLIM7	1,13E-48	0,81964479	0,444	0,082	2,14E-44
PARVA	2,71E-48	0,880549301	0,562	0,126	5,14E-44
PROS1	5,35E-48	0,650989147	0,327	0,045	1,01E-43
HCFC1R1	5,99E-48	0,850863362	0,444	0,084	1,13E-43
CERCAM	7,35E-48	0,629573223	0,281	0,033	1,39E-43
PDE3A	1,04E-47	0,690667006	0,333	0,047	1,98E-43
SLC22A17	1,16E-47	0,768490488	0,275	0,031	2,20E-43
FERMT2	2,40E-47	0,613712866	0,346	0,05	4,55E-43
ISYNA1	9,62E-47	0,788335595	0,353	0,054	1,82E-42
PKIG	9,70E-47	0,777222815	0,359	0,056	1,84E-42
CRIM1	1,23E-46	0,815892133	0,621	0,158	2,33E-42
TNS2	1,63E-46	0,537043897	0,281	0,034	3,09E-42
GADD45A	1,88E-46	0,813976999	0,392	0,066	3,56E-42
ZFHX3	3,41E-46	0,949213459	0,634	0,169	6,46E-42
IFITM3	3,72E-46	1,08188002	0,902	0,494	7,06E-42
TIMP3	3,82E-46	1,681136649	0,418	0,08	7,23E-42
KANK1	6,53E-46	0,612664504	0,294	0,038	1,24E-41
NTRK3	8,58E-46	0,871381852	0,261	0,03	1,63E-41
INMT	1,04E-45	0,76211005	0,32	0,046	1,97E-41
PGRMC1	1,11E-45	0,823403255	0,451	0,089	2,11E-41
GJA1	1,80E-45	0,63361846	0,346	0,052	3,41E-41
SCARA3	4,28E-45	1,022613441	0,333	0,05	8,12E-41
S100A6	4,74E-45	1,085448979	0,941	0,669	8,98E-41
ABI3BP	5,77E-45	0,643298741	0,275	0,034	1,09E-40
PKD2	9,64E-45	0,996644795	0,549	0,136	1,83E-40
FADS2	1,91E-44	0,482826195	0,268	0,032	3,61E-40
TIMP1	3,64E-44	1,528474448	0,889	0,504	6,90E-40
PBX1	9,03E-44	0,843213528	0,425	0,082	1,71E-39
CPXM2	1,24E-43	0,600918527	0,346	0,054	2,34E-39
SH3D19	3,30E-43	0,517920744	0,261	0,031	6,24E-39
LMCD1	4,79E-43	0,7005998	0,255	0,03	9,08E-39
S100A13	1,16E-42	0,622127524	0,32	0,048	2,19E-38
RAB34	1,25E-42	0,682183647	0,399	0,075	2,36E-38
PHLDB2	1,51E-42	0,655894088	0,307	0,044	2,86E-38
SORT1	2,55E-42	0,836919005	0,412	0,081	4,82E-38
CYTH3	5,37E-42	0,495511416	0,294	0,041	1,02E-37
ITGA10	5,44E-42	0,62317191	0,327	0,05	1,03E-37
EPB41L2	7,66E-42	0,884658833	0,438	0,092	1,45E-37
GNA11	1,25E-41	0,578004171	0,294	0,042	2,36E-37
UACA	1,46E-41	1,046163289	0,49	0,118	2,77E-37
HMCN1	1,47E-41	0,863685595	0,353	0,06	2,79E-37
PMP22	3,29E-41	0,65554937	0,425	0,085	6,24E-37
EPHX1	3,32E-41	0,705101744	0,346	0,059	6,29E-37
ZCCHC24	4,16E-41	0,699277898	0,359	0,063	7,88E-37
C9orf3	7,43E-41	0,506216367	0,314	0,048	1,41E-36
EHD2	7,74E-41	0,813895448	0,425	0,085	1,47E-36
PPP1R12B	8,19E-41	0,835068822	0,451	0,097	1,55E-36
MRFAP1	1,68E-40	0,834605461	0,621	0,182	3,18E-36
ZNF704	2,65E-40	0,657001338	0,412	0,08	5,03E-36
APP	5,49E-40	0,749291367	0,569	0,147	1,04E-35
CAV2	5,77E-40	0,891149323	0,582	0,163	1,09E-35
LAMB1	1,41E-39	0,750170494	0,418	0,085	2,67E-35

CLU	1,49E-39	1,378022188	0,719	0,248	2,83E-35
AMOTL2	2,99E-39	0,743867556	0,366	0,067	5,67E-35
CALU	6,48E-39	0,861121669	0,621	0,185	1,23E-34
CDKN1C	7,35E-39	0,601957413	0,353	0,062	1,39E-34
PTK2	9,32E-39	0,709657249	0,327	0,056	1,77E-34
CDH11	1,66E-38	0,609691814	0,261	0,036	3,15E-34
IGFBP3	1,88E-38	1,191009899	0,307	0,049	3,57E-34
KDELR2	3,39E-38	0,836760543	0,562	0,157	6,41E-34
TNS3	7,73E-38	0,728087867	0,353	0,066	1,47E-33
LMNA	9,23E-37	0,738696034	0,771	0,278	1,75E-32
LAMA4	1,06E-36	0,747583245	0,412	0,088	2,01E-32
SMAD6	1,67E-36	0,598865018	0,255	0,035	3,16E-32
NAV2	2,83E-36	0,485471688	0,255	0,035	5,36E-32
RTL8C	3,14E-36	0,741836787	0,529	0,141	5,94E-32
EXT1	3,67E-36	0,604001927	0,333	0,06	6,96E-32
OLFML2B	5,94E-36	0,62477365	0,307	0,052	1,13E-31
POSTN	8,24E-36	1,639204712	0,32	0,059	1,56E-31
MATN2	1,15E-35	0,51004964	0,288	0,045	2,18E-31
FBXO32	1,44E-35	0,625163434	0,314	0,054	2,73E-31
TENT5A	1,75E-35	0,709085267	0,418	0,093	3,32E-31
LAPTM4A	1,84E-35	0,815310774	0,686	0,246	3,48E-31
TRIP6	3,70E-35	0,593764712	0,32	0,057	7,02E-31
SERPINE2	3,92E-35	1,067776409	0,255	0,038	7,44E-31
CST3	4,04E-35	0,772522926	0,843	0,403	7,66E-31
GSTT1	1,04E-34	0,567941458	0,275	0,043	1,97E-30
UGDH	1,58E-34	0,550202675	0,314	0,057	3,00E-30
KLF9	1,67E-34	0,71950815	0,484	0,124	3,16E-30
LGALS1	1,74E-34	0,820552227	0,804	0,347	3,29E-30
SASH1	1,91E-34	0,730386152	0,431	0,103	3,62E-30
CD59	1,96E-34	0,782434252	0,706	0,261	3,72E-30
SELENOW	2,14E-34	0,808355696	0,595	0,181	4,06E-30
ITGA1	2,22E-34	0,652796162	0,373	0,075	4,21E-30
SCARB2	2,89E-34	0,711857119	0,51	0,139	5,48E-30
RCAN1	1,80E-33	0,579680705	0,346	0,069	3,41E-29
HES1	7,17E-33	0,964793561	0,333	0,067	1,36E-28
MAGEH1	2,38E-32	0,45000312	0,281	0,048	4,51E-28
CCND1	6,43E-32	1,261432676	0,373	0,082	1,22E-27
VCAM1	6,49E-32	0,540652659	0,281	0,048	1,23E-27
PLAGL1	7,60E-32	0,716331432	0,268	0,045	1,44E-27
MARVELD1	1,08E-31	0,495105442	0,281	0,05	2,04E-27
IL6ST	1,37E-31	0,642338778	0,66	0,224	2,59E-27
FAM114A1	1,54E-31	0,648541129	0,359	0,08	2,92E-27
TJP2	1,67E-31	0,449884405	0,288	0,051	3,17E-27
CD63	1,69E-31	0,712564247	0,856	0,396	3,20E-27
SETBP1	2,37E-31	0,681678979	0,327	0,066	4,48E-27
TPM4	3,03E-31	0,913048291	0,817	0,412	5,74E-27
EID1	3,41E-31	0,793415127	0,765	0,338	6,46E-27
DAAM1	4,93E-31	0,659262101	0,425	0,106	9,33E-27
ACTN1	7,17E-31	0,771774305	0,706	0,27	1,36E-26
CFL2	7,54E-31	0,533175859	0,261	0,044	1,43E-26
RTL8A	9,17E-31	0,612069124	0,34	0,072	1,74E-26
sep-08	2,24E-30	0,570530683	0,366	0,082	4,25E-26
ARL1	4,31E-30	0,532342839	0,399	0,097	8,16E-26
SNX9	4,92E-30	0,761815269	0,438	0,12	9,33E-26
CDKN2C	6,12E-30	0,690420125	0,261	0,046	1,16E-25
PXDC1	6,45E-30	0,528264247	0,281	0,052	1,22E-25

TCF7L2	6,97E-30	0,607731087	0,373	0,086	1,32E-25
SBDS	7,83E-30	0,519699514	0,392	0,093	1,48E-25
ITGB1	9,45E-30	0,803018208	0,863	0,483	1,79E-25
LEPROT	9,48E-30	0,662587753	0,588	0,19	1,80E-25
CDC42EP1	1,36E-29	0,616571084	0,314	0,064	2,58E-25
DYNLL1	1,38E-29	0,763157505	0,758	0,335	2,62E-25
ENDOD1	1,39E-29	0,520238641	0,294	0,057	2,63E-25
TCEAL3	4,30E-29	0,352200794	0,255	0,043	8,14E-25
NORAD	7,59E-29	0,87057796	0,771	0,366	1,44E-24
PPIC	1,26E-28	0,446027916	0,261	0,047	2,40E-24
MCAM	1,27E-28	1,041696153	0,353	0,084	2,40E-24
ANXA4	1,42E-28	0,579727297	0,405	0,105	2,69E-24
NPTN	1,62E-28	0,740466414	0,503	0,157	3,08E-24
RAB31	2,12E-28	0,717416376	0,523	0,163	4,02E-24
NENF	4,79E-28	0,50085995	0,425	0,112	9,08E-24
LAMA5	7,00E-28	0,480345071	0,261	0,048	1,33E-23
ESD	8,09E-28	0,695655976	0,516	0,16	1,53E-23
GGTA1P	8,82E-28	0,508691394	0,294	0,06	1,67E-23
CD99L2	1,33E-27	0,34008522	0,301	0,061	2,52E-23
S100A16	1,60E-27	0,521237259	0,288	0,057	3,04E-23
DAG1	2,85E-27	0,440989969	0,281	0,056	5,39E-23
SDC4	3,90E-27	0,500101384	0,268	0,052	7,38E-23
NFE2L1	5,13E-27	0,496099347	0,458	0,128	9,72E-23
PLOD1	7,10E-27	0,563249921	0,301	0,066	1,34E-22
TMEM159	7,61E-27	0,49259132	0,261	0,05	1,44E-22
BACE2	1,18E-26	0,551695211	0,327	0,074	2,24E-22
ZBTB16	1,47E-26	0,472173838	0,294	0,061	2,78E-22
RRAGA	1,60E-26	0,476237951	0,314	0,07	3,04E-22
LATS2	7,26E-26	0,539463449	0,275	0,056	1,38E-21
FNDC3B	1,12E-25	0,518030892	0,477	0,144	2,12E-21
TMEM43	2,00E-25	0,515611592	0,503	0,159	3,79E-21
DAP	2,40E-25	0,674195373	0,418	0,123	4,54E-21
MAGED2	3,34E-25	0,581627297	0,451	0,136	6,33E-21
HDGFL3	4,89E-25	0,460798183	0,288	0,062	9,26E-21
INAFM1	5,25E-25	0,510863117	0,301	0,068	9,95E-21
FLNA	5,94E-25	0,977954726	0,824	0,508	1,13E-20
VAMP3	6,18E-25	0,489470519	0,458	0,136	1,17E-20
SLC29A1	7,30E-25	0,608908874	0,333	0,084	1,38E-20
CSF1	8,42E-25	0,471707618	0,281	0,059	1,60E-20
MYOF	8,88E-25	0,532474076	0,346	0,087	1,68E-20
CDC42BPA	8,91E-25	0,490703573	0,294	0,066	1,69E-20
ZFYVE21	9,21E-25	0,419926773	0,294	0,065	1,75E-20
TMEM9	1,38E-24	0,397178675	0,255	0,05	2,62E-20
ARHGEF12	1,50E-24	0,412232833	0,418	0,117	2,84E-20
GALNT2	1,99E-24	0,583988102	0,386	0,107	3,78E-20
TMEM204	2,11E-24	0,575523602	0,301	0,069	4,00E-20
DSEL	2,64E-24	0,523592152	0,255	0,051	4,99E-20
PKD1	2,76E-24	0,462533501	0,32	0,076	5,22E-20
PPP1CB	2,82E-24	0,707576969	0,758	0,36	5,34E-20
ADI1	3,32E-24	0,54516553	0,392	0,11	6,29E-20
FZD4	4,05E-24	0,473262044	0,288	0,064	7,68E-20
PLXNB2	4,39E-24	0,55133609	0,412	0,12	8,31E-20
PEBP1	5,76E-24	0,619985488	0,817	0,398	1,09E-19
EGR1	6,92E-24	0,916725236	0,608	0,252	1,31E-19
KCTD10	7,01E-24	0,675934652	0,327	0,083	1,33E-19
RHOB	1,04E-23	0,43719372	0,366	0,097	1,97E-19

MORF4L2	1,22E-23	0,625861927	0,471	0,156	2,32E-19
NUCKS1	2,11E-23	0,741458191	0,817	0,482	3,99E-19
LIMS2	2,55E-23	0,428745138	0,288	0,065	4,84E-19
ARHGAP29	2,82E-23	0,503492324	0,281	0,064	5,34E-19
IRF2BP2	3,42E-23	0,620940238	0,608	0,233	6,49E-19
YBX3	3,46E-23	0,562082709	0,523	0,182	6,56E-19
TMEM14C	4,30E-23	0,394022026	0,451	0,136	8,15E-19
PIGT	4,83E-23	0,459169514	0,392	0,112	9,16E-19
MT2A	6,02E-23	0,675025087	0,595	0,226	1,14E-18
CTNNA1	6,99E-23	0,576713381	0,458	0,149	1,33E-18
METTL7A	7,51E-23	0,769462578	0,575	0,226	1,42E-18
PTMS	1,13E-22	0,603371055	0,588	0,226	2,15E-18
CKAP4	1,26E-22	0,546361338	0,327	0,084	2,39E-18
SEMA5A	1,41E-22	0,659339693	0,288	0,069	2,68E-18
GBE1	1,77E-22	0,412187585	0,268	0,059	3,35E-18
CASC4	2,06E-22	0,572477287	0,497	0,172	3,91E-18
LIMA1	2,36E-22	0,488708581	0,353	0,097	4,46E-18
OAT	2,53E-22	0,468914507	0,399	0,116	4,80E-18
NAB1	2,99E-22	0,426309855	0,333	0,087	5,67E-18
COPRS	3,41E-22	0,480526215	0,275	0,064	6,46E-18
DPYSL2	4,32E-22	0,599707747	0,556	0,207	8,19E-18
MYO1C	4,44E-22	0,615569394	0,471	0,16	8,41E-18
MEGF6	4,44E-22	0,443877644	0,301	0,074	8,41E-18
CCPG1	5,64E-22	0,413098837	0,307	0,077	1,07E-17
CDK2AP1	7,90E-22	0,666864386	0,412	0,132	1,50E-17
SLC40A1	1,07E-21	0,502202162	0,359	0,1	2,03E-17
SCPEP1	1,22E-21	0,436580842	0,307	0,078	2,32E-17
DUSP1	1,37E-21	0,805101889	0,549	0,216	2,60E-17
ILK	2,07E-21	0,529968384	0,359	0,104	3,93E-17
ZSCAN18	2,18E-21	0,428476174	0,275	0,064	4,14E-17
GPX8	2,35E-21	0,507575294	0,333	0,089	4,45E-17
PDLIM5	3,03E-21	0,595410227	0,614	0,251	5,75E-17
PPFIBP1	3,24E-21	0,46495868	0,275	0,065	6,15E-17
SLC38A2	3,92E-21	0,606484426	0,51	0,188	7,42E-17
ACTN4	4,32E-21	0,655178931	0,856	0,504	8,19E-17
VKORC1	4,45E-21	0,534616964	0,431	0,14	8,42E-17
PRDX4	5,89E-21	0,376533562	0,281	0,069	1,12E-16
ALCAM	6,35E-21	0,513394769	0,405	0,126	1,20E-16
FRY	7,42E-21	0,346084697	0,255	0,057	1,41E-16
MPZL1	7,71E-21	0,364653229	0,288	0,071	1,46E-16
SLC6A2	8,34E-21	0,537379659	0,294	0,076	1,58E-16
SNX18	8,39E-21	0,493030305	0,333	0,091	1,59E-16
FOXP4	1,25E-20	0,491869967	0,268	0,064	2,36E-16
ARF4	1,35E-20	0,484210161	0,49	0,173	2,55E-16
PEA15	1,36E-20	0,584843016	0,601	0,246	2,57E-16
ZNF532	1,43E-20	0,46991117	0,327	0,089	2,70E-16
NDFIP1	2,00E-20	0,561461018	0,575	0,227	3,78E-16
ENAH	2,54E-20	0,497770777	0,458	0,157	4,82E-16
APLP2	2,88E-20	0,707636306	0,745	0,386	5,46E-16
CD81	3,22E-20	0,66311778	0,837	0,514	6,10E-16
TRIP10	3,23E-20	0,436677868	0,268	0,065	6,13E-16
RAB2A	4,61E-20	0,474070304	0,523	0,19	8,73E-16
ENG	4,81E-20	0,578387434	0,471	0,167	9,11E-16
ITGAV	7,63E-20	0,378552405	0,327	0,09	1,44E-15
CALM2	9,18E-20	0,70423212	0,83	0,492	1,74E-15
CSRP1	9,36E-20	0,918841546	0,49	0,192	1,77E-15

HSPA1A	1,48E-19	0,619008606	0,392	0,127	2,81E-15
REXO2	1,71E-19	0,472861788	0,34	0,099	3,25E-15
HDLBP	1,81E-19	0,532755385	0,556	0,221	3,44E-15
S100A4	2,21E-19	0,512081254	0,928	0,68	4,19E-15
FAM3C	3,03E-19	0,368681497	0,333	0,096	5,75E-15
CDKN1B	3,28E-19	0,341564937	0,32	0,088	6,21E-15
MGST3	3,29E-19	0,497615724	0,562	0,229	6,23E-15
ANKH	5,24E-19	0,531334173	0,516	0,2	9,92E-15
CREB3L2	6,25E-19	0,535269767	0,444	0,157	1,18E-14
SEC24D	6,49E-19	0,332293577	0,275	0,07	1,23E-14
ERLEC1	8,44E-19	0,373639886	0,353	0,105	1,60E-14
FBLIM1	8,64E-19	0,597796074	0,647	0,298	1,64E-14
ESAM	1,34E-18	0,441585786	0,268	0,068	2,55E-14
ZMAT3	1,71E-18	0,618405027	0,569	0,243	3,24E-14
TUBB2A	1,89E-18	0,483473046	0,765	0,391	3,58E-14
MBNL2	2,08E-18	0,475201106	0,405	0,137	3,95E-14
DDAH2	2,58E-18	0,477034997	0,359	0,113	4,89E-14
ANO6	2,64E-18	0,370007099	0,379	0,122	4,99E-14
MEF2C	2,85E-18	0,432204391	0,405	0,136	5,41E-14
MARCKS	2,89E-18	0,366356553	0,569	0,22	5,48E-14
LRIG1	2,90E-18	0,274374804	0,275	0,07	5,50E-14
FOS	3,15E-18	0,677067793	0,719	0,37	5,96E-14
SERINC3	3,18E-18	0,393612649	0,523	0,196	6,03E-14
MAGED1	3,76E-18	0,373800117	0,327	0,097	7,13E-14
PTTG1IP	3,83E-18	0,557480725	0,575	0,241	7,25E-14
SQSTM1	4,39E-18	0,557411933	0,627	0,289	8,31E-14
MAP3K20	4,70E-18	0,616692685	0,386	0,133	8,90E-14
C1orf54	5,54E-18	0,397731909	0,255	0,066	1,05E-13
MTUS1	5,68E-18	0,270343911	0,288	0,077	1,08E-13
MAP4	5,86E-18	0,4792057	0,484	0,185	1,11E-13
GSTM3	6,56E-18	0,396507641	0,431	0,149	1,24E-13
SEC31A	7,27E-18	0,560908723	0,503	0,205	1,38E-13
KIF13A	7,42E-18	0,510967651	0,294	0,084	1,41E-13
NAV1	7,47E-18	0,520398886	0,353	0,113	1,42E-13
EMP2	8,97E-18	0,527308644	0,588	0,248	1,70E-13
TUBA1B	1,13E-17	0,52397429	0,758	0,4	2,15E-13
MPRIP	1,19E-17	0,485598922	0,458	0,168	2,25E-13
COPB2	1,69E-17	0,367973233	0,294	0,083	3,21E-13
MORF4L1	1,73E-17	0,508439273	0,758	0,402	3,28E-13
LRP4	3,86E-17	0,49873981	0,601	0,27	7,31E-13
NGRN	4,35E-17	0,420623083	0,288	0,082	8,24E-13
PTPN14	5,22E-17	0,503773913	0,386	0,133	9,89E-13
NELL1	6,91E-17	0,438305459	0,34	0,107	1,31E-12
SLC41A3	9,22E-17	0,356276769	0,261	0,07	1,75E-12
TMBIM1	1,15E-16	0,422787456	0,379	0,13	2,18E-12
H2AFJ	1,26E-16	0,35696392	0,255	0,068	2,39E-12
SDF4	1,34E-16	0,411112247	0,451	0,171	2,54E-12
ALDH2	2,17E-16	0,437187664	0,34	0,112	4,11E-12
FGL2	2,24E-16	0,820294898	0,51	0,227	4,25E-12
SERPINE1	2,36E-16	0,932497794	0,379	0,137	4,48E-12
OXR1	2,74E-16	0,376050102	0,327	0,103	5,20E-12
LPP	2,96E-16	0,807011536	0,732	0,445	5,62E-12
ERG	3,07E-16	0,404672932	0,255	0,068	5,81E-12
ARID5B	3,44E-16	0,577900913	0,418	0,157	6,52E-12
LRRC8A	3,88E-16	0,260997688	0,281	0,08	7,36E-12
ROCK2	3,93E-16	0,444875543	0,444	0,168	7,45E-12

SHC1	3,99E-16	0,352905382	0,431	0,154	7,57E-12
ATP2A2	4,54E-16	0,572346107	0,451	0,178	8,60E-12
ADH5	4,93E-16	0,405919451	0,386	0,136	9,35E-12
RAB13	5,58E-16	0,419719298	0,314	0,099	1,06E-11
ACTG1	5,62E-16	0,399716285	0,974	0,81	1,07E-11
CRTAP	6,27E-16	0,719315649	0,641	0,338	1,19E-11
GABARAPL2	6,35E-16	0,459919678	0,471	0,184	1,20E-11
NDUFC1	6,90E-16	0,50651885	0,418	0,163	1,31E-11
SESTD1	9,72E-16	0,36109471	0,255	0,071	1,84E-11
COPS2	9,73E-16	0,459917808	0,327	0,109	1,84E-11
SESN3	1,12E-15	0,323220091	0,34	0,11	2,12E-11
JMJD8	1,36E-15	0,305043026	0,294	0,09	2,58E-11
PCYOX1	1,49E-15	0,479122162	0,484	0,199	2,83E-11
C12orf57	1,63E-15	0,503523858	0,588	0,273	3,08E-11
SLC25A4	1,77E-15	0,396683678	0,275	0,081	3,36E-11
REEP3	1,90E-15	0,456065723	0,314	0,104	3,61E-11
MAP7D3	2,08E-15	0,262110199	0,275	0,08	3,94E-11
SKI	2,55E-15	0,375431939	0,66	0,309	4,84E-11
ANK3	2,67E-15	0,379642413	0,301	0,093	5,05E-11
TUBB6	2,95E-15	0,366197297	0,261	0,076	5,59E-11
PRDX1	3,83E-15	0,359771435	0,595	0,263	7,26E-11
ARHGAP1	3,94E-15	0,427030934	0,438	0,17	7,47E-11
CBX6	4,78E-15	0,429812298	0,431	0,167	9,05E-11
NUCB1	5,07E-15	0,440193406	0,582	0,265	9,61E-11
JUNB	6,26E-15	0,720041241	0,725	0,437	1,19E-10
GPNMB	6,34E-15	0,291333071	0,399	0,148	1,20E-10
USP11	7,54E-15	0,394038378	0,386	0,14	1,43E-10
KDELR1	7,57E-15	0,386291067	0,425	0,166	1,43E-10
EEA1	8,39E-15	0,397396358	0,353	0,124	1,59E-10
TXNRD1	9,84E-15	0,305484868	0,314	0,103	1,87E-10
GLG1	1,20E-14	0,419002601	0,621	0,298	2,28E-10
RRBP1	1,30E-14	0,42918165	0,34	0,121	2,45E-10
ITM2B	1,34E-14	0,48133326	0,935	0,794	2,53E-10
SLC18A1	1,42E-14	0,385176858	0,405	0,151	2,70E-10
BCAP29	1,62E-14	0,484714333	0,346	0,124	3,06E-10
ANGPT2	1,88E-14	0,467461273	0,288	0,091	3,56E-10
ARHGAP21	1,92E-14	0,285611476	0,275	0,084	3,64E-10
VEZF1	1,98E-14	0,402860208	0,359	0,13	3,76E-10
F2R	2,04E-14	0,703895327	0,438	0,192	3,87E-10
AHNAK	2,10E-14	0,410682626	0,928	0,695	3,98E-10
EVI5	2,58E-14	0,346381662	0,301	0,099	4,89E-10
MYH9	2,72E-14	0,577709671	0,876	0,646	5,15E-10
SF3B6	2,74E-14	0,369769764	0,405	0,155	5,18E-10
YIPF6	2,99E-14	0,335694192	0,301	0,099	5,67E-10
NOTCH2	3,08E-14	0,392104094	0,34	0,121	5,83E-10
SIGMAR1	3,39E-14	0,275110965	0,301	0,097	6,43E-10
TMEM87A	4,03E-14	0,38382814	0,516	0,229	7,63E-10
RNF11	5,26E-14	0,374140774	0,379	0,143	9,97E-10
UBA2	5,49E-14	0,410040982	0,386	0,148	1,04E-09
TSPAN3	6,65E-14	0,377818981	0,353	0,131	1,26E-09
DYNLRB1	9,05E-14	0,363997203	0,575	0,27	1,71E-09
CDKN1A	1,05E-13	0,485587092	0,366	0,139	1,99E-09
TMED7	1,07E-13	0,419993794	0,327	0,118	2,03E-09
SPIN1	1,21E-13	0,280359303	0,301	0,099	2,29E-09
SERINC1	1,30E-13	0,409644211	0,693	0,361	2,46E-09
ISCU	1,34E-13	0,386671228	0,614	0,293	2,53E-09

GRHPR	1,55E-13	0,290719941	0,275	0,088	2,93E-09
HSP90B1	1,77E-13	0,436346249	0,712	0,411	3,35E-09
JUN	1,80E-13	0,606464795	0,412	0,167	3,42E-09
ZBTB20	2,00E-13	0,378280352	0,379	0,143	3,79E-09
TMEM30A	2,04E-13	0,448287351	0,51	0,229	3,86E-09
MYADM	2,24E-13	0,416497367	0,693	0,375	4,25E-09
TXNDC15	2,89E-13	0,349912075	0,373	0,142	5,48E-09
IGFBP4	3,51E-13	0,496807057	0,366	0,14	6,64E-09
SCOC	3,65E-13	0,373137903	0,261	0,084	6,92E-09
ITPRIPL2	4,01E-13	0,443370168	0,444	0,187	7,60E-09
TMEM165	4,05E-13	0,279787125	0,294	0,099	7,68E-09
CUX1	4,26E-13	0,323361544	0,451	0,188	8,08E-09
MXI1	4,29E-13	0,334165572	0,32	0,114	8,13E-09
SMAD7	5,40E-13	0,384676112	0,261	0,084	1,02E-08
ATF4	5,53E-13	0,396711598	0,608	0,296	1,05E-08
FAM210B	5,56E-13	0,351096261	0,458	0,191	1,05E-08
ANXA5	5,72E-13	0,489274871	0,725	0,414	1,08E-08
FAM129A	7,21E-13	0,502142681	0,575	0,285	1,37E-08
ATN1	8,00E-13	0,367374245	0,392	0,156	1,52E-08
SLC14A1	8,57E-13	0,586748659	0,268	0,091	1,62E-08
SETD7	8,84E-13	0,419103024	0,255	0,083	1,68E-08
TNFSF12	8,88E-13	0,300539761	0,399	0,156	1,68E-08
ARL2	9,02E-13	0,325234897	0,268	0,089	1,71E-08
BDH2	1,24E-12	0,300292251	0,275	0,092	2,36E-08
CHSY1	1,41E-12	0,40575174	0,314	0,115	2,67E-08
NFE2L2	1,64E-12	0,401917075	0,418	0,176	3,11E-08
BNIP3L	1,75E-12	0,380809588	0,471	0,206	3,31E-08
RAB1A	1,91E-12	0,353805198	0,431	0,184	3,62E-08
PHPT1	1,93E-12	0,340637736	0,346	0,132	3,66E-08
GSTP1	2,12E-12	0,469688429	0,562	0,281	4,01E-08
TMEM59	2,23E-12	0,321149276	0,66	0,341	4,22E-08
PLEC	2,43E-12	0,425452754	0,601	0,294	4,61E-08
TUBA1A	2,56E-12	0,268741851	0,497	0,219	4,84E-08
DIXDC1	2,59E-12	0,368230038	0,346	0,133	4,92E-08
NR4A1	3,23E-12	0,649250434	0,307	0,116	6,13E-08
RHOC	4,44E-12	0,331820088	0,366	0,145	8,41E-08
PDLIM2	5,72E-12	0,367819007	0,268	0,092	1,08E-07
TXNL1	7,12E-12	0,257483709	0,288	0,101	1,35E-07
RING1	8,14E-12	0,292136818	0,32	0,12	1,54E-07
ESYT2	8,28E-12	0,372595615	0,503	0,239	1,57E-07
sep-02	8,68E-12	0,400290846	0,667	0,359	1,64E-07
PRNP	9,39E-12	0,306404813	0,399	0,167	1,78E-07
SLIRP	1,08E-11	0,311821614	0,333	0,128	2,05E-07
COPS8	1,08E-11	0,323472611	0,314	0,115	2,05E-07
IL1R1	1,09E-11	0,316706408	0,261	0,089	2,07E-07
ARPC1A	1,29E-11	0,41507668	0,32	0,123	2,44E-07
FAM129B	1,55E-11	0,340652953	0,294	0,108	2,94E-07
NDUFA5	1,78E-11	0,306199151	0,359	0,145	3,38E-07
CDIPT	1,89E-11	0,291847191	0,366	0,15	3,58E-07
TMEM147	2,82E-11	0,257066602	0,327	0,127	5,34E-07
HSD17B12	2,96E-11	0,289678234	0,366	0,151	5,62E-07
ADAMTS2	3,27E-11	0,352370685	0,255	0,088	6,19E-07
CAMLG	3,46E-11	0,397170861	0,333	0,135	6,56E-07
CLTA	3,93E-11	0,372510422	0,359	0,146	7,45E-07
NDUFS4	3,95E-11	0,259218437	0,255	0,087	7,49E-07
MSRB2	4,64E-11	0,280836921	0,261	0,092	8,80E-07

TMEM109	4,95E-11	0,291317306	0,484	0,226	9,37E-07
MRPL34	4,97E-11	0,251573917	0,288	0,105	9,42E-07
MYL6	5,52E-11	0,403047496	0,915	0,713	1,05E-06
ALDH3A2	5,61E-11	0,344503658	0,346	0,143	1,06E-06
sep-07	5,88E-11	0,372537248	0,667	0,386	1,11E-06
RAB5B	6,19E-11	0,371889658	0,373	0,16	1,17E-06
PTPN11	7,76E-11	0,321986442	0,392	0,167	1,47E-06
PRKAR1A	7,89E-11	0,338083572	0,673	0,37	1,50E-06
RAB3GAP1	8,43E-11	0,285877977	0,268	0,097	1,60E-06
SPATS2L	9,28E-11	0,257250328	0,314	0,122	1,76E-06
CSNK1E	9,84E-11	0,277173372	0,34	0,138	1,86E-06
DCTN2	1,14E-10	0,325835592	0,34	0,14	2,16E-06
TOB2	1,17E-10	0,322458385	0,314	0,123	2,21E-06
CEBPD	1,26E-10	0,416994183	0,314	0,127	2,40E-06
DYNLT3	1,59E-10	0,272041838	0,261	0,095	3,02E-06
CCL2	1,67E-10	0,790164239	0,255	0,095	3,17E-06
UBE2H	1,76E-10	0,327244483	0,373	0,161	3,34E-06
DYNC1I2	1,83E-10	0,338612296	0,444	0,213	3,47E-06
AP3S1	1,97E-10	0,375312828	0,399	0,182	3,73E-06
C12orf75	2,13E-10	0,352064938	0,301	0,116	4,03E-06
HSP90AB1	2,21E-10	0,411812495	0,804	0,573	4,20E-06
ECE1	2,48E-10	0,274539214	0,386	0,166	4,69E-06
THBS1	2,53E-10	0,375653301	0,268	0,1	4,79E-06
GPX4	2,55E-10	0,277073564	0,556	0,268	4,83E-06
HEXB	3,41E-10	0,260878149	0,333	0,139	6,47E-06
SAV1	3,73E-10	0,255183876	0,307	0,122	7,06E-06
VEGFB	3,95E-10	0,365304042	0,294	0,118	7,49E-06
TAGLN2	5,28E-10	0,288995531	0,654	0,357	1,00E-05
SOD1	5,88E-10	0,286906059	0,641	0,36	1,11E-05
TMED10	5,94E-10	0,282036268	0,536	0,278	1,13E-05
BTG2	6,54E-10	0,375233513	0,294	0,119	1,24E-05
RTN4	7,78E-10	0,325811352	0,739	0,45	1,47E-05
ST13	7,85E-10	0,391330761	0,477	0,239	1,49E-05
ZBTB4	7,87E-10	0,335273448	0,412	0,188	1,49E-05
RAP2A	8,41E-10	0,298242483	0,261	0,1	1,59E-05
CEBPB	9,44E-10	0,252134292	0,314	0,129	1,79E-05
CRIP1	9,48E-10	0,377860269	0,725	0,454	1,80E-05
NDUFA4	9,97E-10	0,36294995	0,641	0,364	1,89E-05
ERGIC1	1,08E-09	0,310193599	0,314	0,131	2,04E-05
STX7	1,19E-09	0,284844522	0,281	0,112	2,26E-05
ANXA2	1,22E-09	0,331880213	0,673	0,397	2,32E-05
PJA2	1,34E-09	0,34469307	0,497	0,253	2,54E-05
PRAF2	1,34E-09	0,296620374	0,268	0,105	2,55E-05
YWHAQ	1,40E-09	0,297157872	0,667	0,372	2,64E-05
IPO7	1,47E-09	0,262817821	0,379	0,171	2,78E-05
PPP2CB	1,51E-09	0,338449182	0,294	0,121	2,86E-05
IMPAD1	1,68E-09	0,366073689	0,379	0,176	3,19E-05
ZMYND11	1,80E-09	0,264172691	0,392	0,176	3,41E-05
KCTD12	1,98E-09	0,430325776	0,412	0,196	3,76E-05
CTNNB1	2,26E-09	0,288577419	0,444	0,215	4,29E-05
MAF1	2,31E-09	0,275453564	0,399	0,184	4,38E-05
TMEM230	2,67E-09	0,261081422	0,438	0,208	5,06E-05
SKP1	2,99E-09	0,27459023	0,673	0,384	5,66E-05
DYNC1H1	3,49E-09	0,306120687	0,529	0,273	6,61E-05
P4HB	3,73E-09	0,268622386	0,595	0,317	7,08E-05
RABGAP1	4,10E-09	0,392658109	0,288	0,12	7,78E-05

NPC2	5,17E-09	0,255579272	0,601	0,312	9,79E-05
REEP5	6,01E-09	0,350315434	0,556	0,308	0,000113893
BMPR2	6,82E-09	0,252830297	0,464	0,227	0,000129172
NADK	9,60E-09	0,250326137	0,275	0,112	0,000181959
SH3BGRL	9,80E-09	0,270559676	0,614	0,337	0,000185721
ZFP36	1,04E-08	0,409375757	0,503	0,262	0,000197225
OS9	1,48E-08	0,262047204	0,516	0,271	0,000280126
LAMP1	1,50E-08	0,264540229	0,569	0,306	0,000284754
RDX	1,51E-08	0,261062691	0,34	0,157	0,000285836
LIN7C	2,39E-08	0,323671333	0,255	0,105	0,000452301
NDUFS5	2,41E-08	0,316700051	0,556	0,307	0,00045623
CNBP	2,54E-08	0,268492045	0,68	0,412	0,000481893
SLC44A1	3,49E-08	0,25451145	0,294	0,128	0,000661767
NDUFB7	4,05E-08	0,253832607	0,34	0,157	0,000768374
HIP1	4,09E-08	0,25612524	0,399	0,196	0,000774694
TMED2	4,56E-08	0,273855729	0,458	0,238	0,000864915
SMAD5	4,65E-08	0,310407376	0,359	0,172	0,000880394
TLN1	4,73E-08	0,285735757	0,725	0,449	0,000896455
PDIA3	6,52E-08	0,256396642	0,405	0,203	0,001236299
EIF4A2	7,59E-08	0,261577818	0,627	0,374	0,001439152
MYDGF	9,98E-08	0,253153033	0,275	0,121	0,001891886
SOX4	1,02E-07	0,44103269	0,268	0,122	0,001926744
KIF5B	1,03E-07	0,263511766	0,556	0,312	0,0019498
SRI	1,10E-07	0,256526629	0,353	0,169	0,002087457
IFI6	1,16E-07	0,546289824	0,458	0,246	0,002189107
ZBTB38	1,92E-07	0,258677551	0,373	0,188	0,003630603
TIMMDC1	2,53E-07	0,273242595	0,268	0,119	0,00479917
SEC62	3,26E-07	0,288898792	0,654	0,398	0,006176145
MRPS21	3,43E-07	0,257876774	0,359	0,179	0,006492583
WASF2	8,85E-07	0,260479628	0,608	0,371	0,016772955
GANAB	2,11E-06	0,254923396	0,333	0,172	0,039915105
CCNI	2,23E-06	0,274294142	0,712	0,488	0,042332713
MYLIP	2,77E-06	0,403148461	0,333	0,172	0,052446954
CANX	7,77E-06	0,254164722	0,667	0,436	0,147270376
C4orf3	7,98E-06	0,259261659	0,601	0,378	0,15129949
RTN3	9,28E-06	0,251880327	0,373	0,203	0,175871563
CCDC85B	1,53E-05	0,257082863	0,261	0,131	0,28910622
PPDPF	7,77E-05	0,272638107	0,712	0,49	1
APOE	0,000510002	0,543614356	0,255	0,149	1
NEK7	0,003680422	0,316242601	0,294	0,179	1

Cluster 9 marker genes

gene	p_val	avg_logFC	pct.1	pct.2	p_val_adj
GNG11	2,91E-165	1,673313799	0,593	0,036	5,51E-161
COL4A1	2,35E-152	1,900985131	0,696	0,061	4,45E-148
APLNR	7,69E-151	1,38399068	0,319	0,006	1,46E-146
VWA1	5,82E-143	1,520816397	0,444	0,02	1,10E-138
SHANK3	1,04E-139	1,629404006	0,511	0,031	1,98E-135
SPARCL1	1,09E-139	2,427033985	0,867	0,124	2,06E-135
PLVAP	1,10E-134	1,9192383	0,563	0,043	2,08E-130
NR2F2	3,13E-125	1,591015461	0,778	0,096	5,93E-121
ADGRL4	3,81E-124	1,731612208	0,526	0,041	7,22E-120
COL18A1	9,64E-123	1,703260959	0,674	0,072	1,83E-118
COL4A2	1,75E-113	1,951630747	0,726	0,1	3,31E-109
ESAM	2,96E-113	1,436067585	0,578	0,056	5,62E-109
DNASE1L3	1,37E-111	1,858313636	0,37	0,019	2,60E-107
LAMB1	3,84E-106	1,472674315	0,644	0,078	7,27E-102
TM4SF1	2,87E-102	1,888332599	0,748	0,118	5,43E-98
SPNS2	2,87E-102	0,786013708	0,267	0,009	5,44E-98
A2M	4,27E-102	1,939566886	0,793	0,137	8,09E-98
ROBO4	6,96E-101	0,934391803	0,37	0,022	1,32E-96
COL15A1	4,39E-100	2,098900098	0,548	0,058	8,31E-96
CLEC14A	8,79E-98	1,450269542	0,504	0,048	1,67E-93
NFIB	2,85E-97	1,492464184	0,785	0,131	5,40E-93
TM4SF18	4,30E-96	1,251142028	0,333	0,018	8,15E-92
ADAMTS9	5,55E-95	0,924647682	0,289	0,012	1,05E-90
SPARC	4,39E-94	1,855126172	0,881	0,201	8,32E-90
AQP1	3,52E-89	1,645260629	0,763	0,135	6,68E-85
PODXL	3,63E-89	1,378974622	0,459	0,043	6,88E-85
RASIP1	1,40E-88	0,952543696	0,333	0,02	2,65E-84
PLS3	1,05E-87	1,169986572	0,6	0,077	1,99E-83
CALCRL	1,68E-87	1,644153227	0,578	0,076	3,19E-83
ADGRL2	2,37E-87	1,074992601	0,311	0,017	4,49E-83
IGFBP4	8,70E-87	1,837351409	0,711	0,126	1,65E-82
ECSCR	2,04E-85	1,168997602	0,422	0,037	3,87E-81
CAV1	6,25E-85	1,660479494	0,733	0,131	1,18E-80
CDH5	3,25E-84	1,240096951	0,393	0,033	6,16E-80
SOX18	8,56E-84	1,302735677	0,407	0,036	1,62E-79
NID1	6,01E-83	1,275568173	0,415	0,038	1,14E-78
GRB10	5,18E-81	1,128072552	0,4	0,036	9,82E-77
RAMP2	3,04E-80	1,276376991	0,467	0,05	5,76E-76
CYYR1	1,62E-79	1,097823946	0,326	0,023	3,07E-75
SLCO2A1	1,83E-79	1,522578782	0,356	0,028	3,47E-75
HYAL2	5,24E-79	1,26570707	0,474	0,054	9,93E-75
KANK3	5,73E-79	0,773250532	0,274	0,015	1,09E-74
POSTN	1,59E-77	1,943583435	0,467	0,054	3,02E-73
CNN3	3,04E-77	1,042197099	0,481	0,057	5,76E-73
SNCG	6,31E-76	0,835762777	0,333	0,025	1,20E-71
TSPAN7	6,89E-76	1,333225954	0,37	0,033	1,31E-71
EPAS1	2,54E-75	1,617331735	0,815	0,201	4,82E-71
CAVIN1	9,36E-75	1,410815818	0,778	0,168	1,77E-70
ARHGAP29	1,67E-74	0,996141654	0,474	0,057	3,17E-70
CLEC3B	1,89E-74	1,315849605	0,422	0,044	3,58E-70
EGFL7	4,37E-72	1,462974636	0,437	0,051	8,27E-68
LDB2	8,98E-72	1,352728819	0,467	0,058	1,70E-67
DPYSL3	1,01E-71	1,132328938	0,481	0,061	1,91E-67

SEMA6A	3,19E-71	1,010366385	0,281	0,018	6,05E-67
WWTR1	1,52E-69	1,054595485	0,556	0,084	2,88E-65
PLPP3	3,97E-69	1,185773449	0,504	0,07	7,52E-65
APP	3,38E-68	1,359843851	0,696	0,144	6,41E-64
IFI27	4,39E-68	1,262776297	0,481	0,064	8,32E-64
LAMA5	5,18E-68	0,950476446	0,4	0,043	9,81E-64
TFPI	7,16E-68	1,020953842	0,459	0,058	1,36E-63
ACKR1	9,05E-67	2,028360445	0,422	0,052	1,72E-62
TCF4	3,18E-66	1,53496713	0,807	0,227	6,02E-62
IGFBP7	1,14E-65	1,781190531	0,919	0,375	2,16E-61
SERPINH1	3,31E-64	0,945022059	0,452	0,06	6,28E-60
TIE1	2,32E-63	1,312221378	0,444	0,06	4,39E-59
TJP1	5,06E-63	0,890682362	0,37	0,04	9,58E-59
LAMA4	2,01E-62	1,129428562	0,526	0,085	3,81E-58
RBPMS	8,15E-62	1,017308515	0,452	0,062	1,54E-57
PLAT	1,06E-61	2,140372113	0,444	0,065	2,01E-57
PXDN	1,18E-61	1,249056544	0,363	0,04	2,24E-57
CD34	1,40E-61	1,378067705	0,459	0,067	2,66E-57
SPTBN1	1,54E-61	1,231361166	0,807	0,221	2,92E-57
BCL6B	9,71E-61	0,867136375	0,259	0,019	1,84E-56
EMCN	1,18E-60	1,579678438	0,541	0,101	2,23E-56
TGM2	1,14E-59	1,136117641	0,526	0,086	2,17E-55
S100A16	1,25E-59	1,149108252	0,407	0,053	2,37E-55
GNG12	1,37E-59	0,914384263	0,481	0,072	2,60E-55
NNMT	1,42E-59	0,888455839	0,43	0,057	2,69E-55
PCDH17	5,06E-59	1,158565883	0,348	0,038	9,60E-55
SASH1	1,44E-58	1,172593008	0,548	0,1	2,74E-54
IL33	2,89E-58	1,25357017	0,467	0,072	5,47E-54
CRIP2	8,08E-58	1,265227395	0,674	0,156	1,53E-53
OLFM1	8,59E-58	0,931856723	0,296	0,028	1,63E-53
NFIA	1,04E-57	1,129425338	0,719	0,169	1,97E-53
CTTN	2,38E-56	1,015665011	0,407	0,056	4,51E-52
PRSS23	2,85E-56	1,035518347	0,504	0,085	5,39E-52
COL6A2	1,02E-55	1,237830803	0,585	0,117	1,94E-51
NUAK1	1,66E-55	1,151789404	0,378	0,049	3,14E-51
SPRY1	2,51E-55	1,079308979	0,363	0,044	4,76E-51
BCAR1	2,65E-55	0,725746215	0,289	0,027	5,02E-51
ZNF521	5,34E-55	0,82515013	0,252	0,02	1,01E-50
PALMD	2,25E-54	1,123627318	0,356	0,044	4,27E-50
LMNA	1,29E-53	1,229328225	0,844	0,278	2,45E-49
EPHB4	5,71E-53	0,953789454	0,296	0,031	1,08E-48
VWF	7,93E-53	1,70063818	0,533	0,107	1,50E-48
ADIRF	8,71E-53	1,237027058	0,593	0,126	1,65E-48
TEK	1,02E-52	0,847645146	0,267	0,024	1,94E-48
CLIC4	1,16E-52	1,142520303	0,615	0,137	2,19E-48
GSN	1,37E-52	1,213012107	0,867	0,298	2,59E-48
ADGRF5	2,46E-52	1,01720656	0,407	0,059	4,66E-48
RAMP3	6,71E-52	1,080434211	0,289	0,03	1,27E-47
ARAP3	1,55E-51	0,718081834	0,341	0,041	2,94E-47
FLT4	1,59E-51	0,789228995	0,252	0,022	3,02E-47
TSPAN18	5,05E-51	0,942992802	0,378	0,051	9,57E-47
TINAGL1	5,74E-51	0,928881232	0,526	0,099	1,09E-46
IFITM3	1,07E-50	1,416496323	0,941	0,495	2,03E-46
NPDC1	1,11E-50	1,059683164	0,481	0,087	2,09E-46
C11orf96	1,14E-50	1,638838691	0,407	0,062	2,15E-46
NELL1	1,20E-50	1,054815381	0,519	0,101	2,28E-46

CD59	1,23E-50	1,429446854	0,77	0,261	2,34E-46
ADCY4	1,41E-50	0,919581186	0,259	0,024	2,66E-46
DOCK6	2,07E-50	0,613179351	0,274	0,027	3,92E-46
ENG	2,46E-50	1,458625788	0,63	0,162	4,66E-46
EHD2	3,73E-50	0,970348627	0,474	0,085	7,07E-46
FXYD6	3,80E-50	1,249811659	0,333	0,042	7,19E-46
MALL	6,90E-50	0,993626927	0,333	0,042	1,31E-45
PDLIM1	2,93E-49	1,323579659	0,733	0,222	5,55E-45
TUBB6	1,16E-48	1,031770241	0,422	0,07	2,21E-44
HTRA1	1,72E-48	0,923229652	0,563	0,114	3,26E-44
IGFBP5	3,03E-48	2,166539956	0,615	0,166	5,75E-44
PRCP	3,50E-48	1,544546328	0,578	0,146	6,62E-44
MMRN1	3,78E-47	1,21713343	0,289	0,033	7,16E-43
THBD	1,16E-46	1,265414464	0,526	0,112	2,19E-42
FERMT2	5,14E-46	0,811526922	0,356	0,052	9,74E-42
LAMC1	3,05E-45	1,046886715	0,474	0,094	5,78E-41
MAGI1	4,39E-45	0,705904904	0,259	0,027	8,32E-41
CD93	4,59E-45	1,363254813	0,519	0,12	8,69E-41
CALD1	5,48E-45	1,135646276	0,622	0,154	1,04E-40
FAM198B	6,68E-45	1,186484989	0,519	0,113	1,27E-40
RBFOX2	1,04E-44	0,882824249	0,37	0,057	1,97E-40
SYNPO	1,65E-44	0,85140911	0,519	0,105	3,14E-40
MPDZ	2,92E-44	0,506491446	0,311	0,039	5,54E-40
FZD4	4,40E-44	1,029543787	0,378	0,061	8,33E-40
ITGA6	1,04E-43	1,128939166	0,489	0,102	1,98E-39
RAI14	3,52E-43	0,895685658	0,363	0,056	6,67E-39
HSPB1	6,87E-43	1,106746688	0,874	0,41	1,30E-38
sep-10	1,74E-42	0,694191618	0,319	0,044	3,29E-38
ID3	2,02E-42	1,113106094	0,407	0,073	3,83E-38
TIMP3	3,40E-42	1,135492814	0,43	0,081	6,44E-38
MYLK	4,24E-42	1,074442429	0,356	0,056	8,04E-38
ACTN1	5,27E-42	1,31210002	0,748	0,271	9,99E-38
SHC1	5,35E-42	1,148928182	0,578	0,15	1,01E-37
VIM	6,18E-42	0,979941848	0,985	0,823	1,17E-37
PDLIM3	1,35E-41	0,952512755	0,304	0,041	2,56E-37
FBN1	1,49E-41	0,821410621	0,356	0,056	2,83E-37
MCAM	4,08E-41	1,338159992	0,422	0,083	7,73E-37
LIMCH1	8,63E-41	0,787034822	0,415	0,076	1,63E-36
MMRN2	1,16E-40	1,27841649	0,526	0,128	2,19E-36
FSTL1	1,28E-40	0,91898765	0,481	0,102	2,43E-36
FGD5	1,59E-40	0,516234537	0,252	0,028	3,01E-36
ETS2	1,69E-40	1,198758786	0,474	0,106	3,20E-36
CD9	2,10E-40	1,16560191	0,659	0,198	3,98E-36
RHOJ	4,22E-40	1,022385457	0,444	0,092	7,99E-36
MYCT1	5,91E-40	0,951869501	0,319	0,047	1,12E-35
CD200	1,65E-39	0,820376085	0,252	0,03	3,13E-35
ID1	2,41E-39	1,302205552	0,407	0,079	4,57E-35
TMEM204	4,73E-39	0,854433146	0,378	0,067	8,97E-35
CAV2	1,26E-38	0,830372985	0,615	0,164	2,38E-34
MTUS1	1,41E-38	0,893386546	0,393	0,074	2,67E-34
TSC22D1	1,62E-38	0,86242595	0,43	0,088	3,08E-34
CLDN5	2,04E-38	0,882684333	0,252	0,03	3,87E-34
CFI	5,18E-38	0,721192317	0,267	0,035	9,82E-34
ERG	9,37E-38	0,885470607	0,363	0,065	1,77E-33
DSTN	1,05E-37	1,120295133	0,681	0,224	1,99E-33
DOCK1	2,04E-37	0,562581754	0,252	0,031	3,87E-33

CAVIN3	2,25E-37	0,967668729	0,407	0,081	4,27E-33
UACA	1,01E-36	1,020978027	0,496	0,12	1,91E-32
AGRN	1,45E-36	0,692657965	0,296	0,044	2,75E-32
LAMB2	1,50E-36	0,72326028	0,393	0,076	2,85E-32
PMP22	1,56E-36	0,917886698	0,415	0,087	2,95E-32
TCF7L1	1,70E-36	0,681919299	0,267	0,036	3,22E-32
COL6A1	2,26E-36	1,057494103	0,519	0,132	4,27E-32
TANC1	2,50E-36	0,645214034	0,259	0,034	4,73E-32
ADAMTS1	2,56E-36	0,880177536	0,326	0,053	4,85E-32
LRRC32	3,75E-36	0,658770546	0,281	0,04	7,11E-32
GJA1	9,89E-36	0,704220092	0,326	0,054	1,87E-31
LRP5	1,13E-35	0,581966246	0,259	0,035	2,15E-31
CXCL12	1,27E-35	1,162583818	0,378	0,072	2,40E-31
NCKAP1	1,28E-35	0,837411696	0,4	0,082	2,43E-31
INSR	2,04E-35	0,977241227	0,348	0,063	3,87E-31
CDC42BPB	3,42E-35	0,739822383	0,348	0,064	6,47E-31
MATN2	6,53E-35	0,717593897	0,296	0,046	1,24E-30
SELENON	6,60E-35	0,945327468	0,4	0,085	1,25E-30
ARHGAP23	1,87E-34	1,135750726	0,348	0,066	3,54E-30
BACE2	2,24E-34	0,857876814	0,37	0,073	4,24E-30
SULF2	5,01E-34	0,861196114	0,385	0,08	9,49E-30
JAG1	9,13E-34	0,675537422	0,37	0,072	1,73E-29
YBX3	1,29E-33	0,849953113	0,593	0,181	2,44E-29
APBB2	1,64E-33	0,623054213	0,296	0,048	3,11E-29
CD151	4,21E-33	0,848665939	0,6	0,18	7,99E-29
MARCKSL1	5,51E-33	0,974152834	0,385	0,083	1,04E-28
AFDN	7,35E-33	0,757432304	0,4	0,086	1,39E-28
EPS8	1,29E-32	0,855114656	0,311	0,055	2,44E-28
FAM107A	1,51E-32	0,757869872	0,341	0,064	2,85E-28
PTK2	1,69E-32	0,73142079	0,319	0,058	3,20E-28
LIMS2	1,94E-32	0,700926548	0,341	0,064	3,68E-28
RDX	1,96E-32	1,06860689	0,519	0,15	3,72E-28
MEF2C	2,86E-32	0,90521454	0,496	0,134	5,42E-28
MGST2	3,14E-32	0,788314076	0,341	0,066	5,96E-28
PDK4	3,89E-32	0,941624194	0,363	0,074	7,38E-28
CTNNA1	1,58E-31	0,835320616	0,526	0,147	3,00E-27
DOCK4	1,89E-31	0,548086812	0,252	0,037	3,58E-27
TPM1	2,57E-31	0,699855499	0,459	0,111	4,86E-27
PECAM1	2,70E-31	1,240535946	0,681	0,292	5,12E-27
EBF1	2,97E-31	0,717037734	0,311	0,056	5,63E-27
PKP4	2,99E-31	0,93137594	0,326	0,062	5,66E-27
KDR	3,56E-31	0,691568016	0,267	0,042	6,75E-27
SUN1	4,45E-31	0,751954843	0,385	0,085	8,43E-27
FOXC1	4,48E-31	0,702657619	0,385	0,082	8,49E-27
S1PR1	5,12E-31	0,850188003	0,333	0,065	9,70E-27
AKR1C3	7,46E-31	0,657704014	0,267	0,042	1,41E-26
ASAP2	1,25E-30	0,625042635	0,304	0,055	2,38E-26
RAB13	1,48E-30	0,811114215	0,407	0,096	2,81E-26
RCAN1	2,67E-30	0,826526637	0,341	0,071	5,05E-26
MYH10	3,59E-30	0,836010056	0,385	0,084	6,80E-26
MAP1B	5,35E-30	1,014480571	0,407	0,098	1,01E-25
LHFPL6	6,48E-30	0,800307904	0,378	0,083	1,23E-25
NFIC	7,47E-30	0,910122795	0,726	0,288	1,42E-25
SVIL	9,79E-30	0,640828187	0,363	0,077	1,86E-25
ECE1	1,57E-29	0,84202304	0,533	0,16	2,98E-25
FSCN1	1,61E-29	1,036885078	0,348	0,076	3,04E-25

JUP	2,24E-29	0,640900977	0,259	0,042	4,24E-25
TNFRSF1A	2,37E-29	0,759703613	0,437	0,112	4,49E-25
TGFB1I1	5,89E-29	0,635684083	0,296	0,054	1,12E-24
CPXM2	7,35E-29	0,820393499	0,304	0,057	1,39E-24
ACVRL1	9,56E-29	0,603814122	0,274	0,047	1,81E-24
FHL1	1,58E-28	0,961407987	0,422	0,109	2,99E-24
PHLDB1	2,07E-28	0,647775496	0,259	0,044	3,92E-24
PKIG	2,23E-28	0,654511	0,311	0,06	4,23E-24
IL6ST	2,25E-28	1,013814023	0,615	0,229	4,27E-24
MGLL	3,57E-28	0,730999892	0,319	0,065	6,77E-24
PLXND1	4,10E-28	0,721211267	0,437	0,114	7,78E-24
CTGF	6,90E-28	0,948163497	0,4	0,1	1,31E-23
MYL9	1,05E-27	1,355180742	0,422	0,111	1,98E-23
CFH	1,10E-27	0,50309349	0,452	0,116	2,08E-23
HDGFL3	1,26E-27	0,632131378	0,311	0,063	2,39E-23
NDRG1	1,56E-27	0,943080816	0,556	0,183	2,96E-23
FLNB	2,22E-27	0,721767193	0,422	0,109	4,20E-23
SERPING1	3,09E-27	0,778628084	0,467	0,127	5,86E-23
PTPRB	3,11E-27	0,538716981	0,311	0,061	5,89E-23
MCTP1	3,31E-27	0,835343555	0,259	0,046	6,26E-23
SOX4	5,88E-27	0,944432234	0,43	0,116	1,11E-22
NRGN	7,65E-27	0,512398118	0,267	0,047	1,45E-22
TGFBR2	9,04E-27	1,240594225	0,593	0,23	1,71E-22
ARHGEF12	1,08E-26	0,682780304	0,437	0,118	2,04E-22
PPFIBP1	1,10E-26	0,621183651	0,311	0,065	2,08E-22
PTMS	1,92E-26	0,832277952	0,622	0,227	3,63E-22
TGFBR3	2,11E-26	1,0084011	0,548	0,184	4,00E-22
CAVIN2	4,54E-26	0,934882772	0,304	0,064	8,60E-22
EMP1	5,69E-26	1,213304506	0,385	0,103	1,08E-21
TMEM47	1,68E-25	0,768730674	0,311	0,067	3,17E-21
TCEAL9	2,61E-25	0,629782885	0,319	0,071	4,94E-21
LAPTM4B	2,89E-25	0,515369307	0,274	0,053	5,47E-21
PBX1	3,34E-25	0,632238646	0,363	0,087	6,33E-21
LMO2	3,82E-25	0,671853276	0,393	0,101	7,25E-21
TRIM47	4,30E-25	0,536231965	0,252	0,046	8,15E-21
LRRC8A	4,83E-25	0,576058228	0,341	0,079	9,14E-21
PPIC	5,02E-25	0,472389455	0,259	0,048	9,51E-21
CCDC50	5,51E-25	0,686574133	0,437	0,125	1,04E-20
ADAM15	5,86E-25	0,837917937	0,289	0,062	1,11E-20
CYR61	9,99E-25	1,236416285	0,319	0,071	1,89E-20
MRTFB	1,09E-24	0,793807137	0,289	0,06	2,07E-20
CEMIP2	1,14E-24	0,974798257	0,378	0,099	2,16E-20
CDH13	1,50E-24	0,849133869	0,326	0,075	2,83E-20
CRIM1	2,07E-24	0,656337371	0,519	0,165	3,91E-20
PROS1	2,38E-24	0,520688658	0,259	0,049	4,51E-20
CDC42BPA	3,31E-24	0,571562077	0,304	0,066	6,27E-20
TPM4	3,38E-24	0,895819007	0,778	0,416	6,41E-20
ITGA5	6,12E-24	0,880792782	0,526	0,188	1,16E-19
FKBP1A	1,00E-23	1,034298594	0,793	0,451	1,89E-19
YWHAE	1,04E-23	0,765140429	0,644	0,26	1,97E-19
CLU	2,93E-23	0,873969482	0,622	0,255	5,56E-19
ITGA10	3,60E-23	0,690108158	0,267	0,055	6,82E-19
MARCKS	3,86E-23	0,75953712	0,6	0,221	7,31E-19
TSPAN3	1,36E-22	0,772603569	0,422	0,129	2,57E-18
JAM2	2,17E-22	0,813201086	0,296	0,069	4,11E-18
NECTIN2	2,26E-22	0,81630251	0,274	0,062	4,29E-18

SLC12A2	3,26E-22	0,56159036	0,333	0,083	6,18E-18
CCND1	4,86E-22	0,736372921	0,333	0,085	9,22E-18
SCARB2	4,94E-22	0,602703372	0,452	0,144	9,37E-18
RGS5	7,07E-22	1,62195161	0,356	0,102	1,34E-17
PPM1F	1,01E-21	0,470113866	0,319	0,078	1,91E-17
SLC6A2	1,09E-21	0,897384531	0,311	0,076	2,07E-17
TIMP2	1,15E-21	0,624647037	0,593	0,219	2,19E-17
PTPRM	1,27E-21	0,702276523	0,304	0,073	2,40E-17
TCF7L2	1,29E-21	0,47889076	0,348	0,089	2,44E-17
GUCY1A1	1,32E-21	1,110336925	0,304	0,076	2,50E-17
RNASE1	1,43E-21	0,600745999	0,57	0,213	2,70E-17
BCAT2	1,64E-21	0,673364158	0,267	0,058	3,11E-17
KCNN3	1,65E-21	0,64820456	0,304	0,073	3,12E-17
LEPROT	2,79E-21	0,801586322	0,526	0,195	5,29E-17
HEG1	2,99E-21	0,968750233	0,556	0,217	5,66E-17
VCL	4,70E-21	0,825286791	0,444	0,149	8,90E-17
DST	5,62E-21	0,782440392	0,378	0,114	1,07E-16
MGP	5,95E-21	0,58218551	0,874	0,555	1,13E-16
RBMS3	6,29E-21	0,531291444	0,311	0,076	1,19E-16
DPYSL2	6,67E-21	0,756154277	0,541	0,209	1,26E-16
TNS1	7,42E-21	0,916692319	0,4	0,126	1,41E-16
PDLIM7	8,42E-21	0,693882991	0,333	0,089	1,59E-16
BEX3	1,18E-20	0,809292967	0,356	0,098	2,23E-16
CYB5R3	1,23E-20	0,69258037	0,637	0,267	2,34E-16
RAB11A	2,15E-20	0,714576042	0,533	0,205	4,07E-16
AEBP1	2,75E-20	1,070910605	0,326	0,089	5,21E-16
RASA4	4,30E-20	0,894798384	0,467	0,161	8,15E-16
MAST4	4,90E-20	0,59836672	0,356	0,098	9,29E-16
NOTCH3	5,23E-20	1,222332622	0,259	0,061	9,91E-16
TNKS1BP1	5,93E-20	0,628372042	0,252	0,055	1,12E-15
MFGE8	8,18E-20	0,855990791	0,296	0,075	1,55E-15
DYNLL1	9,36E-20	0,680526678	0,711	0,339	1,77E-15
CSGALNACT1	1,01E-19	0,573454665	0,267	0,061	1,92E-15
SYPL1	1,01E-19	0,603309237	0,393	0,121	1,92E-15
SLC18A1	1,13E-19	0,706711626	0,444	0,151	2,14E-15
LIMA1	1,37E-19	0,743765024	0,348	0,098	2,60E-15
DOCK9	1,50E-19	0,655671588	0,341	0,095	2,84E-15
NOTCH1	2,02E-19	0,406106872	0,311	0,079	3,83E-15
FAM129B	2,99E-19	0,554619973	0,363	0,106	5,66E-15
FLT1	3,25E-19	0,883341647	0,422	0,145	6,16E-15
RAC1	3,90E-19	0,66429954	0,837	0,493	7,39E-15
PLXNA2	3,96E-19	0,643961974	0,259	0,06	7,50E-15
GRASP	5,34E-19	0,500060464	0,259	0,061	1,01E-14
ADGRG1	5,57E-19	0,333920663	0,289	0,07	1,06E-14
RTL8C	9,14E-19	0,577696638	0,43	0,147	1,73E-14
MPZL2	9,83E-19	0,606741228	0,274	0,066	1,86E-14
ZSCAN18	1,36E-18	0,378233364	0,274	0,065	2,59E-14
CRTAC1	1,55E-18	0,763710811	0,252	0,059	2,94E-14
HES1	1,99E-18	0,633762865	0,281	0,071	3,77E-14
TRIP10	3,06E-18	0,542852489	0,267	0,066	5,80E-14
NFE2L1	3,52E-18	0,590567811	0,4	0,133	6,67E-14
MYOF	3,82E-18	0,523167772	0,319	0,09	7,24E-14
ENDOD1	4,18E-18	0,489943043	0,252	0,06	7,92E-14
TRIOBP	4,53E-18	0,715024135	0,541	0,225	8,58E-14
PGRMC1	4,57E-18	0,673161755	0,326	0,097	8,66E-14
EPHX1	5,68E-18	0,58649363	0,259	0,064	1,08E-13

TIMP1	9,04E-18	0,631094119	0,822	0,509	1,71E-13
CDC42EP1	1,09E-17	0,523794863	0,267	0,068	2,07E-13
MRFAP1	1,17E-17	0,60052269	0,496	0,189	2,22E-13
CSNK1E	1,37E-17	0,529334116	0,407	0,136	2,59E-13
GAS6	1,79E-17	0,554913444	0,393	0,129	3,39E-13
CDK2AP1	1,90E-17	0,610407951	0,4	0,134	3,61E-13
EI24	2,02E-17	0,601640616	0,452	0,161	3,82E-13
LAPTM4A	2,11E-17	0,689027709	0,593	0,253	4,00E-13
ITM2B	2,22E-17	0,532851506	0,941	0,794	4,21E-13
HSPA1A	2,44E-17	0,549135578	0,393	0,128	4,63E-13
C1QTNF1	2,98E-17	0,52668746	0,311	0,089	5,65E-13
TPM2	3,59E-17	1,087604564	0,333	0,105	6,80E-13
EHD4	4,18E-17	0,491948764	0,259	0,066	7,93E-13
SNX9	5,03E-17	0,522142589	0,378	0,124	9,53E-13
TMEM184B	5,41E-17	0,470617452	0,304	0,086	1,03E-12
BMPR2	8,41E-17	0,644629248	0,533	0,226	1,59E-12
SLC29A1	1,09E-16	0,659500287	0,296	0,087	2,06E-12
TCEAL4	1,41E-16	0,673912457	0,319	0,097	2,67E-12
LGALS3	1,49E-16	0,529959604	0,57	0,232	2,82E-12
EFEMP1	2,04E-16	0,669961468	0,422	0,155	3,87E-12
RHOB	2,16E-16	0,597083584	0,326	0,1	4,09E-12
SNTB2	2,28E-16	0,623866328	0,57	0,245	4,31E-12
AMOTL2	2,45E-16	0,417507841	0,274	0,073	4,64E-12
RALGAPA2	2,51E-16	0,643077282	0,267	0,071	4,75E-12
EMP2	2,54E-16	0,628208194	0,57	0,251	4,82E-12
CCDC85B	2,98E-16	0,544600743	0,378	0,126	5,64E-12
PSMB5	3,46E-16	0,520320145	0,252	0,066	6,56E-12
HCFC1R1	3,67E-16	0,477452408	0,311	0,092	6,95E-12
PTPN14	4,45E-16	0,544312994	0,393	0,134	8,43E-12
GIMAP6	6,48E-16	0,647356683	0,304	0,091	1,23E-11
VAMP5	7,64E-16	0,379249138	0,304	0,088	1,45E-11
DUSP6	8,53E-16	0,826588856	0,407	0,151	1,62E-11
CCT6A	9,05E-16	0,546368347	0,489	0,194	1,72E-11
ATN1	9,46E-16	0,670686522	0,422	0,156	1,79E-11
RHOC	9,74E-16	0,571458187	0,407	0,145	1,85E-11
LTBP1	1,15E-15	1,109626246	0,304	0,097	2,19E-11
PTPN12	1,21E-15	0,563526764	0,474	0,186	2,29E-11
CPD	1,39E-15	0,638837561	0,319	0,1	2,63E-11
ARL2	1,44E-15	0,507988569	0,296	0,089	2,73E-11
CTNNB1	1,70E-15	0,718641447	0,504	0,214	3,22E-11
MMP2	2,19E-15	0,555108157	0,296	0,088	4,14E-11
FEZ2	2,78E-15	0,365921366	0,333	0,104	5,27E-11
FOS	3,06E-15	0,80080443	0,681	0,374	5,79E-11
MSRB3	3,33E-15	0,642211665	0,378	0,135	6,32E-11
KLHL42	3,81E-15	0,370285391	0,274	0,076	7,21E-11
ANXA2	6,50E-15	0,709361773	0,696	0,398	1,23E-10
ZMIZ1	7,02E-15	0,589701279	0,348	0,117	1,33E-10
EID1	7,74E-15	0,510308195	0,681	0,344	1,47E-10
BCAP29	7,83E-15	0,490032374	0,363	0,124	1,48E-10
MBNL2	8,92E-15	0,443575436	0,393	0,139	1,69E-10
PAM	1,12E-14	0,498482306	0,348	0,116	2,13E-10
NR4A1	1,23E-14	0,785536222	0,341	0,116	2,33E-10
REXO2	1,37E-14	0,379831388	0,319	0,101	2,60E-10
SELENOM	1,56E-14	0,590873234	0,341	0,113	2,95E-10
HDAC9	1,76E-14	0,69735875	0,437	0,175	3,33E-10
COL14A1	1,88E-14	0,82921394	0,289	0,092	3,57E-10

APOL1	1,98E-14	0,430223856	0,289	0,087	3,74E-10
SLC38A2	1,99E-14	0,609119577	0,467	0,191	3,78E-10
STOM	2,73E-14	0,689734414	0,667	0,374	5,18E-10
MYO1D	2,74E-14	0,473066371	0,267	0,078	5,19E-10
PTTG1IP	2,83E-14	0,477651246	0,556	0,243	5,36E-10
GPX3	3,44E-14	0,562281874	0,311	0,103	6,52E-10
PIK3C2A	4,44E-14	0,658791259	0,496	0,218	8,42E-10
RABGAP1	7,43E-14	0,335902687	0,348	0,118	1,41E-09
CD109	8,41E-14	0,514135395	0,341	0,116	1,59E-09
MXRA7	1,07E-13	0,500581583	0,407	0,154	2,03E-09
ENAH	1,48E-13	0,400030955	0,422	0,16	2,81E-09
BGN	1,49E-13	0,826862132	0,548	0,262	2,82E-09
RELL1	1,59E-13	0,460705249	0,267	0,081	3,00E-09
CD81	2,43E-13	0,494974271	0,807	0,517	4,61E-09
MAP3K20	2,79E-13	0,54106566	0,363	0,136	5,28E-09
PDLIM5	2,99E-13	0,633808576	0,526	0,257	5,68E-09
MYH11	3,42E-13	1,501580493	0,311	0,114	6,47E-09
PEA15	3,55E-13	0,541591021	0,541	0,25	6,74E-09
MEF2A	3,83E-13	0,425604073	0,407	0,159	7,26E-09
DAB2	3,97E-13	0,36593424	0,37	0,135	7,53E-09
FN1	5,02E-13	0,856512556	0,563	0,288	9,50E-09
ZEB1	5,17E-13	0,308260333	0,326	0,11	9,80E-09
NOS3	5,21E-13	0,421829892	0,259	0,077	9,87E-09
TSHZ2	5,58E-13	0,691237613	0,333	0,122	1,06E-08
KCTD10	6,19E-13	0,382899193	0,274	0,086	1,17E-08
CCDC47	6,36E-13	0,439591392	0,333	0,119	1,20E-08
ITGB1	6,38E-13	0,453956032	0,793	0,488	1,21E-08
SLC9A3R2	7,46E-13	0,396909118	0,393	0,148	1,41E-08
SNRK	8,42E-13	0,402667611	0,363	0,134	1,60E-08
NAB1	8,86E-13	0,320135783	0,281	0,09	1,68E-08
DKK3	9,52E-13	0,636830339	0,296	0,1	1,80E-08
ACTN4	1,12E-12	0,615941626	0,763	0,51	2,12E-08
COL3A1	1,13E-12	0,488179668	0,363	0,142	2,15E-08
TUBB2A	1,22E-12	0,47188045	0,711	0,395	2,31E-08
PALLD	1,36E-12	0,713019915	0,289	0,099	2,57E-08
AFF1	1,54E-12	0,338447004	0,37	0,137	2,92E-08
PIM3	1,93E-12	0,557174247	0,333	0,121	3,66E-08
KDELR2	2,66E-12	0,433507308	0,415	0,165	5,03E-08
SBDS	2,95E-12	0,327813369	0,296	0,099	5,60E-08
KLF4	3,34E-12	0,668277511	0,304	0,107	6,34E-08
ZFP36	3,59E-12	0,782329205	0,533	0,262	6,80E-08
YTHDF2	4,12E-12	0,358124441	0,4	0,159	7,81E-08
EGR1	4,67E-12	0,483190943	0,533	0,257	8,85E-08
ATP8B1	5,18E-12	0,571089134	0,281	0,096	9,81E-08
TAGLN	5,34E-12	1,419007	0,356	0,147	1,01E-07
MAPK3	5,78E-12	0,641625211	0,311	0,112	1,10E-07
AKT3	6,20E-12	0,297999752	0,296	0,1	1,18E-07
ZFP36L1	7,01E-12	0,41531683	0,837	0,549	1,33E-07
FLOT1	7,63E-12	0,540677602	0,511	0,244	1,45E-07
TMEM43	9,06E-12	0,413863643	0,407	0,166	1,72E-07
ARHGAP31	9,12E-12	0,379208641	0,326	0,12	1,73E-07
GIMAP7	9,48E-12	0,576903083	0,363	0,143	1,80E-07
MTRNR2L1	9,94E-12	0,697153287	0,763	0,47	1,88E-07
ROCK2	1,02E-11	0,478984263	0,407	0,171	1,93E-07
CKAP4	1,08E-11	0,418420842	0,267	0,088	2,05E-07
JUN	1,12E-11	0,794601834	0,393	0,17	2,12E-07

SORBS3	1,23E-11	0,300920088	0,311	0,11	2,33E-07
SLC40A1	1,24E-11	0,424687193	0,296	0,104	2,34E-07
THBS2	1,28E-11	0,933431694	0,259	0,088	2,42E-07
DYNC1I2	1,56E-11	0,379797409	0,474	0,213	2,96E-07
APLP2	1,69E-11	0,566053677	0,674	0,391	3,20E-07
TMEM30A	1,72E-11	0,432612017	0,496	0,232	3,25E-07
FNIP2	1,85E-11	0,492063312	0,252	0,084	3,51E-07
KLF7	2,12E-11	0,34114611	0,267	0,089	4,01E-07
HSD17B12	2,22E-11	0,380385766	0,378	0,152	4,20E-07
PMEPA1	2,41E-11	0,416695171	0,319	0,119	4,57E-07
FGFR1	2,62E-11	0,457109207	0,304	0,11	4,96E-07
FNDC3B	3,34E-11	0,319842091	0,378	0,15	6,34E-07
KLF2	4,25E-11	0,844421944	0,37	0,162	8,05E-07
DAD1	4,70E-11	0,380253329	0,541	0,253	8,91E-07
MYH9	4,87E-11	0,487829822	0,844	0,649	9,22E-07
MAN1A1	4,89E-11	0,547737384	0,356	0,144	9,27E-07
DLGAP4	5,31E-11	0,269649629	0,296	0,106	1,01E-06
SDCBP	6,10E-11	0,50387974	0,6	0,325	1,16E-06
CD63	6,47E-11	0,37312769	0,726	0,404	1,23E-06
ARF1	7,88E-11	0,520974493	0,696	0,402	1,49E-06
NAA38	9,75E-11	0,37893207	0,333	0,131	1,85E-06
NEDD9	9,80E-11	0,436985368	0,259	0,088	1,86E-06
HSP90AB1	1,04E-10	0,434671952	0,815	0,574	1,97E-06
FAM114A1	1,06E-10	0,459722971	0,252	0,086	2,01E-06
TLNRD1	1,38E-10	0,355056396	0,252	0,085	2,62E-06
RRBP1	1,58E-10	0,401625259	0,319	0,123	2,99E-06
C1R	1,85E-10	0,303142823	0,289	0,104	3,51E-06
PRXL2A	1,93E-10	0,462913105	0,289	0,108	3,66E-06
RAB18	1,99E-10	0,280691836	0,296	0,109	3,76E-06
CHSY1	2,01E-10	0,400844646	0,304	0,116	3,81E-06
RBMS2	2,72E-10	0,491175336	0,259	0,093	5,15E-06
DAAM1	2,76E-10	0,46653826	0,296	0,113	5,22E-06
ASAP1	3,13E-10	0,634782069	0,422	0,198	5,94E-06
DMWD	3,66E-10	0,320087103	0,274	0,098	6,94E-06
MAGED1	3,73E-10	0,31756702	0,274	0,1	7,07E-06
MAP4K4	3,91E-10	0,524435324	0,415	0,187	7,42E-06
F2R	3,96E-10	0,394263813	0,43	0,194	7,51E-06
RNF7	4,07E-10	0,284504867	0,37	0,154	7,71E-06
BRI3	4,38E-10	0,346675423	0,43	0,192	8,30E-06
MAP4	4,85E-10	0,349224369	0,43	0,189	9,20E-06
KLF9	5,18E-10	0,432394081	0,326	0,133	9,82E-06
SERINC3	5,73E-10	0,352895612	0,437	0,201	1,09E-05
HSP90B1	5,91E-10	0,483549284	0,681	0,414	1,12E-05
ASPH	6,28E-10	0,407025765	0,385	0,168	1,19E-05
PLTP	6,38E-10	0,531007943	0,43	0,199	1,21E-05
TACC1	6,69E-10	0,395399059	0,667	0,393	1,27E-05
GUK1	7,21E-10	0,347297492	0,526	0,258	1,37E-05
EEA1	7,29E-10	0,289488392	0,319	0,126	1,38E-05
RAB3GAP1	8,55E-10	0,42697465	0,267	0,098	1,62E-05
SLC44A1	1,07E-09	0,416468404	0,319	0,128	2,02E-05
SELENOW	1,08E-09	0,386962248	0,415	0,191	2,04E-05
MORF4L1	1,11E-09	0,388066211	0,696	0,406	2,10E-05
GALNT1	1,26E-09	0,402651475	0,378	0,167	2,38E-05
GPX8	1,57E-09	0,267038611	0,259	0,094	2,98E-05
ANGPT2	1,60E-09	0,941789776	0,252	0,093	3,04E-05
TUBA1B	1,65E-09	0,378077179	0,674	0,406	3,13E-05

MYO1C	1,92E-09	0,386280746	0,37	0,166	3,63E-05
DIPK2B	2,21E-09	0,444661725	0,43	0,204	4,19E-05
LPAR6	2,72E-09	0,287842538	0,333	0,137	5,15E-05
PPP2R2A	2,74E-09	0,341070123	0,289	0,113	5,18E-05
OGN	2,74E-09	1,104124268	0,319	0,143	5,20E-05
CCT3	2,88E-09	0,367788469	0,378	0,166	5,45E-05
SEC14L1	3,48E-09	0,466049614	0,326	0,14	6,59E-05
CYBRD1	3,88E-09	0,376658515	0,281	0,111	7,36E-05
SLC44A2	4,17E-09	0,488820411	0,481	0,249	7,91E-05
ACTA2	4,36E-09	1,364025224	0,43	0,228	8,25E-05
VAMP3	5,54E-09	0,321185513	0,333	0,144	0,000104964
VCP	5,85E-09	0,423449558	0,459	0,235	0,000110773
TAGLN2	5,87E-09	0,446478917	0,607	0,361	0,000111192
IRF2BP2	6,05E-09	0,38951532	0,474	0,241	0,000114726
SMC4	6,20E-09	0,346371577	0,356	0,154	0,000117567
ARID5B	6,21E-09	0,547364513	0,356	0,161	0,000117769
ATP1B3	6,39E-09	0,638534638	0,511	0,289	0,000121067
MAFG	7,10E-09	0,340114588	0,267	0,103	0,000134451
CYFIP1	7,57E-09	0,486152597	0,407	0,196	0,000143416
ITPRID2	8,56E-09	0,402922717	0,259	0,101	0,000162271
RAB2A	8,78E-09	0,340421058	0,415	0,197	0,000166321
DNAJC10	9,06E-09	0,270552359	0,348	0,152	0,000171601
RALB	9,30E-09	0,324625645	0,259	0,098	0,000176155
COPS2	9,54E-09	0,282261415	0,281	0,112	0,00018086
IPO7	1,02E-08	0,350499522	0,378	0,172	0,000194149
ATXN2	1,05E-08	0,327434658	0,319	0,135	0,000199493
COL1A2	1,15E-08	0,256190178	0,333	0,146	0,000217014
NAV1	1,19E-08	0,405725118	0,289	0,117	0,000226002
S100A10	1,29E-08	0,427774206	0,756	0,52	0,000244057
HNRNPA0	1,45E-08	0,400755889	0,422	0,209	0,000274955
FABP5	1,59E-08	0,574472444	0,348	0,161	0,000301734
CDC37	1,63E-08	0,362622232	0,481	0,251	0,000309165
PKD2	2,24E-08	0,438977318	0,333	0,147	0,000424616
MIDN	2,52E-08	0,341697384	0,496	0,255	0,00047666
SMARCE1	2,68E-08	0,44168814	0,444	0,228	0,0005082
OAT	2,74E-08	0,484347249	0,289	0,123	0,000518284
HDLBP	3,23E-08	0,298921301	0,459	0,227	0,000611876
TFG	3,53E-08	0,281002284	0,296	0,125	0,000669576
BANF1	3,55E-08	0,286256849	0,489	0,254	0,000671945
ERH	3,83E-08	0,366803777	0,385	0,183	0,000725757
DDAH2	3,88E-08	0,321606754	0,281	0,118	0,000735192
MORF4L2	4,67E-08	0,287118033	0,356	0,162	0,000884931
SEC11A	4,85E-08	0,256886566	0,437	0,215	0,000919877
DUSP1	5,07E-08	0,452881559	0,43	0,223	0,000960295
FAM210B	5,64E-08	0,258520528	0,407	0,195	0,00106806
WARS	6,57E-08	0,419013884	0,289	0,125	0,001244471
SOCS3	6,86E-08	0,726034488	0,378	0,199	0,001300066
SQSTM1	8,21E-08	0,357509977	0,541	0,295	0,001555592
TNFSF10	8,29E-08	0,355333617	0,363	0,172	0,00157114
FAM3C	8,78E-08	0,387537261	0,252	0,101	0,001663792
ZFHX3	8,93E-08	0,494742305	0,363	0,183	0,001692794
HSBP1	9,18E-08	0,404082166	0,311	0,139	0,001740393
MICAL2	9,23E-08	0,432697729	0,348	0,165	0,001749604
S100A6	9,56E-08	0,450236605	0,867	0,674	0,001810887
MPRIP	9,58E-08	0,375360667	0,363	0,173	0,001815103
MGST3	9,92E-08	0,257609004	0,459	0,235	0,001879452

DYNC1H1	1,05E-07	0,291823857	0,511	0,276	0,001989326
PARVA	1,09E-07	0,379922667	0,311	0,139	0,002060897
AP2S1	1,28E-07	0,396865697	0,437	0,227	0,002428984
TMEM35B	1,31E-07	0,276212713	0,356	0,166	0,002475799
PRKD3	1,34E-07	0,310404211	0,267	0,112	0,002539569
PRDX1	1,34E-07	0,344238653	0,504	0,269	0,00254452
CALR	1,55E-07	0,372349509	0,637	0,387	0,002929753
SEC31A	1,66E-07	0,39443844	0,415	0,21	0,003141581
TUBA1A	1,69E-07	0,430167501	0,415	0,224	0,003204913
SHROOM4	1,73E-07	0,306235051	0,348	0,164	0,003273412
ARHGEF2	1,84E-07	0,279608332	0,341	0,157	0,003486561
PPP3CA	1,90E-07	0,641946108	0,415	0,23	0,00359714
PPP1R15A	1,91E-07	0,297945262	0,333	0,154	0,003612603
LMAN1	2,23E-07	0,322946882	0,356	0,172	0,004223854
SERBP1	2,47E-07	0,323441393	0,607	0,377	0,00468729
ATP2A2	2,48E-07	0,373128611	0,37	0,183	0,004705818
EIF2S2	2,51E-07	0,357765223	0,511	0,283	0,004753741
TMEM14C	2,53E-07	0,293247245	0,311	0,144	0,004802026
STRN4	2,81E-07	0,270707017	0,326	0,151	0,005326783
ZNHIT1	2,90E-07	0,395256499	0,348	0,175	0,005494803
RGL2	3,28E-07	0,296927707	0,252	0,105	0,006216441
KIAA0355	3,42E-07	0,338245576	0,4	0,2	0,006475809
NCL	3,59E-07	0,385984059	0,519	0,304	0,006794241
SRP14	3,62E-07	0,471113438	0,674	0,439	0,00685643
ENY2	3,94E-07	0,400844638	0,296	0,136	0,007462637
RIPOR1	4,10E-07	0,296970988	0,296	0,137	0,007777331
CAMTA1	4,43E-07	0,401279529	0,304	0,142	0,008386908
PSMA3	5,05E-07	0,291606263	0,319	0,151	0,009567754
SEM1	5,08E-07	0,357221732	0,444	0,239	0,009622031
ADI1	5,15E-07	0,274216168	0,267	0,117	0,009765514
NASP	5,35E-07	0,25211719	0,267	0,115	0,010129225
PICALM	5,36E-07	0,32321452	0,415	0,219	0,010165164
PURA	5,79E-07	0,420950773	0,422	0,224	0,010980649
DAP	6,93E-07	0,273746923	0,289	0,131	0,013129286
CANX	7,11E-07	0,311481251	0,674	0,437	0,013465335
CALU	7,34E-07	0,26328782	0,393	0,198	0,013906455
PDIA3	7,78E-07	0,286958368	0,4	0,205	0,014747822
PFDN2	7,92E-07	0,27352418	0,304	0,139	0,015009387
GIMAP1	8,36E-07	0,375056427	0,319	0,148	0,015845095
AP2B1	8,62E-07	0,296357261	0,311	0,145	0,016344278
PRDX6	9,77E-07	0,303625531	0,459	0,253	0,018509643
FOXP1	1,00E-06	0,39750245	0,719	0,501	0,018958393
PDCD6	1,05E-06	0,380567827	0,274	0,124	0,019935975
NCOA7	1,07E-06	0,349795649	0,422	0,231	0,020201069
SCP2	1,07E-06	0,264611866	0,415	0,216	0,020240025
SGK1	1,10E-06	0,611580519	0,259	0,12	0,020845308
KDELR1	1,15E-06	0,363512324	0,341	0,171	0,021886253
CMIP	1,24E-06	0,569321121	0,519	0,353	0,02343014
PRELP	1,32E-06	0,437715226	0,511	0,306	0,02504986
NUCKS1	1,35E-06	0,290828488	0,719	0,488	0,02563631
SNAP23	1,37E-06	0,289876994	0,326	0,159	0,025921987
ZBTB20	1,41E-06	0,31955719	0,311	0,148	0,026630967
ANO6	1,52E-06	0,319934121	0,274	0,128	0,028775889
QKI	1,81E-06	0,268610913	0,489	0,275	0,034348665
KTN1	1,88E-06	0,335814478	0,6	0,385	0,035653554
MRPL33	1,92E-06	0,468330603	0,326	0,168	0,036432993

SPTAN1	1,97E-06	0,268419123	0,496	0,282	0,037238341
NORAD	1,98E-06	0,39905925	0,593	0,376	0,037490014
AHCYL1	2,16E-06	0,35856989	0,363	0,188	0,040858937
PRKACA	2,21E-06	0,256288859	0,311	0,152	0,041922643
PRKAR1A	2,43E-06	0,256903591	0,593	0,376	0,046126755
PSMA4	2,50E-06	0,307274295	0,356	0,186	0,047467778
RAB1A	2,67E-06	0,288851029	0,363	0,188	0,050563036
C1orf43	2,76E-06	0,307261127	0,415	0,229	0,05235467
PAPSS1	2,77E-06	0,263605799	0,267	0,122	0,052582762
NPTN	2,78E-06	0,269196909	0,333	0,166	0,052683002
ANXA5	2,80E-06	0,254011395	0,659	0,418	0,052973441
CSRP1	2,93E-06	0,417220811	0,363	0,2	0,055510879
ACTG1	2,95E-06	0,320790466	0,941	0,813	0,055871711
SKI	3,00E-06	0,311161517	0,541	0,316	0,056841525
GNG5	3,12E-06	0,264941165	0,481	0,275	0,059057477
PRICKLE4	3,17E-06	0,31407451	0,526	0,31	0,060130306
LPP	3,30E-06	0,417798418	0,652	0,45	0,062549211
PEAK1	3,43E-06	0,266219646	0,252	0,114	0,06493934
NUCB1	3,43E-06	0,27098379	0,474	0,271	0,065035205
LRRC75A-AS1	3,46E-06	0,291468663	0,556	0,335	0,065479723
ARGLU1	3,59E-06	0,339451516	0,556	0,349	0,068093314
TOMM22	3,91E-06	0,291568167	0,274	0,13	0,074087157
PCNP	4,14E-06	0,262207122	0,437	0,235	0,078369923
CLTA	4,27E-06	0,309773961	0,304	0,15	0,080935399
SEC61A1	4,44E-06	0,31120126	0,333	0,171	0,084150035
PTPA	5,00E-06	0,278115673	0,289	0,14	0,094840839
ATP1A1	5,48E-06	0,292747205	0,437	0,241	0,103898723
FBLIM1	5,50E-06	0,283126896	0,511	0,306	0,10419613
TMEM173	5,78E-06	0,288682273	0,452	0,256	0,109439277
MAPRE2	5,97E-06	0,284600486	0,444	0,252	0,113182933
HMGN3	6,13E-06	0,315654432	0,341	0,179	0,116082781
MYL6	6,48E-06	0,332040157	0,837	0,717	0,122737169
sep-02	6,57E-06	0,362059793	0,57	0,365	0,124551745
KCTD12	6,60E-06	0,30935521	0,37	0,199	0,125029319
TIPRL	6,68E-06	0,285876671	0,252	0,117	0,126498843
PLEC	6,89E-06	0,329521065	0,496	0,3	0,130504725
MAGED2	6,94E-06	0,350694812	0,289	0,145	0,131462821
RAB14	7,35E-06	0,287871934	0,363	0,198	0,139334237
VCAN	8,24E-06	0,660688508	0,341	0,189	0,156219902
YWHAG	8,30E-06	0,357321367	0,319	0,167	0,157265252
WSB1	8,39E-06	0,412893315	0,6	0,385	0,159062101
SERPINE1	9,18E-06	0,75343202	0,274	0,143	0,174007971
NDUFS5	9,79E-06	0,305362602	0,519	0,31	0,185545687
YWHAH	1,05E-05	0,253320415	0,333	0,174	0,199067318
CCNL2	1,17E-05	0,345802053	0,393	0,217	0,221273689
CUX1	1,45E-05	0,304095931	0,356	0,194	0,274707044
SLIRP	1,66E-05	0,316855642	0,267	0,132	0,315177661
CALCOCO2	1,95E-05	0,327032528	0,319	0,17	0,369433789
SH2B3	2,16E-05	0,250646331	0,274	0,136	0,41022488
USP22	2,32E-05	0,45360432	0,4	0,236	0,439655684
METAP2	2,36E-05	0,260338775	0,363	0,199	0,447973693
C5orf24	2,36E-05	0,277082571	0,363	0,2	0,44800459
RSU1	2,82E-05	0,321975884	0,43	0,254	0,534420551
VAPA	2,84E-05	0,291247631	0,422	0,25	0,5387131
EIF4A2	3,07E-05	0,326935065	0,585	0,377	0,58173722
UBE2J1	3,20E-05	0,287924862	0,259	0,13	0,607061717

APOE	4,26E-05	0,777616525	0,274	0,149	0,807949338
HEXB	4,31E-05	0,379677287	0,274	0,142	0,816831749
ALDH9A1	4,34E-05	0,346667915	0,274	0,144	0,821883
SLC3A2	4,35E-05	0,327571257	0,296	0,157	0,823560664
RTN4	4,36E-05	0,287781021	0,667	0,455	0,826614665
MYO6	4,97E-05	0,312675226	0,274	0,143	0,942662916
LRRC8C	5,08E-05	0,311963003	0,259	0,132	0,962410986
PCBP1	5,23E-05	0,294008642	0,696	0,483	0,991005648
JUNB	6,82E-05	0,302341849	0,637	0,443	1
BSG	7,72E-05	0,289238567	0,422	0,258	1
HGSNAT	9,74E-05	0,263756571	0,252	0,13	1
IFITM2	9,75E-05	0,348374082	0,874	0,819	1
DEGS1	0,000109923	0,260489246	0,333	0,191	1
NOL7	0,000118553	0,269440759	0,259	0,138	1
PSMD1	0,000133639	0,269544272	0,311	0,177	1
SERPINB6	0,00015816	0,253432288	0,37	0,223	1
CSNK1A1	0,000170963	0,331461845	0,259	0,142	1
ILF2	0,000180135	0,260395825	0,311	0,173	1
MAFB	0,000181807	0,520137451	0,289	0,164	1
ZNF24	0,00019737	0,276592838	0,274	0,147	1
PSMD2	0,000216374	0,264634349	0,356	0,211	1
EIF4H	0,000231953	0,294368816	0,556	0,379	1
LTBP4	0,000256466	0,576858312	0,274	0,163	1
ITPR3	0,00032795	0,257187201	0,252	0,134	1
ESD	0,000359244	0,301168296	0,296	0,172	1
VEZF1	0,000393305	0,253691458	0,252	0,136	1
PPP2CA	0,000481894	0,287405695	0,341	0,214	1
IER2	0,000505435	0,337518498	0,348	0,213	1
CAPZA2	0,000562295	0,336667883	0,37	0,232	1
PEPD	0,000622573	0,263334579	0,259	0,145	1
NSFL1C	0,000632144	0,279818597	0,252	0,138	1
MYADM	0,00107472	0,335061391	0,526	0,384	1
SH3GLB1	0,001080336	0,360450331	0,363	0,235	1
FLNA	0,001158292	0,381486096	0,696	0,515	1
METTL7A	0,004692705	0,275729723	0,348	0,238	1

Cluster 10 marker genes

gene	p_val	avg_logFC	pct.1	pct.2	p_val_adj
GJA5	2,81E-243	1,894323401	0,63	0,021	5,33E-239
MPZL2	2,18E-231	2,147906794	0,778	0,044	4,12E-227
SELP	1,66E-218	2,061110501	0,696	0,035	3,14E-214
ECSCR	3,06E-218	1,333209187	0,652	0,027	5,79E-214
BMX	3,97E-205	1,358175265	0,481	0,012	7,53E-201
SEMA3F	8,49E-202	1,117068578	0,481	0,013	1,61E-197
CLEC14A	1,04E-194	1,690481918	0,689	0,04	1,97E-190
PROCR	2,17E-194	1,73361726	0,689	0,042	4,10E-190
FGF18	1,63E-193	1,328662119	0,407	0,008	3,10E-189
CPAMD8	1,12E-185	1,299504338	0,474	0,015	2,12E-181
IFI27	1,69E-184	1,882426372	0,741	0,053	3,20E-180
PTPRB	8,57E-180	1,914092182	0,674	0,045	1,62E-175
VWF	2,13E-176	2,835720798	0,859	0,093	4,04E-172
CPXM2	2,28E-176	1,472148278	0,659	0,042	4,32E-172
PODXL	9,90E-176	1,593375699	0,622	0,036	1,88E-171
NDRG4	1,49E-175	1,091611436	0,444	0,013	2,83E-171
SULF1	5,23E-171	2,65512459	0,896	0,107	9,91E-167
ANXA3	5,97E-169	0,859271007	0,348	0,006	1,13E-164
CDH5	3,55E-167	1,138983887	0,548	0,026	6,72E-163
CLDN5	1,07E-166	1,659456484	0,489	0,02	2,03E-162
CD34	2,47E-163	1,767917593	0,711	0,057	4,69E-159
ID1	7,92E-162	2,030980703	0,741	0,065	1,50E-157
LIMS2	2,04E-160	1,645250964	0,667	0,05	3,87E-156
AIF1L	2,87E-160	0,956193287	0,393	0,011	5,43E-156
PLXNA2	4,49E-156	1,427187134	0,63	0,044	8,50E-152
EGFL7	2,47E-155	1,278845993	0,63	0,042	4,68E-151
SOX18	1,18E-152	1,290009324	0,541	0,03	2,24E-148
RAMP2	1,49E-152	1,707037561	0,615	0,044	2,81E-148
DKK2	1,07E-151	1,639560862	0,444	0,018	2,02E-147
BMP6	2,23E-147	1,076720881	0,378	0,012	4,23E-143
PALMD	1,08E-146	1,110912947	0,563	0,035	2,06E-142
COL8A1	3,89E-146	1,562321008	0,622	0,047	7,37E-142
ACVRL1	1,03E-142	1,25699826	0,548	0,035	1,95E-138
ITGB4	1,15E-141	1,145003348	0,452	0,021	2,18E-137
SMAD6	1,19E-140	1,167613525	0,489	0,027	2,25E-136
LMO2	3,58E-139	1,832507706	0,756	0,086	6,79E-135
RUNX1T1	7,17E-139	1,428479673	0,593	0,044	1,36E-134
C1R	9,19E-138	1,48659303	0,778	0,083	1,74E-133
TGM2	1,00E-136	1,959914772	0,726	0,077	1,89E-132
MTUS1	2,63E-133	1,649588085	0,659	0,062	4,98E-129
CAV1	1,52E-132	2,100396745	0,859	0,125	2,87E-128
FOXC1	1,76E-132	1,314646386	0,696	0,068	3,33E-128
TSPAN7	6,01E-131	1,111866047	0,481	0,028	1,14E-126
EDN1	1,92E-129	2,278761952	0,541	0,041	3,65E-125
HYAL2	9,38E-129	1,876320987	0,585	0,049	1,78E-124
ELN	3,51E-128	2,557585648	0,785	0,108	6,64E-124
CALCRL	2,16E-125	1,642175757	0,681	0,072	4,09E-121
NES	2,54E-125	1,174495338	0,496	0,033	4,81E-121
CPE	2,94E-125	1,548529359	0,57	0,046	5,57E-121
SLPI	1,49E-123	1,136666048	0,259	0,005	2,82E-119
MMP28	1,62E-122	0,678569568	0,296	0,008	3,07E-118
RBMS3	1,71E-122	1,245381631	0,637	0,062	3,25E-118
RHOB	1,56E-121	1,829849222	0,704	0,084	2,96E-117

CRTAC1	2,10E-121	1,834627072	0,556	0,046	3,98E-117
MEDAG	4,74E-121	1,002693429	0,459	0,028	8,99E-117
OMD	2,97E-120	1,779466496	0,615	0,059	5,63E-116
MALL	6,36E-120	1,170468061	0,496	0,035	1,21E-115
FAM198B	6,58E-117	1,718323925	0,748	0,104	1,25E-112
CAVIN1	5,32E-116	1,716162132	0,904	0,163	1,01E-111
TM4SF1	5,84E-116	1,696679353	0,8	0,116	1,11E-111
CRIM1	3,38E-115	2,294250415	0,844	0,151	6,40E-111
FAM107A	6,34E-113	1,800164269	0,57	0,054	1,20E-108
RAMP3	2,51E-111	1,052028044	0,415	0,024	4,76E-107
ASS1	1,54E-110	1,42398371	0,541	0,049	2,91E-106
CAVIN2	6,68E-110	1,31573913	0,563	0,053	1,27E-105
MECOM	8,51E-109	1,286430072	0,541	0,049	1,61E-104
NFIB	1,15E-108	1,47637517	0,83	0,129	2,17E-104
AQP1	2,11E-108	1,640643525	0,83	0,132	3,99E-104
MANSC1	2,14E-107	0,750075619	0,415	0,026	4,06E-103
MMRN2	2,77E-107	1,611532711	0,77	0,118	5,25E-103
WWTR1	1,87E-106	1,436162058	0,659	0,079	3,54E-102
GAS6	1,93E-106	1,489480419	0,763	0,113	3,66E-102
TEK	2,56E-106	0,766134114	0,37	0,02	4,86E-102
CLEC3B	3,68E-106	1,319442954	0,496	0,041	6,98E-102
CLU	6,51E-106	2,456357413	0,933	0,241	1,23E-101
HMCN1	2,41E-104	1,301874607	0,548	0,053	4,57E-100
FLNB	2,50E-104	1,364350723	0,711	0,097	4,74E-100
BCAT2	2,07E-103	1,119862694	0,519	0,047	3,93E-99
GJA1	3,64E-103	1,301381019	0,511	0,046	6,90E-99
ERG	2,36E-102	1,167608661	0,556	0,057	4,47E-98
NPR1	2,55E-102	0,517104618	0,296	0,011	4,83E-98
AHNAK2	3,12E-101	0,980758394	0,43	0,031	5,91E-97
SHANK3	3,29E-101	0,880549762	0,452	0,034	6,24E-97
FXYD6	1,26E-100	1,185631383	0,459	0,037	2,39E-96
MCAM	6,39E-100	1,288071749	0,63	0,074	1,21E-95
DIPK1B	1,12E-99	0,564886113	0,311	0,014	2,12E-95
PCDH10	1,24E-99	1,077787298	0,333	0,017	2,35E-95
SYNPO	3,00E-99	1,322517903	0,704	0,098	5,68E-95
LAMB1	4,67E-99	1,231743554	0,637	0,078	8,85E-95
PTGIS	2,15E-98	1,721953692	0,748	0,123	4,08E-94
NNMT	4,49E-98	1,066006663	0,533	0,053	8,50E-94
CDC42BPA	5,21E-98	0,894686931	0,548	0,056	9,88E-94
EMP1	1,14E-97	1,426522512	0,667	0,091	2,16E-93
ESAM	2,31E-97	1,020555751	0,556	0,057	4,37E-93
LIFR	5,95E-97	0,906814105	0,4	0,028	1,13E-92
TSPAN6	7,48E-97	0,659864581	0,304	0,014	1,42E-92
CCM2L	7,57E-97	0,653745141	0,289	0,012	1,44E-92
ADIRF	1,52E-95	1,226178889	0,778	0,119	2,88E-91
MYCT1	1,66E-95	0,877497526	0,474	0,041	3,15E-91
GATA2	8,50E-94	0,672691052	0,459	0,038	1,61E-89
JAG1	1,99E-93	1,119470444	0,563	0,064	3,77E-89
EPAS1	2,09E-93	1,694261918	0,889	0,197	3,95E-89
CFI	6,87E-93	0,842161464	0,4	0,029	1,30E-88
MMP2	7,16E-93	1,451519421	0,593	0,075	1,36E-88
RASIP1	7,01E-91	0,678478201	0,341	0,02	1,33E-86
EPHB4	1,20E-90	0,650888673	0,385	0,027	2,27E-86
NPDC1	8,82E-90	1,124506034	0,622	0,081	1,67E-85
LIMCH1	1,18E-89	1,142816889	0,578	0,069	2,24E-85
ITGA10	2,10E-88	1,014253772	0,474	0,046	3,99E-84

CYP1B1	3,13E-88	1,597504663	0,511	0,056	5,93E-84
TSPAN9	6,27E-88	0,614193	0,341	0,021	1,19E-83
C1S	6,54E-88	1,319445296	0,622	0,083	1,24E-83
SRPX	1,80E-87	0,832966909	0,304	0,016	3,42E-83
IGFBP4	5,63E-87	1,40327356	0,733	0,126	1,07E-82
GATA6	1,04E-86	0,818094059	0,407	0,033	1,97E-82
TCN2	5,21E-86	0,893648345	0,37	0,027	9,87E-82
TBX1	5,42E-86	0,893076704	0,407	0,033	1,03E-81
BGN	7,65E-86	1,60838808	0,926	0,246	1,45E-81
LMCD1	1,08E-85	0,741012116	0,37	0,027	2,04E-81
PTPRF	5,76E-85	0,713510278	0,385	0,029	1,09E-80
CD59	7,29E-85	1,791197266	0,896	0,255	1,38E-80
FBLN2	1,58E-84	1,505388678	0,504	0,057	3,00E-80
IL33	2,37E-84	1,268312209	0,556	0,069	4,49E-80
IL1R1	3,40E-84	1,185283512	0,578	0,076	6,44E-80
ADAMTS1	1,69E-83	1,465152247	0,459	0,047	3,20E-79
TIE1	3,07E-83	1,032819886	0,511	0,057	5,81E-79
PTPRG	6,92E-83	0,699943827	0,296	0,017	1,31E-78
HEG1	1,28E-82	2,281493284	0,822	0,205	2,43E-78
ID3	1,55E-82	1,047401688	0,548	0,067	2,95E-78
LDB2	2,31E-82	1,176514983	0,504	0,057	4,38E-78
LIMA1	2,92E-82	1,150440301	0,607	0,087	5,53E-78
PPIC	3,29E-82	1,027768028	0,43	0,041	6,24E-78
PTPRM	4,97E-82	0,992525501	0,533	0,064	9,42E-78
CYR61	4,99E-82	1,742249101	0,519	0,063	9,46E-78
S1PR1	6,20E-82	0,947745409	0,511	0,058	1,17E-77
COX7A1	9,36E-82	0,800970839	0,4	0,034	1,77E-77
CFH	1,40E-81	1,126969473	0,681	0,106	2,65E-77
NFIA	2,12E-81	1,377593908	0,8	0,166	4,02E-77
FSTL1	3,68E-81	1,035959883	0,652	0,094	6,98E-77
CYYR1	3,89E-81	0,671640683	0,333	0,022	7,37E-77
PLXNA4	5,68E-81	0,659143579	0,333	0,022	1,08E-76
ADGRL4	6,77E-81	0,801813971	0,452	0,044	1,28E-76
ARAP3	9,44E-80	0,851022703	0,415	0,038	1,79E-75
ARHGAP23	1,17E-79	0,861519281	0,511	0,059	2,22E-75
EFEMP1	1,38E-79	2,194834883	0,726	0,142	2,62E-75
TMEM47	2,26E-79	0,860814435	0,511	0,058	4,27E-75
PCDH17	4,93E-79	1,218977901	0,4	0,036	9,34E-75
S100A16	7,70E-79	0,752637591	0,474	0,05	1,46E-74
THBD	8,89E-79	1,548594152	0,644	0,106	1,68E-74
PLPP3	9,53E-78	1,115348367	0,533	0,069	1,81E-73
PDLIM1	6,33E-77	1,526719499	0,859	0,217	1,20E-72
CD151	8,58E-77	1,25694901	0,8	0,172	1,63E-72
NOS3	1,00E-76	1,26410232	0,511	0,066	1,90E-72
BMP4	1,02E-76	2,086139836	0,57	0,087	1,94E-72
PECAM1	1,06E-76	1,912351877	0,874	0,284	2,00E-72
SULF2	2,89E-76	0,931865327	0,548	0,073	5,47E-72
S100A13	3,84E-76	0,740156917	0,437	0,045	7,27E-72
NTN1	1,17E-75	0,458608724	0,252	0,012	2,22E-71
CDH11	2,10E-74	0,780826431	0,37	0,032	3,98E-70
IQCK	5,19E-74	0,582596916	0,267	0,015	9,83E-70
LTBP2	9,93E-74	1,129423318	0,674	0,113	1,88E-69
ADCY4	1,17E-73	0,590790144	0,311	0,022	2,21E-69
CLIC4	1,36E-73	1,091419673	0,711	0,133	2,58E-69
AMOTL2	4,68E-73	1,125827745	0,496	0,064	8,87E-69
JCAD	2,54E-72	0,829552692	0,385	0,037	4,81E-68

EFNB2	6,67E-72	0,83219622	0,341	0,028	1,26E-67
CCDC3	9,13E-72	0,839983454	0,385	0,037	1,73E-67
CAV2	1,48E-71	1,122157642	0,756	0,158	2,81E-67
PIK3R3	1,80E-71	0,561512471	0,304	0,021	3,40E-67
APP	2,15E-71	1,267732516	0,711	0,143	4,08E-67
NRN1	2,68E-71	0,6669494	0,304	0,022	5,08E-67
ABI3BP	4,86E-71	0,884683442	0,356	0,031	9,21E-67
BACE2	2,86E-70	0,847600269	0,511	0,067	5,42E-66
AGRN	5,13E-70	0,815254818	0,393	0,04	9,72E-66
MAST4	8,34E-70	1,091758149	0,578	0,088	1,58E-65
EXT1	9,46E-70	0,9740997	0,459	0,057	1,79E-65
HSD17B12	1,45E-69	1,226541644	0,689	0,138	2,75E-65
NCKAP1	1,48E-69	0,913383583	0,533	0,076	2,81E-65
CRIP2	1,49E-69	1,410923545	0,719	0,154	2,82E-65
PKIG	2,20E-69	0,868673001	0,452	0,054	4,16E-65
ITLN1	9,11E-69	4,202665895	0,593	0,113	1,73E-64
CALD1	3,42E-68	0,914974032	0,756	0,149	6,48E-64
BCL6B	5,42E-68	0,663708908	0,274	0,018	1,03E-63
WLS	6,38E-68	0,729832234	0,407	0,044	1,21E-63
ROBO4	8,46E-68	0,656455837	0,311	0,024	1,60E-63
NUAK1	9,39E-68	0,928882159	0,422	0,047	1,78E-63
LTC4S	6,17E-67	0,913066026	0,474	0,062	1,17E-62
ENG	1,11E-66	1,614445253	0,704	0,159	2,10E-62
SLC9A3R2	1,53E-66	1,310586867	0,674	0,136	2,91E-62
TANC1	2,01E-66	0,694103709	0,341	0,031	3,80E-62
RAPGEF3	2,23E-66	0,414068804	0,252	0,015	4,23E-62
LAMB2	4,30E-66	0,76659142	0,511	0,071	8,14E-62
CMAHP	8,67E-66	0,854648766	0,444	0,055	1,64E-61
FGD5	1,12E-65	0,689645902	0,311	0,026	2,13E-61
FERMT2	1,43E-65	0,690507326	0,422	0,049	2,71E-61
CYBRD1	1,80E-65	0,984868369	0,585	0,098	3,42E-61
ACKR3	3,53E-65	0,979763865	0,274	0,019	6,68E-61
CDH13	1,55E-64	1,00997098	0,489	0,068	2,94E-60
MRC2	1,67E-64	0,717950958	0,407	0,046	3,16E-60
ECE1	1,00E-63	1,175304113	0,704	0,153	1,90E-59
RNASE1	1,66E-63	1,360258337	0,778	0,204	3,15E-59
PCDH7	4,60E-63	0,941370057	0,467	0,063	8,72E-59
ITPRID2	7,71E-63	1,029682948	0,541	0,089	1,46E-58
PGRMC2	1,91E-62	1,019314468	0,593	0,106	3,61E-58
PDLIM4	2,11E-62	0,60610749	0,326	0,03	4,01E-58
FN1	1,57E-61	1,545360394	0,874	0,275	2,97E-57
HDGFL3	2,14E-61	0,824670396	0,437	0,057	4,06E-57
TINAGL1	9,18E-61	0,911822816	0,57	0,098	1,74E-56
PLA2G4A	1,53E-60	0,523672452	0,252	0,017	2,90E-56
IGFBP3	1,80E-60	1,859985851	0,385	0,047	3,41E-56
SCARA3	1,87E-60	0,951983679	0,4	0,049	3,55E-56
MGP	3,39E-59	1,957957075	0,948	0,552	6,42E-55
PTPN14	2,64E-58	1,094378606	0,622	0,125	5,01E-54
TNS2	6,21E-58	0,702552675	0,326	0,033	1,18E-53
IFITM3	7,42E-58	1,579738407	0,941	0,495	1,41E-53
BMPR2	9,44E-58	1,247860646	0,77	0,215	1,79E-53
HTRA1	1,35E-57	0,992763397	0,6	0,113	2,55E-53
SPTBN1	1,57E-57	1,169741617	0,793	0,221	2,98E-53
CPNE2	1,74E-57	0,632186458	0,385	0,047	3,30E-53
LRP11	2,38E-57	0,530005339	0,259	0,02	4,51E-53
TSPAN3	7,37E-57	0,944492543	0,607	0,121	1,40E-52

JAM2	8,09E-57	0,802990333	0,444	0,063	1,53E-52
CDA	4,79E-56	0,665530368	0,311	0,031	9,08E-52
PEAR1	8,64E-56	0,541588183	0,274	0,024	1,64E-51
SLCO2A1	1,21E-54	1,044286702	0,304	0,03	2,29E-50
IRF6	1,71E-54	0,667980233	0,296	0,029	3,24E-50
CTNNAL1	2,69E-54	0,586188679	0,274	0,024	5,09E-50
ETS2	7,61E-54	0,909426385	0,548	0,103	1,44E-49
SERPING1	2,29E-53	0,957484716	0,6	0,121	4,34E-49
PDE3A	5,33E-53	0,720606086	0,37	0,047	1,01E-48
ARHGAP29	7,40E-53	0,718343852	0,415	0,059	1,40E-48
CTTN	9,52E-53	0,5429247	0,407	0,056	1,80E-48
KLF4	1,25E-52	1,128579945	0,519	0,098	2,37E-48
OLFM1	1,94E-52	0,512850474	0,289	0,028	3,68E-48
MPDZ	1,98E-52	0,650919247	0,333	0,038	3,76E-48
PGRMC1	5,28E-52	0,772827666	0,504	0,089	1,00E-47
IL6ST	1,42E-51	1,249768455	0,763	0,222	2,69E-47
VCAM1	1,52E-51	1,324991702	0,356	0,046	2,88E-47
SCARF1	2,26E-51	0,493856388	0,341	0,04	4,27E-47
NT5E	8,02E-51	0,645910126	0,333	0,039	1,52E-46
TAL1	8,17E-51	0,402892434	0,267	0,024	1,55E-46
GFOD1	8,62E-51	0,66434551	0,393	0,054	1,63E-46
NOTCH4	1,25E-50	0,733960867	0,289	0,03	2,38E-46
NPR2	1,42E-50	0,731157771	0,326	0,038	2,68E-46
HSPB1	1,72E-50	1,310113578	0,881	0,41	3,26E-46
AKR1C3	2,19E-50	0,633853188	0,333	0,039	4,15E-46
DEPP1	2,42E-50	0,703724255	0,259	0,024	4,59E-46
SH3BP4	4,49E-50	0,852867745	0,356	0,046	8,51E-46
CTGF	5,14E-50	1,900663043	0,496	0,096	9,74E-46
FMOD	1,21E-49	0,655733091	0,326	0,038	2,30E-45
DOCK6	1,40E-49	0,487274185	0,274	0,027	2,66E-45
SELENOM	6,83E-49	0,758861959	0,548	0,105	1,29E-44
PTK2	7,80E-49	0,58132325	0,385	0,055	1,48E-44
CD9	1,36E-48	1,100560876	0,711	0,195	2,59E-44
MTMR11	2,01E-48	0,815666919	0,274	0,028	3,81E-44
ZCCHC24	2,63E-48	0,73145079	0,407	0,062	4,99E-44
THBS1	3,24E-48	1,50995916	0,489	0,092	6,14E-44
TJP1	3,48E-48	0,584219758	0,333	0,042	6,59E-44
PLVAP	5,24E-48	0,800697764	0,37	0,051	9,92E-44
ARHGEF10	8,06E-48	0,559432068	0,289	0,031	1,53E-43
PAPSS2	8,52E-48	0,855586799	0,422	0,069	1,61E-43
PTMS	9,58E-48	1,000975767	0,756	0,221	1,81E-43
ITGA3	1,03E-47	0,595278197	0,341	0,044	1,96E-43
RBFOX2	1,76E-47	0,635378194	0,385	0,057	3,34E-43
MGST2	4,28E-47	0,608166471	0,407	0,063	8,12E-43
GRASP	6,31E-47	0,587093305	0,385	0,055	1,20E-42
DOCK9	1,59E-46	0,690433078	0,489	0,089	3,01E-42
DUSP5	2,60E-46	0,938557277	0,304	0,037	4,93E-42
EVA1C	2,99E-46	0,451012887	0,296	0,034	5,67E-42
FRMD4B	3,10E-46	0,722516666	0,519	0,103	5,88E-42
LAMA5	3,25E-46	0,687774493	0,341	0,046	6,17E-42
MYOF	8,35E-46	0,7301971	0,459	0,084	1,58E-41
TIMP1	1,13E-45	1,223146936	0,911	0,505	2,13E-41
GSN	1,66E-45	1,040316923	0,837	0,3	3,15E-41
EHD2	2,19E-45	0,735098689	0,467	0,085	4,15E-41
DAP	2,81E-45	0,633621329	0,563	0,119	5,32E-41
SOX7	5,47E-45	0,835450602	0,444	0,079	1,04E-40

YBX3	6,37E-45	0,890363364	0,674	0,177	1,21E-40
APBB2	7,78E-45	0,514477482	0,341	0,046	1,47E-40
SASH1	9,64E-45	0,752515775	0,511	0,102	1,83E-40
SH3D19	1,15E-44	0,486862776	0,281	0,032	2,17E-40
ZFYVE21	1,27E-44	0,60154088	0,393	0,062	2,41E-40
KLF2	2,60E-44	1,240915572	0,593	0,153	4,93E-40
PDGFD	4,56E-44	0,889195283	0,348	0,05	8,64E-40
PINK1	6,03E-44	0,728465355	0,415	0,071	1,14E-39
TIMP2	1,04E-43	0,947885494	0,726	0,213	1,97E-39
NFE2L1	1,13E-43	0,714919342	0,563	0,126	2,14E-39
PTTG1IP	1,58E-43	1,073575022	0,719	0,236	3,00E-39
SOX13	1,95E-43	0,550830127	0,281	0,032	3,70E-39
FAXDC2	2,03E-43	0,553408207	0,259	0,028	3,84E-39
TMTC1	3,04E-43	0,742979646	0,326	0,045	5,76E-39
ASAP2	3,54E-43	0,538653271	0,356	0,053	6,70E-39
PLS3	6,11E-43	0,73106112	0,459	0,083	1,16E-38
AFDN	6,91E-43	0,699141329	0,452	0,084	1,31E-38
CDH23	1,24E-42	1,001024446	0,43	0,079	2,35E-38
TLNRD1	1,36E-42	0,68366889	0,43	0,078	2,57E-38
PTGS2	1,91E-42	0,820572256	0,422	0,075	3,62E-38
DUSP1	1,95E-42	0,974646603	0,711	0,211	3,69E-38
SORBS2	2,00E-42	0,763977568	0,296	0,038	3,79E-38
VIM	6,98E-42	1,064455689	0,97	0,824	1,32E-37
KCTD12	9,69E-42	0,995714705	0,667	0,186	1,84E-37
ITPR2	1,47E-41	0,916575586	0,652	0,176	2,78E-37
MEIS2	2,15E-41	0,548525505	0,267	0,031	4,07E-37
CDC42BPB	2,41E-41	0,575804543	0,378	0,062	4,57E-37
LTBP4	3,02E-41	0,907747673	0,6	0,149	5,73E-37
CSGALNACT1	3,31E-41	0,743126072	0,363	0,057	6,28E-37
IGFBP7	4,89E-41	1,212240239	0,881	0,377	9,27E-37
NFIX	5,95E-41	0,603865001	0,437	0,08	1,13E-36
ITGA5	6,37E-41	0,905083141	0,659	0,182	1,21E-36
PGM5	8,71E-41	0,608773213	0,259	0,03	1,65E-36
OGN	9,26E-41	1,289612285	0,541	0,133	1,75E-36
CKAP4	1,69E-40	0,698194522	0,43	0,081	3,21E-36
RAB13	2,05E-40	0,646158927	0,467	0,094	3,88E-36
DHRS3	2,85E-40	0,741092864	0,504	0,108	5,40E-36
NFIC	3,80E-40	0,840722726	0,815	0,284	7,19E-36
CTNNB1	5,40E-40	0,913954107	0,681	0,206	1,02E-35
SERPINE2	5,60E-40	0,686198128	0,289	0,037	1,06E-35
HHEX	8,41E-40	0,699538642	0,267	0,033	1,59E-35
DCHS1	8,43E-40	0,587607471	0,281	0,036	1,60E-35
PIK3C2B	1,39E-39	0,614109214	0,363	0,059	2,63E-35
BCAR1	1,44E-39	0,450903669	0,252	0,029	2,72E-35
WARS	1,68E-39	0,676508747	0,519	0,115	3,19E-35
CCND1	2,87E-39	0,768050996	0,422	0,082	5,44E-35
PDLIM5	2,96E-39	1,005800267	0,733	0,248	5,62E-35
TNKS1BP1	3,54E-39	0,690143167	0,333	0,052	6,71E-35
ALDH1A2	4,54E-39	0,849268478	0,267	0,033	8,61E-35
DAB2IP	7,29E-39	0,501925348	0,252	0,03	1,38E-34
LSR	9,86E-39	0,721300236	0,326	0,049	1,87E-34
ACTN1	1,50E-38	0,943575045	0,785	0,269	2,85E-34
PXDC1	5,11E-38	0,68319524	0,326	0,051	9,69E-34
INPP1	8,28E-38	0,462957549	0,289	0,04	1,57E-33
SLC41A3	1,31E-37	0,486314103	0,378	0,066	2,48E-33
SELENON	1,67E-37	0,555930851	0,43	0,084	3,17E-33

ALDH2	1,69E-37	0,62311952	0,489	0,107	3,21E-33
B3GALNT1	1,87E-37	0,481317966	0,281	0,038	3,54E-33
CSF2RB	3,11E-37	0,7818753	0,415	0,081	5,90E-33
PON2	3,30E-37	0,512413098	0,356	0,06	6,25E-33
sep-10	3,41E-37	0,509393024	0,304	0,045	6,46E-33
GNG12	4,96E-37	0,755668257	0,4	0,076	9,40E-33
DOCK1	5,56E-37	0,437099682	0,252	0,031	1,05E-32
MATN2	5,69E-37	0,709892438	0,304	0,046	1,08E-32
CYB5A	7,44E-37	0,462796215	0,304	0,045	1,41E-32
JUN	1,06E-36	0,90242731	0,585	0,161	2,01E-32
SETBP1	1,67E-36	0,606335153	0,37	0,065	3,16E-32
ABLIM1	3,10E-36	0,743858287	0,652	0,185	5,87E-32
FNIP2	3,58E-36	0,595640703	0,4	0,078	6,78E-32
TMEM120A	4,08E-36	0,673746127	0,356	0,063	7,72E-32
CCDC50	4,73E-36	0,704021189	0,511	0,122	8,96E-32
MSRB3	5,28E-36	0,574193794	0,541	0,128	1,00E-31
ATN1	6,68E-36	0,825816081	0,563	0,15	1,27E-31
MDK	1,83E-35	0,664239989	0,304	0,047	3,46E-31
GABARAPL2	3,63E-35	0,704976496	0,63	0,179	6,88E-31
MRFAP1	4,06E-35	0,725440056	0,63	0,184	7,70E-31
PARVA	6,15E-35	0,753465109	0,526	0,13	1,17E-30
TCF4	6,17E-35	0,797362972	0,719	0,23	1,17E-30
ADAM15	9,21E-35	0,531213446	0,341	0,059	1,75E-30
BTG2	9,63E-35	1,010670002	0,474	0,112	1,82E-30
TEAD1	1,07E-34	0,460648852	0,304	0,047	2,04E-30
SPARCL1	1,38E-34	0,695376607	0,541	0,138	2,62E-30
NQO1	1,47E-34	0,580386378	0,274	0,039	2,78E-30
ALDH1A1	1,48E-34	0,654864449	0,319	0,053	2,81E-30
HERC2P2	1,93E-34	0,51016574	0,281	0,041	3,65E-30
RAPGEF5	1,96E-34	0,44617035	0,252	0,033	3,71E-30
SLC18A1	2,89E-34	0,676975958	0,563	0,146	5,47E-30
TIMP3	4,11E-34	1,256396209	0,393	0,083	7,80E-30
EPHX1	5,02E-34	0,5515238	0,341	0,061	9,52E-30
ITGAV	5,38E-34	0,640374745	0,415	0,088	1,02E-29
SPARC	7,85E-34	0,784362824	0,667	0,21	1,49E-29
FAM171A1	8,65E-34	0,513385265	0,252	0,034	1,64E-29
TRIM47	9,12E-34	0,487017908	0,289	0,044	1,73E-29
YAP1	9,69E-34	0,358467773	0,304	0,048	1,84E-29
LMNA	1,05E-33	0,729910989	0,793	0,28	1,99E-29
MORF4L2	1,71E-33	0,687854577	0,57	0,153	3,24E-29
CTTNBP2NL	1,85E-33	0,513837341	0,296	0,047	3,51E-29
PLAT	2,07E-33	0,469068578	0,363	0,068	3,92E-29
RCAN1	2,29E-33	0,560089276	0,363	0,07	4,34E-29
IFI44L	2,53E-33	0,599466155	0,422	0,09	4,80E-29
TSC22D1	2,57E-33	0,848259342	0,415	0,088	4,87E-29
TSPAN2	2,97E-33	0,532454693	0,267	0,038	5,64E-29
MRPL17	3,06E-33	0,425710723	0,304	0,049	5,79E-29
GALNT2	4,13E-33	0,595875937	0,459	0,105	7,83E-29
SKIL	5,40E-33	0,722408806	0,6	0,17	1,02E-28
BCAP29	7,92E-33	0,637183428	0,489	0,119	1,50E-28
GMDS	9,10E-33	0,467640728	0,259	0,037	1,73E-28
FZD4	1,10E-32	0,613175537	0,341	0,063	2,09E-28
DSTN	1,55E-32	0,681442292	0,681	0,224	2,94E-28
EMP2	3,55E-32	0,899380852	0,696	0,245	6,73E-28
TJP2	3,78E-32	0,567076368	0,304	0,052	7,16E-28
LEPROT	5,91E-32	0,769255707	0,622	0,191	1,12E-27

TCEAL9	6,92E-32	0,481931905	0,356	0,069	1,31E-27
IGFBP2	9,00E-32	0,451280506	0,356	0,067	1,71E-27
ROBO3	9,85E-32	0,578315577	0,274	0,043	1,87E-27
PAM	1,42E-31	0,717926507	0,459	0,111	2,69E-27
SOCS2	2,10E-31	0,383195179	0,259	0,038	3,97E-27
CALU	2,14E-31	0,797594561	0,607	0,188	4,06E-27
CNN3	2,25E-31	0,434516551	0,341	0,063	4,26E-27
C9orf3	2,27E-31	0,424858863	0,296	0,05	4,31E-27
NGRN	7,75E-31	0,505900602	0,378	0,079	1,47E-26
TSPAN18	8,15E-31	0,490671235	0,311	0,054	1,55E-26
CAMSAP2	8,61E-31	0,551637536	0,326	0,061	1,63E-26
DENND5A	8,76E-31	0,422092912	0,385	0,08	1,66E-26
INSIG2	1,11E-30	0,413403182	0,311	0,055	2,11E-26
NUCB2	1,23E-30	0,653303082	0,504	0,13	2,33E-26
AFAP1	1,47E-30	0,521000087	0,289	0,049	2,78E-26
ACVR1	1,87E-30	0,511553891	0,296	0,051	3,54E-26
TTC28	1,90E-30	0,387781544	0,289	0,048	3,60E-26
KCNN3	2,37E-30	0,600634859	0,356	0,071	4,49E-26
FGF2	2,47E-30	0,517675614	0,319	0,058	4,68E-26
SPTAN1	2,64E-30	0,878596585	0,719	0,272	5,01E-26
MRAS	4,53E-30	0,588614635	0,252	0,039	8,58E-26
RAI14	6,05E-30	0,476674082	0,319	0,058	1,15E-25
DST	7,70E-30	0,623739734	0,452	0,111	1,46E-25
SERPINH1	8,61E-30	0,512693835	0,333	0,065	1,63E-25
RBPMS	9,72E-30	0,539726352	0,341	0,067	1,84E-25
RAB11A	1,18E-29	0,634755653	0,63	0,201	2,24E-25
PLSCR4	2,21E-29	0,437955044	0,326	0,062	4,18E-25
LRRC8A	2,31E-29	0,639541277	0,363	0,078	4,39E-25
BEX3	2,47E-29	0,493520804	0,415	0,096	4,69E-25
SLC39A14	2,74E-29	0,407581341	0,259	0,041	5,20E-25
FAM43A	2,81E-29	0,525355628	0,259	0,041	5,33E-25
MYO1C	3,29E-29	0,750264118	0,541	0,159	6,24E-25
MAGI2-AS3	4,52E-29	0,410320198	0,281	0,048	8,57E-25
TMBIM1	6,00E-29	0,66370894	0,474	0,127	1,14E-24
LAPTM4A	6,16E-29	0,726887644	0,681	0,249	1,17E-24
SELENOW	6,17E-29	0,682039072	0,585	0,184	1,17E-24
S100A6	1,14E-28	0,742323888	0,97	0,669	2,17E-24
DPY19L1	1,51E-28	0,582374153	0,393	0,091	2,85E-24
GIMAP6	1,53E-28	0,457027153	0,393	0,087	2,90E-24
RRAS	2,23E-28	0,489329019	0,385	0,086	4,22E-24
GNA11	2,48E-28	0,367190807	0,267	0,044	4,70E-24
SLC29A1	2,87E-28	0,420166726	0,378	0,083	5,43E-24
CLIC2	3,12E-28	0,40169684	0,319	0,062	5,92E-24
SERPINE1	3,54E-28	1,417695532	0,474	0,134	6,71E-24
PDGFB	3,76E-28	0,515151844	0,326	0,065	7,12E-24
CTSF	4,10E-28	0,426202901	0,304	0,057	7,77E-24
NECTIN2	6,06E-28	0,517031963	0,311	0,061	1,15E-23
WSB1	6,48E-28	1,054891982	0,793	0,377	1,23E-23
ATP1A1	6,84E-28	0,582000284	0,681	0,23	1,30E-23
CCDC80	9,40E-28	0,363315691	0,4	0,093	1,78E-23
TNFRSF1A	1,06E-27	0,437093673	0,444	0,111	2,01E-23
CYB5R3	1,07E-27	0,741244305	0,704	0,264	2,03E-23
IFI6	1,25E-27	0,978240644	0,644	0,24	2,37E-23
KDELR2	1,29E-27	0,632655015	0,533	0,16	2,45E-23
RIPOR1	1,31E-27	0,635302864	0,474	0,129	2,47E-23
CTNNA1	1,57E-27	0,612349805	0,511	0,148	2,97E-23

GOLIM4	1,88E-27	0,43862618	0,252	0,042	3,57E-23
LATS2	2,38E-27	0,492942993	0,296	0,057	4,51E-23
TUBB6	2,68E-27	0,567751621	0,341	0,073	5,09E-23
MLLT1	3,48E-27	0,5375731	0,422	0,105	6,60E-23
CDK2AP1	4,00E-27	0,578280459	0,474	0,131	7,57E-23
GPX3	4,08E-27	0,579359776	0,407	0,099	7,74E-23
PPP2CB	5,08E-27	0,543330179	0,444	0,116	9,63E-23
PRXL2A	6,23E-27	0,541820537	0,415	0,102	1,18E-22
FAM114A1	6,95E-27	0,443833715	0,363	0,081	1,32E-22
FEZ2	7,59E-27	0,566683083	0,407	0,101	1,44E-22
MTRF1L	1,02E-26	0,56020213	0,4	0,097	1,93E-22
SHE	2,08E-26	0,503934394	0,407	0,099	3,94E-22
SLC6A2	2,11E-26	0,612388766	0,341	0,075	4,01E-22
RGL2	2,81E-26	0,442291408	0,407	0,099	5,32E-22
MARVELD1	3,75E-26	0,410525373	0,274	0,051	7,12E-22
COPRS	6,75E-26	0,423067062	0,311	0,064	1,28E-21
APOL1	9,32E-26	0,492903456	0,363	0,084	1,77E-21
LRIG1	9,64E-26	0,461910153	0,326	0,069	1,83E-21
SBDS	1,21E-25	0,427944439	0,393	0,095	2,28E-21
VCL	1,24E-25	0,548573332	0,504	0,147	2,35E-21
PDLIM7	1,39E-25	0,476471124	0,37	0,087	2,64E-21
SYNJ2	1,72E-25	0,507131171	0,259	0,047	3,27E-21
ADGRL1	2,02E-25	0,326155372	0,252	0,043	3,82E-21
ZBTB16	2,22E-25	0,446188153	0,304	0,062	4,21E-21
PHLDB1	3,06E-25	0,347069769	0,252	0,044	5,80E-21
PRSS23	4,67E-25	0,794972792	0,37	0,091	8,85E-21
TGFBR2	4,71E-25	0,751204308	0,622	0,228	8,93E-21
RBMS2	4,94E-25	0,620437527	0,363	0,089	9,36E-21
KIF13A	5,68E-25	0,463528471	0,356	0,083	1,08E-20
COL3A1	6,05E-25	0,846300725	0,474	0,137	1,15E-20
GIMAP7	6,42E-25	0,5940722	0,474	0,138	1,22E-20
SOCS3	1,26E-24	0,927373307	0,556	0,191	2,38E-20
TPM2	1,28E-24	0,471215894	0,4	0,102	2,42E-20
FNDC3B	1,50E-24	0,757010679	0,474	0,146	2,84E-20
TNS1	1,67E-24	0,458083041	0,452	0,123	3,16E-20
IGF1R	1,87E-24	0,471652368	0,281	0,056	3,54E-20
INMT	1,89E-24	0,784658421	0,259	0,05	3,57E-20
FKBP1A	2,76E-24	0,77996272	0,8	0,45	5,22E-20
EGR1	2,88E-24	1,009524806	0,637	0,252	5,46E-20
ANO6	3,21E-24	0,470821469	0,437	0,121	6,08E-20
CMIP	3,79E-24	0,727733376	0,756	0,343	7,18E-20
METTL7A	4,83E-24	0,574994645	0,637	0,225	9,14E-20
CCDC85B	6,00E-24	0,442532532	0,452	0,123	1,14E-19
DAG1	6,01E-24	0,386558061	0,281	0,057	1,14E-19
TMEM184B	7,03E-24	0,475420345	0,348	0,084	1,33E-19
TMEM204	1,01E-23	0,473838477	0,319	0,07	1,91E-19
MOB2	1,01E-23	0,39847595	0,289	0,06	1,92E-19
NDUFC1	1,47E-23	0,467904461	0,519	0,16	2,79E-19
RDX	1,70E-23	0,651565243	0,489	0,151	3,22E-19
COL18A1	1,99E-23	0,252483429	0,363	0,086	3,78E-19
EI24	2,17E-23	0,694310983	0,504	0,159	4,10E-19
NUCB1	2,30E-23	0,584246343	0,689	0,262	4,36E-19
AGPAT1	2,35E-23	0,366014528	0,274	0,055	4,46E-19
SNRK	2,64E-23	0,484321127	0,459	0,13	4,99E-19
ITM2B	2,87E-23	0,661903912	0,963	0,793	5,43E-19
PLXND1	4,88E-23	0,528627695	0,415	0,115	9,25E-19

LAPTM4B	5,46E-23	0,381020275	0,267	0,053	1,03E-18
CDKN1C	5,53E-23	0,613877383	0,296	0,066	1,05E-18
RTL8C	5,91E-23	0,548532489	0,474	0,145	1,12E-18
H1F0	6,12E-23	0,386173705	0,274	0,056	1,16E-18
DPYSL2	6,41E-23	0,6216453	0,593	0,207	1,22E-18
BLCAP	6,93E-23	0,445674189	0,37	0,095	1,31E-18
VAMP3	6,96E-23	0,535727282	0,459	0,138	1,32E-18
MAP3K11	9,38E-23	0,349929159	0,311	0,069	1,78E-18
STOM	9,57E-23	0,80446638	0,756	0,37	1,81E-18
SHC1	1,17E-22	0,529302551	0,489	0,153	2,22E-18
RAC1	1,18E-22	0,606381395	0,911	0,49	2,24E-18
POR	1,55E-22	0,410266735	0,311	0,071	2,94E-18
EVI5	1,61E-22	0,514130789	0,37	0,097	3,05E-18
DKK3	1,63E-22	0,500091686	0,378	0,097	3,08E-18
COL5A2	1,69E-22	0,408121829	0,259	0,051	3,20E-18
BST2	2,03E-22	0,661635278	0,519	0,171	3,85E-18
NR4A1	2,22E-22	0,755359173	0,4	0,113	4,21E-18
CD81	2,25E-22	0,741203097	0,852	0,515	4,27E-18
ARPC1A	2,49E-22	0,470676633	0,422	0,12	4,72E-18
CXCL2	2,95E-22	1,001558261	0,311	0,075	5,60E-18
PVR	4,03E-22	0,552494555	0,341	0,085	7,64E-18
FRY	4,18E-22	0,329804989	0,274	0,058	7,93E-18
NR2F2	4,20E-22	0,994745787	0,393	0,113	7,97E-18
RHOJ	4,39E-22	0,460003771	0,37	0,095	8,31E-18
MBNL2	4,81E-22	0,459528305	0,459	0,136	9,11E-18
ADGRF5	5,52E-22	0,590484789	0,289	0,065	1,05E-17
PITPNA	5,67E-22	0,490526293	0,43	0,124	1,08E-17
ZEB1	6,05E-22	0,524486428	0,393	0,107	1,15E-17
PRDX1	6,89E-22	0,576502888	0,659	0,262	1,31E-17
MEF2A	7,40E-22	0,425777661	0,496	0,155	1,40E-17
PAPSS1	8,93E-22	0,502990996	0,407	0,116	1,69E-17
TRIP6	1,29E-21	0,536741828	0,274	0,061	2,44E-17
ARHGEF12	1,31E-21	0,364479185	0,422	0,118	2,48E-17
ICAM1	1,41E-21	0,874750188	0,311	0,076	2,67E-17
ARL2BP	1,50E-21	0,467562415	0,393	0,107	2,84E-17
LPCAT2	1,93E-21	0,433121256	0,363	0,096	3,65E-17
STK38L	2,07E-21	0,63460268	0,319	0,079	3,92E-17
RHEBP1	2,64E-21	0,508746692	0,452	0,139	5,00E-17
TNFSF10	3,06E-21	0,554878569	0,504	0,166	5,80E-17
RAB1A	3,33E-21	0,393106793	0,541	0,181	6,30E-17
SCARB2	3,85E-21	0,494783079	0,459	0,143	7,29E-17
MEGF6	4,18E-21	0,447670162	0,311	0,074	7,92E-17
MRTFB	4,76E-21	0,666556494	0,274	0,061	9,02E-17
SETD7	5,09E-21	0,37757816	0,326	0,081	9,65E-17
BNIP3L	6,14E-21	0,500554858	0,578	0,203	1,16E-16
REXO2	6,15E-21	0,38236397	0,37	0,099	1,17E-16
FSCN1	8,70E-21	0,340380471	0,319	0,077	1,65E-16
HLA-E	1,34E-20	0,610480495	0,948	0,849	2,53E-16
NDRG1	1,54E-20	0,54786368	0,526	0,184	2,92E-16
NPTN	2,22E-20	0,573196342	0,481	0,16	4,20E-16
FBLN5	2,22E-20	0,653256062	0,4	0,117	4,20E-16
NUP160	2,27E-20	0,328747452	0,304	0,072	4,30E-16
RNF11	2,54E-20	0,467009847	0,452	0,141	4,81E-16
DAB2	4,34E-20	0,372614089	0,437	0,132	8,22E-16
SLC38A2	4,93E-20	0,672762993	0,519	0,189	9,34E-16
SCOC	5,21E-20	0,329740005	0,326	0,082	9,87E-16

SLC48A1	5,46E-20	0,547781574	0,296	0,074	1,03E-15
HSP90B1	7,04E-20	0,699292074	0,785	0,41	1,33E-15
IRAK3	8,02E-20	0,401159768	0,296	0,072	1,52E-15
YWHAE	8,89E-20	0,631230159	0,63	0,26	1,68E-15
TSPAN4	8,94E-20	0,352646233	0,259	0,057	1,69E-15
TMED7	9,54E-20	0,36383102	0,4	0,116	1,81E-15
PEA15	1,05E-19	0,406223112	0,652	0,246	1,98E-15
ARL2	1,72E-19	0,31165224	0,333	0,087	3,26E-15
BCR	1,84E-19	0,279624821	0,267	0,059	3,49E-15
CAPZA2	2,33E-19	0,535658867	0,578	0,223	4,41E-15
PHACTR2	2,57E-19	0,478373805	0,6	0,231	4,88E-15
STMN3	2,64E-19	0,516383946	0,467	0,155	5,01E-15
NAV1	2,95E-19	0,731473418	0,37	0,114	5,58E-15
LHFPL6	3,18E-19	0,472335078	0,326	0,085	6,03E-15
BHLHE40	3,38E-19	0,435830229	0,393	0,116	6,41E-15
MFGE8	3,52E-19	0,292146322	0,304	0,075	6,67E-15
DPYSL3	4,15E-19	0,320790455	0,289	0,069	7,87E-15
NOTCH1	4,19E-19	0,382353673	0,311	0,079	7,93E-15
APLP2	4,22E-19	0,521949288	0,807	0,386	7,99E-15
FLT1	4,23E-19	0,77444649	0,437	0,144	8,01E-15
IER3IP1	4,63E-19	0,354054282	0,393	0,115	8,77E-15
ST6GAL1	6,46E-19	0,401856859	0,459	0,148	1,22E-14
TRIP10	7,46E-19	0,456347271	0,274	0,066	1,41E-14
CYSTM1	8,99E-19	0,481122258	0,259	0,061	1,70E-14
CREB3L2	1,04E-18	0,460186666	0,467	0,158	1,98E-14
UNC5B	1,06E-18	0,303148529	0,252	0,056	2,01E-14
TRIM8	1,22E-18	0,542433516	0,356	0,106	2,32E-14
SEC62	1,26E-18	0,491807593	0,822	0,392	2,38E-14
ZNF358	1,95E-18	0,330619853	0,252	0,057	3,70E-14
TENT5A	2,08E-18	0,596117353	0,341	0,099	3,94E-14
MAGED2	2,22E-18	0,322797865	0,437	0,138	4,20E-14
ARRB1	2,49E-18	0,291619732	0,326	0,087	4,72E-14
ATP2A2	2,66E-18	0,410417707	0,504	0,178	5,04E-14
SUN1	2,73E-18	0,319713035	0,326	0,087	5,16E-14
FOS	2,93E-18	0,819178989	0,711	0,372	5,55E-14
KLF10	3,15E-18	0,398230585	0,267	0,064	5,97E-14
KLF9	3,74E-18	0,444153168	0,407	0,13	7,09E-14
FHL1	4,35E-18	0,306003771	0,378	0,111	8,24E-14
PKD2	4,49E-18	0,562224835	0,43	0,143	8,51E-14
DLGAP4	4,86E-18	0,346021876	0,356	0,103	9,21E-14
NORAD	5,01E-18	0,527275448	0,741	0,369	9,49E-14
TFG	5,49E-18	0,341234721	0,393	0,121	1,04E-13
PRXL2B	6,25E-18	0,481435633	0,274	0,068	1,18E-13
CIC	6,29E-18	0,326862665	0,326	0,089	1,19E-13
ADD1	6,43E-18	0,504746661	0,578	0,232	1,22E-13
RHOC	7,89E-18	0,563277136	0,422	0,144	1,50E-13
SLC44A2	8,04E-18	0,657752267	0,578	0,245	1,52E-13
LMBRD1	8,15E-18	0,402494689	0,385	0,117	1,54E-13
KIF13B	8,45E-18	0,490870098	0,311	0,083	1,60E-13
OS9	9,18E-18	0,542956339	0,63	0,267	1,74E-13
LAMA4	1,15E-17	0,353012341	0,333	0,093	2,18E-13
SOX4	1,29E-17	0,536481249	0,378	0,119	2,45E-13
TCEAL8	1,49E-17	0,333062062	0,274	0,068	2,83E-13
FKBP2	1,67E-17	0,430112599	0,444	0,148	3,17E-13
TCF7L2	1,71E-17	0,309871052	0,326	0,09	3,24E-13
ACBD3	1,80E-17	0,394269088	0,304	0,081	3,42E-13

CHMP5	2,14E-17	0,435709437	0,393	0,125	4,06E-13
TPM4	2,16E-17	0,554625089	0,778	0,416	4,09E-13
SELENOS	2,16E-17	0,30262513	0,333	0,094	4,09E-13
PAFAH1B2	2,20E-17	0,38506119	0,533	0,199	4,17E-13
CSRP1	2,34E-17	0,320436047	0,526	0,193	4,43E-13
GABARAPL1	4,29E-17	0,353091045	0,319	0,089	8,12E-13
NFE2L2	4,43E-17	0,362834019	0,489	0,174	8,40E-13
CITED2	5,23E-17	0,28179585	0,252	0,06	9,92E-13
SYPL1	5,95E-17	0,416507172	0,385	0,122	1,13E-12
FGFR1	5,96E-17	0,470949102	0,356	0,108	1,13E-12
PKD1P1	7,01E-17	0,450125054	0,6	0,244	1,33E-12
TCEAL4	7,09E-17	0,271286239	0,333	0,096	1,34E-12
CNPY2	1,04E-16	0,281560945	0,348	0,103	1,97E-12
CTSK	1,04E-16	0,445350669	0,304	0,085	1,98E-12
CRBN	1,19E-16	0,271321576	0,37	0,113	2,26E-12
CDIPT	1,34E-16	0,508663726	0,43	0,148	2,54E-12
CSF1	1,54E-16	0,400213619	0,252	0,062	2,92E-12
YIPF6	1,55E-16	0,391445653	0,333	0,099	2,93E-12
FAM3C	1,61E-16	0,283950518	0,333	0,097	3,06E-12
CCDC47	1,64E-16	0,383692748	0,37	0,117	3,11E-12
CSNK1E	1,71E-16	0,367924816	0,407	0,136	3,24E-12
MAGED1	1,76E-16	0,338078225	0,333	0,098	3,33E-12
SERINC3	1,79E-16	0,431889473	0,511	0,198	3,39E-12
DUSP3	1,84E-16	0,330988102	0,348	0,105	3,49E-12
SORBS3	1,86E-16	0,338927115	0,356	0,108	3,52E-12
PTPRJ	2,12E-16	0,637104728	0,489	0,191	4,02E-12
SLC43A3	2,27E-16	0,42021013	0,333	0,1	4,31E-12
NPEPPS	2,61E-16	0,426255695	0,422	0,144	4,96E-12
ZMPSTE24	2,71E-16	0,283486823	0,341	0,102	5,14E-12
SYNGR2	2,95E-16	0,416953572	0,511	0,198	5,59E-12
AHR	2,95E-16	0,377014799	0,481	0,179	5,59E-12
CRK	4,12E-16	0,414194189	0,43	0,15	7,80E-12
MAFG	4,84E-16	0,325758194	0,333	0,1	9,17E-12
FBLIM1	4,86E-16	0,535869573	0,652	0,3	9,20E-12
ZMIZ1	4,93E-16	0,386871491	0,363	0,117	9,35E-12
A2M	5,47E-16	0,917579768	0,407	0,154	1,04E-11
ZNHIT1	5,82E-16	0,308832628	0,474	0,169	1,10E-11
CD63	6,24E-16	0,409503031	0,785	0,401	1,18E-11
TMEM173	7,30E-16	0,425099534	0,607	0,249	1,38E-11
LAMP1	1,04E-15	0,403019603	0,681	0,303	1,98E-11
TMEM59	1,18E-15	0,519469573	0,711	0,34	2,24E-11
HDAC9	1,27E-15	0,460966986	0,467	0,173	2,40E-11
USP22	1,35E-15	0,359340092	0,563	0,229	2,55E-11
PDIA3	1,54E-15	0,452981054	0,511	0,2	2,92E-11
CAMTA1	1,63E-15	0,39885614	0,4	0,138	3,09E-11
JUNB	1,89E-15	0,811720481	0,748	0,438	3,57E-11
ZFP36	2,17E-15	0,868262078	0,563	0,261	4,12E-11
TRIOBP	2,32E-15	0,45615643	0,548	0,225	4,40E-11
VASH1	2,39E-15	0,326176207	0,319	0,095	4,53E-11
SNX9	2,69E-15	0,275762043	0,378	0,124	5,09E-11
FAM129B	3,07E-15	0,35424434	0,341	0,107	5,82E-11
RALB	3,18E-15	0,353496058	0,319	0,096	6,02E-11
TRAM1	3,28E-15	0,39257873	0,711	0,329	6,21E-11
USP53	3,49E-15	0,383565975	0,363	0,119	6,62E-11
SLC39A1	3,60E-15	0,341135288	0,296	0,087	6,82E-11
ARMCX3	3,62E-15	0,34437019	0,289	0,083	6,87E-11

PLEC	3,64E-15	0,451973249	0,644	0,294	6,90E-11
VAMP5	3,69E-15	0,266586708	0,304	0,088	7,00E-11
HYOU1	3,73E-15	0,344033557	0,37	0,122	7,07E-11
STX7	4,14E-15	0,273529965	0,348	0,11	7,85E-11
DYNC1I2	4,37E-15	0,440689402	0,519	0,211	8,28E-11
SNX18	4,49E-15	0,330039068	0,311	0,094	8,51E-11
GANAB	5,79E-15	0,3512633	0,459	0,167	1,10E-10
CCPG1	5,88E-15	0,25609981	0,281	0,08	1,11E-10
GNG5	6,48E-15	0,449135331	0,607	0,269	1,23E-10
ARHGAP21	6,69E-15	0,372272259	0,289	0,085	1,27E-10
RELL1	6,78E-15	0,312372537	0,281	0,08	1,29E-10
RRBP1	7,95E-15	0,41063718	0,363	0,121	1,51E-10
ZHX1	7,98E-15	0,274457863	0,252	0,067	1,51E-10
SEC61A1	8,01E-15	0,368289441	0,452	0,166	1,52E-10
ZNF395	8,11E-15	0,317222112	0,422	0,149	1,54E-10
NELL1	8,53E-15	0,357726049	0,341	0,109	1,62E-10
TM9SF2	9,12E-15	0,344416022	0,556	0,229	1,73E-10
CAVIN3	9,69E-15	0,460890835	0,289	0,086	1,84E-10
PPP1R15A	1,03E-14	0,415423063	0,415	0,151	1,95E-10
TPM1	1,12E-14	0,533742015	0,348	0,116	2,13E-10
INPP5K	1,38E-14	0,276869013	0,252	0,068	2,62E-10
FCGRT	1,41E-14	0,254088557	0,667	0,284	2,67E-10
TMED10	1,42E-14	0,414929635	0,615	0,276	2,68E-10
PBX1	1,51E-14	0,411175665	0,296	0,09	2,85E-10
VKORC1	1,67E-14	0,373075931	0,4	0,143	3,16E-10
RAB2A	1,77E-14	0,311676465	0,496	0,194	3,35E-10
SKI	1,81E-14	0,403820414	0,674	0,31	3,43E-10
ARF4	2,14E-14	0,369946456	0,459	0,176	4,06E-10
C1orf43	2,22E-14	0,285768666	0,556	0,223	4,20E-10
PRAF2	2,33E-14	0,427247453	0,326	0,103	4,41E-10
ITGB5	2,54E-14	0,313642134	0,289	0,085	4,81E-10
SLIRP	2,67E-14	0,368360835	0,37	0,128	5,06E-10
SEC23A	2,76E-14	0,310841153	0,296	0,088	5,24E-10
TACC1	3,18E-14	0,510335069	0,719	0,391	6,02E-10
MAPK3	3,19E-14	0,339063718	0,341	0,111	6,04E-10
AZIN1	3,22E-14	0,283180462	0,4	0,14	6,10E-10
ADAM9	3,46E-14	0,337440272	0,267	0,077	6,56E-10
PMEPA1	3,60E-14	0,299205072	0,356	0,117	6,82E-10
ARHGEF2	3,64E-14	0,428205531	0,415	0,154	6,89E-10
KLF3	3,67E-14	0,390857384	0,363	0,125	6,96E-10
MYO1D	3,82E-14	0,297906991	0,274	0,078	7,25E-10
GIMAP1	3,83E-14	0,310919422	0,407	0,144	7,26E-10
YIPF3	3,90E-14	0,38049183	0,393	0,141	7,39E-10
CXCL12	4,81E-14	0,514242547	0,267	0,077	9,12E-10
IRF2BP2	4,89E-14	0,385171626	0,548	0,237	9,27E-10
SRP9	4,97E-14	0,299542843	0,578	0,24	9,42E-10
STAU1	5,17E-14	0,469264575	0,422	0,161	9,80E-10
RNF7	6,40E-14	0,356633582	0,415	0,152	1,21E-09
IER3	6,94E-14	0,280896968	0,444	0,167	1,32E-09
IER2	7,61E-14	0,524063396	0,496	0,206	1,44E-09
ZFAND3	8,20E-14	0,343499286	0,274	0,081	1,55E-09
CPT1A	1,14E-13	0,2912334	0,548	0,226	2,15E-09
SERINC1	1,26E-13	0,448473303	0,696	0,363	2,39E-09
P4HB	1,36E-13	0,370483008	0,674	0,315	2,58E-09
G6PC3	1,39E-13	0,313369093	0,267	0,078	2,64E-09
TMEM30A	1,42E-13	0,368958532	0,541	0,23	2,70E-09

ACTN4	1,46E-13	0,49955047	0,852	0,506	2,77E-09
ATP13A3	1,50E-13	0,500674801	0,422	0,162	2,85E-09
CALR	1,68E-13	0,428710081	0,733	0,383	3,18E-09
NCSTN	1,70E-13	0,285101252	0,385	0,138	3,22E-09
DYNLL1	1,70E-13	0,54959975	0,667	0,341	3,22E-09
FLOT1	2,09E-13	0,455421094	0,556	0,242	3,95E-09
SERPINB6	2,30E-13	0,373371167	0,511	0,217	4,35E-09
MYL12B	2,57E-13	0,47148066	0,8	0,525	4,88E-09
MYO6	2,67E-13	0,326609289	0,385	0,138	5,06E-09
KDELR1	2,70E-13	0,488114553	0,437	0,167	5,11E-09
MAN1A1	3,01E-13	0,278996819	0,393	0,142	5,70E-09
HDLBP	3,04E-13	0,282841522	0,533	0,224	5,77E-09
CPD	3,09E-13	0,284520807	0,311	0,101	5,86E-09
WWC3	3,85E-13	0,343005923	0,422	0,162	7,29E-09
F11R	4,20E-13	0,299878023	0,319	0,104	7,96E-09
MYH9	4,41E-13	0,509515848	0,867	0,648	8,36E-09
HAX1	4,52E-13	0,336110356	0,274	0,085	8,56E-09
ARF1	4,57E-13	0,401455094	0,748	0,4	8,65E-09
APOL2	4,59E-13	0,352082448	0,348	0,119	8,69E-09
FLI1	5,26E-13	0,254766573	0,37	0,131	9,97E-09
NAMPT	5,48E-13	0,463292031	0,467	0,192	1,04E-08
MXRA7	5,85E-13	0,281594957	0,415	0,154	1,11E-08
CASC4	5,93E-13	0,35551422	0,444	0,176	1,12E-08
COMT	6,22E-13	0,348902775	0,363	0,132	1,18E-08
HBEGF	6,58E-13	0,343586263	0,281	0,087	1,25E-08
SDCBP	6,69E-13	0,371513568	0,674	0,322	1,27E-08
ANKS1B	7,29E-13	0,313428247	0,489	0,204	1,38E-08
METRNL	7,73E-13	0,279894225	0,363	0,128	1,46E-08
TMEM109	9,12E-13	0,342213419	0,526	0,225	1,73E-08
ZNF664	1,04E-12	0,280374403	0,341	0,119	1,96E-08
LMAN1	1,27E-12	0,38762435	0,422	0,169	2,40E-08
TUBB2A	1,27E-12	0,311602498	0,778	0,392	2,41E-08
DYNC1LI2	1,32E-12	0,279355438	0,4	0,151	2,50E-08
PIP5K1C	1,58E-12	0,404052204	0,296	0,098	3,00E-08
CANX	1,64E-12	0,492197088	0,756	0,433	3,10E-08
TMEM43	1,67E-12	0,409227104	0,415	0,165	3,16E-08
COPG1	1,70E-12	0,322956308	0,296	0,098	3,23E-08
PTPN12	1,82E-12	0,344727687	0,452	0,187	3,45E-08
CPNE3	2,08E-12	0,294173987	0,444	0,177	3,95E-08
ARID5B	2,26E-12	0,486634013	0,4	0,16	4,29E-08
OAZ2	2,42E-12	0,263643935	0,267	0,083	4,59E-08
YWHAG	2,61E-12	0,301465959	0,415	0,162	4,94E-08
TMED2	2,98E-12	0,367473042	0,526	0,236	5,65E-08
HNRNPAB	3,05E-12	0,405774568	0,474	0,201	5,78E-08
TMED9	3,09E-12	0,291588731	0,393	0,15	5,85E-08
MAP1B	3,15E-12	0,431504314	0,304	0,102	5,96E-08
DNAJC10	3,36E-12	0,285594506	0,393	0,15	6,37E-08
HNRNPA0	3,57E-12	0,348462925	0,489	0,206	6,76E-08
LRRC8C	3,63E-12	0,2604142	0,356	0,128	6,88E-08
UFD1	3,83E-12	0,262897864	0,348	0,126	7,26E-08
PLSCR1	4,32E-12	0,302812965	0,281	0,092	8,18E-08
ITGB1	4,38E-12	0,376395389	0,815	0,487	8,31E-08
PCMTD1	4,41E-12	0,281586306	0,496	0,209	8,36E-08
TMEM14C	4,58E-12	0,258194566	0,378	0,141	8,68E-08
RTL8A	4,98E-12	0,304228441	0,252	0,077	9,43E-08
ARL8A	5,09E-12	0,311390604	0,341	0,122	9,65E-08

HSPA5	5,11E-12	0,41037945	0,637	0,31	9,68E-08
SEC13	5,30E-12	0,252092913	0,333	0,118	1,00E-07
MIDN	5,40E-12	0,452116223	0,541	0,253	1,02E-07
GALNT15	5,56E-12	0,423483258	0,274	0,09	1,05E-07
UACA	5,71E-12	0,378342537	0,348	0,126	1,08E-07
GORASP2	5,79E-12	0,268165594	0,319	0,11	1,10E-07
BZW1	6,77E-12	0,350585769	0,593	0,281	1,28E-07
SLC20A1	7,15E-12	0,286795203	0,393	0,149	1,35E-07
ADI1	7,63E-12	0,260808126	0,326	0,115	1,45E-07
HERPUD1	8,85E-12	0,296898569	0,422	0,167	1,68E-07
PURA	1,00E-11	0,265905771	0,511	0,221	1,90E-07
PPP1R15B	1,05E-11	0,282652207	0,281	0,093	2,00E-07
COL1A2	1,07E-11	0,40928023	0,37	0,145	2,03E-07
EID1	1,11E-11	0,358659067	0,674	0,344	2,10E-07
AHCYL1	1,14E-11	0,291718943	0,444	0,185	2,16E-07
TGOLN2	1,18E-11	0,325580056	0,763	0,416	2,23E-07
EPB41L2	1,21E-11	0,261872127	0,296	0,1	2,29E-07
PRCP	1,43E-11	0,364441313	0,385	0,154	2,72E-07
PNP	1,49E-11	0,270102024	0,333	0,12	2,83E-07
KLHDC10	1,67E-11	0,254340505	0,259	0,083	3,17E-07
HMGN3	1,80E-11	0,292193993	0,43	0,175	3,42E-07
YWHAH	1,81E-11	0,360181288	0,415	0,17	3,44E-07
TNRC18P2	1,90E-11	0,261896374	0,319	0,114	3,59E-07
CHSY1	1,93E-11	0,299394913	0,319	0,116	3,65E-07
SRP14	2,04E-11	0,395853792	0,763	0,435	3,87E-07
ALCAM	2,33E-11	0,390374643	0,341	0,13	4,42E-07
SF3B6	2,42E-11	0,264032823	0,4	0,157	4,58E-07
RAB21	2,59E-11	0,353598968	0,341	0,126	4,90E-07
POLR2L	2,60E-11	0,410507929	0,585	0,288	4,92E-07
CD46	2,76E-11	0,290331025	0,481	0,21	5,24E-07
CAPNS1	3,53E-11	0,364212633	0,726	0,409	6,70E-07
RAB14	3,55E-11	0,302621352	0,459	0,194	6,72E-07
CDC42EP3	3,63E-11	0,407721679	0,37	0,146	6,88E-07
SEC14L1	4,46E-11	0,356567815	0,356	0,139	8,44E-07
TGFBR3	4,76E-11	0,514763518	0,43	0,189	9,02E-07
DNAJC3	5,01E-11	0,406010556	0,415	0,175	9,49E-07
SMIM7	5,19E-11	0,25614858	0,259	0,085	9,84E-07
ANXA7	5,34E-11	0,257790722	0,43	0,178	1,01E-06
TAGLN2	6,31E-11	0,363336767	0,674	0,358	1,20E-06
TRIP11	6,37E-11	0,278778845	0,311	0,113	1,21E-06
DDAH2	6,68E-11	0,26789669	0,319	0,117	1,27E-06
MAP4	6,75E-11	0,271837152	0,444	0,188	1,28E-06
ARFGEF3	7,86E-11	0,286072076	0,267	0,089	1,49E-06
EPN1	7,94E-11	0,250949632	0,304	0,109	1,50E-06
ZMYND11	8,22E-11	0,270188497	0,422	0,176	1,56E-06
SPINT2	8,29E-11	0,402172448	0,467	0,202	1,57E-06
ITFG1	8,97E-11	0,398088458	0,274	0,093	1,70E-06
NIPAL3	9,03E-11	0,25517945	0,393	0,155	1,71E-06
IMPAD1	9,33E-11	0,336828183	0,422	0,176	1,77E-06
PITPNB	1,05E-10	0,278199955	0,296	0,106	1,98E-06
KTN1	1,30E-10	0,307663533	0,704	0,381	2,46E-06
ATRAID	1,40E-10	0,301223789	0,452	0,196	2,65E-06
IGFBP5	1,41E-10	0,665973691	0,407	0,175	2,67E-06
IL13RA1	1,51E-10	0,264347815	0,422	0,18	2,85E-06
QKI	1,59E-10	0,251013712	0,578	0,271	3,01E-06
AFF4	1,85E-10	0,277190342	0,333	0,126	3,51E-06

NHP2	2,07E-10	0,250223069	0,356	0,14	3,92E-06
CRTAP	2,36E-10	0,327588661	0,659	0,339	4,46E-06
RNF10	2,45E-10	0,277349819	0,407	0,17	4,65E-06
RAB7A	2,61E-10	0,338942679	0,6	0,303	4,95E-06
LAMC1	2,75E-10	0,250719842	0,289	0,102	5,21E-06
NENF	3,01E-10	0,310272786	0,311	0,119	5,70E-06
SQSTM1	3,71E-10	0,380969226	0,593	0,293	7,02E-06
BRI3	4,15E-10	0,265318174	0,437	0,191	7,86E-06
SEC11A	4,21E-10	0,287569554	0,467	0,214	7,98E-06
ALDH3A2	4,22E-10	0,328830544	0,356	0,144	8,00E-06
MTCH1	4,82E-10	0,270144754	0,452	0,198	9,13E-06
PIK3C2A	5,02E-10	0,301672971	0,481	0,218	9,51E-06
sep-02	5,34E-10	0,341038568	0,667	0,361	1,01E-05
MESD	6,47E-10	0,291296448	0,326	0,126	1,23E-05
NUCKS1	6,87E-10	0,351262449	0,793	0,485	1,30E-05
C6orf89	7,01E-10	0,260679067	0,452	0,199	1,33E-05
ANXA2	7,04E-10	0,398131808	0,696	0,398	1,33E-05
ARL5A	7,46E-10	0,280038147	0,43	0,191	1,41E-05
SNHG7	7,64E-10	0,287954948	0,43	0,191	1,45E-05
SWAP70	7,67E-10	0,258390666	0,519	0,245	1,45E-05
GARS	7,99E-10	0,26093378	0,333	0,131	1,51E-05
DDOST	8,30E-10	0,268867494	0,533	0,253	1,57E-05
MACF1	8,67E-10	0,299354805	0,704	0,383	1,64E-05
LINC00674	9,73E-10	0,298525292	0,585	0,297	1,84E-05
SELENOF	1,02E-09	0,271887977	0,541	0,258	1,93E-05
ZBTB4	1,15E-09	0,319731285	0,422	0,189	2,18E-05
GLG1	1,20E-09	0,253383037	0,607	0,3	2,27E-05
RAB11B	1,25E-09	0,283058396	0,481	0,219	2,37E-05
DUSP6	1,38E-09	0,337055696	0,363	0,153	2,61E-05
NAA10	1,47E-09	0,295675895	0,289	0,108	2,79E-05
AFF1	1,82E-09	0,26207963	0,341	0,139	3,46E-05
PRELP	1,99E-09	0,327828563	0,578	0,303	3,78E-05
TM9SF3	2,24E-09	0,292164947	0,556	0,284	4,24E-05
HSP90AB1	2,30E-09	0,382839696	0,859	0,572	4,37E-05
CTDSP2	2,43E-09	0,250386365	0,459	0,21	4,60E-05
ISCU	2,44E-09	0,255490077	0,593	0,296	4,63E-05
VAPA	2,82E-09	0,295367188	0,504	0,246	5,34E-05
SLFN11	3,05E-09	0,25899434	0,252	0,09	5,77E-05
SMIM14	3,19E-09	0,275553921	0,304	0,12	6,04E-05
ROCK2	3,94E-09	0,342217858	0,385	0,172	7,46E-05
SH3BP2	4,50E-09	0,264789834	0,496	0,241	8,52E-05
TROVE2	4,95E-09	0,260745632	0,459	0,216	9,39E-05
PRICKLE4	5,23E-09	0,294196303	0,6	0,307	9,91E-05
YIPF5	5,43E-09	0,327756476	0,341	0,145	0,000102864
TXNRD1	6,26E-09	0,327330695	0,274	0,105	0,000118557
EMCN	7,14E-09	0,541781342	0,281	0,112	0,00013527
PEBP1	8,33E-09	0,26708031	0,719	0,405	0,000157806
SMC6	1,46E-08	0,259582588	0,259	0,099	0,000277309
COX17	1,52E-08	0,399939819	0,415	0,191	0,000288741
DYNC1H1	2,65E-08	0,250564789	0,533	0,275	0,000501796
FOXP1	2,82E-08	0,328330603	0,785	0,499	0,000534685
AP2M1	3,22E-08	0,294912461	0,511	0,268	0,000610218
RTN4	4,03E-08	0,335579621	0,77	0,451	0,000764526
IFITM2	4,04E-08	0,338188101	0,941	0,816	0,000765445
CD93	4,97E-08	0,497991211	0,296	0,129	0,000941587
WDR6	6,28E-07	0,329267889	0,289	0,129	0,011902987

HIP1	6,55E-07	0,252821852	0,4	0,197	0,012420904
MCL1	8,68E-07	0,274723704	0,674	0,406	0,016455572
CLTC	9,44E-07	0,253874986	0,57	0,316	0,017890808
CEBPD	1,00E-06	0,395899802	0,281	0,129	0,018982712
SRSF6	2,52E-06	0,315956038	0,437	0,239	0,047726275
DRAM1	4,84E-06	0,276398038	0,319	0,156	0,091797806
H3F3B	5,28E-06	0,258432549	0,837	0,592	0,099978028
MAN2B2	7,13E-06	0,287096889	0,281	0,134	0,135196468
NCOA7	7,34E-06	0,315236194	0,415	0,231	0,139146074
GOLGA2	8,51E-06	0,258705811	0,341	0,175	0,161312746
TNPO2	1,94E-05	0,265093724	0,274	0,133	0,367305655
CDKN1A	0,000361559	0,440534075	0,259	0,145	1
BCYRN1	0,000551941	0,378067347	0,341	0,201	1

Cluster 11 marker genes

gene	p_val	avg_logFC	pct.1	pct.2	p_val_adj
CD79A	0	2,824512603	0,786	0,015	0
BANK1	4,20E-280	2,186527912	0,598	0,011	7,95E-276
CD19	1,52E-182	1,579863523	0,368	0,005	2,88E-178
FAM129C	4,25E-178	1,576282347	0,402	0,008	8,05E-174
VPREB3	7,37E-177	1,496201978	0,316	0,003	1,40E-172
BCL11A	1,07E-120	1,813131746	0,47	0,028	2,04E-116
FCRLA	4,93E-114	1,733593534	0,427	0,024	9,35E-110
CD22	4,22E-107	1,577735105	0,393	0,021	8,01E-103
FCER2	2,28E-94	1,578218345	0,265	0,009	4,32E-90
CD79B	4,25E-91	1,445929161	0,385	0,025	8,06E-87
RALGPS2	1,72E-80	1,497390271	0,325	0,02	3,26E-76
IGLL5	3,88E-67	4,666903862	0,402	0,043	7,36E-63
ADAM28	1,74E-63	1,518530795	0,436	0,053	3,29E-59
P2RX5	2,44E-60	1,402923223	0,325	0,029	4,62E-56
IRF8	3,13E-58	1,380061439	0,41	0,051	5,94E-54
JCHAIN	4,06E-57	2,791451978	0,256	0,018	7,69E-53
FAM30A	1,30E-53	1,347325488	0,265	0,021	2,46E-49
HLA-DOB	6,03E-51	1,298595301	0,274	0,024	1,14E-46
MS4A1	2,74E-47	1,974151635	0,513	0,106	5,19E-43
CD74	3,24E-47	1,281301089	0,991	0,872	6,14E-43
CD37	1,83E-42	1,452571477	0,932	0,528	3,47E-38
SELL	1,67E-39	1,417361388	0,709	0,234	3,17E-35
POU2AF1	1,44E-37	1,362257211	0,385	0,068	2,74E-33
HLA-DRA	2,73E-36	1,018075383	0,932	0,551	5,18E-32
POU2F2	5,24E-35	1,331499663	0,667	0,232	9,93E-31
HVCN1	2,85E-33	1,54745183	0,376	0,076	5,40E-29
GNAO1	1,01E-30	1,059354498	0,718	0,274	1,91E-26
CD24	1,29E-29	1,220250258	0,256	0,039	2,45E-25
HLA-DRB1	7,00E-29	0,63921155	0,94	0,539	1,33E-24
WDFY4	8,92E-29	1,105048883	0,274	0,045	1,69E-24
PRKCB	1,40E-27	1,136933536	0,752	0,359	2,65E-23
HLA-DMB	4,36E-26	1,013587372	0,53	0,165	8,26E-22
HLA-DPA1	6,48E-26	0,600114271	0,94	0,612	1,23E-21
FCMR	3,81E-25	1,224046328	0,581	0,215	7,21E-21
HLA-DQA1	1,86E-23	0,807272132	0,65	0,255	3,52E-19
LTB	5,17E-23	0,982580237	0,607	0,233	9,80E-19
STRBP	2,90E-21	0,921255327	0,35	0,091	5,50E-17
HLA-DPB1	3,99E-21	0,673144178	0,786	0,456	7,55E-17
SYK	3,06E-20	1,075568303	0,479	0,18	5,79E-16
mrt-01	3,35E-20	1,032279287	0,333	0,089	6,35E-16
SEL1L3	8,39E-20	1,29927018	0,385	0,118	1,59E-15
HLA-DMA	4,94E-19	1,008445052	0,504	0,19	9,35E-15
BIRC3	9,74E-18	1,172610336	0,496	0,206	1,85E-13
TCF4	2,35E-16	1,087488913	0,53	0,24	4,45E-12
EEF2	3,40E-16	0,683742729	0,872	0,662	6,44E-12
CHD7	5,48E-16	0,827006302	0,308	0,089	1,04E-11
TPD52	2,45E-15	0,835694678	0,265	0,072	4,64E-11
SNX2	3,55E-15	0,981985422	0,376	0,142	6,73E-11
EEF1G	8,28E-15	0,64871335	0,872	0,711	1,57E-10
EEF1B2	8,78E-15	0,686627972	0,88	0,667	1,66E-10
LY86	5,00E-14	1,027677352	0,265	0,078	9,48E-10
EZR	5,07E-14	0,730786925	0,538	0,258	9,60E-10
BASP1	7,26E-14	0,952511583	0,308	0,101	1,38E-09

RIPOR2	2,51E-13	0,845817955	0,573	0,299	4,75E-09
HLA-DRB6	5,76E-13	0,839686627	0,462	0,208	1,09E-08
PABPC1	6,48E-13	0,445330859	0,966	0,821	1,23E-08
SPIB	1,07E-12	0,892770146	0,393	0,167	2,03E-08
ADAM19	2,41E-12	0,758455321	0,265	0,082	4,56E-08
CCDC50	3,06E-12	0,927988415	0,333	0,13	5,79E-08
RACK1	3,72E-12	0,507230586	0,923	0,783	7,05E-08
EEF1A1	1,10E-11	0,342636819	1	0,962	2,08E-07
SWAP70	3,08E-11	0,766177494	0,47	0,249	5,83E-07
PRDM2	5,03E-11	0,905542507	0,359	0,151	9,52E-07
ZNF395	7,06E-11	0,966700351	0,35	0,154	1,34E-06
MEF2C	7,83E-11	0,997348494	0,333	0,142	1,48E-06
BRD8	1,31E-10	0,802131828	0,53	0,302	2,49E-06
SMIM14	1,43E-10	0,972766769	0,299	0,121	2,70E-06
FCHSD2	1,50E-10	0,845623164	0,282	0,106	2,85E-06
LYN	1,71E-10	0,83907577	0,342	0,148	3,24E-06
TLR10	2,38E-10	0,668001088	0,316	0,124	4,51E-06
PNISR	6,65E-10	0,699356332	0,658	0,458	1,26E-05
FAU	1,79E-09	0,512357093	0,872	0,816	3,40E-05
CD83	4,18E-09	0,954660005	0,274	0,111	7,93E-05
MTSS1	4,32E-09	0,753455796	0,274	0,11	8,18E-05
PPM1K	7,25E-09	0,864568614	0,385	0,198	0,000137447
CD52	8,52E-09	0,573720208	0,692	0,48	0,000161469
INPP5D	2,30E-08	0,742085174	0,419	0,229	0,000436463
LAPTM5	3,41E-08	0,496581242	0,786	0,613	0,000645919
RASGRP2	3,69E-08	0,691813893	0,274	0,116	0,000698444
DDX17	5,42E-08	0,462207889	0,718	0,562	0,001026326
DNAJC10	7,93E-08	0,772545174	0,316	0,155	0,001502191
MAP3K1	8,35E-08	0,822025699	0,359	0,183	0,001581448
EVI2B	2,08E-07	0,615665978	0,564	0,358	0,003935255
NAP1L1	3,03E-07	0,534711302	0,735	0,63	0,005741898
JMJD1C	3,46E-07	0,500764382	0,453	0,269	0,006559346
HLA-DOA	4,14E-07	0,622515577	0,256	0,115	0,007848126
PTPN6	4,45E-07	0,719787242	0,299	0,15	0,008430555
MTRNR2L1	1,18E-06	0,353362566	0,658	0,476	0,022280053
GGA2	1,18E-06	0,890133619	0,299	0,155	0,02245223
PDE7A	1,37E-06	0,758498157	0,385	0,219	0,025972264
REL	2,28E-06	0,710762753	0,291	0,147	0,043204809
HLA-DRB5	2,82E-06	0,45369732	0,316	0,162	0,0535078
CYBA	3,67E-06	0,509598676	0,598	0,443	0,069635902
SP110	3,98E-06	0,692982571	0,402	0,25	0,075351665
ODC1	4,10E-06	0,744064555	0,256	0,123	0,077784138
OAZ1	4,57E-06	0,418304047	0,709	0,609	0,086526308
ADD3	5,04E-06	0,571582115	0,513	0,348	0,095524193
NCOA3	5,58E-06	0,69468819	0,316	0,176	0,105798178
ST6GAL1	6,69E-06	0,720590392	0,291	0,156	0,126702749
PTPRCAP	9,26E-06	0,368452503	0,675	0,48	0,175404089
DGKD	1,14E-05	0,626815637	0,256	0,126	0,215820921
ERP29	1,27E-05	0,585243654	0,368	0,222	0,241434473
GAS5	1,43E-05	0,452749946	0,436	0,28	0,270607105
RIPOR1	1,61E-05	0,709998347	0,265	0,139	0,305615695
sep-06	2,26E-05	0,477572015	0,496	0,341	0,428442442
MKNK2	2,80E-05	0,811788753	0,436	0,305	0,530628583
RNASET2	2,90E-05	0,521936585	0,496	0,356	0,549632455
SMN1	3,92E-05	0,578114902	0,308	0,18	0,743581286
UBA52	4,39E-05	0,28823627	0,855	0,851	0,831147

ATP2B1	7,76E-05	0,443015842	0,444	0,3	1
TOMM7	9,22E-05	0,367329103	0,718	0,592	1
CHD2	9,53E-05	0,645375041	0,41	0,274	1
SKAP2	9,98E-05	0,597106068	0,342	0,211	1
HSH2D	0,000102528	0,548065276	0,282	0,158	1
LIMD2	0,000114633	0,458579577	0,402	0,262	1
SMCHD1	0,000155816	0,490581892	0,41	0,282	1
PFDN5	0,00028783	0,406165912	0,632	0,543	1
SRRM2	0,000363735	0,479581616	0,59	0,525	1
PRPF4B	0,000366088	0,426175251	0,342	0,226	1
UBA7	0,000407838	0,504358054	0,282	0,171	1
AKAP9	0,00042577	0,437184167	0,487	0,379	1
ORAI2	0,000460631	0,465936304	0,538	0,44	1
ITSN2	0,000465144	0,597114398	0,299	0,195	1
NACA	0,000513316	0,3334762	0,675	0,576	1
UHMK1	0,000537622	0,545445394	0,368	0,254	1
CD53	0,000567117	0,462411804	0,581	0,502	1
ZFAS1	0,000585002	0,488668903	0,487	0,388	1
BTG1	0,000656804	0,348890973	0,658	0,58	1
LTA4H	0,00065799	0,593435429	0,282	0,179	1
TAPBP	0,000795657	0,328738379	0,581	0,489	1
SNHG1	0,000826993	0,575051189	0,265	0,159	1
COMMD6	0,000897763	0,420383678	0,47	0,362	1
AFTPH	0,000991876	0,445176793	0,333	0,225	1
RBM26	0,001235356	0,313650875	0,35	0,232	1
HNRNPDL	0,001264645	0,449117314	0,53	0,44	1
C6orf48	0,001312868	0,418582024	0,376	0,271	1
CNTRL	0,001425525	0,457234343	0,274	0,173	1
NLRP6	0,001528078	0,330637446	0,385	0,275	1
CUL3	0,00166611	0,486476072	0,256	0,161	1
FNBP4	0,001793439	0,602794821	0,333	0,233	1
CXCR4	0,001817478	0,508409177	0,385	0,283	1
SNHG5	0,001872413	0,335185301	0,504	0,402	1
MYCBP2	0,001930837	0,476841653	0,487	0,397	1
RUBCN	0,002026515	0,399146487	0,256	0,156	1
ANKRD44	0,002289241	0,545894091	0,368	0,271	1
mrt-06	0,002339665	0,448574335	0,41	0,321	1
IKZF3	0,002445426	0,334600283	0,538	0,429	1
HIST1H4C	0,002510552	0,464993955	0,299	0,212	1
HNRNPH1	0,002905009	0,332438341	0,65	0,56	1
NIN	0,003289346	0,362118319	0,325	0,227	1
YPEL3	0,003471263	0,308944941	0,47	0,367	1
ARGLU1	0,003610083	0,36537493	0,444	0,355	1
MAP4K4	0,004235897	0,443929562	0,282	0,193	1
MED13L	0,004485798	0,421959941	0,299	0,21	1
TNRC6B	0,004639825	0,445754215	0,479	0,392	1
PARP14	0,005050584	0,51452143	0,35	0,264	1
SEC62	0,005356991	0,397294624	0,487	0,407	1
NOP53	0,005605296	0,280979561	0,615	0,559	1
TTC3	0,005734508	0,39390819	0,402	0,319	1
EIF3H	0,006502278	0,360008307	0,479	0,396	1
OGA	0,00711184	0,311187525	0,419	0,335	1
GRB2	0,00729095	0,320591878	0,419	0,331	1
HMGN1	0,007760942	0,476061718	0,453	0,392	1
SETX	0,007979227	0,513738147	0,342	0,263	1
KIAA1551	0,008083953	0,380850855	0,419	0,332	1

PTPN2	0,008131674	0,336638573	0,376	0,286	1
PTK2B	0,008140702	0,393730301	0,436	0,346	1
METTL7A	0,008660845	0,46215096	0,325	0,239	1
BBX	0,008890108	0,493633408	0,35	0,279	1

Cluster 12 marker genes

gene	p_val	avg_logFC	pct.1	pct.2	p_val_adj
CLEC10A	1,19E-199	2,246612388	0,705	0,028	2,26E-195
FCER1A	1,36E-184	2,439438533	0,58	0,019	2,59E-180
CPVL	2,50E-139	2,234797234	0,864	0,08	4,73E-135
CD1C	1,28E-137	2,118993217	0,5	0,021	2,42E-133
NAPSB	2,29E-134	1,487300487	0,545	0,026	4,34E-130
CD1E	5,25E-113	1,256158268	0,261	0,004	9,95E-109
HLA-DQA2	1,69E-85	1,657918206	0,58	0,055	3,20E-81
LGALS2	2,80E-80	0,957780789	0,398	0,024	5,31E-76
HLA-DQB2	2,11E-78	1,42042904	0,477	0,039	4,00E-74
FCGR2B	1,27E-76	1,584184656	0,568	0,059	2,41E-72
HLA-DQA1	1,17E-74	2,296887539	0,966	0,25	2,22E-70
MS4A6A	1,48E-73	1,727813047	0,761	0,117	2,81E-69
AIF1	3,00E-73	1,381788634	0,864	0,14	5,69E-69
MNDA	3,50E-73	1,161066262	0,614	0,069	6,62E-69
GNAO1	1,42E-71	2,198758488	0,977	0,271	2,69E-67
HLA-DMB	1,04E-70	1,532374857	0,864	0,159	1,97E-66
IL1R2	1,59E-68	0,661634023	0,284	0,013	3,01E-64
LYZ	3,75E-68	2,292097067	0,943	0,248	7,11E-64
HLA-DMA	1,96E-67	1,424731671	0,898	0,182	3,71E-63
HLA-DRB6	2,12E-65	2,0040923	0,875	0,199	4,02E-61
HLA-DPB1	6,53E-63	2,359843346	1	0,453	1,24E-58
CTSH	4,31E-61	1,288702353	0,761	0,131	8,16E-57
PLD4	6,12E-60	0,87935852	0,295	0,018	1,16E-55
MPEG1	1,23E-58	1,461329747	0,693	0,113	2,33E-54
CST3	2,22E-58	2,181433579	1	0,407	4,21E-54
HLA-DRA	1,08E-57	2,286956194	1	0,552	2,04E-53
CLEC7A	1,23E-57	1,172002541	0,693	0,114	2,33E-53
HLA-DRB1	3,31E-57	2,193574429	1	0,541	6,28E-53
HLA-DPA1	1,51E-56	2,240863172	1	0,613	2,85E-52
HCK	1,73E-55	1,148382612	0,67	0,109	3,28E-51
SPI1	7,11E-55	1,208131928	0,807	0,159	1,35E-50
CD74	6,77E-52	1,940240766	1	0,873	1,28E-47
LST1	8,69E-52	1,370207541	0,625	0,106	1,65E-47
JAML	5,85E-51	1,233045235	0,67	0,123	1,11E-46
FGL2	4,05E-49	1,357989015	0,852	0,223	7,68E-45
FCGR2A	1,18E-46	1,06034903	0,5	0,071	2,24E-42
LY86	1,71E-45	0,945412955	0,5	0,073	3,24E-41
PAK1	2,40E-45	0,768632965	0,42	0,052	4,54E-41
RNASE6	6,41E-45	1,140789546	0,523	0,082	1,22E-40
TNFSF13B	8,70E-45	1,075737161	0,602	0,106	1,65E-40
BASP1	6,97E-44	1,099838038	0,557	0,096	1,32E-39
CSF1R	9,65E-44	1,190433083	0,625	0,118	1,83E-39
CFP	3,49E-43	0,868338578	0,42	0,053	6,61E-39
CD86	1,54E-42	0,958530399	0,466	0,068	2,93E-38
NAGA	1,01E-41	0,835130703	0,523	0,086	1,92E-37
RTN1	8,86E-41	0,844859363	0,341	0,039	1,68E-36
GPX1	7,13E-40	1,214271719	0,886	0,296	1,35E-35
TSPAN33	3,85E-38	0,924365776	0,33	0,039	7,30E-34
COTL1	3,37E-37	1,236959231	0,955	0,428	6,39E-33
ZNF385A	2,49E-36	0,777256302	0,58	0,115	4,72E-32
ITGAX	3,75E-36	0,972064161	0,568	0,115	7,10E-32
GCA	2,11E-35	0,757650162	0,432	0,069	3,99E-31
SERPINA1	3,86E-35	1,019258687	0,648	0,148	7,31E-31

GRN	5,15E-35	1,132264032	0,92	0,373	9,76E-31
RAB31	4,63E-34	0,878077104	0,682	0,166	8,78E-30
DUSP3	6,30E-33	0,873684729	0,511	0,104	1,19E-28
CD1D	7,65E-33	0,627108663	0,273	0,03	1,45E-28
HSPA7	1,69E-32	0,853839811	0,261	0,028	3,20E-28
mrt-01	2,47E-32	0,923380484	0,466	0,087	4,69E-28
PPT1	6,37E-32	1,088042737	0,807	0,271	1,21E-27
HLA-DOA	5,10E-31	0,759913469	0,523	0,109	9,67E-27
ALDH2	7,99E-31	1,057871239	0,511	0,112	1,51E-26
C1orf162	1,12E-30	1,024223901	0,648	0,176	2,12E-26
HLA-DRB5	1,14E-30	1,813548428	0,602	0,156	2,17E-26
SPINT2	1,68E-30	1,129905088	0,682	0,2	3,19E-26
PLBD1	1,75E-30	0,571723392	0,386	0,063	3,32E-26
TUBA1A	9,23E-30	1,022160593	0,727	0,218	1,75E-25
DSE	1,23E-29	0,77452363	0,409	0,073	2,34E-25
IL1B	2,65E-29	1,457161292	0,409	0,075	5,01E-25
CCDC88A	6,64E-29	0,982180394	0,693	0,208	1,26E-24
F13A1	1,04E-28	0,89008595	0,352	0,056	1,97E-24
SEMA4A	1,39E-28	0,581663645	0,33	0,049	2,63E-24
PEA15	3,92E-28	1,062713563	0,739	0,249	7,44E-24
IGSF6	9,69E-28	0,969431068	0,341	0,055	1,84E-23
SERPINF1	1,71E-27	0,870231916	0,364	0,062	3,23E-23
NDRG2	2,03E-27	0,641315292	0,284	0,039	3,85E-23
VSIG4	2,29E-27	1,079745858	0,409	0,079	4,34E-23
TYMP	3,72E-27	0,870653821	0,67	0,19	7,04E-23
FCER1G	4,06E-27	0,956334617	0,864	0,32	7,69E-23
LYN	4,75E-27	0,712965935	0,58	0,143	8,99E-23
CYBB	4,80E-27	0,674213488	0,636	0,159	9,09E-23
WDFY4	5,73E-27	0,826492963	0,307	0,046	1,09E-22
NCF2	5,91E-27	0,973860923	0,432	0,086	1,12E-22
CEBPD	7,08E-27	0,90069551	0,523	0,125	1,34E-22
RIN2	1,82E-26	0,818845035	0,443	0,092	3,44E-22
PID1	8,61E-26	0,564117109	0,261	0,036	1,63E-21
CLEC5A	2,44E-25	0,856780812	0,284	0,042	4,63E-21
CSF2RB	3,78E-25	0,615783822	0,42	0,086	7,16E-21
FPR1	4,94E-25	0,740136301	0,364	0,067	9,35E-21
TGFBI	5,45E-25	0,517661504	0,58	0,143	1,03E-20
CX3CR1	5,99E-25	0,65215461	0,375	0,069	1,14E-20
PLEKHO1	8,33E-25	0,724908016	0,545	0,138	1,58E-20
PYCARD	8,43E-25	0,814454523	0,591	0,163	1,60E-20
FCGRT	1,23E-24	0,800314873	0,795	0,286	2,34E-20
SYK	1,37E-24	0,725171623	0,625	0,178	2,60E-20
CALHM6	2,17E-24	0,580163652	0,33	0,056	4,12E-20
ACTB	3,07E-24	0,804410565	1	0,977	5,81E-20
THEMIS2	6,25E-24	0,64033245	0,409	0,085	1,18E-19
NR4A3	7,50E-24	0,677381843	0,273	0,041	1,42E-19
FTH1	1,59E-23	0,808733837	1	0,81	3,02E-19
KCTD12	1,86E-23	0,873081539	0,636	0,194	3,53E-19
MAN2B1	2,80E-23	0,782644272	0,557	0,157	5,31E-19
GSTP1	8,58E-23	0,93342012	0,75	0,281	1,63E-18
TBC1D9	1,31E-22	0,659991231	0,295	0,051	2,47E-18
PLSCR1	1,46E-22	0,643233606	0,409	0,092	2,76E-18
TUBA1B	1,82E-22	1,088474458	0,864	0,405	3,46E-18
FGR	4,70E-22	0,654568758	0,557	0,153	8,91E-18
GGTA1P	5,90E-22	0,667866818	0,33	0,064	1,12E-17
ANPEP	7,11E-22	0,515577687	0,364	0,073	1,35E-17

AP1S2	8,45E-22	0,7445412	0,58	0,172	1,60E-17
DPYSL2	8,72E-22	0,778445868	0,659	0,211	1,65E-17
CMTM6	9,03E-22	0,772272213	0,83	0,338	1,71E-17
MRAP	1,02E-21	0,552284945	0,295	0,051	1,93E-17
TMSB10	2,16E-21	0,668429431	0,989	0,865	4,09E-17
CXCL8	2,22E-21	1,026956887	0,364	0,076	4,21E-17
CXCL16	2,25E-21	0,843951037	0,614	0,196	4,25E-17
ALCAM	2,43E-21	0,692360607	0,489	0,129	4,60E-17
PTPRE	3,03E-21	0,778123339	0,58	0,173	5,75E-17
KLF4	3,03E-21	0,772880951	0,432	0,107	5,75E-17
IFI30	3,73E-21	1,077631079	0,886	0,448	7,07E-17
GPR183	4,02E-21	0,679919262	0,489	0,124	7,61E-17
CD83	6,63E-21	0,678587953	0,443	0,108	1,26E-16
CDK2AP1	8,82E-21	0,780407477	0,489	0,136	1,67E-16
NAMPT	1,82E-20	1,058690715	0,58	0,193	3,45E-16
CSTA	2,54E-20	0,592537331	0,284	0,051	4,80E-16
RBM47	3,38E-20	0,564851271	0,557	0,16	6,40E-16
BTK	4,83E-20	0,480102717	0,284	0,051	9,15E-16
RPPH1	4,93E-20	0,706354896	0,261	0,044	9,34E-16
PLEK	5,18E-20	0,765970783	0,67	0,222	9,82E-16
TBXAS1	5,79E-20	0,517589349	0,364	0,079	1,10E-15
AXL	5,84E-20	0,672281123	0,284	0,053	1,11E-15
SHTN1	9,52E-20	0,669464347	0,318	0,065	1,80E-15
TLR2	9,62E-20	0,69778997	0,33	0,07	1,82E-15
GDI2	1,00E-19	0,79571155	0,739	0,297	1,90E-15
GSN	1,39E-19	0,353824948	0,83	0,308	2,64E-15
RNF130	1,41E-19	0,724595833	0,523	0,156	2,66E-15
CLIC2	1,49E-19	0,576754032	0,318	0,066	2,82E-15
GRINA	1,51E-19	0,52737897	0,477	0,127	2,87E-15
SIRPA	1,80E-19	0,666212916	0,443	0,116	3,41E-15
RGS10	2,36E-19	0,680648441	0,568	0,178	4,47E-15
LGALS9	3,12E-19	0,565849457	0,398	0,096	5,91E-15
TYROBP	3,98E-19	0,828410334	0,864	0,448	7,54E-15
IFNGR1	5,03E-19	1,006290427	0,523	0,169	9,53E-15
PPA1	1,23E-18	0,598773008	0,511	0,15	2,34E-14
ALOX5	1,46E-18	0,777916013	0,409	0,105	2,77E-14
IL13RA1	1,77E-18	0,9747674	0,534	0,18	3,36E-14
H2AFY	1,95E-18	0,859379703	0,705	0,297	3,69E-14
CSF3R	2,35E-18	0,394260574	0,307	0,062	4,45E-14
CTSS	2,38E-18	0,65591327	0,83	0,381	4,50E-14
PARVG	2,40E-18	0,603537252	0,477	0,135	4,54E-14
FAM49B	2,78E-18	0,650391397	0,705	0,252	5,28E-14
RNASET2	2,91E-18	0,749008536	0,795	0,349	5,51E-14
ADA2	2,98E-18	0,807928861	0,625	0,224	5,65E-14
CAT	3,17E-18	0,622795301	0,511	0,155	6,01E-14
IRF8	4,52E-18	0,809235845	0,284	0,058	8,57E-14
EFHD2	5,05E-18	0,737440961	0,705	0,281	9,58E-14
TMSB4X	6,95E-18	0,611372875	1	0,934	1,32E-13
PARL	9,13E-18	0,544765536	0,398	0,101	1,73E-13
ETV6	9,57E-18	0,748564767	0,523	0,168	1,81E-13
SLAMF8	1,24E-17	0,422658817	0,261	0,049	2,35E-13
RNF144B	1,91E-17	0,701215066	0,295	0,063	3,61E-13
GM2A	2,11E-17	0,610520764	0,443	0,123	4,01E-13
SAT1	2,39E-17	0,595041451	0,818	0,356	4,53E-13
RAB32	2,78E-17	0,624880877	0,295	0,064	5,27E-13
PSAP	3,22E-17	0,486823724	0,955	0,549	6,10E-13

ITGB2	8,67E-17	0,679739424	0,875	0,433	1,64E-12
GRK3	9,77E-17	0,524557222	0,398	0,106	1,85E-12
C1orf54	1,11E-16	0,861132727	0,295	0,069	2,10E-12
LRRK2	1,53E-16	0,364760019	0,261	0,052	2,90E-12
MYD88	1,53E-16	0,597787963	0,443	0,128	2,90E-12
SYNGR2	1,79E-16	0,635212756	0,58	0,2	3,38E-12
CD4	1,85E-16	0,656804804	0,818	0,371	3,51E-12
RBPJ	1,87E-16	0,676947699	0,773	0,35	3,55E-12
CD93	3,53E-16	0,55475408	0,443	0,128	6,69E-12
AP2S1	3,89E-16	0,591023316	0,614	0,225	7,37E-12
PABPC4	4,44E-16	0,793744943	0,705	0,324	8,42E-12
UNC93B1	4,53E-16	0,514765695	0,42	0,117	8,59E-12
LAT2	5,17E-16	0,522587301	0,33	0,078	9,80E-12
RGS2	5,99E-16	0,783137606	0,398	0,112	1,14E-11
GRASP	7,05E-16	0,593083141	0,284	0,063	1,34E-11
IFNGR2	7,17E-16	0,538176658	0,443	0,134	1,36E-11
FPR3	8,40E-16	0,61447902	0,261	0,054	1,59E-11
RHOG	8,70E-16	0,685505891	0,58	0,212	1,65E-11
EPB41L3	1,25E-15	0,541024416	0,307	0,073	2,36E-11
NPC2	1,67E-15	0,572931844	0,761	0,313	3,17E-11
FTL	2,02E-15	0,416027451	0,977	0,744	3,82E-11
ZFP36	2,19E-15	0,662895286	0,67	0,262	4,14E-11
SNX10	2,28E-15	0,460189539	0,398	0,109	4,32E-11
RCC2	2,37E-15	0,516893876	0,466	0,148	4,50E-11
ATP2B1	2,90E-15	0,662484017	0,693	0,294	5,49E-11
LSP1	2,93E-15	0,688156862	0,761	0,363	5,55E-11
TMEM107	3,60E-15	0,510715191	0,386	0,107	6,82E-11
FAM49A	4,15E-15	0,491284341	0,33	0,083	7,87E-11
SECTM1	4,39E-15	0,464798868	0,284	0,064	8,31E-11
SAMHD1	7,10E-15	0,565868737	0,375	0,106	1,34E-10
C20orf27	7,18E-15	0,377191523	0,261	0,056	1,36E-10
FBP1	7,25E-15	0,62522985	0,318	0,079	1,37E-10
USB1	8,68E-15	0,446177157	0,318	0,08	1,65E-10
ATG16L2	1,06E-14	0,519942016	0,375	0,104	2,01E-10
RTN4	1,26E-14	0,656339823	0,83	0,454	2,39E-10
TPP1	1,59E-14	0,568652283	0,864	0,407	3,02E-10
VASP	1,62E-14	0,481743554	0,557	0,196	3,08E-10
MFSD1	1,74E-14	0,49415713	0,477	0,156	3,30E-10
OLR1	2,21E-14	0,747590995	0,261	0,058	4,19E-10
KDM2B	2,52E-14	0,374698734	0,295	0,07	4,78E-10
IGFLR1	3,38E-14	0,519853244	0,295	0,073	6,41E-10
FOXN2	3,50E-14	0,498926349	0,295	0,072	6,64E-10
SLC15A3	3,58E-14	0,441325601	0,295	0,072	6,78E-10
MGAT1	4,86E-14	0,68944603	0,523	0,195	9,22E-10
KLF5	7,11E-14	0,494188619	0,614	0,233	1,35E-09
GABARAP	8,10E-14	0,699593398	0,841	0,54	1,54E-09
ATP6V0D1	9,86E-14	0,535279063	0,409	0,128	1,87E-09
AMPD2	1,00E-13	0,592004424	0,273	0,065	1,89E-09
ATP6V1B2	1,03E-13	0,492554073	0,466	0,157	1,95E-09
ACTG1	1,12E-13	0,62011415	0,989	0,813	2,12E-09
VASH1	1,25E-13	0,622700157	0,341	0,097	2,37E-09
PLXNB2	1,41E-13	0,462742515	0,409	0,126	2,67E-09
AMPD3	1,67E-13	0,472372507	0,295	0,074	3,16E-09
ARF5	1,95E-13	0,579700058	0,477	0,168	3,69E-09
CNPY3	1,99E-13	0,58733907	0,523	0,191	3,77E-09
RUNX1	2,89E-13	0,590956405	0,398	0,122	5,48E-09

PLAUR	3,08E-13	0,817627793	0,443	0,153	5,84E-09
ARPC1B	3,17E-13	0,576726971	0,75	0,377	6,00E-09
SH3BGRL	3,28E-13	0,488374997	0,75	0,339	6,22E-09
RASSF2	3,41E-13	0,502981646	0,42	0,135	6,47E-09
ATP6V0B	3,49E-13	0,42866763	0,659	0,26	6,61E-09
STX7	4,20E-13	0,401288099	0,375	0,112	7,96E-09
CHP1	5,67E-13	0,439528365	0,5	0,179	1,07E-08
IL18	6,66E-13	0,557158585	0,33	0,094	1,26E-08
S100A11	7,56E-13	0,528904228	0,977	0,585	1,43E-08
CBX6	8,62E-13	0,5115649	0,477	0,172	1,63E-08
RNPEP	8,91E-13	0,36355667	0,364	0,107	1,69E-08
RAB7A	9,87E-13	0,480712284	0,693	0,305	1,87E-08
AHR	1,05E-12	0,76426047	0,477	0,183	1,99E-08
SLC25A5	1,27E-12	0,608256693	0,659	0,299	2,41E-08
AOAH	1,38E-12	0,491170159	0,375	0,115	2,62E-08
PTTG1IP	2,10E-12	0,486624286	0,602	0,247	3,98E-08
CEP170	2,14E-12	0,484812108	0,398	0,131	4,05E-08
RAC1	2,18E-12	0,485593129	0,875	0,497	4,14E-08
HMGA1	2,41E-12	0,507509378	0,523	0,199	4,56E-08
CD163	2,90E-12	0,577469136	0,352	0,107	5,49E-08
UVRAG	3,64E-12	0,444347384	0,33	0,097	6,90E-08
CD14	3,79E-12	0,369397935	0,455	0,154	7,18E-08
CDKN1A	4,84E-12	0,698024364	0,409	0,142	9,17E-08
ARPC3	4,86E-12	0,624417407	0,75	0,426	9,21E-08
VAMP8	5,00E-12	0,347869179	0,523	0,192	9,48E-08
C1QA	5,33E-12	0,5618714	0,364	0,113	1,01E-07
PON2	5,97E-12	0,434379845	0,261	0,067	1,13E-07
IER3	7,03E-12	0,792523256	0,455	0,171	1,33E-07
ADAM8	8,66E-12	0,384736629	0,455	0,155	1,64E-07
LTA4H	9,13E-12	0,605352859	0,466	0,174	1,73E-07
CASP1	1,24E-11	0,50764857	0,398	0,134	2,34E-07
RB1	1,34E-11	0,4593413	0,375	0,121	2,54E-07
SEC13	1,52E-11	0,340860612	0,375	0,12	2,88E-07
MTCH2	1,69E-11	0,423060664	0,318	0,095	3,20E-07
SUMO3	1,79E-11	0,46798863	0,5	0,186	3,39E-07
LAMTOR1	1,81E-11	0,388645126	0,409	0,14	3,43E-07
EVI2A	2,09E-11	0,461295899	0,534	0,211	3,95E-07
METRNL	2,10E-11	0,434240321	0,386	0,131	3,98E-07
MEF2C	2,18E-11	0,412989629	0,409	0,142	4,13E-07
CYBA	2,88E-11	0,469399442	0,841	0,437	5,46E-07
LILRB2	3,20E-11	0,524634913	0,364	0,118	6,06E-07
RHOQ	3,61E-11	0,448675958	0,443	0,162	6,84E-07
C1QB	3,66E-11	0,464133946	0,409	0,144	6,94E-07
PLXNC1	4,21E-11	0,286736103	0,307	0,088	7,97E-07
SH2B3	4,81E-11	0,457353149	0,398	0,134	9,11E-07
ARPC4	4,93E-11	0,443627882	0,739	0,364	9,34E-07
HADHB	5,82E-11	0,412436462	0,432	0,157	1,10E-06
ADPGK	6,41E-11	0,297186892	0,33	0,101	1,21E-06
APEX1	6,45E-11	0,464411238	0,42	0,15	1,22E-06
OTULINL	7,66E-11	0,342426241	0,284	0,08	1,45E-06
KIAA0930	7,84E-11	0,512108419	0,42	0,155	1,49E-06
PSMB3	9,24E-11	0,384997642	0,523	0,21	1,75E-06
BICD2	1,12E-10	0,462559569	0,386	0,133	2,12E-06
ATP6AP2	1,36E-10	0,425210541	0,682	0,322	2,58E-06
POU2F2	1,44E-10	0,608996554	0,545	0,239	2,73E-06
LAMTOR2	1,51E-10	0,358839447	0,33	0,106	2,86E-06

PLCB2	1,56E-10	0,390386618	0,432	0,155	2,96E-06
SF3B5	1,59E-10	0,42452672	0,466	0,177	3,01E-06
RAB8A	1,62E-10	0,473880949	0,398	0,142	3,07E-06
PRCP	1,64E-10	0,37038146	0,42	0,157	3,11E-06
LPCAT2	1,64E-10	0,436997163	0,318	0,101	3,12E-06
AP3D1	1,84E-10	0,488549372	0,477	0,192	3,48E-06
PER1	2,17E-10	0,389876937	0,33	0,106	4,12E-06
EDEM1	2,32E-10	0,398616601	0,386	0,134	4,41E-06
YWHAH	2,70E-10	0,563898099	0,443	0,173	5,12E-06
UBE2J1	2,92E-10	0,346583633	0,375	0,128	5,53E-06
DOCK2	3,89E-10	0,320528043	0,477	0,178	7,37E-06
SERP1	4,31E-10	0,433904188	0,727	0,349	8,17E-06
CHCHD7	4,51E-10	0,339999036	0,261	0,075	8,55E-06
NDUFS7	4,52E-10	0,339074377	0,364	0,124	8,57E-06
QKI	4,56E-10	0,539102505	0,614	0,275	8,65E-06
INSIG1	4,85E-10	0,626641736	0,295	0,094	9,19E-06
PPP1CB	5,19E-10	0,498719486	0,727	0,369	9,83E-06
MAFB	5,47E-10	0,4102525	0,432	0,162	1,04E-05
MCL1	5,53E-10	0,47217632	0,773	0,408	1,05E-05
SULF2	5,85E-10	0,387581621	0,284	0,087	1,11E-05
KCNK6	5,85E-10	0,534220153	0,443	0,171	1,11E-05
PRKCD	5,97E-10	0,458807791	0,284	0,088	1,13E-05
GUSB	6,08E-10	0,407861512	0,295	0,093	1,15E-05
BCL2A1	6,13E-10	0,386505823	0,261	0,074	1,16E-05
TPI1	6,89E-10	0,402273169	0,648	0,301	1,31E-05
ATP6V1A	7,37E-10	0,37337129	0,466	0,189	1,40E-05
OAZ1	7,58E-10	0,485684298	0,864	0,606	1,44E-05
ZFP36L1	7,69E-10	0,558864123	0,875	0,552	1,46E-05
NFKBIZ	7,80E-10	0,401699544	0,489	0,2	1,48E-05
HEXA	8,00E-10	0,352834589	0,341	0,114	1,52E-05
DYNC1LI1	8,29E-10	0,503192443	0,295	0,094	1,57E-05
FAM129A	8,45E-10	0,546242276	0,602	0,29	1,60E-05
CACUL1	1,01E-09	0,443550738	0,386	0,147	1,91E-05
STK38L	1,01E-09	0,39215401	0,273	0,084	1,92E-05
MBOAT7	1,05E-09	0,355482586	0,318	0,105	1,99E-05
SSR3	1,16E-09	0,431478357	0,557	0,254	2,19E-05
NCOA4	1,23E-09	0,439429645	0,568	0,245	2,33E-05
ARPC5	1,28E-09	0,522394869	0,705	0,374	2,42E-05
SNAP23	1,31E-09	0,332094721	0,42	0,158	2,48E-05
RHOA	1,46E-09	0,415978438	0,92	0,593	2,76E-05
SRRM4	1,75E-09	0,581525876	0,284	0,092	3,31E-05
PICALM	1,93E-09	0,463672342	0,511	0,219	3,65E-05
PAPSS2	1,98E-09	0,342871164	0,261	0,079	3,75E-05
DBNL	2,09E-09	0,472704675	0,398	0,155	3,96E-05
PRPF19	2,15E-09	0,347276316	0,284	0,09	4,08E-05
CUX1	2,17E-09	0,51225064	0,466	0,193	4,10E-05
LCP1	2,20E-09	0,437702081	0,977	0,696	4,17E-05
MLEC	2,27E-09	0,389869025	0,409	0,158	4,30E-05
NOP10	2,28E-09	0,307169379	0,375	0,135	4,33E-05
MS4A4A	2,33E-09	0,493914115	0,489	0,198	4,41E-05
ARF3	2,42E-09	0,37149723	0,534	0,235	4,59E-05
HNRNPAB	2,60E-09	0,498522541	0,477	0,205	4,94E-05
ABHD12	2,65E-09	0,402644384	0,307	0,103	5,03E-05
CYTH4	2,68E-09	0,473588527	0,352	0,126	5,09E-05
YBX1	2,83E-09	0,454600011	0,875	0,566	5,36E-05
WARS	2,94E-09	0,35264681	0,352	0,126	5,58E-05

SEC11A	3,04E-09	0,435296045	0,5	0,217	5,76E-05
PACSIN2	3,15E-09	0,329171501	0,273	0,085	5,97E-05
PGLS	3,39E-09	0,284480846	0,33	0,112	6,43E-05
BRK1	3,56E-09	0,409946294	0,625	0,314	6,75E-05
ATG3	3,70E-09	0,415507956	0,398	0,156	7,01E-05
YBX3	3,90E-09	0,270996293	0,477	0,19	7,39E-05
HERPUD1	4,34E-09	0,452595441	0,42	0,17	8,23E-05
SNX3	4,60E-09	0,67498993	0,466	0,207	8,72E-05
SGK1	4,66E-09	0,343285679	0,341	0,12	8,84E-05
MIS18BP1	5,12E-09	0,420370252	0,352	0,13	9,71E-05
CCS	5,41E-09	0,302094438	0,261	0,08	0,000102507
TNFAIP2	5,74E-09	0,292582763	0,375	0,141	0,000108748
PABPC1	5,90E-09	0,367801337	0,977	0,822	0,000111772
CYFIP1	6,22E-09	0,482047574	0,455	0,198	0,000117788
FOS	6,25E-09	0,423981362	0,739	0,377	0,000118348
SRGN	6,42E-09	0,510441229	0,92	0,722	0,000121715
ALKBH7	6,47E-09	0,419794339	0,273	0,087	0,000122566
HBEGF	6,74E-09	0,646660592	0,273	0,09	0,00012767
NAP1L1	7,25E-09	0,408128648	0,932	0,626	0,000137321
PRSS8	7,26E-09	0,451031386	0,852	0,641	0,0001376
MICAL1	7,37E-09	0,306382731	0,364	0,13	0,000139743
TSPO	7,46E-09	0,519220513	0,58	0,276	0,000141383
GLIPR1	9,56E-09	0,313789549	0,489	0,207	0,000181202
BCL3	9,86E-09	0,334066141	0,284	0,093	0,00018688
DUSP2	1,12E-08	0,62230327	0,284	0,097	0,000212539
LITAF	1,14E-08	0,520702763	0,807	0,502	0,000215398
OSTC	1,15E-08	0,428876986	0,409	0,168	0,000217281
MRPL3	1,25E-08	0,32687921	0,273	0,089	0,000237337
RNH1	1,26E-08	0,466762811	0,466	0,209	0,00023835
S100A10	1,28E-08	0,426405589	0,852	0,521	0,000242914
SEC61B	1,29E-08	0,421540224	0,511	0,231	0,000244426
RGS19	1,32E-08	0,432058625	0,375	0,146	0,00025101
GNAI2	1,36E-08	0,44229944	0,886	0,653	0,000257597
COX7B	1,44E-08	0,377835789	0,625	0,302	0,000272003
PIK3AP1	1,64E-08	0,300115743	0,341	0,123	0,000311562
DEGS1	1,70E-08	0,323246703	0,455	0,19	0,000321681
TRIM8	1,71E-08	0,496034455	0,307	0,111	0,000324477
SF3B4	1,72E-08	0,537143326	0,455	0,198	0,000325423
PTAFR	1,82E-08	0,375169317	0,42	0,173	0,000344993
BRD8	1,96E-08	0,455231296	0,602	0,302	0,000371204
ARRB2	2,18E-08	0,422486666	0,591	0,282	0,000413634
NDUFB1	2,37E-08	0,377129143	0,466	0,204	0,000448362
FCN1	2,40E-08	0,564726699	0,341	0,129	0,000453902
MEF2A	2,43E-08	0,555257965	0,386	0,163	0,00046048
VSIR	2,52E-08	0,489439011	0,375	0,153	0,000477673
PKM	2,55E-08	0,418568808	0,875	0,597	0,000482332
TTYH3	2,56E-08	0,386872829	0,33	0,12	0,000485974
TAOK3	2,82E-08	0,330713694	0,614	0,3	0,000534175
ACTR2	2,96E-08	0,414158202	0,807	0,516	0,000560203
ATP5F1EP2	3,13E-08	0,363573726	0,739	0,402	0,000592381
GOLGA8S	3,28E-08	0,334988301	0,318	0,115	0,000620696
CAMTA1	3,35E-08	0,383441753	0,364	0,143	0,000633888
RAB5C	3,40E-08	0,350755469	0,398	0,162	0,000644634
CDC42	3,52E-08	0,453774062	0,83	0,537	0,00066775
VOPP1	3,55E-08	0,431048182	0,545	0,254	0,000672151
NOTCH2	3,76E-08	0,251996337	0,341	0,126	0,00071318

SUPT4H1	3,83E-08	0,516719196	0,341	0,133	0,000726716
ACAA1	3,84E-08	0,355561282	0,273	0,092	0,000727267
PFN1	4,35E-08	0,397855592	0,977	0,869	0,000825262
COX5A	4,39E-08	0,403142916	0,466	0,209	0,000832332
DENND1B	4,39E-08	0,343803869	0,364	0,14	0,000832692
EVI2B	5,45E-08	0,323558111	0,682	0,357	0,001033266
CD68	5,46E-08	0,33297553	0,477	0,212	0,001035116
BANF1	5,47E-08	0,342379345	0,545	0,256	0,001036666
SESN3	5,70E-08	0,317718273	0,318	0,115	0,001079502
CHMP1B	6,05E-08	0,383294285	0,341	0,132	0,00114612
VPS35	6,50E-08	0,354423097	0,466	0,205	0,001231336
PITPNA	6,76E-08	0,328139426	0,341	0,131	0,001280473
LSM1	6,79E-08	0,264131841	0,295	0,105	0,001286562
NAAA	6,86E-08	0,417320334	0,432	0,188	0,001299484
NAGK	8,40E-08	0,462076901	0,432	0,194	0,001592671
RAP1GDS1	9,36E-08	0,311997253	0,284	0,101	0,001774242
STARD7	9,54E-08	0,278371502	0,364	0,146	0,00180876
VPS29	9,56E-08	0,319793175	0,466	0,209	0,001811253
SERPINB9	9,67E-08	0,716580407	0,42	0,192	0,00183197
DDX21	1,06E-07	0,465790937	0,591	0,302	0,00201453
ADAP2	1,10E-07	0,325817055	0,295	0,106	0,002088095
TMEM219	1,14E-07	0,462438327	0,489	0,24	0,002158124
MTHFD2	1,18E-07	0,467862103	0,318	0,121	0,002232339
AHCYL1	1,31E-07	0,332402357	0,432	0,189	0,002488101
CBL	1,37E-07	0,452137576	0,307	0,118	0,0025952
HCLS1	1,37E-07	0,40647641	0,75	0,452	0,002601864
ETFA	1,44E-07	0,309762349	0,307	0,116	0,00272156
RIN3	1,64E-07	0,263125719	0,261	0,089	0,003111953
ZMIZ1	1,67E-07	0,42118226	0,318	0,121	0,003165515
EIF3L	1,79E-07	0,309302394	0,727	0,414	0,003389246
HEXB	1,79E-07	0,298062152	0,352	0,142	0,003392168
APH1A	1,97E-07	0,386873632	0,307	0,118	0,003739578
ATP1B3	2,00E-07	0,369427151	0,58	0,29	0,003791358
PCBP2	2,03E-07	0,352319267	0,852	0,542	0,003854661
TIMM8B	2,34E-07	0,300836024	0,261	0,092	0,004428658
RFX5	2,52E-07	0,333644186	0,273	0,098	0,004783426
SSR1	2,64E-07	0,313625974	0,693	0,382	0,004999353
PLEKHB2	2,68E-07	0,389069579	0,545	0,268	0,005080721
ETS2	2,84E-07	0,445803041	0,295	0,116	0,005387873
TMEM14C	2,87E-07	0,457364434	0,341	0,146	0,005433728
SERPINB1	2,95E-07	0,321283892	0,5	0,237	0,005593167
GLRX	3,03E-07	0,304650694	0,341	0,137	0,00575032
MAP3K13	3,20E-07	0,360565816	0,364	0,152	0,006064939
GLUL	3,24E-07	0,414532142	0,557	0,287	0,006131904
DAZAP2	3,58E-07	0,335430425	0,807	0,498	0,006784871
TRPS1	3,79E-07	0,511754436	0,261	0,096	0,007176224
LOC441081	4,01E-07	0,28792069	0,341	0,137	0,007608131
NDUFB2	4,45E-07	0,34380252	0,489	0,236	0,008440793
MTRNR2L1	4,51E-07	0,429142205	0,773	0,474	0,008541588
CLIC1	4,58E-07	0,324569685	0,761	0,444	0,008683983
COX6B1	4,90E-07	0,307333441	0,693	0,371	0,009287031
UCP2	6,14E-07	0,426742756	0,682	0,398	0,011629381
CREG1	6,21E-07	0,260444235	0,364	0,15	0,011766737
ATP5MC2	6,90E-07	0,37463711	0,795	0,523	0,01307861
PGK1	7,06E-07	0,523403332	0,682	0,42	0,013384401
GSTO1	7,32E-07	0,349102545	0,42	0,197	0,013872924

GNB2	7,36E-07	0,325802562	0,443	0,207	0,013945354
NUMB	7,67E-07	0,352988611	0,284	0,112	0,014534458
SATB1	7,98E-07	0,362083418	0,455	0,205	0,015127735
ECHS1	8,11E-07	0,272387331	0,273	0,103	0,015368766
ADGRE2	8,18E-07	0,362430061	0,318	0,128	0,015495342
BLOC1S6	8,38E-07	0,322716748	0,455	0,215	0,015873774
EIF6	9,48E-07	0,288903054	0,591	0,299	0,017964799
ENO1	9,50E-07	0,416751684	0,727	0,487	0,017996907
PTMA	9,58E-07	0,304805033	0,989	0,878	0,018162183
IL6R	1,03E-06	0,372487368	0,455	0,214	0,019518581
SERF2	1,05E-06	0,352033786	0,909	0,7	0,019987959
GNG5	1,11E-06	0,300887713	0,545	0,276	0,020972067
GRB2	1,14E-06	0,320603994	0,602	0,327	0,021670569
CIAO2A	1,17E-06	0,378726366	0,307	0,126	0,022209731
FABP5	1,18E-06	0,404584038	0,375	0,163	0,022367387
ATP5F1C	1,19E-06	0,387313387	0,477	0,242	0,022458898
OS9	1,23E-06	0,319006858	0,534	0,275	0,023382898
MARCKS	1,27E-06	0,265419132	0,489	0,229	0,023985082
PHB2	1,40E-06	0,259977077	0,489	0,235	0,026441244
AKR1B1	1,44E-06	0,400722171	0,364	0,162	0,027206971
ITGB2-AS1	1,48E-06	0,270418299	0,307	0,121	0,028039437
ATP5ME	1,49E-06	0,283668444	0,568	0,293	0,028289172
LGALS3	1,57E-06	0,475350774	0,477	0,24	0,029770362
APPL1	1,58E-06	0,256873536	0,341	0,144	0,029963621
PTPA	1,63E-06	0,551464422	0,318	0,142	0,030918256
RNF149	2,15E-06	0,340973425	0,443	0,21	0,04075444
CANX	2,21E-06	0,292848714	0,761	0,438	0,041885603
LAPTM5	2,29E-06	0,323185343	0,909	0,611	0,043440086
SDHC	2,54E-06	0,282379641	0,307	0,127	0,048047239
SND1	2,54E-06	0,317620607	0,375	0,168	0,048067625
AP1B1	2,60E-06	0,26747985	0,341	0,146	0,049273266
AURKAIP1	3,10E-06	0,332024768	0,295	0,124	0,058724409
FKBP1A	3,10E-06	0,366282166	0,716	0,458	0,058817496
NAIP	3,17E-06	0,264871758	0,33	0,14	0,059989773
SOD2	3,51E-06	0,539966701	0,443	0,221	0,066576117
CALR	3,72E-06	0,351663464	0,67	0,39	0,070506151
RNU4ATAC	4,12E-06	0,286251669	0,375	0,17	0,077981344
LGALS1	4,26E-06	0,274115571	0,648	0,361	0,080704949
EPB41L2	4,28E-06	0,282144368	0,261	0,104	0,081055711
RALA	4,41E-06	0,343947424	0,33	0,144	0,083593458
ENY2	4,68E-06	0,286832939	0,318	0,138	0,088756951
ACTR3	4,92E-06	0,271370183	0,625	0,353	0,093315734
MTDH	5,61E-06	0,288398942	0,75	0,495	0,106225953
NR4A2	5,84E-06	0,438737553	0,409	0,209	0,110605884
ATP5F1D	6,33E-06	0,292956215	0,352	0,161	0,119892628
BCAT1	6,75E-06	0,299500165	0,352	0,159	0,12783087
TUBGCP2	6,80E-06	0,295687829	0,261	0,106	0,12887524
KHSRP	7,83E-06	0,302656441	0,466	0,237	0,148353634
UQCRH	8,06E-06	0,276492806	0,375	0,172	0,152777512
CFL1	8,17E-06	0,305872968	0,932	0,873	0,154911757
ANXA2	8,35E-06	0,256393994	0,705	0,402	0,15816345
PTPN6	8,87E-06	0,258636206	0,341	0,15	0,167994313
APLP2	9,27E-06	0,294093261	0,693	0,395	0,175642865
MLXIP	1,10E-05	0,311649734	0,273	0,114	0,208535081
C1QC	1,31E-05	0,291196548	0,273	0,113	0,248616909
NDUFV2	1,37E-05	0,300252138	0,352	0,167	0,259008577

CARD16	1,50E-05	0,254137987	0,261	0,107	0,283453854
ARPC2	1,57E-05	0,25879726	0,943	0,772	0,297105739
CAPNS1	1,76E-05	0,307887425	0,693	0,414	0,334371098
RASA4	1,85E-05	0,303753454	0,352	0,168	0,350755359
F11R	2,02E-05	0,298086046	0,261	0,109	0,38286303
SNRPF	2,14E-05	0,271858197	0,375	0,181	0,406049613
ATP5MC3	2,32E-05	0,33111808	0,477	0,269	0,440383664
TM9SF2	2,45E-05	0,269531338	0,455	0,236	0,46488909
UBE2R2	2,82E-05	0,320467799	0,386	0,196	0,534794247
PCBP1	2,93E-05	0,280429224	0,761	0,485	0,554607407
RAB10	3,00E-05	0,327163885	0,42	0,212	0,567789268
EIF4H	3,16E-05	0,311690551	0,625	0,379	0,598754244
H2AFZ	3,24E-05	0,278800594	0,466	0,239	0,613669146
DPYD	3,34E-05	0,279627038	0,284	0,126	0,632347037
mrt-02	3,68E-05	0,288087942	0,284	0,13	0,697971188
DDAH2	3,73E-05	0,252352839	0,273	0,121	0,707360502
REL	4,37E-05	0,250409839	0,318	0,147	0,828981772
MOB3A	4,45E-05	0,359026481	0,386	0,198	0,842906617
ARL8B	4,46E-05	0,318022952	0,307	0,144	0,845365156
PEPD	4,47E-05	0,329632417	0,307	0,145	0,846377459
GADD45B	5,88E-05	0,310084286	0,364	0,188	1
ZYX	6,64E-05	0,376605484	0,625	0,402	1
CMIP	7,50E-05	0,420013051	0,58	0,354	1
SLC25A3	9,24E-05	0,25641191	0,636	0,373	1
SNRPG	9,41E-05	0,274466748	0,33	0,162	1
MYL6	9,60E-05	0,313101739	0,886	0,718	1
POLR1D	0,000102506	0,312883822	0,318	0,158	1
ITPK1	0,000104047	0,282225725	0,318	0,156	1
SH3BP2	0,000106012	0,313476307	0,443	0,246	1
ARL8A	0,000107972	0,251404817	0,273	0,127	1
COX8A	0,000111391	0,255752879	0,614	0,378	1
PPIA	0,000121463	0,287852753	0,92	0,783	1
VMP1	0,000132249	0,304071688	0,364	0,189	1
JPT1	0,000138631	0,296007967	0,273	0,128	1
COX6A1	0,00016815	0,285411315	0,614	0,364	1
HSPA8	0,000170669	0,277812654	0,795	0,586	1
ASAP1	0,000188998	0,289988194	0,375	0,202	1
RPN2	0,000215256	0,256909707	0,534	0,325	1
ARHGAP18	0,000219608	0,254893523	0,261	0,124	1
SLAMF7	0,000235075	0,30411856	0,295	0,142	1
MYDGF	0,000291843	0,276332761	0,261	0,125	1
CEBPB	0,000300092	0,261380565	0,273	0,134	1
ATP5F1B	0,000408726	0,259771528	0,591	0,378	1
PDIA3	0,000480857	0,261680782	0,375	0,208	1
TMED9	0,000635893	0,258602382	0,295	0,156	1
ARF6	0,000651541	0,274947419	0,489	0,313	1
WAS	0,00074803	0,307473524	0,5	0,301	1
RAP2B	0,000810482	0,389158004	0,318	0,181	1
CHMP4A	0,000841732	0,293429419	0,284	0,148	1
CSTB	0,000842846	0,301373144	0,523	0,316	1
CMTM3	0,00099781	0,363651247	0,341	0,192	1
SPTLC2	0,001162114	0,299437956	0,364	0,206	1
DDX3X	0,001299527	0,432551409	0,511	0,335	1
TUBB2A	0,001801225	0,370647946	0,625	0,402	1
RIPOR1	0,002321981	0,32902883	0,261	0,14	1
GABARAPL2	0,002350817	0,290832905	0,33	0,193	1

Cluster 13 marker genes

gene	p_val	avg_logFC	pct.1	pct.2	p_val_adj
HPGDS	7,25E-101	1,9890923	0,686	0,028	1,37E-96
SLC18A2	1,51E-98	2,492244413	0,629	0,024	2,86E-94
HDC	3,51E-95	2,659686868	0,629	0,025	6,65E-91
RAB44	1,34E-86	0,811438535	0,314	0,005	2,53E-82
P2RX1	2,22E-82	1,865222667	0,571	0,024	4,21E-78
SLC45A3	2,81E-80	1,521533567	0,514	0,019	5,33E-76
CADPS	1,85E-69	1,189719292	0,429	0,015	3,50E-65
LIF	3,07E-66	2,40629435	0,429	0,016	5,81E-62
RGS13	1,25E-64	1,253156893	0,371	0,012	2,37E-60
CTSG	2,03E-64	3,483900305	0,457	0,02	3,84E-60
PRG2	1,31E-62	1,266105147	0,286	0,007	2,47E-58
CDK15	4,28E-62	0,92213678	0,343	0,01	8,10E-58
KIT	8,54E-55	2,798279639	0,6	0,043	1,62E-50
SLC24A3	4,91E-52	1,027054671	0,343	0,013	9,30E-48
TPSAB1	4,32E-50	4,097764212	0,686	0,066	8,19E-46
HPGD	8,47E-49	2,532100875	0,486	0,031	1,60E-44
CPA3	9,62E-49	3,468895379	0,629	0,056	1,82E-44
MAOB	9,81E-49	1,835068521	0,486	0,031	1,86E-44
GATA2	3,60E-48	2,187435382	0,6	0,05	6,82E-44
ENPP3	3,35E-47	0,970574116	0,286	0,01	6,35E-43
VWA5A	1,43E-43	2,465513553	0,629	0,063	2,71E-39
ADCYAP1	2,16E-41	2,78896202	0,457	0,033	4,09E-37
HS6ST1	9,69E-40	1,39246825	0,486	0,038	1,84E-35
CMA1	2,01E-38	1,577473933	0,257	0,01	3,81E-34
CD22	1,24E-37	1,137227752	0,429	0,03	2,34E-33
SIGLEC17P	4,49E-37	1,749667267	0,543	0,051	8,51E-33
RHEX	1,00E-36	1,81750594	0,686	0,087	1,90E-32
COL13A1	1,55E-35	0,665943777	0,286	0,014	2,94E-31
PTGS1	1,16E-32	1,518230522	0,6	0,072	2,20E-28
MLPH	1,13E-31	1,258248658	0,571	0,064	2,13E-27
RAB27B	5,07E-31	1,319908895	0,4	0,033	9,60E-27
MS4A1	9,72E-31	2,542733135	0,714	0,114	1,84E-26
PIK3R6	1,98E-27	0,703527426	0,286	0,018	3,75E-23
IL1RL1	7,79E-26	1,051936228	0,543	0,069	1,48E-21
BTK	7,44E-25	0,962247753	0,457	0,053	1,41E-20
IFITM10	4,14E-23	0,966091789	0,314	0,027	7,85E-19
PTGS2	6,91E-22	1,88351224	0,543	0,085	1,31E-17
BACE2	3,64E-21	1,231598785	0,514	0,081	6,90E-17
SMYD3	1,43E-20	0,648628445	0,257	0,02	2,71E-16
CSF2RB	2,30E-20	1,165652976	0,543	0,09	4,35E-16
ALDH1A1	1,83E-19	1,108857761	0,429	0,06	3,47E-15
FCER1A	2,20E-19	0,460096436	0,314	0,031	4,16E-15
CSRP3	5,21E-17	1,615965808	0,686	0,188	9,87E-13
BMP2K	1,30E-16	0,907815418	0,343	0,045	2,46E-12
MEIS2	1,37E-16	0,676227282	0,314	0,038	2,60E-12
EPB41L1	1,86E-16	0,982405048	0,343	0,046	3,53E-12
TMEM176B	2,10E-16	1,766690118	0,6	0,136	3,98E-12
ATP6V0A2	3,45E-16	1,052765104	0,486	0,09	6,54E-12
RAB37	4,91E-16	0,941411966	0,429	0,068	9,31E-12
ACSL4	6,93E-16	1,224288515	0,543	0,115	1,31E-11
IL18R1	9,04E-16	0,925226973	0,343	0,047	1,71E-11
LTC4S	9,07E-16	1,139596792	0,429	0,075	1,72E-11
TMEM154	1,61E-15	0,584509493	0,314	0,039	3,05E-11

EGR3	2,60E-15	1,309993816	0,371	0,058	4,92E-11
LPCAT2	3,52E-15	1,152067379	0,514	0,103	6,66E-11
TIMP3	4,97E-15	0,985225022	0,486	0,091	9,42E-11
NRIP3	6,44E-15	0,787027619	0,286	0,035	1,22E-10
PAQR5	2,61E-14	0,49309103	0,486	0,089	4,94E-10
RGS1	1,09E-13	1,471157179	0,486	0,098	2,06E-09
ALOX5	1,12E-13	1,291989713	0,486	0,109	2,11E-09
PBX1	1,14E-13	1,074225424	0,457	0,094	2,16E-09
LAT2	1,27E-13	0,826679161	0,429	0,081	2,40E-09
SCIN	1,91E-13	0,601544057	0,286	0,038	3,61E-09
MITF	5,75E-13	0,782798872	0,371	0,064	1,09E-08
IL18	9,18E-13	0,864396291	0,457	0,096	1,74E-08
ZNRF1	1,10E-12	0,699075923	0,4	0,074	2,08E-08
CTNNBL1	1,46E-12	0,788764603	0,4	0,078	2,76E-08
EXTL3	1,99E-12	0,600864359	0,314	0,049	3,77E-08
CAVIN2	4,10E-12	1,01509811	0,371	0,071	7,76E-08
AKAP12	4,87E-12	0,781211995	0,343	0,059	9,22E-08
AGAP1	1,72E-11	0,740278939	0,4	0,081	3,27E-07
RASGRP4	3,93E-11	0,435809398	0,286	0,045	7,45E-07
GCSAML	1,18E-10	0,434361197	0,286	0,046	2,23E-06
SIGLEC8	1,20E-10	0,609257832	0,343	0,065	2,28E-06
FADS1	1,62E-10	0,893054063	0,543	0,146	3,07E-06
FAM129B	1,77E-10	0,86079564	0,457	0,113	3,35E-06
SLC44A1	1,80E-10	0,945059103	0,486	0,132	3,41E-06
SOX13	2,28E-10	0,644680736	0,257	0,04	4,32E-06
CD82	3,57E-10	1,02431403	0,543	0,168	6,77E-06
ELL2	8,44E-10	0,943073326	0,4	0,096	1,60E-05
NDST2	9,29E-10	0,642351123	0,286	0,051	1,76E-05
CXCL16	1,01E-09	0,83520381	0,629	0,203	1,91E-05
ITPRID2	1,05E-09	0,666777466	0,429	0,104	1,99E-05
GM2A	1,23E-09	0,625121975	0,486	0,128	2,33E-05
SOX4	1,85E-09	1,057691477	0,457	0,126	3,50E-05
EFCAB14	1,99E-09	0,903539526	0,657	0,241	3,77E-05
TRIO	2,09E-09	0,563491242	0,257	0,044	3,97E-05
MTHFD1	2,57E-09	0,504028656	0,314	0,062	4,87E-05
MSRA	2,78E-09	0,543654161	0,286	0,054	5,27E-05
GALC	2,98E-09	0,920989815	0,429	0,115	5,64E-05
LAX1	3,18E-09	0,842717275	0,457	0,121	6,03E-05
CSF1	3,27E-09	1,313605279	0,314	0,067	6,20E-05
CTSD	3,81E-09	1,051075028	0,714	0,383	7,22E-05
CLU	5,83E-09	0,537993275	0,714	0,265	0,000110569
TESPA1	8,18E-09	0,636156334	0,543	0,163	0,000154931
SLC2A6	8,41E-09	0,676464038	0,314	0,066	0,000159418
PAK1	1,72E-08	0,784167194	0,286	0,059	0,000326531
RIN3	1,78E-08	0,721144468	0,371	0,091	0,000336452
RGS2	1,81E-08	0,929581512	0,429	0,117	0,00034253
GAPT	2,01E-08	0,321314831	0,343	0,075	0,000381593
STX3	3,11E-08	0,816206295	0,4	0,107	0,000589113
ACER3	3,16E-08	0,452810006	0,286	0,059	0,00059913
ARHGEF6	4,40E-08	0,848887432	0,6	0,219	0,000833472
SAMSN1	4,87E-08	1,090792118	0,486	0,169	0,000923756
ALS2	4,91E-08	0,46466997	0,286	0,06	0,000930733
CD9	6,69E-08	0,739633145	0,6	0,213	0,001267728
RHOBTB3	7,02E-08	0,748755128	0,514	0,166	0,001329947
UNC5B	7,13E-08	0,704352214	0,286	0,062	0,001351171
STMN1	7,53E-08	0,708536151	0,429	0,123	0,001426466

BTN2A2	8,32E-08	0,376349064	0,286	0,06	0,001575929
TENT5A	8,54E-08	0,925055758	0,371	0,106	0,001618946
SQSTM1	8,96E-08	0,942098994	0,657	0,301	0,001697086
ZNF618	9,99E-08	0,446651155	0,314	0,072	0,001893066
TDRD3	1,06E-07	1,097779436	0,314	0,078	0,002004563
MYO10	1,23E-07	0,926907327	0,4	0,114	0,002338432
SCCPDH	1,65E-07	0,364076413	0,257	0,053	0,003120153
SLC11A2	1,84E-07	0,647517561	0,429	0,127	0,003483047
EGR1	1,96E-07	1,013609998	0,657	0,264	0,003718016
LMNA	2,14E-07	1,524563363	0,657	0,298	0,004051591
GBE1	2,28E-07	0,615535965	0,286	0,067	0,004322374
SLC43A3	2,58E-07	0,760460394	0,371	0,107	0,004884056
MAST4	2,95E-07	0,798312445	0,371	0,105	0,005585585
CD63	3,89E-07	1,01257705	0,714	0,414	0,007379294
sep-02	4,09E-07	0,6667198	0,714	0,37	0,007749619
VPS37B	4,40E-07	0,687719076	0,286	0,067	0,008347455
CHPT1	4,86E-07	0,35277631	0,286	0,066	0,009218805
TFRC	4,87E-07	0,670881245	0,371	0,105	0,009231734
FOSB	6,31E-07	1,536753463	0,771	0,493	0,011958512
PLCG2	8,13E-07	0,495137668	0,257	0,057	0,015401709
TNIK	8,21E-07	0,646130955	0,371	0,107	0,015564723
HSD17B12	8,87E-07	0,722268049	0,457	0,158	0,016815855
CHD7	9,64E-07	0,679033686	0,343	0,094	0,018275474
SLC33A1	1,20E-06	0,581478939	0,257	0,059	0,022647681
PIK3C2B	1,39E-06	0,406029745	0,286	0,069	0,026352747
LAPTM4A	1,40E-06	1,025337282	0,571	0,263	0,026549333
PPP1R15A	1,41E-06	1,055510396	0,429	0,159	0,026667438
CAPG	2,10E-06	0,839960171	0,457	0,164	0,039705383
PRKCA	2,40E-06	0,534994033	0,286	0,072	0,045558324
SLC18A1	2,41E-06	0,622142428	0,457	0,16	0,045736456
MLLT1	2,48E-06	0,674446554	0,371	0,115	0,04705863
TNFSF10	2,76E-06	0,633056678	0,486	0,176	0,052385741
PTAFR	3,42E-06	0,588094072	0,486	0,176	0,064796611
IRS2	3,74E-06	0,732679779	0,343	0,103	0,070818353
PRKX	3,76E-06	0,644347054	0,543	0,22	0,071252287
STXBP5	3,83E-06	0,500736179	0,343	0,101	0,072559416
PLIN2	3,98E-06	0,503812708	0,343	0,101	0,075413594
ASAH1	4,76E-06	0,671734074	0,543	0,221	0,090287747
TMEM176A	7,25E-06	0,622134009	0,314	0,09	0,137376953
ABCC1	7,60E-06	0,551357707	0,314	0,092	0,144072452
STAB1	8,18E-06	0,329810646	0,314	0,09	0,155024783
ARHGAP18	8,29E-06	0,715475406	0,371	0,125	0,157150562
KLRG1	8,88E-06	0,500761788	0,486	0,173	0,16828607
BTG2	9,07E-06	0,75405843	0,371	0,125	0,171883082
KIF13B	1,01E-05	0,329875028	0,314	0,09	0,19109421
RNASEH2B	1,22E-05	0,316931344	0,371	0,115	0,231883538
ORC3	1,56E-05	0,654182483	0,486	0,193	0,295117603
C4orf48	2,37E-05	0,353501595	0,286	0,082	0,448901271
CLCN3	2,45E-05	0,561558231	0,343	0,113	0,464023266
SERINC3	2,47E-05	0,464886673	0,514	0,208	0,467406586
LOC100287792	2,52E-05	0,399888395	0,257	0,069	0,477385113
FHOD1	2,60E-05	0,742595298	0,257	0,073	0,493349815
TMEM256	2,65E-05	0,503942597	0,343	0,112	0,502314311
CCDC88A	2,84E-05	0,337692769	0,543	0,217	0,537367868
SLC50A1	3,80E-05	0,377415513	0,371	0,125	0,719694591
NR4A1	4,02E-05	0,883540002	0,343	0,123	0,761976722

TUBA1B	4,13E-05	0,561543639	0,743	0,413	0,782656175
GGA2	4,19E-05	0,409267031	0,429	0,157	0,793189968
BHLHE40	4,19E-05	1,284892427	0,343	0,125	0,79335964
MBOAT7	4,43E-05	0,743859205	0,314	0,108	0,838862192
LIMD1	5,29E-05	0,33607187	0,286	0,085	1
ATN1	5,49E-05	0,472718163	0,429	0,164	1
LMLN	5,55E-05	0,63134332	0,371	0,131	1
KIAA1549	5,99E-05	0,442729768	0,343	0,116	1
DYNLL1	6,15E-05	0,491147397	0,686	0,351	1
SPATS2L	6,16E-05	0,278397133	0,371	0,128	1
SNX6	6,58E-05	0,701494009	0,429	0,184	1
MCTP2	8,44E-05	0,337752988	0,457	0,173	1
DNAAF4	8,73E-05	0,41072278	0,286	0,088	1
ARMCX3	9,27E-05	0,293472353	0,286	0,089	1
ABCC9	9,49E-05	0,557229157	0,314	0,106	1
IRAK4	0,000117408	0,282386082	0,429	0,161	1
ANXA1	0,000123247	0,83433052	0,714	0,503	1
KLF7	0,000126753	0,494633362	0,286	0,095	1
PLAUR	0,000131399	0,962328529	0,4	0,159	1
LRCH4	0,000131488	0,490406673	0,286	0,094	1
PPP3CA	0,000139938	0,441067835	0,514	0,235	1
NSMCE1	0,000143205	0,510202733	0,371	0,144	1
SDC3	0,000147989	0,423190253	0,314	0,109	1
ZNF737	0,000153222	0,361811922	0,514	0,21	1
IFIT1	0,000168517	0,350281401	0,286	0,093	1
CIZ1	0,000176062	0,610929073	0,257	0,084	1
CWF19L1	0,000195788	0,305455012	0,514	0,213	1
GFOD2	0,000213867	0,421499828	0,429	0,175	1
DUSP6	0,000221878	1,232168113	0,371	0,159	1
MYADM	0,000232899	0,865958925	0,629	0,387	1
USP53	0,000243752	0,398347957	0,343	0,127	1
SLC26A2	0,000244637	0,325206857	0,371	0,141	1
RBBP6	0,000258724	0,2829791	0,486	0,202	1
PWARSN	0,000272	0,281425955	0,371	0,137	1
KCNE3	0,000273136	0,482743729	0,257	0,084	1
LOC100289495	0,000273241	0,475100204	0,343	0,127	1
ENC1	0,000275963	0,320621814	0,257	0,081	1
PDE4A	0,000284261	0,411967581	0,286	0,099	1
JRK	0,000286193	0,329039971	0,314	0,111	1
DDAH2	0,000287119	0,682225251	0,314	0,123	1
RRBP1	0,000301627	0,3669476	0,343	0,129	1
SERPINB1	0,00030196	0,785712833	0,486	0,241	1
CD69	0,000336554	0,689021921	0,4	0,17	1
RHOG	0,000337497	0,461392957	0,486	0,219	1
CYTH2	0,000349278	0,37183499	0,286	0,099	1
FANCD2	0,000351551	0,27378671	0,257	0,08	1
EP300	0,000354348	0,38419125	0,486	0,22	1
UNC13D	0,000389051	0,575758236	0,371	0,155	1
NR4A2	0,000412843	0,678086388	0,457	0,211	1
SELENOK	0,000438774	0,372119892	0,343	0,132	1
VIM	0,000449692	0,674212894	0,886	0,829	1
HNF1A-AS1	0,000459645	0,300287677	0,286	0,098	1
FOS	0,000477396	0,698248931	0,686	0,383	1
C12orf77	0,000499552	0,331576591	0,257	0,085	1
MAPK6	0,00054423	0,381183782	0,257	0,087	1
CLASP1	0,00057866	0,259057014	0,257	0,086	1

MGAT4B	0,000609229	0,435418534	0,257	0,09	1
AHR	0,000657638	0,639347487	0,4	0,189	1
ANKRD28	0,00067643	0,512178045	0,257	0,091	1
GLUL	0,000682607	0,91154796	0,514	0,292	1
ANP32A-IT1	0,000684204	0,27086551	0,343	0,132	1
LINC-ROR	0,000688124	0,347916807	0,314	0,116	1
CRYBB2P1	0,000698051	0,441050659	0,4	0,169	1
TOX4	0,000701277	0,327983549	0,286	0,103	1
VAV1	0,000730301	0,259225445	0,286	0,102	1
NFKBIA	0,000769028	0,714430737	0,571	0,325	1
CPM	0,000780412	0,966306021	0,429	0,215	1
FLAD1	0,000786643	0,316916436	0,257	0,087	1
MTAP	0,000797461	0,361968664	0,314	0,122	1
PRPF3	0,000800825	0,718622833	0,314	0,132	1
RRP7A	0,000816096	0,361953638	0,514	0,244	1
RAC2	0,000908665	0,491797413	0,771	0,537	1
OBSL1	0,000923137	0,262320883	0,257	0,088	1
GANAB	0,000936728	0,441237052	0,4	0,177	1
VPS4B	0,000948972	0,51442133	0,429	0,197	1
CASP10	0,000969307	0,445798564	0,429	0,2	1
DEGS1	0,001150832	0,430105335	0,429	0,194	1
LINC00842	0,001171751	0,510082592	0,286	0,111	1
RNF130	0,001245803	0,510316664	0,371	0,164	1
ZNF33A	0,00126887	0,357617711	0,371	0,156	1
PGPEP1	0,001319904	0,411612021	0,514	0,264	1
DNAJC3	0,001340818	0,465199305	0,4	0,182	1
NPHP3	0,001380207	0,307992965	0,314	0,126	1
DDX27	0,001420724	0,310201831	0,286	0,11	1
FTH1	0,001456528	0,489016206	0,914	0,814	1
SEC22B	0,001477121	0,482434371	0,257	0,098	1
SSBP3	0,001507609	0,338087914	0,343	0,142	1
ACBD7	0,001614651	0,460443023	0,4	0,186	1
SPG7	0,001621621	0,318846282	0,343	0,144	1
SLC16A3	0,001668841	0,470130542	0,257	0,098	1
ARFGEF2	0,001762864	0,457767695	0,257	0,097	1
ALOX5AP	0,001912992	0,500812081	0,543	0,293	1
NCBP1	0,001941392	0,254614808	0,371	0,161	1
FOXP1	0,001951801	0,443047147	0,714	0,508	1
ZNF621	0,002203517	0,457541622	0,629	0,368	1
COL18A1	0,002209813	0,303364091	0,257	0,095	1
STX16	0,00222274	0,450963975	0,514	0,27	1
TRPS1	0,002228708	0,340404615	0,257	0,099	1
ADGRE2	0,002234641	0,357484909	0,314	0,131	1
ANKRD10	0,002366008	0,289927836	0,371	0,164	1
GXYLT1	0,002657331	0,285447785	0,314	0,135	1
IFIT3	0,002707603	0,299863094	0,314	0,128	1
SDHD	0,002739866	0,319638227	0,286	0,118	1
ZMAT1	0,002844036	0,492428084	0,257	0,102	1
GOSR1	0,002867605	0,394653898	0,429	0,216	1
DNAL1	0,00289184	0,462002449	0,4	0,203	1
HEXA-AS1	0,00303813	0,466892854	0,314	0,136	1
PASD1	0,003058657	0,285547314	0,257	0,099	1
MON2	0,003109489	0,303152135	0,314	0,134	1
LINC00667	0,00323954	0,331397057	0,257	0,101	1
HSP90AB1	0,003256478	0,359530567	0,886	0,58	1
SHANK2-AS3	0,003349158	0,309637061	0,343	0,152	1

CAPRIN1	0,003436445	0,363976195	0,429	0,211	1
PRRG4	0,003586086	0,256233033	0,257	0,1	1
SLC25A1	0,003830964	0,406817404	0,257	0,107	1
TSPYL2	0,003854644	0,278181049	0,4	0,185	1
ELF1	0,00396764	0,375432799	0,371	0,178	1
GAS7	0,004025521	0,25173595	0,429	0,209	1
CD44	0,004156323	0,675148058	0,657	0,573	1
JUN	0,004243185	0,537451508	0,371	0,177	1
WSB1	0,004287991	0,461139069	0,657	0,391	1
ARHGAP25	0,004384187	0,257347097	0,314	0,136	1
VMA21	0,004386531	0,313232138	0,314	0,142	1
PRDX1	0,004474362	0,431244436	0,486	0,276	1
RHBDD2	0,004482089	0,534230431	0,286	0,128	1
AK4	0,004626182	0,399465503	0,371	0,179	1
SIGLEC10	0,004750763	0,389214273	0,4	0,198	1
LGALS3	0,00480041	0,395751284	0,457	0,244	1
UBTF	0,004832927	0,405825271	0,257	0,107	1
FAM192A	0,005020745	0,548827162	0,371	0,191	1
VCL	0,005176424	0,365212968	0,343	0,16	1
RASSF5	0,005381994	0,362126952	0,571	0,327	1
SPINT2	0,005383139	0,584593031	0,4	0,211	1
AP1S3	0,005501903	0,348923972	0,371	0,179	1
SSR1	0,005808544	0,296859417	0,657	0,387	1
NLRP6	0,006012886	0,447281542	0,486	0,277	1
MFSD11	0,006417806	0,312561012	0,257	0,109	1
COMMD9	0,006541945	0,250550548	0,286	0,124	1
ANXA4	0,006601385	0,486168934	0,257	0,117	1
P4HB	0,00663246	0,418206006	0,543	0,327	1
RBMX	0,006760443	0,290427972	0,6	0,346	1
ATP6AP1	0,007048823	0,392436944	0,343	0,162	1
CHD9	0,00707416	0,313096282	0,457	0,247	1
SENP5	0,007167466	0,396884336	0,257	0,114	1
JUNB	0,007391187	0,399957794	0,686	0,448	1
ARHGAP15	0,007397318	0,43006766	0,286	0,13	1
TMF1	0,007522439	0,351510842	0,4	0,195	1
SYNGR2	0,007550594	0,379226091	0,4	0,209	1
DAP	0,007661987	0,415932077	0,286	0,136	1
NFKBIZ	0,007752617	0,593522288	0,371	0,206	1
PLEKHB2	0,0080209	0,29008381	0,486	0,273	1
SYAP1	0,008097191	0,704940712	0,4	0,225	1
RAB7A	0,008488949	0,343329669	0,543	0,313	1
ATP5PB	0,008604726	0,360480361	0,486	0,291	1
NR1H2	0,008753814	0,305825956	0,286	0,135	1
PEBP1	0,008846575	0,426422221	0,571	0,416	1
TET2	0,008918825	0,519196902	0,4	0,217	1
ITPR1	0,008943057	0,291842296	0,257	0,114	1
ABI2	0,00900686	0,26324375	0,4	0,205	1
ZFP36	0,009528052	0,698635234	0,457	0,271	1
OSBPL8	0,009672742	0,453009516	0,457	0,288	1
ZNF271P	0,009768717	0,298669849	0,257	0,113	1

Cluster CD4.0 marker genes

gene	p_val	avg_logFC	pct.1	pct.2	p_val_adj
NKG7	8,50E-27	2,067869812	0,547	0,142	1,61E-22
GZMH	6,84E-19	1,157810554	0,328	0,044	1,30E-14
CCL4	5,50E-18	1,584027926	0,511	0,162	1,04E-13
CCL5	5,22E-15	1,013844427	0,788	0,487	9,90E-11
PRF1	3,31E-12	1,017109261	0,35	0,098	6,27E-08
GNLY	3,85E-12	1,769516524	0,526	0,265	7,29E-08
SLAMF7	5,27E-10	0,6858639	0,255	0,062	9,99E-06
CST7	7,59E-10	0,989356382	0,46	0,206	1,44E-05
CTSW	2,06E-09	0,878088329	0,489	0,245	3,91E-05
TARP	8,77E-09	0,886491301	0,35	0,131	0,000166164
GZMA	1,86E-07	0,603520217	0,635	0,41	0,003526757
ITM2C	2,43E-07	0,697679981	0,343	0,144	0,004612576
PLEK	6,01E-07	0,729967882	0,285	0,111	0,011381584
FCER1G	9,81E-07	0,954061667	0,401	0,214	0,018594482
APOBEC3G	1,27E-06	0,587779884	0,423	0,229	0,023984256
KLRD1	1,47E-06	0,809788601	0,328	0,149	0,027899328
RUNX3	2,35E-06	0,47190289	0,635	0,441	0,044495818
GZMK	3,09E-06	0,595100312	0,453	0,237	0,058645704
SLAMF6	5,74E-06	0,4807537	0,445	0,24	0,108769028
TGFBR3	6,22E-06	0,725544368	0,365	0,186	0,117943313
IL2RB	1,05E-05	0,779074225	0,496	0,309	0,199813686
TMEM212	2,32E-05	0,36916543	0,854	0,722	0,439433792
CTSD	4,62E-05	0,403864802	0,453	0,263	0,87642283
SYNE1	9,57E-05	0,460078756	0,423	0,25	1
MTHFR	0,000104001	0,401988041	0,343	0,18	1
CEACAMP1	0,000120314	0,427305417	0,321	0,162	1
POTEM	0,000129943	0,410001855	0,555	0,366	1
SRGN	0,000159585	0,364713385	0,876	0,778	1
TYROBP	0,000163807	0,587102709	0,482	0,335	1
EFHD2	0,000220206	0,387863084	0,365	0,204	1
CFLAR	0,000229385	0,347266454	0,912	0,845	1
PIP4K2A	0,000261045	0,415313099	0,511	0,351	1
HCST	0,000285201	0,313144966	0,701	0,523	1
WAS	0,000382783	0,499812689	0,474	0,317	1
DYNLL1	0,000633074	0,377156328	0,394	0,237	1
GNPTAB	0,000683955	0,382151294	0,394	0,25	1
XRCC2	0,000798217	0,326450376	0,489	0,332	1
BHMT2	0,000871068	0,358127167	0,328	0,193	1
HOPX	0,00088192	0,486943242	0,27	0,149	1
CBLB	0,000909559	0,396526661	0,27	0,144	1
IER2	0,001156505	0,424876224	0,255	0,131	1
HNRNPAB	0,001280655	0,370450798	0,285	0,16	1
LOC643406	0,001323465	0,321646465	0,839	0,727	1
ACTN4	0,001395882	0,341474546	0,62	0,487	1
LOC286437	0,001455081	0,329041857	0,54	0,371	1
HLA-DRB1	0,001490395	0,375583324	0,591	0,441	1
GNAI2	0,001510325	0,296406031	0,752	0,644	1
ANKRD20A9P	0,001706402	0,345543157	0,774	0,696	1
LOC100131257	0,001889798	0,306631332	0,971	0,954	1
APOL6	0,001942391	0,286601341	0,657	0,518	1
HLA-DPB1	0,002026417	0,349886964	0,46	0,33	1
LINC00670	0,002180581	0,284863509	0,401	0,263	1
ORC4	0,002195643	0,282918073	0,423	0,284	1

RAMP2-AS1	0,002196882	0,310757556	0,277	0,157	1
F5	0,002246055	0,33067526	0,745	0,642	1
B4GALT1	0,002335813	0,275819347	0,292	0,165	1
NUDT9	0,002427814	0,528652674	0,277	0,162	1
APMAP	0,003209434	0,314915593	0,263	0,149	1
CSRP1	0,003219622	0,354382055	0,263	0,149	1
NFATC2	0,003497	0,268859252	0,453	0,304	1
ZNRF3-AS1	0,003536353	0,372419508	0,27	0,16	1
ZNF471	0,00355809	0,279887727	0,759	0,68	1
METTL2B	0,003559253	0,29181008	0,255	0,142	1
SPCS3	0,003969883	0,254567359	0,35	0,222	1
HSH2D	0,003991	0,347797838	0,277	0,162	1
PSAP	0,004065501	0,319204279	0,533	0,405	1
PTP4A2	0,004118327	0,25979862	0,781	0,649	1
METTL21A	0,004252357	0,424504409	0,314	0,201	1
PSMD7	0,004454927	0,325394969	0,263	0,149	1
ARL5A	0,004517499	0,268102913	0,285	0,168	1
PTP4A1	0,004521165	0,428765892	0,255	0,147	1
XAF1	0,004765704	0,322202944	0,825	0,753	1
ITGB2	0,004870597	0,310611869	0,642	0,526	1
EIF5	0,004892868	0,378553749	0,372	0,245	1
HLA-DPA1	0,004909367	0,268675569	0,686	0,541	1
CNPY3	0,00504182	0,452545549	0,285	0,173	1
HAUS2	0,005163693	0,273025345	0,343	0,214	1
CCDC144B	0,005207425	0,270842167	0,774	0,644	1
SHISA9	0,005800182	0,31583755	0,35	0,227	1
RAB3B	0,00699235	0,34195615	0,336	0,216	1
MAB21L3	0,007240196	0,286243653	0,891	0,907	1
PDIA3	0,008097543	0,262291447	0,255	0,152	1
TPTE2P1	0,008281773	0,427659162	0,453	0,358	1

Cluster CD4.1 marker genes

gene	p_val	avg_logFC	pct.1	pct.2	p_val_adj
GZMK	1,68E-10	0,874169004	0,528	0,233	3,18E-06
GZMA	6,59E-07	0,563358718	0,667	0,417	0,012494149
CCL5	0,00036219	0,313631772	0,694	0,532	1
KLF5	0,000386669	0,417816287	0,463	0,302	1
AHNAK	0,001854686	0,368529822	0,824	0,779	1
SNTB2	0,001936896	0,624303981	0,352	0,23	1
MYADM	0,004767254	0,4192883	0,454	0,341	1
BTG1	0,005961982	0,309839052	0,759	0,695	1
ATP2A3	0,00700046	0,415403727	0,315	0,209	1

Cluster CD4.2 marker genes

gene	p_val	avg_logFC	pct.1	pct.2	p_val_adj
SEC23IP	0,000461133	0,567810838	0,267	0,137	1
ZNF480	0,001867355	0,358341787	0,693	0,618	1
IL7R	0,003629203	0,348325013	0,723	0,585	1
CTSB	0,005467946	0,261524059	0,594	0,491	1
SARAF	0,009314131	0,297392716	0,802	0,752	1

Cluster CD4.3 marker genes

gene	p_val	avg_logFC	pct.1	pct.2	p_val_adj
FOXP3	3,27E-23	1,731189164	0,319	0,023	6,20E-19
IL2RA	2,80E-17	1,513412323	0,287	0,032	5,30E-13
MAL	1,08E-15	0,857770399	0,511	0,135	2,05E-11
LTB	6,74E-14	0,845332256	0,809	0,45	1,28E-09
RTKN2	4,60E-10	1,049618839	0,298	0,077	8,71E-06
TTN	1,79E-09	0,788980369	0,436	0,158	3,39E-05
ICOS	4,12E-09	0,57217179	0,277	0,067	7,80E-05
BIRC3	6,15E-09	0,759725507	0,574	0,283	0,000116589
SELL	7,11E-08	0,832872244	0,543	0,271	0,001347293
RACK1	1,64E-07	0,386252226	0,968	0,898	0,003104168
TIGIT	1,02E-06	0,803667888	0,309	0,116	0,019388973
TBC1D4	1,85E-06	0,859626966	0,33	0,135	0,035077073
POU2F2	4,06E-06	0,442760857	0,436	0,195	0,07700357
DGKA	1,00E-05	0,528825642	0,681	0,432	0,189854654
PTEN	1,71E-05	0,507083695	0,394	0,193	0,323585755
AK2	1,94E-05	0,476019846	0,309	0,123	0,367911796
LDHB	2,18E-05	0,415536836	0,745	0,503	0,41350675
TRIM22	2,70E-05	0,54878196	0,521	0,299	0,511363373
LEF1	6,03E-05	0,441394157	0,426	0,218	1
RSRP1	6,29E-05	0,430593989	0,287	0,125	1
GATA3	9,54E-05	0,479172878	0,372	0,186	1
ETS1	0,000103478	0,413849031	0,819	0,698	1
CYLD	0,000106939	0,396106647	0,66	0,443	1
EEF2	0,000127647	0,313464802	0,904	0,694	1
WDR26	0,00017171	0,448711882	0,404	0,216	1
SERBP1	0,000182445	0,411789618	0,532	0,336	1
FCMR	0,000190352	0,351802383	0,574	0,348	1
C1orf162	0,0003341	0,655841901	0,319	0,167	1
IL6ST	0,000438144	0,306857319	0,404	0,223	1
NDUFV2	0,000455668	0,382198852	0,34	0,179	1
ADD1	0,000498011	0,478396303	0,394	0,223	1
MARF1	0,000500769	0,339320874	0,298	0,142	1
IL6R	0,000624939	0,40055309	0,372	0,218	1
NCOA3	0,000770888	0,416339269	0,34	0,188	1
PABPC1	0,000835413	0,253058143	0,968	0,914	1
EIF3E	0,000870485	0,354333319	0,702	0,508	1
RASGRP1	0,000927071	0,307172749	0,489	0,297	1
NLRP1	0,000975352	0,253877182	0,404	0,223	1
SOD1	0,001062795	0,337450255	0,606	0,427	1
PIM2	0,00116025	0,4924257	0,266	0,135	1
DIPK2B	0,001194855	0,340400155	0,319	0,169	1
ITK	0,001307413	0,324707526	0,468	0,29	1
DYNLRB1	0,001389377	0,441404302	0,426	0,253	1
FAM208A	0,001426083	0,316511551	0,351	0,2	1
UQCRB	0,001559763	0,416344623	0,511	0,362	1
FAU	0,001738243	0,258747251	0,979	0,916	1
SESN3	0,001900735	0,404252293	0,266	0,139	1
CRYBG1	0,001969549	0,291335194	0,372	0,218	1
POLR2B	0,001981938	0,367332716	0,309	0,169	1
NOP53	0,002053504	0,31920717	0,809	0,717	1
HSPA8	0,002082227	0,309235893	0,819	0,691	1
ATP6V1F	0,002171197	0,315752502	0,351	0,206	1
TRAF3IP3	0,002501636	0,355158366	0,372	0,22	1

NPM1	0,002775739	0,283463492	0,734	0,596	1
TCF7	0,002848564	0,370083907	0,606	0,441	1
STAT3	0,002921611	0,444818909	0,5	0,355	1
GNB5	0,003454001	0,367237925	0,33	0,195	1
ZFAS1	0,003467071	0,384920632	0,564	0,415	1
CCR7	0,00375101	0,507365565	0,277	0,158	1
RAB11FIP1	0,003847167	0,336348657	0,383	0,234	1
SCARNA21	0,004249809	0,406821496	0,255	0,139	1
HIPK2	0,004438531	0,268054055	0,309	0,169	1
SUB1	0,004514005	0,333575375	0,585	0,427	1
EEF1B2	0,004630678	0,255156842	0,904	0,812	1
PPP1CB	0,004633331	0,263413216	0,543	0,376	1
TAB2	0,005139281	0,315607653	0,34	0,204	1
CYTIP	0,005171853	0,281515711	0,479	0,311	1
GOLGA8A	0,005460884	0,278549281	0,521	0,374	1
MAP3K1	0,005658079	0,282588817	0,33	0,193	1
AES	0,005830307	0,276220618	0,809	0,687	1
LRRC8C	0,006137769	0,339917037	0,266	0,155	1
TRIR	0,006265176	0,268401009	0,649	0,462	1
CEP85L	0,006277604	0,338594547	0,34	0,211	1
STAT5B	0,006757949	0,407029707	0,34	0,213	1
PCED1B-AS1	0,006800788	0,340004708	0,298	0,179	1
FAM227A	0,006847641	0,28629549	0,383	0,244	1
PPP1CC	0,006854426	0,274640527	0,415	0,267	1
NCL	0,007249052	0,265714433	0,447	0,295	1
TXLNGY	0,007660504	0,359523581	0,266	0,153	1
TXNIP	0,007726794	0,27187474	0,66	0,476	1
RASA3	0,008316902	0,287652694	0,277	0,162	1
SNRPB2	0,008415541	0,278362613	0,33	0,197	1
ETNK1	0,008691119	0,347247414	0,255	0,144	1
SORL1	0,00870033	0,333740597	0,628	0,503	1
FAM49B	0,009710908	0,340542754	0,372	0,251	1
SNHG5	0,009833826	0,286805688	0,585	0,452	1
CBX7	0,009949694	0,290826048	0,298	0,176	1

Cluster CD4.4 marker genes

gene	p_val	avg_logFC	pct.1	pct.2	p_val_adj
EEF1A1	8,19E-08	0,318243745	1	0,995	0,001552182
TPT1	8,61E-08	0,440740618	0,988	0,98	0,001631115
PABPC1	1,19E-07	0,419796388	0,953	0,918	0,002251514
LTB	3,85E-06	0,547854503	0,741	0,47	0,072900315
PASK	5,11E-06	0,488471774	0,282	0,098	0,096758403
LEF1	7,98E-06	0,586498371	0,447	0,218	0,151143523
EEF1G	1,61E-05	0,36509439	0,894	0,784	0,304942614
GOLGB1	1,70E-05	0,465929118	0,412	0,193	0,321720719
EIF3L	1,91E-05	0,430913711	0,694	0,434	0,361563181
CCR7	4,59E-05	0,575441548	0,329	0,15	0,870710087
NPM3	7,92E-05	0,426405175	0,694	0,507	1
ZC3HAV1	9,58E-05	0,365013585	0,718	0,548	1
HADHB	0,000107168	0,354554841	0,294	0,125	1
TCF7	0,000119888	0,465987401	0,647	0,436	1
ABLIM1	0,000130147	0,526792018	0,459	0,255	1
ANXA1	0,000149946	0,387494533	0,729	0,548	1
C6orf48	0,000160744	0,479463696	0,506	0,286	1
FAM117A	0,00016161	0,371660564	0,271	0,114	1
MAP3K1	0,000228256	0,453085201	0,365	0,189	1
TMSB10	0,000292734	0,25617146	0,976	0,93	1
PIM1	0,000387152	0,563735956	0,306	0,145	1
TMEM248	0,000412928	0,487028579	0,388	0,218	1
LDHB	0,000440787	0,300835753	0,729	0,511	1
GSTK1	0,000453175	0,335041611	0,682	0,466	1
LAMTOR5	0,00059464	0,371664561	0,271	0,123	1
CDC14A	0,000629743	0,301888833	0,424	0,23	1
NACA	0,000649958	0,269820165	0,824	0,62	1
HPCAL1	0,000768522	0,405607349	0,353	0,189	1
ZFAS1	0,001136064	0,425755644	0,6	0,411	1
VPS13A	0,001164961	0,354485603	0,282	0,136	1
RBMS1	0,001178787	0,333636829	0,624	0,436	1
ATP5PD	0,001188238	0,330444041	0,471	0,289	1
SLC2A3	0,001236309	0,44109306	0,576	0,389	1
ADAM19	0,00127448	0,378577631	0,259	0,12	1
TNFRSF25	0,001311538	0,357011937	0,365	0,2	1
DGKA	0,001357742	0,264022186	0,635	0,445	1
PSMD11	0,001493709	0,299027687	0,341	0,182	1
UBR5	0,001539718	0,434188582	0,318	0,17	1
CD6	0,001616673	0,388987177	0,459	0,293	1
UBA52	0,001707953	0,256864694	0,988	0,939	1
KMT2A	0,001916783	0,447418336	0,588	0,439	1
EEF1B2	0,001969559	0,288511819	0,882	0,818	1
PGGHG	0,002015553	0,327476809	0,341	0,186	1
BIN1	0,002395887	0,401937331	0,376	0,223	1
FKBP11	0,002496491	0,290820046	0,282	0,143	1
BCL2	0,002668812	0,476551605	0,4	0,252	1
CLSTN1	0,002835632	0,391170571	0,306	0,173	1
IL7R	0,002935249	0,369915314	0,753	0,584	1
PSMB4	0,003010421	0,250810237	0,541	0,343	1
GPR171	0,003323367	0,347128143	0,365	0,214	1
ARID5B	0,003623018	0,48996377	0,306	0,173	1
VAMP4	0,003786021	0,339348998	0,259	0,13	1
GIMAP4	0,003909209	0,383457509	0,424	0,261	1

ZC3H11A	0,004069956	0,464855732	0,376	0,22	1
HK1	0,004282945	0,494724869	0,282	0,159	1
HSPA8	0,004817895	0,296802696	0,8	0,698	1
TMC8	0,004949104	0,459673535	0,518	0,361	1
PLEC	0,005070679	0,381860235	0,482	0,314	1
RBBP6	0,006065935	0,292453393	0,341	0,2	1
SYNE2	0,006317753	0,345004896	0,682	0,575	1
ANP32B	0,006342149	0,28392217	0,612	0,432	1
sep-06	0,006565577	0,302381275	0,635	0,466	1
ISG20	0,00697092	0,323741692	0,365	0,232	1
RNF10	0,007556286	0,410044018	0,282	0,164	1
MAL	0,00801196	0,353314123	0,306	0,182	1
COX7A2	0,008095886	0,451501852	0,518	0,38	1
EIF3E	0,008314454	0,252140339	0,694	0,514	1
MBTPS1	0,008406392	0,45204668	0,259	0,143	1
GNG5	0,00869045	0,263120426	0,353	0,211	1
STAT6	0,009603802	0,305325386	0,412	0,268	1

Cluster CD8.0 marker genes

gene	p_val	avg_logFC	pct.1	pct.2	p_val_adj
LEF1	7,60E-12	1,375751694	0,447	0,101	1,44E-07
LTB	4,03E-11	1,251135262	0,596	0,204	7,63E-07
SELL	1,69E-07	1,351962132	0,447	0,166	0,003208283
IL7R	1,88E-07	0,810075448	0,702	0,384	0,003560116
PDE3B	7,07E-07	0,951944601	0,34	0,105	0,013390527
EEF1A1	1,31E-06	0,410289381	1	0,994	0,024828104
EEF1G	3,07E-06	0,677376806	0,915	0,756	0,05822641
TRADD	2,77E-05	0,562095805	0,298	0,093	0,52479395
TPT1	2,90E-05	0,532892808	1	0,976	0,549171852
FAU	3,20E-05	0,430177582	1	0,893	0,607314654
FLT3LG	6,60E-05	0,858839629	0,426	0,202	1
PABPC1	0,000132876	0,454894464	0,936	0,871	1
BIRC3	0,000150303	0,681323031	0,362	0,152	1
TMSB10	0,000212107	0,31675806	0,979	0,935	1
NPM3	0,000229022	0,726411686	0,553	0,331	1
TCF7	0,000231159	0,872357212	0,511	0,315	1
LDLRAP1	0,000240862	0,681687883	0,319	0,129	1
ARIH1	0,000319867	0,438769793	0,319	0,123	1
FXYD5	0,000453925	0,60313978	0,723	0,527	1
LOC100506023	0,000454006	0,562546913	0,404	0,204	1
UBA52	0,000498022	0,346309124	0,957	0,903	1
IGBP1	0,000621901	0,618432349	0,34	0,154	1
CDC14A	0,000684542	0,550815254	0,362	0,168	1
NAP1L1	0,000890162	0,419063314	0,809	0,644	1
RACK1	0,0008962	0,419648887	0,936	0,846	1
RFC2	0,00090555	0,569273835	0,255	0,101	1
NOP53	0,001061279	0,534638286	0,745	0,618	1
ATP8B2	0,001473056	0,569603586	0,298	0,137	1
TAB2	0,001562616	0,851335925	0,319	0,152	1
LRRC75A-AS1	0,001768166	0,682889984	0,468	0,261	1
SFSWAP	0,002070957	0,47182729	0,255	0,109	1
TESPA1	0,002071223	0,466796286	0,319	0,154	1
EIF3E	0,00223554	0,531223641	0,596	0,398	1
ARHGAP1	0,002719302	0,473246772	0,319	0,152	1
PGK1	0,002786078	0,472666067	0,638	0,44	1
EIF3M	0,002925379	0,492855945	0,34	0,17	1
MAP3K1	0,003220728	0,534832132	0,277	0,129	1
EEF1D	0,003749926	0,441131975	0,723	0,594	1
NUCB1	0,003942196	0,592805207	0,34	0,178	1
ZNHIT1	0,004123301	0,616989041	0,277	0,137	1
LINC00346	0,004385577	0,548842642	0,34	0,186	1
EEF1B2	0,004788787	0,58511905	0,766	0,699	1
NSD3	0,005027681	0,607964085	0,362	0,204	1
UNC45A	0,005082218	0,400079521	0,277	0,127	1
DDB1	0,005176527	0,380166698	0,298	0,143	1
PIK3IP1	0,005535214	0,34194524	0,383	0,202	1
SPTBN1	0,005595569	0,39761346	0,319	0,164	1
SNHG8	0,00591067	0,787585549	0,319	0,18	1
RTN3	0,006504888	0,472808865	0,255	0,119	1
GDI1	0,007044316	0,37271829	0,404	0,226	1
FAM106A	0,007619263	0,404029537	0,255	0,121	1
HNRNPF	0,007805072	0,375565244	0,426	0,244	1
COX7C	0,00815907	0,334505971	0,809	0,697	1

CS	0,008554912	0,576493234	0,298	0,145	1
TTC39C	0,00865607	0,373612124	0,34	0,182	1
ERGIC3	0,008764165	0,51213885	0,383	0,226	1
GATA3	0,009199683	0,798290156	0,362	0,208	1

Cluster CD8.1 marker genes

gene	p_val	avg_logFC	pct.1	pct.2	p_val_adj
LEF1	7,60E-12	1,375751694	0,447	0,101	1,44E-07
LTB	4,03E-11	1,251135262	0,596	0,204	7,63E-07
SELL	1,69E-07	1,351962132	0,447	0,166	0,003208283
IL7R	1,88E-07	0,810075448	0,702	0,384	0,003560116
PDE3B	7,07E-07	0,951944601	0,34	0,105	0,013390527
EEF1A1	1,31E-06	0,410289381	1	0,994	0,024828104
EEF1G	3,07E-06	0,677376806	0,915	0,756	0,05822641
TRADD	2,77E-05	0,562095805	0,298	0,093	0,52479395
TPT1	2,90E-05	0,532892808	1	0,976	0,549171852
FAU	3,20E-05	0,430177582	1	0,893	0,607314654
FLT3LG	6,60E-05	0,858839629	0,426	0,202	1
PABPC1	0,000132876	0,454894464	0,936	0,871	1
BIRC3	0,000150303	0,681323031	0,362	0,152	1
TMSB10	0,000212107	0,31675806	0,979	0,935	1
NPM3	0,000229022	0,726411686	0,553	0,331	1
TCF7	0,000231159	0,872357212	0,511	0,315	1
LDLRAP1	0,000240862	0,681687883	0,319	0,129	1
ARIH1	0,000319867	0,438769793	0,319	0,123	1
FXYD5	0,000453925	0,60313978	0,723	0,527	1
LOC100506023	0,000454006	0,562546913	0,404	0,204	1
UBA52	0,000498022	0,346309124	0,957	0,903	1
IGBP1	0,000621901	0,618432349	0,34	0,154	1
CDC14A	0,000684542	0,550815254	0,362	0,168	1
NAP1L1	0,000890162	0,419063314	0,809	0,644	1
RACK1	0,0008962	0,419648887	0,936	0,846	1
RFC2	0,00090555	0,569273835	0,255	0,101	1
NOP53	0,001061279	0,534638286	0,745	0,618	1
ATP8B2	0,001473056	0,569603586	0,298	0,137	1
TAB2	0,001562616	0,851335925	0,319	0,152	1
LRRC75A-AS1	0,001768166	0,682889984	0,468	0,261	1
SFSWAP	0,002070957	0,47182729	0,255	0,109	1
TESPA1	0,002071223	0,466796286	0,319	0,154	1
EIF3E	0,00223554	0,531223641	0,596	0,398	1
ARHGAP1	0,002719302	0,473246772	0,319	0,152	1
PGK1	0,002786078	0,472666067	0,638	0,44	1
EIF3M	0,002925379	0,492855945	0,34	0,17	1
MAP3K1	0,003220728	0,534832132	0,277	0,129	1
EEF1D	0,003749926	0,441131975	0,723	0,594	1
NUCB1	0,003942196	0,592805207	0,34	0,178	1
ZNHIT1	0,004123301	0,616989041	0,277	0,137	1
LINC00346	0,004385577	0,548842642	0,34	0,186	1
EEF1B2	0,004788787	0,58511905	0,766	0,699	1
NSD3	0,005027681	0,607964085	0,362	0,204	1
UNC45A	0,005082218	0,400079521	0,277	0,127	1
DDB1	0,005176527	0,380166698	0,298	0,143	1
PIK3IP1	0,005535214	0,34194524	0,383	0,202	1
SPTBN1	0,005595569	0,39761346	0,319	0,164	1
SNHG8	0,00591067	0,787585549	0,319	0,18	1
RTN3	0,006504888	0,472808865	0,255	0,119	1
GDI1	0,007044316	0,37271829	0,404	0,226	1
FAM106A	0,007619263	0,404029537	0,255	0,121	1
HNRNPF	0,007805072	0,375565244	0,426	0,244	1
COX7C	0,00815907	0,334505971	0,809	0,697	1

CS	0,008554912	0,576493234	0,298	0,145	1
TTC39C	0,00865607	0,373612124	0,34	0,182	1
ERGIC3	0,008764165	0,51213885	0,383	0,226	1
GATA3	0,009199683	0,798290156	0,362	0,208	1

Cluster CD8.2 marker genes

gene	p_val	avg_logFC	pct.1	pct.2	p_val_adj
LEF1	7,60E-12	1,375751694	0,447	0,101	1,44E-07
LTB	4,03E-11	1,251135262	0,596	0,204	7,63E-07
SELL	1,69E-07	1,351962132	0,447	0,166	0,003208283
IL7R	1,88E-07	0,810075448	0,702	0,384	0,003560116
PDE3B	7,07E-07	0,951944601	0,34	0,105	0,013390527
EEF1A1	1,31E-06	0,410289381	1	0,994	0,024828104
EEF1G	3,07E-06	0,677376806	0,915	0,756	0,05822641
TRADD	2,77E-05	0,562095805	0,298	0,093	0,52479395
TPT1	2,90E-05	0,532892808	1	0,976	0,549171852
FAU	3,20E-05	0,430177582	1	0,893	0,607314654
FLT3LG	6,60E-05	0,858839629	0,426	0,202	1
PABPC1	0,000132876	0,454894464	0,936	0,871	1
BIRC3	0,000150303	0,681323031	0,362	0,152	1
TMSB10	0,000212107	0,31675806	0,979	0,935	1
NPM3	0,000229022	0,726411686	0,553	0,331	1
TCF7	0,000231159	0,872357212	0,511	0,315	1
LDLRAP1	0,000240862	0,681687883	0,319	0,129	1
ARIH1	0,000319867	0,438769793	0,319	0,123	1
FXYD5	0,000453925	0,60313978	0,723	0,527	1
LOC100506023	0,000454006	0,562546913	0,404	0,204	1
UBA52	0,000498022	0,346309124	0,957	0,903	1
IGBP1	0,000621901	0,618432349	0,34	0,154	1
CDC14A	0,000684542	0,550815254	0,362	0,168	1
NAP1L1	0,000890162	0,419063314	0,809	0,644	1
RACK1	0,0008962	0,419648887	0,936	0,846	1
RFC2	0,00090555	0,569273835	0,255	0,101	1
NOP53	0,001061279	0,534638286	0,745	0,618	1
ATP8B2	0,001473056	0,569603586	0,298	0,137	1
TAB2	0,001562616	0,851335925	0,319	0,152	1
LRRC75A-AS1	0,001768166	0,682889984	0,468	0,261	1
SFSWAP	0,002070957	0,47182729	0,255	0,109	1
TESPA1	0,002071223	0,466796286	0,319	0,154	1
EIF3E	0,00223554	0,531223641	0,596	0,398	1
ARHGAP1	0,002719302	0,473246772	0,319	0,152	1
PGK1	0,002786078	0,472666067	0,638	0,44	1
EIF3M	0,002925379	0,492855945	0,34	0,17	1
MAP3K1	0,003220728	0,534832132	0,277	0,129	1
EEF1D	0,003749926	0,441131975	0,723	0,594	1
NUCB1	0,003942196	0,592805207	0,34	0,178	1
ZNHIT1	0,004123301	0,616989041	0,277	0,137	1
LINC00346	0,004385577	0,548842642	0,34	0,186	1
EEF1B2	0,004788787	0,58511905	0,766	0,699	1
NSD3	0,005027681	0,607964085	0,362	0,204	1
UNC45A	0,005082218	0,400079521	0,277	0,127	1
DDB1	0,005176527	0,380166698	0,298	0,143	1
PIK3IP1	0,005535214	0,34194524	0,383	0,202	1
SPTBN1	0,005595569	0,39761346	0,319	0,164	1
SNHG8	0,00591067	0,787585549	0,319	0,18	1
RTN3	0,006504888	0,472808865	0,255	0,119	1
GDI1	0,007044316	0,37271829	0,404	0,226	1
FAM106A	0,007619263	0,404029537	0,255	0,121	1
HNRNPF	0,007805072	0,375565244	0,426	0,244	1
COX7C	0,00815907	0,334505971	0,809	0,697	1

CS	0,008554912	0,576493234	0,298	0,145	1
TTC39C	0,00865607	0,373612124	0,34	0,182	1
ERGIC3	0,008764165	0,51213885	0,383	0,226	1
GATA3	0,009199683	0,798290156	0,362	0,208	1

Cluster My.0 marker genes

gene	p_val	avg_logFC	pct.1	pct.2	p_val_adj
S100A8	3,14E-65	2,997815431	0,787	0,136	5,94E-61
S100A9	1,85E-57	2,820792233	0,828	0,235	3,50E-53
FCN1	2,32E-46	1,638840439	0,799	0,249	4,40E-42
SERPINA1	1,10E-36	1,360454286	0,833	0,486	2,08E-32
VCAN	5,27E-32	1,508715084	0,718	0,3	9,99E-28
S100A12	7,32E-32	1,744292073	0,322	0,009	1,39E-27
CFP	3,36E-28	1,258111446	0,523	0,129	6,37E-24
CTSS	1,19E-24	0,919959051	0,874	0,795	2,26E-20
S100A4	5,28E-19	0,709788432	0,914	0,8	1,00E-14
FPR1	7,30E-19	1,075660153	0,569	0,251	1,38E-14
COTL1	6,11E-18	0,789829544	0,833	0,694	1,16E-13
CLEC12A	1,41E-15	0,824902693	0,293	0,06	2,67E-11
ICAM3	1,17E-13	0,995700588	0,287	0,071	2,21E-09
MYO1G	5,89E-13	0,952347278	0,402	0,157	1,12E-08
PABPC1	1,30E-12	0,480944381	0,897	0,876	2,47E-08
CSTA	2,35E-12	0,838652807	0,443	0,203	4,45E-08
LST1	6,68E-12	0,860714573	0,615	0,412	1,27E-07
PRAM1	8,78E-12	0,672918937	0,253	0,062	1,66E-07
TYMP	6,40E-11	0,623199543	0,736	0,631	1,21E-06
SRGN	7,05E-11	0,509901883	0,92	0,871	1,34E-06
CD52	1,41E-10	0,765537954	0,569	0,339	2,67E-06
NBEAL2	2,00E-10	0,881459538	0,305	0,106	3,79E-06
G0S2	2,04E-10	0,93959073	0,27	0,081	3,87E-06
TNFRSF1B	3,26E-10	0,703882291	0,678	0,544	6,18E-06
BCL2A1	4,06E-10	0,842789428	0,385	0,175	7,69E-06
CYBB	6,14E-10	0,560855117	0,759	0,62	1,16E-05
WARS	6,83E-10	1,022276731	0,414	0,217	1,29E-05
HLA-B	7,08E-10	0,370809691	0,943	0,924	1,34E-05
LYZ	8,94E-10	0,688410703	0,833	0,772	1,69E-05
VSIR	9,64E-10	0,747236995	0,454	0,242	1,83E-05
RIPOR2	1,66E-09	0,646645799	0,356	0,143	3,14E-05
CSF3R	1,66E-09	1,121805934	0,414	0,224	3,15E-05
CCDC69	1,93E-09	0,77553336	0,402	0,196	3,67E-05
CD300E	2,34E-09	0,64519662	0,362	0,154	4,43E-05
MYO1F	2,80E-09	0,761817935	0,523	0,353	5,30E-05
SLC11A1	4,95E-09	0,696799695	0,431	0,226	9,37E-05
NCF2	7,48E-09	0,670593656	0,517	0,327	0,000141829
PRKCB	1,49E-08	0,778917762	0,511	0,341	0,000282167
CD48	4,39E-08	0,721844019	0,517	0,343	0,000831839
IL1B	4,53E-08	0,693617657	0,471	0,267	0,000859197
DDR2	6,93E-08	0,864043935	0,477	0,32	0,001314138
CD37	1,19E-07	0,468027025	0,713	0,615	0,002254993
FKBP1A	1,24E-07	0,394258124	0,776	0,677	0,002341249
JUNB	1,49E-07	0,499665034	0,747	0,657	0,002814157
TIMP1	1,54E-07	0,960793182	0,764	0,728	0,002922085
AIF1	1,67E-07	0,501411907	0,741	0,691	0,003163952
FGR	1,76E-07	0,707044235	0,546	0,387	0,003328356
NAMPT	1,91E-07	0,683912522	0,603	0,454	0,003611751
PTPRC	2,06E-07	0,508965808	0,695	0,652	0,003902625
MEGF9	2,15E-07	0,733604418	0,368	0,196	0,004067124
HLA-C	3,39E-07	0,423135571	0,822	0,809	0,006420996
SECTM1	1,24E-06	0,643707898	0,408	0,24	0,023477551
NFAM1	1,30E-06	0,662994192	0,391	0,23	0,024697997

GRK2	1,37E-06	0,678073552	0,575	0,5	0,025986769
MNDA	1,53E-06	0,819248435	0,477	0,353	0,029016153
FYB1	1,62E-06	0,415958333	0,77	0,7	0,030615122
STXBP2	1,64E-06	0,746512017	0,385	0,242	0,031025035
SH3BGRL3	1,84E-06	0,300155768	0,943	0,922	0,034802472
OAZ1	2,99E-06	0,393463619	0,793	0,788	0,05665499
ADGRE5	4,55E-06	0,712398311	0,402	0,258	0,086183297
PTGS2	4,81E-06	0,554900036	0,299	0,143	0,091165795
GNAI2	4,94E-06	0,34588623	0,879	0,859	0,093679678
PECAM1	6,44E-06	0,676425	0,569	0,456	0,122063859
LILRB3	7,28E-06	0,5481407	0,437	0,276	0,138048337
NFKBIZ	7,56E-06	0,595903909	0,511	0,359	0,143299131
S100A6	7,97E-06	0,261132745	0,845	0,779	0,151076767
ITGAL	8,54E-06	0,651906674	0,414	0,26	0,161884301
POU2F2	1,14E-05	0,646132797	0,523	0,394	0,216429298
SELL	1,37E-05	0,641060011	0,385	0,228	0,260001336
RXRA	1,45E-05	0,531954187	0,368	0,224	0,275717536
CPPED1	1,62E-05	0,553267163	0,425	0,286	0,30785703
TNFSF10	1,74E-05	0,554094999	0,316	0,173	0,330560577
VMP1	1,79E-05	0,476569694	0,5	0,35	0,339344982
LILRB2	2,21E-05	0,675880135	0,546	0,442	0,419335465
TSPAN14	2,48E-05	0,424021434	0,471	0,334	0,46992708
IFITM2	5,17E-05	0,406148256	0,874	0,864	0,980484462
HCK	5,33E-05	0,649702574	0,534	0,475	1
SAT1	5,58E-05	0,326082519	0,805	0,781	1
AGTRAP	5,89E-05	0,581779244	0,282	0,154	1
EVI2B	5,91E-05	0,525210344	0,598	0,532	1
LYST	6,61E-05	0,566369412	0,333	0,203	1
RAB27A	8,04E-05	0,585641861	0,282	0,159	1
GMFG	8,65E-05	0,474354099	0,557	0,454	1
CARD16	8,72E-05	0,526129798	0,282	0,161	1
VASP	9,04E-05	0,596995908	0,489	0,401	1
SPN	0,000140773	0,543468608	0,385	0,256	1
GCA	0,000144991	0,641215153	0,322	0,21	1
RAC2	0,000155164	0,483939548	0,511	0,424	1
UBA52	0,000168953	0,304704411	0,874	0,869	1
HLA-F	0,000171979	0,513183499	0,351	0,237	1
HBEGF	0,000175401	0,511087487	0,385	0,253	1
GABARAP	0,000192977	0,289396992	0,828	0,804	1
HK3	0,000211154	0,392797926	0,282	0,157	1
CASP1	0,000252097	0,491879491	0,345	0,228	1
CFD	0,000263377	0,490096719	0,391	0,27	1
RGS2	0,000278037	0,585528759	0,425	0,297	1
PGM5P2	0,00030255	0,371151604	0,845	0,802	1
IER3	0,000372567	0,274271911	0,598	0,472	1
FGL2	0,000407982	0,406607143	0,592	0,5	1
JAML	0,000427843	0,427429945	0,437	0,311	1
SOD2	0,00051092	0,405067309	0,54	0,465	1
PFDN5	0,000511251	0,36816255	0,69	0,682	1
EMP3	0,000592634	0,366418702	0,678	0,597	1
SLC2A3	0,000601404	0,63987354	0,425	0,325	1
USP15	0,000644274	0,437609472	0,305	0,196	1
LY6E	0,000730781	0,500296119	0,448	0,35	1
PTP4A2	0,000758652	0,363581209	0,678	0,673	1
SULF2	0,000777663	0,385175551	0,259	0,145	1
GPSM3	0,000867566	0,42733417	0,5	0,426	1

SORL1	0,000884597	0,416192937	0,483	0,369	1
ARPC1B	0,000955927	0,377761961	0,632	0,594	1
AP1S2	0,001060569	0,482404216	0,489	0,417	1
ZFP36	0,001070027	0,394608848	0,603	0,548	1
GPCPD1	0,00124182	0,572650832	0,356	0,256	1
LIMD2	0,001317368	0,462182148	0,368	0,26	1
ZNF652	0,001321417	0,468056773	0,328	0,219	1
MPEG1	0,001379541	0,291816046	0,598	0,502	1
IL17RA	0,001641131	0,470336028	0,477	0,394	1
EHBP1L1	0,001780908	0,394923955	0,374	0,276	1
TIGD1	0,001814138	0,371396138	0,466	0,364	1
IRAK3	0,001935874	0,325907267	0,259	0,157	1
IRF1	0,00211834	0,521396482	0,391	0,309	1
NEAT1	0,002230184	0,309017547	0,644	0,634	1
SCIMP	0,002358301	0,459414095	0,253	0,161	1
SRRM4	0,002415912	0,468621094	0,316	0,212	1
STX10	0,002510069	0,482711545	0,253	0,164	1
FOSL2	0,002671586	0,354500469	0,253	0,154	1
STK17B	0,002732659	0,385572634	0,477	0,387	1
RASSF5	0,00287427	0,392758238	0,414	0,325	1
LOC100131257	0,002885813	0,348440695	0,971	0,961	1
CFLAR	0,002907163	0,359132885	0,839	0,839	1
TMA7	0,003192987	0,257217802	0,598	0,544	1
SLC7A5P2	0,003319839	0,475308039	0,253	0,157	1
CNPY3	0,003835178	0,407511945	0,494	0,422	1
CAPNS1	0,003855726	0,323302021	0,621	0,569	1
PLXNC1	0,003939705	0,381811069	0,31	0,217	1
MAB21L3	0,003956821	0,319046637	0,925	0,912	1
PGD	0,0039888	0,547008348	0,328	0,249	1
SP100	0,004253823	0,415878463	0,707	0,705	1
CWF19L1	0,004539056	0,438488746	0,259	0,173	1
CYBA	0,004575478	0,286698774	0,69	0,724	1
SIGLEC10	0,004672541	0,492894939	0,339	0,256	1
ZNF471	0,005812874	0,439137313	0,655	0,645	1
GLTP	0,006139634	0,366578596	0,379	0,286	1
NFKBIA	0,006388007	0,374628814	0,655	0,624	1
RNF141	0,00663644	0,386934838	0,259	0,173	1
TRMT10B	0,006796739	0,314567907	0,27	0,177	1
PSMB9	0,007240528	0,413567079	0,27	0,187	1
GIMAP4	0,007897538	0,418077109	0,333	0,251	1
SHKBP1	0,007949341	0,455852065	0,362	0,295	1
SMCHD1	0,008986157	0,371766525	0,282	0,198	1
NR4A1	0,009228808	0,411249961	0,259	0,177	1

Cluster My.1 marker genes

gene	p_val	avg_logFC	pct.1	pct.2	p_val_adj
C1QC	1,42E-54	1,717032637	0,876	0,285	2,69E-50
C1QA	3,38E-53	1,820451828	0,906	0,322	6,41E-49
C1QB	3,69E-51	1,87836209	0,871	0,324	6,99E-47
FOLR2	2,29E-38	1,472117445	0,5	0,057	4,33E-34
SLC40A1	1,80E-35	1,552753403	0,571	0,11	3,42E-31
DAB2	4,57E-29	1,185419676	0,712	0,267	8,65E-25
MS4A4A	1,47E-24	1,072778866	0,812	0,466	2,79E-20
MARCKS	9,27E-24	0,809160394	0,888	0,555	1,76E-19
SLCO2B1	6,44E-22	1,085113004	0,553	0,194	1,22E-17
MAF	5,60E-21	1,089826086	0,618	0,249	1,06E-16
LGMN	8,86E-21	1,12047217	0,565	0,203	1,68E-16
IGF1	1,13E-20	1,083622759	0,276	0,027	2,13E-16
MSR1	4,13E-19	0,851368905	0,729	0,356	7,83E-15
HLA-DRB1	4,69E-19	0,519993687	0,988	0,904	8,88E-15
CD74	6,34E-19	0,55071629	1	0,991	1,20E-14
F13A1	2,85E-18	1,17998211	0,5	0,18	5,40E-14
SIGLEC1	5,22E-18	0,891743304	0,382	0,091	9,90E-14
CXCL12	3,40E-17	0,893280503	0,253	0,032	6,45E-13
IGSF21	4,41E-17	0,822717036	0,324	0,066	8,36E-13
PLTP	8,73E-17	0,955169595	0,688	0,356	1,65E-12
MS4A6A	1,08E-16	0,767952444	0,794	0,486	2,04E-12
STAB1	1,45E-15	0,922850069	0,547	0,228	2,75E-11
HLA-DPB1	3,32E-15	0,408017661	0,965	0,763	6,29E-11
FCGR3A	3,57E-15	0,818485391	0,559	0,242	6,77E-11
MAFB	5,88E-15	0,692904792	0,829	0,555	1,11E-10
CD14	6,31E-15	0,608769431	0,876	0,582	1,20E-10
GGTA1P	2,80E-14	0,68678228	0,412	0,137	5,31E-10
HLA-DPA1	3,02E-14	0,446040462	0,971	0,888	5,72E-10
CCL3	3,66E-14	0,94194205	0,529	0,242	6,93E-10
ME1	6,15E-14	0,544947883	0,288	0,064	1,16E-09
HLA-DRB6	9,75E-14	0,621145738	0,753	0,484	1,85E-09
VSIG4	9,89E-14	0,761370385	0,582	0,285	1,87E-09
CD163	1,98E-13	0,87691183	0,688	0,406	3,76E-09
CTSB	2,56E-13	0,3681497	0,971	0,874	4,86E-09
FCGR2A	3,41E-13	0,981207642	0,529	0,274	6,45E-09
HLA-DRA	8,78E-13	0,433200611	0,988	0,922	1,66E-08
GPR34	8,46E-12	0,647066556	0,288	0,078	1,60E-07
GAS6	9,90E-12	0,691407076	0,376	0,139	1,88E-07
OLFML2B	1,12E-11	0,661352037	0,359	0,123	2,13E-07
EGR1	2,04E-11	0,722876014	0,712	0,479	3,87E-07
CMKLR1	3,60E-11	0,654823744	0,282	0,082	6,81E-07
HLA-DMA	4,19E-11	0,564727718	0,741	0,511	7,95E-07
TMEM176B	5,71E-11	0,76046221	0,588	0,326	1,08E-06
FUCA1	9,03E-11	0,721514109	0,353	0,13	1,71E-06
C2	1,11E-10	0,762295742	0,312	0,103	2,10E-06
CSF1R	1,14E-10	0,572234074	0,741	0,507	2,16E-06
NELL1	1,30E-10	0,683656928	0,459	0,21	2,47E-06
C3AR1	1,48E-10	0,600060209	0,5	0,249	2,80E-06
MGAT4A	2,24E-10	0,5072977	0,435	0,192	4,24E-06
SDC3	2,95E-10	0,629547139	0,4	0,167	5,59E-06
JUN	3,14E-10	0,685292863	0,465	0,224	5,95E-06
AP1B1	4,52E-10	0,562431934	0,565	0,322	8,57E-06
SERPING1	4,80E-10	0,57324621	0,335	0,123	9,09E-06

KLF6	5,34E-10	0,586394178	0,847	0,728	1,01E-05
HLA-DQA1	1,04E-09	0,337167608	0,829	0,58	1,98E-05
GNAO1	1,26E-09	0,355192982	0,853	0,639	2,39E-05
LPAR6	1,28E-09	0,504794411	0,453	0,215	2,42E-05
CTSC	1,75E-09	0,565696053	0,753	0,589	3,31E-05
HLA-DMB	2,87E-09	0,459719979	0,741	0,484	5,44E-05
AKR1B1	3,98E-09	0,644108353	0,429	0,205	7,55E-05
GPR155	4,11E-09	0,458629808	0,565	0,299	7,78E-05
RGL1	5,27E-09	0,805584547	0,276	0,098	9,98E-05
ADAM28	8,26E-09	0,498250483	0,312	0,114	0,000156536
ZFHX3	9,57E-09	0,720288972	0,553	0,329	0,000181357
CCDC170	2,07E-08	0,572437065	0,259	0,089	0,000392903
USP53	2,92E-08	0,606074029	0,253	0,087	0,000552769
MKNK1	3,01E-08	0,585754663	0,335	0,146	0,000570798
AP2A2	4,23E-08	0,580537785	0,371	0,174	0,000802494
FCGRT	4,31E-08	0,427113489	0,847	0,708	0,000816228
FRMD4A	4,51E-08	0,567221331	0,265	0,096	0,000853983
NCF4	5,04E-08	0,418661603	0,447	0,224	0,000955896
A2M	6,65E-08	0,548937751	0,382	0,183	0,00126108
PDK4	8,19E-08	0,622061444	0,353	0,158	0,00155133
GLIPR1L2	1,04E-07	0,724646002	0,529	0,315	0,001970116
PDGFB	1,08E-07	0,449681646	0,271	0,098	0,00204695
IER3	1,09E-07	0,600082455	0,653	0,452	0,002074248
ZFP36L1	1,14E-07	0,461810383	0,894	0,797	0,00216483
CREG1	1,48E-07	0,526360384	0,571	0,372	0,002795633
SGK1	1,70E-07	0,592706146	0,524	0,308	0,003225728
ITM2B	1,75E-07	0,359321451	0,9	0,874	0,003314717
TLR7	1,84E-07	0,515490506	0,306	0,128	0,003487585
RB1	2,17E-07	0,506105544	0,394	0,205	0,00410319
BTG2	2,64E-07	0,425066468	0,371	0,176	0,005000336
HLA-DOA	2,65E-07	0,466006407	0,418	0,217	0,005025077
FOS	2,89E-07	0,586028703	0,794	0,708	0,005479945
LAIR1	3,20E-07	0,426411088	0,588	0,361	0,006057189
SPRED1	3,23E-07	0,371444625	0,376	0,18	0,006129024
HMOX1	3,52E-07	0,281519457	0,353	0,16	0,006678134
MERTK	5,01E-07	0,445547238	0,253	0,096	0,009493898
CREBL2	5,57E-07	0,517555175	0,424	0,242	0,01054738
TMEM176A	8,50E-07	0,524307935	0,412	0,221	0,016106115
RNASE1	8,61E-07	0,885578077	0,576	0,4	0,016312147
NPC2	9,16E-07	0,324235122	0,829	0,701	0,017364547
CD93	1,10E-06	0,447695112	0,547	0,34	0,02077311
KCTD12	1,32E-06	0,446034526	0,665	0,493	0,025009127
ITSN1	1,68E-06	0,516978483	0,253	0,105	0,031909943
RGS1	1,81E-06	0,498120167	0,347	0,169	0,034338614
CPM	1,87E-06	0,611007455	0,494	0,308	0,035409858
ADAP2	1,98E-06	0,578443429	0,559	0,374	0,037498131
FAM20A	2,53E-06	0,43585106	0,253	0,103	0,04798004
KCNMA1	2,56E-06	0,380300519	0,294	0,132	0,04843764
DSC2	3,07E-06	0,465745395	0,253	0,107	0,058201775
CXCL3	4,44E-06	0,765643477	0,341	0,183	0,084133955
ADAM9	4,87E-06	0,408395413	0,353	0,18	0,092351487
EMP2	5,65E-06	0,360510776	0,341	0,176	0,107110188
TLR4	5,93E-06	0,412335014	0,359	0,192	0,112452775
GNB4	6,68E-06	0,357116682	0,453	0,276	0,126539758
PLAU	7,16E-06	0,456545219	0,341	0,174	0,135750038
HERPUD1	7,56E-06	0,550298499	0,435	0,269	0,143246039

MEF2A	8,94E-06	0,399461873	0,441	0,258	0,169338278
RCBTB2	9,53E-06	0,305366976	0,3	0,139	0,180543521
TSPAN4	9,99E-06	0,398858083	0,259	0,114	0,189224484
ASAH1	1,00E-05	0,379032553	0,635	0,466	0,190256938
PMP22	1,05E-05	0,359195292	0,294	0,144	0,198696681
HEXA	1,05E-05	0,386145686	0,371	0,203	0,199110018
ATP6AP1	1,08E-05	0,488101578	0,447	0,276	0,204002433
CD84	1,10E-05	0,422911393	0,676	0,509	0,208298838
CREM	1,26E-05	0,458598519	0,288	0,137	0,23807452
TPCN1	1,32E-05	0,342259039	0,282	0,13	0,249635481
PIK3IP1	1,36E-05	0,346512363	0,253	0,112	0,257171721
TIMP2	1,37E-05	0,367628724	0,594	0,447	0,259546964
LRP1	1,53E-05	0,29828654	0,576	0,381	0,289728971
CXCL16	1,61E-05	0,425570314	0,682	0,546	0,305903596
PLXND1	1,67E-05	0,460265737	0,388	0,228	0,316995515
IQGAP2	1,70E-05	0,399641654	0,447	0,274	0,322832453
SASH1	2,26E-05	0,390340989	0,253	0,119	0,428108835
ELL2	2,35E-05	0,419047987	0,3	0,151	0,445420243
CTSD	2,49E-05	0,40127737	0,776	0,664	0,472139245
TCF4	2,81E-05	0,344434059	0,494	0,311	0,532356109
CCL4	3,59E-05	0,858449827	0,465	0,326	0,679391567
ST8SIA4	3,87E-05	0,350064341	0,347	0,194	0,734042685
ARHGAP18	4,25E-05	0,321829456	0,412	0,242	0,804971455
RGS10	4,26E-05	0,338852131	0,488	0,313	0,806850631
ST6GAL1	4,26E-05	0,314369189	0,259	0,126	0,808005061
TNFSF12	4,78E-05	0,333786124	0,306	0,16	0,906711409
CEL	6,09E-05	0,383246883	0,571	0,422	1
HLA-DRB5	6,10E-05	0,414444387	0,535	0,363	1
IL10RA	7,01E-05	0,363708478	0,465	0,311	1
TMEM51	7,01E-05	0,404053183	0,3	0,158	1
PRNP	7,67E-05	0,280489296	0,359	0,199	1
RUNX1	7,86E-05	0,49106922	0,412	0,276	1
ZNF829	8,17E-05	0,410207616	0,359	0,205	1
SNX6	8,32E-05	0,343058637	0,412	0,26	1
SAT1	8,52E-05	0,27167915	0,853	0,763	1
SLAMF8	8,54E-05	0,391355613	0,329	0,192	1
SLC15A3	9,46E-05	0,367722375	0,441	0,272	1
NFKBIA	0,000103667	0,387187669	0,712	0,603	1
NAIP	0,00012708	0,296328759	0,4	0,235	1
C6orf62	0,000148013	0,441317138	0,482	0,331	1
RAB32	0,000154943	0,328370639	0,353	0,21	1
TXNIP	0,000156768	0,349320556	0,659	0,541	1
TNF	0,000166955	0,544721253	0,271	0,148	1
PGM5P2	0,000171731	0,513311472	0,876	0,79	1
ITPR2	0,000198596	0,461474333	0,365	0,231	1
DNASE2	0,000206334	0,349368756	0,253	0,13	1
DRAM2	0,000229409	0,317759123	0,353	0,212	1
TGFBR2	0,000230887	0,304454967	0,306	0,171	1
TACC1	0,000242689	0,278067786	0,571	0,418	1
FRMD4B	0,00024303	0,344790538	0,294	0,164	1
APMAP	0,000251481	0,350327995	0,282	0,16	1
EIF5	0,000265437	0,325992786	0,429	0,283	1
ABCA1	0,000270941	0,292032049	0,424	0,26	1
ASTN2	0,000290471	0,590151891	0,594	0,441	1
C20orf194	0,000319707	0,292307758	0,259	0,139	1
RHOB	0,00036485	0,324011055	0,265	0,144	1

YWHAH	0,000438322	0,320976225	0,476	0,336	1
HNMT	0,000464483	0,379319215	0,406	0,269	1
P2RY13	0,000544858	0,299866545	0,276	0,151	1
CXCL2	0,000545338	0,509668909	0,388	0,26	1
USP9X	0,000567246	0,281468522	0,394	0,263	1
MFSD1	0,000575763	0,257245062	0,518	0,377	1
GPNMB	0,000615712	0,555183488	0,518	0,368	1
PEBP1	0,000617411	0,355866644	0,606	0,447	1
CCL2	0,000662457	0,651417398	0,312	0,189	1
HTRA1	0,000675622	0,340597517	0,271	0,151	1
DUSP3	0,000697133	0,300787127	0,424	0,285	1
RRBP1	0,000699627	0,390021874	0,294	0,183	1
MAN1A1	0,00072254	0,326278268	0,329	0,201	1
NFIC	0,000732598	0,337569541	0,518	0,37	1
ST8SIA1	0,000735117	0,439786128	0,288	0,174	1
GPX3	0,000754003	0,317693778	0,253	0,142	1
SLC18A1	0,000786078	0,299925928	0,376	0,237	1
RNF130	0,000795703	0,279821532	0,565	0,416	1
TNFRSF1A	0,000918813	0,25508816	0,335	0,208	1
TBXAS1	0,000925972	0,381450279	0,394	0,265	1
ANKRD12	0,000982686	0,313098255	0,676	0,518	1
CREBRF	0,000992941	0,369141355	0,376	0,256	1
GDE1	0,001101012	0,368894376	0,341	0,228	1
IFI16	0,001178827	0,301214985	0,518	0,379	1
SLC4A7	0,001188203	0,390520361	0,382	0,26	1
FOXN3	0,001275223	0,296088963	0,541	0,402	1
TGFBR1	0,001470333	0,360544837	0,312	0,196	1
NPL	0,001550696	0,304755861	0,276	0,164	1
AKR1A1	0,001580851	0,290787819	0,412	0,283	1
RBM47	0,001622872	0,313272851	0,547	0,425	1
PHAX	0,001647751	0,282280166	0,253	0,148	1
ST3GAL6	0,001778322	0,264697404	0,271	0,164	1
CXCL8	0,001840648	0,471965972	0,429	0,317	1
TMEM212	0,001934884	0,416348187	0,788	0,701	1
PIK3R1	0,002088831	0,319207756	0,312	0,194	1
MAB21L3	0,002206368	0,486707494	0,947	0,904	1
CD81	0,002336552	0,30134524	0,671	0,566	1
LAMP2	0,002468992	0,331803035	0,494	0,372	1
CYP20A1	0,00250687	0,255194479	0,576	0,443	1
DUSP6	0,002551389	0,440992341	0,406	0,304	1
IDH1	0,00266193	0,375460403	0,271	0,169	1
PEPD	0,002890487	0,30095225	0,341	0,228	1
CD59	0,002957526	0,295726891	0,429	0,311	1
ASB4	0,003128638	0,360302239	0,294	0,192	1
FPR3	0,003160386	0,282788697	0,324	0,21	1
PLEKHA5	0,003510511	0,394596684	0,335	0,228	1
HOOK3	0,003619499	0,260469625	0,547	0,427	1
LOC441081	0,003766798	0,379757813	0,353	0,237	1
PBRM1	0,003768421	0,368573626	0,324	0,219	1
CXCR4	0,003799384	0,254197649	0,435	0,311	1
RAB3IP	0,003872879	0,322850283	0,335	0,228	1
CLTA	0,004039175	0,298331684	0,376	0,263	1
SPOP	0,004508565	0,28361935	0,394	0,274	1
EPS15	0,004511998	0,297194179	0,324	0,219	1
TCF25	0,004828301	0,29133694	0,412	0,297	1
MED13L	0,004838917	0,314049129	0,359	0,249	1

ARL4C	0,004990269	0,316622609	0,535	0,418	1
NR4A2	0,005179515	0,436383717	0,435	0,313	1
ZBTB4	0,005179714	0,322505718	0,271	0,176	1
NCOA4	0,005212802	0,283495011	0,524	0,422	1
MBD1	0,005219607	0,273649169	0,324	0,219	1
SYNE1	0,005505775	0,297396312	0,265	0,169	1
WASHC4	0,005546942	0,283195266	0,329	0,226	1
SAA1	0,005692105	0,360340916	0,388	0,281	1
RNASE6	0,005950819	0,289516647	0,459	0,336	1
CCDC144B	0,006180317	0,388027164	0,724	0,635	1
TYW3	0,006252965	0,316555844	0,406	0,299	1
LOC100131257	0,006521608	0,404127129	0,982	0,957	1
DUSP1	0,006685302	0,313102692	0,535	0,429	1
HSD3BP4	0,007562246	0,312729439	0,271	0,176	1
SREK1IP1	0,007742391	0,317450659	0,329	0,228	1
mrt-01	0,008481713	0,273738855	0,412	0,311	1
USP33	0,008587601	0,433828355	0,641	0,575	1
XRCC2	0,008698676	0,364826482	0,394	0,29	1
XAF1	0,009029093	0,416290408	0,829	0,731	1

Cluster My.2 marker genes

gene	p_val	avg_logFC	pct.1	pct.2	p_val_adj
SPP1	6,33E-53	2,781235983	0,864	0,261	1,20E-48
APOE	1,48E-43	1,801406224	0,814	0,233	2,81E-39
APOC1	3,88E-42	1,84841261	0,671	0,124	7,35E-38
MMP9	1,30E-41	2,495981266	0,543	0,064	2,46E-37
FN1	2,55E-40	2,442744216	0,779	0,276	4,82E-36
VIM	9,55E-38	1,140659621	0,971	0,882	1,81E-33
GPNMB	1,23E-33	1,08872556	0,821	0,286	2,33E-29
CSTB	3,16E-33	1,70550327	0,836	0,449	5,99E-29
S100A10	3,12E-28	1,27007321	0,871	0,609	5,91E-24
MATK	6,42E-26	0,777813336	0,279	0,013	1,22E-21
FABP5	8,68E-26	1,571426098	0,693	0,286	1,65E-21
RNASE1	6,82E-25	0,835635434	0,829	0,335	1,29E-20
LMNA	8,73E-25	0,997192747	0,743	0,306	1,65E-20
CTSB	4,90E-24	1,011988227	0,964	0,882	9,28E-20
MMP19	6,40E-24	1,313058968	0,364	0,049	1,21E-19
SCD	2,22E-21	0,952122119	0,471	0,111	4,20E-17
CAPG	3,49E-20	0,92059934	0,721	0,344	6,62E-16
CYP27A1	8,01E-20	0,926196167	0,379	0,075	1,52E-15
NR1H3	1,15E-19	0,831408485	0,343	0,058	2,18E-15
EMP1	1,18E-19	0,873292164	0,421	0,096	2,24E-15
LHFPL2	1,48E-19	0,876576643	0,514	0,15	2,81E-15
PLD3	5,71E-19	1,05750155	0,671	0,342	1,08E-14
SLC18A1	2,07E-18	0,856733282	0,557	0,192	3,92E-14
GSN	7,69E-18	0,893161791	0,807	0,553	1,46E-13
TUBA1C	9,96E-18	1,077033965	0,571	0,224	1,89E-13
CD276	2,39E-17	0,690451277	0,264	0,034	4,53E-13
MARCO	3,49E-17	0,987664946	0,307	0,053	6,62E-13
TNS1	5,97E-17	0,767824989	0,4	0,098	1,13E-12
PKM	1,05E-16	0,657800067	0,921	0,776	1,99E-12
SLC39A8	1,21E-15	0,883057506	0,321	0,068	2,29E-11
CD9	2,47E-15	0,987972594	0,564	0,241	4,69E-11
ITGB5	4,05E-15	0,731514566	0,4	0,111	7,68E-11
RAB13	4,28E-15	0,709448972	0,421	0,126	8,12E-11
S100A6	5,26E-15	0,688839165	0,929	0,759	9,97E-11
LGALS3	5,35E-15	0,861245105	0,707	0,395	1,01E-10
FAM129B	5,61E-15	0,650285762	0,436	0,128	1,06E-10
SLC43A3	8,33E-15	0,745491321	0,521	0,205	1,58E-10
FTL	1,09E-14	0,801487751	0,993	0,981	2,07E-10
SDC2	1,57E-14	0,948476165	0,393	0,115	2,98E-10
COL6A2	1,68E-14	1,055473032	0,279	0,056	3,18E-10
CD109	2,46E-14	0,592251245	0,371	0,096	4,67E-10
IL4I1	3,99E-14	0,653713438	0,286	0,06	7,56E-10
TREM2	4,14E-14	0,742269046	0,364	0,098	7,84E-10
CD63	9,07E-14	0,734311202	0,843	0,613	1,72E-09
MITF	1,48E-13	0,66368845	0,429	0,139	2,81E-09
LGALS1	2,68E-13	0,809172678	0,793	0,571	5,08E-09
FBP1	5,46E-13	0,917155094	0,514	0,216	1,03E-08
CTSL	6,64E-13	0,971893357	0,586	0,278	1,26E-08
ALDOA	1,14E-12	0,716829461	0,764	0,536	2,16E-08
ACP5	3,33E-12	1,045473376	0,393	0,135	6,31E-08
ANXA2	4,02E-12	0,744114604	0,764	0,594	7,62E-08
FAM20C	4,34E-12	0,616998164	0,343	0,103	8,22E-08
RGCC	5,79E-12	0,795056506	0,4	0,137	1,10E-07

HTRA1	7,42E-12	0,800364829	0,379	0,126	1,41E-07
PAPSS1	2,31E-11	0,561414111	0,429	0,165	4,37E-07
PLIN2	3,00E-11	0,858728298	0,429	0,175	5,68E-07
LDHA	3,39E-11	0,789504641	0,693	0,451	6,42E-07
LAPTM5	3,64E-11	0,478647195	0,921	0,861	6,90E-07
YWHAG	3,65E-11	0,623845205	0,543	0,269	6,91E-07
ANPEP	3,88E-11	0,756672932	0,514	0,246	7,34E-07
PLTP	3,93E-11	0,511829242	0,7	0,374	7,45E-07
ABCA1	4,01E-11	0,75634031	0,514	0,244	7,60E-07
PLEC	5,48E-11	0,53747172	0,593	0,303	1,04E-06
CTSD	7,77E-11	0,876194851	0,814	0,66	1,47E-06
MAPK13	7,79E-11	0,668315781	0,336	0,109	1,48E-06
NELL1	8,35E-11	0,503734187	0,5	0,214	1,58E-06
PDXK	1,04E-10	0,600735585	0,479	0,207	1,98E-06
GALM	1,14E-10	0,479330238	0,414	0,152	2,17E-06
MPP1	1,24E-10	0,695844717	0,507	0,231	2,35E-06
NME1	1,35E-10	0,616013308	0,321	0,1	2,55E-06
CCDC88A	1,60E-10	0,640513724	0,736	0,485	3,04E-06
SPARC	3,41E-10	0,437161979	0,443	0,175	6,47E-06
FLNB	4,18E-10	0,493835822	0,257	0,066	7,92E-06
HSD3B7	4,31E-10	0,507518406	0,286	0,083	8,17E-06
NPC2	4,71E-10	0,50704427	0,871	0,697	8,93E-06
GSTO1	6,46E-10	0,516150387	0,629	0,344	1,22E-05
ARHGEF10L	8,18E-10	0,426322074	0,264	0,073	1,55E-05
CALM1	1,36E-09	0,46160492	0,929	0,835	2,59E-05
FLNA	1,87E-09	0,611507515	0,714	0,489	3,54E-05
MYO1E	2,12E-09	0,464115132	0,293	0,094	4,02E-05
PRDX1	2,86E-09	0,684492887	0,614	0,376	5,42E-05
CRIP1	4,51E-09	0,773514023	0,614	0,389	8,55E-05
BCAT1	4,73E-09	0,556498252	0,557	0,31	8,96E-05
TUBB6	4,97E-09	0,423343796	0,293	0,094	9,42E-05
TUBB2A	5,04E-09	0,618803629	0,7	0,496	9,55E-05
MYOF	7,42E-09	0,454502289	0,35	0,128	0,000140571
TXN	1,01E-08	0,481343372	0,5	0,252	0,000191366
PLOD1	1,21E-08	0,55441024	0,307	0,109	0,000229516
PPP1R14B	1,83E-08	0,521852815	0,264	0,085	0,000345901
ATP6V1F	2,03E-08	0,560561624	0,586	0,37	0,000384536
GNAS	2,05E-08	0,371391287	0,936	0,874	0,000387883
COL6A1	2,64E-08	0,772009652	0,307	0,118	0,000499593
SLC38A6	2,82E-08	0,332888076	0,257	0,079	0,000534345
LILRB4	4,31E-08	0,411021445	0,5	0,256	0,000817302
CD82	4,32E-08	0,405503992	0,329	0,124	0,000818019
ENO1	5,00E-08	0,551638178	0,771	0,643	0,000947871
GPI	6,10E-08	0,574672967	0,529	0,295	0,001155555
ADAM9	6,84E-08	0,445844798	0,393	0,179	0,00129573
MFSD10	7,23E-08	0,351378955	0,307	0,111	0,001370571
TGFBR1	8,10E-08	0,415287842	0,4	0,177	0,001535539
ALCAM	8,12E-08	0,707065939	0,457	0,233	0,001538392
HSPD1	8,76E-08	0,56136534	0,471	0,235	0,001660111
DSTN	9,00E-08	0,380395931	0,4	0,179	0,001705017
PHLDA1	9,24E-08	0,485612638	0,286	0,1	0,001751033
CD68	1,08E-07	0,605460631	0,821	0,65	0,002038003
CCR1	1,18E-07	0,416522309	0,586	0,31	0,002233653
MGLL	1,22E-07	0,562398693	0,307	0,12	0,002303749
NCEH1	1,29E-07	0,285041169	0,257	0,083	0,002438491
ANXA5	1,32E-07	0,532775798	0,807	0,675	0,002492411

CD151	1,32E-07	0,498698568	0,436	0,214	0,002501474
ACTN1	1,42E-07	0,480629983	0,7	0,464	0,002699347
TOMM5	1,72E-07	0,468596768	0,279	0,105	0,003250261
ENG	1,91E-07	0,431594161	0,479	0,259	0,003611407
SOX4	1,94E-07	0,536075505	0,386	0,184	0,003683952
SLC16A3	2,07E-07	0,572810381	0,493	0,269	0,003918921
ARL6IP1	2,53E-07	0,493399055	0,564	0,327	0,004800342
TNS3	2,78E-07	0,261563996	0,293	0,107	0,00526485
ABCG1	2,94E-07	0,572428201	0,3	0,122	0,005568983
ACP2	3,06E-07	0,433651062	0,264	0,096	0,005791276
CTSZ	3,42E-07	0,799019146	0,529	0,318	0,006488496
HSPB1	4,14E-07	0,510466148	0,7	0,517	0,007836756
EMP3	4,20E-07	0,441910142	0,757	0,579	0,007964017
SLC1A3	4,23E-07	0,308796118	0,279	0,103	0,008017212
CORO1C	4,27E-07	0,382205563	0,4	0,188	0,008089096
PTMS	4,30E-07	0,563300197	0,493	0,274	0,008141211
RALA	4,63E-07	0,533174218	0,379	0,182	0,008768065
GLA	5,76E-07	0,392037711	0,286	0,113	0,010909325
ANXA4	5,79E-07	0,379784679	0,307	0,128	0,010971718
TAGLN2	5,92E-07	0,408575517	0,65	0,436	0,011209215
RGS1	6,25E-07	0,537214413	0,371	0,173	0,011852605
PLAU	7,82E-07	0,635032423	0,364	0,177	0,014817523
NTAN1	8,48E-07	0,449988437	0,293	0,122	0,016060945
GGA1	8,64E-07	0,453566312	0,379	0,177	0,016375467
AGAP3	9,40E-07	0,426238842	0,279	0,111	0,017818966
FAM107B	1,02E-06	0,354977756	0,436	0,22	0,019281802
PI4K2A	1,59E-06	0,377121923	0,279	0,111	0,030147456
TPM4	1,63E-06	0,41583841	0,707	0,526	0,030814096
S100A11	1,87E-06	0,375467226	0,921	0,853	0,035405853
VDAC1	1,89E-06	0,399960335	0,543	0,342	0,035762474
ZNF395	1,98E-06	0,443876742	0,264	0,105	0,037580249
CD44	2,08E-06	0,394764561	0,736	0,573	0,039494903
ATP6AP2	2,11E-06	0,494228213	0,664	0,485	0,039941422
CARD19	2,17E-06	0,366662187	0,264	0,105	0,041096393
SLC37A2	3,32E-06	0,408506644	0,457	0,246	0,062888258
P4HB	3,37E-06	0,529302453	0,564	0,368	0,063847831
KCNMA1	3,41E-06	0,326608194	0,314	0,137	0,064554711
ZC3H15	3,48E-06	0,276703002	0,307	0,132	0,066032953
GNPTG	4,10E-06	0,340574384	0,314	0,141	0,077725099
PDIA3	4,22E-06	0,382024744	0,429	0,226	0,080031521
PSMC3	4,75E-06	0,334506918	0,279	0,115	0,090066339
SLC11A2	5,01E-06	0,34307858	0,35	0,16	0,094951791
SH3BGRL3	5,22E-06	0,25989147	0,971	0,915	0,099000037
CALM3	5,54E-06	0,556968043	0,514	0,325	0,104949856
PEBP1	5,76E-06	0,383514281	0,643	0,447	0,109220455
FNDC3B	6,00E-06	0,355195848	0,386	0,197	0,113709975
DPY30	6,05E-06	0,309940612	0,257	0,103	0,114574247
PPIB	6,42E-06	0,413397596	0,429	0,235	0,1216655
PLXND1	6,61E-06	0,346556694	0,429	0,226	0,125341091
RHOC	7,00E-06	0,326348652	0,35	0,169	0,132676447
MGST3	7,19E-06	0,316014689	0,457	0,25	0,136156111
MRPL34	7,75E-06	0,391903248	0,264	0,111	0,146925635
CSE1L	8,17E-06	0,368374989	0,271	0,115	0,154746127
TWF2	8,81E-06	0,416565967	0,386	0,205	0,166920836
FKBP2	9,48E-06	0,322629819	0,379	0,192	0,179667108
TSPAN3	1,01E-05	0,5189608	0,35	0,179	0,190478819

RDX	1,05E-05	0,319675407	0,35	0,167	0,198260677
CALU	1,08E-05	0,378517095	0,436	0,252	0,204339658
MSR1	1,09E-05	0,426792134	0,607	0,417	0,207212248
CALR	1,14E-05	0,57388295	0,671	0,496	0,215824567
TXNDC17	1,20E-05	0,355445909	0,271	0,12	0,226977181
C19orf70	1,26E-05	0,28121114	0,264	0,111	0,23813122
FPR3	1,34E-05	0,284128125	0,386	0,199	0,253795612
GAPDH	1,40E-05	0,404674097	0,864	0,818	0,266079319
RNH1	1,41E-05	0,433756869	0,55	0,335	0,267739244
IRAK1	1,47E-05	0,414221976	0,371	0,19	0,278181084
TMEM51	1,48E-05	0,385923197	0,329	0,158	0,280050055
SCARB2	1,59E-05	0,387674565	0,407	0,22	0,301031138
ST14	1,62E-05	0,35643586	0,336	0,165	0,307753678
PGAM1	1,75E-05	0,338731115	0,407	0,224	0,332303313
FTH1	1,91E-05	0,508963734	1	0,97	0,361226441
ITGA5	1,95E-05	0,291179486	0,429	0,237	0,369614676
PLP2	1,98E-05	0,350228265	0,479	0,291	0,375463415
ZNF706	2,08E-05	0,325103598	0,343	0,177	0,39484901
HAVCR2	2,86E-05	0,300294284	0,343	0,171	0,541085061
SPTAN1	2,91E-05	0,389276111	0,364	0,186	0,550797494
CEBPB	2,99E-05	0,511285855	0,507	0,323	0,566589484
DHRS3	3,00E-05	0,352944575	0,264	0,118	0,568161798
TSPO	3,31E-05	0,3355193	0,579	0,419	0,627836536
TTYH3	3,38E-05	0,5094274	0,514	0,355	0,640248838
BCAP31	3,51E-05	0,335494002	0,536	0,335	0,66428793
GPX4	3,54E-05	0,416613107	0,571	0,425	0,67013904
IER3IP1	3,62E-05	0,370036526	0,257	0,115	0,686465551
VKORC1	3,89E-05	0,446303596	0,357	0,19	0,737738857
ATF5	4,20E-05	0,404778117	0,286	0,137	0,795065945
MTSS1	4,31E-05	0,364890523	0,393	0,209	0,816952362
CD36	4,36E-05	0,917681507	0,436	0,284	0,82561079
HEXB	4,83E-05	0,472624411	0,464	0,282	0,914645629
HSPE1	4,83E-05	0,287516848	0,364	0,192	0,916072644
CDK6	5,01E-05	0,312830553	0,321	0,158	0,950214112
IFI6	5,31E-05	0,583569608	0,521	0,338	1
CLEC5A	5,43E-05	0,384517176	0,314	0,154	1
CD59	5,82E-05	0,386651832	0,479	0,303	1
CEL	6,12E-05	0,705753639	0,579	0,429	1
ITGB1	6,41E-05	0,379036257	0,629	0,474	1
ABI1	6,53E-05	0,367414545	0,357	0,197	1
LSM4	6,60E-05	0,508223485	0,414	0,246	1
GPX3	7,02E-05	0,41453547	0,286	0,139	1
OLR1	7,16E-05	0,441696937	0,343	0,184	1
RAC1	7,50E-05	0,264001875	0,864	0,784	1
LRP1	8,03E-05	0,467524972	0,571	0,395	1
PGK1	8,10E-05	0,371690042	0,686	0,538	1
TIMM8B	8,24E-05	0,354649099	0,293	0,15	1
ADAM8	8,51E-05	0,352768587	0,343	0,19	1
MAPKAP1	8,62E-05	0,299290698	0,271	0,128	1
ECHS1	8,99E-05	0,326245814	0,314	0,16	1
ATP6V0B	9,22E-05	0,351788801	0,721	0,598	1
EIF3J	9,61E-05	0,337436437	0,336	0,179	1
CLIC1	9,70E-05	0,346299587	0,707	0,609	1
GPR137B	0,000105443	0,377183343	0,279	0,137	1
COMT	0,00010876	0,301772517	0,393	0,231	1
CREG1	0,000110475	0,252338295	0,579	0,382	1

CCT5	0,00011269	0,395353965	0,386	0,222	1
SMS	0,000114515	0,435255145	0,407	0,244	1
DARS	0,000121562	0,386590805	0,471	0,303	1
RAD23A	0,000138848	0,289598614	0,536	0,355	1
ABHD12	0,000143651	0,36151199	0,4	0,226	1
SORT1	0,000154852	0,349738679	0,321	0,171	1
IL1RN	0,000160881	0,923783441	0,271	0,137	1
LIMS1	0,000173269	0,362712433	0,507	0,346	1
P4HA1	0,000174873	0,328181891	0,286	0,141	1
HSP90AB1	0,000179077	0,323354731	0,779	0,641	1
ARHGAP18	0,000200418	0,345463969	0,421	0,25	1
ATP6V1A	0,000208406	0,292497459	0,536	0,348	1
ANKH	0,000209791	0,303910752	0,293	0,152	1
HNRNPAB	0,000211383	0,309839919	0,457	0,284	1
ZNHIT1	0,000221813	0,289882715	0,379	0,212	1
TMED9	0,000222053	0,312799681	0,414	0,254	1
STIP1	0,000240259	0,347578095	0,279	0,141	1
RNF145	0,000259928	0,396908323	0,529	0,372	1
ASPH	0,000266152	0,369624019	0,371	0,216	1
RDH11	0,000276923	0,256160067	0,3	0,154	1
NDUFB4	0,000291686	0,294159893	0,429	0,256	1
EIF5B	0,000299138	0,288486149	0,586	0,417	1
VCP	0,000306297	0,308841672	0,429	0,263	1
PPDPF	0,000340039	0,290526366	0,614	0,421	1
TREM1	0,00036956	0,387501965	0,3	0,162	1
RABAC1	0,000374895	0,437366844	0,336	0,194	1
MANF	0,000379758	0,273120968	0,271	0,139	1
GRN	0,000390791	0,297070379	0,936	0,84	1
YWHAE	0,000396141	0,291872574	0,564	0,406	1
HSPA9	0,000399879	0,253264964	0,4	0,237	1
PNP	0,000410835	0,285205135	0,271	0,141	1
BSG	0,000510088	0,254445395	0,45	0,288	1
AKR1A1	0,000511221	0,276026729	0,443	0,282	1
HSP90AA1	0,000518801	0,417139748	0,857	0,769	1
CERS2	0,000548973	0,324670385	0,321	0,182	1
TOMM40	0,000554862	0,295532071	0,257	0,135	1
LGMN	0,000605451	0,402977075	0,429	0,267	1
APP	0,000632929	0,289947286	0,286	0,152	1
ACTN4	0,000646505	0,293188233	0,643	0,506	1
UBALD2	0,00067069	0,28748841	0,371	0,224	1
AP2M1	0,000677286	0,273484233	0,55	0,387	1
CALM2	0,000779093	0,272833771	0,707	0,609	1
ATP6V1G1	0,000795209	0,337274729	0,457	0,312	1
DMXL2	0,000810354	0,330937253	0,443	0,284	1
PDLIM5	0,000814481	0,34032418	0,436	0,269	1
ZNF385A	0,000861733	0,260063469	0,55	0,387	1
NFE2L1	0,000867217	0,251396796	0,286	0,156	1
LAMP1	0,000914342	0,327221602	0,564	0,421	1
EHD4	0,001030262	0,254924938	0,257	0,137	1
SRRM1	0,001067796	0,307842199	0,493	0,335	1
DUT	0,001069351	0,311260974	0,371	0,229	1
TCIRG1	0,00111728	0,262285371	0,536	0,353	1
CAPZA2	0,00113103	0,261954045	0,529	0,359	1
SLC11A1	0,001141403	0,301642577	0,407	0,248	1
YTHDF2	0,00118274	0,346567384	0,343	0,212	1
KHSRP	0,001209949	0,305226183	0,493	0,327	1

GHITM	0,001220251	0,30640036	0,436	0,28	1
NDUFB2	0,001236724	0,25567189	0,457	0,299	1
IRF2BP2	0,001264048	0,309979025	0,414	0,267	1
SOD1	0,001538186	0,319088604	0,471	0,321	1
CHCHD10	0,00155628	0,327247486	0,414	0,276	1
CD84	0,001568877	0,270172753	0,664	0,524	1
IL18	0,001710695	0,291062614	0,357	0,224	1
CPM	0,001885783	0,329693026	0,479	0,325	1
EIF4G2	0,001915739	0,26197753	0,729	0,632	1
ACTR1A	0,002062241	0,401229929	0,3	0,177	1
SDF4	0,002260645	0,271701387	0,321	0,194	1
H2AFV	0,002325111	0,360757291	0,393	0,265	1
GAA	0,002403151	0,272900296	0,429	0,286	1
ISCU	0,002415696	0,265570681	0,407	0,263	1
PLIN3	0,002557346	0,30901521	0,479	0,329	1
KIAA2013	0,002650874	0,254338413	0,264	0,15	1
PSMC6	0,002812836	0,300697778	0,257	0,141	1
NUP62	0,002911143	0,252404816	0,45	0,31	1
EIF4G1	0,002969831	0,274662181	0,3	0,177	1
SGK1	0,003107525	0,286405459	0,471	0,338	1
AUP1	0,003143102	0,279482854	0,421	0,286	1
NUCKS1	0,003557948	0,348011072	0,693	0,547	1
ITGB2	0,003605106	0,294974079	0,743	0,673	1
RTL8C	0,003712256	0,284928707	0,293	0,182	1
MTCH2	0,003833852	0,313126718	0,271	0,16	1
NDUFB8	0,004074123	0,253413417	0,343	0,222	1
ME2	0,004550269	0,271072816	0,279	0,167	1
IFI30	0,004591219	0,269084075	0,929	0,885	1
NDUFV2	0,004597386	0,258167588	0,329	0,214	1
GNPTAB	0,004605535	0,343490522	0,321	0,197	1
CCT2	0,004613319	0,293626105	0,329	0,218	1
CD81	0,004845378	0,259968713	0,679	0,571	1
CCNL2	0,00522013	0,322922377	0,279	0,169	1
RASA4	0,005225904	0,374966664	0,329	0,212	1
PA2G4	0,005410347	0,381935745	0,329	0,22	1
STX4	0,005501743	0,376417981	0,257	0,154	1
NCL	0,006073011	0,391739725	0,457	0,331	1
SDC3	0,006316563	0,311188411	0,321	0,205	1
KIAA0930	0,006554426	0,2956328	0,45	0,325	1
ATP13A3	0,006740894	0,272768483	0,3	0,194	1
NEMF	0,006802972	0,280645125	0,393	0,274	1
LARP1	0,006871234	0,254244934	0,571	0,444	1
GLUL	0,007463685	0,264407621	0,764	0,643	1
MFSD1	0,00758239	0,343403847	0,507	0,389	1
H2AFZ	0,007920195	0,279623199	0,4	0,276	1
CCL2	0,00800528	0,605760942	0,3	0,201	1
SEC61A1	0,008449296	0,265407508	0,35	0,229	1
ARHGDIA	0,008616651	0,297877326	0,657	0,543	1
RAN	0,008933646	0,266309319	0,414	0,297	1
HSP90B1	0,009292168	0,621266194	0,614	0,504	1
GM2A	0,009629246	0,404139243	0,436	0,312	1

Cluster My.3 marker genes

gene	p_val	avg_logFC	pct.1	pct.2	p_val_adj
FCER1A	3,47E-56	2,394814932	0,576	0,027	6,58E-52
CLEC10A	9,67E-44	1,843483228	0,717	0,116	1,83E-39
HLA-DPB1	7,07E-38	1,301825205	1	0,787	1,34E-33
CD1C	4,91E-36	2,013468762	0,478	0,047	9,31E-32
HLA-DPA1	1,78E-35	1,131981199	1	0,895	3,38E-31
HLA-DRA	1,08E-32	1,029765422	1	0,93	2,05E-28
NAPSB	9,33E-32	1,323335573	0,522	0,07	1,77E-27
HLA-DRB1	2,96E-31	0,94596974	1	0,915	5,61E-27
CD74	1,63E-29	0,830412657	1	0,992	3,10E-25
HLA-DQA1	1,37E-28	1,288256498	0,967	0,593	2,60E-24
CD1E	6,12E-28	1,218215794	0,261	0,006	1,16E-23
GNAO1	1,02E-27	1,193494749	0,978	0,649	1,93E-23
CPVL	8,72E-27	1,309261084	0,87	0,376	1,65E-22
HLA-DQA2	2,33E-22	1,291038609	0,576	0,155	4,41E-18
CST3	4,49E-20	1,013200161	1	0,886	8,51E-16
FCGR2B	7,65E-19	1,111436039	0,565	0,165	1,45E-14
PPA1	8,72E-19	0,769667348	0,489	0,116	1,65E-14
EEF1A1	1,41E-16	0,513522437	1	0,969	2,68E-12
HLA-DQB2	1,92E-16	1,065457855	0,478	0,132	3,63E-12
HLA-DRB6	4,39E-16	0,952241987	0,87	0,504	8,32E-12
NAP1L1	2,13E-14	0,636795519	0,935	0,601	4,03E-10
NDRG2	2,50E-14	0,642182349	0,272	0,043	4,73E-10
IL1R2	4,56E-14	0,441714455	0,272	0,041	8,63E-10
FGL2	1,35E-13	0,689193894	0,859	0,467	2,56E-09
HLA-DMA	2,51E-13	0,613456565	0,902	0,517	4,75E-09
PLD4	3,65E-13	0,733519532	0,293	0,056	6,92E-09
JAML	5,50E-13	0,746362154	0,652	0,293	1,04E-08
HLA-DMB	7,41E-13	0,671332506	0,859	0,502	1,40E-08
LYZ	9,01E-13	0,761700837	0,946	0,762	1,71E-08
COTL1	1,31E-12	0,555278175	0,957	0,694	2,48E-08
FAU	2,15E-12	0,56800433	0,913	0,781	4,08E-08
TPT1	3,10E-12	0,552739251	1	0,917	5,87E-08
SLC38A1	3,89E-12	0,694157191	0,304	0,068	7,37E-08
EEF1B2	4,12E-12	0,656828724	0,88	0,595	7,81E-08
TMSB4X	1,45E-11	0,487905766	1	0,975	2,75E-07
SPINT2	1,58E-11	0,781132119	0,685	0,357	3,00E-07
ACTG1	3,01E-11	0,594745827	0,989	0,874	5,71E-07
HINT1	1,48E-10	0,617662615	0,652	0,32	2,81E-06
KLF5	1,51E-10	0,582680654	0,609	0,281	2,86E-06
AXL	1,93E-10	0,543557888	0,293	0,072	3,65E-06
EEF1G	3,26E-10	0,50854433	0,913	0,696	6,18E-06
PABPC1	3,37E-10	0,446962051	0,978	0,864	6,39E-06
PON2	3,49E-10	0,527407915	0,261	0,06	6,61E-06
COMMD6	3,72E-10	0,522508053	0,609	0,273	7,05E-06
LGALS2	6,20E-10	0,50764554	0,391	0,124	1,17E-05
PPP1CB	7,51E-10	0,636313329	0,739	0,411	1,42E-05
sep-06	7,59E-10	0,581579241	0,554	0,246	1,44E-05
EDEM1	1,10E-09	0,561583584	0,402	0,143	2,09E-05
TMSB10	1,57E-09	0,365149157	0,989	0,942	2,97E-05
CLIC2	1,88E-09	0,544450423	0,304	0,091	3,57E-05
CBX6	2,45E-09	0,554054979	0,467	0,19	4,64E-05
PAK1	2,76E-09	0,49470413	0,424	0,157	5,23E-05
HLA-DOA	8,34E-09	0,477008211	0,522	0,229	0,000158116

NR4A3	8,68E-09	0,569656836	0,261	0,068	0,000164562
GRASP	9,84E-09	0,498477666	0,283	0,078	0,000186454
STK17B	1,57E-08	0,460608237	0,685	0,364	0,000298242
CX3CR1	1,94E-08	0,521933217	0,38	0,132	0,000367246
CDK2AP1	2,68E-08	0,58004377	0,489	0,223	0,000507488
BASP1	3,88E-08	0,511065038	0,554	0,271	0,000734559
HSPA8	5,77E-08	0,508249489	0,804	0,552	0,001092655
DENND1B	8,46E-08	0,543324734	0,359	0,138	0,001602686
USB1	1,00E-07	0,439691236	0,304	0,105	0,001897807
MS4A6A	1,16E-07	0,542316649	0,772	0,537	0,002204602
NUP210	1,19E-07	0,337369355	0,261	0,078	0,002258069
GDI2	1,21E-07	0,511341869	0,739	0,455	0,002289195
TBC1D9	1,32E-07	0,551104481	0,304	0,107	0,002499972
TAP1	1,48E-07	0,340381338	0,326	0,114	0,002810112
NOP53	1,70E-07	0,355014016	0,75	0,436	0,003212272
GOLGA8S	2,50E-07	0,553827151	0,304	0,109	0,004733145
SESN3	2,84E-07	0,445455958	0,315	0,11	0,005391209
ELOVL5	4,73E-07	0,428284862	0,315	0,112	0,008969177
ADAM8	4,92E-07	0,413125206	0,435	0,188	0,009323683
SARAF	5,19E-07	0,423067345	0,707	0,417	0,009830289
CD1D	5,26E-07	0,448445389	0,261	0,085	0,009973423
TUBA1A	5,37E-07	0,437992831	0,728	0,43	0,010179841
TSPAN33	5,78E-07	0,632048396	0,326	0,13	0,01094963
SUB1	5,78E-07	0,466692676	0,587	0,335	0,010953558
RCC2	6,45E-07	0,396136013	0,478	0,236	0,012218042
HNRNPA1	6,56E-07	0,417818816	0,685	0,422	0,012430072
CHCHD7	7,00E-07	0,414888293	0,283	0,099	0,013266483
UVRAG	7,07E-07	0,444019734	0,315	0,12	0,013389665
CAT	7,33E-07	0,434996586	0,511	0,262	0,013896116
SATB1	7,35E-07	0,592749815	0,478	0,221	0,013933717
EIF3F	8,03E-07	0,478194803	0,543	0,298	0,015216823
OSTC	8,17E-07	0,454159148	0,402	0,178	0,015487116
FAM129A	8,45E-07	0,515383876	0,598	0,343	0,016016946
GPR183	1,17E-06	0,373624779	0,511	0,24	0,022222553
DOCK2	1,19E-06	0,378431601	0,489	0,238	0,022570131
CTSH	1,31E-06	0,455742438	0,772	0,479	0,024787716
SLAMF7	1,55E-06	0,549438444	0,283	0,099	0,029454342
GSTP1	1,65E-06	0,473927521	0,75	0,469	0,031242376
RACK1	1,90E-06	0,317630927	0,935	0,775	0,035973281
RAB7A	1,93E-06	0,357751453	0,707	0,419	0,036660825
SLC25A5	2,07E-06	0,471346877	0,652	0,382	0,039256766
SEC61B	2,08E-06	0,55980922	0,489	0,26	0,039431269
EIF3L	2,14E-06	0,330716907	0,739	0,465	0,04045825
LIMD2	2,38E-06	0,296645566	0,511	0,252	0,045177912
HNRNPA2B1	3,05E-06	0,353448855	0,859	0,593	0,057817894
TOMM7	3,16E-06	0,438538539	0,696	0,434	0,059829346
KDM2B	3,44E-06	0,371549259	0,293	0,112	0,065246445
RTN1	3,49E-06	0,503003929	0,348	0,153	0,066048385
NACA	4,05E-06	0,327679072	0,837	0,543	0,076712749
PARL	4,22E-06	0,393056917	0,391	0,176	0,079958317
SNX3	4,27E-06	0,72669676	0,478	0,266	0,08093798
AP3D1	4,28E-06	0,466104289	0,467	0,24	0,081092877
PLSCR1	4,39E-06	0,391804373	0,413	0,196	0,083199362
RGS10	4,64E-06	0,385092669	0,565	0,326	0,087884946
PEA15	4,75E-06	0,478513463	0,75	0,529	0,090045278
GCA	5,19E-06	0,331693938	0,435	0,207	0,098297376

WDFY4	5,30E-06	0,662819198	0,293	0,12	0,100420088
MNDA	6,32E-06	0,272205948	0,62	0,347	0,119704586
ITGB2-AS1	6,39E-06	0,431764971	0,315	0,13	0,121081568
PID1	6,46E-06	0,374079438	0,261	0,095	0,122366834
PCBP2	7,38E-06	0,306090629	0,848	0,589	0,139820268
BRK1	7,50E-06	0,372130568	0,62	0,39	0,142100435
PPT1	7,76E-06	0,406568297	0,793	0,533	0,147002358
MYD88	8,18E-06	0,39060942	0,467	0,238	0,154960507
BTF3	8,20E-06	0,40965026	0,739	0,49	0,155297109
PTMA	8,46E-06	0,301030272	0,989	0,905	0,16039482
LDHB	8,90E-06	0,411368996	0,391	0,19	0,168715611
MAP3K13	9,54E-06	0,408355658	0,37	0,167	0,180817934
FOXN2	9,75E-06	0,4664053	0,283	0,116	0,184761198
SSR1	1,05E-05	0,30943	0,696	0,428	0,198789525
UQCRH	1,07E-05	0,380973406	0,38	0,18	0,202938729
YBX1	1,33E-05	0,34305426	0,88	0,638	0,252093324
AHR	1,47E-05	0,506507682	0,478	0,273	0,279504794
COX7C	1,57E-05	0,297356385	0,815	0,55	0,297875508
TUBA1B	1,64E-05	0,513777943	0,87	0,641	0,310792675
CMTM6	1,68E-05	0,32780667	0,837	0,583	0,318981428
BLOC1S6	1,75E-05	0,350812919	0,457	0,242	0,332233196
UBA52	1,86E-05	0,275585508	0,957	0,855	0,351565007
CAMTA1	1,86E-05	0,381845046	0,38	0,182	0,353374869
LSP1	2,12E-05	0,349183364	0,75	0,525	0,401316197
C1orf162	2,36E-05	0,371979476	0,641	0,432	0,447602973
HLA-DRB5	2,38E-05	0,897012025	0,598	0,378	0,45182963
PAIP2	2,48E-05	0,277556872	0,467	0,236	0,469175777
HMGN4	2,50E-05	0,310965253	0,326	0,143	0,474440774
APEX1	2,59E-05	0,37247926	0,435	0,215	0,491418814
RBPJ	2,60E-05	0,35157258	0,772	0,527	0,492246392
NAGA	2,64E-05	0,275204188	0,511	0,281	0,49981546
ATP5F1E	2,67E-05	0,326036949	0,272	0,112	0,506092107
SMDT1	2,70E-05	0,381907254	0,348	0,165	0,511249144
ALCAM	2,73E-05	0,281395407	0,478	0,25	0,517332173
MBNL1	3,04E-05	0,295464614	0,717	0,465	0,576271616
SFPQ	3,19E-05	0,26961301	0,489	0,258	0,604696268
UBE2J1	3,38E-05	0,31486549	0,37	0,174	0,640084371
PRPF19	3,72E-05	0,388093898	0,272	0,116	0,705034944
ALDH2	3,98E-05	0,523247436	0,511	0,3	0,754551564
INSIG1	4,00E-05	0,546801549	0,293	0,13	0,757981502
ETV6	4,33E-05	0,444074319	0,522	0,31	0,821316739
SERPINF1	4,35E-05	0,512357503	0,348	0,165	0,824487827
GAPT	4,57E-05	0,401491776	0,272	0,112	0,865230451
PHB2	4,71E-05	0,397228994	0,489	0,281	0,892037392
LCP1	4,72E-05	0,312225879	0,978	0,853	0,893674994
SEMA4A	4,91E-05	0,323506329	0,326	0,147	0,930004101
PARVG	5,25E-05	0,360793072	0,478	0,269	0,995482687
SNHG5	5,42E-05	0,334447371	0,652	0,43	1
CNN2	5,46E-05	0,34711579	0,62	0,391	1
CLEC7A	5,55E-05	0,311388119	0,685	0,436	1
DYNC1LI1	5,76E-05	0,480672446	0,304	0,14	1
MICAL1	5,91E-05	0,31387657	0,359	0,174	1
MSL3	5,96E-05	0,305095815	0,304	0,138	1
HNRNPH1	6,13E-05	0,328639466	0,717	0,498	1
IGFLR1	6,49E-05	0,443661879	0,293	0,14	1
CSF2RB	6,89E-05	0,326209018	0,413	0,213	1

PTPN2	7,21E-05	0,299018329	0,478	0,271	1
IFNGR1	8,31E-05	0,583165413	0,533	0,339	1
MRPL3	8,43E-05	0,292716601	0,272	0,116	1
PTBP3	8,86E-05	0,346795134	0,565	0,364	1
SEC13	0,00010268	0,275357128	0,359	0,176	1
NDUFB1	0,000108186	0,364376475	0,467	0,273	1
CD48	0,00011066	0,285018575	0,587	0,359	1
PPIA	0,000113205	0,346784813	0,924	0,81	1
SELENOK	0,000114359	0,280638868	0,272	0,122	1
AKNA	0,00011629	0,313193644	0,457	0,256	1
DAZAP2	0,000116687	0,26730968	0,793	0,56	1
ATP5F1EP2	0,000120261	0,289699444	0,75	0,502	1
DUSP3	0,00012321	0,312147721	0,5	0,293	1
AMPD3	0,000125996	0,378624906	0,326	0,159	1
IL13RA1	0,000139976	0,537586829	0,533	0,347	1
EIF3E	0,00014069	0,302385617	0,63	0,411	1
SUMO3	0,000143805	0,32644289	0,478	0,262	1
RAP1GDS1	0,00015032	0,342432532	0,283	0,132	1
DEFA6	0,0001606	0,346384748	0,348	0,174	1
ATP5MC2	0,000163224	0,321516892	0,804	0,605	1
LSM7	0,000166378	0,27942848	0,272	0,122	1
ARF5	0,000172511	0,399859077	0,457	0,269	1
HMGN2	0,000180052	0,401681338	0,413	0,231	1
EIF6	0,000180074	0,255447488	0,609	0,36	1
NPM3	0,000182785	0,281002597	0,576	0,357	1
BICD2	0,000185954	0,315514205	0,38	0,198	1
ALKBH7	0,000202954	0,371892329	0,272	0,124	1
PTTG1IP	0,000203873	0,263307604	0,609	0,388	1
HADHB	0,000205204	0,296370165	0,435	0,246	1
GAS5	0,000217059	0,345630549	0,359	0,186	1
COX7B	0,000224775	0,282747107	0,62	0,386	1
PSMD13	0,000231231	0,297940124	0,304	0,147	1
CCNI	0,000237856	0,298903668	0,707	0,479	1
SF3B5	0,000240198	0,257914122	0,478	0,269	1
ETFA	0,000244336	0,267943543	0,293	0,143	1
DPYSL2	0,000255666	0,317463039	0,674	0,417	1
CDC42SE2	0,000268156	0,341706403	0,38	0,211	1
KCNK6	0,00027238	0,392597956	0,435	0,242	1
PTAR1	0,000272742	0,273505967	0,283	0,13	1
HMGN1	0,000279571	0,268775334	0,576	0,355	1
NDUFB2	0,000280295	0,290910157	0,489	0,308	1
LY86	0,000283995	0,331554166	0,511	0,314	1
APPL1	0,000291346	0,3120811	0,348	0,178	1
C1orf54	0,00030211	0,584739476	0,293	0,155	1
STK38L	0,000342996	0,325881954	0,283	0,138	1
HMGN3	0,000347825	0,300382189	0,359	0,192	1
DSE	0,000350713	0,314757052	0,413	0,231	1
EIF3K	0,000371968	0,317907442	0,554	0,374	1
N4BP2	0,000372641	0,393202228	0,261	0,122	1
ATP5F1C	0,000378792	0,391439783	0,478	0,308	1
EIF4H	0,000384428	0,350811124	0,63	0,434	1
CTDSP2	0,000387699	0,253313721	0,38	0,211	1
TNFSF13B	0,00038858	0,311055533	0,598	0,382	1
HMGA1	0,000389184	0,271783428	0,511	0,32	1
SPTLC2	0,000389446	0,410090761	0,37	0,2	1
ANKRD44	0,00042096	0,375604634	0,402	0,223	1

PRPF8	0,000426229	0,282393643	0,413	0,229	1
RAB8A	0,000440268	0,304118679	0,424	0,25	1
RPS27A	0,000468809	0,29527465	0,891	0,729	1
SNAP23	0,000478007	0,256835784	0,413	0,227	1
MPEG1	0,00050807	0,425187364	0,696	0,5	1
HNRNPAB	0,000516004	0,344295381	0,478	0,297	1
COX5A	0,000533325	0,34782679	0,446	0,269	1
ATP5F1A	0,000541245	0,255978371	0,554	0,362	1
ARF6	0,000553619	0,393595489	0,489	0,331	1
ARPP19	0,000594365	0,273052536	0,435	0,258	1
CASP1	0,000597949	0,26844852	0,413	0,234	1
LITAF	0,000610608	0,277880437	0,815	0,61	1
NR3C1	0,000613828	0,44060742	0,522	0,339	1
ADPGK	0,000633025	0,27064499	0,337	0,184	1
CD86	0,000636753	0,318156875	0,478	0,302	1
LST1	0,000659617	0,358137708	0,62	0,444	1
NFKB1	0,000693326	0,343461884	0,391	0,229	1
VASH1	0,000704354	0,392555141	0,348	0,196	1
KLF4	0,000737666	0,331373408	0,435	0,269	1
CPNE3	0,000744298	0,564148506	0,326	0,18	1
RASSF5	0,000763705	0,255147433	0,522	0,32	1
FNBP1	0,000767913	0,302360362	0,565	0,364	1
IRF8	0,000790332	0,584575186	0,293	0,167	1
DDX21	0,000811828	0,363959033	0,587	0,397	1
ARPC3	0,000831484	0,28638894	0,739	0,589	1
HIST1H4C	0,000835398	0,254032534	0,283	0,141	1
DDX5	0,00091609	0,304859695	0,783	0,61	1
SDHC	0,000929658	0,319350405	0,304	0,163	1
mrt-01	0,000936718	0,366577176	0,478	0,314	1
UBB	0,000973246	0,276297899	0,739	0,566	1
MYL12A	0,000980202	0,315627275	0,75	0,521	1
HSPA7	0,001006322	0,507010544	0,261	0,136	1
SNHG6	0,00101127	0,256141485	0,446	0,277	1
ADAR	0,001117676	0,265072696	0,478	0,295	1
RFX5	0,001138163	0,293925875	0,261	0,13	1
RNF144B	0,001180319	0,346972659	0,304	0,161	1
SLC9A7	0,001198075	0,306618934	0,283	0,149	1
SERPINB9	0,001279036	0,5922063	0,402	0,248	1
ATP5MD	0,001292954	0,290443145	0,478	0,304	1
ACAA1	0,001329635	0,32207626	0,272	0,143	1
MTCH2	0,001367695	0,268272652	0,304	0,165	1
GABARAPL2	0,001372827	0,41856107	0,315	0,176	1
PITPNA	0,001440673	0,270077452	0,337	0,19	1
H2AFZ	0,001449913	0,356116092	0,457	0,277	1
FLOT2	0,001472801	0,277031377	0,326	0,178	1
ATP5MG	0,001481944	0,353822064	0,533	0,384	1
SCRN1	0,001482767	0,300298004	0,261	0,132	1
RNASE6	0,001488644	0,346965027	0,522	0,343	1
PABPC4	0,001509534	0,296143613	0,717	0,566	1
CBL	0,00151044	0,382850971	0,304	0,174	1
SF3B4	0,001529374	0,269526632	0,478	0,295	1
VOPP1	0,001534368	0,318808863	0,554	0,357	1
EVI2A	0,001537241	0,293683633	0,522	0,345	1
PPP1R12A	0,001573554	0,255899548	0,478	0,297	1
MRPL20	0,001633313	0,30183652	0,272	0,14	1
RAN	0,001677032	0,271365138	0,467	0,298	1

EIF4EBP2	0,001691021	0,281623014	0,446	0,281	1
PRKAR1A	0,001770327	0,254401305	0,62	0,43	1
CCND2	0,0017709	0,25793089	0,293	0,155	1
ATP5F1D	0,001834503	0,29383739	0,348	0,205	1
POLR1D	0,001894845	0,392283687	0,315	0,184	1
TAB2	0,001936231	0,352399703	0,283	0,151	1
ZFR	0,002030026	0,260234594	0,315	0,174	1
PSMA1	0,002217926	0,300301668	0,391	0,25	1
PSMB2	0,002398507	0,317098825	0,402	0,246	1
NDUFA4	0,002449496	0,291987355	0,522	0,37	1
TMEM14B	0,002467091	0,254835997	0,304	0,167	1
SPCS3	0,002792561	0,31629994	0,424	0,285	1
MIS18BP1	0,00284047	0,299814145	0,348	0,209	1
HNRNPA3	0,002919209	0,260918762	0,565	0,391	1
NDUFA7	0,002958614	0,263936274	0,261	0,141	1
TBCA	0,00298504	0,260940522	0,424	0,269	1
CUX1	0,00317556	0,294591674	0,467	0,293	1
CLINT1	0,003179513	0,362067436	0,283	0,167	1
PACS1	0,003372459	0,296845195	0,261	0,141	1
MLXIP	0,003836544	0,277363962	0,272	0,151	1
ENY2	0,003841254	0,2821036	0,304	0,18	1
ANP32B	0,00385626	0,343012178	0,533	0,374	1
H2AFY	0,003976795	0,302996395	0,717	0,576	1
TNKS2	0,004095187	0,257231709	0,337	0,205	1
MYL12B	0,004279744	0,2542726	0,674	0,502	1
CIRBP	0,004455708	0,279943026	0,576	0,434	1
EIF3H	0,004613853	0,264645336	0,533	0,372	1
SUPT4H1	0,004679023	0,390598739	0,348	0,219	1
SEC11A	0,004794711	0,265681225	0,489	0,335	1
RUNX3	0,00483551	0,274687669	0,37	0,229	1
PDIA3	0,004905828	0,30190826	0,391	0,252	1
SLC8A1	0,005142437	0,254789637	0,261	0,147	1
NDUFV2	0,00514334	0,259969419	0,348	0,221	1
TRIM8	0,005988749	0,335501793	0,304	0,184	1
COX6A1	0,006115344	0,302692664	0,609	0,426	1
TMEM14C	0,006432506	0,362825114	0,337	0,221	1
AMPD2	0,006522654	0,287894875	0,272	0,153	1
SNRPG	0,006637804	0,28072291	0,326	0,2	1
TOMM20	0,007125317	0,255810425	0,424	0,277	1
CDKN1A	0,007196685	0,351437415	0,424	0,291	1
ATP5MC3	0,007520489	0,268387323	0,457	0,333	1
TMEM230	0,008370505	0,318665986	0,337	0,213	1
MTHFD2	0,008634686	0,264698081	0,315	0,186	1
RAP1A	0,009653044	0,271514334	0,511	0,386	1

Cluster My.4 marker genes

gene	p_val	avg_logFC	pct.1	pct.2	p_val_adj
IL1RL1	2,74E-23	1,790113784	0,562	0,066	5,19E-19
GNLY	1,01E-18	1,863100839	0,844	0,276	1,91E-14
PTK2B	2,06E-16	1,781782057	0,906	0,429	3,90E-12
SPOCK2	2,22E-16	1,562439785	0,656	0,153	4,21E-12
PLSCR4	5,71E-14	0,960635646	0,312	0,031	1,08E-09
TSPYL2	6,95E-13	1,477603093	0,562	0,134	1,32E-08
MGP	8,36E-13	2,083656533	0,906	0,589	1,58E-08
IFITM2	8,59E-13	1,208911682	1	0,859	1,63E-08
PTPRCAP	9,60E-13	1,47989624	0,719	0,245	1,82E-08
PLAT	2,32E-12	1,606739327	0,344	0,047	4,40E-08
C11orf96	3,49E-12	1,211916373	0,312	0,038	6,60E-08
IL32	4,14E-12	1,820949355	0,625	0,198	7,84E-08
VAMP2	6,84E-12	1,291434605	0,875	0,502	1,30E-07
CD3E	1,21E-11	1,609795542	0,656	0,219	2,29E-07
GZMA	1,26E-11	1,305631318	0,438	0,082	2,38E-07
CD3D	2,62E-10	1,217006655	0,312	0,047	4,97E-06
IL2RG	3,41E-10	1,435732267	0,844	0,528	6,46E-06
CFH	3,89E-10	1,306888874	0,312	0,049	7,38E-06
EIF4B	5,29E-10	1,105051156	0,906	0,613	1,00E-05
CALD1	1,34E-09	1,10742311	0,406	0,087	2,53E-05
CFL1	2,38E-09	0,815855289	1	0,934	4,52E-05
PTMA	5,24E-09	0,834065907	0,969	0,915	9,94E-05
LCK	6,23E-09	0,968825566	0,312	0,056	0,000117975
GZMK	9,92E-09	0,958317366	0,375	0,08	0,000188009
GPS2	1,01E-08	1,449811427	0,688	0,323	0,000192266
PFN1	1,25E-08	0,725952111	0,969	0,924	0,000237266
TRMT112	2,63E-08	1,144799146	0,719	0,387	0,000499069
IGFBP4	2,70E-08	1,284772082	0,469	0,141	0,000511341
IGFBP7	2,96E-08	1,526545558	0,75	0,424	0,000560818
MICAL2	4,78E-08	1,21397675	0,531	0,186	0,000905138
APEX2	6,99E-08	1,081398559	0,375	0,09	0,001324182
FCMR	1,11E-07	1,239293621	0,281	0,054	0,002104029
SELL	4,83E-07	0,985373578	0,594	0,255	0,009143836
TARP	5,08E-07	0,97780352	0,375	0,102	0,009620651
CSRP1	5,57E-07	1,115322418	0,562	0,245	0,010550093
ZNF275	5,60E-07	1,312220554	0,344	0,087	0,010604467
HSPB1	7,10E-07	0,907264578	0,781	0,547	0,013445046
ETS1	8,69E-07	0,968476742	0,406	0,125	0,016467312
EIF4G2	1,02E-06	0,928528627	0,844	0,644	0,019316096
IFITM1	1,33E-06	1,273799025	0,562	0,26	0,025157735
SH3BGRL3	1,43E-06	0,751640796	0,969	0,925	0,027048434
PKM	1,60E-06	0,571977367	0,969	0,8	0,030267776
PDLIM1	1,62E-06	1,427487565	0,531	0,233	0,030656203
PNPLA2	1,62E-06	1,100002494	0,594	0,297	0,030694416
IGFBP5	1,79E-06	1,082729307	0,438	0,155	0,033859409
JUND	2,27E-06	0,768272849	0,875	0,793	0,04303942
MYH9	2,54E-06	0,730721477	0,812	0,622	0,048060695
MS4A1	2,61E-06	0,885350552	0,344	0,094	0,049551576
PPIA	2,62E-06	0,81797576	0,938	0,821	0,049675698
IL7R	6,82E-06	0,997817789	0,406	0,137	0,129320259
SKI	7,66E-06	1,170818307	0,562	0,29	0,145065195
ACKR1	7,76E-06	0,821706257	0,281	0,069	0,14704987
CD81	1,30E-05	0,744563524	0,812	0,583	0,246866423

ARHGDIB	1,60E-05	0,600807778	0,906	0,828	0,303142346
HNRNPC	2,16E-05	0,738772021	0,781	0,648	0,408760725
TIMP1	2,26E-05	0,398170818	0,875	0,731	0,428478145
THBS1	2,30E-05	0,819399634	0,375	0,123	0,436462645
CLU	2,40E-05	1,179258227	0,5	0,233	0,454136807
FTO	3,01E-05	0,85968348	0,406	0,151	0,571232335
FCN1	3,92E-05	0,651706988	0,719	0,389	0,742829424
SPINT2	5,09E-05	0,793284027	0,656	0,392	0,963613258
SERF2	6,72E-05	0,627985908	0,938	0,837	1
PITPNC1	0,000102275	0,90837853	0,375	0,144	1
CLDND1	0,000103186	0,89467677	0,5	0,257	1
HLA-A	0,000107368	0,494776642	0,938	0,872	1
YPEL3	0,000110611	0,786379349	0,625	0,396	1
CCL5	0,000160348	1,181339584	0,469	0,233	1
CPNE1	0,000174449	0,732336034	0,562	0,316	1
NKG7	0,000180441	0,872498947	0,344	0,132	1
CRTAP	0,000203708	0,743069254	0,688	0,531	1
HNRNPUL1	0,000216463	1,004306577	0,625	0,465	1
ALOX5AP	0,000254777	0,865365509	0,562	0,359	1
IMPAD1	0,000284661	0,929942289	0,438	0,215	1
AHNAK	0,000286155	0,523051487	0,812	0,712	1
YWHAZ	0,000296439	0,398118197	0,906	0,736	1
TRIR	0,000317891	0,727241037	0,656	0,483	1
NFKBIB	0,000335435	0,98675131	0,312	0,12	1
GIGYF1	0,000442306	0,92155517	0,406	0,198	1
HECTD3	0,000565959	0,736026934	0,281	0,101	1
ITM2B	0,000576868	0,554211488	0,938	0,878	1
FKBP1A	0,000662748	0,518252307	0,781	0,701	1
FAM192A	0,000686192	0,888494923	0,438	0,234	1
GNAS	0,000709347	0,380982793	0,938	0,885	1
SLC29A1	0,00079052	1,170489595	0,312	0,134	1
COX4I1	0,000839026	0,482749652	0,812	0,679	1
CAPNS1	0,001016194	0,697968911	0,719	0,576	1
RAB12	0,001136786	0,862024485	0,969	0,852	1
ARFIP1	0,001206858	0,814025778	0,281	0,111	1
ITM2C	0,001224674	0,669663463	0,281	0,106	1
APLP2	0,001327695	0,562179084	0,75	0,679	1
NUCB1	0,001331865	0,597172814	0,594	0,415	1
TNPO2	0,001363873	0,70019672	0,344	0,156	1
SRSF2	0,00143968	0,820744551	0,562	0,399	1
CD37	0,001448115	0,638789074	0,75	0,637	1
IFI30	0,001458706	0,381333049	0,938	0,892	1
COL3A1	0,001483955	0,953380039	0,312	0,134	1
CCL4	0,00159331	0,278599234	0,594	0,352	1
RAB2B	0,001703942	0,865065835	0,312	0,139	1
SPARC	0,001792564	1,197882532	0,406	0,227	1
LATS1	0,001965539	0,601395242	0,281	0,116	1
TP53I11	0,002251954	0,718483436	0,312	0,137	1
PIN1	0,002357217	0,536872815	0,656	0,509	1
ACTB	0,002379679	0,253088082	1	0,998	1
CD2	0,002472545	0,800503647	0,312	0,134	1
ACTA2	0,002541279	0,826305899	0,438	0,245	1
CYB5B	0,002572302	0,769862773	0,344	0,172	1
SEMA4D	0,002582471	0,908413683	0,375	0,203	1
TRIM28	0,003208046	0,971198762	0,469	0,328	1
SETD1B	0,003348297	0,575309942	0,281	0,118	1

FAM89B	0,003414989	0,659304642	0,344	0,17	1
FOXO1	0,003432396	0,749758792	0,312	0,144	1
GABARAP	0,00348955	0,325382028	0,938	0,804	1
COL1A2	0,003832375	0,923164943	0,281	0,12	1
MLLT6	0,004768362	0,598526549	0,469	0,293	1
ARPC2	0,005071892	0,298047673	0,969	0,894	1
SELENOO	0,005380192	0,618629754	0,344	0,177	1
HSP90AB1	0,005395228	0,555958606	0,719	0,67	1
CMTM7	0,005467237	0,718844269	0,406	0,241	1
COX6A1	0,005533833	0,600311417	0,594	0,446	1
PATL1	0,00557135	0,820683471	0,312	0,155	1
SPTAN1	0,006287427	0,694754219	0,375	0,219	1
EPAS1	0,0065897	0,913026781	0,375	0,219	1
SUPT5H	0,007009898	0,839126215	0,375	0,224	1
FOXP1	0,007104761	0,62946601	0,562	0,431	1
GPCPD1	0,007333679	0,673525741	0,438	0,276	1
RING1	0,007634366	0,615797512	0,312	0,158	1
SRRT	0,007860898	0,768832395	0,312	0,163	1
OTUB1	0,008133341	0,555834517	0,406	0,24	1
ANXA5	0,008343265	0,335847292	0,781	0,701	1
CYLD	0,00837902	0,751466915	0,375	0,22	1
DMAC2	0,008641647	0,683255127	0,281	0,137	1
PCSK7	0,008849874	0,563491373	0,281	0,132	1
G0S2	0,009321001	0,439053346	0,281	0,127	1
IFITM3	0,009698883	0,467786707	0,781	0,715	1

Cluster E.0 marker genes

gene	p_val	avg_logFC	pct.1	pct.2	p_val_adj
DNASE1L3	6,69E-22	2,189639742	0,495	0,018	1,27E-17
ITGA6	9,70E-20	1,449180168	0,653	0,148	1,84E-15
PLVAP	4,02E-18	1,366512981	0,752	0,296	7,62E-14
EMCN	1,47E-17	1,302055776	0,703	0,237	2,79E-13
APLNR	6,39E-17	1,536127396	0,406	0,018	1,21E-12
ACKR1	7,48E-17	1,878277797	0,554	0,101	1,42E-12
VWA1	8,80E-17	1,42510912	0,574	0,13	1,67E-12
COL4A1	1,11E-16	1,344428124	0,743	0,29	2,10E-12
CD93	8,93E-16	1,184990137	0,693	0,237	1,69E-11
GNG11	1,79E-15	1,296370792	0,673	0,266	3,39E-11
SEMA6A	5,06E-15	1,261383248	0,376	0,024	9,59E-11
TM4SF18	8,19E-15	1,354044829	0,446	0,059	1,55E-10
HLA-B	1,35E-14	0,83644488	0,95	0,87	2,56E-10
RASA4	2,56E-14	1,447380591	0,525	0,124	4,85E-10
PRCP	4,53E-14	1,384316453	0,693	0,355	8,59E-10
COL15A1	6,59E-14	1,969504371	0,564	0,178	1,25E-09
ADGRL4	6,95E-14	1,202958854	0,703	0,361	1,32E-09
CEMIP2	9,55E-14	1,386432732	0,485	0,107	1,81E-09
MARCKS	1,37E-12	1,377460994	0,644	0,302	2,59E-08
OAF	1,75E-12	0,761770766	0,317	0,018	3,31E-08
RGCC	4,65E-12	1,068126554	0,356	0,041	8,82E-08
HLA-A	6,56E-12	0,766944331	0,891	0,846	1,24E-07
FLT4	9,28E-12	0,984864439	0,337	0,036	1,76E-07
MARCKSL1	2,18E-11	1,067934328	0,505	0,154	4,13E-07
SPNS2	4,28E-11	0,901754174	0,356	0,059	8,10E-07
GMFG	4,75E-11	0,704668351	0,327	0,036	9,01E-07
TSHZ2	9,80E-11	1,193365822	0,406	0,095	1,86E-06
GRB10	1,13E-10	1,02476864	0,515	0,183	2,13E-06
SHANK3	1,27E-10	1,042152475	0,683	0,361	2,40E-06
COTL1	1,34E-10	0,966768468	0,495	0,154	2,54E-06
ETS1	1,77E-10	1,06664637	0,634	0,32	3,35E-06
RGS16	2,08E-10	1,173634805	0,297	0,03	3,94E-06
COL4A2	2,92E-10	1,031968094	0,723	0,42	5,54E-06
RFLNB	3,18E-10	0,938315577	0,386	0,083	6,02E-06
KDR	3,26E-10	0,873557936	0,317	0,041	6,17E-06
NOSTRIN	3,53E-10	0,92559989	0,327	0,047	6,70E-06
DIPK2B	3,95E-10	0,851839159	0,515	0,183	7,49E-06
ADAMTS9	5,64E-10	0,992463108	0,356	0,071	1,07E-05
FABP5	5,87E-10	1,362388961	0,446	0,13	1,11E-05
B2M	1,11E-09	0,572462814	0,97	0,953	2,10E-05
DYSF	1,15E-09	0,785310592	0,317	0,047	2,17E-05
POSTN	1,17E-09	1,340726831	0,535	0,201	2,21E-05
TCF4	1,28E-09	0,743156218	0,851	0,71	2,42E-05
ADGRL2	1,29E-09	1,092142851	0,347	0,065	2,44E-05
PCDH12	2,52E-09	0,756011685	0,297	0,041	4,78E-05
PDE2A	3,33E-09	0,776259981	0,257	0,024	6,31E-05
LPAR6	2,04E-08	0,696274594	0,416	0,124	0,00038649
NELL1	3,04E-08	0,809439823	0,594	0,331	0,000576893
SPARCL1	3,87E-08	0,741371542	0,842	0,621	0,000733591
PLAT	4,77E-08	1,802132067	0,545	0,32	0,000903956
CLEC2B	1,20E-07	0,642930956	0,287	0,059	0,002282463
SPRY1	1,70E-07	0,858680411	0,416	0,142	0,003219644
MTRNR2L1	2,34E-07	0,786367849	0,802	0,604	0,004425763

A2M	3,55E-07	0,646427074	0,772	0,497	0,00672115
KANK3	4,07E-07	0,780047963	0,366	0,124	0,007717703
CD74	4,45E-07	0,575619364	0,931	0,864	0,008423722
CD109	5,06E-07	0,7012232	0,406	0,142	0,009593148
THSD7A	5,06E-07	0,668693343	0,317	0,089	0,009595196
S100A10	5,48E-07	0,598309656	0,842	0,71	0,010381542
HLA-DRB1	7,04E-07	0,669828721	0,772	0,538	0,0133383
SPARC	1,22E-06	0,528448589	0,891	0,704	0,023166431
LYN	1,26E-06	0,649355572	0,277	0,071	0,023785084
PXDN	1,95E-06	1,055922168	0,376	0,142	0,037028279
SHC1	2,24E-06	0,797562195	0,653	0,462	0,042539427
TSPAN18	2,63E-06	0,749773834	0,505	0,249	0,049809236
TMSB10	2,93E-06	0,420793722	0,95	0,911	0,055469142
HLA-DRA	4,02E-06	0,63728361	0,752	0,562	0,076160719
MAGI1	5,87E-06	0,676396108	0,327	0,112	0,111330501
MMRN1	6,66E-06	0,725777593	0,386	0,148	0,126238422
PTMA	7,35E-06	0,446859215	0,921	0,935	0,139326153
CTSH	7,53E-06	0,483888126	0,257	0,065	0,142766814
FSCN1	8,41E-06	0,969665079	0,465	0,254	0,15939062
TGFBR2	8,58E-06	0,801700723	0,703	0,55	0,162682754
ADGRF5	9,00E-06	0,625111353	0,505	0,254	0,170475716
ROBO4	9,24E-06	0,565691432	0,495	0,249	0,175015619
PKP4	1,33E-05	0,910148142	0,396	0,183	0,25159391
SLC12A7	1,34E-05	0,744838592	0,277	0,089	0,252989925
ZFP36L1	1,58E-05	0,554100903	0,822	0,746	0,299247672
SLCO2A1	1,59E-05	0,815677533	0,475	0,243	0,300948846
ATP2B1	1,82E-05	0,803595577	0,465	0,254	0,345541269
TGFBR3	1,83E-05	0,73268897	0,624	0,408	0,347504381
TMSB4X	2,47E-05	0,364925413	0,97	0,97	0,468013457
IGFBP4	2,51E-05	0,630431869	0,782	0,686	0,475927827
DOCK4	2,58E-05	0,526516093	0,327	0,124	0,488702505
ESAM	2,76E-05	0,587835554	0,663	0,509	0,522201023
PPP2R5A	3,11E-05	0,601182583	0,366	0,16	0,589870788
NID1	3,96E-05	0,838301254	0,446	0,26	0,750012699
FKBP1A	5,01E-05	0,561078849	0,842	0,769	0,949737138
CD200	5,16E-05	0,802043502	0,297	0,112	0,978704182
CFL1	5,51E-05	0,426294966	0,901	0,846	1
MFAP3	6,44E-05	0,515720588	0,277	0,095	1
KLF6	0,000102894	0,981581234	0,673	0,562	1
FLRT2	0,000110518	0,50583485	0,267	0,095	1
ASAP1	0,000112161	0,848527312	0,505	0,331	1
MTRNR2L8	0,000115874	0,839960263	0,277	0,107	1
RDX	0,000128903	0,63749724	0,604	0,444	1
PPM1F	0,00014258	0,500954566	0,376	0,183	1
HLA-DPA1	0,000144261	0,610774139	0,683	0,55	1
PPP3CA	0,000145383	0,908387494	0,495	0,337	1
TUBB6	0,00014616	0,689091187	0,495	0,314	1
SUN1	0,000149198	0,638037479	0,465	0,29	1
HLA-C	0,000163068	0,375438293	0,891	0,864	1
SLC2A3	0,000175782	0,502902608	0,327	0,148	1
EEF1G	0,000178788	0,377207128	0,851	0,828	1
ACTB	0,000193732	0,28650286	0,98	0,994	1
PIP4K2A	0,000222994	0,472438028	0,327	0,136	1
EFNA1	0,000271997	0,519378118	0,287	0,118	1
ELK3	0,000306848	0,47320434	0,287	0,118	1
NDRG1	0,000320535	0,647862874	0,624	0,491	1

SPG7	0,000348842	0,532197449	0,307	0,136	1
FAM241A	0,000359989	0,580402326	0,257	0,107	1
ADD3	0,00036087	0,749626234	0,356	0,189	1
AP2S1	0,000362551	0,623222443	0,505	0,32	1
MAFB	0,000371648	1,115761019	0,337	0,172	1
MEF2C	0,000378062	0,678708429	0,495	0,325	1
RALGAPA2	0,000408937	0,646645914	0,347	0,172	1
MCTP1	0,000410417	0,676477638	0,347	0,178	1
RAI14	0,000413286	0,667495746	0,446	0,278	1
CYYR1	0,000431032	0,715765174	0,436	0,266	1
TIE1	0,000431142	0,630759923	0,574	0,42	1
DUSP6	0,00043227	0,749561239	0,495	0,32	1
SGK1	0,00043829	0,910862566	0,337	0,178	1
BTN3A2	0,000483936	0,530714408	0,257	0,101	1
PMP22	0,000492397	0,680722849	0,396	0,231	1
SERPINH1	0,000514187	0,571513059	0,495	0,331	1
RHOJ	0,000522875	0,674120791	0,495	0,355	1
RASIP1	0,000553149	0,562013904	0,446	0,272	1
TMEM204	0,000573922	0,584805265	0,446	0,29	1
RBP7	0,000574234	0,872495842	0,277	0,13	1
GALNT1	0,000652744	0,524419711	0,386	0,207	1
SNTB2	0,000666665	0,590918632	0,594	0,462	1
FZD4	0,000679231	0,645803377	0,465	0,296	1
FLT1	0,000837487	0,463682195	0,535	0,367	1
NR2F2	0,000957611	0,281498856	0,723	0,503	1
SLC12A2	0,000970782	0,379039428	0,376	0,195	1
RALGDS	0,000974271	0,517690704	0,257	0,112	1
EHD4	0,001028698	0,542803942	0,297	0,148	1
LDB2	0,001066645	0,486893019	0,584	0,426	1
INSR	0,001125904	0,844499108	0,366	0,219	1
TM4SF1	0,001215675	0,462638597	0,782	0,769	1
ANXA2	0,001267323	0,499754635	0,703	0,692	1
DPYSL3	0,001489609	0,686136294	0,465	0,337	1
LMNA	0,00151811	0,364073126	0,851	0,799	1
PRKD2	0,001544776	0,42272509	0,257	0,118	1
ARPC2	0,00161024	0,329169346	0,812	0,763	1
ADGRG1	0,001901604	0,371030841	0,386	0,225	1
EFHD2	0,001906985	0,526329492	0,327	0,183	1
CYTH1	0,001972891	0,35817795	0,317	0,16	1
TMA7	0,00271893	0,368627783	0,752	0,645	1
MSN	0,003005586	0,475263351	0,683	0,615	1
TPM3	0,003090746	0,385058508	0,733	0,669	1
PITPNC1	0,003333225	0,503409897	0,257	0,13	1
JAK1	0,003335345	0,494812299	0,525	0,42	1
ZNF521	0,003446254	0,526911088	0,337	0,195	1
ENTPD1	0,003502885	0,481358807	0,594	0,485	1
RAB11A	0,003677524	0,403573469	0,653	0,538	1
ETS2	0,003692129	0,567470395	0,584	0,467	1
AKAP13	0,003802486	0,614279056	0,485	0,361	1
GAPDH	0,00423437	0,258771762	0,901	0,858	1
SELENON	0,004491697	0,561241534	0,475	0,379	1
ENG	0,004565094	0,290696256	0,782	0,598	1
CALCRL	0,004579354	0,3927607	0,703	0,586	1
STAB1	0,004825881	0,437294272	0,317	0,183	1
HLA-F	0,004917793	0,568361223	0,406	0,272	1
PTBP3	0,00496725	0,441948095	0,356	0,225	1

MGLL	0,005226171	0,553130135	0,356	0,225	1
NUAK1	0,005323616	0,520474704	0,465	0,361	1
CDH5	0,00536757	0,473049573	0,525	0,438	1
CDC37	0,005463375	0,467494608	0,554	0,444	1
ABHD17A	0,005671831	0,516553994	0,386	0,266	1
ARHGEF3	0,005844756	0,355224088	0,257	0,13	1
CCND2	0,006182745	0,399746097	0,337	0,195	1
ARHGDIB	0,006280546	0,452136052	0,713	0,615	1
SNCG	0,006458107	0,446447192	0,366	0,225	1
OLFM1	0,006462163	0,586655005	0,366	0,249	1
BAZ1A	0,006836746	0,439936249	0,327	0,183	1
GIT2	0,006846327	0,371469429	0,277	0,142	1
PXN	0,00727254	0,375545044	0,406	0,278	1
VASP	0,00785483	0,437203227	0,317	0,189	1
NCK2	0,007884668	0,323148633	0,327	0,195	1
TNFRSF1A	0,008027706	0,509870111	0,505	0,402	1
ADAM15	0,008099337	0,60110038	0,386	0,272	1
GBP2	0,008113976	0,438742376	0,307	0,172	1
IFITM2	0,008229208	0,281564087	0,901	0,911	1
SOX4	0,008290122	0,625158644	0,475	0,361	1
MYL12A	0,008416465	0,358528501	0,713	0,698	1
API5	0,008424017	0,440515765	0,366	0,243	1
PTK2B	0,008615815	0,336078517	0,376	0,254	1
PIK3C2A	0,009809522	0,454272399	0,554	0,45	1
PRPF38B	0,009822855	0,406991215	0,396	0,254	1

Cluster E.1 marker genes

gene	p_val	avg_logFC	pct.1	pct.2	p_val_adj
BMP4	2,09E-22	1,942317284	0,814	0,235	3,96E-18
EFEMP1	6,67E-19	1,740877031	0,914	0,455	1,26E-14
IGFBP3	1,37E-18	1,895875575	0,614	0,105	2,60E-14
EDN1	1,26E-15	1,817732785	0,7	0,235	2,38E-11
THBS1	8,58E-14	1,469984222	0,7	0,24	1,63E-09
MGP	1,60E-12	1,195128254	0,943	0,9	3,03E-08
OMD	3,89E-12	1,40028441	0,657	0,245	7,36E-08
CRIM1	6,50E-12	1,056052266	0,843	0,625	1,23E-07
NRG1	1,36E-11	0,977541953	0,3	0,025	2,58E-07
CTGF	1,74E-11	1,629112257	0,729	0,35	3,31E-07
TNFRSF21	3,45E-11	0,864875177	0,3	0,03	6,54E-07
CP	3,83E-11	1,066199398	0,371	0,06	7,25E-07
PCDH10	2,93E-10	1,032234186	0,429	0,095	5,55E-06
FN1	1,03E-09	0,970025831	0,886	0,66	1,94E-05
BMX	3,41E-09	1,04836277	0,529	0,195	6,45E-05
BMP6	7,29E-09	0,932239048	0,457	0,145	0,000138161
CYR61	1,59E-08	0,769304594	0,671	0,33	0,000300721
PDGFD	3,78E-07	0,929847365	0,429	0,15	0,007153862
CXCL2	4,85E-07	1,214471642	0,371	0,115	0,009191638
SERPINE1	6,44E-07	0,942876733	0,629	0,285	0,012209188
RHOB	7,01E-07	0,891159441	0,7	0,45	0,013274774
ALDH1A1	1,09E-06	0,667562025	0,386	0,13	0,02070617
LTBP2	2,07E-06	0,897672272	0,686	0,385	0,039229532
COL8A1	2,15E-06	0,820245331	0,6	0,31	0,0407445
PTGS2	2,16E-06	0,893004408	0,457	0,19	0,040908202
DHRS3	2,56E-06	0,691498293	0,529	0,25	0,048569405
BGN	3,79E-06	0,48716596	0,943	0,665	0,071807944
OAZ1	4,31E-06	0,520049246	0,829	0,69	0,081638504
EGR1	4,32E-06	0,744516288	0,786	0,515	0,081859872
SULF1	6,75E-06	0,452917088	0,843	0,5	0,128002534
PKHD1L1	8,62E-06	0,727502197	0,286	0,085	0,163335479
PAPSS2	8,80E-06	0,598792043	0,443	0,19	0,16677456
GALNT15	9,32E-06	0,739341533	0,4	0,16	0,176687274
AMOTL2	2,01E-05	0,889514817	0,543	0,33	0,380883058
INSIG2	3,84E-05	0,487203142	0,357	0,145	0,728583164
ARRB2	4,16E-05	0,450387086	0,257	0,07	0,788945459
GJA5	4,54E-05	0,590252719	0,529	0,275	0,859595197
FLNB	4,81E-05	0,622026731	0,714	0,515	0,91192242
SAT1	5,17E-05	0,614674586	0,6	0,335	0,978803955
FAM107B	6,29E-05	0,56863064	0,514	0,27	1
PLCXD3	7,38E-05	0,46700053	0,286	0,095	1
BMP2	7,48E-05	0,571554477	0,3	0,11	1
IER3	7,50E-05	0,72911547	0,486	0,27	1
CITED2	9,92E-05	0,502830337	0,286	0,1	1
PROCR	0,000113534	0,47208117	0,629	0,39	1
NEXN	0,000175789	0,56077486	0,286	0,1	1
NR5A1	0,000184013	0,570635819	0,614	0,395	1
SELP	0,000192809	0,531094098	0,629	0,4	1
CYBRD1	0,000231472	0,612513577	0,586	0,38	1
INMT	0,00023793	0,79118288	0,343	0,15	1
IGFBP2	0,00025245	0,393149738	0,429	0,205	1
PLXNA4	0,000256234	0,433201667	0,343	0,145	1
CCDC71L	0,000299945	0,279851466	0,257	0,085	1

OGN	0,000300165	0,626144472	0,6	0,37	1
HMCN1	0,000321653	0,443874231	0,486	0,265	1
PHACTR2	0,000330058	0,555348125	0,614	0,46	1
AAK1	0,000344559	0,524669209	0,729	0,57	1
MYL12B	0,000388899	0,348048853	0,8	0,715	1
CYP4X1	0,000411063	0,546825065	0,257	0,095	1
PVR	0,000420032	0,551461475	0,443	0,225	1
S100A13	0,00046661	0,434843323	0,471	0,26	1
TIMP1	0,000560925	0,367406232	0,857	0,87	1
C1R	0,000614188	0,36501331	0,714	0,47	1
LIMCH1	0,000615119	0,569522676	0,657	0,44	1
IRF6	0,000620561	0,33902064	0,314	0,13	1
SELL	0,000757914	0,744215494	0,486	0,29	1
MTUS1	0,000808073	0,532118976	0,657	0,48	1
ENG	0,000831985	0,463587307	0,757	0,635	1
CLU	0,000993934	0,407653351	0,871	0,745	1
NR4A2	0,001035554	0,452974659	0,357	0,165	1
TMEM98	0,00117445	0,415423549	0,257	0,105	1
FBLIM1	0,001197375	0,444794465	0,7	0,54	1
CTNNAL1	0,00125411	0,485208097	0,314	0,15	1
PKD2	0,001351118	0,522418556	0,514	0,335	1
MEDAG	0,001366032	0,345216665	0,414	0,205	1
MEGF6	0,001383645	0,534437792	0,343	0,18	1
CEBPD	0,001544255	0,549246608	0,4	0,22	1
sep-11	0,001655962	0,40143871	0,529	0,33	1
IL6ST	0,001713572	0,442445827	0,8	0,65	1
PTGIS	0,001765139	0,326460457	0,629	0,41	1
HBEGF	0,001918334	0,529531162	0,329	0,16	1
LMO2	0,001982882	0,523958891	0,643	0,55	1
NUS1P3	0,002242336	0,3147475	0,286	0,13	1
MFSD1	0,002333054	0,312114667	0,386	0,205	1
MRPS15	0,002391458	0,35543012	0,3	0,14	1
SUPT4H1	0,002511569	0,474078459	0,343	0,17	1
SCRN1	0,002794003	0,438109032	0,271	0,125	1
GJA1	0,003062789	0,61587467	0,543	0,375	1
TNFRSF10D	0,003164551	0,308039667	0,286	0,135	1
GNPTAB	0,003972785	0,278575723	0,343	0,18	1
CLINT1	0,00397465	0,30933629	0,329	0,16	1
CDA	0,004224437	0,327317418	0,314	0,155	1
C1RL	0,004339881	0,425858865	0,329	0,175	1
SRPX	0,004460384	0,307259692	0,286	0,135	1
SLC38A2	0,004550417	0,41725965	0,6	0,455	1
FAM43A	0,004584236	0,502045547	0,314	0,17	1
POLDIP2	0,0046272	0,330601536	0,271	0,125	1
BNIP3L	0,004967396	0,425379384	0,586	0,43	1
ELN	0,004977886	0,380018387	0,657	0,45	1
MLLT1	0,005433633	0,381111128	0,429	0,27	1
IGF1R	0,005770566	0,44626805	0,343	0,195	1
PDE3A	0,005860802	0,369038366	0,386	0,21	1
IQCK	0,005894695	0,329144711	0,286	0,145	1
NAV1	0,006000526	0,632713771	0,429	0,295	1
WWTR1	0,006435681	0,504192354	0,7	0,575	1
TMEM47	0,00652215	0,342345183	0,543	0,365	1
GYG1	0,006589244	0,384337316	0,314	0,17	1
TGOLN2	0,006650305	0,348969432	0,714	0,595	1
METRNL	0,006727065	0,396395568	0,357	0,21	1

HHEX	0,006774023	0,53464788	0,343	0,195	1
ABLIM1	0,006885039	0,312880818	0,586	0,41	1
RGS3	0,006936902	0,280665511	0,314	0,16	1
C1S	0,007813879	0,476745567	0,529	0,365	1
MDH2	0,007931628	0,318641501	0,343	0,195	1
TBC1D5	0,007936315	0,349639249	0,386	0,24	1
ARL6IP1	0,008081038	0,255719031	0,557	0,375	1
JUNB	0,008125217	0,416966935	0,757	0,67	1
ZMPSTE24	0,00864479	0,311776604	0,371	0,22	1
PGRMC2	0,008667299	0,458304904	0,557	0,385	1
CMAHP	0,008772644	0,361271896	0,457	0,295	1
ADSS	0,008797676	0,253233165	0,3	0,16	1
BMPR2	0,009261893	0,339059166	0,757	0,615	1
VCAM1	0,009792098	0,646883	0,357	0,225	1

Cluster E.2 marker genes

gene	p_val	avg_logFC	pct.1	pct.2	p_val_adj
DKK2	6,02E-28	1,860693435	0,708	0,068	1,14E-23
LTC4S	2,26E-23	1,108902373	0,769	0,122	4,27E-19
FGF18	2,34E-23	1,506774698	0,646	0,078	4,43E-19
CPAMD8	1,81E-21	1,401988838	0,692	0,127	3,43E-17
ELN	2,87E-20	1,449126983	0,923	0,371	5,44E-16
SULF1	7,78E-20	1,320640702	0,954	0,473	1,47E-15
HEG1	9,71E-20	1,464432866	0,938	0,61	1,84E-15
MPZL2	2,95E-19	1,142547239	0,923	0,4	5,58E-15
LYPD2	7,02E-19	1,212556779	0,354	0	1,33E-14
CDH23	3,61E-18	1,367507228	0,662	0,141	6,84E-14
LSR	1,25E-17	1,09540629	0,538	0,073	2,37E-13
FBLN2	1,96E-17	1,616653695	0,723	0,224	3,71E-13
PTGIS	2,23E-17	1,225471741	0,877	0,337	4,23E-13
PTPRJ	2,45E-17	1,2937962	0,677	0,161	4,63E-13
CLDN5	5,60E-17	1,336468652	0,785	0,239	1,06E-12
ASS1	1,18E-16	1,098701167	0,754	0,224	2,24E-12
AIF1L	1,97E-16	0,993619097	0,615	0,117	3,73E-12
SRGN	2,25E-16	1,084891668	0,938	0,644	4,27E-12
ITLN1	8,00E-16	1,627796206	0,769	0,254	1,52E-11
NOG	2,71E-15	1,125508712	0,354	0,02	5,14E-11
SLC9A3R2	3,06E-15	1,14871264	0,862	0,429	5,80E-11
CLU	3,17E-15	0,894341742	1	0,707	6,02E-11
RAMP2	4,76E-15	1,007223467	0,892	0,429	9,03E-11
PLLP	9,63E-15	0,748535028	0,4	0,039	1,82E-10
RNASE1	1,25E-14	1,050316067	0,938	0,59	2,37E-10
CYP1B1	1,59E-14	0,925224907	0,769	0,244	3,02E-10
GJA5	1,79E-14	1,068218008	0,738	0,215	3,40E-10
KCNN4	3,47E-14	0,615097744	0,4	0,039	6,57E-10
PTPRB	5,11E-14	0,969929103	0,846	0,38	9,68E-10
MECOM	5,11E-14	1,087512442	0,708	0,244	9,69E-10
ITPR2	5,43E-14	0,883688804	0,831	0,356	1,03E-09
HYAL2	7,42E-14	1,22265919	0,862	0,424	1,41E-09
FAM19A5	1,45E-13	0,861862185	0,292	0,01	2,75E-09
ANXA3	7,26E-13	0,690806575	0,492	0,088	1,37E-08
FGL2	7,84E-13	0,769044291	0,646	0,171	1,49E-08
IFI27	1,22E-12	0,952757758	0,877	0,527	2,32E-08
KLF2	1,93E-12	0,783993622	0,862	0,361	3,65E-08
SEMA3F	2,29E-12	0,78643743	0,662	0,224	4,34E-08
ACKR3	4,04E-12	1,196981954	0,446	0,083	7,65E-08
PECAM1	7,34E-12	0,775256716	0,985	0,712	1,39E-07
AHNAK2	7,87E-12	0,777724064	0,569	0,151	1,49E-07
BCAT2	1,16E-11	0,791976024	0,738	0,283	2,20E-07
HYAL1	1,50E-11	0,625382559	0,4	0,063	2,84E-07
SPTAN1	1,61E-11	0,872081366	0,862	0,527	3,05E-07
NOS3	1,61E-11	0,860402702	0,692	0,288	3,06E-07
FAM107A	2,25E-11	1,230671795	0,738	0,366	4,27E-07
ABI3BP	2,87E-11	1,032842394	0,492	0,122	5,43E-07
MMP28	3,47E-11	0,70341213	0,431	0,083	6,57E-07
TGM2	4,24E-11	0,82019913	0,892	0,541	8,04E-07
PTPRG	4,64E-11	0,705821485	0,477	0,102	8,78E-07
FXYD6	4,76E-11	0,660260348	0,769	0,278	9,02E-07
ST6GALNAC1	4,83E-11	0,42115047	0,262	0,015	9,15E-07
ITPRID2	5,04E-11	0,889195892	0,723	0,298	9,56E-07

NDRG4	6,30E-11	0,827897656	0,538	0,151	1,19E-06
NES	6,96E-11	0,841597093	0,677	0,254	1,32E-06
CPXM2	7,22E-11	0,793698136	0,785	0,385	1,37E-06
IFI6	7,31E-11	1,061770236	0,8	0,434	1,38E-06
KIF26A	9,75E-11	0,665747742	0,4	0,068	1,85E-06
STMN3	1,36E-10	0,924545863	0,6	0,215	2,58E-06
DUSP5	1,67E-10	0,913980819	0,477	0,117	3,17E-06
MTMR11	1,91E-10	1,061667786	0,446	0,102	3,61E-06
GAS6	1,94E-10	0,82126649	0,877	0,483	3,68E-06
ARL4D	2,19E-10	0,869558881	0,354	0,054	4,14E-06
NTN1	4,48E-10	0,503978944	0,415	0,083	8,49E-06
CALM1	6,20E-10	0,496915868	1	0,888	1,17E-05
LIMS2	6,42E-10	0,797300006	0,785	0,415	1,22E-05
PODXL	7,95E-10	0,707562262	0,815	0,454	1,51E-05
GCH1	1,09E-09	0,649565725	0,308	0,039	2,06E-05
CD58	1,11E-09	0,639071784	0,415	0,093	2,11E-05
SELP	1,11E-09	0,790448013	0,769	0,361	2,11E-05
GATA6	1,13E-09	0,616373732	0,569	0,171	2,14E-05
HLA-E	1,33E-09	0,537413786	0,985	0,893	2,53E-05
HSPA2	1,40E-09	0,450298587	0,308	0,039	2,66E-05
GFOD1	1,72E-09	0,706220356	0,538	0,176	3,27E-05
B3GALNT1	1,77E-09	0,678276395	0,431	0,107	3,36E-05
RUNX1T1	1,83E-09	0,682622844	0,723	0,322	3,47E-05
LRIG1	2,11E-09	0,728412782	0,462	0,132	4,00E-05
C1R	2,32E-09	0,779230858	0,846	0,434	4,39E-05
PROCR	3,42E-09	0,822896293	0,754	0,356	6,49E-05
PTGDS	4,19E-09	1,322845602	0,354	0,068	7,95E-05
PLCG2	5,16E-09	0,442806174	0,385	0,078	9,78E-05
CD59	5,71E-09	0,568865032	0,954	0,795	0,000108212
AFAP1L2	5,85E-09	0,45280332	0,338	0,059	0,000110787
C1S	6,91E-09	0,683981912	0,723	0,307	0,000130904
VWF	7,68E-09	0,599836617	0,954	0,615	0,000145473
EMP2	7,77E-09	0,728304602	0,862	0,561	0,000147283
ID1	8,34E-09	0,710557597	0,877	0,478	0,000157988
HMCN1	9,71E-09	0,841931068	0,615	0,229	0,000184097
TLNRD1	1,06E-08	0,694302436	0,615	0,254	0,000201637
ALDH2	1,42E-08	0,740699582	0,631	0,283	0,000268612
POU4F1	1,74E-08	0,771556826	0,292	0,044	0,00032901
S100A6	1,81E-08	0,439324887	1	0,893	0,000343612
EMP3	1,87E-08	1,053786253	0,631	0,293	0,000354848
MINPP1	2,10E-08	0,493216357	0,338	0,063	0,000398794
MTRF1L	2,57E-08	0,702521821	0,554	0,205	0,000487089
HSD17B12	2,72E-08	0,595917103	0,785	0,454	0,000516252
SMAD6	3,07E-08	0,739385822	0,569	0,21	0,000580943
CD320	3,30E-08	0,557379012	0,385	0,093	0,000624907
ATP2A3	3,68E-08	0,463433291	0,431	0,117	0,000697738
PLEC	4,32E-08	0,571105777	0,815	0,493	0,000818927
CD81	5,10E-08	0,569828591	0,908	0,805	0,000967123
GAS2L1	5,15E-08	0,570563879	0,369	0,093	0,000975183
NFIX	5,60E-08	0,606703505	0,6	0,254	0,001060938
MMP2	6,73E-08	0,818064575	0,677	0,371	0,00127499
MMRN2	7,63E-08	0,605354661	0,862	0,58	0,001445358
TSPAN2	7,80E-08	0,548181867	0,385	0,093	0,001477433
IL1R1	8,33E-08	0,721918475	0,662	0,298	0,001578781
CLDN15	8,62E-08	0,387355013	0,308	0,059	0,001633579
HIF3A	9,51E-08	0,62155946	0,292	0,054	0,001801411

LMO2	9,78E-08	0,574648845	0,877	0,478	0,001853839
COBLL1	1,01E-07	0,407506518	0,308	0,059	0,001919314
TCN2	1,04E-07	0,779852756	0,508	0,185	0,00196269
JAG1	1,09E-07	0,692223217	0,738	0,38	0,002062907
NQO1	1,12E-07	0,584254933	0,431	0,127	0,002118133
TMBIM1	1,16E-07	0,669929201	0,585	0,254	0,002205816
PIK3R3	1,44E-07	0,317967249	0,462	0,141	0,002737628
LFNG	1,57E-07	0,43075978	0,369	0,088	0,002975387
CPE	1,75E-07	0,733637148	0,677	0,288	0,003311081
ARHGAP4	1,84E-07	0,416331562	0,277	0,049	0,003495026
C19orf33	1,99E-07	0,429539362	0,292	0,059	0,00377716
RHOA	2,12E-07	0,438527731	0,938	0,712	0,00402103
PTPRF	2,93E-07	0,36725086	0,477	0,156	0,005550614
MEDAG	3,46E-07	0,601103692	0,508	0,18	0,006551315
FRMD4B	3,46E-07	0,451428641	0,646	0,263	0,006560813
MALL	3,61E-07	0,481477314	0,692	0,327	0,006844627
DOK4	3,65E-07	0,508174839	0,323	0,083	0,006923746
BST2	3,67E-07	0,586591439	0,646	0,283	0,006957022
METTL7A	4,03E-07	0,46350664	0,785	0,4	0,00763262
NPEPPS	4,15E-07	0,48064367	0,569	0,234	0,007863224
LIMA1	4,67E-07	0,536928659	0,754	0,39	0,008852505
HRAS	5,00E-07	0,500612284	0,462	0,156	0,009466499
SELL	5,03E-07	0,352535086	0,615	0,254	0,009526911
ITGA3	5,11E-07	0,499625869	0,523	0,2	0,009684578
KCTD12	5,44E-07	0,624052577	0,769	0,439	0,010303734
CCDC3	5,62E-07	0,552832138	0,554	0,22	0,010640451
GLUL	5,89E-07	0,886563563	0,662	0,376	0,011164511
TIMP3	6,09E-07	0,829462917	0,662	0,332	0,011549955
ACVRL1	6,36E-07	0,579585418	0,677	0,327	0,012048622
GRB14	6,37E-07	0,546675172	0,262	0,049	0,012080601
PLA2G4A	6,44E-07	0,592458952	0,323	0,083	0,012197552
CFB	7,61E-07	0,446913708	0,354	0,093	0,014428365
EVA1C	7,74E-07	0,564818786	0,431	0,151	0,014668901
ID2	8,28E-07	0,729774393	0,646	0,312	0,015699306
SEC11C	8,82E-07	0,42653202	0,477	0,176	0,016718204
HSPA12B	9,09E-07	0,443236715	0,338	0,088	0,017234004
ARL2BP	1,10E-06	0,469641541	0,508	0,19	0,020907287
TBX1	1,13E-06	0,449770223	0,477	0,176	0,021345858
COL8A1	1,13E-06	0,531766991	0,646	0,302	0,02139278
FAM198B	1,17E-06	0,552743836	0,862	0,561	0,022136881
SOX7	1,19E-06	0,564631746	0,585	0,268	0,022607258
DAP	1,24E-06	0,389720194	0,708	0,337	0,023560021
PINK1	1,29E-06	0,514458032	0,585	0,249	0,02443775
FEZ1	1,36E-06	0,537380297	0,354	0,102	0,025828016
PTPN14	1,49E-06	0,680025859	0,723	0,439	0,028295589
FGR	1,50E-06	0,520558174	0,323	0,083	0,028343278
PIK3C2B	1,83E-06	0,488760269	0,477	0,185	0,034711647
CGNL1	2,15E-06	0,464356408	0,292	0,068	0,040709535
SLC25A29	2,32E-06	0,437187767	0,323	0,088	0,044003726
CELF2	2,52E-06	0,555114432	0,523	0,234	0,047661884
LTBP4	2,54E-06	0,416011277	0,708	0,351	0,048084546
ADAMTS1	2,85E-06	0,827301482	0,631	0,317	0,054036424
SETBP1	2,97E-06	0,471349172	0,446	0,156	0,056299735
RBMS3	3,07E-06	0,486065434	0,723	0,395	0,058267904
ARHGEF37	3,12E-06	0,264682432	0,277	0,059	0,05915057
NPR1	3,29E-06	0,494355244	0,369	0,122	0,062259583

TMEM120A	3,35E-06	0,552574677	0,446	0,166	0,063403492
PTPRM	3,43E-06	0,587830508	0,662	0,341	0,065020495
AGRN	3,49E-06	0,538489177	0,569	0,273	0,066166295
LRRFIP1	3,77E-06	0,424959374	0,954	0,702	0,071404097
PLXNA2	3,89E-06	0,473504038	0,708	0,361	0,0736962
PLCB4	4,09E-06	0,389900062	0,338	0,093	0,077439121
KLF4	4,35E-06	0,626727062	0,631	0,341	0,082483929
ITGB4	5,06E-06	0,589583818	0,569	0,278	0,095960951
EIF4EBP2	5,08E-06	0,308629349	0,6	0,283	0,09634249
SHE	5,29E-06	0,482749904	0,538	0,244	0,100269146
CAPN5	5,97E-06	0,348232974	0,262	0,063	0,1131866
ICAM1	6,18E-06	0,807920681	0,415	0,151	0,117159982
TSPAN9	6,73E-06	0,446660442	0,446	0,176	0,127443254
TRIP11	6,86E-06	0,435241267	0,431	0,156	0,129946371
PGRMC2	7,60E-06	0,494672179	0,631	0,366	0,143987333
ZFYVE21	7,73E-06	0,487454478	0,508	0,224	0,146414578
CTNNB1	8,01E-06	0,426162246	0,815	0,522	0,151840259
RAB7A	8,20E-06	0,517647782	0,738	0,473	0,155378062
GANAB	8,23E-06	0,452754569	0,6	0,268	0,155881423
TNKS1BP1	8,59E-06	0,506653007	0,508	0,224	0,162763082
RARRES2	8,98E-06	0,564251666	0,262	0,063	0,170096607
SLC48A1	9,14E-06	0,508049775	0,446	0,176	0,173212168
STMN1	9,48E-06	0,598242535	0,462	0,19	0,179687195
TNS2	1,03E-05	0,497763698	0,492	0,22	0,194650334
ABLIM1	1,10E-05	0,454849766	0,723	0,371	0,207711917
AMFR	1,17E-05	0,43117848	0,446	0,176	0,221734538
TM6SF1	1,21E-05	0,288063335	0,308	0,083	0,229062067
NIPAL3	1,26E-05	0,44292736	0,492	0,205	0,238504274
CDH11	1,27E-05	0,551912972	0,431	0,171	0,240320645
FUT8	1,36E-05	0,437722063	0,262	0,068	0,257602232
ATP13A3	1,39E-05	0,777503593	0,523	0,254	0,264101783
LINC00674	1,43E-05	0,448222833	0,738	0,454	0,270483934
GOLGA3	1,47E-05	0,357359433	0,523	0,229	0,277714619
WWC3	1,48E-05	0,358583691	0,6	0,288	0,280790977
LAMP1	1,62E-05	0,47620978	0,769	0,498	0,306684008
CAPZA2	1,81E-05	0,453136799	0,692	0,405	0,3429836
TMSB10	1,82E-05	0,315400097	0,985	0,907	0,345218717
IFI44L	1,91E-05	0,358464532	0,538	0,229	0,361002592
MEIS2	1,92E-05	0,449996966	0,369	0,132	0,364436182
MEIS3P1	1,93E-05	0,258572227	0,262	0,063	0,364792737
DUSP22	1,96E-05	0,406324799	0,338	0,112	0,372181071
PEF1	2,06E-05	0,461551846	0,431	0,176	0,390836469
GSN	2,22E-05	0,324493267	0,938	0,824	0,420585829
ARL6IP5	2,22E-05	0,528357126	0,785	0,571	0,421273612
CNOT8	2,24E-05	0,386041438	0,369	0,137	0,424794256
PPIC	2,28E-05	0,690625316	0,538	0,283	0,432760608
KLF3	2,50E-05	0,566211775	0,492	0,22	0,474171799
CEBPB	2,55E-05	0,457540631	0,415	0,156	0,482628853
PLD3	2,75E-05	0,477913292	0,431	0,185	0,521984797
LYST	2,90E-05	0,428644929	0,569	0,273	0,549004809
GLOD4	2,90E-05	0,357824521	0,292	0,088	0,550388909
GOSR2	2,90E-05	0,27034505	0,292	0,088	0,550388909
SH3BP4	2,95E-05	0,553053483	0,446	0,18	0,55936934
IRF9	3,00E-05	0,390827804	0,262	0,073	0,5693159
MRAS	3,05E-05	0,629428242	0,369	0,141	0,578914763
RIPOR1	3,12E-05	0,506813207	0,615	0,312	0,591415557

BGN	3,21E-05	0,306157023	0,908	0,683	0,608880119
STX7	3,49E-05	0,48206557	0,446	0,19	0,66119195
SMPD1	3,59E-05	0,373775435	0,277	0,083	0,681134811
SLAIN2	3,70E-05	0,380819511	0,446	0,185	0,701586173
FOXC1	3,99E-05	0,410749063	0,769	0,468	0,75682595
TTYH3	4,10E-05	0,258718842	0,369	0,127	0,776690523
SH3BP2	4,17E-05	0,422643858	0,646	0,346	0,789356834
SKIL	4,28E-05	0,389196046	0,677	0,38	0,810903812
CD34	4,28E-05	0,470482771	0,815	0,512	0,811334499
CYSTM1	4,44E-05	0,602769552	0,369	0,151	0,842120583
NAA10	4,71E-05	0,569824956	0,4	0,166	0,892116079
MKNK2	4,91E-05	0,434515595	0,723	0,449	0,930964673
SYNPO	4,97E-05	0,472141854	0,785	0,556	0,942661195
PLTP	5,18E-05	0,258817847	0,677	0,361	0,981205163
IER5	5,26E-05	0,30798161	0,323	0,107	0,996364773
ARRB1	5,39E-05	0,382740138	0,415	0,171	1
TEK	5,41E-05	0,309246651	0,538	0,249	1
C14orf132	5,63E-05	0,316189864	0,277	0,078	1
TSPAN6	5,66E-05	0,426704489	0,385	0,156	1
ARFGEF3	5,69E-05	0,414064968	0,354	0,127	1
LRP11	5,74E-05	0,368939883	0,338	0,117	1
TENT5A	5,99E-05	0,662581834	0,4	0,171	1
PHACTR4	5,99E-05	0,446993786	0,662	0,385	1
CALCRL	6,10E-05	0,415725154	0,862	0,556	1
FTH1	6,24E-05	0,355920968	0,969	0,873	1
SLC25A11	6,39E-05	0,420760748	0,292	0,093	1
SSRP1	6,45E-05	0,389633991	0,369	0,141	1
HPCAL1	6,97E-05	0,435704083	0,508	0,249	1
SOX13	7,08E-05	0,536532358	0,4	0,161	1
FAXDC2	7,44E-05	0,39956852	0,338	0,122	1
ZBTB4	7,47E-05	0,47284699	0,554	0,283	1
EXT1	7,55E-05	0,4375145	0,523	0,273	1
SLPI	7,65E-05	0,352041528	0,292	0,088	1
MET	7,67E-05	0,366127912	0,323	0,112	1
CDIP1	7,67E-05	0,406792031	0,323	0,117	1
NPR2	7,95E-05	0,259819934	0,385	0,137	1
CYB5A	8,19E-05	0,440947598	0,354	0,141	1
ATP6AP1	8,28E-05	0,362973047	0,446	0,205	1
SIRPA	8,37E-05	0,424042163	0,4	0,171	1
MPC2	8,77E-05	0,327838095	0,369	0,141	1
IFIT1	9,15E-05	0,664635149	0,292	0,098	1
FXYD5	9,59E-05	0,428686208	0,738	0,478	1
AZIN1	0,000100191	0,46009954	0,492	0,234	1
ANOS1	0,000103493	0,310010908	0,277	0,083	1
JCAD	0,000106618	0,467468016	0,508	0,234	1
CALU	0,000111331	0,495472078	0,692	0,439	1
TAL1	0,000111669	0,300482272	0,354	0,132	1
WSB1	0,00011433	0,466990029	0,862	0,644	1
OCIAD2	0,000115215	0,419896801	0,323	0,117	1
GATA2	0,000118371	0,493876138	0,492	0,234	1
ECE1	0,000122271	0,444948264	0,785	0,566	1
ABR	0,00012541	0,355632398	0,477	0,234	1
AKT1	0,000126392	0,433509759	0,492	0,244	1
ZBTB1	0,000130366	0,274613228	0,262	0,078	1
FKBP5	0,000130396	0,490346334	0,523	0,273	1
CCM2L	0,000133708	0,346525275	0,415	0,176	1

SOX17	0,000140102	0,487152751	0,262	0,083	1
TSPO	0,000143715	0,380448582	0,569	0,317	1
LY6E	0,000145194	0,397913367	0,6	0,341	1
GSTO1	0,000150286	0,363138308	0,554	0,293	1
DIPK1B	0,000152262	0,267328376	0,415	0,18	1
OS9	0,000156487	0,589226372	0,692	0,478	1
GDI1	0,000156529	0,271490091	0,646	0,337	1
GABARAPL2	0,000160182	0,499502386	0,692	0,449	1
FAM107B	0,000162346	0,459207677	0,523	0,273	1
CPNE3	0,000163012	0,32750353	0,569	0,288	1
CLPTM1L	0,000163864	0,41081635	0,4	0,166	1
TMEM59	0,000165183	0,548275678	0,785	0,571	1
CIC	0,000167359	0,386091094	0,415	0,19	1
CDC42EP3	0,000167611	0,471310871	0,523	0,263	1
DIAPH1	0,000168381	0,593670828	0,462	0,21	1
NFATC2	0,000181517	0,311886461	0,385	0,161	1
PRDX1	0,000188232	0,32936167	0,754	0,527	1
BHLHE40	0,000191647	0,437116057	0,492	0,234	1
PKD1P1	0,000195882	0,323533494	0,723	0,424	1
SYNGR2	0,00019816	0,354547617	0,6	0,366	1
GABARAPL1	0,00020026	0,53355354	0,385	0,185	1
CMKLR1	0,000200686	0,349889893	0,308	0,107	1
H3F3B	0,000209972	0,322666256	0,954	0,785	1
MCAM	0,000219768	0,254652395	0,754	0,454	1
TMTC1	0,000232407	0,474009611	0,462	0,224	1
PDE4A	0,000236812	0,323100469	0,262	0,088	1
SRPX	0,000243205	0,563344825	0,323	0,127	1
IDS	0,000245286	0,343991981	0,785	0,478	1
NUCB2	0,000248548	0,56800684	0,569	0,322	1
PKN3	0,000249058	0,538462983	0,308	0,112	1
FGD5	0,000250871	0,454301955	0,446	0,229	1
GOLGA4	0,000269338	0,310929364	0,6	0,332	1
PER3	0,000290628	0,264112549	0,354	0,141	1
PBRM1	0,000291763	0,42617201	0,385	0,171	1
PTMS	0,000312322	0,291238137	0,892	0,624	1
ECSCR	0,000318917	0,307793707	0,738	0,473	1
DPY19L1	0,000322975	0,509994791	0,462	0,249	1
FGF2	0,000331446	0,290813693	0,369	0,146	1
POLR3GL	0,00034097	0,280929796	0,415	0,195	1
KLC1	0,000351509	0,297413136	0,492	0,254	1
mrt-06	0,000353888	0,524820539	0,631	0,395	1
CRTAC1	0,000355349	0,674366599	0,585	0,346	1
NUP160	0,000360553	0,269674483	0,385	0,171	1
EVI5	0,000383378	0,308769211	0,462	0,22	1
GRN	0,000390969	0,333276407	0,8	0,546	1
HDGF	0,0004029	0,374487314	0,585	0,322	1
ZNF503	0,000431008	0,547634042	0,323	0,127	1
SH3BGRL3	0,000437584	0,41327709	0,923	0,79	1
BBS2	0,000441904	0,258541284	0,262	0,088	1
DENND3	0,000456561	0,280044097	0,308	0,122	1
PRXL2B	0,000457318	0,332610117	0,354	0,151	1
SV2A	0,000479996	0,447973045	0,277	0,102	1
SLC44A2	0,000480034	0,514735972	0,646	0,493	1
ENO2	0,000483428	0,377260397	0,262	0,093	1
RAMP3	0,000487618	0,418060845	0,554	0,288	1
SNX18	0,000493119	0,374828277	0,369	0,171	1

HSPA1B	0,000494461	0,344986134	0,277	0,098	1
CMTM6	0,000505445	0,317513705	0,6	0,356	1
WLS	0,000505585	0,426273764	0,477	0,254	1
CLEC3B	0,000522648	0,450926134	0,662	0,395	1
MIA3	0,000543993	0,49500723	0,431	0,205	1
SERPINE2	0,000552049	0,477807421	0,338	0,141	1
RPRD1A	0,000554563	0,331201021	0,277	0,102	1
DUSP1	0,000556502	0,393560352	0,785	0,502	1
ORAI1	0,000562753	0,328014079	0,262	0,098	1
MOB2	0,000594656	0,297471887	0,385	0,166	1
ARMCX2	0,00060537	0,29153563	0,308	0,122	1
NPTN	0,000626291	0,386963971	0,585	0,351	1
ENSA	0,000628943	0,415601617	0,585	0,366	1
SHROOM4	0,000636825	0,280410063	0,569	0,307	1
CAV1	0,000643916	0,338586929	0,908	0,761	1
SLC18A1	0,000645241	0,254232393	0,738	0,429	1
NCOR2	0,000694714	0,316197575	0,415	0,205	1
KCNN3	0,000696059	0,426259492	0,508	0,273	1
DDOST	0,0007043	0,409623829	0,662	0,415	1
IMPDH1	0,000728817	0,28652583	0,338	0,146	1
CRIP2	0,000744596	0,404018755	0,815	0,659	1
SEC22C	0,000797671	0,359155042	0,415	0,205	1
CDV3	0,000809803	0,261108247	0,6	0,356	1
NDUFC1	0,000809833	0,254636088	0,585	0,346	1
BCL2L1	0,000812811	0,329655487	0,477	0,254	1
TGOLN2	0,000818844	0,287103313	0,815	0,566	1
PSMD6	0,00084314	0,305465927	0,369	0,171	1
DNAJC1	0,000852014	0,541411032	0,385	0,18	1
SEC62	0,000865975	0,274188454	0,877	0,659	1
MAP7D1	0,000869697	0,31665545	0,446	0,224	1
PTTG1IP	0,000874387	0,318002721	0,769	0,595	1
AES	0,000881717	0,326606002	0,831	0,595	1
LSG1	0,000886772	0,337173165	0,369	0,185	1
ISCU	0,000890335	0,310390777	0,677	0,424	1
PGM5	0,000922841	0,28508733	0,385	0,18	1
ADGRL1	0,000939988	0,322316915	0,308	0,127	1
IFITM1	0,000959237	0,369723621	0,846	0,639	1
TMED9	0,000976448	0,309194758	0,492	0,273	1
ARPC1B	0,001003396	0,436009626	0,662	0,449	1
NFKBIA	0,001044512	0,433377619	0,677	0,439	1
ARIH1	0,001080287	0,348425015	0,292	0,112	1
ALDH1A2	0,001122003	0,464394557	0,369	0,176	1
CMIP	0,00114041	0,285710958	0,831	0,576	1
CDKN1C	0,001159255	0,501249782	0,385	0,18	1
SLC43A3	0,001161286	0,276780034	0,4	0,185	1
F11R	0,00116347	0,289707629	0,4	0,195	1
FAM234A	0,001231122	0,291300621	0,292	0,122	1
PSMC3	0,001249036	0,254280753	0,385	0,19	1
LYPLA1	0,00131615	0,256277735	0,338	0,151	1
FBXO9	0,001350034	0,305102997	0,477	0,249	1
CTSZ	0,001385988	0,344980132	0,292	0,122	1
SERINC1	0,00140072	0,290473945	0,769	0,576	1
EEF2K	0,001409008	0,295077303	0,262	0,102	1
PPP2CB	0,00144437	0,361468831	0,523	0,302	1
IGFBP6	0,001476834	0,425282862	0,323	0,146	1
TRAM1	0,001479415	0,275185687	0,769	0,512	1

SELENBP1	0,00167591	0,263115536	0,292	0,117	1
MGAT4A	0,001786737	0,395881373	0,415	0,224	1
ARHGEF10	0,001791191	0,32467657	0,4	0,2	1
ATP6V0E1	0,001794557	0,333764828	0,662	0,468	1
PAFAH1B2	0,001807301	0,324187636	0,646	0,4	1
LPGAT1	0,00181525	0,391182427	0,415	0,224	1
VPS29	0,00187035	0,269451304	0,492	0,263	1
IFNAR1	0,001872264	0,325352689	0,477	0,263	1
KDELR3	0,001889789	0,408365979	0,262	0,107	1
PTEN	0,001946132	0,476591737	0,538	0,332	1
CTSK	0,001973532	0,455441508	0,385	0,2	1
IKBKG	0,001986957	0,271769671	0,292	0,127	1
MYO1C	0,001991878	0,304659082	0,646	0,395	1
PHF1	0,00202342	0,262560119	0,4	0,2	1
BTBD6	0,00210274	0,295230171	0,292	0,127	1
BLCAP	0,002103092	0,262127608	0,446	0,229	1
CTDSP1	0,002172465	0,261461608	0,538	0,322	1
CARD16	0,002198818	0,33482973	0,323	0,151	1
TMEM184B	0,002289123	0,254632102	0,492	0,273	1
FLOT2	0,002322209	0,402241237	0,415	0,234	1
CCND1	0,002381529	0,299397436	0,538	0,327	1
TM2D3	0,002419782	0,3131119	0,292	0,127	1
HNRNPAB	0,002466212	0,376704481	0,585	0,341	1
ACSL5	0,002526037	0,347700285	0,369	0,19	1
PNMA1	0,002531131	0,314262977	0,323	0,151	1
MANSC1	0,002539646	0,250282731	0,477	0,268	1
TSPAN3	0,002563433	0,32405134	0,677	0,463	1
BTG2	0,002586199	0,381477041	0,508	0,302	1
S100A4	0,002595333	0,304773697	0,662	0,454	1
CSDE1	0,00262248	0,254397305	0,923	0,727	1
APC	0,002702609	0,275503326	0,262	0,107	1
SPG11	0,002702609	0,259330879	0,262	0,107	1
PIGK	0,00279627	0,261476849	0,308	0,141	1
RSRP1	0,002804725	0,319442858	0,415	0,224	1
MAP3K6	0,00297165	0,477757146	0,262	0,112	1
INPP1	0,002998515	0,282141225	0,369	0,185	1
ZEB1	0,003138913	0,453912132	0,523	0,307	1
ATP5PD	0,003154056	0,274915842	0,569	0,346	1
CD151	0,003182914	0,293954732	0,862	0,649	1
NFIA	0,003638767	0,273798959	0,892	0,717	1
GGNBP2	0,003710917	0,347898752	0,431	0,234	1
CRIM1	0,003752083	0,275828269	0,846	0,629	1
DYNLRB1	0,003866586	0,34306819	0,662	0,444	1
BMPR2	0,003887825	0,315450004	0,785	0,61	1
NPDC1	0,003931095	0,264810033	0,738	0,493	1
PLXNA4	0,003961619	0,268764533	0,323	0,156	1
SERINC3	0,00405061	0,331143398	0,615	0,429	1
PAPSS2	0,004094628	0,283297367	0,4	0,21	1
SLCO2A1	0,00410578	0,322025389	0,492	0,278	1
POR	0,004146484	0,336993701	0,369	0,195	1
RABEP1	0,004149227	0,345276136	0,338	0,176	1
HIP1	0,004252063	0,331245787	0,523	0,293	1
PLEKHA3	0,004286643	0,532311978	0,277	0,132	1
YIPF6	0,004328061	0,311355246	0,431	0,239	1
FOXP1	0,00435731	0,260117375	0,892	0,707	1
ARL8B	0,004409346	0,256962923	0,323	0,166	1

CLEC14A	0,004453111	0,259256325	0,769	0,541	1
TIMP2	0,004625719	0,346275288	0,8	0,615	1
AFDN	0,004651692	0,283791422	0,585	0,376	1
SCARA3	0,004704462	0,451198885	0,446	0,259	1
ERAP2	0,004835717	0,361836856	0,508	0,327	1
ITSN2	0,004875902	0,342323513	0,431	0,254	1
DYNC1LI2	0,004900988	0,251462714	0,508	0,322	1
MXD4	0,004926511	0,405634288	0,462	0,278	1
NR3C1	0,004952561	0,3497085	0,662	0,415	1
AHR	0,004963987	0,284166558	0,554	0,341	1
B4GALT1	0,005031221	0,325168782	0,523	0,317	1
CRK	0,005075413	0,264754013	0,523	0,302	1
IL10RB	0,005140992	0,295243343	0,338	0,176	1
RHEBP1	0,005215028	0,376972932	0,508	0,298	1
REX1BD	0,005224088	0,258959167	0,323	0,166	1
MAST4	0,005243029	0,359281425	0,615	0,42	1
PCDH7	0,005247898	0,373210416	0,492	0,278	1
SLC25A23	0,00525423	0,306169212	0,262	0,117	1
SDC3	0,005320376	0,267902458	0,323	0,161	1
DSTYK	0,005337587	0,440878918	0,385	0,195	1
PPDPF	0,005375235	0,373281618	0,769	0,624	1
HMG20B	0,005583113	0,275713128	0,323	0,161	1
RUBCN	0,005645967	0,303417827	0,262	0,117	1
IDH3B	0,005787263	0,306908317	0,277	0,137	1
TOB2	0,006076769	0,336259669	0,4	0,229	1
TSN	0,00607984	0,268056398	0,354	0,185	1
RAB27A	0,006188556	0,377938191	0,492	0,293	1
RAP1B	0,00629554	0,354683702	0,585	0,38	1
RNF40	0,006478959	0,356164548	0,262	0,122	1
SCPEP1	0,006785772	0,37412521	0,292	0,146	1
PRDX2	0,006803475	0,285491647	0,4	0,229	1
LSM12	0,006834676	0,291551446	0,323	0,166	1
NDUFB9	0,006849016	0,378588891	0,462	0,283	1
ATP1A1	0,006986015	0,250612374	0,708	0,512	1
MAP2	0,007019752	0,350893606	0,262	0,127	1
TMEM173	0,007052936	0,34613378	0,692	0,478	1
TPST2	0,007564868	0,25924742	0,277	0,132	1
TSC2	0,007906906	0,299275086	0,292	0,141	1
HSD17B4	0,007915825	0,256567809	0,292	0,146	1
FXR1	0,00803475	0,262185544	0,369	0,205	1
SEC61G	0,008117575	0,265117307	0,585	0,38	1
STAT3	0,00812188	0,282321557	0,677	0,478	1
MTCH1	0,008318328	0,292004811	0,538	0,361	1
LUC7L	0,008433602	0,280186958	0,308	0,156	1
TPRG1L	0,008558273	0,386452963	0,277	0,141	1
PDLIM1	0,008566849	0,261257398	0,923	0,756	1
INSIG1	0,008641242	0,335419896	0,292	0,141	1
ITGA10	0,00882103	0,262221122	0,523	0,322	1
SELENOW	0,009017479	0,371970461	0,615	0,463	1
NT5E	0,009544712	0,407775174	0,385	0,224	1
NCSTN	0,009589063	0,275436737	0,431	0,263	1
TNRC6C	0,009760787	0,284346365	0,538	0,337	1
TMEM109	0,009764402	0,277378646	0,615	0,424	1
KIDINS220	0,009787757	0,252889593	0,446	0,263	1

Cluster E.3 marker genes

gene	p_val	avg_logFC	pct.1	pct.2	p_val_adj
NOTCH3	3,00E-33	2,555940177	0,853	0,068	5,68E-29
LMOD1	1,48E-32	2,07784166	0,706	0,025	2,80E-28
TBX2	3,57E-32	1,774095728	0,618	0,008	6,77E-28
GUCY1A1	3,21E-31	2,246156368	0,941	0,123	6,07E-27
PDGFRB	4,89E-30	1,864265922	0,794	0,059	9,27E-26
AGT	1,63E-29	2,141553043	0,765	0,059	3,08E-25
ITGA7	3,73E-29	1,403717706	0,529	0,004	7,06E-25
MYH11	3,47E-28	3,244636411	0,882	0,131	6,57E-24
RGS5	1,89E-27	3,04989386	0,912	0,161	3,59E-23
PLN	2,13E-27	1,494823093	0,559	0,013	4,03E-23
C11orf96	5,14E-27	2,471514518	0,912	0,14	9,74E-23
ANTXR1	2,93E-26	1,672678473	0,765	0,068	5,56E-22
PDE5A	9,10E-26	1,418475461	0,676	0,047	1,73E-21
ASPN	9,92E-26	1,746695599	0,676	0,047	1,88E-21
COL14A1	2,22E-24	2,002623227	0,824	0,123	4,22E-20
MFAP4	2,64E-24	2,338670752	0,676	0,059	5,00E-20
ADH1B	8,70E-24	2,050600633	0,735	0,076	1,65E-19
SOD3	1,67E-23	2,054738099	0,706	0,076	3,17E-19
FXYD1	3,39E-23	1,428440893	0,588	0,034	6,42E-19
LTBP1	4,50E-23	2,25832626	0,853	0,169	8,52E-19
PARM1	2,28E-22	1,444772298	0,441	0,008	4,32E-18
PRRX1	9,75E-22	1,208506328	0,618	0,047	1,85E-17
COL1A1	2,35E-21	1,395743833	0,765	0,102	4,45E-17
MYLK	6,31E-21	1,793582394	0,794	0,14	1,19E-16
CSPG4	6,44E-21	1,094402457	0,441	0,013	1,22E-16
CNN1	1,16E-20	1,386608082	0,412	0,008	2,20E-16
HEYL	3,45E-20	1,507107183	0,471	0,021	6,53E-16
TAGLN	1,26E-19	2,759310337	0,912	0,288	2,38E-15
CAMK2N1	2,07E-19	1,178039036	0,529	0,038	3,93E-15
MRVI1	6,64E-19	1,138714439	0,471	0,025	1,26E-14
MYL9	6,68E-19	2,48845884	0,912	0,322	1,27E-14
THBS2	8,82E-19	2,116638201	0,735	0,127	1,67E-14
SMOC2	2,16E-18	1,332498423	0,441	0,021	4,09E-14
MXRA8	4,09E-18	1,097454229	0,441	0,021	7,75E-14
HSPB6	4,74E-18	2,039142647	0,647	0,093	8,99E-14
CALD1	4,82E-18	1,448258052	1	0,644	9,13E-14
AEBP1	1,21E-17	1,930771238	0,853	0,25	2,30E-13
SPECC1	1,53E-17	0,828195772	0,382	0,013	2,90E-13
ANGPT1	5,85E-17	1,125824209	0,412	0,021	1,11E-12
EFHD1	6,32E-17	1,098713858	0,471	0,034	1,20E-12
COL6A1	1,31E-16	1,554595064	0,912	0,339	2,48E-12
TPM2	1,68E-16	1,99389318	0,853	0,297	3,19E-12
ACTA2	2,03E-16	3,218500534	0,824	0,288	3,86E-12
GEM	2,04E-16	1,080002512	0,5	0,042	3,87E-12
LRP1	2,27E-16	1,204775783	0,706	0,123	4,31E-12
LGALS3BP	2,61E-16	0,808250113	0,441	0,03	4,95E-12
SDC2	5,31E-16	1,362510389	0,618	0,089	1,01E-11
VCAN	5,60E-16	1,984375816	0,794	0,208	1,06E-11
ADGRL3	8,20E-16	0,911637413	0,353	0,013	1,55E-11
APOE	9,45E-16	2,800900126	0,647	0,131	1,79E-11
ITGA8	1,72E-15	1,173524199	0,441	0,034	3,27E-11
PCOLCE	1,72E-15	1,261639082	0,588	0,081	3,27E-11
ACTG2	2,04E-15	0,956514406	0,294	0,004	3,86E-11

LRRC17	2,81E-15	1,032199999	0,471	0,047	5,32E-11
OLFML2B	2,88E-15	1,169449973	0,5	0,051	5,46E-11
NOV	5,75E-15	1,467287874	0,559	0,081	1,09E-10
RGS4	5,97E-15	0,987352375	0,353	0,017	1,13E-10
PHLDA1	7,58E-15	1,123086546	0,529	0,068	1,44E-10
PALLD	1,13E-14	1,572548629	0,765	0,203	2,15E-10
MAP9	1,35E-14	0,879111423	0,382	0,025	2,57E-10
ANO1	1,88E-14	1,002819862	0,382	0,025	3,57E-10
WFDC1	1,89E-14	1,259243657	0,412	0,034	3,59E-10
PDLIM3	2,13E-14	1,563389954	0,706	0,174	4,04E-10
FAT1	4,16E-14	0,878889231	0,294	0,008	7,88E-10
FRZB	9,50E-14	0,756592279	0,471	0,051	1,80E-09
NTRK3	1,05E-13	1,272117991	0,382	0,03	1,99E-09
PRKG1	1,28E-13	1,061090231	0,382	0,03	2,42E-09
SYNPO2	1,42E-13	1,354620081	0,559	0,089	2,70E-09
PDGFRA	1,74E-13	0,903600079	0,382	0,03	3,30E-09
ATP1A2	1,80E-13	1,013546498	0,412	0,038	3,41E-09
SSPN	3,48E-13	0,992317226	0,471	0,059	6,59E-09
COL6A2	4,73E-13	1,446554539	0,824	0,322	8,97E-09
NDUFA4L2	6,72E-13	0,892549043	0,353	0,025	1,27E-08
AXL	7,88E-13	0,895830371	0,353	0,025	1,49E-08
VASN	1,12E-12	0,72810631	0,353	0,025	2,13E-08
ISLR	1,45E-12	1,057482216	0,529	0,081	2,74E-08
RCAN2	1,90E-12	1,255942988	0,5	0,076	3,60E-08
GUCY1B1	3,32E-12	0,737616257	0,324	0,021	6,29E-08
IGFBP5	3,51E-12	1,035207674	0,941	0,449	6,66E-08
COL1A2	6,21E-12	1,23628215	0,824	0,284	1,18E-07
SLIT1	7,82E-12	1,396127132	0,5	0,089	1,48E-07
TBX18	9,10E-12	0,606573767	0,294	0,017	1,72E-07
SORBS1	9,62E-12	1,058057165	0,441	0,059	1,82E-07
PRELP	1,24E-11	1,364690345	0,912	0,492	2,35E-07
FHL1	1,38E-11	1,486819303	0,794	0,343	2,61E-07
MAP1B	1,67E-11	1,382565944	0,765	0,297	3,17E-07
EDNRA	3,47E-11	0,645199834	0,265	0,013	6,58E-07
EMILIN1	3,88E-11	1,164345592	0,559	0,127	7,36E-07
ZFHX3	4,41E-11	1,265282581	0,765	0,297	8,37E-07
ID4	5,47E-11	0,890717842	0,441	0,064	1,04E-06
CPED1	6,89E-11	1,053755543	0,353	0,038	1,31E-06
SUSD5	7,69E-11	0,882005625	0,294	0,021	1,46E-06
MFGE8	1,02E-10	1,427616239	0,706	0,242	1,93E-06
TNS1	1,24E-10	1,317137413	0,824	0,369	2,34E-06
OGN	1,31E-10	1,484300722	0,824	0,373	2,49E-06
ADGRE5	1,35E-10	0,569872468	0,324	0,03	2,55E-06
ARHGEF17	2,23E-10	1,072163731	0,412	0,064	4,23E-06
AKAP12	2,58E-10	1,189322807	0,5	0,102	4,88E-06
NT5DC2	2,64E-10	0,875395911	0,382	0,051	5,01E-06
ECM2	2,67E-10	0,913780337	0,559	0,123	5,06E-06
FLNA	2,71E-10	1,453022708	0,853	0,678	5,13E-06
TPM1	3,19E-10	1,098189877	0,824	0,343	6,05E-06
INHBA	3,49E-10	1,184518848	0,324	0,034	6,60E-06
S100A4	4,08E-10	1,319425007	0,853	0,453	7,73E-06
S1PR3	4,14E-10	0,778267424	0,294	0,025	7,84E-06
DSTN	4,55E-10	1,24418714	0,941	0,644	8,62E-06
TTLL7	4,97E-10	0,665353494	0,294	0,025	9,42E-06
C2orf40	5,97E-10	0,650341566	0,294	0,025	1,13E-05
CLMN	5,97E-10	0,594063227	0,294	0,025	1,13E-05

CRYAB	6,10E-10	0,98580488	0,588	0,153	1,16E-05
PPP1R14A	6,56E-10	1,197767319	0,559	0,157	1,24E-05
ITGA1	7,83E-10	1,052600965	0,471	0,093	1,48E-05
EDIL3	8,65E-10	0,730852414	0,324	0,034	1,64E-05
DMD	1,05E-09	1,145949828	0,441	0,081	1,99E-05
EBF1	1,10E-09	1,311408201	0,529	0,136	2,09E-05
FMO2	1,36E-09	1,322295694	0,441	0,081	2,57E-05
LOXL1	1,88E-09	0,957827909	0,353	0,051	3,56E-05
COL18A1	3,70E-09	1,376310593	0,794	0,479	7,02E-05
MYH10	3,73E-09	1,493654664	0,647	0,237	7,06E-05
KITLG	3,73E-09	0,713665687	0,382	0,059	7,07E-05
TGFB1I1	4,45E-09	0,857249392	0,647	0,212	8,43E-05
PPP1R12B	7,72E-09	1,329342299	0,412	0,081	0,000146255
COL12A1	1,16E-08	0,649406052	0,382	0,059	0,000218993
RNF24	1,35E-08	0,948629234	0,412	0,081	0,000255939
LGALS1	1,85E-08	0,995304509	0,824	0,483	0,000350714
CDC14B	1,91E-08	0,550354895	0,294	0,034	0,000361188
ITGA11	2,13E-08	0,462740096	0,265	0,025	0,00040342
CSRP2	2,26E-08	0,754420343	0,324	0,047	0,000429209
SPEG	2,50E-08	0,767659018	0,412	0,085	0,000472902
MYO1B	3,18E-08	0,758240089	0,382	0,068	0,000602107
DKK3	5,32E-08	1,122140069	0,676	0,288	0,001007663
PDE1A	7,31E-08	0,822793794	0,324	0,051	0,001385413
MICAL2	1,23E-07	1,188520513	0,647	0,305	0,002328229
BCL6	1,31E-07	0,624749463	0,324	0,055	0,002479176
FBLN1	1,76E-07	0,914575717	0,382	0,081	0,003337722
KANK2	1,95E-07	0,962155602	0,559	0,182	0,003703582
NFATC4	2,14E-07	0,65724737	0,294	0,042	0,004058521
LPP	2,24E-07	1,015203502	0,853	0,64	0,004250248
MTFMT	2,28E-07	0,466694538	0,265	0,034	0,004312148
SPARCL1	2,51E-07	0,83161768	0,941	0,669	0,004756925
ARHGEF10L	2,72E-07	0,960591215	0,294	0,047	0,005150038
FAM129A	2,77E-07	0,783622054	0,618	0,22	0,005257679
AOC3	2,98E-07	0,538019414	0,382	0,076	0,005656027
ABCB10	3,06E-07	0,381448514	0,265	0,034	0,005805444
ROR1	3,28E-07	0,634033929	0,294	0,047	0,006214716
PKDCC	4,76E-07	0,604676959	0,294	0,047	0,009016981
SELENBP1	5,13E-07	0,711929817	0,441	0,119	0,009729539
RAB23	6,47E-07	0,467996369	0,265	0,038	0,012267389
ISYNA1	7,71E-07	0,787451323	0,471	0,144	0,014606328
CHPF	1,30E-06	0,571800465	0,265	0,042	0,02463759
LGALSL	1,40E-06	0,64911781	0,353	0,076	0,026594147
GGTA1P	1,48E-06	0,513411515	0,382	0,089	0,028110801
GPRASP1	1,52E-06	0,614882468	0,265	0,042	0,028896806
SORT1	1,57E-06	0,900732399	0,5	0,169	0,029739095
CDK5RAP2	1,58E-06	0,583529855	0,441	0,127	0,02993315
SLC40A1	1,64E-06	1,157905212	0,559	0,246	0,031162815
ITGBL1	1,75E-06	0,498743892	0,412	0,102	0,033095813
NPNT	2,49E-06	0,603620136	0,324	0,068	0,047203928
MCAM	2,57E-06	1,062338512	0,735	0,496	0,048789518
FBXO32	2,66E-06	0,672517036	0,324	0,068	0,050440594
CARMN	2,71E-06	1,134188807	0,471	0,153	0,05135351
ANGPT2	2,76E-06	0,341689889	0,471	0,131	0,052381343
FN1	2,78E-06	0,711854611	0,941	0,686	0,05273239
SNX9	3,37E-06	0,677731774	0,706	0,331	0,063829346
OAT	3,90E-06	0,933044137	0,588	0,233	0,073938918

ZEB2	4,75E-06	0,744905258	0,412	0,119	0,090064213
CYFIP2	5,69E-06	0,711452489	0,294	0,059	0,107788302
FBLN5	6,55E-06	0,666586492	0,647	0,271	0,124053249
FSTL1	6,83E-06	0,745147423	0,824	0,53	0,129520567
SLC14A1	7,09E-06	0,643049885	0,324	0,076	0,134273494
TGFBI	7,14E-06	0,804103001	0,265	0,051	0,135294702
LTBP4	9,69E-06	0,834125505	0,735	0,394	0,183587916
NOTCH2	1,32E-05	0,616151761	0,324	0,081	0,249463393
PTPRS	1,42E-05	0,675261661	0,412	0,131	0,268468706
C1orf198	1,44E-05	0,951301324	0,471	0,174	0,27254005
MAPRE2	1,48E-05	0,842969786	0,706	0,394	0,281278106
MSL2	1,53E-05	0,324535145	0,324	0,076	0,289042009
ADIRF	1,67E-05	0,753704374	0,853	0,661	0,317349563
PGF	1,99E-05	0,624415276	0,294	0,068	0,376835107
MT2A	2,18E-05	0,713165112	0,706	0,36	0,412166734
CRIP1	2,23E-05	0,644156314	0,735	0,432	0,42334451
NR2F2	2,43E-05	0,448103844	0,941	0,534	0,460963024
SVIL	2,49E-05	0,608616785	0,529	0,216	0,471429468
PIK3R2	2,57E-05	0,50830977	0,265	0,055	0,486240548
GADD45A	3,16E-05	0,620702277	0,471	0,178	0,598500186
ITIH5	3,28E-05	0,685065526	0,412	0,131	0,621687167
CCDC3	3,61E-05	0,797060914	0,588	0,258	0,683369961
RBPMS	3,64E-05	0,748994264	0,647	0,36	0,688853811
GGT5	3,65E-05	0,820786909	0,382	0,127	0,691255969
MAP3K20	3,75E-05	0,76441888	0,588	0,288	0,711504554
KCNMB1	3,90E-05	0,614986675	0,294	0,072	0,739426368
FILIP1L	4,00E-05	1,108856415	0,441	0,174	0,757582401
PLS3	4,15E-05	0,724603718	0,735	0,5	0,787188713
PNPLA3	4,41E-05	0,390806471	0,265	0,055	0,834785617
COL3A1	4,72E-05	0,459407599	0,706	0,377	0,895088808
LRRC32	4,88E-05	0,767292639	0,5	0,216	0,925484306
PDE3A	6,22E-05	0,798064856	0,5	0,22	1
UBA2	6,36E-05	0,757978231	0,471	0,208	1
KCNK3	7,31E-05	0,41391224	0,412	0,14	1
SBF2-AS1	8,28E-05	0,521921205	0,618	0,28	1
MZF1	8,80E-05	0,56655159	0,265	0,064	1
IFT22	9,09E-05	0,428241908	0,294	0,076	1
NUBPL	9,86E-05	0,588292831	0,471	0,182	1
GLIPR1	0,000101615	0,593198219	0,353	0,114	1
FBLIM1	0,00010287	0,568738476	0,824	0,547	1
SGIP1	0,00011142	0,433454551	0,265	0,064	1
ITGB5	0,000113217	0,711220211	0,5	0,22	1
DST	0,000116876	0,690172379	0,647	0,381	1
PCYOX1	0,000121102	0,633897039	0,618	0,326	1
CST3	0,000128735	0,524673015	0,912	0,75	1
PDK4	0,000137342	0,98701086	0,5	0,208	1
FAM227A	0,000145227	0,609816306	0,471	0,195	1
BGN	0,000149383	0,739780633	0,912	0,712	1
SERPING1	0,000152426	0,583365795	0,853	0,487	1
GULP1	0,000169223	0,557590517	0,294	0,085	1
PFN2	0,000169964	0,769927218	0,382	0,127	1
PCDH7	0,00017268	0,652467329	0,588	0,292	1
FKBP7	0,000179385	0,480950818	0,412	0,153	1
CAVIN3	0,000182507	0,575663744	0,588	0,314	1
PRDM7	0,000222894	0,625985173	0,412	0,161	1
LHFPL6	0,000237933	0,703807737	0,588	0,318	1

LOC646214	0,000257305	0,491026975	0,412	0,157	1
ARRDC3-AS1	0,000264958	0,669162887	0,529	0,254	1
ERRFI1	0,000278164	0,571251909	0,294	0,089	1
SPARC	0,000296154	0,620178872	0,853	0,763	1
PMEPA1	0,000296813	0,667462927	0,559	0,305	1
MT1E	0,000297834	0,576065899	0,353	0,119	1
PRICKLE2	0,000303691	0,568661195	0,265	0,072	1
SERPINB8	0,000308804	0,593440139	0,265	0,076	1
PPP1R12A	0,000339267	0,478670668	0,618	0,343	1
ECHDC2	0,000344041	0,455802042	0,294	0,089	1
sep-07	0,00034682	0,732047392	0,735	0,5	1
CEACAMP1	0,00034884	0,362206181	0,412	0,144	1
ZNF480	0,000349747	0,62278193	0,765	0,504	1
PQLC2	0,000355387	0,587244656	0,294	0,089	1
IGFBP7	0,000364878	0,504996274	0,941	0,894	1
SGCB	0,000410609	0,747428923	0,382	0,157	1
GLIPR2	0,000418252	0,496262916	0,294	0,097	1
POLR2I	0,000446714	0,509576371	0,382	0,148	1
PKD1	0,000446963	0,638173871	0,441	0,186	1
H2AFJ	0,000447702	0,366586877	0,353	0,127	1
CBX7	0,000448703	0,550170395	0,5	0,246	1
FAM118A	0,000459928	0,497683325	0,324	0,106	1
CX3CL1	0,000492714	0,461326598	0,441	0,186	1
FAM20B	0,000513203	0,464029272	0,471	0,203	1
CCS	0,000525435	0,492289269	0,265	0,081	1
ZXDC	0,000535911	0,587423234	0,529	0,271	1
MRPL36	0,000552663	0,774255383	0,412	0,174	1
CARF	0,000560877	0,459542492	0,265	0,076	1
CSRP3	0,000570565	0,642984661	0,529	0,288	1
HSPA1A	0,000571023	0,34531577	0,618	0,318	1
LINC00672	0,000579922	0,390262507	0,265	0,076	1
C1QTNF1	0,00058719	0,795980715	0,5	0,258	1
ANP32A-IT1	0,000609621	0,484829277	0,265	0,076	1
LINC00294	0,000644574	0,645563787	0,382	0,148	1
COL4A2	0,000654697	0,54021387	0,735	0,504	1
MXI1	0,000658855	0,457218723	0,412	0,182	1
ZNF618	0,000665433	0,659085095	0,324	0,114	1
MFAP2	0,000673041	0,428497702	0,294	0,093	1
TSIX	0,00070147	0,335705693	0,765	0,517	1
LOC286437	0,00071448	0,587816795	0,618	0,356	1
NME4	0,000718058	0,426970806	0,412	0,165	1
HK1	0,000720417	0,569835386	0,5	0,258	1
ZNF829	0,000778414	0,535451145	0,412	0,174	1
FLJ42627	0,000814745	0,347709629	0,471	0,203	1
MTG2	0,000841743	0,388714909	0,265	0,081	1
ALPK1	0,000852586	0,371802718	0,471	0,203	1
GLG1	0,000865553	0,592500545	0,676	0,483	1
CD82	0,000894268	0,381808679	0,294	0,097	1
TPCN1	0,000914402	0,831181985	0,294	0,102	1
CBR1	0,00091536	0,514067318	0,265	0,085	1
PLEKHG2	0,000926143	0,367209006	0,265	0,081	1
ZNF551	0,000948677	0,544174546	0,294	0,097	1
BRAT1	0,000952899	0,324769412	0,294	0,093	1
MXRA7	0,000972711	0,725859403	0,618	0,381	1
GJC1	0,000986886	0,666633186	0,471	0,212	1
PDE6A	0,000990763	0,62709257	0,618	0,309	1

NDRG2	0,000997733	0,481283411	0,294	0,102	1
SGCD	0,001002397	0,38572019	0,265	0,081	1
DSEL	0,001006138	0,431078026	0,294	0,097	1
USP11	0,001018954	0,625939617	0,412	0,186	1
GJA4	0,001039595	0,616324969	0,324	0,119	1
SKP1	0,001104587	0,442881894	0,794	0,564	1
CHURC1	0,001158536	0,671333478	0,647	0,39	1
SH3BGRL	0,00118115	0,55146669	0,676	0,475	1
SC5D	0,001203705	0,595028846	0,294	0,106	1
DTX3	0,001220247	0,464360706	0,294	0,102	1
MID1IP1	0,001220798	0,654132956	0,294	0,106	1
TPTE2P1	0,001225159	0,620775477	0,559	0,305	1
VCL	0,001227309	0,725120074	0,618	0,453	1
GNAL	0,001238115	0,563791323	0,294	0,106	1
PHC3	0,001356978	0,429795705	0,5	0,237	1
LPAL2	0,001368568	0,580479035	0,353	0,14	1
ITGA3	0,001431177	0,627615582	0,471	0,25	1
ANKRD20A9P	0,00147167	0,451183424	0,794	0,623	1
ABI2	0,001498646	0,701548258	0,5	0,275	1
LOC100131257	0,001543741	0,332544786	1	0,907	1
SYNM	0,001568792	0,551641543	0,353	0,144	1
RTL8C	0,001728966	0,424418909	0,676	0,419	1
ANKLE1	0,001749683	0,557602017	0,324	0,119	1
SELENOM	0,00177594	0,753534403	0,618	0,419	1
PPP1CB	0,001788577	0,599278586	0,706	0,517	1
ODF2L	0,001801438	0,440775401	0,882	0,725	1
EMC10	0,001846088	0,539635416	0,382	0,174	1
HEXIM1	0,001858858	0,691367831	0,412	0,186	1
GYPC	0,001892057	0,315768169	0,324	0,119	1
ADH5	0,001893946	0,513201567	0,441	0,229	1
NEXN	0,001946573	0,673001964	0,324	0,123	1
FOSB	0,002051516	0,586081001	0,794	0,61	1
INAFM1	0,002060201	0,612153749	0,412	0,199	1
TEKT4P2	0,002073769	0,727461934	0,324	0,14	1
PLAC9	0,002090426	0,628279618	0,353	0,148	1
RABGAP1	0,002164296	0,451708298	0,529	0,271	1
SASH1	0,002240324	0,550977083	0,647	0,513	1
IDS	0,002384796	0,448556012	0,706	0,53	1
LAMA5	0,002430684	0,662699954	0,618	0,335	1
ZNF503	0,002555968	0,414090947	0,353	0,148	1
FXN	0,002569292	0,326297711	0,382	0,161	1
P3H4	0,002570239	0,476994264	0,324	0,136	1
POLH	0,002578636	0,409262336	0,471	0,233	1
CTC1	0,002690376	0,286947695	0,265	0,089	1
VSTM4	0,002741713	0,367250254	0,353	0,148	1
RAB11FIP2	0,002802905	0,310240125	0,294	0,114	1
SIGMAR1	0,002878547	0,517766874	0,382	0,182	1
HCG18	0,002901337	0,299320826	0,647	0,411	1
S100A6	0,003092637	0,336493978	0,971	0,911	1
PACSIN2	0,003146118	0,690429946	0,382	0,203	1
PCGF5	0,003147216	0,474872776	0,529	0,305	1
PLEKHM3	0,003185276	0,562612018	0,294	0,114	1
PDXDC2P	0,003214468	0,337495183	0,353	0,144	1
KANK1	0,003224709	0,721376831	0,353	0,161	1
YAP1	0,003265987	0,651423597	0,412	0,225	1
MIR100HG	0,003294391	0,422046079	0,265	0,093	1

ACADSB	0,003367285	0,296308602	0,618	0,356	1
EPS8	0,003433716	0,735732537	0,441	0,237	1
CHCHD10	0,003460736	0,633583023	0,353	0,169	1
TSC22D1	0,003506711	0,479612369	0,588	0,398	1
ZMYM2	0,003512738	0,459812405	0,382	0,174	1
ZCCHC8	0,003626677	0,562445197	0,265	0,102	1
DVL3	0,003633811	0,293262729	0,382	0,169	1
KLHL42	0,003800995	0,584868616	0,382	0,182	1
ZNHIT6	0,003903566	0,467647986	0,324	0,14	1
SLC6A20	0,003945801	0,309242192	0,294	0,114	1
PWWP2A	0,004028803	0,428491192	0,265	0,102	1
MTHFD1L	0,004047633	0,613465324	0,324	0,153	1
ILF3-DT	0,004083299	0,428459508	0,353	0,157	1
SZRD1	0,004378022	0,491352092	0,441	0,242	1
BICD2	0,004814057	0,360650805	0,353	0,157	1
MID1	0,004978544	0,64808453	0,324	0,136	1
NKAIN3-IT1	0,004980688	0,706387911	0,412	0,22	1
MBD1	0,005316836	0,587618059	0,529	0,364	1
IGFBP6	0,005650756	0,463756174	0,353	0,165	1
GNRHR2	0,005702535	0,259882957	0,265	0,102	1
TNS3	0,005706037	0,483211645	0,324	0,153	1
ZNF430	0,005772402	0,284862009	0,294	0,119	1
LOC440300	0,005815672	0,463455672	0,324	0,14	1
ADAMTS1	0,005823282	0,497642189	0,618	0,36	1
PJA2	0,005963392	0,393570876	0,618	0,411	1
RAB34	0,005969344	0,477427392	0,353	0,169	1
LAMA4	0,005981525	0,375433136	0,618	0,403	1
CALM2	0,005998174	0,485205898	0,824	0,729	1
CEBPD	0,006015702	0,732186499	0,441	0,242	1
GPX3	0,006082203	0,640642206	0,5	0,339	1
C12orf57	0,006135182	0,52526769	0,559	0,347	1
NELFE	0,006275254	0,506711807	0,324	0,148	1
CYTH3	0,006306296	0,361888352	0,294	0,127	1
CDH13	0,006392686	0,404281332	0,618	0,377	1
LAMC1	0,00658936	0,553736671	0,559	0,356	1
GSTP1	0,006914125	0,502750817	0,706	0,483	1
PHLDB1	0,007024772	0,42175171	0,412	0,233	1
CDKN1B	0,007102686	0,426376283	0,382	0,182	1
JAM3	0,007108675	0,291820193	0,353	0,165	1
TGFBR1	0,007231378	0,381225536	0,324	0,153	1
LINC00550	0,0073688	0,558004919	0,294	0,136	1
OPA3	0,007735094	0,496410527	0,471	0,284	1
CREBBP	0,007770123	0,367564233	0,441	0,233	1
PATJ	0,007787519	0,361379934	0,353	0,161	1
FMOD	0,007885778	0,432007634	0,382	0,199	1
PEBP1	0,007895108	0,474385826	0,794	0,636	1
AP4S1	0,008063268	0,517128456	0,412	0,22	1
SUN2	0,008078872	0,593237715	0,647	0,441	1
BAG1	0,008190334	0,416817244	0,412	0,225	1
C2CD2	0,008193761	0,394574134	0,294	0,127	1
ASB8	0,008204128	0,307100899	0,353	0,165	1
LINC00346	0,008204128	0,28890867	0,353	0,165	1
RDH11	0,00827704	0,368419798	0,441	0,233	1
DAAM1	0,008786266	0,462656294	0,471	0,267	1
PLP2	0,008947404	0,432956524	0,471	0,263	1
CD63	0,009016622	0,389463899	0,882	0,737	1

TCF7L2	0,009037184	0,328506789	0,529	0,309	1
CCL2	0,009048909	0,367013925	0,353	0,174	1
SQSTM1	0,009275983	0,527486511	0,735	0,542	1
KLRD1	0,009382131	0,566920304	0,294	0,131	1
DCAF6	0,00950702	0,416106491	0,353	0,174	1
CEBPB	0,009746976	0,463488113	0,382	0,195	1
ZBTB20	0,009871606	0,331237882	0,5	0,297	1
IRF2BPL	0,009961235	0,37401393	0,382	0,191	1
TMEM47	0,009973175	0,479652467	0,559	0,39	1
COPS2	0,009996057	0,305940237	0,412	0,225	1

Cluster SMC.0 marker genes

gene	p_val	avg_logFC	pct.1	pct.2	p_val_adj
CFH	2,76E-11	2,081330795	0,784	0,262	5,23E-07
LUM	3,80E-10	2,717296054	0,631	0,048	7,20E-06
LTBP2	2,95E-08	1,346764737	0,838	0,548	0,000559497
COL15A1	4,66E-07	1,540759098	0,514	0,071	0,008837441
CCDC80	5,40E-07	2,130050107	0,721	0,357	0,010226229
COL1A2	7,98E-07	1,540023737	0,892	0,762	0,015127301
TIMP1	1,65E-06	1,169451637	0,91	0,833	0,031176984
F2R	2,11E-06	1,203316647	0,559	0,119	0,039993627
AEBP1	2,60E-06	0,87270135	0,838	0,786	0,049260422
VCAN	3,79E-06	1,012380812	0,874	0,762	0,071821374
MGP	3,80E-06	1,236712388	0,982	0,976	0,072071979
DCN	5,15E-06	2,251521435	0,405	0,024	0,097504167
ISLR	5,84E-06	0,990586681	0,613	0,19	0,110669291
FBLN2	6,06E-06	1,399293483	0,505	0,119	0,114800217
UNC5B	7,56E-06	1,068881573	0,532	0,119	0,143175459
COL3A1	8,30E-06	1,934975813	0,82	0,595	0,157349854
C1S	1,01E-05	0,792739656	0,829	0,595	0,190889774
SERPINE2	1,24E-05	1,53248481	0,351	0	0,235444825
PRSS23	3,57E-05	0,991499158	0,658	0,238	0,676154785
POSTN	3,60E-05	2,226540779	0,414	0,071	0,682489706
SFRP2	4,47E-05	2,202828951	0,315	0	0,847594291
C1R	6,23E-05	0,870532333	0,829	0,643	1
OMD	6,90E-05	1,115524147	0,622	0,31	1
SFRP4	7,24E-05	1,549553142	0,468	0,143	1
SULF1	8,47E-05	0,80260227	0,766	0,429	1
LAMB1	8,82E-05	0,880296339	0,514	0,167	1
PRELP	0,000113404	0,759014013	0,901	0,857	1
BGN	0,000122196	0,71508872	0,919	0,952	1
COL6A3	0,000128692	1,33782559	0,541	0,19	1
COL6A2	0,00013252	0,699958764	0,856	0,762	1
FBLN1	0,000141457	1,202677855	0,577	0,262	1
CST3	0,000148783	0,62393661	0,829	0,881	1
IGFBP6	0,000153963	1,430505373	0,559	0,262	1
ADD3	0,000157065	0,625776762	0,559	0,19	1
SPRY1	0,000184711	0,647048214	0,459	0,119	1
CHPF	0,000203198	0,861149335	0,414	0,095	1
FN1	0,000246816	0,552191676	0,937	0,952	1
MMP2	0,000268652	0,961075464	0,559	0,238	1
MFAP2	0,000275011	0,717855405	0,261	0	1
ZCCHC24	0,000293338	0,795710212	0,441	0,143	1
GSN	0,00030572	1,020739723	0,901	0,857	1
THBS2	0,000316801	0,586370691	0,847	0,595	1
CRTAC1	0,000338055	1,043000828	0,306	0,024	1
TGFBR2	0,000341301	0,708943065	0,495	0,167	1
SCAF1	0,000459977	0,589839616	0,297	0,024	1
PCOLCE2	0,000471831	1,146342418	0,459	0,19	1
PCOLCE	0,000483556	0,79997394	0,631	0,333	1
SDF4	0,000649182	0,497155658	0,541	0,214	1
LAMA2	0,000664121	0,638057238	0,459	0,143	1
MXRA8	0,000678732	0,86752248	0,622	0,405	1
COL16A1	0,000687868	0,738612141	0,369	0,095	1
NPY1R	0,000797587	0,858509982	0,297	0,048	1
SERPINE1	0,000810044	1,516487708	0,45	0,19	1

ITM2B	0,000860894	0,449771124	0,919	0,976	1
MARCKS	0,001109576	0,685518148	0,649	0,357	1
CD59	0,001245927	0,572088875	0,739	0,619	1
CDH11	0,001284964	0,632683886	0,333	0,071	1
S100A10	0,001298226	0,811430154	0,685	0,452	1
PLAT	0,00148716	0,686070827	0,288	0,048	1
EFEMP1	0,001519027	0,572695053	0,856	0,738	1
GAP43	0,001525613	0,85412218	0,36	0,095	1
NF1	0,001546156	0,515724392	0,369	0,095	1
FXYD5	0,001564788	0,695444367	0,532	0,286	1
TFPI2	0,001594807	1,199723364	0,279	0,048	1
COL1A1	0,001714952	1,492836852	0,793	0,548	1
CCL5	0,001821021	1,109132229	0,315	0,071	1
MRC2	0,001911631	0,544645892	0,532	0,238	1
ATP1A1	0,001960144	0,471120064	0,459	0,167	1
PLXDC2	0,002033131	0,66417267	0,27	0,048	1
CRLF1	0,002072798	0,980139525	0,27	0,048	1
PMP22	0,002115407	0,612173946	0,495	0,238	1
CTSF	0,002127542	0,596864141	0,45	0,167	1
FHL2	0,002194878	0,66040101	0,378	0,119	1
GGT5	0,002292067	0,807984468	0,333	0,095	1
SPTBN1	0,002330513	0,461775495	0,784	0,643	1
MT2A	0,002413505	0,770461961	0,649	0,452	1
MEGF6	0,002643425	0,5701758	0,369	0,119	1
DAP	0,002665017	0,558268726	0,495	0,214	1
EMP1	0,002754821	0,638812415	0,387	0,143	1
CTSB	0,002886482	0,50746826	0,739	0,429	1
HLA-A	0,003150727	0,577606674	0,829	0,786	1
TPM3	0,003263346	0,635696101	0,495	0,238	1
MAGT1	0,003346877	0,486573842	0,441	0,167	1
NPC2	0,003394282	0,575427983	0,658	0,452	1
AHI1	0,003516655	0,450808224	0,315	0,071	1
KDELR1	0,003588401	0,379793582	0,505	0,214	1
MEDAG	0,003643343	0,695342543	0,279	0,071	1
ECM1	0,003810136	0,542574755	0,351	0,119	1
CMKLR1	0,003858769	0,368540554	0,297	0,071	1
CYB5R3	0,00387803	0,481863712	0,802	0,714	1
DAB2	0,004843937	0,375097449	0,324	0,095	1
QSOX1	0,005010371	0,477062643	0,279	0,071	1
GPX3	0,005046498	0,676576594	0,64	0,452	1
C6orf62	0,005190194	0,585201367	0,315	0,095	1
PIK3IP1	0,005239691	0,364859049	0,261	0,048	1
FLOT1	0,005478827	0,478935174	0,45	0,19	1
MATN2	0,005594145	0,420926793	0,351	0,119	1
YKT6	0,005721624	0,393716762	0,252	0,048	1
CALU	0,005776714	0,529404981	0,676	0,476	1
LRP1	0,005778643	0,555863985	0,712	0,548	1
TCF4	0,006325663	0,481228409	0,802	0,738	1
PAFAH1B2	0,006435806	0,298596087	0,414	0,143	1
ADAMTS2	0,006629567	0,445317061	0,315	0,095	1
MEG3	0,006825589	0,539100778	0,279	0,071	1
SLC41A3	0,007027259	0,394115242	0,324	0,095	1
PLTP	0,007624552	0,62809616	0,45	0,214	1
HLA-B	0,007674075	0,436801549	0,91	0,786	1
PPIC	0,007848571	0,352396661	0,324	0,095	1
TMEM204	0,008327524	0,580234924	0,36	0,143	1

IFNGR2	0,008531852	0,402009539	0,342	0,119	1
UACA	0,008837547	0,621895936	0,55	0,333	1
CREB3L2	0,009126522	0,588139662	0,495	0,31	1
PLEC	0,009206228	0,500274722	0,658	0,452	1
LOXL1	0,009280548	0,364917673	0,378	0,143	1
DUSP6	0,009408009	0,597787066	0,252	0,071	1
NAV1	0,00952322	0,599792131	0,414	0,19	1
LRP6	0,009576053	0,471745199	0,261	0,071	1
RPN2	0,009604726	0,520406397	0,559	0,31	1
GRN	0,009616783	0,589275893	0,514	0,262	1
TAGLN2	0,009680675	0,464473274	0,703	0,524	1
AGL	0,009737265	0,499937048	0,261	0,071	1
ABLIM1	0,009835375	0,563353278	0,288	0,095	1
NUCB2	0,00993704	0,47291441	0,36	0,143	1

Cluster SMC.1 marker genes

gene	p_val	avg_logFC	pct.1	pct.2	p_val_adj
MCAM	3,29E-14	1,712102866	0,786	0,189	6,23E-10
TPM2	4,36E-13	1,182403631	0,976	0,73	8,26E-09
ACTA2	1,29E-12	1,305195601	0,976	0,739	2,44E-08
MYH11	8,65E-11	1,42829643	0,952	0,559	1,64E-06
RBPMS	1,67E-10	1,183730559	0,81	0,369	3,17E-06
MYL9	2,05E-10	1,059225836	0,976	0,793	3,89E-06
RGS5	3,30E-10	1,623478221	0,881	0,505	6,25E-06
DSTN	4,44E-10	1,032375686	0,929	0,793	8,41E-06
MYL6	5,03E-10	0,729839943	0,952	0,901	9,53E-06
TAGLN	7,01E-10	0,86237679	1	0,82	1,33E-05
CNN1	5,04E-09	1,288560436	0,524	0,108	9,55E-05
PALLD	7,91E-09	0,833074185	0,952	0,586	0,000149806
TNS1	2,52E-08	1,197473258	0,881	0,64	0,000476852
PLN	2,80E-07	1,440728498	0,595	0,234	0,005298265
MYLK	9,83E-07	0,914667733	0,833	0,667	0,018628194
CALD1	1,25E-06	0,54026321	1	0,91	0,023708749
CSRP2	3,29E-06	1,011271624	0,381	0,081	0,062409707
FRZB	4,44E-06	0,773758122	0,786	0,477	0,084077581
DLX5	6,34E-06	0,997464088	0,286	0,036	0,120121558
SYNPO2	6,43E-06	0,854297747	0,69	0,351	0,1218984
CSRP1	9,47E-06	1,132352289	0,69	0,414	0,179483443
GUCY1A1	9,68E-06	0,654529149	0,952	0,685	0,183379349
ADAMTS1	1,25E-05	1,105309908	0,476	0,162	0,237189026
LPP	1,42E-05	0,734141913	0,881	0,676	0,269015993
PPP1R12B	1,52E-05	0,884786304	0,667	0,369	0,288538677
MAP1B	1,65E-05	0,494862324	0,881	0,811	0,313048371
RAMP1	1,87E-05	0,995864615	0,381	0,099	0,355175174
FLNA	3,03E-05	0,751318108	0,857	0,811	0,573879954
C11orf96	3,39E-05	1,177777126	0,714	0,495	0,642111198
PLK2	8,75E-05	1,120678405	0,31	0,072	1
PDE5A	9,81E-05	0,895724124	0,762	0,505	1
PDE8B	0,000143355	0,851977809	0,31	0,081	1
NFIA	0,000148068	0,797911747	0,881	0,712	1
A2M	0,000150596	0,553879361	0,929	0,703	1
ITGA7	0,000153456	0,881344295	0,452	0,189	1
ACTG2	0,000207935	0,842429084	0,262	0,054	1
ACTB	0,000217408	0,318943729	0,976	1	1
MPRIP	0,000223875	0,6633225	0,667	0,378	1
MAP3K20	0,000234899	0,824008168	0,571	0,315	1
HSPA1A	0,000238124	1,017903729	0,571	0,324	1
CPED1	0,000281894	0,754140767	0,476	0,234	1
ATP1A2	0,000331588	0,758803363	0,262	0,054	1
EFHD1	0,000396647	0,882054188	0,571	0,333	1
SORBS1	0,000416448	0,926694008	0,5	0,252	1
MYH9	0,000422764	0,577444946	0,976	0,838	1
ATP2A2	0,000442878	0,790541312	0,595	0,396	1
KCNAB1	0,000540536	0,672675392	0,262	0,063	1
MRVI1	0,000627849	0,71394727	0,548	0,324	1
MFAP4	0,00076936	0,619721536	0,786	0,514	1
FYCO1	0,000807901	0,675991095	0,381	0,153	1
DOCK10	0,000869631	0,618145685	0,31	0,099	1
NIPSNAP2	0,000906381	0,478868062	0,286	0,081	1
PPP1R14A	0,000917193	0,607853506	0,69	0,459	1

VCL	0,000969455	0,480297739	0,786	0,613	1
C12orf75	0,001062069	0,689853252	0,476	0,234	1
CARMN	0,001370399	0,68542008	0,405	0,162	1
ATRX	0,001508838	0,570300548	0,69	0,432	1
NOTCH3	0,001611575	0,467932222	0,905	0,676	1
SYNM	0,001701331	0,652514571	0,452	0,234	1
AKAP1	0,00176833	0,585851646	0,381	0,162	1
LMOD1	0,001802171	0,775830692	0,619	0,414	1
ARID5B	0,002059621	0,708180759	0,571	0,36	1
MSN	0,002124143	0,602694141	0,619	0,414	1
TGFB1I1	0,002231575	0,52350055	0,619	0,432	1
KANK2	0,002297573	0,622928153	0,667	0,486	1
FBXO32	0,002494961	0,553431731	0,476	0,252	1
ID4	0,00253916	0,602844278	0,69	0,477	1
SLMAP	0,002561082	0,924512531	0,333	0,135	1
BTF3	0,002564267	0,296350986	0,881	0,676	1
NDUFA4	0,002854274	0,500112481	0,714	0,613	1
CYTH2	0,002926703	0,406701455	0,286	0,099	1
CAVIN3	0,003234023	0,66053471	0,643	0,459	1
WDR1	0,003373147	0,624277841	0,595	0,477	1
KCTD10	0,003439974	0,49804945	0,476	0,27	1
KIAA0040	0,00369601	0,381879677	0,286	0,099	1
SSRP1	0,004452409	0,659487248	0,262	0,099	1
CCDC3	0,004508117	0,46070043	0,524	0,297	1
NTRK3	0,0046867	0,617581565	0,405	0,207	1
SELENOW	0,004703965	0,544486129	0,69	0,559	1
ACTN1	0,004892602	0,538882731	0,81	0,667	1
PFKM	0,005315277	0,405345835	0,31	0,126	1
SSB	0,005723254	0,441049803	0,524	0,315	1
HCFC1R1	0,005860333	0,512209327	0,571	0,396	1
BEX4	0,005990279	0,656548349	0,286	0,108	1
GEM	0,006427012	0,834986015	0,571	0,387	1
COX7A2	0,006477317	0,568561985	0,643	0,459	1
DUSP3	0,006523463	0,533717186	0,381	0,207	1
SMIM10	0,00672445	0,537527136	0,262	0,099	1
EPAS1	0,006800278	0,286415875	0,881	0,694	1
DIS3L	0,006911664	0,586275436	0,31	0,135	1
SUSD5	0,006941922	0,68205015	0,357	0,171	1
NPM1	0,007131883	0,393735744	0,81	0,586	1
SMTN	0,007167829	0,561222363	0,31	0,135	1
HSPB8	0,007595509	0,755851804	0,357	0,189	1
PIN1	0,007607387	0,562909515	0,667	0,55	1
FILIP1L	0,007693567	0,923130895	0,381	0,198	1
CRIP1	0,007754277	0,483741245	0,81	0,694	1
DEPP1	0,007988235	0,596409716	0,31	0,135	1
TCEAL1	0,007988235	0,499483822	0,31	0,135	1
ABHD4	0,008212897	0,459282445	0,262	0,099	1
CAT	0,008575353	0,549122925	0,405	0,225	1
CALM2	0,008982779	0,418239627	0,857	0,82	1
PPP1CB	0,009657514	0,360837588	0,833	0,73	1
SLC6A2	0,009969728	0,587872136	0,429	0,243	1

Cluster Mixed.0 marker genes

gene	p_val	avg_logFC	pct.1	pct.2	p_val_adj
EEF1A1	2,55E-05	0,655209175	0,805	0,764	0,482541748
JUND	0,000111817	0,623108947	0,54	0,393	1
B2M	0,000173227	0,553310954	0,735	0,625	1
SERF2	0,000200533	0,741863319	0,522	0,416	1
TMSB4X	0,000343697	0,804595976	0,628	0,61	1
HLA-C	0,001564439	0,508479683	0,593	0,502	1
VIM	0,002014796	1,053222456	0,566	0,528	1
ACTG1	0,002014817	0,733948404	0,496	0,446	1
ACTN4	0,002143355	0,705981943	0,354	0,232	1
ZFP36L2	0,002650786	0,557978009	0,372	0,258	1
TMSB10	0,00305265	0,610170869	0,566	0,491	1
EEF1G	0,003064063	0,694731585	0,425	0,348	1
ANXA1	0,004330108	0,764472929	0,301	0,191	1
FAU	0,005024358	0,573730193	0,487	0,404	1
S100A6	0,005977757	0,586354671	0,398	0,315	1
RACK1	0,006954577	0,554960798	0,469	0,371	1
HLA-B	0,007636019	0,436162375	0,655	0,607	1

Cluster Mixed.1 marker genes

gene	p_val	avg_logFC	pct.1	pct.2	p_val_adj
LINC01554	3,93E-16	0,673519619	0,363	0,04	7,45E-12
ALAD	6,44E-15	0,517412808	0,431	0,072	1,22E-10
VNN1	8,03E-15	0,449933714	0,412	0,065	1,52E-10
LINC00629	1,16E-13	0,548577135	0,51	0,119	2,20E-09
ZNF80	1,20E-13	0,474641312	0,314	0,036	2,28E-09
FAM169B	2,70E-12	0,308947363	0,324	0,047	5,11E-08
FUCA2	2,90E-12	0,458503947	0,343	0,054	5,49E-08
SLC19A3	1,21E-11	0,38424328	0,324	0,05	2,29E-07
ABHD12B	2,62E-11	0,403440243	0,343	0,061	4,96E-07
MFSD11	2,81E-11	0,275769925	0,402	0,086	5,32E-07
SGCG	2,84E-11	0,383581681	0,49	0,122	5,38E-07
LOC284632	3,98E-11	0,34704263	0,304	0,047	7,54E-07
ASTN2	5,24E-11	0,706834913	0,961	0,831	9,94E-07
LOC257396	5,28E-11	0,576905327	0,451	0,119	1,00E-06
AP5Z1	6,22E-11	0,564528495	0,431	0,108	1,18E-06
SLC28A2	1,13E-10	0,364909649	0,255	0,032	2,14E-06
NOL10	1,35E-10	0,331482921	0,382	0,083	2,56E-06
LINC00842	1,71E-10	0,66496618	0,657	0,252	3,24E-06
ANK3	1,77E-10	0,427305422	0,333	0,065	3,36E-06
GBP5	2,72E-10	0,43272973	0,373	0,083	5,16E-06
CPN2	2,77E-10	0,431090765	0,48	0,129	5,24E-06
LINC00051	3,68E-10	0,334596101	0,304	0,054	6,97E-06
OGFOD1	3,76E-10	0,481265837	0,392	0,097	7,13E-06
IQCH-AS1	4,44E-10	0,34722908	0,402	0,097	8,40E-06
AK9	4,53E-10	0,315533161	0,284	0,047	8,58E-06
OR11A1	4,55E-10	0,328756369	0,294	0,05	8,61E-06
VN1R2	4,70E-10	0,456741513	0,373	0,086	8,91E-06
ZNF264	4,73E-10	0,290546505	0,618	0,201	8,96E-06
DEPDC1B	4,91E-10	0,27697138	0,382	0,086	9,31E-06
SULT1C2P1	5,30E-10	0,349410878	0,382	0,09	1,00E-05
ASCC1	5,45E-10	0,457028168	0,48	0,14	1,03E-05
FKBP7	5,73E-10	0,39041208	0,529	0,162	1,09E-05
LOC285762	5,81E-10	0,447514405	0,51	0,151	1,10E-05
CRTAM	7,48E-10	0,306187144	0,363	0,083	1,42E-05
TMEM135	8,99E-10	0,510631908	0,471	0,133	1,70E-05
PLEKHA6	9,62E-10	0,508146989	0,422	0,115	1,82E-05
ZNF93	1,08E-09	0,419842072	0,333	0,072	2,04E-05
SLCO1A2	1,27E-09	0,334525815	0,304	0,058	2,40E-05
ZNF37BP	1,28E-09	0,432487231	0,951	0,745	2,43E-05
CHP2	1,32E-09	0,378345893	0,422	0,112	2,50E-05
CHAF1B	1,45E-09	0,29708245	0,265	0,043	2,74E-05
KIAA0825	1,45E-09	0,329974633	0,255	0,04	2,76E-05
LOC157273	1,96E-09	0,345312926	0,51	0,162	3,71E-05
LINC00221	2,20E-09	0,328111385	0,5	0,151	4,17E-05
DIRC1	2,23E-09	0,341889857	0,324	0,068	4,22E-05
CES3	2,24E-09	0,561730747	0,373	0,094	4,24E-05
TRIM4	2,30E-09	0,50178269	0,667	0,281	4,36E-05
FOCAD	2,84E-09	0,308053246	0,402	0,104	5,37E-05
SETMAR	2,85E-09	0,364113331	0,549	0,176	5,40E-05
QSER1	2,92E-09	0,395042354	0,265	0,047	5,54E-05
LOC286359	3,22E-09	0,338056513	0,284	0,054	6,10E-05
SHANK2-AS1	3,48E-09	0,299190018	0,353	0,083	6,60E-05
FRA10AC1	4,21E-09	0,391335369	0,441	0,129	7,98E-05

RASAL2-AS1	5,02E-09	0,397026919	0,49	0,151	9,52E-05
LOC100507373	7,92E-09	0,429640315	0,49	0,155	0,000150143
CRIPAK	9,70E-09	0,250294968	0,294	0,061	0,000183743
CHRM3	9,86E-09	0,424144932	0,265	0,05	0,000186907
JRK	1,08E-08	0,33235613	0,51	0,169	0,000203881
LINC00648	1,23E-08	0,33012194	0,284	0,058	0,000232755
UMPS	1,36E-08	0,260878907	0,255	0,047	0,000258037
ZNF471	1,39E-08	0,372379629	1	0,903	0,00026317
GNE	1,46E-08	0,288088872	0,471	0,144	0,000277036
OTX2-AS1	1,68E-08	0,362853632	0,382	0,104	0,000319076
CCDC168	1,97E-08	0,432459585	0,373	0,104	0,000373244
OR7D2	2,05E-08	0,292662852	0,343	0,086	0,000388338
FAM173B	2,13E-08	0,287362374	0,304	0,068	0,000403416
NPAP1	2,47E-08	0,359196608	0,549	0,194	0,000467719
ALKBH4	2,58E-08	0,316453925	0,284	0,061	0,000489036
SLC9A3R2	2,95E-08	0,579791087	0,598	0,248	0,000559573
MCTP2	3,09E-08	0,472888977	0,676	0,288	0,00058576
TP53RK	3,14E-08	0,340470799	0,304	0,072	0,00059596
SLC7A11-AS1	3,37E-08	0,302376696	0,363	0,097	0,000638201
RGR	3,56E-08	0,390041615	0,373	0,104	0,000674178
PPP2R2B	3,56E-08	0,253492236	0,255	0,05	0,000674363
DGKB	3,70E-08	0,337334045	0,343	0,09	0,00070104
PBLD	3,78E-08	0,388124488	0,294	0,068	0,000715694
PGM3	4,18E-08	0,331655201	0,471	0,155	0,00079303
LOC100507377	4,31E-08	0,271605713	0,255	0,05	0,000815956
IFIT1	4,42E-08	0,385700821	0,412	0,126	0,000837534
USP33	4,52E-08	0,515044959	0,971	0,871	0,000857226
LOC339166	4,63E-08	0,316431055	0,294	0,068	0,000878255
TRMT5	4,74E-08	0,268657775	0,441	0,137	0,000898847
CCDC144B	5,60E-08	0,398922426	1	0,914	0,001062056
HERC2P4	6,58E-08	0,300535331	0,284	0,065	0,001246069
CA5B	6,68E-08	0,375528551	0,618	0,252	0,001265929
ZNF585B	6,93E-08	0,400430356	0,363	0,104	0,001313641
MYO5B	6,99E-08	0,277653809	0,284	0,065	0,001325243
CDYL	8,10E-08	0,352484301	0,402	0,122	0,001535136
UVSSA	8,16E-08	0,364484538	0,353	0,101	0,001546601
PARP11	8,22E-08	0,377314043	0,324	0,086	0,001558582
LINC00670	8,25E-08	0,466883214	0,892	0,604	0,00156338
SCD5	8,51E-08	0,625448463	0,765	0,496	0,001612051
NABP1	9,63E-08	0,314870924	0,314	0,079	0,001825047
CDK12	9,72E-08	0,3720383	0,255	0,054	0,00184198
PCLO	9,86E-08	0,278658799	0,304	0,076	0,001867687
PAPOLG	1,07E-07	0,250526858	0,265	0,058	0,002026486
LOC401324	1,14E-07	0,438301903	0,5	0,18	0,002154131
RNF217-AS1	1,18E-07	0,375171019	0,51	0,194	0,002235436
NUDT16P1	1,20E-07	0,258425091	0,363	0,101	0,00226574
RTKN2	1,24E-07	0,364936039	0,304	0,079	0,002356367
ZBTB38	1,26E-07	0,339924875	0,373	0,112	0,002385951
UCA1	1,32E-07	0,375891147	0,422	0,14	0,002502768
ERVFRD-1	1,51E-07	0,3054317	0,284	0,068	0,002854089
LONRF2	1,61E-07	0,341508071	0,304	0,079	0,00304715
GP6	1,66E-07	0,262431163	0,431	0,137	0,003139993
CTSC	1,70E-07	0,372950052	0,951	0,73	0,003216748
OLMALINC	1,75E-07	0,28368579	0,353	0,101	0,003309484
ZNF544	1,76E-07	0,304396454	0,275	0,065	0,003342203
ALG10B	1,90E-07	0,259676372	0,363	0,108	0,003608643

NSUN4	1,99E-07	0,267743958	0,353	0,101	0,00377182
SERPINA10	2,15E-07	0,279649044	0,343	0,097	0,004069933
NF1	2,20E-07	0,26637334	0,353	0,104	0,004166426
XIRP2	2,53E-07	0,273088366	0,265	0,061	0,004788762
TTN	2,93E-07	0,645840429	0,539	0,216	0,005547344
TRPM7	2,98E-07	0,406476094	0,657	0,295	0,005654249
EPPIN	3,08E-07	0,449809449	0,637	0,309	0,005833737
TLR4	3,19E-07	0,259106421	0,363	0,112	0,006048398
C20orf203	3,35E-07	0,36772176	0,422	0,147	0,006346324
SLC50A1	3,35E-07	0,271090134	0,402	0,129	0,006352958
ADIPOQ-AS1	3,43E-07	0,420925308	0,52	0,201	0,006508106
PATE4	3,58E-07	0,310513912	0,422	0,14	0,006787926
CPPED1	4,05E-07	0,345311632	0,559	0,223	0,007677777
TECRL	4,24E-07	0,383700233	0,598	0,252	0,008027828
DPY19L2P2	4,47E-07	0,361656922	0,471	0,173	0,008478144
LOC284581	4,86E-07	0,413527728	0,725	0,363	0,009213375
LOC100506023	4,88E-07	0,535881822	0,784	0,518	0,00924831
DBF4	4,93E-07	0,299675948	0,324	0,094	0,009333416
TLR8-AS1	5,06E-07	0,437445187	0,618	0,309	0,009593347
COQ7	6,17E-07	0,259653608	0,265	0,065	0,011694298
MMAA	6,50E-07	0,257689062	0,441	0,151	0,012314913
INIP	6,58E-07	0,26593913	0,284	0,076	0,012476325
GLIPR1L2	7,03E-07	0,356576245	0,647	0,299	0,013324414
CDKN2B-AS1	7,55E-07	0,276852673	0,441	0,155	0,014312282
LOC100131257	8,28E-07	0,300735452	1	1	0,015691818
COX10-AS1	8,30E-07	0,371260297	0,588	0,263	0,015734909
HCG18	1,05E-06	0,341102401	0,951	0,784	0,019809695
LINC00189	1,07E-06	0,319771368	0,373	0,122	0,020188132
LINC00652	1,13E-06	0,445691631	0,647	0,313	0,021451632
TESPA1	1,29E-06	0,297607089	0,5	0,194	0,024350921
DNAL1	1,31E-06	0,362941342	0,686	0,309	0,024848008
GLG1	1,38E-06	0,407097379	0,333	0,104	0,026232055
RASSF8	1,52E-06	0,25869706	0,598	0,252	0,028869533
TMEM212	1,57E-06	0,326889052	0,99	0,953	0,029804436
LINC00674	1,59E-06	0,322930209	0,539	0,227	0,030108573
LINC00410	1,67E-06	0,257860488	0,5	0,194	0,031620828
CNTRL	1,84E-06	0,490948227	0,265	0,076	0,03495854
ERVK13-1	2,01E-06	0,253881657	0,324	0,101	0,038169962
DESI1	2,05E-06	0,259965156	0,373	0,126	0,038896388
ZDHHC21	2,21E-06	0,326426141	0,333	0,108	0,041923339
TUNAR	2,25E-06	0,300098652	0,265	0,072	0,042599368
DNM1P46	2,27E-06	0,296625169	0,431	0,158	0,043030366
SHISA9	2,60E-06	0,394550624	0,794	0,475	0,049197178
F5	2,82E-06	0,388679396	0,971	0,91	0,053447252
ZNF780A	3,04E-06	0,283706974	0,451	0,165	0,057550511
LOC100190986	3,05E-06	0,301543865	0,794	0,414	0,057702909
APOL4	3,05E-06	0,370103718	0,725	0,392	0,0578187
PTCSC3	3,41E-06	0,255089253	0,775	0,392	0,064557462
SYNRG	3,43E-06	0,254351735	0,48	0,187	0,06504356
BAAT	4,09E-06	0,381036507	0,647	0,313	0,077421842
A1CF	4,78E-06	0,33648969	0,304	0,097	0,090500498
UTP14C	4,95E-06	0,28622994	0,471	0,187	0,093728014
XAF1	4,95E-06	0,297294979	0,99	0,939	0,093820138
APOL6	5,09E-06	0,395711493	0,843	0,658	0,096429007
KLRD1	5,27E-06	0,330203104	0,676	0,317	0,099901338
STAT5A	5,80E-06	0,382815745	0,647	0,317	0,109905852

LOC339803	5,89E-06	0,276167794	0,48	0,198	0,111654757
LINC00641	6,09E-06	0,268063789	0,265	0,076	0,115425478
PNPLA4	6,52E-06	0,331933347	0,422	0,165	0,123463681
FOXL2NB	6,61E-06	0,270954393	0,569	0,245	0,125263111
PPHLN1	6,64E-06	0,256704462	0,275	0,083	0,125785213
ADA2	6,86E-06	0,359779693	0,314	0,104	0,129975057
PLEKHA5	9,09E-06	0,380122046	0,784	0,511	0,172327428
GBP4	1,01E-05	0,255217862	0,716	0,367	0,191168718
ESRG	1,10E-05	0,437254642	0,99	0,817	0,208714809
POTEM	1,11E-05	0,350444884	0,902	0,716	0,209702678
NCBP1	1,12E-05	0,257731237	0,451	0,183	0,213081289
MCFD2	1,23E-05	0,371702472	0,922	0,669	0,233153613
NXNL2	1,32E-05	0,35088914	0,853	0,669	0,250117988
ORC4	1,58E-05	0,362866513	0,853	0,594	0,299898323
LINC00649	1,81E-05	0,262665286	0,549	0,245	0,342737178
ARHGEF26-AS1	1,97E-05	0,354014848	0,843	0,622	0,372426455
ACADSB	2,05E-05	0,380742208	0,931	0,781	0,388261504
TSG1	2,23E-05	0,26221738	0,627	0,309	0,422792
RAD21-AS1	2,27E-05	0,348559727	0,892	0,68	0,430182174
ZNF445	2,29E-05	0,282582413	0,686	0,335	0,434114632
CFLAR	2,70E-05	0,250744918	1	0,953	0,511867859
FAM111B	2,97E-05	0,358243248	0,775	0,46	0,56302377
XRCC2	3,23E-05	0,314955731	0,902	0,665	0,612110631
DDHD1	3,73E-05	0,254859345	0,98	0,892	0,706767984
SYNE2	4,37E-05	0,295340657	0,431	0,183	0,828412404
NPIPA1	4,53E-05	0,282445174	0,265	0,09	0,858540727
C2orf91	4,56E-05	0,332779607	0,706	0,403	0,86339615
EVL	4,59E-05	0,400088977	0,294	0,108	0,869159075
CC2D2A	4,63E-05	0,254200736	0,569	0,266	0,876548881
REXO1L1P	4,78E-05	0,37198443	0,627	0,324	0,905278662
DEGS1	4,82E-05	0,259821029	0,294	0,108	0,913623613
TSIX	5,07E-05	0,362567731	0,99	0,914	0,96086168
LOC286437	5,57E-05	0,317661115	0,873	0,701	1
MRPL36	5,88E-05	0,36594735	0,706	0,453	1
LOC401557	6,01E-05	0,30081937	0,255	0,086	1
CD47	7,04E-05	0,291417507	0,392	0,165	1
ENTPD1	7,20E-05	0,328097888	0,931	0,723	1
NLRP6	7,58E-05	0,414336823	0,529	0,273	1
NKAIN3-IT1	0,000112033	0,287375606	0,853	0,604	1
MC2R	0,000115632	0,355818122	0,569	0,306	1
TPTE2P1	0,000157686	0,254899574	0,922	0,719	1
LOC220729	0,000160979	0,279531228	0,637	0,363	1
LOC646214	0,000196387	0,329285021	0,745	0,511	1
TIGD1	0,000262171	0,314970305	0,853	0,741	1
ZNF441	0,000331563	0,253073561	0,657	0,374	1
SLC37A2	0,000342935	0,280093155	0,353	0,155	1
IBA57	0,000366304	0,269351876	0,471	0,223	1
LINC00547	0,000945776	0,261400334	0,804	0,554	1
LOC100130451	0,00099983	0,323985953	0,725	0,514	1
SRRM4	0,001350161	0,535591107	0,275	0,119	1
CD2	0,002020806	0,292068271	0,275	0,129	1
ALPK1	0,005431295	0,254112017	0,725	0,55	1

Cluster Mixed.2 marker genes

gene	p_val	avg_logFC	pct.1	pct.2	p_val_adj
TMEM212	5,41E-08	0,430736056	0,988	0,956	0,001024273
LOC100131257	1,97E-06	0,321112485	1	1	0,037336408
CFLAR	3,78E-06	0,32171526	1	0,956	0,071614246
MAB21L3	7,72E-06	0,367081127	0,988	0,98	0,146218523
PGM5P2	2,11E-05	0,344908102	1	0,973	0,399248155
CCDC144B	4,57E-05	0,315901417	0,976	0,925	0,865734707
SP100	4,87E-05	0,313868688	0,988	0,932	0,922270916
F5	5,74E-05	0,349328157	0,976	0,912	1
ASTN2	6,92E-05	0,36291418	0,965	0,837	1
XAF1	7,59E-05	0,290348895	0,988	0,942	1
ZNF37BP	0,000185	0,414189884	0,894	0,773	1
KLF15	0,000405578	0,42961899	0,812	0,692	1
POTEM	0,000784793	0,318778365	0,871	0,736	1
LOC286437	0,000793033	0,318616493	0,847	0,719	1
KLHL5	0,00079987	0,29208217	0,835	0,739	1
USP33	0,001684238	0,334585188	0,941	0,885	1
MCFD2	0,002158256	0,276515051	0,835	0,708	1
HCG18	0,002960395	0,268384989	0,918	0,803	1
NXNL2	0,004017971	0,297658958	0,847	0,681	1
CRYBB2P1	0,004251232	0,535661756	0,482	0,346	1
ARHGEF26-AS1	0,004524014	0,38017513	0,765	0,658	1
TPTE2P1	0,004572241	0,268240194	0,871	0,746	1
SAA1	0,00598994	0,294980713	0,812	0,732	1
RAD21-AS1	0,007272511	0,268418053	0,788	0,722	1

Cluster Mixed.3 marker genes

gene	p_val	avg_logFC	pct.1	pct.2	p_val_adj
LINC00311	0,000376298	0,66510716	0,5	0,327	1
BTN3A2	0,00624849	0,667336919	0,288	0,17	1
YME1L1	0,007615596	0,503821097	0,412	0,283	1

Table S3

Legend

Page	Relates to	Description
All_DEG	Figure 7A, Figure S7A	List of all differentially expressed genes divided across gene expression patterns
FUMA_MAGMA	M&M	Results from MAGMA mapped to FUMA derived information per gene.
Report	M&M	Overview of number of GWAS genes after each filtering step
Enrichment	Figure 7B	Enrichtment P values for permutations over random data per gene expression pattern
GWAS_DEG	Figure 7B	List of 74 mapped GWAS genes with FUMA derived information per gene; and linked to known GWAS locus information.
Enriched_GWAS_DEG	Figure 7C, 7D, 7E	More detailed information on the three groups of enriched genes similar to Online Table IIIA, but with LD information per independent SNP.
Erdmann	Figure 7A, 7B	Table 1 from Erdmann et al. 2018 mapped to the 15 gene expression patterns

Online Table III

All_DEG

DEG	genepattern	DEG	genepattern	DEG	genepattern
MEF2C	1	CD3E	7	LEPROT	10
SELL	1	IL2RB	7	CDC42EP1	10
BIRC3	1	IL32	7	DYNLL1	10
SWAP70	1	ITM2C	7	NORAD	10
ADAM28	1	TARP	7	MCAM	10
DOK3	1	SH2D1A	7	NPTN	10
MTSS1	1	EOMES	7	THSD4	10
CCDC50	1	FYN	7	DEPP1	10
PIK3C2B	1	RAC2	7	DAP	10
RIPOR1	1	PTPRCAP	7	SLC29A1	10
CD79A	1	RUNX3	7	FILIP1L	10
BANK1	1	BCL11B	7	MORF4L2	10
CD19	1	CD96	7	PTMS	10
FAM129C	1	SAMD3	7	MYO1C	10
VPREB3	1	PVRIG	7	ADAMTS1	10
BCL11A	1	TC2N	7	SLC38A2	10
BLK	1	SLC38A1	7	CSRP1	10
FCRLA	1	AUTS2	7	PLK2	10
CD22	1	ARAP2	7	CRTAC1	10
COCH	1	THEMIS	7	SERPINE1	10
FCER2	1	LCK	7	NR4A1	10
CD79B	1	PIP4K2A	7	NID1	10
RALGPS2	1	HLA-F	7	SNCG	10
IGLL5	1	PRKCH	7	ARHGAP29	10
TCL1A	1	STK17A	7	CLEC3B	10
P2RX5	1	PARP8	7	LAMA5	10
IRF8	1	ITGAL	7	TJP1	10
JCHAIN	1	ACAP1	7	S100A16	10
FCRL5	1	NLRC5	7	BCAR1	10
FAM30A	1	NFATC2	7	TUBB6	10
HLA-DOB	1	FAM102A	7	FZD4	10
MS4A1	1	GNG2	7	sep-10	10
LINC00494	1	GNAQ	7	TMEM204	10
CXCR5	1	RAB27A	7	ERG	10
DERL3	1	CD69	7	TCF7L1	10
RUBCNL	1	TBCD	7	TANC1	10
TSPAN13	1	DUSP4	7	CDC42BPB	10
POU2AF1	1	TNFAIP3	7	MATN2	10
FCRL2	1	CLDND1	7	BACE2	10
ARHGAP24	1	MAL	7	APBB2	10
HVCN1	1	IL7R	7	LIMS2	10
NAPSB	1	LEF1	7	CTNNA1	10
BACH2	1	TCF7	7	RAPGEF5	10
CD24	1	SPOCK2	7	AKR1C3	10
CPNE5	1	DGKA	7	RCAN1	10
WDFY4	1	TNFRSF25	7	HDGFL3	10
AIM2	1	FLT3LG	7	SOX4	10
BLNK	1	CD40LG	7	ARHGEF12	10
MZB1	1	ITK	7	TSPAN3	10
GNG7	1	CYLD	7	SLC6A2	10
OSBPL10	1	GOLGA8A	7	TMTC1	10
AFF3	1	CDC14A	7	LIMA1	10
CXXC5	1	PAG1	7	PLXNA2	10
CD180	1	SYNE2	7	EMP2	10

CHD7	1
SNX2	1
CD40	1
TBC1D9	1
TP53INP1	1
ZNF395	1
SMIM14	1
LRMP	1
FCHSD2	1
TLR10	1
SEMA4B	1
COBLL1	1
DENND5B	1
TMEM154	1
PLPP5	1
VNN2	1
DNAJC10	1
MAP3K1	1
CYB561A3	1
IFT57	1
FAM3C	1
GSAP	1
GGA2	1
ITPR1	1
CHPT1	1
SYVN1	1
FLT3	1
GAPT	1
RFX5	1
SCPEP1	1
CD55	1
PPP1R9B	1
RNGTT	1
WASHC4	1
VOPP1	1
MED13L	1
DRAM2	1
ENGASE	1
TP53I11	1
SLC17A9	1
CHCHD10	1
RNASEH2B	1
SESN3	1
SLC15A4	1
SLC9A7	1
XBP1	1
KLF7	1
BICD2	1
RCC2	1
SND1	1
ARHGAP27	1
STX7	1
SCRN1	1
PLCG2	1
TRIO	1
SERTAD2	1
RHBDF2	1
CCNG2	1
ANKRD10	1
IMPDH2	1
SMARCB1	1

GATA3	7
PDE3B	7
DPP4	7
ZC3HAV1	7
TRAT1	7
PIK3IP1	7
BCL2	7
KLRD1	7
GNLY	7
GZMB	7
B2M	7
HLA-B	7
HLA-A	7
HLA-C	7
TMA7	7
sep-09	7
PSME1	7
JAK1	7
ARHGEF1	7
FNBP1	7
CDC42SE2	7
EVL	7
OST4	7
MAPK1	7
CCND3	7
EMB	7
CCND2	7
IKZF1	7
STAT1	7
RNF44	7
CNOT6L	7
RARRES3	7
CORO1A	7
CD247	7
MIAT	7
ICAM3	7
CD7	7
HOPX	7
PRF1	7
FGFBP2	7
KLRF1	7
SPON2	7
SH2D1B	7
TXK	7
FCRL6	7
TBX21	7
S1PR5	7
MYBL1	7
KLRB1	7
CD160	7
NCAM1	7
BIN2	7
KLRC3	7
TSPAN32	7
IL18RAP	7
PYHIN1	7
ZAP70	7
APMAP	7
TTC38	7
CHST12	7
ABHD17A	7

ATN1	10
ALDH1A2	10
PDLIM5	10
C7	10
SMAD6	10
SLPI	10
RHOB	10
MEDAG	10
AHNAK2	10
PCDH10	10
CDC42BPA	10
TSPAN9	10
SRPX	10
PTPRF	10
IL1R1	10
PTPRG	10
PPIC	10
HSD17B12	10
WLS	10
CYP4X1	10
ARL4D	10
PTPN14	10
TNS2	10
MPDZ	10
VCAM1	10
NT5E	10
NPR2	10
MTMR11	10
THBS1	10
CP	10
MYOF	10
ZFYVE21	10
PINK1	10
NFE2L1	10
SORBS2	10
PGM5	10
CKAP4	10
PXDC1	10
MDK	10
ITGAV	10
BCAP29	10
LRRC8A	10
TMBIM1	10
FNDC3B	10
STK38L	10
HSP90B1	10
NAV1	10
ANO6	10
ITGA3	10
TEAD1	10
FGF2	10
PEAR1	10
CTNNAL1	10
SYNJ2	10
ARHGEF10	10
DAB2IP	10
CSNK1E	10
FNIP2	10
IGF1R	10
MEGF6	10
CAMSAP2	10

UBE2J1	1
COMMD3	1
MGAT5	1
ITSN2	1
ZBTB18	1
IRF7	1
IL4R	1
ANKRD13A	1
PARP14	1
DUSP22	1
TCF3	1
MCM5	1
PELI1	1
TPCN1	1
OCRL	1
DMXL1	1
KLF6	2
ANXA1	2
JUND	2
COX8A	2
MCL1	2
EIF4H	2
CALM3	2
ID2	2
LPCAT1	2
FOSB	2
SIGLEC17P	2
TMEM176B	2
ARHGAP18	2
RGS1	2
CPM	2
DUSP6	2
PTGS2	2
SLC2A6	2
SLC25A37	2
MITF	2
FAM129B	2
RAB34	2
TENT5A	2
PLAGL1	2
GATA2	2
LTC4S	2
CSF2RB	2
BTG2	2
ALDH1A1	2
FCER1A	2
PAK1	2
NDRG2	2
SNX3	2
HPGDS	2
SLC18A2	2
HDC	2
RAB44	2
P2RX1	2
SLC45A3	2
CADPS	2
LIF	2
RGS13	2
CTSG	2
PRG2	2
CDK15	2

SLC9A3R1	7
GOLGA8S	7
C1orf21	7
RAP1GAP2	7
USP28	7
SYNE1	7
ZNF683	7
GNPTAB	7
PTPN4	7
TPST2	7
PBXIP1	7
IL2RG	7
RIPOR2	7
CYFIP2	7
RASGRP1	7
RORA	7
ORC3	7
OCIAD2	7
BTN3A2	7
SYTL1	7
NBEAL2	7
sep-01	7
OGT	7
TBC1D10C	7
CLEC2D	7
ANKRD12	7
STAT4	7
GLCCI1	7
EML4	7
CD6	7
AAK1	7
AKNA	7
TMC8	7
TMC6	7
SEMA4D	7
CCDC69	7
ATM	7
DEFA6	7
STK4	7
RASAL3	7
GBP5	7
MGAT4A	7
BTN3A1	7
RNF125	7
NLRC3	7
KLRG1	7
TSPAN14	7
JAK3	7
KIAA1551	7
PRKX	7
IL27RA	7
STK10	7
CARD8	7
PTPRJ	7
ADGRE5	7
TNIK	7
PTP4A2	7
MATK	7
RGCC	7
PRKACB	7
MLLT6	7

ACVR1	10
TRIP6	10
MAP2	10
TRIP10	10
ENDOD1	10
CTDSPL	10
DAG1	10
CFL2	10
sep-08	10
SH3D19	10
ANXA4	10
ATP2A2	10
MAGI2-AS3	10
RARRES2	10
RHBDF1	10
MTURN	10
LRP5	10
LAPTM4B	10
CDC42EP5	10
FLRT2	10
PSMB5	10
FAM171A1	10
TJP2	10
NGRN	10
SLC7A2	10
RHOC	10
RRBP1	10
C9orf3	10
FEZ1	10
DDAH1	10
RGS3	10
ZBTB16	10
SV2A	10
GALNT2	10
GALNT15	10
ROCK2	10
SETD7	10
EVI5	10
MRAS	10
ARMCX2	10
DYNC1I2	10
KIF13A	10
FAXDC2	10
GOLIM4	10
LATS2	10
RAB1A	10
SLIRP	10
ARPC1A	10
CYSTM1	10
KLF10	10
GNA11	10
PLPP1	10
COPRS	10
ANKRD50	10
RAB3GAP1	10
GJA4	10
SNX18	10
GSTT1	10
CHSY1	10
NAB1	10
SPATS2L	10

KIT	2
SLC24A3	2
TPSAB1	2
HPGD	2
CPA3	2
MAOB	2
ENPP3	2
VWA5A	2
EVPL	2
ADCYAP1	2
HS6ST1	2
CALB2	2
CMA1	2
RHEX	2
COL13A1	2
ADAMTS3	2
TPSG1	2
PTGS1	2
RAB27B	2
PIK3R6	2
KCNH2	2
IL1RL1	2
BTK	2
IFITM10	2
SMYD3	2
CSRP3	2
BMP2K	2
MEIS2	2
EPB41L1	2
ATP6V0A2	2
RAB37	2
ACSL4	2
IL18R1	2
EGR3	2
LPCAT2	2
NRIP3	2
TMEM44	2
SCIN	2
GMPR	2
ZNRF1	2
CTNNBL1	2
EXTL3	2
FADS1	2
SLC44A1	2
CD82	2
KIAA1522	2
ELL2	2
NDST2	2
EFCAB14	2
GALC	2
CSF1	2
RIN3	2
KRT19	2
CNRIP1	2
STX3	2
ARHGEF6	2
STMN1	2
ADRB2	2
SQSTM1	2
TDRD3	2
SLC11A2	2

PPP2R5C	7
ITGB7	7
DENND2D	7
SKAP1	7
BIN1	7
NFATC3	7
SH2D2A	7
SYTL2	7
CARD11	7
GZMM	7
TIGIT	7
PTPN22	7
PRKCQ-AS1	7
LLGL2	7
CBLB	7
TOP2B	7
ESYT1	7
CDC25B	7
SBK1	7
FKBP11	7
TENT5C	7
ARHGEF3	7
SIDT1	7
SACM1L	7
LIME1	7
NCR3	7
GYPC	7
DIP2A	7
RBL2	7
LEPROTL1	7
GPR171	7
CD5	7
RAPGEF6	7
INPP4B	7
PGGHG	7
CD27	7
SUSD3	7
CD3G	7
ANO9	7
TTC39C	7
HECA	7
PRKY	7
SIT1	7
SLFN5	7
LY6E	7
CASP4	7
PSMB9	7
ANKRD36B	7
LPIN1	7
ZFYVE28	7
TSEN54	7
LY9	7
APOBEC3H	7
CXCR3	7
USP9Y	7
KIF21B	7
LAG3	7
WNK1	7
GRK6	7
TXLNGY	7
LDLRAP1	7

CST3	11
MARCKS	11
NPC2	11
TIMP2	11
KCTD12	11
ACTN1	11
MAFB	11
DAB2	11
APLP2	11
FOS	11
LRP1	11
VCAN	11
ZFHX3	11
CD93	11
CTSL	11
EGR1	11
PLTP	11
SLC40A1	11
RNASE1	11
GPNMB	11
CXCL2	11
APOE	11
METTL7A	11
CD63	11
CEBPD	11
LGMN	11
ZFP36	11
FABP5	11
CALM2	11
RTN4	11
JUNB	11
CALR	11
LAMP1	11
GSTP1	11
ATP6AP2	11
TUBA1B	11
CSTB	11
DUSP1	11
IFI6	11
PEA15	11
LGALS3	11
TUBA1A	11
AHR	11
CYFIP1	11
SOCS3	11
STAB1	11
GPR34	11
GGTA1P	11
C3	11
LILRB5	11
TMEM37	11
ME1	11
CREG1	11
SGK1	11
CCL8	11
OLFML2B	11
FUCA1	11
FCGBP	11
NELL1	11
PLD3	11
MERTK	11

GBE1	2
ST7	2
VPS37B	2
TFRC	2
PPP1R15A	2
MLLT1	2
USP53	2
IRS2	2
MYADM	2
RARA	2
LMAN2	2
MBOAT7	2
NR4A2	2
ARRB1	2
SERPINB1	2
TXNDC12	2
KRT1	2
ZMAT1	2
HAAO	2
PRKCA	2
ANKRD28	2
STXBP5	2
NDFIP2	2
SLC33A1	2
ARL2	2
SNX6	2
COL4A5	2
NCOR2	2
PER1	2
EIF3B	2
MPV17	2
PARVB	2
BHLHE40	2
F11R	2
DHRS9	2
ADAM12	2
APEX1	2
PADI2	2
NME8	2
SSBP3	2
ZNF618	2
VAC14	2
NT5DC2	2
CYB5D1	2
PLK3	2
AP3D1	2
HADHB	2
BCL3	2
NUMB	2
MSRA	2
ARHGEF40	2
NEK6	2
OAF	2
SLC27A3	2
TMEM256	2
ABCC1	2
NSMCE1	2
SELENOK	2
PDE4A	2
ZNF428	2
NT5C	2

MDFIC	7
ARHGAP15	7
SPATA13	7
PTGER2	7
ANXA6	7
KLF13	7
SIGIRR	7
DIAPH1	7
STK39	7
CCDC88C	7
TOX	7
BCL9L	7
STK26	7
PRKCQ	7
EIF4EBP2	7
PTPN7	7
PATL2	7
LYAR	7
KLRC1	7
TRAF3IP3	7
GSTK1	7
MAP4K1	7
PCED1B-AS1	7
IDS	7
VAMP2	7
ATP8A1	7
SLA2	7
KIF21A	7
RFLNB	7
SCARNA21	7
TUBA4A	7
GVINP1	7
RABGAP1L	7
ERBIN	7
STK38	7
CEP85L	7
ATXN7	7
CAPN15	7
DOCK11	7
PSTPIP1	7
SYTL3	7
SCARNA17	7
DENND1C	7
CCR5	7
FGD3	7
UNC13D	7
PRMT2	7
RASSF1	7
RAP1B	7
DHRS7	7
PPP6R1	7
PPP1CA	7
GSE1	7
TAP1	7
INPP4A	7
HELZ	7
WDR26	7
TCF25	7
SPSB3	7
SIPA1	7
CABIN1	7

PDK4	11
RGL1	11
LYVE1	11
DUSP3	11
SDC3	11
ADAM9	11
ABCA1	11
NINJ1	11
PLXNB2	11
CMKLR1	11
FRMD4A	11
KCNMA1	11
SPRED1	11
GAA	11
SLC7A8	11
RAC1	11
YWHAH	11
PLXND1	11
TLR4	11
SDCBP	11
HEXB	11
TSPAN4	11
APOC1	11
ATP6AP1	11
IDH1	11
MEF2A	11
CCL2	11
ITSN1	11
HERPUD1	11
BRI3	11
CREBL2	11
SOD2	11
GNS	11
OTUD1	11
ATP6V0A1	11
QKI	11
AP2A2	11
NCOA4	11
DPYSL2	11
MTRNR2L1	11
WARS	11
NRGN	11
RXRA	11
FKBP1A	11
CEBPB	11
ALDH2	11
ATP1B3	11
MMP9	11
MMP19	11
CYP27A1	11
LHFPL2	11
SCD	11
NR1H3	11
FAM20C	11
SLC43A3	11
TUBA1C	11
ANXA5	11
SDC2	11
CD276	11
LGALS1	11
ANXA2	11

EMD	2
FHOD1	2
MEPCE	2
CDS2	2
ELOVL1	2
NR1H2	2
CDK4	2
SLC38A10	2
CIZ1	2
CLCN3	2
EIF4G1	2
ARHGAP33	2
AAMP	2
C4orf48	2
FMN1	2
MTHFD1	2
FAM198B	3
LMO2	3
THBD	3
CALCRL	3
ID1	3
VWF	3
PECAM1	3
HEG1	3
SLC9A3R2	3
GFOD1	3
RAB3D	3
CDA	3
STOM	3
EMP1	3
CMIP	3
GNG11	3
APLNR	3
VWA1	3
SHANK3	3
PLVAP	3
ADGRL4	3
ESAM	3
DNASE1L3	3
SPNS2	3
ROBO4	3
CLEC14A	3
TM4SF18	3
ADAMTS9	3
PODXL	3
RASIP1	3
ADGRL2	3
ECSCR	3
PDE2A	3
CDH5	3
SOX18	3
GRB10	3
PCDH12	3
RAMP2	3
CYYR1	3
SLCO2A1	3
HYAL2	3
KANK3	3
TSPAN7	3
NEURL1B	3
EFNA1	3

DDX6	7
ARF6	7
BUB3	7
IGF2R	7
CNOT2	7
NCOA1	7
MAN1A2	7
ITPKB	7
SAMD9L	7
ADAR	7
KIAA2026	7
ANAPC5	7
LBR	7
PARP15	7
KDM5A	7
IPCEF1	7
ABRACL	7
CBFB	7
TIAL1	7
STAT5B	7
PCSK7	7
KLF12	7
UBL3	7
DNAJC1	7
RAB8B	7
SETD2	7
PITPNC1	7
TECR	7
PAXX	7
LPIN2	7
HIPK1	7
DNAJB1	7
MAP2K1	7
OAS2	7
FCGRT	8
GRN	8
RAB31	8
CTSB	8
PSAP	8
FTL	8
CYBB	8
GPX1	8
CD14	8
CD68	8
SPI1	8
AIF1	8
TNFAIP2	8
CTSH	8
LILRB2	8
MS4A6A	8
LYZ	8
CD163	8
TYMP	8
CSF1R	8
CXCL16	8
IER3	8
SERPINA1	8
C1QA	8
HLA-DRA	8
MPEG1	8
C1QC	8

SLC18A1	11
GSTO1	11
YWHAG	11
FABP4	11
ALCAM	11
MGLL	11
RNH1	11
PRDX1	11
TUBB2A	11
GPX4	11
NUPR1	11
ECM1	11
ZNF503	11
SORT1	11
EPB41L2	11
PMP22	11
CDKN1C	11
LAPTM4A	11
CDK2AP1	11
HSPA1A	11
CRTAP	11
DYSF	11
MGST2	11
TNFRSF1A	11
YWHAE	11
NECTIN2	11
RASA4	11
PDGFB	11
KLF4	11
PAPSS2	11
FRMD4B	11
PTTG1IP	11
BST2	11
ICAM1	11
PLSCR1	11
AXL	11
C1orf54	11
VASH1	11
CDKN1A	11
BEX4	11
SLC38A6	11
PAPSS1	11
ABCG1	11
FEZ2	11
CANX	11
IRAK3	11
MID1IP1	11
BNIP3L	11
ITPRIPL2	11
HSBP1	11
IER2	11
CTBP2	11
ZFAND5	11
MIDN	11
RAB10	11
OS9	11
PICALM	11
PHC2	11
PRKACA	11
AP2M1	11
RTN3	11

EGFL7	3
LDB2	3
SEMA6A	3
NOSTRIN	3
IFI27	3
ACKR1	3
TIE1	3
PLAT	3
CD34	3
LHX6	3
BCL6B	3
EMCN	3
PCDH17	3
OLFM1	3
TCIM	3
NUAK1	3
ZNF521	3
PALMD	3
EPHB4	3
TEK	3
ADGRF5	3
RAMP3	3
ARAP3	3
FLT4	3
TSPAN18	3
NPDC1	3
ADCY4	3
DOCK6	3
FXYD6	3
MALL	3
PDLIM1	3
RAPGEF3	3
PRCP	3
MMRN1	3
MAGI1	3
ITGA6	3
RAI14	3
THSD7A	3
SHC1	3
MMRN2	3
ETS2	3
RHOJ	3
MYCT1	3
CD200	3
MTUS1	3
CLDN5	3
CFI	3
RBP7	3
AGRN	3
NRN1	3
ITGB4	3
INSR	3
SELENON	3
ARHGAP23	3
GPR146	3
SULF2	3
MARCKSL1	3
NOTCH4	3
AFDN	3
FAM107A	3
RDX	3

FGL2	8
TGFBI	8
CPVL	8
NAMPT	8
GLUL	8
CTSS	8
SAT1	8
S100A9	8
FTH1	8
TYROBP	8
MSR1	8
C1QB	8
FCER1G	8
MS4A4A	8
C5AR1	8
S100A8	8
IL1B	8
CXCL8	8
IFI30	8
GNAO1	8
FCN1	8
SPP1	8
HLA-DRB1	8
HLA-DMA	8
CD74	8
HLA-DPA1	8
HLA-DPB1	8
HLA-DRB6	8
FGR	8
HLA-DMB	8
LYN	8
CTSD	8
FCGR3A	8
HLA-DQA1	8
CCL3	8
LST1	8
HLA-DRB5	8
ACTB	8
OAZ1	8
GNAI2	8
ARPC5	8
S100A11	8
GABARAP	8
ZFP36L1	8
GLIPR1L2	8
ATP6V0C	8
NFKBIA	8
RHOG	8
CEL	8
C1orf162	8
ITGAM	8
CD4	8
CAPG	8
RGS2	8
FOLR2	8
SLCO2B1	8
F13A1	8
VSIG4	8
SIGLEC1	8
FCGR2A	8
IGSF21	8

TPD52L2	11
RNF181	11
PSMD2	11
SEC11A	11
CUX1	11
PNKD	11
BANF1	11
SERPINB6	11
PLXDC2	11
MAN1A1	11
AKR1A1	11
FMNL2	11
BLVRA	11
KDELR1	11
MAFG	11
FAM20A	11
ABCC3	11
LAMP2	11
FCHO2	11
RNF13	11
COLEC12	11
DDAH2	11
CLTA	11
HSP90AA1	11
HEXA	11
VAMP3	11
TMEM14C	11
RCBTB2	11
BCAP31	11
PEPD	11
ARHGEF2	11
NAA20	11
HDLBP	11
SLC38A7	11
EPN1	11
COMT	11
ARL6IP1	11
OAZ2	11
CLTC	11
C20orf194	11
CORO1C	11
CAPZA2	11
ARHGEF10L	11
HSD3B7	11
PHLDA1	11
VKORC1	11
NME1	11
P4HA1	11
KHSRP	11
MYO1E	11
PLOD1	11
NUCB1	11
ACP2	11
TMED9	11
TMEM219	11
SEC61A1	11
RAB7A	11
AGAP3	11
ASPH	11
LIMS1	11
SFXN3	11

PKP4	3
KDR	3
SUN1	3
S1PR1	3
SELE	3
ECE1	3
FSCN1	3
JUP	3
ACVRL1	3
STC1	3
EFNB2	3
NDRG1	3
FLNB	3
MCTP1	3
TGFBR2	3
CAVIN2	3
ADAM15	3
MRTFB	3
MCF2L	3
PALM	3
ITGA5	3
JAM2	3
PDIA5	3
PTPRM	3
BCAT2	3
KCNN3	3
ST6GALNAC3	3
RAB11A	3
TNKS1BP1	3
DOCK9	3
FLT1	3
MPZL2	3
ADAMTS5	3
TRIOBP	3
EI24	3
H19	3
BMPR2	3
RALGAPA2	3
GIMAP6	3
CPD	3
CTNNB1	3
APOLD1	3
HDAC9	3
TTC28	3
PIK3C2A	3
CYP1B1	3
MAPK3	3
CLIC2	3
KLF2	3
GJA5	3
SELP	3
BMX	3
SEMA3F	3
PROCR	3
FGF18	3
CPAMD8	3
PTPRB	3
NDRG4	3
ANXA3	3
AIF1L	3
DKK2	3

C3AR1	8
ADAP2	8
SLC15A3	8
HMOX1	8
NCF4	8
C2	8
RNASE6	8
LILRB4	8
CLEC7A	8
CXCL3	8
SDS	8
TMEM176A	8
GATM	8
SLAMF8	8
AP1B1	8
TMEM51	8
PLAU	8
FPR3	8
LY86	8
RAB32	8
TBXAS1	8
IGSF6	8
SIRPA	8
RNF130	8
CCR1	8
PILRA	8
SLC1A3	8
TLR2	8
CD86	8
DSC2	8
CD209	8
NPL	8
TTYH3	8
IGF1	8
MARCH1	8
MNDA	8
ASAH1	8
BLVRB	8
CTSZ	8
MFSD1	8
LAIR1	8
RIN2	8
THEMIS2	8
HLA-DQB2	8
TREM2	8
RBM47	8
VMO1	8
ETV5	8
CFD	8
EPB41L3	8
TNFSF13B	8
HNMT	8
HCK	8
ITGAX	8
ALOX5	8
ARRB2	8
LRRC25	8
HLA-DOA	8
DSE	8
SHTN1	8
CD83	8

RAB5A	11
EHD4	11
CRYL1	11
SLC48A1	11
SEC14L1	11
ZMIZ1	11
TRIM8	11
NFE2L2	11
RALB	11
NCSTN	11
DENND5A	11
FLOT1	11
GABARAPL1	11
FZD1	11
SDC4	11
DOCK4	11
H1F0	11
PIM3	11
ATF3	11
PTPA	11
GUSB	11
ANAPC11	11
LTBR	11
MARVELD1	11
CITED2	11
MTRNR2L8	11
TIMM8B	11
TMSB4X	12
TMSB10	12
ACTG1	12
RPS27A	12
PTMA	12
YWHAB	12
MYL6	12
S100A4	12
TMBIM6	12
RHOA	12
HNRNPA2B1	12
ZFP36L2	12
COX4I1	12
HSPA8	12
S100A10	12
YBX1	12
ENO1	12
PCBP1	12
SNHG5	12
EIF4G2	12
EIF3L	12
UQCR11	12
ATP5F1EP2	12
GAPDH	12
TLN1	12
HNRNPK	12
PGK1	12
COX6B1	12
PCBP2	12
ARPC1B	12
COX6C	12
CHCHD2	12
LDHA	12
ATP5F1B	12

BMP6	3	FCGR2B	8	SERP1	12
RUNX1T1	3	EMILIN2	8	PSMA7	12
EDN1	3	DMXL2	8	PSMB1	12
NES	3	P2RY13	8	MSN	12
MMP28	3	RUNX1	8	PSMB4	12
ASS1	3	CXCL1	8	SH3BGRL	12
MECOM	3	UNC93B1	8	IQGAP1	12
MANSC1	3	SYK	8	HNRNPD	12
LIFR	3	LPAR6	8	RPN2	12
TSPAN6	3	PTAFR	8	SLC25A3	12
CCM2L	3	HLA-DQA2	8	NDUFA1	12
TCN2	3	PLAUR	8	PNRC1	12
TBX1	3	MGAT1	8	ATP6V0E1	12
PLXNA4	3	GM2A	8	ATP5ME	12
SLC6A1	3	TLR7	8	ELOB	12
NOG	3	LY96	8	NDUFS5	12
NOS3	3	CCL18	8	LRRC75A-AS1	12
BMP4	3	TNF	8	RNF145	12
JCAD	3	MAN2B1	8	GNG5	12
MAST4	3	COLGALT1	8	RAP1A	12
ITLN1	3	CCDC88A	8	TAX1BP1	12
POU4F1	3	PLEKHO2	8	ALDOA	12
CMAHP	3	ZEB2	8	SNRPB	12
FGD5	3	ATP6V0B	8	DDX3X	12
ACKR3	3	NAGK	8	FAM120A	12
ITPRID2	3	RB1	8	COX6A1	12
PDLIM4	3	PPT1	8	ATP5MPL	12
LYPD2	3	BCAT1	8	CSNK2B	12
CPNE2	3	CCDC170	8	CTDSP1	12
ALDH1A3	3	IFNGR1	8	PRICKLE4	12
IRF6	3	TNFSF15	8	ATP5IF1	12
SH3BP4	3	MPP1	8	GIMAP4	12
DUSP5	3	ABHD12	8	SERF2	12
SOX7	3	TPP1	8	ASAP1	12
BMP2	3	AKR1B1	8	FAM129A	12
CDH23	3	CALHM6	8	SLC25A5	12
TLNRD1	3	IL18	8	INSIG1	12
ITPR2	3	NAGA	8	TGFBR1	12
CSGALNACT1	3	ADA2	8	CAPNS1	12
DHRS3	3	HBEGF	8	HSPD1	12
HHEX	3	GRINA	8	RALA	12
TMEM120A	3	DUSP2	8	TXN	12
NRG1	3	PLIN2	8	RILPL2	12
WSB1	3	NEAT1	8	PPA1	12
RBMS2	3	LOC441081	8	CDC42	12
PTGDS	3	CREB5	8	BAG6	12
SOX13	3	NFKBIZ	8	TGOLN2	12
TNFSF10	3	SRRM4	8	CARD16	12
PON2	3	S100A12	8	STX10	12
DPY19L1	3	CFP	8	CHMP2A	12
PIK3R3	3	FPR1	8	SF3B5	12
HERC2P2	3	CSTA	8	WASF2	12
IQCK	3	SLC11A1	8	DDX21	12
TRIM47	3	NCF2	8	PSMB3	12
SHE	3	ASGR2	8	CAST	12
PRXL2A	3	PRAM1	8	JPT1	12
MTRF1L	3	CSF3R	8	NDUFB10	12
CTTNBP2NL	3	SECTM1	8	TRAPPC1	12
MYO6	3	C19orf38	8	IFITM2	12
TSPAN2	3	CLEC12A	8	ARF5	12
NQO1	3	APOBEC3A	8	PKN1	12

GIMAP7	3
AFAP1	3
PRXL2B	3
ROBO3	3
PKN3	3
PKHD1L1	3
IFI44L	3
NCOA7	3
ITGA2	3
VAMP5	3
DOCK1	3
SLC12A2	3
SOCS2	3
HAPLN3	3
CD109	3
STXBP1	3
KAT2A	3
SLCO3A1	3
DIPK1B	3
PHACTR2	3
PVR	3
KIAA0355	3
GPM6A	3
SH2D3C	3
NEDD9	3
ATP8B1	3
NOTCH1	3
MET	3
FAM43A	3
ESM1	3
FAM241A	3
SCARF1	3
GRASP	3
RGL2	3
DCHS1	3
ATP13A3	3
EVA1C	3
GIMAP1	3
GPRC5B	3
MRPL17	3
RELL1	3
APOL1	3
CYB561	3
MAP4K4	3
ARHGAP31	3
TMTC4	3
PTPRK	3
CCDC47	3
TAOK2	3
GIMAP8	3
ELK3	3
HDAC5	3
LTB	4
EEF1A1	4
TPT1	4
EEF1G	4
NPM3	4
EEF1B2	4
CCR7	4
NOP53	4
SARAF	4

HK3	8
BCL2A1	8
AQP9	8
LGALS2	8
G0S2	8
EREG	8
DDR2	8
ASGR1	8
SLC7A7	8
PLBD1	8
GCA	8
LILRB3	8
JAML	8
BRE-AS1	8
LILRP2	8
ZNF385A	8
CD36	8
VSIR	8
AP1S2	8
LRRK2	8
ODF3B	8
PGD	8
VMP1	8
VASP	8
CNPY3	8
PYCARD	8
OSCAR	8
PLXNC1	8
ANPEP	8
SLC16A3	8
IL1RN	8
CHST15	8
MXD1	8
ATP6V0D1	8
ACSL1	8
TALDO1	8
PTPRE	8
LILRB1	8
LGALS9	8
AGTRAP	8
ATP6V1B2	8
ARPC4	8
SIRPB1	8
SCIMP	8
FAM49A	8
CMTM6	8
LTA4H	8
BASP1	8
FBP1	8
AP2S1	8
GRB2	8
ZYX	8
PLIN3	8
PRKCD	8
CLEC4E	8
PPM1F	8
IL17RA	8
RPPH1	8
SH3BP2	8
DBNL	8
UBE2R2	8

COX5B	12
ATG3	12
ARL6IP4	12
COX7B	12
BRK1	12
UQCRC2	12
COPE	12
SLC25A11	12
HADHA	12
NEDD8	12
RAB1B	12
NADK	12
WDR1	12
GBP2	12
XRCC6	12
H1FX	12
PET100	12
ARF3	12
OTUB1	12
SF3B4	12
ANXA11	12
GPS2	12
GPI	12
CORO1B	12
SPOP	12
SPG21	12
ACADVL	12
PSMD1	12
PGAM1	12
TWF2	12
VPS29	12
HNRNPAB	12
UBA1	12
PPP2R1A	12
NAPA	12
EDF1	12
GHITM	12
PFKL	12
HPCAL1	12
LSM4	12
CCT5	12
TRIM28	12
ERGIC3	12
PSMA4	12
NDUFB2	12
HSPA9	12
SEC61G	12
NDUFA3	12
PPIA	12
SLC3A2	12
VDAC1	12
CD164	12
KLHDC3	12
SURF4	12
SLC25A39	12
ACTR1A	12
UPP1	12
HDGF	12
PITPNA	12
NPEPPS	12
HNRNPF	12

ADD3	4
SNHG8	4
NOSIP	4
FAU	4
EIF1	4
RACK1	4
UBA52	4
DDX5	4
EEF2	4
COX7C	4
NACA	4
BTG1	4
CCNI	4
EIF3E	4
NAP1L1	4
HNRNPH1	4
DDX17	4
HNRNPDL	4
EEF1D	4
SSR4	4
SSR2	4
FXYD5	4
ATP5MG	4
HINT1	4
SRRM2	4
EIF3K	4
YWHAZ	4
ZFAS1	4
HNRNPA1	4
COMMD6	4
SRSF2	4
EIF3H	4
SNHG6	4
CIRBP	4
TSC22D3	4
JTB	4
SUB1	4
EIF4B	4
SNRPD2	4
sep-06	4
PLP2	4
MCM2	4
TAPBP	4
FBL	4
YPEL5	4
MYCBP2	4
NONO	4
TRIM22	4
MBD2	4
EIF3G	4
GTF3A	4
PDCD4	4
BIRC6	4
LGALS13	4
FCMR	4
ATP2A3	4
PPP1R16B	4
NKTR	4
CYTH1	4
NUP210	4
ANKRD44	4

ATG16L2	8
PPIF	8
IL4I1	8
MARCO	8
ACP5	8
PKM	8
CLEC5A	8
TREM1	8
OLR1	8
EIF4EBP1	8
PDXK	8
SLC37A2	8
SLC31A2	8
H2AFY	8
CLDN1	8
ATP6V1F	8
CHI3L1	8
ATF5	8
IFNGR2	8
CADM1	8
KIAA0930	8
MRAP	8
CLEC10A	8
CD1C	8
CD1E	8
IL1R2	8
PLD4	8
PKIB	8
RTN1	8
S100B	8
TSPAN33	8
CD1D	8
HSPA7	8
SPINT2	8
SERPINF1	8
C15orf48	8
CLEC9A	8
PLEKHO1	8
CLEC4A	8
NR4A3	8
GDI2	8
IL13RA1	8
CAT	8
ETV6	8
B3GNT5	8
RNF144B	8
OGFRL1	8
SYNGR2	8
RBPJ	8
PABPC4	8
VEGFA	8
LAT2	8
PIK3AP1	8
RASSF2	8
SAMHD1	8
GRK3	8
ALDH3B1	8
IL6R	8
IRF5	8
SCARNA9	8
ADGRE2	8

B4GALT1	12
KLF3	12
ROMO1	12
CPNE3	12
MAPRE2	12
PNP	12
PXN	12
UFC1	12
TMEM258	12
PAIP2	12
CDC37	12
PTPRA	12
RALY	12
HNRNPM	12
PSMD13	12
CSNK1D	12
THOC7	12
SMARCC2	12
MEF2D	12
NDUFB1	12
GNB1	12
PHB2	12
SUMO3	12
ATP5MD	12
ATP5F1C	12
COX5A	12
ATP5PO	12
NDUFV2	12
ATP5F1D	12
MTDH	12
ATP5F1A	12
EIF6	12
SFPQ	12
CHMP4A	12
TBCB	12
SNRPG	12
CIAO2B	12
USB1	12
DYNC1LI1	12
MTCH2	12
PIN1	12
OXA1L	12
ATP5PD	12
NECAP2	12
CCT7	12
TMEM50A	12
PTBP1	12
WTAP	12
MAF1	12
YTHDF2	12
PFDN2	12
GALNT1	12
PSMA3	12
ENY2	12
PPP2CA	12
CCT4	12
NDUFA6	12
UBE2L3	12
KARS	12
TBCA	12
ITM2A	13

PDE7A	4	RNU11	8	LDHB	13
PPP3CA	4	AMPD2	8	IFITM1	13
STRBP	4	SLC43A2	8	ETS1	13
SEL1L3	4	PTCH2	8	TOMM7	13
TPD52	4	EHBP1L1	8	TBC1D4	13
ADAM19	4	HPSE	8	GAS5	13
PRDM2	4	ADAMTSL4	8	CALM1	13
PNISR	4	UQCRC1	8	HLA-E	13
PPM1K	4	C20orf27	8	MYL12A	13
RASGRP2	4	PYGL	8	UBB	13
ST6GAL1	4	BACH1	8	BTF3	13
FAM107B	4	KYNU	8	MBNL1	13
ISG20	4	CD300C	8	AES	13
ZBTB44	4	KDM6B	8	ARL6IP5	13
SEC61B	4	CD300LF	8	MYL12B	13
NPM1	4	ABI3	8	PPDPF	13
RHOH	4	METRNL	8	COX7A2	13
SF3B1	4	ARHGAP26	8	CRIP1	13
HIST1H4C	4	RNU4ATAC	8	SUN2	13
ERP29	4	GAS7	8	TRAM1	13
RBM3	4	NAIP	8	SELENOF	13
NDUFB11	4	TPI1	8	SPCS1	13
UBE2I	4	BLOC1S1	8	SHISA5	13
METTL9	4	GADD45B	8	ADGRG1	13
OGA	4	MYD88	8	CLIC3	13
TRAF5	4	GNB2	8	TGFBR3	13
RBM38	4	UBXN11	8	OPTN	13
KMT2E	4	TSPO	8	LBH	13
OFD1	4	LAMTOR1	8	ABLIM1	13
LYST	4	RAB5C	8	TNRC6C	13
ORMDL3	4	VPS35	8	MACF1	13
FAM214A	4	FOSL2	8	SOD1	13
SMCHD1	4	MAP3K3	8	FOXO1	13
PASK	4	PFKFB3	8	NR3C1	13
BCLAF1	4	PPP4C	8	ANAPC16	13
EIF3F	4	TRIM25	8	UTRN	13
ATP6V1G1	4	TMEM107	8	CEMIP2	13
LARP1	4	ZDHHC7	8	SNTB2	13
H2AFV	4	LOC644936	8	LSR	13
TNRC6B	4	RELT	8	SLC44A2	13
ELOVL5	4	BID	8	RBMS1	13
MYLIP	4	TFEC	8	NTAN1	13
MKNK2	4	HAVCR2	8	SNRK	13
AZIN1	4	TLR1	8	ZEB1	13
EPRS	4	PLA2G7	8	STMN3	13
CCNY	4	CEP170	8	LRIG1	13
MAT2B	4	GNB4	8	PURA	13
MFNG	4	SLC8A1	8	LINC00674	13
ACSL5	4	TLR5	8	PKD1P1	13
FLI1	4	TRIB1	8	FOXP1	13
SON	4	FGD4	8	NUP160	13
FUS	4	DAPK1	8	KTN1	13
UHMK1	4	RHOQ	8	PRDX2	13
PPP1R12A	4	RNF135	8	ADD1	13
PSMB8	4	SH2B3	8	NPIPA1	13
RASA3	4	SCAMP2	8	EIF4A2	13
C9orf16	4	RAP2B	8	TRIP11	13
UXT	4	ARL8B	8	TNRC6A	13
BRD2	4	OAS1	8	C12orf75	13
SRSF11	4	NRROS	8	PLEKHA1	13
SNRPB2	4	PLEKHB2	8	YWHAQ	13

ARHGAP25	4
HNRNPU	4
DGKD	4
CSNK1G2	4
TRIM33	4
CDK5RAP3	4
CTBP1	4
FAM53B	4
NR5A1	4
UBXN4	4
OSBPL8	4
SEC11C	4
USP34	4
AKAP9	4
IDH3B	4
SPG11	4
IL16	4
UQCRH	4
KHDRBS1	4
PSMA1	4
EIF3D	4
CHD4	4
UBE2D3	4
NFKB1	4
ANP32B	4
UBE2D2	4
SNRNP70	4
RAN	4
RBM39	4
CCT8	4
EIF3M	4
TNFAIP8	4
SMDT1	4
TRADD	4
NOP58	4
PIM2	4
SNHG1	4
ILF3	4
FNBP4	4
G3BP2	4
CUTA	4
CHD2	4
PNN	4
TAB2	4
RNF19A	4
EP300	4
PDE4B	4
SET	4
HNRNPA3	4
PHIP	4
RAD21	4
XRCC5	4
DDX3Y	4
MXD4	4
PILRB	4
EWSR1	4
HMGN4	4
HNRNPUL1	4
AP1G2	4
ORMDL1	4
STAG2	4

CACUL1	8
ARAP1	8
FES	8
GDE1	8
ST14	8
SIGLEC7	8
MFSD12	8
GLA	8
SMS	8
LYL1	8
ATP6V1A	8
MLEC	8
NUP62	8
SRD5A3	8
SEMA4A	8
PID1	8
PSTPIP2	8
SMCO4	8
NLRP3	8
FOXN2	8
PARL	8
BATF3	8
TRPS1	8
GCK	8
CHP1	8
SUPT4H1	8
RNPEP	8
RAB8A	8
CIAO2A	8
OTULINL	8
IMPA2	8
GLRX	8
MTHFD2	8
SLC12A7	8
RASGRP4	8
LOC1001312	9
PGM5P2	9
TMEM212	9
ODF2L	9
MAB21L3	9
CFLAR	9
CCDC144B	9
XAF1	9
ZNF471	9
ESRG	9
TSIX	9
DDHD1	9
ZNF480	9
F5	9
SP100	9
ASTN2	9
USP33	9
ACADSB	9
HCG18	9
TPTE2P1	9
NXNL2	9
SAA1	9
POTEM	9
LINC00550	9
ZNF37A	9
SBF2-AS1	9

ABCA2	13
sep-07	13
ERAP2	13
KMT2A	13
PPP2R5A	13
GLS	13
NCK2	13
ATP8B2	13
AQP3	13
PSIP1	13
SVIP	13
ITPR3	13
SNRNP200	13
CASK	13
CCSER2	13
NEK7	13
MPRIP	13
C12orf57	13
CBX7	13
USP11	13
ZBTB20	13
SMAD7	13
ANK3	13
UGP2	13
DMWD	13
ARGLU1	13
SMARCE1	13
CCNL2	13
C6orf48	13
CCNG1	13
PLCG1	13
HIP1R	13
ZMYND11	13
WWC3	13
ARL2BP	13
FAM117A	13
CAPN2	13
KAT2B	13
ESYT2	13
CDR2	13
ATP2B4	13
DTX3	13
LANCL1	13
C4orf3	13
AKT3	13
GUK1	13
PCNP	13
CDC42EP3	13
TMEM245	13
PCMTD1	13
APOL2	13
AK1	13
CD46	13
TROVE2	13
MIER1	13
TMEM230	13
PAFAH1B1	13
ALKBH5	13
PUM2	13
CGGBP1	13
MEX3C	13

POLR2B	4
PDS5A	4
TRA2B	4
ELF1	4
SRSF5	4
PPP1CC	4
TSPYL2	4
PRRC2C	4
SYNCRIP	4
MATR3	4
C8orf59	4
PHTF2	4
TAP2	4
NCOA3	4
JMJD1C	4
SP110	4
IQSEC1	4
LSM7	4
ARHGEF18	4
YPEL3	4
ODC1	4
UBA7	4
TMEM134	4
DCAF8	4
ANKLE2	4
MARS	4
EIF1B	4
TAF7	4
MAP3K2	4
TMEM243	4
MARCH6	4
SELENOH	4
HMGN1	4
PPP6R3	4
SAFB2	4
TMEM131	4
TNK2	4
PSMA2	4
IRF3	4
ATXN2L	4
PPP3CC	4
TMEM131L	4
LUC7L3	4
TUG1	4
BBX	4
NIN	4
NGLY1	4
STIM2	4
LOC643406	5
ANKRD20A9	5
ZNF829	5
ADH4	5
KLHL5	5
C2orf91	5
UGT2B15	5
SPDYE7P	5
LEPR	5
RNF213	5
LRRC27	5
ZNF445	5
TRMT9B	5

LINC00670	9
ZNF37BP	9
RAD21-AS1	9
XRCC2	9
NFX1	9
HYDIN	9
ENTPD1	9
TIGD1	9
NKAIN3-IT1	9
HEXA-AS1	9
LOC286437	9
LOC1005060	9
FAM111B	9
ALPK1	9
ARHGEF26-A	9
MCFD2	9
LINC00547	9
ORC4	9
ASB4	9
CTSC	9
SCD5	9
HTRA4	9
LINC-ROR	9
ARRDC3-AS1	9
LOC1001304	9
UTS2B	9
ACBD7	9
RAB3IP	9
LOC646214	9
PTCSC3	9
PLEKHA5	9
ADGRG4	9
ULBP1	9
LOC284581	9
SPART	9
LINC00842	9
REXO1L1P	9
MC2R	9
CD300E	9
SHISA9	9
ANKLE1	9
TLR8-AS1	9
ABCA9	9
ZNF441	9
CDC27	9
FAM92B	9
MRPL36	9
LOC1003792	9
APOL4	9
LOC220729	9
ANKRD36BP	9
LINC00346	9
ANKRD30BP	9
LINC00652	9
BAAT	9
RAB12	9
COX10-AS1	9
GPLD1	9
PCA3	9
LOC1005061	9
DYNAP	9

C19orf66	13
TES	13
ZRANB2	13
PIGK	13
NELL2	13
CMPK1	13
DUT	13
TOMM20	13
KRTCAP2	13
ATRX	13
SSU72	13
WAC	13
DHX15	13
DDX24	13
TNFSF12	13
HNRNPH3	13
SRI	13
SERBP1	13
ALDH9A1	13
HUWE1	13
SMARCA2	13
KRT10	13
RABAC1	13
PRRC2B	13
COPB1	13
CUL3	13
TTC3	13
CALD1	14
COL4A2	14
COL6A1	14
PRELP	14
TPM2	14
TAGLN	14
COL6A2	14
DSTN	14
MAP1B	14
THBS2	14
AEBP1	14
COL14A1	14
LTBP1	14
IGFBP5	14
COL1A2	14
MYL9	14
COL1A1	14
MYH11	14
CCDC80	14
ACTA2	14
RGS5	14
BCYRN1	14
ITGB5	14
SOD3	14
NOTCH3	14
PDGFRB	14
MYLK	14
MXRA8	14
ANTXR1	14
FRZB	14
GUCY1A1	14
MFAP4	14
PRRX1	14
MFGE8	14

FAM227A	5
LOC729732	5
LINC00649	5
CCDC148	5
LOC441666	5
SLC14A2	5
PRR11	5
LINC00663	5
POLH	5
ZNF793	5
SFTPB	5
AP4S1	5
ORAI2	5
CENPN	5
CYP20A1	5
ZNRF3-AS1	5
ZNF582-AS1	5
ZFP14	5
ALG1L9P	5
ZNF716	5
HUNK	5
CEACAMP1	5
PNPLA3	5
ATCAY	5
NPAP1	5
RASSF8	5
LOC1005069	5
PIN4P1	5
RAB3B	5
ZNF738	5
TLCD2	5
POM121L10	5
LINC00476	5
GJC1	5
SLFN12	5
TNFRSF9	5
IL10	5
METTL8	5
LIN28A	5
DIPK2B	5
POM121L8P	5
ZBTB8A	5
SIGLEC16	5
DNMBP-AS1	5
ARIH2OS	5
FOXRED2	5
RBSN	5
LOC1001348	5
DBT	5
LINC00578	5
LOC1005065	5
FXN	5
ZNF470	5
PPARA	5
C4orf19	5
LOC1001285	5
AIPL1	5
CHURC1	5
FZD3	5
ATP5MGL	5
LOC1001285	5

NUDT9	9
TECRL	9
RNF217-AS1	9
TSG1	9
EPPIN	9
APOL6	9
SLC6A20	9
LINC00410	9
ZNF667	9
FGF5	9
ZNF662	9
HLA-L	9
LPAL2	9
FOXL2NB	9
ABCC9	9
LINC00310	9
LOC157273	9
MGC27345	9
LOC285762	9
FECH	9
DZIP3	9
CTCFL	9
CDKN2B-AS1	9
CPN2	9
LOC283856	9
LSAMP	9
PLEKHA6	9
LOC1002894	9
CEP126	9
LOC440300	9
OXTR	9
HSD3BP4	9
ZNF551	9
RASAL2-AS1	9
KLF15	9
SGCG	9
HHLA2	9
LINC00507	9
SLC7A14	9
CLEC19A	9
ZKSCAN8	9
LOC440173	9
NCMAP	9
CARMN	9
PATE4	9
L2HGDH	9
DNM1P46	9
MCTP2	9
PASD1	9
LOC1005073	9
MYLK3	9
GNRHR2	9
DNAAF4	9
TRAPPC8	9
TEX35	9
COL6A4P2	9
SLC5A12	9
LOC401324	9
LINC00221	9
FAM120C	9
AP1S3	9

PDE5A	14
ID4	14
CRYAB	14
IGFBP2	14
MYH10	14
PDGFRA	14
ASPN	14
PCOLCE	14
FXYD1	14
ECM2	14
DKK3	14
NDUFA4L2	14
LMOD1	14
COL6A3	14
ADH1B	14
PDLIM3	14
SFRP4	14
RCAN2	14
SMOC2	14
PCOLCE2	14
ANO1	14
SSPN	14
GEM	14
ISLR	14
PPP1R14A	14
C2orf40	14
ITGBL1	14
SLIT1	14
TPM1	14
PALLD	14
LUM	14
EFHD1	14
IGFBP6	14
FHL1	14
C1QTNF1	14
HSPB6	14
DPYSL3	14
C1orf198	14
EPDR1	14
LHFPL6	14
MRVI1	14
C11orf96	14
BTF3P11	14
PRKG1	14
FBLN5	14
TBX2	14
COL12A1	14
HEYL	14
PFN2	14
ANGPT1	14
NEXN	14
CXCL12	14
COL21A1	14
EMILIN1	14
CNN3	14
TGFB1I1	14
FIBIN	14
TNS1	14
EBF1	14
DPT	14
LOXL1	14

SMA5	5
VSTM4	5
PABPC1P2	5
NLRP12	5
CABP4	5
MEG3	5
TMEM213	5
GUCY2C	5
ZNF114	5
FBXL18	5
LRRC2	5
SLC15A2	5
SLC35E2A	5
LINC00514	5
QPCTL	5
SCML4	5
FLJ42627	5
ZNF529	5
GRM6	5
ITIH5	5
TYW3	5
GK5	5
KREMEN1	5
EMX2OS	5
SGSM1	5
LINC00574	5
HAUS2	5
TMEM17	5
sep-14	5
NMNAT1	5
ZNF818P	5
PDE6A	5
PALM2	5
XPNPEP3	5
TMC7	5
NUBPL	5
ZNF665	5
FAM106A	5
ZNF483	5
THAP6	5
ALG1	5
JHY	5
THEM4	5
KBTBD6	5
MTRNR2L4	5
FBXL19-AS1	5
GOLGA8IP	5
SULT2A1	5
GPR155	5
TRAF3IP2	5
ZNF557	5
MARVELD3	5
TFDP2	5
COA1	5
FLVCR1	5
LINC00311	5
MTG2	5
OPA3	5
LMOD3	5
C21orf62	5
ABHD11	5

CHML	9
ADGRF2	9
ADIPOQ-AS1	9
ALOX12P2	9
CC2D2A	9
LECT2	9
HTATSF1P2	9
CRYBB2P1	9
SEC23IP	9
CCDC168	9
BRD7P3	9
DPY19L2P2	9
HAR1B	9
ZNF737	9
MMAA	9
LHFPL5	9
ZNF696	9
MLF1	9
LINC00629	9
KCNJ5	9
NXPE3	9
SHROOM4	9
STAT5A	9
ZNF425	9
DNAL1	9
UCA1	9
NOS1	9
C1orf140	9
PTGER3	9
FKBP7	9
DUXA	9
PTGER4P2-CI	9
IYD	9
C1RL-AS1	9
CMBL	9
TSC22D1-AS1	9
PEX5L	9
METTL21A	9
EPHA1-AS1	9
LOC1005075	9
ZNF621	9
KLHL7-DT	9
HTR3B	9
TRIM4	9
URB1	9
CASP10	9
COG6	9
C20orf203	9
BSN-DT	9
PATE2	9
ICA1L	9
LOC339803	9
LINC02249	9
CA5B	9
FDPSP2	9
PIPSL	9
LOC1002886	9
MYO10	9
ARGFX	9
FKBP14	9
ELMOD1	9

JAM3	14
LAMA2	14
ARHGEF17	14
KANK2	14
AGT	14
AOC3	14
VASN	14
FBN1	14
GAP43	14
TMEM30B	14
COL18A1	14
CNN1	14
PRDM6	14
EFEMP2	14
GPC6	14
MIR100HG	14
PPP1R3C	14
CRLF1	14
LRRC17	14
ANG	14
LRRC32	14
CHPF	14
RGS4	14
INHBA	14
SYDE1	14
MYO1D	14
FMO2	14
SFRP2	14
FHL2	14
TFPI2	14
GULP1	14
PDZRN3	14
FKBP10	14
LGALS3BP	14
PLN	14
COL5A2	14
COL5A1	14
AKAP12	14
COL4A1	14
DMD	14
ITGA11	14
sep-04	14
UNC5B	14
SORBS1	14
NPY1R	14
COL16A1	14
CAVIN3	14
ITGA7	14
SVIL	14
KCNMB1	14
YAP1	14
PXDN	14
PTN	14
PARM1	14
TWSG1	14
PHLDB1	14
RAMP1	14
OLFML1	14
MXRA7	14
MYO1B	14
PGF	14

PRDM7	5
RFWD3	5
MANEAL	5
DSG2	5
VPS53	5
PLA2G12A	5
KIF1C	5
IVD	5
METTL2B	5
ERCC4	5
LINC02210	5
RINL	5
DHTKD1	5
SPIB	5
COQ10B	5
PATJ	5
ARMC9	5
MR1	5
CACNG8	5
MIGA1	5
ZMYM5	5
LYRM7	5
HCG11	5
DPYSL5	5
BCL2L15	5
PBOV1	5
BAIAP2L1	5
C3orf62	5
PNPT1	5
NAIF1	5
PPARD	5
DFFA	5
LOC729683	5
IKZF3	5
NFAM1	5
MEGF9	5
MDM4	5
IRF1	5
SPN	5
LOC1001909	5
TSHZ2	5
SNX22	5
SLAMF6	5
CD28	5
ZNF197	5
PHAX	5
C17orf75	5
NUGGC	5
TTN	5
FGD5P1	5
CLSPN	5
GUSBP3	5
GAMT	5
ZNF814	5
ZNF320	5
GEMIN8	5
C17orf77	5
WDR17	5
TMEM181	5
SLC35A3	5
SH3TC2	5

PDP2	9
HHLA3	9
SCN11A	9
ZNF714	9
GTF2H5	9
RAMP2-AS1	9
CHIAP2	9
SEC14L4	9
ROCK1P1	9
BHMT2	9
GGT6	9
CYP27C1	9
ZFP42	9
JPX	9
ZNF208	9
VSIG1	9
KCNK6	9
SLC4A4	9
IRGQ	9
HNF1A-AS1	9
PNPLA4	9
JRK	9
KCNK3	9
SETMAR	9
TSTD3	9
EFHC1	9
ASB11	9
KIAA1549	9
SSTR2	9
CHST6	9
PXMP4	9
ZNF562	9
KRT18	9
PTPRZ1	9
TMEM192	9
TOR1AIP2	9
GOSR1	9
GDPD1	9
YAF2	9
IBA57	9
NLN	9
GFOD2	9
KCNA7	9
INF2	9
SPATA5	9
GBP4	9
ZNF333	9
ZHX3	9
KIAA1143	9
C9orf64	9
IPO9	9
ST3GAL6	9
WNT7B	9
ANP32A-IT1	9
SLC31A1	9
CPPED1	9
SIGLEC10	9
MLPH	9
AGAP1	9
SIGLEC8	9
LAX1	9

SYNPO2	14
SPRY1	14
LMO7	14
SGCB	14
GGT5	14
TCEAL4	14
MAP1A	14
FBLN1	14
SSC5D	14
MICAL2	14
PMEPA1	14
CDH2	14
FAT4	14
WISP2	14
PDGFA	14
PLSCR4	14
CPED1	14
LAMC1	14
CPQ	14
SYNM	14
COL15A1	14
HCFC1R1	14
SLC22A17	14
KRT17	14
ITGA8	14
ISYNA1	14
GADD45A	14
KANK1	14
NTRK3	14
PHLDB2	14
SUSD5	14
UACA	14
PPP1R12B	14
ZNF704	14
MID1	14
LAMA4	14
POSTN	14
FBXO32	14
PAPPA	14
ITGA1	14
MXRA5	14
KCTD10	14
MT2A	14
SEMA5A	14
CYTL1	14
ZMAT3	14
MAP3K20	14
LPP	14
F2R	14
TNC	14
SPRY4	14
PPFIBP1	14
RGS16	14
ANGPT2	14
C5orf24	14
ANKH	14
MGST3	14
ADH5	14
GLIS2	14
OAT	14
VEZF1	14

RAB11FIP4	5
TMEM56	5
DNAJC22	5
KLB	5
ZNF526	5
ACP7	5
AKAP5	5
ARNTL2	5
DNASE1	5
TCAF2	5
ORC6	5
TTTY15	5
KIF14	5
SLC16A7	5
LINC00294	5
LRRC58	5
LOC1001283	5
PTPRVP	5
NMT2	5
PGM2L1	5
UCKL1-AS1	5
CARF	5
ZNF626	5
SCAI	5
NICN1	5
WDR92	5
AGMAT	5
CLN8	5
NIPAL3	5
SLFN13	5
CDHR3	5
PDE4C	5
FBXL20	5
MAP3K9	5
PRKXP1	5
ZNF766	5
NKD1	5
GNAL	5
LRRC57	5
CFAP74	5
PARD6G	5
LOC1005064	5
BMP7	5
SMN1	5
BCAS1	5
NANOS1	5
POU6F1	5
MPPED2	5
CEACAM5	5
APOC1P1	5
PWARSN	5
HSH2D	5
TMEM156	5
CORO2A	5
PTPN2	5
RNF222	5
KCNJ3	5
IQCC	5
FLJ30679	5
RNF141	5
ADM2	5

TESPA1	9
RHOBTB3	9
ZNF264	9
LINC00189	9
RGR	9
XIAP	9
AASS	9
OR7D2	9
DIO2	9
MAPK13	9
ARFGEF3	9
TMEM241	9
LOC257396	9
CTSV	9
ACVR2B-AS1	9
TRIM58	9
MTAP	9
GNE	9
OTX2-AS1	9
P3H4	9
CXorf38	9
SAMD5	9
PART1	9
MOG	9
PPIL6	9
LINC00672	9
ADCY2	9
CWF19L1	9
MOK	9
PAQR5	9
SYT15	9
GSTM3	9
FBXO17	9
TACR2	9
CHP2	9
LOC401557	9
PGPEP1	9
ZNF865	9
ABI2	9
IQCH-AS1	9
CES3	9
GCSAML	9
OPRM1	9
LINC00707	9
LOC643201	9
FANCD2	9
CPSF2	9
F2RL3	9
POFUT1	9
RNF14	9
DIS3	9
IDO1	9
TET2	9
TRMT5	9
IFIT1	9
PGM3	9
CLMN	9
IFT22	9
TPMT	9
GOLGA2	9
EMC1	9

ENAH	14
PCYOX1	14
SGCD	14
HSPB8	14
NAV2	14
INAFM1	14
EDIL3	14
GPX8	14
SPSB1	14
SLC14A1	14
CACNA1C	14
ADAMTS2	14
DSEL	14
CSRP2	14
NTRK2	14
CTHRC1	14
ROBO1	14
DIXDC1	14
AKR1C2	14
SLC25A4	14
KITLG	14
PTPN11	14
UBA2	14
RNF24	14
OLFML2A	14
ARHGEF26	14
ERRFI1	14
KIAA1671	14
CRISPLD1	14
THY1	14
NEO1	14
PRUNE2	14
RASAL2	14
UGDH	14
ZNF532	14
TPPP3	14
LMO3	14
ILK	14
RRAGA	14
MT1X	14
SMAD5	14
DIP2C	14
ADCY9	14
CYTH3	14
TMEM159	14
RSU1	14
NLGN2	14
SGIP1	14
CAMK2N1	14
CCL4	15
PTPRC	15
CD44	15
HCST	15
WIPF1	15
PABPC1	15
FYB1	15
FOXP3	15
SORL1	15
PLEK	15
IL2RA	15
PFN1	15

ZC3H12D	5
GPR82	5
FOXE1	5
BLZF1	5
MPV17L	5
ANTXR2	5
KIF1B	5
MREG	5
CXorf21	5
DRAM1	5
CACNG2	5
C8orf33	5
OLMALINC	5
ABL2	5
MACC1	5
TMEM106A	5
TTPAL	5
PCAT1	5
TRPM7	5
SYNPO2L	5
EHHADH	5
RFC2	5
C1orf220	5
C18orf61	5
LRRC37A4P	5
PCSK5	5
CHMP4C	5
ZNF527	5
ARL11	5
LINC01551	5
QRSL1	5
ZYG11B	5
GNAS	6
AHNAK	6
ITM2B	6
H3F3B	6
MYH9	6
FLNA	6
CSDE1	6
ARF1	6
CD81	6
P4HB	6
ITGB1	6
sep-02	6
PEBP1	6
TMEM59	6
CNBP	6
ISCU	6
EID1	6
TAGLN2	6
SRP14	6
PPP1CB	6
NCL	6
REEP5	6
LRP4	6
OSBPL5	6
SPTAN1	6
ARID5B	6
ESD	6
NUCKS1	6
IRF2BP2	6

GGCX	9
BVES	9
GALNT6	9
ZNF669	9
PRRG4	9
SGK494	9
RRP7A	9
SLC4A8	9
TRIM65	9
LOC1005064	9
C9orf66	9
SULT1C2P1	9
SCN2B	9
SLC4A1	9
ZNF492	9
FAM104B	9
TIGAR	9
B3GNT7	9
SHCBP1	9
RFPL1S	9
SRSF12	9
GP6	9
DTWD2	9
LMLN	9
LRPAP1	9
SLC50A1	9
GSN	10
TCF4	10
TIMP1	10
IFITM3	10
CLIC4	10
GAS6	10
FN1	10
ADIRF	10
EPAS1	10
NFIB	10
CD59	10
CAVIN1	10
IGFBP7	10
HTRA1	10
SPARC	10
SERPING1	10
A2M	10
AQP1	10
CRIP2	10
BGN	10
CD9	10
FSTL1	10
SULF1	10
LMNA	10
CAV1	10
ENG	10
HSPB1	10
EFEMP1	10
TGM2	10
COL3A1	10
SPARCL1	10
TM4SF1	10
C1R	10
NR2F2	10
MGP	10

LCP1	15
ARHGDIB	15
ARPC2	15
SRGN	15
LAPTM5	15
PRSS8	15
CD53	15
ACTR2	15
DAZAP2	15
SH3BGRL3	15
ARPC3	15
PFDN5	15
CLIC1	15
CD48	15
CD52	15
TXNIP	15
CYBA	15
LITAF	15
COTL1	15
ITGB2	15
STK17B	15
CD37	15
CELF2	15
RASSF5	15
GMFG	15
HCLS1	15
CAB39	15
KLF5	15
BRD8	15
IFI16	15
PSMB10	15
LIMD2	15
RNASET2	15
CXCR4	15
UCP2	15
GNA13	15
PREX1	15
ARHGAP9	15
ALOX5AP	15
WAS	15
CYTIP	15
IQGAP2	15
PTK2B	15
CX3CR1	15
EFHD2	15
ARL4C	15
SLAMF7	15
MBL2	15
CD300A	15
GPR183	15
MAF	15
MKNK1	15
PRKCB	15
TNFRSF1B	15
STXBP2	15
MYO1F	15
POU2F2	15
SKAP2	15
MYO1G	15
CASP1	15
ATP2B1	15

ACTN4	6
SYPL1	6
PGRMC2	6
GABARAPL2	6
SKIL	6
NUCB2	6
TMEM173	6
SBDS	6
RHEBP1	6
ATP1A1	6
MAGED2	6
SLC41A3	6
SEC62	6
MBNL2	6
IER3IP1	6
TMED10	6
TMEM109	6
RNF11	6
LMAN1	6
LMBRD1	6
TMEM30A	6
CHMP5	6
PDIA3	6
CDIPT	6
CBX6	6
WDR83OS	6
PNPLA2	6
TMEM43	6
MAP4	6
ZSCAN18	6
VEGFB	6
CASC4	6
ARF4	6
REXO2	6
CREB3L2	6
NDFIP1	6
NDUFC1	6
TMED7	6
GRK5	6
CCDC85B	6
SKI	6
TMEM184B	6
PLEC	6
PRAF2	6
EIF5B	6
PTPN12	6
HSPA5	6
NDUFA4	6
DAD1	6
TACC1	6
HSP90AB1	6
TM9SF2	6
TRMT112	6
C1orf43	6
EIF1AX	6
ATF4	6
SERINC1	6
BZW1	6
SRP9	6
SERINC3	6
TMED2	6
IGFBP4	10
LTBP2	10
OGN	10
CYR61	10
CLU	10
CTGF	10
ELN	10
CFH	10
TIMP3	10
YBX3	10
CRIM1	10
VIM	10
S100A6	10
FBLIM1	10
TPM4	10
SPTBN1	10
RAB13	10
PLS3	10
C1S	10
PLAC9	10
TMEM47	10
CDH13	10
FMOD	10
CYBRD1	10
NFIX	10
SELENOM	10
OMD	10
CPE	10
JAG1	10
MRC2	10
LAMB2	10
SYNPO	10
ID3	10
FOXC1	10
LIMCH1	10
GPX3	10
CX3CL1	10
PTPRS	10
RBPMS	10
GNG12	10
TFPI	10
PAM	10
NFIA	10
NNMT	10
PRSS23	10
RRAS	10
PDGFD	10
HEY2	10
EPS8	10
IL33	10
GATA6	10
CCDC3	10
PLPP3	10
CFB	10
WWTR1	10
RBMS3	10
LTBP4	10
PCDH7	10
VCL	10
TCEAL9	10
COL8A1	10
RNF149	15
SHKBP1	15
EMP3	15
SLC39A8	15
TPM3	15
EVI2B	15
DOCK8	15
CD84	15
GRK2	15
CFL1	15
FAM49B	15
KCNAB2	15
BAZ1A	15
GPSM3	15
SLA	15
VAMP8	15
INPP5D	15
IL10RA	15
ARHGAP45	15
LCP2	15
FMNL1	15
ARHGAP30	15
ARHGAP4	15
LSP1	15
DOCK2	15
SAMSN1	15
SELPLG	15
LPXN	15
HMGA1	15
CSK	15
SLC2A3	15
CLEC2B	15
PIK3CD	15
EVI2A	15
PTPN6	15
RNF166	15
PTBP3	15
ITGB2-AS1	15
ADAM8	15
EZR	15
PLCB2	15
ADCY7	15
RGS10	15
APBB1IP	15
REL	15
PARVG	15
SERPINB9	15
SLC4A7	15
AOAH	15
PRR13	15
SASH3	15
GLIPR2	15
CYTH4	15
DOK2	15
CMTM7	15
NCKAP1L	15
RGS19	15
SNX10	15
VAV1	15
IL10RB	15
TCIRG1	15

HNRNPA0	6
RAB2A	6
FKBP2	6
BLCAP	6
PPP2CB	6
GANAB	6
SCP2	6
ANXA7	6
ATRAID	6
TM9SF3	6
SF3B6	6
SEM1	6
SELENOS	6
DYNLRB1	6
ERH	6
VCP	6
KIF5B	6
PRDX6	6
GLG1	6
POLR2L	6
MRPS21	6
NDUFB4	6
SKP1	6
STAU1	6
COX17	6
ZBTB4	6
KIF13B	6
RWDD1	6
SRSF6	6
PIP5K1C	6
MAN2B2	6
BCL2L1	6
CPT1A	6
HMGN3	6
PRKAR1A	6
NENF	6
ADI1	6
ARL1	6
MRPL33	6
MXI1	6
SEC31A	6
MORF4L1	6
PIGT	6
SDF4	6
PKD1	6
ERLEC1	6
PJA2	6
MAGED1	6
CLSTN1	6
DYNC1H1	6
PARK7	6
SAP18	6
PDXDC1	6
ARHGAP1	6
RAB5B	6
CETN2	6
WRNIP1	6
SNU13	6
CCT6A	6
NAA38	6
NOL7	6
GLT8D2	10
NCKAP1	10
COX7A1	10
ASAP2	10
MT1E	10
CTSK	10
SORBS3	10
CTTN	10
PTGIS	10
DST	10
THRB	10
SERPINH1	10
NDN	10
MMP2	10
TSC22D1	10
DCN	10
CYB5R3	10
CTSF	10
TINAGL1	10
CD151	10
OSMR	10
FBLN2	10
RBFOX2	10
MSRB3	10
BEX3	10
NFIC	10
FZD8	10
FGFR1	10
PDLIM7	10
PARVA	10
PROS1	10
CERCAM	10
PDE3A	10
FERMT2	10
PKIG	10
INMT	10
PGRMC1	10
GJA1	10
SCARA3	10
ABI3BP	10
PKD2	10
PBX1	10
CPXM2	10
LMCD1	10
S100A13	10
ITGA10	10
HMCN1	10
EPHX1	10
ZCCHC24	10
EHD2	10
MRFAP1	10
APP	10
CAV2	10
LAMB1	10
AMOTL2	10
CALU	10
PTK2	10
CDH11	10
IGFBP3	10
KDELR2	10
TNS3	10
GPCPD1	15
LRRFIP1	15
ATP5MC2	15
ARHGDIA	15
CAPZB	15
TGFB1	15
TRIR	15
MAPKAPK3	15
GLIPR1	15
HIGD2A	15
ANP32A	15
NDUFS7	15
LFNG	15
TAF10	15
FERMT3	15
MAP2K3	15
SELENOO	15
TSC22D4	15
MOB3A	15
JARID2	15
TYK2	15
SLC7A5P2	15
SH3BP1	15
CAPZA1	15
MX2	15
CCDC88B	15
ST8SIA1	15
ST8SIA4	15
CMTM3	15
MYO9B	15
AMPD3	15
PIK3R5	15
SH3KBP1	15
C6orf62	15
ACTR3	15
FOXN3	15
ROCK1	15
NABP1	15
PLEKHA2	15
GALM	15
APOBR	15
DOCK10	15
PPM1M	15
DENND1B	15
UBALD2	15
HNRNPC	15
PPP1R18	15
IRAK1	15
GGA1	15
AKAP13	15
MICAL1	15
IGFLR1	15
SATB1	15
TAOK3	15
CNN2	15
RCSD1	15
EDEM1	15
CRYBG1	15
ITGA4	15
CD244	15
TIAM1	15

PDCD6	6
SPRYD3	6
USP22	6
SH3GLB1	6
CDKN1B	6
CFDP1	6
CCL5	7
CD8A	7
GZMK	7
GZMA	7
NKG7	7
CD8B	7
CST7	7
GZMH	7
CD3D	7
CTSW	7
APOBEC3G	7
CD2	7

RTL8C	10
EXT1	10
SERPINE2	10
KLF9	10
SASH1	10
SELENOW	10
SCARB2	10
HES1	10
CCND1	10
IL6ST	10
FAM114A1	10
SETBP1	10
DAAM1	10
RTL8A	10
SNX9	10
CDKN2C	10
TCF7L2	10
HSPA1B	10

DENND4B	15
IVNS1ABP	15
UBE2L6	15
RAPGEF1	15
AGTPBP1	15
IDH2	15
MIS18BP1	15
SPCS3	15
CBL	15
APRT	15
PSME2	15
LRCH4	15
ARID1A	15
PTPN1	15
BRD4	15
DPEP2	15
jun-10	
nov-14	

Table S3

FUMA_MAGMA

Index	phenotype	ensg	symbol	chr	start	end	strand	NSNPS	NPARAM	N	ZSTAT	P	type	entrezID	HUGO	pLI	ncRVIS	posMapSI	posMapN	eqtlMapS	eqtlMapn	eqtlMapn	eqtlMapts	eqtlDirect	ciMap	ciMapts	minGwasP	IndSigSNPs
			Gene information						MAGMA results											FUMA mapping								
1	UKBB_CAD	ENSG00000116209	TMEM59	1	5,4E+07	5,5E+07	-1	356	27	323776	0,58549	0,27911	protein_c	9528	TMEM59	2,23E-07	0,206649282	0	0	1	3,54E-06	3,80E-06	GTEx_v7_	+	No	NA	2,84E-10	rs11591147
2	UKBB_CAD	ENSG00000162390	ACOT11	1	5,5E+07	5,5E+07	1	827	71	326700	-0,67823	0,75119	protein_c	26027	ACOT11	1,33E-10	4	1	2	0	NA	NA	NA	NA	No	NA	0,03705	rs11591147
3	UKBB_CAD	ENSG00000162391	FAM151A	1	5,5E+07	5,5E+07	-1	527	38	327166	-0,21829	0,5864	protein_c	338094	FAM151A	3,92E-13	0,726173397	1	2	0	NA	NA	NA	NA	No	NA	0,03705	rs11591147
4	UKBB_CAD	ENSG00000271723	MROH7-T	1	5,5E+07	5,5E+07	1	860	66	326872	-0,71454	0,76255	protein_c	1E+08	MROH7-T	NA	NA	1	2	0	NA	NA	NA	NA	No	NA	0,03705	rs11591147
5	UKBB_CAD	ENSG00000184313	MROH7	1	5,5E+07	5,5E+07	1	762	54	326334	-2	0,93598	protein_c	374977	MROH7	6,65E-19	0,291274148	1	2	0	NA	NA	NA	NA	No	NA	0,03705	rs11591147
6	UKBB_CAD	ENSG00000243725	TTC4	1	5,5E+07	5,5E+07	1	528	47	328596	0,13019	0,44821	protein_c	7268	TTC4	0,04397	1	1	2	0	NA	NA	NA	NA	No	NA	0,03705	rs11591147
7	UKBB_CAD	ENSG00000143001	TMEM61	1	5,5E+07	5,5E+07	1	484	56	328978	3	0,00026	protein_c	199964	TMEM61	0,00438	NA	55	18,29	0	NA	NA	NA	NA	Yes	Mesenchy	2,14E-12	rs11591147;rs34232196;rs56170783:rs61772626
8	UKBB_CAD	ENSG00000162399	BSND	1	5,5E+07	5,5E+07	1	508	61	328016	4	5,34E-05	protein_c	7809	BSND	4,41E-07	3	56	18,29	1	2,56E-07	0,00019	BIOS_eQT	+	No	NA	2,84E-10	rs11591147;rs34232196
9	UKBB_CAD	ENSG00000169174	PCSK9	1	5,6E+07	5,6E+07	1	478	69	325285	4	1,29E-05	protein_c	255738	PCSK9	1,03E-10	0,264270736	56	18,29	0	NA	NA	NA	NA	No	NA	2,84E-10	rs11591147;rs34232196
10	UKBB_CAD	ENSG00000162402	USP24	1	5,6E+07	5,6E+07	-1	803	46	326846	3	0,00076	protein_c	23358	USP24	1	-1	58	18,29	0	NA	NA	NA	NA	Yes	GM12878	2,14E-12	rs34232196;rs11591147;rs56170783:rs61772626:rs56170783;rs61772626
11	UKBB_CAD	ENSG00000162407	PPAP2B	1	5,7E+07	5,7E+07	-1	984	54	330392	5	9,82E-08	protein_c	8613	PPAP2B	0,96183	-0,049380558	161	22,6	11	2,27E-15	0	xQTLServe	+	No	NA	2,14E-12	rs56170783;rs61772626
12	UKBB_CAD	ENSG00000085433	WDR47	1	1,1E+08	1,1E+08	-1	577	45	324677	-0,10623	0,5423	protein_c	22911	WDR47	0,90566	-0,351230971	0	0	0	NA	NA	NA	NA	Yes	IMR90:Me	0,011349	rs7528419
13	UKBB_CAD	ENSG00000197780	TAF13	1	1,1E+08	1,1E+08	-1	389	42	320486	2	0,03427	protein_c	6884	TAF13	0,01985	0,249428817	0	0	1	1,57E-05	0,04327	eQTLGen_	-	Yes	IMR90:Me	0,011349	rs7528419
14	UKBB_CAD	ENSG00000215717	TMEM167	1	1,1E+08	1,1E+08	1	389	40	320333	3	0,00539	protein_c	56900	TMEM167	0,63248	-0,038371693	1	0,638	1	3,33E-08	0,00014	eQTLGen_	+	Yes	IMR90:Me	0,000501	rs7528419
15	UKBB_CAD	ENSG00000179902	C1orf194	1	1,1E+08	1,1E+08	-1	392	42	320968	3	0,00188	protein_c	127003	C1orf194	2,50E-07	NA	2	6	0	NA	NA	NA	NA	Yes	IMR90:Me	0,009666	rs7528419
16	UKBB_CAD	ENSG00000116299	KIAA1324	1	1,1E+08	1,1E+08	1	646	61	322958	3	0,00119	protein_c	57535	KIAA1324	2,15E-08	NA	33	12,64	9	#######	0	BIOS_eQT	+	Yes	IMR90:Me	1,84E-07	rs7528419
17	UKBB_CAD	ENSG00000031698	SARS	1	1,1E+08	1,1E+08	1	399	48	319000	7	3,03E-12	protein_c	6301	SARS	0,99915	-0,332037527	87	26,1	4	7,82E-26	0	BIOS_eQT	-	Yes	IMR90:Me	3,77E-27	rs7528419
18	UKBB_CAD	ENSG00000143126	CELSR2	1	1,1E+08	1,1E+08	1	347	51	316551	8	9,99E-17	protein_c	1952	CELSR2	1	0,807304437	115	26,1	5	4,22E-11	0	BIOS_eQT	+	No	NA	3,77E-27	rs7528419
19	UKBB_CAD	ENSG00000134222	PSRC1	1	1,1E+08	1,1E+08	-1	296	45	313683	9	2,46E-18	protein_c	84722	PSRC1	1,75E-06	-0,208880013	107	26,1	11	#######	0	BIOS_eQT	-	No	NA	3,77E-27	rs7528419
20	UKBB_CAD	ENSG00000221986	MYBPHL	1	1,1E+08	1,1E+08	-1	291	42	312757	9	8,44E-18	protein_c	343263	MYBPHL	0,00462	0,281706372	101	26,1	0	NA	NA	NA	NA	No	NA	3,77E-27	rs7528419
21	UKBB_CAD	ENSG00000134243	SORT1	1	1,1E+08	1,1E+08	-1	378	42	316568	7	7,25E-14	protein_c	6272	SORT1	0,99993	-1	111	25,5	4	2,37E-09	2,60E-05	eQTLGen_	-	Yes	Aorta:GM	3,77E-27	rs7528419
22	UKBB_CAD	ENSG00000143106	PSMA5	1	1,1E+08	1,1E+08	-1	233	16	323899	2	0,02859	protein_c	5686	PSMA5	0,97426	-1	28	18,17	0	NA	NA	NA	NA	Yes	IMR90:Me	1,18E-06	rs7528419
23	UKBB_CAD	ENSG00000143028	SYPL2	1	1,1E+08	1,1E+08	1	269	18	326038	2	0,01793	protein_c	284612	SYPL2	0,01677	0,437866135	21	18,17	5	4,34E-40	5,51E-33	GTEx_v7_	+	Yes	Aorta:GM	1,84E-07	rs7528419
24	UKBB_CAD	ENSG00000162650	ATXN7L2	1	1,1E+08	1,1E+08	1	261	17	326226	2	0,01885	protein_c	127002	ATXN7L2	0,71895	NA	19	18,17	5	5,66E-13	9,00E-09	GTEx_v7_	-	Yes	Aorta:GM	1,84E-07	rs7528419
25	UKBB_CAD	ENSG00000174151	CYB561D1	1	1,1E+08	1,1E+08	1	258	19	325845	2	0,01517	protein_c	284613	CYB561D1	0,01583	-0,395994268	20	18,17	0	NA	NA	NA	NA	Yes	Aorta:GM	1,36E-06	rs7528419
26	UKBB_CAD	ENSG00000181754	AMIGO1	1	1,1E+08	1,1E+08	-1	250	25	325814	2	0,01511	protein_c	57463	AMIGO1	0,88849	-0,212526248	17	18,17	5	5,94E-14	0	BIOS_eQT	-	Yes	IMR90:Me	1,84E-07	rs7528419
27	UKBB_CAD	ENSG00000156097	GPR61	1	1,1E+08	1,1E+08	1	227	31	324785	3	0,0047	protein_c	83873	GPR61	0,80149	-0,586870154	8	16,76	0	NA	NA	NA	NA	No	NA	1,36E-06	rs7528419
28	UKBB_CAD	ENSG00000065135	GNAI3	1	1,1E+08	1,1E+08	1	308	35	326314	3	0,00116	protein_c	2773	GNAI3	0,03881	0,458884037	7	8,57	5	7,78E-79	0	eQTLGen_	-	Yes	IMR90:Me	1,84E-07	rs7528419
29	UKBB_CAD	ENSG00000134183	GNAT2	1	1,1E+08	1,1E+08	-1	232	33	325081	3	0,00242	protein_c	2780	GNAT2	0,00816	-0,092828331	2	8	0	NA	NA	NA	NA	No	NA	0,003483	rs7528419
30	UKBB_CAD	ENSG00000116337	AMPD2	1	1,1E+08	1,1E+08	1	260	31	324520	3	0,00309	protein_c	271	AMPD2	0,00854	-0,430987978	2	8	0	NA	NA	NA	NA	No	NA	0,003483	rs7528419
31	UKBB_CAD	ENSG00000168765	GSTM4	1	1,1E+08	1,1E+08	1	307	47	317970	2	0,01074	protein_c	2948	GSTM4	0,00092	0,373951732	1	8	7	4,99E-52	0	BIOS_eQT	-	Yes	IMR90	1,84E-07	rs7528419
32	UKBB_CAD	ENSG00000213366	GSTM2	1	1,1E+08	1,1E+08	1	384	56	317468	3	0,00426	protein_c	2946	GSTM2	0,0089	-0,190008313	1	8	4	6,28E-25	0	eQTLGen_	-	No	NA	0,000202	rs7528419
33	UKBB_CAD	ENSG00000134184	GSTM1	1	1,1E+08	1,1E+08	1	337	49	316155	2	0,01571	protein_c	2944	GSTM1	0,34571	NA	0	0	9	1,57E-50	0	BIOS_eQT	+	No	NA	1,84E-07	rs7528419
34	UKBB_CAD	ENSG00000134202	GSTM3	1	1,1E+08	1,1E+08	-1	347	47	315561	0,59126	0,27717	protein_c	2947	GSTM3	0,00057	0,168105336	0	0	8	#######	0	BIOS_eQT	-	No	NA	3,32E-05	rs7528419
35	UKBB_CAD	ENSG00000163131	CTSS	1	1,5E+08	1,5E+08	-1	319	16	325977	3	0,00028	protein_c	1520	CTSS	0,62828	-0,390794499	0	0	3	5,85E-07	0,0019	eQTLGen_	-	No	NA	0,010994	rs11810571
36	UKBB_CAD	ENSG00000143387	CTSK	1	1,5E+08	1,5E+08	-1	213	18	322331	3	0,00032	protein_c	1513	CTSK	0,00014	-0,588566526	0	0	5	9,88E-17	0	eQTLGen_	-	No	NA	7,90E-07	rs11810571
37	UKBB_CAD	ENSG00000163141	BNIPL	1	1,5E+08	1,5E+08	1	314	40	322089	2	0,04063	protein_c	149428	BNIPL	1,03E-09	0,962108931	0	0	5	7,82E-13	0	eQTLGen_	+	No	NA	7,90E-07	rs11810571
38	UKBB_CAD	ENSG00000197622	CDC42SE1	1	1,5E+08	1,5E+08	-1	313	36	322568	1	0,06947	protein_c	56882	CDC42SE1	0,01352	-0,166256064	0	0	1	9,10E-06	0,02574	eQTLGen_	-	No	NA	0,009271	rs11810571
39	UKBB_CAD	ENSG00000143458	GABPB2	1	1,5E+08	1,5E+08	1	371	33	320191	1	0,11999	protein_c	126626	GABPB2	0,00263	0,476201693	1	9	0	NA	NA	NA	NA	No	NA	0,001434	rs11810571
40	UKBB_CAD	ENSG00000143434	SEMA6C	1	1,5E+08	1,5E+08	-1	221	34	319629	1	0,13393	protein_c	10500	SEMA6C	5,65E-05	-0,756766598	1	9	0	NA	NA	NA	NA	No	NA	0,001434	rs11810571
41	UKBB_CAD	ENSG00000163154	TNFAIP8L	1	1,5E+08	1,5E+08	1	181	34	317583	2	0,06664	protein_c	79626	TNFAIP8L	0,50473	-0,485809395	1	9	0	NA	NA	NA	NA	No	NA	0,001434	rs11810571
42	UKBB_CAD	ENSG00000163156	SCNM1	1	1,5E+08	1,5E+08	1	203	36	318441	2	0,0508	protein_c	79005	SCNM1	4,73E-05	-0,377994046	1	9	0	NA	NA	NA	NA	No	NA	0,001434	rs11810571
43	UKBB_CAD	ENSG00000163155	LYSMD1	1	1,5E+08	1,5E+08	-1	190	36	317676	2	0,04786	protein_c	388695	LYSMD1	0,18195	-0,634866856	1	9	0	NA	NA	NA	NA	No	NA	0,001434	rs11810571
44	UKBB_CAD	ENSG00000163157	TMOD4	1	1,5E+08	1,5E+08	-1	180	35	318508	2	0,05449	protein_c	29765	TMOD4	1,33E-07	-0,283548368	1	9	0	NA	NA	NA	NA	No	NA	0,001434	rs11810571
45	UKBB_CAD	ENSG00000163159	VPS72	1	1,5E+08	1,5E+08	-1	210	36	319348	2	0,04499	protein_c	6944	VPS72	0,24943	-0,425641667	1	9	0	NA	NA	NA	NA	No	NA	0,001434	rs11810571
46	UKBB_CAD	ENSG00000143398	PIP5K1A	1	1,5E+08	1,5E+08	1	210	27	318800	2	0,06004	protein_c	8394	PIP5K1A	0,01459	-0,371333534	1	9	0	NA	NA	NA	NA	No	NA	0,001434	rs11810571
47	UKBB_CAD	ENSG00000143393	PI4KB	1	1,5E+08	1,5E+08	-1	216	29	318330	0,9632	0,16772	protein_c	5298	PI4KB	0,99872	-0,310213069	0	0	0	NA	NA	NA	NA	Yes	Mesenchy	0,012047	rs11810571
48	UKBB_CAD	ENSG00000143390	RFX5	1	1,5E+08	1,5E+08	-1	177	31	319725	0,75456	0,22526	protein_c	5993	RFX5	0,00014	-0,738997583	0	0	0	NA	NA	NA	NA	Yes	Mesenchy	0,012047	rs11810571
49	UKBB_CAD	ENSG00000159377	PSMB4	1	1,5E+08	1,5E+08	1	191	25	322056	0,18241	0,42763	protein_c	5692	PSMB4	0,39881	-0,145832206	0	0	0	NA	NA	NA	NA	Yes	IMR90:Me	0,012047	rs11810571
50	UKBB_CAD	ENSG00000143442	POGZ	1	1,5E+08	1,5E+08	-1	277	25	322713	0,16578	0,43417	protein_c	23126	POGZ	1	-0,430760681	0	0	0	NA	NA	NA	NA	Yes	IMR90:Me	0,012047	rs11810571
51	UKBB_CAD	ENSG00000143375	CGN	1	1,5E+08	1,5E+08	1	265	33	320226	0,85901	0,19517	protein_c	57530	CGN	1,82E-22	-0,177275847	35	15,67	0	NA	NA	NA	NA	Yes	IMR90:Me	0,000285	rs11810571
52	UKBB_CAD	ENSG00000143367	TUFT1	1	1,5E+08	1,5E+08	1	292	38	322047	2	0,03299	protein_c	7286	TUFT1	0,01808	-0,311947419	60	15,67	1	5,30E-06	0,01525	eQTLGen_	-	Yes	IMR90:Me	0,000285	rs11810571
53	UKBB_CAD	ENSG00000143376	SNX27	1	1,5E+08	1,5E+08	1	353	30	326008	3	0,00344	protein_c	81609	SNX27	0,99988	0,907633168	103	19,71	6	3,30E-54	0	BIOS_eQT	+	Yes	GM12878	7,90E-07	rs11810571
54	UKBB_CAD	ENSG00000159409	CELF3	1	1,5E+08	1,5E+08	-1	225	25	324905	2	0,00844	protein_c	11189	CELF3	0,29314	0,169820041	48	19,71	0	NA	NA	NA	NA	Yes	IMR90:Me	1,09E-07	rs11810571
55	UKBB_CAD	ENSG00000178796	RIIAD1	1	1,5E+08	1,5E+08	1	248	25	325905	3	0,0028	protein_c	284485	RIIAD1	NA	NA	59	19,71	1	2,58E-13	1,56E-05	xQTLServe	NA	No	NA	1,09E-07	rs11810571
56	UKBB_CAD	ENSG00000240510	AL589765	1	1,5E+08	1,5E+08	1	248	25	325905	3	0,0028	protein_c	NA	NA	NA	NA	59	19,71	0	NA	NA	NA	NA	No	NA	1,09E-07	rs11810571
57	UKBB_CAD	ENSG00000143436	MRPL9	1	1,5E+08	1,5E+08	-1	260	18	328090	4	4,15E-05	protein_c	65005	MRPL9	0,05131	-0,632687464	89	20,6	7	7,10E-11	0	BIOS_eQT	+	Yes	GM12878	2,21E-08	rs11810571
58	UKBB_CAD	ENSG00000143450	OAZ3	1	1,5E+08	1,5E+08	1	275	19	327426	4	1,88E-05	protein_c	51686	OAZ3	9,17E-05	0,187899691	98	20,6	7	1,84E-12	0	BIOS_eQT	+	Yes	GM12878	2,21E-08	rs11810571
59	UKBB_CAD	ENSG00000182134	TDRKH	1	1,5E+08	1,5E+08	-1	312	26	327460	5	1,82E-06	protein_c	11022	TDRKH	0,00696	0,365422897	112	20,6	10	3.2717000	0	BIOS_eQT	+	No	NA	2,21E-08	rs11810571
60	UKBB_CAD	ENSG00000213171	LINGO4	1	1,5E+08	1,5E+08	-1	288	31	327635	4	2,31E-05	protein_c	339398	LINGO4	0,00285	-0,028421878	88	20,6	6	2,81E-20	0	BIOS_eQT	+	No	NA	2,21E-08	rs11810571
61	UKBB_CAD	ENSG00000143365	RORC	1	1,5E+08	1,5E+08	-1	341	39	327644	4	6,12E-05	protein_c	6097	RORC	0,99785	-0,558270634	87	20,6	2	1,45E-08	2,33E-05	BIOS_eQT	-	No	NA	2,21E-08	rs11810571
62	UKBB_CAD	ENSG00000225556	C2CD4D	1	1,5E+08	1,5E+08	-1	262	36	327747	4	7,68E-06	protein_c	1E+08	C2CD4D	NA	0,095564455	58	20,6	0	NA	NA	NA	NA	Yes	IMR90:Me	2,21E-08	rs11810571
63	UKBB_CAD	ENSG00000196407	THEM5	1	1,5E+08	1,5E+08	-1	269	35	326899	3	0,00108	protein_c	284486	THEM5	5,98E-07	-0,032711685	47	20,6	1	1,08E-05	0,03029	eQTLGen_	-	No	NA	5,21E-08	rs11810571
64	UKBB_CAD	ENSG00000159445	THEM4	1	1,5E+08	1,5E+08	-1	377	26	328862	0,13842	0,44496	protein_c	117145	THEM4	0,00024	0,737769726	15	10	8	1,94E-27	0	eQTLGen_	-	Yes	IMR90	7,90E-07	rs11810571
65	UKBB_CAD	ENSG00000197747	S100A10	1	1,5E+08	1,5E+08	-1	322	19	331011	-0,40273	0,65643	protein_c	6281	S100A10	0,17325	-0,60763383	0	0	0	NA	NA	NA	NA	Yes	IMR90:Me	0,012047	rs11810571
66	UKBB_CAD	ENSG00000163191	S100A11	1	1,5E+08	1,5E+08	-1	198	29	325393	-0,00948	0,50378	protein_c	6282	S100A11	0,05011	-0,225330308	1	0,555	0	NA	NA	NA	NA	No	NA	0,027292	rs11810571
67	UKBB_CAD	ENSG00000182898	TCHHL1	1	1,5E+08	1,5E+08	-1	104	24	315083	0,00783	0,49688	protein_c	126637	TCHHL1	2,23E-08	-0,433542251	1	0,555	0	NA	NA	NA	NA	No	NA	0,027292	rs11810571
68	UKBB_CAD	ENSG00000215853	RPTN	1	1,5E+08	1,5E+08	-1	188	23	320343	0,17255	0,4315	protein_c	126638	RPTN	3,18E-18	-0,652485756	1	14,59	0	NA	NA	NA	NA	No	NA	0,034322	rs11810571
69	UKBB_CAD	ENSG00000197915	HRNR	1	1,5E+08	1,5E+08	-1	351	13	324012	-0,00789	0,50315	protein_c	388697	HRNR	5,01E-29	1	1	14,59	0	NA	NA	NA	NA	No	NA	0,034322	rs11810571
70	UKBB_CAD	ENSG00000189171	S100A13	1	1,5E+08	1,5E+08	-1	199	20	304868	0,49345	0,31085	protein_c	6284	S100A13	0,03515	-0,436440334	0	0	1	4,04E-06	0,01167	eQTLGen_	-	No	NA	0,021083	rs6689306
71	UKBB_CAD	ENSG00000198837	DENND4B	1	1,5E+08	1,5E+08	-1	185	17	324001	1	0,09465	protein_c	9909	DENND4B	0,99999	NA	0	0	1	1,19E-06	0,00357	eQTLGen_	-	No	NA	0,021083	rs6689306
72	UKBB_CAD	ENSG00000143552	NUP210L	1	1,5E+08	1,5E+08	-1	392	24	324743	0,06755	0,47307	protein_c	91181	NUP210L	0,14464	0,363113447	0	0	1	8,91E-07	0,00281	eQTLGen_	+	No	NA	4,67E-06	rs6689306
73	UKBB_CAD	ENSG00000143595	AQP10	1	1,5E+08	1,5E+08	1	309	25	330214	0,34993	0,36319	protein_c	89872	AQP10	0,01528	-0,980404431	1	0,622	1	3,32E-10	0	eQTLGen_	+	Yes	GM12878	4,67E-06	rs6689306
74	UKBB_CAD	ENSG00000143515	ATP8B2	1	1,5E+08	1,5E+08	1	418	29	330912	0,35172	0,36252	protein_c	57198	ATP8B2	0,99956	-0,493030255	3	0,622	0	NA	NA	NA	NA	Yes	GM12878	4,67E-06	rs6689306
75	UKBB_CAD	ENSG00000160712	IL6R	1	1,5E+08	1,5E+08	1	483	39	328672	5	1,90E-06	protein_c	3570	IL6R	0,04787	-0,79334249	134	13,41	2	3,68E-13	0	GTEx_v7_	+	No	NA	1,46E-09	rs6689306
76	UKBB_CAD	ENSG00000169291	SHE	1	1,5E+08	1,5E+08	-1	382	21	329121	5	2,17E-06	protein_c	126669	SHE	0,72162	-1	194	14,09	1	2,03E-07	0,00013	BIOS_eQT	+	No	NA	1,46E-09	rs6689306
77	UKBB_CAD	ENSG00000163239	TDRD10	1	1,5E+08	1,5E+08	1	371	22	329145	3	0,00109	protein_c	126668	TDRD10	2,45E-06	-0,428923944	132	14,09	0	NA	NA	NA	NA	No	NA	6,84E-09	rs6689306

78	UKBB_CAD	ENSG00000160714	UBE2Q1	1	1,5E+08	1,5E+08	-1	308	25	329473	3	0,00145	protein_c	55585	UBE2Q1	0,96936	-0,5126963	84	14,09	2	2,97E-33	0	eQTLGen	-	Yes	IMR90:Me	4,67E-06	rs6689306
79	UKBB_CAD	ENSG00000160716	CHRNB2	1	1,5E+08	1,5E+08	1	305	26	329478	3	0,00116	protein_c	1141	CHRNB2	0,72025	-0,35158637	73	14,09	0	NA	NA	NA	NA	Yes	IMR90:Me	4,67E-06	rs6689306
80	UKBB_CAD	ENSG00000160710	ADAR	1	1,5E+08	1,5E+08	-1	340	30	327932	3	0,00062	protein_c	103	ADAR	0,9076	-0,566599759	59	14,09	0	NA	NA	NA	NA	Yes	Mesenchy	4,67E-06	rs6689306
81	UKBB_CAD	ENSG00000268141	AL606500	1	1,5E+08	1,5E+08	1	216	24	327036	2	0,00982	protein_c	NA	NA	NA	NA	5	7	0	NA	NA	NA	NA	No	NA	6,34E-05	rs6689306
82	UKBB_CAD	ENSG00000143603	KCNN3	1	1,5E+08	1,5E+08	-1	777	112	329362	2	0,0146	protein_c	3782	KCNN3	0,86494	-0,629504502	2	4	0	NA	NA	NA	NA	Yes	IMR90:Me	4,67E-06	rs6689306
83	UKBB_CAD	ENSG00000160685	ZBTB7B	1	1,5E+08	1,5E+08	1	183	40	309786	1	0,06844	protein_c	51043	ZBTB7B	0,83739	NA	0	0	0	NA	NA	NA	NA	Yes	IMR90:Me	4,67E-06	rs6689306
84	UKBB_CAD	ENSG00000118873	RAB3GAP	1	2,2E+08	2,2E+08	-1	600	36	328988	-0,43547	0,66839	protein_c	25782	RAB3GAP	0,99874	-1	0	0	0	NA	NA	NA	NA	Yes	IMR90:Me	2,28E-05	rs67180937
85	UKBB_CAD	ENSG00000162817	C1orf115	1	2,2E+08	2,2E+08	1	385	25	329486	-0,31454	0,62344	protein_c	79762	C1orf115	0,28364	2	0	0	0	NA	NA	NA	NA	Yes	IMR90:hE	2,28E-05	rs67180937
86	UKBB_CAD	ENSG00000186205	mrt-01	1	2,2E+08	2,2E+08	1	419	42	328461	2	0,01987	protein_c	64757	mrt-01	8,20E-05	0,263393569	0	0	0	NA	NA	NA	NA	Yes	IMR90:Me	2,28E-05	rs67180937
87	UKBB_CAD	ENSG00000136630	HLX	1	2,2E+08	2,2E+08	1	283	26	329347	2	0,02236	protein_c	3142	HLX	0,56122	0,873925502	0	0	0	NA	NA	NA	NA	Yes	Mesenchy	2,28E-05	rs67180937
88	UKBB_CAD	ENSG00000143507	DUSP10	1	2,2E+08	2,2E+08	-1	569	48	331009	0,27071	0,39331	protein_c	11221	DUSP10	0,49952	-0,178838857	0	0	0	NA	NA	NA	NA	Yes	IMR90	2,28E-05	rs67180937
89	UKBB_CAD	ENSG00000143512	HHIPL2	1	2,2E+08	2,2E+08	-1	322	27	278886	5	1,36E-07	protein_c	79802	HHIPL2	1,14E-17	0,034392812	96	13,57	10	1,26E-12	0	BIOS_eQT	+	No	NA	9,47E-14	rs67180937
90	UKBB_CAD	ENSG00000143498	TAF1A	1	2,2E+08	2,2E+08	-1	384	34	282968	7	1,24E-11	protein_c	9015	TAF1A	6,60E-07	-0,09265048	192	19,55	15	#######	0	BIOS_eQT	+	Yes	IMR90:hE	9,47E-14	rs67180937
91	UKBB_CAD	ENSG00000154305	MIA3	1	2,2E+08	2,2E+08	1	340	25	284916	8	9,96E-15	protein_c	375056	MIA3	0,47139	-0,616197151	256	19,55	11	6,02E-14	0	BIOS_eQT	+	Yes	IMR90:hE	8,45E-14	rs67180937
92	UKBB_CAD	ENSG00000186063	AIDA	1	2,2E+08	2,2E+08	-1	291	19	279036	7	3,41E-12	protein_c	64853	AIDA	0,08237	-1	213	19,55	10	6,02E-14	0	BIOS_eQT	+	Yes	GM12878	8,45E-14	rs67180937
93	UKBB_CAD	ENSG00000162819	BROX	1	2,2E+08	2,2E+08	1	262	20	281562	7	5,00E-12	protein_c	148362	BROX	2,48E-05	1	194	19,1	7	1,12E-45	0	GTEx_v7_	-	Yes	GM12878	9,47E-14	rs67180937
94	UKBB_CAD	ENSG00000197520	FAM177B	1	2,2E+08	2,2E+08	1	249	26	281314	6	1,93E-10	protein_c	400823	FAM177B	9,21E-06	-0,425513654	173	19,1	17	3.2717000	0	BIOS_eQT	+	No	NA	9,47E-14	rs67180937
95	UKBB_CAD	ENSG00000154309	DISP1	1	2,2E+08	2,2E+08	1	968	54	272494	4	9,10E-05	protein_c	84976	DISP1	4,23E-06	-0,23613449	119	15,18	3	1,13E-13	0	eQTLGen	-	Yes	IMR90:Me	1,98E-09	rs67180937
96	UKBB_CAD	ENSG00000187554	TLR5	1	2,2E+08	2,2E+08	-1	451	23	303216	0,73456	0,2313	protein_c	7100	TLR5	5,69E-09	-0,227899441	0	0	0	NA	NA	NA	NA	Yes	IMR90:Me	0,001277	rs67180937
97	UKBB_CAD	ENSG00000143502	SUSD4	1	2,2E+08	2,2E+08	-1	603	37	315401	0,9312	0,17588	protein_c	55061	SUSD4	0,00457	-1	0	0	0	NA	NA	NA	NA	Yes	Mesenchy	0,001277	rs67180937
98	UKBB_CAD	ENSG00000143514	TP53BP2	1	2,2E+08	2,2E+08	-1	400	22	326980	-0,7391	0,77008	protein_c	7159	TP53BP2	0,00202	NA	0	0	0	NA	NA	NA	NA	Yes	IMR90:Me	0,001277	rs67180937
99	UKBB_CAD	ENSG00000143771	CNIH4	1	2,2E+08	2,2E+08	1	260	16	326782	-0,44077	0,67031	protein_c	29097	CNIH4	0,08472	0,734052539	0	0	0	NA	NA	NA	NA	Yes	Mesenchy	2,28E-05	rs67180937
100	UKBB_CAD	ENSG00000170745	KCNS3	2	1,8E+07	1,9E+07	1	2658	158	329707	-0,15514	0,56164	protein_c	3790	KCNS3	0,01609	-0,035368259	0	0	0	NA	NA	NA	NA	Yes	IMR90:Me	0,020198	rs16986953
101	UKBB_CAD	ENSG00000240857	RDH14	2	1,9E+07	1,9E+07	-1	328	39	328259	0,00179	0,49928	protein_c	57665	RDH14	0,23294	-0,65948759	0	0	0	NA	NA	NA	NA	Yes	IMR90:Me	0,020198	rs16986953
102	UKBB_CAD	ENSG00000143867	OSR1	2	2E+07	2E+07	-1	311	33	328123	-0,23681	0,5936	protein_c	130497	OSR1	0,77476	-2	0	0	2	3,12E-06	0,00684	GTEx_v7_	-	No	NA	4,77E-10	rs16986953
103	UKBB_CAD	ENSG00000183891	TTC32	2	2E+07	2E+07	-1	324	40	329431	0,65599	0,25591	protein_c	130502	TTC32	0,0899	0,090613208	1	2	0	NA	NA	NA	NA	Yes	IMR90	0,017795	rs16986953
104	UKBB_CAD	ENSG00000118961	C2orf43	2	2,1E+07	2,1E+07	-1	639	72	325040	-0,30357	0,61927	protein_c	60526	C2orf43	0,00065	-0,605069014	1	7,1	2	5,84E-30	0	eQTLGen	+	No	NA	0,010389	rs585967
105	UKBB_CAD	ENSG00000084674	APOB	2	2,1E+07	2,1E+07	-1	358	40	318354	5	1,23E-07	protein_c	338	APOB	5,49E-16	-0,34689194	68	23	1	3,12E-05	0,04232	GTEx_v7_	-	No	NA	2,76E-08	rs585967
106	UKBB_CAD	ENSG00000218819	TDRD15	2	2,1E+07	2,1E+07	1	434	20	330559	5	1,07E-07	protein_c	1E+08	TDRD15	NA	NA	231	14,96	0	NA	NA	NA	NA	No	NA	3,54E-08	rs585967
107	UKBB_CAD	ENSG00000115970	THADA	2	4,3E+07	4,4E+07	-1	1749	56	327441	5	4,97E-07	protein_c	63892	THADA	1,24E-28	-0,379900358	2	11,39	0	NA	NA	NA	NA	No	NA	NA	rs76866386
108	UKBB_CAD	ENSG00000152518	ZFP36L2	2	4,3E+07	4,3E+07	-1	259	35	325849	4	4,27E-06	protein_c	678	ZFP36L2	0,45643	-0,440540073	1	11,39	0	NA	NA	NA	NA	No	NA	NA	rs76866386
109	UKBB_CAD	ENSG00000152527	PLEKHH2	2	4,4E+07	4,4E+07	1	961	57	329975	3	0,00072	protein_c	130271	PLEKHH2	1,79E-26	0,460070846	77	18,1	0	NA	NA	NA	NA	No	NA	0,005042	rs76866386
110	UKBB_CAD	ENSG00000138036	DYNC2LI1	2	4,4E+07	4,4E+07	1	578	39	327124	4	7,50E-06	protein_c	51626	DYNC2LI1	8,38E-05	1	120	23,8	3	8,74E-07	6,34E-09	GTEx_v7_	+	No	NA	5,65E-10	rs76866386;rs4299376
111	UKBB_CAD	ENSG00000138075	ABCG5	2	4,4E+07	4,4E+07	-1	666	37	326765	5	1,81E-07	protein_c	64240	ABCG5	1,90E-15	1	134	23,8	0	NA	NA	NA	NA	No	NA	5,65E-10	rs76866386;rs4299376
112	UKBB_CAD	ENSG00000143921	ABCG8	2	4,4E+07	4,4E+07	1	676	52	326269	5	1,99E-06	protein_c	64241	ABCG8	3,75E-16	-0,050037409	112	23,8	0	NA	NA	NA	NA	No	NA	5,65E-10	rs76866386;rs4299376
113	UKBB_CAD	ENSG00000138095	LRPPRC	2	4,4E+07	4,4E+07	-1	962	40	327073	3	0,00461	protein_c	10128	LRPPRC	0,00043	-0,577712281	52	23,8	0	NA	NA	NA	NA	No	NA	5,65E-10	rs4299376;rs76866386
114	UKBB_CAD	ENSG00000152284	TCF7L1	2	8,5E+07	8,6E+07	1	1092	81	322970	-0,21366	0,58459	protein_c	83439	TCF7L1	0,75375	NA	0	0	0	NA	NA	NA	NA	Yes	IMR90:Tro	2,32E-12	rs7568458
115	UKBB_CAD	ENSG00000152291	TGOLN2	2	8,6E+07	8,6E+07	-1	394	41	318738	1	0,13379	protein_c	10618	TGOLN2	0,00018	0,987165954	0	0	5	1,03E-10	0	eQTLGen	-	Yes	GM12878	2,32E-12	rs7568458
116	UKBB_CAD	ENSG00000042445	RETSAT	2	8,6E+07	8,6E+07	-1	415	35	317522	0,60736	0,2718	protein_c	54884	RETSAT	2,05E-10	1	0	0	11	1,34E-28	0	BIOS_eQT	-	Yes	IMR90	2,32E-12	rs7568458
117	UKBB_CAD	ENSG00000115459	ELMOD3	2	8,6E+07	8,6E+07	1	498	42	318880	0,65944	0,25481	protein_c	84173	ELMOD3	4,56E-11	-0,039152959	0	0	0	NA	NA	NA	NA	Yes	IMR90	2,32E-12	rs7568458
118	UKBB_CAD	ENSG00000042493	CAPG	2	8,6E+07	8,6E+07	-1	428	42	321447	3	0,00463	protein_c	822	CAPG	5,33E-09	-0,25125754	6	8	4	7,68E-22	0	BIOS_eQT	+	Yes	IMR90:Me	2,32E-12	rs7568458
119	UKBB_CAD	ENSG00000152292	SH2D6	2	8,6E+07	8,6E+07	1	431	49	322740	4,12	1,89E-05	protein_c	284948	SH2D6	0,0037	-0,172465518	26	8	3	1,68E-05	0,01093	BIOS_eQT	-	Yes	IMR90:Me	2,32E-12	rs7568458
120	UKBB_CAD	ENSG00000168906	MAT2A	2	8,6E+07	8,6E+07	1	377	24	322774	7	1,92E-12	protein_c	4144	MAT2A	0,73323	-1	186	20,9	7	1,87E-26	0	BIOS_eQT	-	Yes	GM12878	2,39E-13	rs7568458
121	UKBB_CAD	ENSG00000115486	GGCX	2	8,6E+07	8,6E+07	-1	391	27	321606	7	2,75E-13	protein_c	2677	GGCX	1,20E-08	-0,045096532	185	20,9	13	3.2717000	0	xQTLServe	+	Yes	GM12878	2,39E-13	rs7568458
122	UKBB_CAD	ENSG00000118640	VAMP8	2	8,6E+07	8,6E+07	1	361	27	320725	7	3,68E-13	protein_c	8673	VAMP8	0,27049	-0,056335469	172	20,9	12	3.2717000	0	eQTLGen	-	Yes	GM12878	2,39E-13	rs7568458
123	UKBB_CAD	ENSG00000168899	VAMP5	2	8,6E+07	8,6E+07	1	295	29	319151	7	8,05E-14	protein_c	10791	VAMP5	0,05162	0,554615104	134	20,9	13	3.2717000	0	BIOS_eQT	-	Yes	IMR90:Me	2,39E-13	rs7568458
124	UKBB_CAD	ENSG00000168894	RNF181	2	8,6E+07	8,6E+07	1	268	28	319502	7	9,85E-12	protein_c	51255	RNF181	3,14E-09	-0,10549856	116	20,9	6	4,39E-11	0	BIOS_eQT	-	Yes	IMR90:Me	2,39E-13	rs7568458
125	UKBB_CAD	ENSG00000168890	TMEM150	2	8,6E+07	8,6E+07	-1	265	28	319533	7	1,67E-11	protein_c	129303	TMEM150	0,14571	-0,552878007	120	20,9	10	2,45E-29	0	BIOS_eQT	-	Yes	IMR90:Me	2,39E-13	rs7568458
126	UKBB_CAD	ENSG00000168883	USP39	2	8,6E+07	8,6E+07	1	387	43	320056	6	9,61E-11	protein_c	10713	USP39	0,03161	-0,577551893	128	20,9	11	#######	0	xQTLServe	-	Yes	IMR90:Me	2,39E-13	rs7568458
127	UKBB_CAD	ENSG00000168887	C2orf68	2	8,6E+07	8,6E+07	-1	251	35	320370	7	3,84E-11	protein_c	388969	C2orf68	0,01249	-1	111	20,9	11	4,71E-19	0	BIOS_eQT	-	Yes	IMR90:Me	2,39E-13	rs7568458
128	UKBB_CAD	ENSG00000168878	SFTPB	2	8,6E+07	8,6E+07	-1	294	34	320290	4	0,00019	protein_c	6439	SFTPB	0,04074	-0,143858929	49	10	0	NA	NA	NA	NA	No	NA	1,85E-05	rs7568458
129	UKBB_CAD	ENSG00000115523	GNLY	2	8,6E+07	8,6E+07	1	337	35	322165	2	0,00708	protein_c	10578	GNLY	0,00155	1	32	10	12	#######	0	BIOS_eQT	+	Yes	GM12878	2,32E-12	rs7568458
130	UKBB_CAD	ENSG00000168874	ATOH8	2	8,6E+07	8,6E+07	1	472	66	325005	0,32767	0,37158	protein_c	84913	ATOH8	0,8882	NA	0	0	6	6,64E-44	0	BIOS_eQT	+	No	NA	2,32E-12	rs7568458
131	UKBB_CAD	ENSG00000115525	ST3GAL5	2	8,6E+07	8,6E+07	-1	573	30	326233	0,84621	0,19872	protein_c	8869	ST3GAL5	0,02785	-0,500757054	0	0	2	1,09E-06	0,00334	eQTLGen	+	Yes	IMR90	6,57E-12	rs7568458
132	UKBB_CAD	ENSG00000068654	POLR1A	2	8,6E+07	8,6E+07	-1	545	29	318234	4	0,00018	protein_c	25885	POLR1A	0,99986	-2	0	0	0	NA	NA	NA	NA	Yes	IMR90:Me	6,57E-12	rs7568458
133	UKBB_CAD	ENSG00000132300	PTCD3	2	8,6E+07	8,6E+07	1	414	21	320167	3	0,00225	protein_c	55037	PTCD3	1,03E-06	0,826214551	0	0	0	NA	NA	NA	NA	Yes	IMR90:Me	6,57E-12	rs7568458
134	UKBB_CAD	ENSG00000115993	TRAK2	2	2E+08	2E+08	-1	390	24	321086	-0,27108	0,60684	protein_c	66008	TRAK2	6,92E-10	-1	0	0	0	NA	NA	NA	NA	Yes	Mesenchy	0,00097	rs114123510
135	UKBB_CAD	ENSG00000003393	ALS2	2	2E+08	2E+08	-1	585	43	320924	-0,52563	0,70043	protein_c	57679	ALS2	0,00077	-0,243554293	0	0	1	6,40E-10	0	eQTLGen	+	Yes	IMR90:Me	0,000684	rs114123510
136	UKBB_CAD	ENSG00000155760	FZD7	2	2E+08	2E+08	1	329	30	325097	-0,65261	0,743	protein_c	8324	FZD7	0,7302	-0,133052921	1	20,2	0	NA	NA	NA	NA	Yes	IMR90	0,00097	rs114123510
137	UKBB_CAD	ENSG00000182329	AC079354	2	2E+08	2E+08	1	613	39	325514	2	0,01017	protein_c	1E+08	KIAA2012	NA	NA	100	18,52	0	NA	NA	NA	NA	No	NA	5,69E-07	rs114123510
138	UKBB_CAD	ENSG00000116030	SUMO1	2	2E+08	2E+08	-1	289	19	324078	3	0,00169	protein_c	7341	SUMO1	0,80037	-0,848299364	73	18,52	2	8,77E-07	0,00278	BIOS_eQT	+	Yes	IMR90:Me	5,69E-07	rs114123510
139	UKBB_CAD	ENSG00000267889	AC079354	2	2E+08	2E+08	1	225	20	324597	3	0,00409	protein_c	NA	NA	NA	NA	55	18,21	0	NA	NA	NA	NA	No	NA	1,24E-06	rs114123510
140	UKBB_CAD	ENSG00000055044	NOP58	2	2E+08	2E+08	1	290	16	322610	2	0,0355	protein_c	51602	NOP58	0,50948	0,121100695	38	13,15	17	8,08E-27	0	eQTLGen	-	Yes	IMR90:Me	5,16E-19	rs114123510
141	UKBB_CAD	ENSG00000204217	BMPR2	2	2E+08	2E+08	1	543	26	320029	1	0,09348	protein_c	659	BMPR2	0,99667	-4	75	21,2	19	2,12E-52	0	eQTLGen	-	Yes	Mesenchy	5,16E-19	rs114123510
142	UKBB_CAD	ENSG00000138439	FAM117B	2	2E+08	2E+08	1	374	37	319227	6	1,26E-10	protein_c	150864	FAM117B	0,06739	-0,487936546	132	18,41	30	3.2717000	0	BIOS_eQT	-	No	NA	5,16E-19	rs114123510
143	UKBB_CAD	ENSG00000163596	ICA1L	2	2E+08	2E+08	-1	427	27	321235	8	6,52E-17	protein_c	130026	ICA1L	0,44203	-0,760182865	246	18,32	24	#######	0	xQTLServe	+	No	NA	4,48E-19	rs114123510
144	UKBB_CAD	ENSG00000138442	WDR12	2	2E+08	2E+08	-1	503	19	322928	8	1,46E-17	protein_c	55759	WDR12	0,60901	-0,727801816	344	18,32	0	NA	NA	NA	NA	No	NA	2,88E-19	rs114123510
145	UKBB_CAD	ENSG00000138380	CARF	2	2E+08	2E+08	1	370	17	322357	8	3,22E-16	protein_c	79800	CARF	0,00454	-1	253	18,32	23	7,32E-12	1,24E-08	BIOS_eQT	-	No	NA	2,88E-19	rs114123510
146	UKBB_CAD	ENSG00000144426	NBEAL1	2	2E+08	2E+08	1	551	23	321487	7	1,23E-11	protein_c	65065	NBEAL1	1,09E-10	-2	244	17,35	21	1,67E-46	5,44E-40	GTEx_v7_	-	No	NA	2,88E-19	rs114123510
147	UKBB_CAD	ENSG00000119004	CYP20A1	2	2E+08	2E+08	1	301	23	321904	5	2,63E-06	protein_c	57404	CYP20A1	7,41E-10	-0,358177346	77	22,9	0	NA	NA	NA	NA	No	NA	8,56E-17	rs114123510
148	UKBB_CAD	ENSG00000138443	ABI2	2	2E+08	2E+08	1	377	28	320798	5	2,13E-06	protein_c	10152	ABI2	0,95333	-2	96	22,9	0	NA	NA	NA	NA	No	NA	4,86E-15	rs114123510
149	UKBB_CAD	ENSG00000173166	RAPH1	2	2E+08	2E+08	-1	439	32	321500	4	2,06E-05	protein_c	65059	RAPH1	0,95785	-0,345684761	115	18,94	8	6,15E-08	0,00027	eQTLGen	-	No	NA	1,19E-10	rs114123510
150	UKBB_CAD	ENSG00000178562	CD28	2	2E+08	2E+08	1	399	33	320914	0,64647	0,25899	protein_c	940	CD28	0,51199	-0,824137128	3	4,42	0	NA	NA	NA	NA	No	NA	1,56E-05	rs114123510
151	UKBB_CAD	ENSG00000163599	CTLA4	2	2E+08	2E+08	1	278	19	323520	-0,52202	0,69917	protein_c	1493	CTLA4	0,57732	-0,782345735	1	2	0	NA	NA	NA	NA	No	NA	0,008623	rs114123510
152	UKBB_CAD	ENSG00000144452	ABCA12	2	2,2E+08	2,2E+08	-1	884	67	318446	-0,64664	0,74107	protein_c	26154	ABCA12	2,02E-07	-0,411531269	5	2	0	NA	NA	NA	NA	No	NA	NA	rs1250229
153	UKBB_CAD	ENSG00000138363	ATIC	2	2,2E+08	2,2E+08	1	503	36	319736	1	0,09949	protein_c	471	ATIC	2,49E-13	0,368896235	56	14,47	2	5,99E-12	0	BIOS_eQT	-	No	NA	0,000589	rs1250229
154	UKBB_CAD	ENSG00000115414	FN1	2	2,2E+08	2,2E+08	-1	630	49	319989	4	5,97E-05	protein_c	2335	FN1	0,06444	0,193079429	103	14,47	2	1,33E-16	0	BIOS_eQT	+	No	NA	1,85E-13	rs1250229
155	UKBB_CAD	ENSG00000164048	ZNF589	3	4,8E+07	4,8E+07	1	326	18	326862	3	0,00295	protein_c	51385	ZNF589	6,50E-07	-0,372175713	0	0	1	1,09E-27	0	eQTLGen	-	No	NA	0,000298	rs7623687
156	UKBB_CAD	ENSG00000229453	SPINK8	3	4,8E+07	4,8E+07	-1	304	16	329543	2	0,00897	protein_c	646424	SPINK8	0,00112	-0,205479315	0	0	1	1,57E-06	0,00467	eQTLGen	-	No	NA	0,000298	rs7623687
157	UKBB_CAD	ENSG00000164049	FBXW12	3	4,8E+07	4,8E+07	1	276	17	328671	1	0,06923	protein_c	285231	FBXW12	1,97E-07	0,783335407	3	11,36	0	NA	NA	NA	NA	No	NA	0,017893	rs7623687
158	UKBB_CAD	ENSG00000164050	PLXNB1	3	4,8E+07	4,8E+07	1	234	16	324454	0,44253	0,32905	protein_c	5364	PLXNB1	0,8502	NA	3	11,36	0	NA	NA	NA	NA	No	NA	0,017893	rs7623687
159	UKBB_CAD	ENSG00000164051	CCDC51	3	4,8E+07	4,8E+07	-1	173	14	323418	0,72672	0,2337	protein_c	79714	CCDC51	1,46E-06	0,085933137	4	11,36	0	NA	NA	NA	NA	No	NA	0,000307	rs7623687

160	UKBB_CAD	ENSG00000232112	TMA7	3	4,8E+07	4,8E+07	1	162	15	322875	0,74823	0,22716	protein_c	51372	TMA7	0,01001	0,321268329	4	11,36	3	6,76E-06	0,01928	eQTLGen_	+	No	NA	1,46E-06	rs7623687
161	UKBB_CAD	ENSG00000164053	ATRIP	3	4,8E+07	4,9E+07	1	190	21	323054	1	0,14254	protein_c	84126	ATRIP	0,00149	NA	4	11,36	5	8,28E-15	0	eQTLGen_	-	No	NA	1,46E-06	rs7623687
162	UKBB_CAD	ENSG00000213689	TREX1	3	4,9E+07	4,9E+07	1	172	22	322207	1	0,13294	protein_c	11277	TREX1	0,09334	-0,110327998	3	6	1	1,20E-26	0	eQTLGen_	-	No	NA	0,000276	rs7623687
163	UKBB_CAD	ENSG00000164054	SHISA5	3	4,9E+07	4,9E+07	-1	208	26	323043	1	0,09609	protein_c	51246	SHISA5	9,94E-08	-0,442030213	9	9	4	1,16E-13	0	eQTLGen_	+	No	NA	7,51E-07	rs7623687
164	UKBB_CAD	ENSG00000114268	PFKFB4	3	4,9E+07	4,9E+07	-1	218	31	312853	1	0,11177	protein_c	5210	PFKFB4	0,00032	-0,131320268	10	25,6	1	1,57E-05	0,04355	eQTLGen_	+	No	NA	1,40E-07	rs7623687
165	UKBB_CAD	ENSG00000145040	UCN2	3	4,9E+07	4,9E+07	-1	140	27	308076	0,85393	0,19657	protein_c	90226	UCN2	0,15724	0,006326735	9	25,6	0	NA	NA	NA	NA	No	NA	7,51E-07	rs7623687
166	UKBB_CAD	ENSG00000114270	COL7A1	3	4,9E+07	4,9E+07	-1	182	34	305198	0,36505	0,35754	protein_c	1294	COL7A1	2,11E-18	NA	9	25,6	7	1,19E-12	0	eQTLGen_	-	No	NA	7,51E-07	rs7623687
167	UKBB_CAD	ENSG00000010256	UQCRC1	3	4,9E+07	4,9E+07	-1	161	33	300740	-0,07125	0,5284	protein_c	7384	UQCRC1	0,13247	-0,703757501	4	25,6	4	1,13E-07	0,00039	eQTLGen_	-	No	NA	9,01E-08	rs7623687
168	UKBB_CAD	ENSG00000183396	TMEM89	3	4,9E+07	4,9E+07	-1	136	26	299457	0,47456	0,31755	protein_c	440955	TMEM89	0,71108	-0,32514209	5	25,6	0	NA	NA	NA	NA	No	NA	4,86E-08	rs7623687
169	UKBB_CAD	ENSG00000225697	SLC26A6	3	4,9E+07	4,9E+07	-1	153	22	303070	0,28131	0,38924	protein_c	65010	SLC26A6	3,31E-05	-0,384056203	5	25,6	8	1,20E-25	0	eQTLGen_	-	No	NA	4,86E-08	rs7623687
170	UKBB_CAD	ENSG00000008300	CELSR3	3	4,9E+07	4,9E+07	-1	194	21	309944	0,59359	0,27639	protein_c	1951	CELSR3	0,99966	-0,878907614	9	14,74	6	3,72E-10	0	eQTLGen_	+	No	NA	4,86E-08	rs7623687
171	UKBB_CAD	ENSG00000213672	NCKIPSD	3	4,9E+07	4,9E+07	-1	195	17	317642	0,88491	0,1881	protein_c	51517	NCKIPSD	0,00713	-0,551018211	11	14,74	8	4,94E-46	0	xQTLServe	+	No	NA	4,86E-08	rs7623687
172	UKBB_CAD	ENSG00000068745	IP6K2	3	4,9E+07	4,9E+07	-1	203	20	319987	1	0,13132	protein_c	51447	IP6K2	0,98946	-0,392798697	16	14,74	16	4,11E-84	0	BIOS_eQT	+	No	NA	4,86E-08	rs7623687
173	UKBB_CAD	ENSG00000114302	PRKAR2A	3	4,9E+07	4,9E+07	-1	212	20	322812	0,73538	0,23106	protein_c	5576	PRKAR2A	0,98325	-3	25	6	7	4,21E-11	0	eQTLGen_	+	No	NA	1,46E-06	rs7623687
174	UKBB_CAD	ENSG00000178537	SLC25A20	3	4,9E+07	4,9E+07	-1	137	13	320543	0,54303	0,29355	protein_c	788	SLC25A20	2,05E-07	-0,773546303	27	10	0	NA	NA	NA	NA	No	NA	0,000353	rs7623687
175	UKBB_CAD	ENSG00000221883	ARIH2OS	3	4,9E+07	4,9E+07	-1	111	12	319403	0,84528	0,19898	protein_c	646450	ARIH2OS	0,43761	-0,628345895	22	10	0	NA	NA	NA	NA	No	NA	1,04E-06	rs7623687
176	UKBB_CAD	ENSG00000177479	ARIH2	3	4,9E+07	4,9E+07	1	176	18	314434	1	0,10505	protein_c	10425	ARIH2	0,98741	-2	30	10	11	1,19E-34	0	BIOS_eQT	+	No	NA	1,36E-07	rs7623687
177	UKBB_CAD	ENSG00000178467	P4HTM	3	4,9E+07	4,9E+07	1	118	17	312767	2	0,03477	protein_c	54681	P4HTM	0,02327	NA	24	9	14	3,27E-76	0	BIOS_eQT	+	No	NA	1,36E-07	rs7623687
178	UKBB_CAD	ENSG00000178252	WDR6	3	4,9E+07	4,9E+07	1	103	15	310725	2	0,0317	protein_c	11180	WDR6	9,20E-07	-0,058949543	19	9	22	3.2717000	0	BIOS_eQT	-	No	NA	1,36E-07	rs7623687
179	UKBB_CAD	ENSG00000178149	DALRD3	3	4,9E+07	4,9E+07	-1	101	16	309801	2	0,04942	protein_c	55152	DALRD3	1,85E-05	0,072306759	20	8	4	7,48E-21	0	BIOS_eQT	+	No	NA	1,36E-07	rs7623687
180	UKBB_CAD	ENSG00000178057	NDUFAF3	3	4,9E+07	4,9E+07	1	100	16	309370	2	0,04492	protein_c	25915	NDUFAF3	4,86E-05	-2	19	8	7	7,24E-08	0,00029	eQTLGen_	-	No	NA	1,36E-07	rs7623687
181	UKBB_CAD	ENSG00000178035	IMPDH2	3	4,9E+07	4,9E+07	-1	106	17	311027	1	0,09326	protein_c	3615	IMPDH2	0,00135	-0,509342888	19	7	0	NA	NA	NA	NA	No	NA	1,36E-07	rs7623687
182	UKBB_CAD	ENSG00000198218	QRICH1	3	4,9E+07	4,9E+07	-1	164	24	309595	2	0,05964	protein_c	54870	QRICH1	0,99607	-0,27929443	26	23,1	13	2,07E-35	0	BIOS_eQT	+	Yes	GM12878	1,36E-07	rs7623687
183	UKBB_CAD	ENSG00000172053	QARS	3	4,9E+07	4,9E+07	-1	119	19	313931	1	0,06981	protein_c	5859	QARS	8,79E-08	-0,634755548	21	23,1	0	NA	NA	NA	NA	Yes	GM12878	6,50E-08	rs7623687
184	UKBB_CAD	ENSG00000172046	USP19	3	4,9E+07	4,9E+07	-1	131	18	314003	1	0,13615	protein_c	10869	USP19	0,99999	-0,429632772	16	23,1	2	1,76E-06	0,00226	GTEx_v7_	-	Yes	GM12878	6,50E-08	rs7623687
185	UKBB_CAD	ENSG00000172037	LAMB2	3	4,9E+07	4,9E+07	-1	144	19	315768	0,98874	0,1614	protein_c	3913	LAMB2	7,45E-11	-0,451081722	12	23,1	7	1,31E-14	0	eQTLGen_	+	No	NA	6,50E-08	rs7623687
186	UKBB_CAD	ENSG00000177352	CCDC71	3	4,9E+07	4,9E+07	-1	136	16	317513	0,81767	0,20677	protein_c	64925	CCDC71	0,01699	NA	13	23,1	7	1,45E-17	0	eQTLGen_	-	No	NA	6,50E-08	rs7623687
187	UKBB_CAD	ENSG00000185909	KLHDC8B	3	4,9E+07	4,9E+07	1	137	15	320463	0,75653	0,22467	protein_c	200942	KLHDC8B	0,01042	-0,077352225	13	23,1	17	#######	0	BIOS_eQT	-	No	NA	6,50E-08	rs7623687
188	UKBB_CAD	ENSG00000236980	C3orf84	3	4,9E+07	4,9E+07	-1	156	15	322232	0,40863	0,34141	protein_c	646498	C3orf84	0,00022	0,059049684	14	13,08	0	NA	NA	NA	NA	No	NA	6,50E-08	rs7623687
189	UKBB_CAD	ENSG00000173421	CCDC36	3	4,9E+07	4,9E+07	1	211	19	322393	0,09431	0,46243	protein_c	339834	CCDC36	0,50935	-0,452712817	16	15,69	6	5,91E-11	2,56E-24	GTEx_v7_	+	No	NA	1,40E-07	rs7623687
190	UKBB_CAD	ENSG00000225399	RP11-3B7	3	4,9E+07	4,9E+07	1	131	16	322922	0,14477	0,44245	protein_c	NA	NA	NA	NA	11	15,69	3	2,49E-05	1,32E-09	GTEx_v7_	+	No	NA	2,01E-05	rs7623687
191	UKBB_CAD	ENSG00000188315	C3orf62	3	4,9E+07	4,9E+07	-1	139	16	324079	0,01014	0,49596	protein_c	375341	C3orf62	0,00712	-2	11	15,69	7	1,08E-26	0	BIOS_eQT	+	Yes	IMR90:Me	1,40E-07	rs7623687
192	UKBB_CAD	ENSG00000114316	USP4	3	4,9E+07	4,9E+07	-1	226	22	325273	3	0,00251	protein_c	7375	USP4	6,20E-07	NA	62	19,43	3	4,77E-07	0,00156	eQTLGen_	-	No	NA	3,24E-08	rs7623687
193	UKBB_CAD	ENSG00000233276	GPX1	3	4,9E+07	4,9E+07	-1	167	16	327167	4	2,98E-05	protein_c	2876	GPX1	0,00693	-0,616399091	81	19,43	20	0	0	xQTLServe	+	No	NA	3,87E-09	rs7623687
194	UKBB_CAD	ENSG00000067560	RHOA	3	4,9E+07	4,9E+07	-1	255	14	325927	4	7,40E-06	protein_c	387	RHOA	0,8047	-0,430262742	135	19,43	4	9,07E-05	0,01145	GTEx_v7_	+	No	NA	3,72E-09	rs7623687
195	UKBB_CAD	ENSG00000145022	TCTA	3	4,9E+07	4,9E+07	1	187	16	324961	5	1,21E-06	protein_c	6988	TCTA	0,01326	-0,59594101	112	17,41	9	1,22E-07	0,00039	eQTLGen_	-	No	NA	3,72E-09	rs7623687
196	UKBB_CAD	ENSG00000145020	AMT	3	4,9E+07	4,9E+07	-1	189	16	325079	5	1,05E-06	protein_c	275	AMT	0,00057	-0,022637077	114	17,41	22	#######	0	BIOS_eQT	-	No	NA	3,72E-09	rs7623687
197	UKBB_CAD	ENSG00000145029	NICN1	3	4,9E+07	4,9E+07	-1	191	17	323863	5	1,01E-06	protein_c	84276	NICN1	0,25725	-1	112	17,41	21	#######	0	GTEx_v7_	-	No	NA	3,72E-09	rs7623687
198	UKBB_CAD	ENSG00000173402	DAG1	3	5E+07	5E+07	1	278	17	324319	5	5,92E-07	protein_c	1605	DAG1	0,94092	-0,838742424	148	19,64	18	4,46E-26	0	BIOS_eQT	-	No	NA	5,17E-09	rs7623687
199	UKBB_CAD	ENSG00000164061	BSN	3	5E+07	5E+07	1	351	23	318837	5	6,99E-07	protein_c	8927	BSN	1	-1	153	25,5	18	1,02E-18	0	BIOS_eQT	+	Yes	IMR90	7,50E-09	rs7623687
200	UKBB_CAD	ENSG00000164062	APEH	3	5E+07	5E+07	1	183	22	312591	4	3,56E-06	protein_c	327	APEH	0,01322	-0,27180506	76	25,5	20	#######	0	BIOS_eQT	-	No	NA	3,74E-08	rs7623687
201	UKBB_CAD	ENSG00000173531	MST1	3	5E+07	5E+07	-1	179	22	312423	4	5,45E-06	protein_c	4485	MST1	1,73E-10	0,158165154	79	25,5	11	1,08E-16	0	BIOS_eQT	-	No	NA	3,74E-08	rs7623687
202	UKBB_CAD	ENSG00000164068	RNF123	3	5E+07	5E+07	1	223	23	314551	4	1,96E-05	protein_c	63891	RNF123	0,96971	-0,46199081	108	25,5	14	3,27E-29	0	BIOS_eQT	+	No	NA	3,74E-08	rs7623687
203	UKBB_CAD	ENSG00000176020	AMIGO3	3	5E+07	5E+07	-1	185	19	318522	4	7,86E-05	protein_c	386724	AMIGO3	7,51E-06	NA	93	25,5	10	3,27E-29	0	BIOS_eQT	+	No	NA	3,74E-08	rs7623687
204	UKBB_CAD	ENSG00000173540	GMPPB	3	5E+07	5E+07	-1	185	19	318522	4	7,86E-05	protein_c	29925	GMPPB	8,25E-05	-0,65955402	93	25,5	10	3,27E-29	0	BIOS_eQT	+	No	NA	3,74E-08	rs7623687
205	UKBB_CAD	ENSG00000176095	IP6K1	3	5E+07	5E+07	-1	267	16	318038	4	0,00016	protein_c	9807	IP6K1	0,23518	-2	118	25,5	5	1,23E-05	0,00706	GTEx_v7_	+	No	NA	1,66E-08	rs7623687
206	UKBB_CAD	ENSG00000187492	CDHR4	3	5E+07	5E+07	-1	177	17	321463	3	0,00025	protein_c	389118	CDHR4	0,01347	0,043609676	78	20,7	8	1,60E-06	0,00077	GTEx_v7_	+	No	NA	1,60E-08	rs7623687
207	UKBB_CAD	ENSG00000185614	FAM212A	3	5E+07	5E+07	1	163	17	321566	4	0,00021	protein_c	389119	FAM212A	0,00059	NA	67	20,7	19	5,05E-52	0	BIOS_eQT	-	No	NA	1,60E-08	rs7623687
208	UKBB_CAD	ENSG00000182179	UBA7	3	5E+07	5E+07	-1	173	18	322008	4	8,30E-05	protein_c	7318	UBA7	2,68E-14	-0,315380801	69	16,53	20	3.2717000	0	BIOS_eQT	-	No	NA	1,60E-08	rs7623687
209	UKBB_CAD	ENSG00000183763	TRAIP	3	5E+07	5E+07	-1	191	22	320550	4	1,30E-05	protein_c	10293	TRAIP	0,1251	-0,132254904	57	12,03	0	NA	NA	NA	NA	Yes	IMR90:Me	1,60E-08	rs7623687
210	UKBB_CAD	ENSG00000164076	CAMKV	3	5E+07	5E+07	-1	162	20	320860	4	7,93E-06	protein_c	79012	CAMKV	0,99685	-0,638322632	45	12,03	0	NA	NA	NA	NA	Yes	IMR90:Me	1,60E-08	rs7623687
211	UKBB_CAD	ENSG00000164078	MST1R	3	5E+07	5E+07	-1	171	18	324440	4	5,16E-05	protein_c	4486	MST1R	1,69E-20	-0,377644955	32	15,69	0	NA	NA	NA	NA	No	NA	1,60E-08	rs7623687
212	UKBB_CAD	ENSG00000228008	CTD-2330	3	5E+07	5E+07	1	189	17	325517	4	0,00022	protein_c	NA	NA	NA	NA	25	15,69	7	1,90E-05	4,83E-05	GTEx_v7_	+	No	NA	6,04E-07	rs7623687
213	UKBB_CAD	ENSG00000164077	MON1A	3	5E+07	5E+07	-1	203	17	324486	3	0,00036	protein_c	84315	MON1A	0,00637	-0,629673162	24	15,69	17	9,05E-21	0	eQTLGen_	-	No	NA	6,04E-07	rs7623687
214	UKBB_CAD	ENSG00000004534	RBM6	3	5E+07	5E+07	1	469	21	326260	3,14	0,00084	protein_c	10180	RBM6	1	-0,685653183	24	15,69	21	3.2717000	0	xQTLServe	-	No	NA	8,89E-07	rs7623687
215	UKBB_CAD	ENSG00000003756	RBM5	3	5E+07	5E+07	1	230	26	325206	3	0,00086	protein_c	10181	RBM5	1	-0,605888711	8	8,24	0	NA	NA	NA	NA	Yes	IMR90:Me	0,002649	rs7623687
216	UKBB_CAD	ENSG00000001617	SEMA3F	3	5E+07	5E+07	1	234	33	320902	3	0,00471	protein_c	6405	SEMA3F	0,99992	NA	5	4	0	NA	NA	NA	NA	No	NA	0,002649	rs7623687
217	UKBB_CAD	ENSG00000114349	GNAT1	3	5E+07	5E+07	1	183	27	319019	2	0,0366	protein_c	2779	GNAT1	2,51E-06	-0,274733411	1	3	0	NA	NA	NA	NA	No	NA	NA	rs7623687
218	UKBB_CAD	ENSG00000114353	GNAI2	3	5E+07	5E+07	1	207	36	312146	2	0,02388	protein_c	2771	GNAI2	0,86116	-0,965006324	2	8	0	NA	NA	NA	NA	No	NA	NA	rs7623687
219	UKBB_CAD	ENSG00000179564	LSMEM2	3	5E+07	5E+07	1	161	33	306365	1	0,10715	protein_c	132228	LSMEM2	2,89E-05	-0,548022292	3	8	0	NA	NA	NA	NA	No	NA	NA	rs7623687
220	UKBB_CAD	ENSG00000214706	IFRD2	3	5E+07	5E+07	-1	162	33	304816	1,13	0,12923	protein_c	7866	IFRD2	5,53E-06	-0,616737733	3	8	2	2,27E-07	0,00074	eQTLGen_	-	No	NA	0,002019	rs7623687
221	UKBB_CAD	ENSG00000186792	HYAL3	3	5E+07	5E+07	-1	157	32	304378	0,9992	0,15885	protein_c	8372	HYAL3	1,52E-05	-0,353149956	3	8	18	#######	0	GTEx_v7_	+	No	NA	8,89E-07	rs7623687
222	UKBB_CAD	ENSG00000243477	NAT6	3	5E+07	5E+07	-1	153	31	303849	1	0,15519	protein_c	24142	NAT6	0,40295	-0,113189262	3	8	7	9,39E-13	0	eQTLGen_	-	No	NA	8,89E-07	rs7623687
223	UKBB_CAD	ENSG00000114378	HYAL1	3	5E+07	5E+07	-1	161	33	302924	1	0,12596	protein_c	3373	HYAL1	0,00554	0,476164437	4	8	0	NA	NA	NA	NA	No	NA	NA	rs7623687
224	UKBB_CAD	ENSG00000068001	HYAL2	3	5E+07	5E+07	-1	147	30	298317	1	0,14492	protein_c	8692	HYAL2	0,02063	-1	4	8	0	NA	NA	NA	NA	No	NA	NA	rs7623687
225	UKBB_CAD	ENSG00000114383	TUSC2	3	5E+07	5E+07	-1	148	30	298580	0,99705	0,15937	protein_c	11334	TUSC2	0,70303	-0,593776389	4	8	0	NA	NA	NA	NA	No	NA	NA	rs7623687
226	UKBB_CAD	ENSG00000068028	RASSF1	3	5E+07	5E+07	-1	158	33	299344	1	0,12762	protein_c	11186	RASSF1	4,86E-08	-0,411946648	3	8	0	NA	NA	NA	NA	No	NA	NA	rs7623687
227	UKBB_CAD	ENSG00000004838	ZMYND10	3	5E+07	5E+07	-1	159	34	302780	1	0,11085	protein_c	51364	ZMYND10	3,60E-07	-0,824530982	3	8	0	NA	NA	NA	NA	No	NA	NA	rs7623687
228	UKBB_CAD	ENSG00000114388	NPRL2	3	5E+07	5E+07	-1	149	31	306222	1	0,11538	protein_c	10641	NPRL2	0,34517	-0,107063929	3	8	0	NA	NA	NA	NA	No	NA	NA	rs7623687
229	UKBB_CAD	ENSG00000114395	CYB561D2	3	5E+07	5E+07	1	163	34	307692	1	0,10672	protein_c	11068	CYB561D2	0,01471	-0,298456388	3	8	0	NA	NA	NA	NA	No	NA	NA	rs7623687
230	UKBB_CAD	ENSG00000272104	XXcos-LU	3	5E+07	5E+07	1	183	35	308647	1	0,13156	protein_c	NA	NA	NA	NA	3	8	0	NA	NA	NA	NA	No	NA	NA	rs7623687
231	UKBB_CAD	ENSG00000126062	TMEM115	3	5E+07	5E+07	-1	155	32	307229	1	0,12604	protein_c	11070	TMEM115	0,8769	-0,780371829	2	8	0	NA	NA	NA	NA	No	NA	NA	rs7623687
232	UKBB_CAD	ENSG00000007402	CACNA2D	3	5E+07	5,1E+07	-1	439	32	315698	0,92565	0,17731	protein_c	9254	CACNA2D	0,99969	NA	1	7	0	NA	NA	NA	NA	No	NA	NA	rs7623687
233	UKBB_CAD	ENSG00000114735	HEMK1	3	5,1E+07	5,1E+07	1	202	20	318609	2	0,02676	protein_c	51409	HEMK1	8,23E-05	-3	0	0	8	1,75E-07	0,00058	eQTLGen_	-	No	NA	4,76E-06	rs7623687
234	UKBB_CAD	ENSG00000114738	MAPKAPK	3	5,1E+07	5,1E+07	1	262	17	320670	2	0,02793	protein_c	7867	MAPKAPK	2,71E-06	0,077268555	0	0	8	7,71E-30	0	eQTLGen_	-	No	NA	4,76E-06	rs7623687
235	UKBB_CAD	ENSG00000088538	DOCK3	3	5,1E+07	5,1E+07	1	1600	21	325337	3	0,00167	protein_c	1795	DOCK3	1	-0,836764533	0	0	2	4,19E-06	3,07E-05	GTEx_v7_	+	No	NA	5,96E-06	rs7623687
236	UKBB_CAD	ENSG00000160145	KALRN	3	1,2E+08	1,2E+08	1	2667	117	329867	4	0,00016	protein_c	8997	KALRN	1	-0,64291779	50	20,7	1	1,94E-07	0,00064	eQTLGen_	+	No	NA	1,00E-08	rs62265630
237	UKBB_CAD	ENSG00000114491	UMPS	3	1,2E+08	1,2E+08	1	548	33	330947	3	0,00188	protein_c	7372	UMPS	0,00011	1	51	20,7	2	4,33E-39	0	BIOS_eQT	-	No	NA	1,00E-08	rs62265630
238	UKBB_CAD	ENSG00000082781	ITGB5	3	1,2E+08	1,2E+08	-1	989	45	328756	2	0,01193	protein_c	3693	ITGB5	0,00048	1	96	20,7	1	8,46E-25	0	eQTLGen_	-	No	NA	1,00E-08	rs62265630
239	UKBB_CAD	ENSG00000173702	MUC13	3	1,2E+08	1,2E+08	-1	535	47	327972	-0,41052	0,65929	protein_c	56667	MUC13	0,02513	NA	22	6,34	0	NA	NA	NA	NA	No	NA	0,026901	rs62265630
240	UKBB_CAD	ENSG00000173706	HEG1	3	1,2E+08	1,2E+08	-1	661	79	328306	-0,90814	0,8181	protein_c	57493	HEG1	6,99E-06	0,180704267	5	6,34	0	NA	NA	NA	NA	No	NA	0,026901	rs62265630
241	UKBB_CAD	ENSG00000158163	DZIP1L	3	1,4E+08	1,4E+08	-1	392	24	325519	1	0,12306	protein_c	199221	DZIP1L	6,93E-20	-0,028954451	17	11,85	0	NA	NA	NA	NA	No	NA	0,002671	rs139016349

242	UKBB_CAD	ENSG00000118017	A4GNT	3	1,4E+08	1,4E+08	-1	259	12	324943	1	0,08798	protein_c	51146	A4GNT	0,59841	0,526700882	24	11,85	0	NA	NA	NA	NA	No	NA	0,002671	rs139016349
243	UKBB_CAD	ENSG00000138231	DBR1	3	1,4E+08	1,4E+08	-1	205	15	322611	2	0,03304	protein_c	51163	DBR1	8,71E-05	0,19192925	27	11,85	0	NA	NA	NA	NA	No	NA	4,86E-06	rs139016349
244	UKBB_CAD	ENSG00000114098	ARMC8	3	1,4E+08	1,4E+08	1	289	34	321777	6	6,05E-09	protein_c	25852	ARMC8	0,99988	-0,678949088	77	18,85	0	NA	NA	NA	NA	No	NA	5,37E-10	rs139016349
245	UKBB_CAD	ENSG00000181322	NME9	3	1,4E+08	1,4E+08	-1	245	30	322208	6	1,90E-09	protein_c	347736	NME9	2,63E-05	-0,73564367	97	18,85	0	NA	NA	NA	NA	Yes	IMR90:Tro	2,41E-11	rs139016349
246	UKBB_CAD	ENSG00000158186	MRAS	3	1,4E+08	1,4E+08	1	306	26	326161	7	2,50E-11	protein_c	22808	MRAS	0,85995	-0,413633907	161	18,85	18	4,98E-46	0	BIOS_eQT	+	Yes	IMR90:Tro	9,20E-12	rs139016349
247	UKBB_CAD	ENSG00000158220	ESYT3	3	1,4E+08	1,4E+08	1	294	29	326426	6	2,74E-09	protein_c	83850	ESYT3	2,43E-11	-0,947164338	148	18,65	4	1,10E-06	2,20E-05	GTEx_v7_	+	Yes	IMR90:Me	2,90E-09	rs139016349
248	UKBB_CAD	ENSG00000114107	CEP70	3	1,4E+08	1,4E+08	-1	422	20	325304	4	8,51E-05	protein_c	80321	CEP70	5,95E-11	-0,571935395	225	18,65	9	5,16E-44	0	eQTLGen_	-	Yes	GM12878	5,27E-09	rs139016349
249	UKBB_CAD	ENSG00000158234	FAIM	3	1,4E+08	1,4E+08	1	228	19	323858	3	0,00039	protein_c	55179	FAIM	0,06812	-0,930130513	135	17,13	17	0	0	xQTLServe	-	Yes	IMR90:Me	4,72E-09	rs139016349
250	UKBB_CAD	ENSG00000051382	PIK3CB	3	1,4E+08	1,4E+08	-1	480	18	323308	2	0,00674	protein_c	5291	PIK3CB	0,99998	-0,399991609	280	17,03	2	2,78E-06	0,0081	eQTLGen_	+	Yes	IMR90	0,000248	rs139016349
251	UKBB_CAD	ENSG00000183770	FOXL2	3	1,4E+08	1,4E+08	-1	262	27	326990	-0,27205	0,60721	protein_c	668	FOXL2	NA	-0,760660692	7	9	0	NA	NA	NA	NA	Yes	GM12878	5,27E-09	rs139016349
252	UKBB_CAD	ENSG00000206262	C3orf72	3	1,4E+08	1,4E+08	1	264	28	326652	-0,40227	0,65626	protein_c	401089	C3orf72	0,09344	0,669830103	5	9	0	NA	NA	NA	NA	Yes	GM12878	5,27E-09	rs139016349
253	UKBB_CAD	ENSG00000206260	PRR23A	3	1,4E+08	1,4E+08	-1	220	23	326008	-0,69804	0,75742	protein_c	729627	PRR23A	0,01808	-0,400504951	2	10,67	0	NA	NA	NA	NA	No	NA	0,004976	rs139016349
254	UKBB_CAD	ENSG00000175110	MRPS22	3	1,4E+08	1,4E+08	1	1129	66	326955	-1	0,89195	protein_c	56945	MRPS22	6,39E-05	NA	5	10,67	0	NA	NA	NA	NA	No	NA	0,004976	rs139016349
255	UKBB_CAD	ENSG00000184814	PRR23B	3	1,4E+08	1,4E+08	-1	209	19	326444	-0,37753	0,64711	protein_c	389151	PRR23B	0,00253	-1	2	10,67	0	NA	NA	NA	NA	No	NA	0,004976	rs139016349
256	UKBB_CAD	ENSG00000233701	PRR23C	3	1,4E+08	1,4E+08	-1	209	15	325600	-0,38461	0,64974	protein_c	389152	PRR23C	0,00064	0,459235323	3	10,67	0	NA	NA	NA	NA	No	NA	0,006149	rs139016349
257	UKBB_CAD	ENSG00000181467	RAP2B	3	1,5E+08	1,5E+08	1	267	34	326101	-0,45432	0,6752	protein_c	5912	RAP2B	0,27548	-0,801645112	0	0	0	NA	NA	NA	NA	Yes	Mesenchy	0,034674	rs12493885
258	UKBB_CAD	ENSG00000114790	ARHGEF26	3	1,5E+08	1,5E+08	1	671	27	325016	4	1,09E-05	protein_c	26084	ARHGEF26	5,99E-06	-0,076339008	312	24,1	10	1,02E-18	5,14E-14	BIOS_eQT	+	No	NA	3,29E-08	rs12493885
259	UKBB_CAD	ENSG00000174953	DHX36	3	1,5E+08	1,5E+08	-1	341	24	327747	4	0,00019	protein_c	170506	DHX36	0,99056	-0,635375291	129	24,1	14	#######	0	xQTLServe	-	No	NA	4,03E-06	rs12493885
260	UKBB_CAD	ENSG00000174948	GPR149	3	1,5E+08	1,5E+08	-1	500	41	328043	3	0,00101	protein_c	344758	GPR149	2,65E-05	0,039091	128	24,1	0	NA	NA	NA	NA	No	NA	1,07E-05	rs12493885
261	UKBB_CAD	ENSG00000196549	MME	3	1,5E+08	1,5E+08	1	651	70	318704	-0,90643	0,81765	protein_c	4311	MME	2,19E-08	0,206162936	0	0	2	1,01E-12	0	eQTLGen_	-	No	NA	0,00408	rs12493885
262	UKBB_CAD	ENSG00000138759	FRAS1	4	7,9E+07	7,9E+07	1	2191	67	329782	-0,46094	0,67758	protein_c	80144	FRAS1	2,29E-23	-0,327183247	0	0	0	NA	NA	NA	NA	Yes	Mesenchy	0,019775	rs10857147
263	UKBB_CAD	ENSG00000163291	PAQR3	4	8E+07	8E+07	-1	391	34	323336	-1	0,87016	protein_c	152559	PAQR3	0,02039	2	0	0	0	NA	NA	NA	NA	Yes	IMR90:Me	0,019775	rs10857147
264	UKBB_CAD	ENSG00000152784	PRDM8	4	8,1E+07	8,1E+07	1	480	34	330004	3	0,00497	protein_c	56978	PRDM8	NA	0,457538182	52	20,5	2	1,40E-48	0	BIOS_eQT	+	No	NA	4,78E-08	rs10857147
265	UKBB_CAD	ENSG00000138675	FGF5	4	8,1E+07	8,1E+07	1	571	45	323492	3	0,00044	protein_c	2250	FGF5	4,32E-05	0,709603293	118	20,5	0	NA	NA	NA	NA	No	NA	8,96E-09	rs10857147
266	UKBB_CAD	ENSG00000197826	C4orf22	4	8,1E+07	8,2E+07	1	2110	47	322185	-0,86313	0,80597	protein_c	255119	C4orf22	1,12E-05	-0,223573604	3	8	0	NA	NA	NA	NA	Yes	IMR90:Me	0,001866	rs10857147
267	UKBB_CAD	ENSG00000152785	BMP3	4	8,2E+07	8,2E+07	1	305	22	326082	-0,79877	0,78779	protein_c	651	BMP3	0,11024	1	0	0	0	NA	NA	NA	NA	Yes	Mesenchy	0,019775	rs10857147
268	UKBB_CAD	ENSG00000151615	POU4F2	4	1,5E+08	1,5E+08	1	287	21	320547	-0,22937	0,59071	protein_c	5458	POU4F2	0,18013	-0,695034981	2	5	0	NA	NA	NA	NA	No	NA	0,021464	rs4593108
269	UKBB_CAD	ENSG00000137473	TTC29	4	1,5E+08	1,5E+08	-1	867	37	327566	0,07554	0,46989	protein_c	83894	TTC29	1,57E-06	-0,637384565	3	5	0	NA	NA	NA	NA	No	NA	0,016272	rs6841581;rs4593108
270	UKBB_CAD	ENSG00000151617	EDNRA	4	1,5E+08	1,5E+08	1	462	43	329698	5	4,98E-08	protein_c	1909	EDNRA	0,98602	-0,145871514	173	19,67	0	NA	NA	NA	NA	Yes	IMR90:Me	4,57E-10	rs6841581;rs4593108
271	UKBB_CAD	ENSG00000164168	TMEM184	4	1,5E+08	1,5E+08	1	354	33	320428	3	0,0011	protein_c	55751	TMEM184	0,0176	-0,573835645	5	8	0	NA	NA	NA	NA	Yes	IMR90:Me	7,92E-08	rs6841581;rs4593108
272	UKBB_CAD	ENSG00000164169	PRMT10	4	1,5E+08	1,5E+08	-1	304	25	318047	2	0,00629	protein_c	90826	PRMT10	7,84E-06	-0,658528725	3	8	0	NA	NA	NA	NA	No	NA	0,046444	rs4593108
273	UKBB_CAD	ENSG00000071205	ARHGAP1	4	1,5E+08	1,5E+08	1	971	75	323758	-1	0,87082	protein_c	79658	ARHGAP1	1,22E-10	NA	4	6	0	NA	NA	NA	NA	Yes	IMR90	7,92E-08	rs6841581;rs4593108
274	UKBB_CAD	ENSG00000164116	GUCY1A3	4	1,6E+08	1,6E+08	1	642	60	329030	5	1,52E-07	protein_c	2982	GUCY1A3	1,41E-06	2	121	13,42	3	2,18E-31	0	BIOS_eQT	-	No	NA	1,22E-09	rs2306556
275	UKBB_CAD	ENSG00000061918	GUCY1B3	4	1,6E+08	1,6E+08	1	537	62	327216	5	1,91E-07	protein_c	2983	GUCY1B3	0,00092	-0,241661798	115	13,42	0	NA	NA	NA	NA	No	NA	1,22E-09	rs2306556
276	UKBB_CAD	ENSG00000256394	ASIC5	4	1,6E+08	1,6E+08	-1	536	40	322652	2	0,06665	protein_c	51802	ASIC5	2,26E-07	0,440633629	21	9	0	NA	NA	NA	NA	No	NA	0,000454	rs2306556
277	UKBB_CAD	ENSG00000151790	TDO2	4	1,6E+08	1,6E+08	1	727	46	325746	0,19894	0,42115	protein_c	6999	TDO2	1,52E-08	-0,187110818	4	6,73	0	NA	NA	NA	NA	No	NA	0,000891	rs2306556
278	UKBB_CAD	ENSG00000078401	EDN1	6	1,2E+07	1,2E+07	1	304	41	328463	0,35294	0,36207	protein_c	1906	EDN1	0,08102	-0,888329633	0	0	0	NA	NA	NA	NA	Yes	hESC	7,40E-05	rs9349379
279	UKBB_CAD	ENSG00000112137	PHACTR1	6	1,3E+07	1,3E+07	1	1952	93	328345	9	1,23E-18	protein_c	221692	PHACTR1	0,66412	NA	419	20,6	8	1,58E-19	0	BIOS_eQT	+	No	NA	9,95E-36	rs9349379
280	UKBB_CAD	ENSG00000145979	TBC1D7	6	1,3E+07	1,3E+07	-1	644	35	332904	0,79321	0,21383	protein_c	51256	TBC1D7	8,30E-06	0,944871699	0	0	7	1,84E-13	0	eQTLGen_	+	No	NA	1,21E-08	rs9349379
281	UKBB_CAD	ENSG00000112242	E2F3	6	2E+07	2E+07	1	523	68	323259	3	0,00466	protein_c	1871	E2F3	0,84246	-2	0	0	0	NA	NA	NA	NA	Yes	Mesenchy	1,54E-07	rs6909752
282	UKBB_CAD	ENSG00000145996	CDKAL1	6	2,1E+07	2,1E+07	1	2762	109	327841	0,66619	0,25265	protein_c	54901	CDKAL1	0,57765	0,904338397	0	0	0	NA	NA	NA	NA	Yes	IMR90:Me	1,54E-07	rs6909752
283	UKBB_CAD	ENSG00000112273	HDGFL1	6	2,3E+07	2,3E+07	1	503	32	325187	6	1,74E-08	protein_c	154150	HDGFL1	0,31334	1	188	22	0	NA	NA	NA	NA	No	NA	5,59E-09	rs6909752
284	UKBB_CAD	ENSG00000152954	NRSN1	6	2,4E+07	2,4E+07	1	551	57	320946	-0,96823	0,83354	protein_c	140767	NRSN1	0,00431	1	0	0	0	NA	NA	NA	NA	Yes	IMR90	1,54E-07	rs6909752
285	UKBB_CAD	ENSG00000079950	STX7	6	1,3E+08	1,3E+08	-1	608	64	328564	-0,77879	0,78195	protein_c	8417	STX7	0,00426	2	0	0	0	NA	NA	NA	NA	Yes	Mesenchy	0,006381	rs12202017
286	UKBB_CAD	ENSG00000112303	VNN2	6	1,3E+08	1,3E+08	-1	585	51	328530	-0,06806	0,52713	protein_c	8875	VNN2	1,02E-08	1	0	0	0	NA	NA	NA	NA	Yes	IMR90:Me	0,006381	rs12202017
287	UKBB_CAD	ENSG00000146409	SLC18B1	6	1,3E+08	1,3E+08	-1	624	37	327577	-0,41811	0,66207	protein_c	116843	SLC18B1	3,95E-10	0,973266142	0	0	0	NA	NA	NA	NA	Yes	IMR90:Me	0,006381	rs12202017
288	UKBB_CAD	ENSG00000112306	RPS12	6	1,3E+08	1,3E+08	1	483	26	326433	-0,66101	0,7457	protein_c	6206	RPS12	0,82744	0,479144793	0	0	0	NA	NA	NA	NA	Yes	IMR90:Me	0,006381	rs12202017
289	UKBB_CAD	ENSG00000118526	TCF21	6	1,3E+08	1,3E+08	1	275	26	325601	6	1,63E-10	protein_c	6943	TCF21	0,80537	0,400264301	120	12,85	0	NA	NA	NA	NA	Yes	IMR90:Me	6,02E-14	rs12202017
290	UKBB_CAD	ENSG00000028839	TBPL1	6	1,3E+08	1,3E+08	1	340	36	324420	2	0,05717	protein_c	9519	TBPL1	0,87962	2	22	12,85	0	NA	NA	NA	NA	Yes	IMR90:Me	2,45E-10	rs12202017
291	UKBB_CAD	ENSG00000146411	SLC2A12	6	1,3E+08	1,3E+08	-1	384	53	324310	2	0,02894	protein_c	154091	SLC2A12	0,01941	0,77929054	1	4	0	NA	NA	NA	NA	Yes	IMR90:Me	0,006381	rs12202017
292	UKBB_CAD	ENSG00000118514	ALDH8A1	6	1,4E+08	1,4E+08	-1	324	23	326983	0,44722	0,32736	protein_c	64577	ALDH8A1	9,46E-06	0,269937741	0	0	0	NA	NA	NA	NA	Yes	IMR90:Me	0,006381	rs12202017
293	UKBB_CAD	ENSG00000112096	SOD2	6	1,6E+08	1,6E+08	-1	716	32	326605	0,4371	0,33102	protein_c	6648	SOD2	0,23715	2	1	0,201	1	7,82E-07	0,00252	eQTLGen_	-	No	NA	6,31E-10	rs186696265;rs624249
294	UKBB_CAD	ENSG00000146457	WTAP	6	1,6E+08	1,6E+08	1	497	27	327172	-0,19465	0,57717	protein_c	9589	WTAP	0,99558	2	1	0,201	0	NA	NA	NA	NA	No	NA	0,027481	rs186696265
295	UKBB_CAD	ENSG00000120437	ACAT2	6	1,6E+08	1,6E+08	1	433	27	327449	0,06997	0,47211	protein_c	39	ACAT2	1,26E-05	0,731905322	2	7	0	NA	NA	NA	NA	No	NA	1,67E-07	rs186696265
296	UKBB_CAD	ENSG00000120438	TCP1	6	1,6E+08	1,6E+08	-1	421	28	327548	0,65658	0,25573	protein_c	6950	TCP1	0,98909	0,157899873	2	7	0	NA	NA	NA	NA	No	NA	1,67E-07	rs186696265
297	UKBB_CAD	ENSG00000112110	MRPL18	6	1,6E+08	1,6E+08	1	421	36	327440	1	0,07263	protein_c	29074	MRPL18	0,00079	2	3	7	1	1,23E-05	0,03446	eQTLGen_	-	No	NA	6,31E-10	rs186696265;rs624249
298	UKBB_CAD	ENSG00000146453	PNLDC1	6	1,6E+08	1,6E+08	1	480	41	328352	2	0,02607	protein_c	154197	PNLDC1	2,54E-08	0,054243557	3	7	0	NA	NA	NA	NA	No	NA	2,02E-09	rs186696265
299	UKBB_CAD	ENSG00000130368	MAS1	6	1,6E+08	1,6E+08	1	293	21	328158	2	0,05292	protein_c	4142	MAS1	0,00087	-0,186942489	2	2	0	NA	NA	NA	NA	No	NA	2,29E-13	rs186696265
300	UKBB_CAD	ENSG00000197081	IGF2R	6	1,6E+08	1,6E+08	1	735	72	325100	6	3,46E-10	protein_c	3482	IGF2R	1	-0,312060143	29	13,65	0	NA	NA	NA	NA	No	NA	4,54E-15	rs186696265;rs10455872
301	UKBB_CAD	ENSG00000175003	SLC22A1	6	1,6E+08	1,6E+08	1	435	62	325918	7	2,46E-12	protein_c	6580	SLC22A1	1,24E-13	-0,158083671	38	13,65	0	NA	NA	NA	NA	No	NA	1,59E-10	rs186696265;rs10455872;rs624249;rs9365196
302	UKBB_CAD	ENSG00000112499	SLC22A2	6	1,6E+08	1,6E+08	-1	973	54	331312	7	2,97E-12	protein_c	6582	SLC22A2	1,42E-09	0,905296138	489	19,11	0	NA	NA	NA	NA	No	NA	2,54E-23	rs186696265;rs10455872;rs624249;rs9365196;rs9457995
303	UKBB_CAD	ENSG00000146477	SLC22A3	6	1,6E+08	1,6E+08	1	689	32	327095	7	2,69E-12	protein_c	6581	SLC22A3	3,32E-05	0,310629054	367	13,04	4	3,44E-15	0	BIOS_eQT	+	No	NA	1,63E-32	rs10455872;rs9365196;rs624249;rs9457995;rs186696265
304	UKBB_CAD	ENSG00000198670	LPA	6	1,6E+08	1,6E+08	-1	552	42	322780	9	4,20E-20	protein_c	4018	LPA	2,44E-32	0,042144435	327	26,3	1	4,88E-07	0,00037	BIOS_eQT	-	No	NA	1,71E-49	rs10455872;rs9365196;rs9457995;rs624249;rs186696265
305	UKBB_CAD	ENSG00000122194	PLG	6	1,6E+08	1,6E+08	1	566	31	325242	7	1,91E-11	protein_c	5340	PLG	0,00244	0,479258267	247	17,13	0	NA	NA	NA	NA	No	NA	1,08E-43	rs9457995;rs9365196;rs10455872;rs624249;rs186696265
306	UKBB_CAD	ENSG00000085511	MAP3K4	6	1,6E+08	1,6E+08	1	837	46	323334	3	0,00583	protein_c	4216	MAP3K4	0,99999	NA	19	6,45	0	NA	NA	NA	NA	No	NA	2,64E-18	rs186696265;rs10455872
307	UKBB_CAD	ENSG00000026652	AGPAT4	6	1,6E+08	1,6E+08	-1	939	90	326147	3	0,0024	protein_c	56895	AGPAT4	0,6745	0,748253089	35	8	0	NA	NA	NA	NA	No	NA	6,87E-09	rs186696265;rs10455872
308	UKBB_CAD	ENSG00000185345	PARK2	6	1,6E+08	1,6E+08	-1	6058	278	329211	-0,46471	0,67893	protein_c	5071	PARK2	0,02181	0,549835313	3	7	0	NA	NA	NA	NA	No	NA	0,040028	rs186696265
309	UKBB_CAD	ENSG00000106524	ANKMY2	7	1,7E+07	1,7E+07	-1	569	74	327495	-1	0,87548	protein_c	57037	ANKMY2	0,00022	0,257890143	0	0	0	NA	NA	NA	NA	Yes	IMR90	0,024021	rs2107595
310	UKBB_CAD	ENSG00000136261	BZW2	7	1,7E+07	1,7E+07	1	753	86	327962	-0,16316	0,5648	protein_c	28969	BZW2	0,99582	-0,094537216	0	0	0	NA	NA	NA	NA	Yes	IMR90	0,024021	rs2107595
311	UKBB_CAD	ENSG00000048052	HDAC9	7	1,8E+07	1,9E+07	1	2995	204	326060	2	0,02763	protein_c	9734	HDAC9	0,99838	-0,115886844	75	19,01	0	NA	NA	NA	NA	No	NA	3,41E-13	rs2107595
312	UKBB_CAD	ENSG00000122691	TWIST1	7	1,9E+07	1,9E+07	-1	625	63	314136	5	3,48E-07	protein_c	7291	TWIST1	0,18203	NA	79	19,01	1	9,44E-06	0,00913	GTEx_v7_	+	No	NA	3,41E-13	rs2107595
313	UKBB_CAD	ENSG00000146618	FERD3L	7	1,9E+07	1,9E+07	-1	280	33	327199	-1	0,87709	protein_c	222894	FERD3L	0,25816	-0,104378399	6	14,77	0	NA	NA	NA	NA	No	NA	0,019838	rs2107595
314	UKBB_CAD	ENSG00000164651	SP8	7	2,1E+07	2,1E+07	-1	244	28	327637	-0,42824	0,66576	protein_c	221833	SP8	NA	NA	0	0	0	NA	NA	NA	NA	Yes	hESC	0,024021	rs2107595
315	UKBB_CAD	ENSG00000164649	CDCA7L	7	2,2E+07	2,2E+07	-1	683	75	327520	-0,8047	0,7895	protein_c	55536	CDCA7L	0,12066	-0,449039521	0	0	0	NA	NA	NA	NA	Yes	Trophobla	0,024021	rs2107595
316	UKBB_CAD	ENSG00000128604	IRF5	7	1,3E+08	1,3E+08	1	322	43	320254	-0,34117	0,63351	protein_c	3663	IRF5	0,28848	NA	0	0	0	NA	NA	NA	NA	Yes	Mesenchy	6,26E-13	rs11556924
317	UKBB_CAD	ENSG00000158457	TSPAN33	7	1,3E+08	1,3E+08	1	439	53	326362	-2	0,93819	protein_c	340348	TSPAN33	0,0001	0,982621066	0	0	1	7,20E-06	0,02052	eQTLGen_	-	No	NA	6,26E-13	rs11556924
318	UKBB_CAD	ENSG00000128602	SMO	7	1,3E+08	1,3E+08	1	355	38	324125	-0,97417	0,83501	protein_c	6608	SMO	0,06371	NA	0	0	0	NA	NA	NA	NA	Yes	IMR90:Me	6,26E-13	rs11556924
319	UKBB_CAD	ENSG00000128578	STRIP2	7	1,3E+08	1,3E+08	1	493	52	323489	0,2605	0,39724	protein_c	57464	STRIP2	7,35E-10	-0,301291227	0	0	0	NA	NA	NA	NA	Yes	GM12878	6,26E-13	rs11556924
320	UKBB_CAD	ENSG00000240204	SMKR1	7	1,3E+08	1,3E+08	1	443	58	323464	0,74415	0,22839	protein_c	1E+08	SMKR1	NA	NA	0	0	0	NA	NA	NA	NA	Yes	IMR90:Me	6,26E-13	rs11556924
321	UKBB_CAD	ENSG00000106459	NRF1	7	1,3E+08	1,3E+08	1	786	43	328436	1	0,15779	protein_c	4899	NRF1	0,99697	-0,678127541	0	0	0	NA	NA	NA	NA	Yes	Aorta:GM	6,26E-13	rs11556924
322	UKBB_CAD	ENSG00000186591	UBE2H	7	1,3E+08	1,3E+08	-1	875	33	327832	2	0,01037	protein_c	7328	UBE2H	0,97097	-2	29	11,89	0	NA	NA	NA	NA	Yes	GM12878	6,26E-13	rs11556924
323	UKBB_CAD	ENSG00000091732	ZC3HC1	7	1,3E+08	1,3E+08	-1	455	36	319818	4	9,80E-05	protein_c	51530	ZC3HC1	0,00051	-0,120113838	133	34	0	NA	NA	NA	NA	Yes	IMR90	6,26E-13	rs11556924

324	UKBB_CAD	ENSG00000128607	KLHDC10	7	1,3E+08	1,3E+08	1	476	47	321178	3	0,0005	protein_c	23008	KLHDC10	0,99668	-0,826631253	141	34	3	3,02E-74	0	BIOS_eQT	+	Yes	IMR90	6,26E-13	rs11556924
325	UKBB_CAD	ENSG00000146842	TMEM209	7	1,3E+08	1,3E+08	-1	402	40	325324	2	0,02836	protein_c	84928	TMEM209	0,0052	-2	42	12,56	2	1,06E-13	0	eQTLGen_	+	No	NA	8,43E-05	rs11556924
326	UKBB_CAD	ENSG00000165120	SSMEM1	7	1,3E+08	1,3E+08	1	276	24	326803	0,79785	0,21248	protein_c	136263	SSMEM1	6,43E-13	0,269904475	4	11,67	0	NA	NA	NA	NA	No	NA	0,044182	rs11556924
327	UKBB_CAD	ENSG00000158525	CPA5	7	1,3E+08	1,3E+08	1	431	41	323778	2	0,03248	protein_c	93979	CPA5	0,04749	1	0	0	3	9,69E-08	0,00034	eQTLGen_	+	No	NA	6,26E-13	rs11556924
328	UKBB_CAD	ENSG00000106477	CEP41	7	1,3E+08	1,3E+08	-1	427	50	322906	0,62896	0,26469	protein_c	95681	CEP41	0,00013	-0,511705656	0	0	0	NA	NA	NA	NA	Yes	GM12878	6,26E-13	rs11556924
329	UKBB_CAD	ENSG00000106484	MEST	7	1,3E+08	1,3E+08	1	171	26	322759	-1	0,8536	protein_c	4232	MEST	0,99753	-0,346601943	0	0	0	NA	NA	NA	NA	Yes	IMR90:Me	6,26E-13	rs11556924
330	UKBB_CAD	ENSG00000158623	COPG2	7	1,3E+08	1,3E+08	-1	475	28	324800	0,58902	0,27792	protein_c	26958	COPG2	0,05604	NA	0	0	0	NA	NA	NA	NA	Yes	GM12878	6,26E-13	rs11556924
331	UKBB_CAD	ENSG00000174595	KLF14	7	1,3E+08	1,3E+08	-1	313	34	325960	3	0,00157	protein_c	136259	KLF14	0,21811	NA	0	0	0	NA	NA	NA	NA	Yes	GM12878	6,26E-13	rs11556924
332	UKBB_CAD	ENSG00000213203	GIMAP1	7	1,5E+08	1,5E+08	1	371	31	329428	2	0,03674	protein_c	170575	GIMAP1	0,83932	0,633162789	1	8,8	0	NA	NA	NA	NA	No	NA	0,041756	rs3918226
333	UKBB_CAD	ENSG00000196329	GIMAP5	7	1,5E+08	1,5E+08	1	465	42	329661	2	0,05955	protein_c	55340	GIMAP5	0,18919	2	2	8,8	0	NA	NA	NA	NA	No	NA	0,008089	rs3918226
334	UKBB_CAD	ENSG00000106565	TMEM176	7	1,5E+08	1,5E+08	-1	547	48	330016	1	0,11096	protein_c	28959	TMEM176	3,00E-09	0,708327806	5	8,8	0	NA	NA	NA	NA	No	NA	0,000246	rs3918226
335	UKBB_CAD	ENSG00000002933	TMEM176	7	1,5E+08	1,5E+08	1	506	44	330867	1	0,15861	protein_c	55365	TMEM176	0,39981	NA	5	8,8	0	NA	NA	NA	NA	No	NA	0,000246	rs3918226
336	UKBB_CAD	ENSG00000002726	AOC1	7	1,5E+08	1,5E+08	1	695	52	330572	2	0,04952	protein_c	26	AOC1	1,24E-08	1	14	7	0	NA	NA	NA	NA	No	NA	1,35E-05	rs3918226
337	UKBB_CAD	ENSG00000055118	KCNH2	7	1,5E+08	1,5E+08	-1	343	46	322936	3	0,00305	protein_c	3757	KCNH2	0,99605	NA	47	12,19	0	NA	NA	NA	NA	No	NA	1,58E-12	rs3918226
338	UKBB_CAD	ENSG00000164867	NOS3	7	1,5E+08	1,5E+08	1	333	54	320486	2	0,01699	protein_c	4846	NOS3	2,21E-05	0,132489185	39	12,19	0	NA	NA	NA	NA	No	NA	1,58E-12	rs3918226
339	UKBB_CAD	ENSG00000181652	ATG9B	7	1,5E+08	1,5E+08	-1	318	53	320040	2	0,00947	protein_c	285973	ATG9B	1,85E-11	NA	36	12,19	0	NA	NA	NA	NA	No	NA	1,58E-12	rs3918226
340	UKBB_CAD	ENSG00000197150	ABCB8	7	1,5E+08	1,5E+08	1	335	51	319483	2	0,01315	protein_c	11194	ABCB8	4,60E-06	-0,816779123	35	15,01	0	NA	NA	NA	NA	No	NA	1,58E-12	rs3918226
341	UKBB_CAD	ENSG00000213199	ASIC3	7	1,5E+08	1,5E+08	1	306	46	320536	0,98903	0,16132	protein_c	9311	ASIC3	7,96E-07	0,064110614	18	15,01	0	NA	NA	NA	NA	No	NA	0,000559	rs3918226
342	UKBB_CAD	ENSG00000164885	CDK5	7	1,5E+08	1,5E+08	-1	308	43	321073	0,7806	0,21752	protein_c	1020	CDK5	0,94752	-0,194515947	14	15,01	0	NA	NA	NA	NA	No	NA	0,000559	rs3918226
343	UKBB_CAD	ENSG00000164889	SLC4A2	7	1,5E+08	1,5E+08	1	348	43	322024	0,69778	0,24266	protein_c	6522	SLC4A2	0,99996	0,287437707	14	15,01	1	8,00E-11	0	eQTLGen_	+	No	NA	0,000559	rs3918226
344	UKBB_CAD	ENSG00000164896	FASTK	7	1,5E+08	1,5E+08	-1	319	35	323695	0,17989	0,42862	protein_c	10922	FASTK	0,03053	NA	4	15,01	1	1,18E-05	0,03319	eQTLGen_	-	No	NA	0,000559	rs3918226
345	UKBB_CAD	ENSG00000164897	TMUB1	7	1,5E+08	1,5E+08	-1	313	35	323283	0,23742	0,40617	protein_c	83590	TMUB1	0,31489	NA	4	15,01	1	1,34E-45	0	eQTLGen_	+	No	NA	0,000559	rs3918226
346	UKBB_CAD	ENSG00000133612	AGAP3	7	1,5E+08	1,5E+08	1	443	53	323898	0,08037	0,46797	protein_c	116988	AGAP3	0,99138	NA	3	15,01	1	5,21E-12	0	eQTLGen_	-	No	NA	0,003163	rs3918226
347	UKBB_CAD	ENSG00000013374	NUB1	7	1,5E+08	1,5E+08	1	551	70	322534	-1	0,91999	protein_c	51667	NUB1	1,15E-05	0,438819908	0	0	0	NA	NA	NA	NA	Yes	IMR90	0,00357	rs3918226
348	UKBB_CAD	ENSG00000147687	TATDN1	8	1,3E+08	1,3E+08	-1	485	41	326608	0,99361	0,16021	protein_c	83940	TATDN1	1,73E-08	0,357863168	0	0	0	NA	NA	NA	NA	Yes	GM12878	1,47E-06	rs2954029
349	UKBB_CAD	ENSG00000147684	NDUFB9	8	1,3E+08	1,3E+08	1	470	42	327299	0,18455	0,42679	protein_c	4715	NDUFB9	0,04289	-0,016587227	0	0	0	NA	NA	NA	NA	Yes	GM12878	1,47E-06	rs2954029
350	UKBB_CAD	ENSG00000104549	SQLE	8	1,3E+08	1,3E+08	1	462	29	327585	-0,09689	0,53859	protein_c	6713	SQLE	0,91297	1	0	0	0	NA	NA	NA	NA	Yes	IMR90:Me	1,47E-06	rs2954029
351	UKBB_CAD	ENSG00000164961	KIAA0196	8	1,3E+08	1,3E+08	-1	603	24	327030	-0,41383	0,6605	protein_c	9897	KIAA0196	4,81E-13	-0,762157486	0	0	0	NA	NA	NA	NA	Yes	GM12878	1,47E-06	rs2954029
352	UKBB_CAD	ENSG00000156831	NSMCE2	8	1,3E+08	1,3E+08	1	1059	63	325215	0,1901	0,42462	protein_c	286053	NSMCE2	0,07245	-0,471934498	0	0	0	NA	NA	NA	NA	Yes	GM12878	1,47E-06	rs2954029
353	UKBB_CAD	ENSG00000173334	TRIB1	8	1,3E+08	1,3E+08	1	324	47	321154	4	4,53E-06	protein_c	10221	TRIB1	0,60659	-0,456914247	94	18,82	0	NA	NA	NA	NA	No	NA	5,24E-13	rs2954029
354	UKBB_CAD	ENSG00000198642	KLHL9	9	2,1E+07	2,1E+07	-1	346	15	325604	-0,64537	0,74066	protein_c	55958	KLHL9	0,98834	-0,351146078	0	0	0	NA	NA	NA	NA	Yes	Mesenchy	1,19E-16	rs2891168
355	UKBB_CAD	ENSG00000099810	MTAP	9	2,2E+07	2,2E+07	1	604	41	328370	5	2,17E-08	protein_c	4507	MTAP	0,00018	2	45	22,6	1	8,48E-08	0,00033	eQTLGen_	+	Yes	GM12878	1,48E-22	rs140458508;rs36228834;rs2891168
356	UKBB_CAD	ENSG00000264545	RP11-145I	9	2,2E+07	2,2E+07	1	863	61	326828	12	4,01E-32	protein_c	NA	NA	NA	NA	205	22,6	0	NA	NA	NA	NA	Yes	GM12878	1,00E-91	rs140458508;rs36228834;rs2891168;rs3217978
357	UKBB_CAD	ENSG00000224854	C9orf53	9	2,2E+07	2,2E+07	1	220	40	320865	10	1,72E-24	protein_c	51198	C9orf53	NA	NA	84	22,6	0	NA	NA	NA	NA	No	NA	4,03E-49	rs36228834;rs140458508;rs2891168;rs3217978
358	UKBB_CAD	ENSG00000147889	CDKN2A	9	2,2E+07	2,2E+07	-1	295	36	322227	12	6,13E-34	protein_c	1029	CDKN2A	0,35765	-0,259234065	132	22,6	3	6,36E-21	0	eQTLGen_	-	No	NA	4,03E-49	rs36228834;rs140458508;rs2891168;rs3217978
359	UKBB_CAD	ENSG00000147883	CDKN2B	9	2,2E+07	2,2E+07	-1	256	24	323728	12	1,31E-31	protein_c	1030	CDKN2B	0,28106	-2	148	22,6	6	#######	0	BIOS_eQT	-	Yes	IMR90:Me	1,86E-54	rs140458508;rs36228834;rs2891168;rs3217978
360	UKBB_CAD	ENSG00000160271	RALGDS	9	1,4E+08	1,4E+08	-1	552	62	321376	0,96507	0,16726	protein_c	5900	RALGDS	0,29671	-0,211408361	34	12,11	1	6,18E-08	0,00027	BIOS_eQT	-	No	NA	0,000691	rs507666
361	UKBB_CAD	ENSG00000148288	GBGT1	9	1,4E+08	1,4E+08	-1	392	56	320901	1	0,07191	protein_c	26301	GBGT1	0,0001	0,668450581	34	12,11	2	3,98E-17	0	BIOS_eQT	-	No	NA	0,000691	rs507666
362	UKBB_CAD	ENSG00000171102	OBP2B	9	1,4E+08	1,4E+08	-1	556	46	324549	1,62	0,05262	protein_c	29989	OBP2B	0,18297	-0,121398936	88	13,17	0	NA	NA	NA	NA	No	NA	6,21E-05	rs507666
363	UKBB_CAD	ENSG00000148296	SURF6	9	1,4E+08	1,4E+08	-1	443	36	328232	2	0,01087	protein_c	6838	SURF6	8,12E-05	-0,437227567	60	9	1	4,91E-19	0	BIOS_eQT	+	No	NA	1,34E-12	rs507666
364	UKBB_CAD	ENSG00000148297	MED22	9	1,4E+08	1,4E+08	-1	434	32	328390	0,28719	0,38698	protein_c	6837	MED22	0,00632	-0,130266294	21	7	1	2,51E-37	0	BIOS_eQT	+	No	NA	0,000192	rs507666
365	UKBB_CAD	ENSG00000148303	RPL7A	9	1,4E+08	1,4E+08	1	390	28	327858	0,41099	0,34054	protein_c	6130	RPL7A	0,97941	NA	16	6	0	NA	NA	NA	NA	No	NA	0,000192	rs507666
366	UKBB_CAD	ENSG00000148290	SURF1	9	1,4E+08	1,4E+08	-1	417	29	327384	0,46531	0,32085	protein_c	6834	SURF1	1,02E-13	NA	19	16,34	2	1,15E-61	0	xQTLServe	-	No	NA	0,000192	rs507666
367	UKBB_CAD	ENSG00000148291	SURF2	9	1,4E+08	1,4E+08	1	426	30	326634	0,49344	0,31085	protein_c	6835	SURF2	3,07E-07	NA	18	16,34	0	NA	NA	NA	NA	No	NA	0,000192	rs507666
368	UKBB_CAD	ENSG00000148248	SURF4	9	1,4E+08	1,4E+08	-1	453	28	325437	0,40044	0,34442	protein_c	6836	SURF4	0,68642	-0,490474887	18	16,34	0	NA	NA	NA	NA	No	NA	0,000192	rs507666
369	UKBB_CAD	ENSG00000198870	C9orf96	9	1,4E+08	1,4E+08	1	467	21	320215	0,6431	0,26008	protein_c	169436	C9orf96	2,55E-16	NA	20	16,34	0	NA	NA	NA	NA	No	NA	0,00022	rs507666
370	UKBB_CAD	ENSG00000148300	REXO4	9	1,4E+08	1,4E+08	-1	415	26	319488	1	0,12201	protein_c	57109	REXO4	9,99E-06	2	21	16,34	0	NA	NA	NA	NA	No	NA	0,00022	rs507666
371	UKBB_CAD	ENSG00000160323	ADAMTS1	9	1,4E+08	1,4E+08	1	538	33	308251	3	0,00501	protein_c	11093	ADAMTS1	7,88E-11	-0,276084391	38	16,34	0	NA	NA	NA	NA	No	NA	0,00022	rs507666
372	UKBB_CAD	ENSG00000160325	CACFD1	9	1,4E+08	1,4E+08	1	418	43	296352	3	0,00212	protein_c	11094	CACFD1	0,00575	NA	32	10,16	1	5,70E-21	0	BIOS_eQT	-	No	NA	0,000512	rs507666
373	UKBB_CAD	ENSG00000160326	SLC2A6	9	1,4E+08	1,4E+08	-1	377	43	290477	3	0,00091	protein_c	11182	SLC2A6	0,00037	-0,033260488	32	10,16	1	6,93E-05	0,03899	BIOS_eQT	-	No	NA	0,000512	rs507666
374	UKBB_CAD	ENSG00000187616	TMEM8C	9	1,4E+08	1,4E+08	-1	245	43	272854	3	0,00165	protein_c	389827	TMEM8C	0,00217	0,106132843	21	10,16	0	NA	NA	NA	NA	No	NA	0,000512	rs507666
375	UKBB_CAD	ENSG00000197859	ADAMTSL	9	1,4E+08	1,4E+08	1	406	63	296987	2	0,00966	protein_c	9719	ADAMTSL	0,07106	NA	16	10,16	0	NA	NA	NA	NA	No	NA	0,000512	rs507666
376	UKBB_CAD	ENSG00000197321	SVIL	10	3E+07	3E+07	-1	1648	99	330606	0,17968	0,4287	protein_c	6840	SVIL	0,9994	-0,110100822	0	0	0	NA	NA	NA	NA	Yes	IMR90:Me	8,25E-11	rs1887318
377	UKBB_CAD	ENSG00000165757	KIAA1462	10	3E+07	3E+07	-1	634	55	324151	6	1,68E-09	protein_c	57608	KIAA1462	0,10429	1	148	19,05	3	4,02E-16	2,57E-13	GTEx_v7_	+	Yes	IMR90:Me	4,12E-12	rs1887318
378	UKBB_CAD	ENSG00000107968	MAP3K8	10	3,1E+07	3,1E+07	1	372	30	325776	-1	0,89829	protein_c	1326	MAP3K8	0,87095	-0,406879135	0	0	0	NA	NA	NA	NA	Yes	IMR90:Me	8,25E-11	rs1887318
379	UKBB_CAD	ENSG00000169813	HNRNPF	10	4,4E+07	4,4E+07	-1	445	27	327914	2	0,05629	protein_c	3185	HNRNPF	0,86404	1	0	0	1	8,27E-06	0,02337	eQTLGen_	+	No	NA	0,025665	rs1870634
380	UKBB_CAD	ENSG00000196793	ZNF239	10	4,4E+07	4,4E+07	-1	483	38	325945	0,09675	0,46146	protein_c	8187	ZNF239	0,00087	0,570932296	0	0	1	2,87E-23	0	eQTLGen_	-	No	NA	0,025665	rs1870634
381	UKBB_CAD	ENSG00000198298	ZNF485	10	4,4E+07	4,4E+07	1	388	41	327033	-0,35135	0,63734	protein_c	220992	ZNF485	3,53E-05	0,070203911	3	5	0	NA	NA	NA	NA	Yes	IMR90:Me	2,85E-05	rs1870634;rs1632484:rs1870634;rs1632484
382	UKBB_CAD	ENSG00000169740	ZNF32	10	4,4E+07	4,4E+07	-1	396	32	327267	0,22037	0,41279	protein_c	7580	ZNF32	0,27621	NA	17	8	0	NA	NA	NA	NA	Yes	IMR90:Me	2,85E-05	rs1870634;rs1870634:rs1632484:rs1870634;rs1632484
383	UKBB_CAD	ENSG00000107562	CXCL12	10	4,5E+07	4,5E+07	-1	823	65	330685	6	6,32E-10	protein_c	6387	CXCL12	0,11859	NA	237	21,3	0	NA	NA	NA	NA	Yes	IMR90:Me	9,54E-13	rs1870634;rs1632484;rs1870634:rs1632484:rs1870634
384	UKBB_CAD	ENSG00000269515	AL137026	10	4,5E+07	4,5E+07	1	502	57	330837	2	0,02841	protein_c	NA	NA	NA	NA	48	8	0	NA	NA	NA	NA	No	NA	0,000114	rs1632484;rs1870634
385	UKBB_CAD	ENSG00000107551	RASSF4	10	4,5E+07	4,5E+07	1	481	54	326484	-1	0,88477	protein_c	83937	RASSF4	2,05E-11	0,297552094	0	0	0	NA	NA	NA	NA	Yes	IMR90:Me	2,85E-05	rs1870634:rs1870634;rs1632484
386	UKBB_CAD	ENSG00000165511	C10orf25	10	4,5E+07	4,5E+07	-1	368	37	326817	-2	0,9623	protein_c	220979	C10orf25	0,01257	-0,929200156	0	0	0	NA	NA	NA	NA	Yes	IMR90:Me	0,005727	rs1870634:rs1632484
387	UKBB_CAD	ENSG00000165512	ZNF22	10	4,5E+07	4,6E+07	1	370	35	327277	-2	0,96018	protein_c	7570	ZNF22	0,12668	-0,107262233	0	0	0	NA	NA	NA	NA	Yes	IMR90:Me	0,005727	rs1870634:rs1632484
388	UKBB_CAD	ENSG00000204174	NPY4R	10	4,7E+07	4,7E+07	1	5	3	228720	0,92597	0,17723	protein_c	5540	NPY4R	6,12E-09	0,487893073	0	0	0	NA	NA	NA	NA	Yes	Mesenchy	2,85E-05	rs1870634:rs1632484:rs1870634;rs1632484
389	UKBB_CAD	ENSG00000107796	ACTA2	10	9,1E+07	9,1E+07	-1	573	55	324983	0,76678	0,22161	protein_c	59	ACTA2	0,73422	0,078588985	0	0	2	4,35E-13	0	eQTLGen_	+	No	NA	1,55E-15	rs2246942
390	UKBB_CAD	ENSG00000138135	CH25H	10	9,1E+07	9,1E+07	-1	314	39	325863	6	1,84E-10	protein_c	9023	CH25H	0,34229	0,282167007	59	21,8	0	NA	NA	NA	NA	No	NA	3,51E-16	rs2246942
391	UKBB_CAD	ENSG00000107798	LIPA	10	9,1E+07	9,1E+07	-1	1058	68	317319	4	1,15E-05	protein_c	3988	LIPA	0,01128	-0,139630972	59	21,8	2	3.2716700	0	BIOS_eQT	+	No	NA	3,51E-16	rs2246942
392	UKBB_CAD	ENSG00000119922	IFIT2	10	9,1E+07	9,1E+07	1	412	43	313183	2	0,02059	protein_c	3433	IFIT2	0,00148	-0,433526105	4	11,96	0	NA	NA	NA	NA	No	NA	1,37E-12	rs2246942
393	UKBB_CAD	ENSG00000185745	IFIT1	10	9,1E+07	9,1E+07	1	429	38	311456	1	0,09435	protein_c	3434	IFIT1	0,00535	0,443641667	0	0	1	5,82E-06	0,01656	eQTLGen_	-	No	NA	9,53E-14	rs2246942
394	UKBB_CAD	ENSG00000152778	IFIT5	10	9,1E+07	9,1E+07	1	370	40	315948	1	0,09707	protein_c	24138	IFIT5	0,05814	1	0	0	2	6,46E-25	0	eQTLGen_	-	No	NA	1,55E-15	rs2246942
395	UKBB_CAD	ENSG00000198408	MGEA5	10	1E+08	1E+08	-1	189	29	324094	-0,40538	0,6574	protein_c	10724	MGEA5	0,7141	-2	1	5	0	NA	NA	NA	NA	No	NA	NA	rs11191416
396	UKBB_CAD	ENSG00000120049	KCNIP2	10	1E+08	1E+08	-1	175	26	324885	-0,17516	0,56952	protein_c	30819	KCNIP2	0,01869	NA	1	5	0	NA	NA	NA	NA	No	NA	NA	rs11191416
397	UKBB_CAD	ENSG00000120029	C10orf76	10	1E+08	1E+08	-1	507	35	324224	-0,28647	0,61274	protein_c	79591	C10orf76	0,05534	0,414157392	3	9	0	NA	NA	NA	NA	No	NA	0,025663	rs11191416
398	UKBB_CAD	ENSG00000166189	HPS6	10	1E+08	1E+08	1	178	37	322585	-0,63511	0,73732	protein_c	79803	HPS6	0,30174	-0,084659977	1	7	0	NA	NA	NA	NA	No	NA	0,025663	rs11191416
399	UKBB_CAD	ENSG00000198728	LDB1	10	1E+08	1E+08	-1	209	37	317819	0,08515	0,46607	protein_c	8861	LDB1	0,87945	NA	1	7	0	NA	NA	NA	NA	No	NA	0,025663	rs11191416
400	UKBB_CAD	ENSG00000148840	PPRC1	10	1E+08	1E+08	1	218	37	318568	0,5834	0,27981	protein_c	23082	PPRC1	1	-0,462640235	1	7	0	NA	NA	NA	NA	No	NA	0,025663	rs11191416
401	UKBB_CAD	ENSG00000077150	NFKB2	10	1E+08	1E+08	1	190	39	321456	1	0,08517	protein_c	4791	NFKB2	0,9997	-0,681516998	3	7	0	NA	NA	NA	NA	Yes	GM12878	0,000192	rs11191416
402	UKBB_CAD	ENSG00000059915	PSD	10	1E+08	1E+08	-1	250	52	323646	2	0,05839	protein_c	5662	PSD	0,98733	NA	13	11,36	0	NA	NA	NA	NA	No	NA	0,001109	rs11191416
403	UKBB_CAD	ENSG00000107872	FBXL15	10	1E+08	1E+08	1	230	49	323632	1	0,08126	protein_c	79176	FBXL15	0,0021	-2	13	11,36	0	NA	NA	NA	NA	No	NA	0,001109	rs11191416
404	UKBB_CAD	ENSG00000107874	CUEDC2	10	1E+08	1E+08	-1	259	42	324309	2	0,063	protein_c	79004	CUEDC2	0,07191	-0,180115673	16	11,36	0	NA	NA	NA	NA	No	NA	0,001109	rs11191416
405	UKBB_CAD	ENSG00000120055	C10orf95	10	1E+08	1E+08	-1	279	40	326853	2	0,01755	protein_c	79946	C10orf95	0,01139	-0,659447928	17	11,36	0	NA	NA	NA	NA	Yes	IMR90	0,000192	rs11191416

406	UKBB_CAD	ENSG00000138111	TMEM180	10	1E+08	1E+08	1	318	33	328827	2	0,05749	protein_c	79847	TMEM180	0,00018	3	29	11,36	12	#######	0	BIOS_eQT	-	Yes	IMR90	2,28E-08	rs11191416
407	UKBB_CAD	ENSG00000138107	ACTR1A	10	1E+08	1E+08	-1	361	25	329758	2	0,04262	protein_c	10121	ACTR1A	0,94123	-0,272535339	38	16,96	11	1,22E-24	0	eQTLGen_	-	Yes	GM12878	2,28E-08	rs11191416
408	UKBB_CAD	ENSG00000107882	SUFU	10	1E+08	1E+08	1	615	24	330622	2	0,02767	protein_c	51684	SUFU	0,99916	0,124860143	86	20,5	4	3,39E-06	0,00936	BIOS_eQT	-	Yes	GM12878	3,04E-06	rs11191416
409	UKBB_CAD	ENSG00000171206	TRIM8	10	1E+08	1E+08	1	265	22	330383	2	0,01878	protein_c	81603	TRIM8	0,99201	-0,521290389	48	20,5	8	2,40E-40	0	BIOS_eQT	+	Yes	IMR90	3,04E-06	rs11191416
410	UKBB_CAD	ENSG00000138175	ARL3	10	1E+08	1E+08	-1	335	27	330256	3	0,00165	protein_c	403	ARL3	0,7161	-0,600025748	65	20,5	8	2,28E-36	0	xQTLServe	-	Yes	IMR90:Me	3,04E-06	rs11191416
411	UKBB_CAD	ENSG00000156398	SFXN2	10	1E+08	1E+08	1	338	28	329901	3	0,00335	protein_c	118980	SFXN2	0,00108	-0,837011237	54	15,08	13	#######	0	BIOS_eQT	+	Yes	IMR90:Me	2,28E-08	rs11191416
412	UKBB_CAD	ENSG00000166272	WBP1L	10	1E+08	1E+08	1	480	39	329514	5	2,13E-06	protein_c	54838	WBP1L	0,90939	0,614640467	124	15,56	6	5,06E-18	0	BIOS_eQT	-	Yes	Aorta:GM	5,58E-09	rs11191416
413	UKBB_CAD	ENSG00000148795	CYP17A1	10	1E+08	1E+08	-1	324	27	329265	5	1,02E-07	protein_c	1586	CYP17A1	0,03503	0,086681977	121	15,56	11	1,19E-28	0	BIOS_eQT	-	No	NA	5,58E-09	rs11191416
414	UKBB_CAD	ENSG00000166275	C10orf32	10	1E+08	1E+08	1	343	25	329186	6	1,80E-08	protein_c	119032	C10orf32	0,03164	0,490358211	163	18,11	15	0	0	xQTLServe	+	Yes	IMR90:Me	5,58E-09	rs11191416
415	UKBB_CAD	ENSG00000270316	C10orf32-	10	1E+08	1E+08	1	443	28	329693	5	3,22E-08	protein_c	1E+08	C10orf32-	NA	NA	198	18,11	0	NA	NA	NA	NA	Yes	IMR90:Me	5,58E-09	rs11191416
416	UKBB_CAD	ENSG00000214435	AS3MT	10	1E+08	1E+08	1	397	24	330083	5	1,79E-07	protein_c	57412	AS3MT	5,09E-07	0,726881178	176	18,11	15	0	0	xQTLServe	+	Yes	IMR90:Me	5,58E-09	rs11191416
417	UKBB_CAD	ENSG00000148842	CNNM2	10	1E+08	1E+08	1	774	22	330542	4	5,20E-06	protein_c	54805	CNNM2	0,99411	-0,511524122	280	18,11	4	6,76E-10	0	eQTLGen_	+	Yes	GM12878	2,22E-08	rs11191416
418	UKBB_CAD	ENSG00000076685	NT5C2	10	1E+08	1E+08	-1	582	29	329239	4	6,00E-05	protein_c	22978	NT5C2	0,0522	0,04832858	148	17,62	16	3.2717000	0	BIOS_eQT	-	Yes	IMR90:Me	2,28E-08	rs11191416
419	UKBB_CAD	ENSG00000235376	RPEL1	10	1,1E+08	1,1E+08	1	303	25	321226	1	0,0729	protein_c	729020	RPEL1	0,0024	NA	40	15,11	0	NA	NA	NA	NA	No	NA	1,17E-07	rs11191416
420	UKBB_CAD	ENSG00000148798	INA	10	1,1E+08	1,1E+08	1	269	21	318917	1	0,06905	protein_c	9118	INA	0,29292	-0,860661456	41	15,11	0	NA	NA	NA	NA	Yes	Aorta:GM	2,74E-06	rs11191416
421	UKBB_CAD	ENSG00000156374	PCGF6	10	1,1E+08	1,1E+08	-1	291	22	319970	2	0,04186	protein_c	84108	PCGF6	0,00025	-0,491784974	45	15,11	1	4,84E-29	0	BIOS_eQT	-	Yes	IMR90	2,74E-06	rs11191416
422	UKBB_CAD	ENSG00000148835	TAF5	10	1,1E+08	1,1E+08	1	199	29	319742	1	0,09398	protein_c	6877	TAF5	0,99956	-0,333196464	17	8	0	NA	NA	NA	NA	No	NA	0,000356	rs11191416
423	UKBB_CAD	ENSG00000173915	USMG5	10	1,1E+08	1,1E+08	-1	185	29	315755	1	0,11815	protein_c	84833	USMG5	0,24335	-0,219458172	10	6	14	3.2717000	0	BIOS_eQT	-	No	NA	2,28E-08	rs11191416
424	UKBB_CAD	ENSG00000148843	PDCD11	10	1,1E+08	1,1E+08	1	318	40	319197	0,90125	0,18373	protein_c	22984	PDCD11	0,46845	-0,805378431	10	6	1	9,23E-10	6,19E-06	BIOS_eQT	+	No	NA	0,000356	rs11191416
425	UKBB_CAD	ENSG00000138172	CALHM2	10	1,1E+08	1,1E+08	-1	272	37	318612	0,39068	0,34802	protein_c	51063	CALHM2	5,29E-07	-0,689941205	4	6	8	8,18E-22	0	eQTLGen_	-	No	NA	2,28E-08	rs11191416
426	UKBB_CAD	ENSG00000185933	CALHM1	10	1,1E+08	1,1E+08	-1	283	35	319887	0,34026	0,36683	protein_c	255022	CALHM1	9,36E-05	0,209560501	4	6	1	3,12E-06	0,00235	BIOS_eQT	+	No	NA	0,022659	rs11191416
427	UKBB_CAD	ENSG00000183128	CALHM3	10	1,1E+08	1,1E+08	-1	289	34	322423	0,36656	0,35697	protein_c	119395	CALHM3	NA	-0,052014803	2	1	0	NA	NA	NA	NA	No	NA	0,022659	rs11191416
428	UKBB_CAD	ENSG00000107954	NEURL1	10	1,1E+08	1,1E+08	1	577	57	325351	1	0,08158	protein_c	9148	NEURL1	0,53757	0,420609325	3	4	0	NA	NA	NA	NA	No	NA	0,018987	rs11191416
429	UKBB_CAD	ENSG00000107957	SH3PXD2A	10	1,1E+08	1,1E+08	-1	1041	80	327927	1	0,13403	protein_c	9644	SH3PXD2A	0,23274	-1	1	4	8	2,38E-23	0	eQTLGen_	+	No	NA	2,28E-08	rs11191416
430	UKBB_CAD	ENSG00000166436	TRIM66	11	8633584	8693413	-1	484	38	324268	0,98676	0,16188	protein_c	9866	TRIM66	NA	NA	0	0	1	6,20E-07	0,00198	eQTLGen_	-	No	NA	0,007557	rs10840293
431	UKBB_CAD	ENSG00000166452	AKIP1	11	8932686	8941631	1	403	21	323716	1	0,06714	protein_c	56672	AKIP1	1,86E-05	NA	0	0	5	2,33E-15	0	eQTLGen_	-	No	NA	1,30E-08	rs10840293
432	UKBB_CAD	ENSG00000166471	TMEM41B	11	9302201	9336327	-1	462	31	325032	3	0,00054	protein_c	440026	TMEM41B	0,02019	2	0	0	4	5,88E-06	0,0167	eQTLGen_	+	No	NA	0,015733	rs10840293
433	UKBB_CAD	ENSG00000205339	IPO7	11	9406169	9469673	1	479	34	324797	3	0,00174	protein_c	10527	IPO7	1	-1	1	2	0	NA	NA	NA	NA	No	NA	0,021884	rs10840293
434	UKBB_CAD	ENSG00000268403	AC132192	11	9481747	9482010	-1	225	19	323129	2	0,02179	protein_c	644656	LOC64465	NA	NA	0	0	0	NA	NA	NA	NA	Yes	Mesenchy	1,30E-08	rs10840293
435	UKBB_CAD	ENSG00000166478	ZNF143	11	9481866	9550071	1	405	35	322377	3	0,00483	protein_c	7702	ZNF143	0,02197	-1	6	4	4	3,75E-16	0	eQTLGen_	-	Yes	Mesenchy	1,30E-08	rs10840293
436	UKBB_CAD	ENSG00000166483	WEE1	11	9595228	9615004	1	312	38	324064	3	0,00172	protein_c	7465	WEE1	0,99711	NA	47	15,01	14	8,76E-49	0	BIOS_eQT	-	Yes	Aorta:GM	1,30E-08	rs10840293
437	UKBB_CAD	ENSG00000133789	SWAP70	11	9685624	9774538	1	712	34	324268	5	1,43E-06	protein_c	23075	SWAP70	0,34884	-0,389632948	321	18,57	18	#######	0	BIOS_eQT	-	Yes	GM12878	6,88E-09	rs10840293
438	UKBB_CAD	ENSG00000133812	SBF2	11	9800214	1E+07	-1	2249	48	326892	4	3,09E-05	protein_c	81846	SBF2	0,38005	0,210546538	578	20,1	12	#######	0	BIOS_eQT	-	Yes	IMR90:Me	6,88E-09	rs10840293
439	UKBB_CAD	ENSG00000148926	ADM	11	1E+07	1E+07	1	331	20	326190	2	0,02476	protein_c	133	ADM	0,57663	-1	43	19,05	11	#######	0	BIOS_eQT	-	Yes	Aorta:GM	1,30E-08	rs10840293
440	UKBB_CAD	ENSG00000133805	AMPD3	11	1E+07	1,1E+07	1	1185	92	325101	2	0,06215	protein_c	272	AMPD3	1,33E-08	0,348490889	40	19,05	0	NA	NA	NA	NA	Yes	Aorta:GM	1,30E-08	rs10840293
441	UKBB_CAD	ENSG00000110315	RNF141	11	1,1E+07	1,1E+07	-1	494	53	321494	-0,49461	0,68956	protein_c	50862	RNF141	0,0007	-0,118241395	0	0	0	NA	NA	NA	NA	Yes	Mesenchy	0,000328	rs10840293
442	UKBB_CAD	ENSG00000072952	MRVI1	11	1,1E+07	1,1E+07	-1	813	63	321577	-0,11213	0,54464	protein_c	10335	MRVI1	0,00184	-0,670039603	0	0	3	2,14E-10	0	eQTLGen_	-	Yes	IMR90:Me	1,30E-08	rs10840293
443	UKBB_CAD	ENSG00000170962	PDGFD	11	1E+08	1E+08	-1	1298	82	327394	2	0,06392	protein_c	80310	PDGFD	0,01595	1	50	15,12	2	8,03E-22	0	eQTLGen_	-	Yes	Aorta:GM	6,69E-05	rs2839812
444	UKBB_CAD	ENSG00000196954	CASP4	11	1E+08	1E+08	-1	366	26	323449	1	0,09112	protein_c	837	CASP4	8,21E-07	0,227584853	0	0	0	NA	NA	NA	NA	Yes	IMR90:Me	0,000208	rs2839812
445	UKBB_CAD	ENSG00000137752	CASP1	11	1E+08	1E+08	-1	611	26	322056	-0,02024	0,50807	protein_c	834	CASP1	0,1368	1	0	0	0	NA	NA	NA	NA	Yes	IMR90:Me	0,000208	rs2839812
446	UKBB_CAD	ENSG00000204397	CARD16	11	1E+08	1E+08	-1	549	21	321654	-0,29026	0,61419	protein_c	114769	CARD16	0,04016	-0,174322066	0	0	0	NA	NA	NA	NA	Yes	IMR90:Me	0,000208	rs2839812
447	UKBB_CAD	ENSG00000255221	CARD17	11	1E+08	1E+08	-1	367	16	323670	-0,49962	0,69133	protein_c	440068	CARD17	0,0003	0,46179097	0	0	0	NA	NA	NA	NA	Yes	IMR90:Me	0,000208	rs2839812
448	UKBB_CAD	ENSG00000152578	GRIA4	11	1,1E+08	1,1E+08	1	1135	58	327525	0,36675	0,3569	protein_c	2893	GRIA4	0,98875	-0,809001848	0	0	0	NA	NA	NA	NA	Yes	IMR90:Me	0,000208	rs2839812
449	UKBB_CAD	ENSG00000170903	MSANTD4	11	1,1E+08	1,1E+08	-1	308	23	329131	2	0,06058	protein_c	84437	MSANTD4	0,00546	-0,743698695	0	0	0	NA	NA	NA	NA	Yes	IMR90:Me	0,000208	rs2839812
450	UKBB_CAD	ENSG00000182359	KBTBD3	11	1,1E+08	1,1E+08	-1	247	30	327228	2	0,04286	protein_c	143879	KBTBD3	2,60E-05	0,499796283	0	0	0	NA	NA	NA	NA	Yes	IMR90:Me	0,000208	rs2839812
451	UKBB_CAD	ENSG00000149313	AASDHPPT	11	1,1E+08	1,1E+08	1	240	27	327129	1	0,08011	protein_c	60496	AASDHPPT	0,08394	-0,525706188	0	0	0	NA	NA	NA	NA	Yes	IMR90:Me	0,000208	rs2839812
452	UKBB_CAD	ENSG00000198707	CEP290	12	8,8E+07	8,9E+07	-1	348	21	317428	2	0,05698	protein_c	80184	CEP290	1,04E-34	-0,583026543	0	0	0	NA	NA	NA	NA	Yes	Mesenchy	0,002041	rs2681472
453	UKBB_CAD	ENSG00000139324	TMTC3	12	8,9E+07	8,9E+07	1	268	14	319925	0,85118	0,19734	protein_c	160418	TMTC3	0,00905	-2	0	0	0	NA	NA	NA	NA	Yes	Mesenchy	0,002041	rs2681472
454	UKBB_CAD	ENSG00000049130	KITLG	12	8,9E+07	8,9E+07	-1	356	14	326933	0,77743	0,21845	protein_c	4254	KITLG	0,50853	-0,184130758	0	0	0	NA	NA	NA	NA	Yes	IMR90:hE	1,83E-07	rs2681472
455	UKBB_CAD	ENSG00000139318	DUSP6	12	9E+07	9E+07	-1	316	34	325175	1	0,0847	protein_c	1848	DUSP6	0,91121	-0,308177435	21	23,5	0	NA	NA	NA	NA	Yes	IMR90:hE	0,00164	rs2681472
456	UKBB_CAD	ENSG00000139323	POC1B	12	9E+07	9E+07	-1	593	37	327914	5	9,53E-07	protein_c	282809	POC1B	5,89E-05	-1	216	20,8	9	#######	0	BIOS_eQT	-	No	NA	1,39E-10	rs2681472
457	UKBB_CAD	ENSG00000257594	GALNT4	12	9E+07	9E+07	-1	310	21	328580	5	2,42E-07	protein_c	8693	GALNT4	0,11293	0,062534855	143	20,8	7	9,07E-64	0	GTEx_v7_	+	No	NA	1,39E-10	rs2681472
458	UKBB_CAD	ENSG00000259075	POC1B-GA	12	9E+07	9E+07	-1	310	21	328580	5	2,42E-07	protein_c	1E+08	POC1B-GA	0,0376	0,027960093	143	20,8	2	3,26E-08	4,58E-05	BIOS_eQT	-	No	NA	3,39E-09	rs2681472
459	UKBB_CAD	ENSG00000070961	ATP2B1	12	9E+07	9E+07	-1	418	36	327383	5	1,42E-07	protein_c	490	ATP2B1	1	-2	105	20,8	6	2,55E-12	1,11E-08	GTEx_v7_	+	No	NA	7,63E-11	rs2681472
460	UKBB_CAD	ENSG00000139329	LUM	12	9,1E+07	9,2E+07	-1	353	25	328897	-0,77505	0,78085	protein_c	4060	LUM	0,10192	0,839935967	0	0	0	NA	NA	NA	NA	Yes	IMR90:Me	0,002041	rs2681472
461	UKBB_CAD	ENSG00000185046	ANKS1B	12	9,9E+07	1E+08	-1	3865	201	326773	-1	0,84725	protein_c	56899	ANKS1B	0,99277	NA	0	0	9	0	0	xQTLServe	NA	No	NA	9,47E-14	rs67180937
462	UKBB_CAD	ENSG00000174456	C12orf76	12	1,1E+08	1,1E+08	-1	428	31	323011	-0,31274	0,62276	protein_c	400073	C12orf76	0,0001	-0,549717679	0	0	1	1,35E-05	0,03763	eQTLGen_	+	No	NA	0,001295	rs10774625
463	UKBB_CAD	ENSG00000111229	ARPC3	12	1,1E+08	1,1E+08	-1	341	19	319277	-0,87602	0,80949	protein_c	10094	ARPC3	0,93151	-0,463582933	0	0	1	1,25E-05	0,0351	eQTLGen_	+	No	NA	0,002774	rs10774625
464	UKBB_CAD	ENSG00000111231	GPN3	12	1,1E+08	1,1E+08	-1	327	20	318416	-0,7932	0,78617	protein_c	51184	GPN3	0,0001	-0,618749154	0	0	5	#######	0	eQTLGen_	+	No	NA	0,000298	rs10774625
465	UKBB_CAD	ENSG00000204856	FAM216A	12	1,1E+08	1,1E+08	1	335	19	318463	-0,83803	0,79899	protein_c	29902	FAM216A	0,00112	-0,854490491	0	0	5	2,93E-28	0	eQTLGen_	+	No	NA	0,000298	rs10774625
466	UKBB_CAD	ENSG00000111237	VPS29	12	1,1E+08	1,1E+08	-1	308	18	317985	-0,82959	0,79661	protein_c	51699	VPS29	0,93474	-0,696582918	0	0	4	4,03E-13	0	eQTLGen_	-	No	NA	0,000298	rs10774625
467	UKBB_CAD	ENSG00000204852	TCTN1	12	1,1E+08	1,1E+08	1	287	17	317860	-0,69538	0,75659	protein_c	79600	TCTN1	1,87E-09	-0,456703465	0	0	7	1,05E-10	0	eQTLGen_	-	No	NA	0,000298	rs10774625
468	UKBB_CAD	ENSG00000186298	PPP1CC	12	1,1E+08	1,1E+08	-1	297	16	321519	-0,72	0,76424	protein_c	5501	PPP1CC	0,94491	NA	0	0	0	NA	NA	NA	NA	Yes	Mesenchy	0,036071	rs10774625
469	UKBB_CAD	ENSG00000173093	CCDC63	12	1,1E+08	1,1E+08	1	498	45	326607	2	0,0097	protein_c	160762	CCDC63	4,48E-10	0,140065731	97	16,57	0	NA	NA	NA	NA	No	NA	9,31E-05	rs10774625
470	UKBB_CAD	ENSG00000111245	MYL2	12	1,1E+08	1,1E+08	-1	352	27	327009	3	0,00238	protein_c	4633	MYL2	0,01681	-0,496131607	123	17,3	0	NA	NA	NA	NA	No	NA	9,31E-05	rs10774625
471	UKBB_CAD	ENSG00000111249	CUX2	12	1,1E+08	1,1E+08	1	548	38	328543	3	0,00449	protein_c	23316	CUX2	0,99966	-1	163	21,7	0	NA	NA	NA	NA	Yes	IMR90:hE	2,40E-10	rs10774625
472	UKBB_CAD	ENSG00000198324	FAM109A	12	1,1E+08	1,1E+08	-1	204	19	329422	4	0,00011	protein_c	144717	FAM109A	0,00515	-0,607261333	119	21,7	27	1,54E-57	0	BIOS_eQT	-	No	NA	2,40E-10	rs10774625
473	UKBB_CAD	ENSG00000111252	SH2B3	12	1,1E+08	1,1E+08	1	172	18	325281	5	3,97E-08	protein_c	10019	SH2B3	0,00592	-0,243853728	114	17,18	31	#######	0	BIOS_eQT	+	No	NA	9,22E-14	rs10774625
474	UKBB_CAD	ENSG00000204842	ATXN2	12	1,1E+08	1,1E+08	-1	245	19	323435	6	1,85E-08	protein_c	6311	ATXN2	0,99986	-0,359942109	171	17,18	0	NA	NA	NA	NA	Yes	IMR90:Me	9,22E-14	rs10774625
475	UKBB_CAD	ENSG00000089234	BRAP	12	1,1E+08	1,1E+08	-1	145	13	324084	5	2,31E-06	protein_c	8315	BRAP	0,0157	-0,900035073	104	12,92	0	NA	NA	NA	NA	Yes	GM12878	1,68E-11	rs10774625
476	UKBB_CAD	ENSG00000111271	ACAD10	12	1,1E+08	1,1E+08	1	173	14	323302	4	6,85E-06	protein_c	80724	ACAD10	4,01E-20	-0,311041181	120	16,28	25	1,02E-22	0	eQTLGen_	-	Yes	GM12878	2,05E-09	rs10774625
477	UKBB_CAD	ENSG00000257767	RP11-162I	12	1,1E+08	1,1E+08	1	164	11	325270	4	1,93E-05	protein_c	NA	NA	NA	NA	121	16,28	1	1,68E-07	9,90E-05	BIOS_eQT	-	No	NA	2,54E-09	rs10774625
478	UKBB_CAD	ENSG00000111275	ALDH2	12	1,1E+08	1,1E+08	1	212	10	327816	4	4,19E-05	protein_c	217	ALDH2	1,73E-06	NA	166	16,28	23	#######	0	xQTLServe	-	Yes	IMR90:Me	2,54E-09	rs10774625
479	UKBB_CAD	ENSG00000089022	MAPKAPK	12	1,1E+08	1,1E+08	1	297	8	330554	4	6,27E-05	protein_c	8550	MAPKAPK	0,02342	NA	240	19,64	25	#######	0	BIOS_eQT	-	Yes	GM12878	2,54E-09	rs10774625
480	UKBB_CAD	ENSG00000198270	TMEM116	12	1,1E+08	1,1E+08	-1	305	12	329543	4	1,76E-05	protein_c	89894	TMEM116	1,15E-09	-0,6208399	237	19,64	31	3.2717000	0	BIOS_eQT	-	No	NA	7,36E-10	rs10774625
481	UKBB_CAD	ENSG00000089248	ERP29	12	1,1E+08	1,1E+08	1	169	12	328584	4	2,29E-05	protein_c	10961	ERP29	0,47065	-0,618385075	122	16,24	22	1,93E-53	0	BIOS_eQT	+	No	NA	7,36E-10	rs10774625
482	UKBB_CAD	ENSG00000111300	NAA25	12	1,1E+08	1,1E+08	-1	241	18	324834	4	1,87E-05	protein_c	80018	NAA25	0,99999	-1	145	19,29	25	6,40E-98	0	BIOS_eQT	-	No	NA	7,36E-10	rs10774625
483	UKBB_CAD	ENSG00000135148	TRAFD1	12	1,1E+08	1,1E+08	1	147	16	320837	4	1,54E-05	protein_c	10906	TRAFD1	1,83E-05	-1	83	19,29	1	6,52E-07	0,00204	eQTLGen_	+	Yes	GM12878	2,39E-09	rs10774625
484	UKBB_CAD	ENSG00000173064	HECTD4	12	1,1E+08	1,1E+08	-1	380	21	325273	4	2,22E-05	protein_c	283450	HECTD4	1	NA	198	19,29	4	4,28E-06	0,00048	BIOS_eQT	+	No	NA	2,39E-09	rs10774625
485	UKBB_CAD	ENSG00000089009	RPL6	12	1,1E+08	1,1E+08	-1	134	17	327031	4	6,87E-05	protein_c	6128	RPL6	0,98829	0,026755082	67	15,46	0	NA	NA	NA	NA	No	NA	1,03E-08	rs10774625
486	UKBB_CAD	ENSG00000179295	PTPN11	12	1,1E+08	1,1E+08	1	281	18	326879	3	0,00033	protein_c	5781	PTPN11	0,99988	-2	60	15,46	19	4,28E-25	0	BIOS_eQT	-	No	NA	1,03E-08	rs10774625
487	UKBB_CAD	ENSG00000089127	OAS1	12	1,1E+08	1,1E+08	1	375	20	324546	0,40411	0,34306	protein_c	4938	OAS1	0,00124	-0,362759403	0	0	1	5,71E-08	0,00024	eQTLGen_	-	No	NA	0,003465	rs10774625

488	UKBB_CAD	ENSG00000257218	GATC	12	1,2E+08	1,2E+08	1	381	31	329553	0,96674	0,16684	protein_c	283459	GATC	7,79E-05	1	0	0	1	7,43E-07	0,00235	eQTLGen_	+	No	NA	0,036516	rs2244608
489	UKBB_CAD	ENSG00000110871	COQ5	12	1,2E+08	1,2E+08	-1	462	23	330004	1	0,11928	protein_c	84274	COQ5	7,95E-05	0,02662786	0	0	6	6,35E-17	0	eQTLGen_	+	No	NA	1,30E-06	rs2244608
490	UKBB_CAD	ENSG00000022840	RNF10	12	1,2E+08	1,2E+08	1	516	39	329228	1	0,11025	protein_c	9921	RNF10	0,50296	0,037115157	4	2	5	5,43E-09	3,88E-05	eQTLGen_	+	No	NA	0,015926	rs2244608
491	UKBB_CAD	ENSG00000167272	POP5	12	1,2E+08	1,2E+08	-1	369	41	328604	1	0,11328	protein_c	51367	POP5	1,28E-05	-0,243976841	5	2	2	9,37E-08	0,00033	eQTLGen_	+	Yes	IMR90	0,015926	rs2244608
492	UKBB_CAD	ENSG00000157782	CABP1	12	1,2E+08	1,2E+08	1	500	54	327844	0,96693	0,16679	protein_c	9478	CABP1	0,9944	NA	38	9	0	NA	NA	NA	NA	No	NA	0,00947	rs2244608
493	UKBB_CAD	ENSG00000110917	MLEC	12	1,2E+08	1,2E+08	1	491	40	327438	0,42678	0,33477	protein_c	9761	MLEC	0,47092	1	34	9	4	1,85E-16	0	BIOS_eQT	+	Yes	GM12878	0,00947	rs2244608
494	UKBB_CAD	ENSG00000175970	UNC119B	12	1,2E+08	1,2E+08	1	461	37	327479	0,13963	0,44448	protein_c	84747	UNC119B	0,67733	0,550413245	35	12,07	1	4,62E-07	0,00034	BIOS_eQT	-	Yes	GM12878	0,00947	rs2244608
495	UKBB_CAD	ENSG00000122971	ACADS	12	1,2E+08	1,2E+08	1	448	38	327315	0,16887	0,43295	protein_c	35	ACADS	0,00016	0,905088094	42	13,61	4	2,34E-13	1,41E-05	xQTLServe	+	No	NA	0,008366	rs2244608
496	UKBB_CAD	ENSG00000157837	SPPL3	12	1,2E+08	1,2E+08	-1	914	33	329145	2	0,03215	protein_c	121665	SPPL3	0,90779	0,151809144	219	18,23	9	0	0	xQTLServe	-	No	NA	4,88E-07	rs2244608
497	UKBB_CAD	ENSG00000272214	AC079602	12	1,2E+08	1,2E+08	-1	431	36	326854	5	4,56E-08	protein_c	NA	NA	NA	NA	200	25,6	0	NA	NA	NA	NA	No	NA	2,32E-09	rs2244608
498	UKBB_CAD	ENSG00000135100	HNF1A	12	1,2E+08	1,2E+08	1	507	35	326809	5	9,75E-08	protein_c	6927	HNF1A	0,96966	-0,628781489	207	25,6	3	1,85E-10	0	BIOS_eQT	-	No	NA	2,32E-09	rs2244608
499	UKBB_CAD	ENSG00000157895	C12orf43	12	1,2E+08	1,2E+08	-1	450	40	326657	5	7,12E-07	protein_c	64897	C12orf43	2,56E-07	0,963740862	171	25,6	3	#######	0	BIOS_eQT	-	No	NA	2,32E-09	rs2244608
500	UKBB_CAD	ENSG00000135114	OASL	12	1,2E+08	1,2E+08	-1	422	44	326115	4	4,66E-06	protein_c	8638	OASL	5,07E-12	0,332934746	116	25,6	8	#######	0	BIOS_eQT	+	No	NA	2,32E-09	rs2244608
501	UKBB_CAD	ENSG00000089041	P2RX7	12	1,2E+08	1,2E+08	1	741	39	330269	-0,10068	0,5401	protein_c	5027	P2RX7	3,79E-06	0,326948751	1	0	1	4,69E-07	0,00152	eQTLGen_	+	No	NA	0,023731	rs2244608
502	UKBB_CAD	ENSG00000135124	P2RX4	12	1,2E+08	1,2E+08	1	542	38	329138	0,00393	0,49843	protein_c	5025	P2RX4	2,37E-09	NA	1	0	8	1,53E-35	0	eQTLGen_	+	No	NA	1,09E-06	rs2244608
503	UKBB_CAD	ENSG00000110931	CAMKK2	12	1,2E+08	1,2E+08	-1	508	43	326715	-0,02395	0,50955	protein_c	10645	CAMKK2	0,50348	0,336425978	0	0	8	1,98E-23	0	eQTLGen_	-	No	NA	1,09E-06	rs2244608
504	UKBB_CAD	ENSG00000073060	SCARB1	12	1,3E+08	1,3E+08	-1	727	102	326732	5	5,45E-08	protein_c	949	SCARB1	0,07722	NA	45	10,82	0	NA	NA	NA	NA	No	NA	4,24E-09	rs11057830
505	UKBB_CAD	ENSG00000150991	UBC	12	1,3E+08	1,3E+08	-1	493	60	325848	3	0,00037	protein_c	7316	UBC	0,70416	3	1	7	0	NA	NA	NA	NA	No	NA	0,022661	rs11057830
506	UKBB_CAD	ENSG00000150990	DHX37	12	1,3E+08	1,3E+08	-1	723	69	317625	2	0,05649	protein_c	57647	DHX37	0,97848	1	1	7	0	NA	NA	NA	NA	No	NA	0,022661	rs11057830
507	UKBB_CAD	ENSG00000187498	COL4A1	13	1,1E+08	1,1E+08	-1	1246	144	318780	8	2,62E-14	protein_c	1282	COL4A1	1	-0,456418322	174	11,57	0	NA	NA	NA	NA	No	NA	4,14E-10	rs11617955;rs4773141;rs9515203
508	UKBB_CAD	ENSG00000134871	COL4A2	13	1,1E+08	1,1E+08	1	1652	165	322023	6	2,73E-10	protein_c	1284	COL4A2	2,11E-06	NA	157	12,36	0	NA	NA	NA	NA	No	NA	6,48E-10	rs4773141;rs9515203
509	UKBB_CAD	ENSG00000205476	CCDC85C	14	1E+08	1E+08	-1	644	52	319458	3	0,00325	protein_c	317762	CCDC85C	NA	-2	48	16,09	2	4,42E-16	0	BIOS_eQT	-	No	NA	1,49E-07	rs10139550
510	UKBB_CAD	ENSG00000182218	HHIPL1	14	1E+08	1E+08	1	413	47	325987	5	8,29E-08	protein_c	84439	HHIPL1	0,00214	-1	150	16,09	2	8,01E-11	0	BIOS_eQT	+	No	NA	1,84E-09	rs10139550
511	UKBB_CAD	ENSG00000036530	CYP46A1	14	1E+08	1E+08	1	475	57	327468	4	3,69E-06	protein_c	10858	CYP46A1	0,75469	NA	133	16,09	1	7,46E-07	0,00064	BIOS_eQT	-	No	NA	1,84E-09	rs10139550
512	UKBB_CAD	ENSG00000066629	EML1	14	1E+08	1E+08	1	984	87	326873	1	0,07023	protein_c	2009	EML1	0,99945	0,059551707	36	8	0	NA	NA	NA	NA	No	NA	1,53E-06	rs10139550
513	UKBB_CAD	ENSG00000197119	SLC25A29	14	1E+08	1E+08	-1	289	34	320999	0,85068	0,19747	protein_c	123096	SLC25A29	0,00606	NA	0	0	2	6,36E-12	0	eQTLGen_	+	No	NA	6,95E-06	rs10139550
514	UKBB_CAD	ENSG00000166949	SMAD3	15	6,7E+07	6,7E+07	1	673	74	328433	4	5,46E-05	protein_c	4088	SMAD3	0,86827	-0,091610054	76	20,8	1	3,29E-13	0	eQTLGen_	+	Yes	Aorta:GM	4,81E-12	rs72743461
515	UKBB_CAD	ENSG00000259202	RP11-342	15	6,7E+07	6,7E+07	-1	321	47	328123	5	1,14E-07	protein_c	NA	NA	NA	NA	76	20,8	0	NA	NA	NA	NA	No	NA	4,81E-12	rs72743461
516	UKBB_CAD	ENSG00000103591	AAGAB	15	6,7E+07	6,8E+07	-1	375	32	326548	3	0,0013	protein_c	79719	AAGAB	0,0081	0,196795346	42	8	1	7,27E-69	0	BIOS_eQT	+	Yes	GM12878	6,36E-12	rs72743461
517	UKBB_CAD	ENSG00000103599	IQCH	15	6,8E+07	6,8E+07	1	698	30	325782	-0,65846	0,74488	protein_c	64799	IQCH	2,69E-12	-1	0	0	0	NA	NA	NA	NA	Yes	GM12878	1,25E-09	rs72743461
518	UKBB_CAD	ENSG00000189227	C15orf61	15	6,8E+07	6,8E+07	1	269	16	326068	0,41325	0,33971	protein_c	145853	C15orf61	NA	-0,166715843	0	0	0	NA	NA	NA	NA	Yes	IMR90	0,035052	rs72743461
519	UKBB_CAD	ENSG00000137764	MAP2K5	15	6,8E+07	6,8E+07	1	980	31	327301	0,95522	0,16973	protein_c	5607	MAP2K5	0,47592	NA	0	0	2	3,20E-15	0	eQTLGen_	+	Yes	IMR90	0,035052	rs72743461
520	UKBB_CAD	ENSG00000188779	SKOR1	15	6,8E+07	6,8E+07	1	386	31	327507	1	0,15262	protein_c	390598	SKOR1	NA	-0,128486491	0	0	0	NA	NA	NA	NA	Yes	GM12878	1,25E-09	rs72743461
521	UKBB_CAD	ENSG00000033800	PIAS1	15	6,8E+07	6,8E+07	1	540	22	317894	-1	0,93279	protein_c	8554	PIAS1	0,99702	-0,402983589	0	0	0	NA	NA	NA	NA	Yes	Mesenchy	0,035052	rs72743461
522	UKBB_CAD	ENSG00000167202	TBC1D2B	15	7,8E+07	7,8E+07	-1	772	42	303227	-0,09575	0,53814	protein_c	23102	TBC1D2B	6,51E-05	-0,109963236	6	0,048	0	NA	NA	NA	NA	No	NA	NA	rs4887085;rs7164479
523	UKBB_CAD	ENSG00000140395	WDR61	15	7,9E+07	7,9E+07	-1	422	41	328306	0,91608	0,17981	protein_c	80349	WDR61	0,08977	-0,301818815	0	0	0	NA	NA	NA	NA	Yes	IMR90:Me	7,99E-08	rs4887085
524	UKBB_CAD	ENSG00000166426	CRABP1	15	7,9E+07	7,9E+07	1	315	53	325465	0,70691	0,23981	protein_c	1381	CRABP1	0,1255	NA	0	0	0	NA	NA	NA	NA	Yes	Mesenchy	7,99E-08	rs4887085
525	UKBB_CAD	ENSG00000136381	IREB2	15	7,9E+07	7,9E+07	1	461	37	324051	1	0,11076	protein_c	3658	IREB2	1	0,131406258	10	6,2	6	1,43E-43	0	eQTLGen_	+	Yes	Mesenchy	6,40E-15	rs4887085;rs7164479
526	UKBB_CAD	ENSG00000188266	HYKK	15	7,9E+07	7,9E+07	1	344	21	327723	0,91352	0,18048	protein_c	123688	HYKK	0,00021	-0,450544025	24	6	0	NA	NA	NA	NA	Yes	Mesenchy	7,99E-08	rs7164479;rs4887085
527	UKBB_CAD	ENSG00000268838	AC027228	15	7,9E+07	7,9E+07	1	276	16	327930	0,83693	0,20132	protein_c	NA	NA	NA	NA	25	6	0	NA	NA	NA	NA	No	NA	0,033913	rs7164479;rs4887085
528	UKBB_CAD	ENSG00000041357	PSMA4	15	7,9E+07	7,9E+07	1	292	17	327855	0,9466	0,17192	protein_c	5685	PSMA4	0,62042	0,678454526	29	6	6	4,02E-14	0	eQTLGen_	-	No	NA	6,40E-15	rs7164479;rs4887085
529	UKBB_CAD	ENSG00000169684	CHRNA5	15	7,9E+07	7,9E+07	1	382	18	327733	1	0,12619	protein_c	1138	CHRNA5	2,41E-06	0,42114192	59	7	2	4,14E-11	0	BIOS_eQT	+	Yes	IMR90:Me	4,65E-13	rs7164479;rs4887085
530	UKBB_CAD	ENSG00000080644	CHRNA3	15	7,9E+07	7,9E+07	-1	361	27	326853	3	0,0006	protein_c	1136	CHRNA3	0,00706	0,011573306	99	14,58	4	3,36E-08	4,56E-05	BIOS_eQT	+	Yes	IMR90:Me	6,40E-15	rs7164479;rs4887085
531	UKBB_CAD	ENSG00000117971	CHRNB4	15	7,9E+07	7,9E+07	-1	678	27	308716	7	1,73E-12	protein_c	1143	CHRNB4	0,01863	-0,17732684	449	16,02	0	NA	NA	NA	NA	No	NA	2,60E-14	rs4887085;rs7164479
532	UKBB_CAD	ENSG00000136378	ADAMTS7	15	7,9E+07	7,9E+07	-1	655	20	307234	9	1,72E-18	protein_c	11173	ADAMTS7	2,99E-07	NA	598	16,02	5	1,95E-13	8,94E-18	BIOS_eQT	-	No	NA	6,38E-18	rs7164479;rs4887085
533	UKBB_CAD	ENSG00000185787	MORF4L1	15	7,9E+07	7,9E+07	1	723	28	319765	9	2,56E-18	protein_c	10933	MORF4L1	0,80294	0,191369587	566	17,42	4	1,55E-20	0	BIOS_eQT	+	No	NA	6,38E-18	rs7164479;rs4887085
534	UKBB_CAD	ENSG00000103811	CTSH	15	7,9E+07	7,9E+07	-1	505	34	324061	4	5,60E-05	protein_c	1512	CTSH	3,17E-06	0,548947651	162	17,42	8	#######	0	BIOS_eQT	-	No	NA	6,40E-15	rs7164479;rs4887085
535	UKBB_CAD	ENSG00000058335	RASGRF1	15	7,9E+07	7,9E+07	-1	741	85	323332	2	0,01823	protein_c	5923	RASGRF1	0,99961	-0,134039904	50	13,87	0	NA	NA	NA	NA	No	NA	9,15E-07	rs7164479;rs4887085
536	UKBB_CAD	ENSG00000185043	CIB1	15	9,1E+07	9,1E+07	-1	381	28	321848	-0,60799	0,7284	protein_c	10519	CIB1	0,0161	NA	0	0	1	6,69E-06	0,01909	eQTLGen_	-	No	NA	4,86E-09	rs2071382
537	UKBB_CAD	ENSG00000197299	BLM	15	9,1E+07	9,1E+07	1	589	39	324174	2	0,04141	protein_c	641	BLM	4,14E-11	-0,398747899	26	15,6	0	NA	NA	NA	NA	No	NA	1,41E-09	rs2071382
538	UKBB_CAD	ENSG00000140564	FURIN	15	9,1E+07	9,1E+07	1	392	59	317830	8	3,16E-14	protein_c	5045	FURIN	0,99993	-0,156379719	81	16,83	4	5,00E-13	0	BIOS_eQT	+	No	NA	7,14E-13	rs2071382
539	UKBB_CAD	ENSG00000182511	FES	15	9,1E+07	9,1E+07	1	347	48	315197	7	1,11E-13	protein_c	2242	FES	2,77E-05	NA	83	16,83	5	3.2717000	0	BIOS_eQT	-	No	NA	7,14E-13	rs2071382
540	UKBB_CAD	ENSG00000196547	MAN2A2	15	9,1E+07	9,1E+07	1	404	48	316349	7	1,36E-11	protein_c	4122	MAN2A2	3,61E-09	1	77	16,83	4	1,55E-17	0	eQTLGen_	+	No	NA	7,14E-13	rs2071382
541	UKBB_CAD	ENSG00000140553	UNC45A	15	9,1E+07	9,1E+07	1	458	39	318860	5	1,93E-06	protein_c	55898	UNC45A	9,49E-08	0,063099405	40	16,3	0	NA	NA	NA	NA	No	NA	7,14E-13	rs2071382
542	UKBB_CAD	ENSG00000184508	HDDC3	15	9,1E+07	9,1E+07	-1	382	33	317430	5	9,84E-07	protein_c	374659	HDDC3	0,07183	-0,132810158	39	16,3	2	6,58E-09	4,52E-05	eQTLGen_	-	No	NA	7,14E-13	rs2071382
543	UKBB_CAD	ENSG00000166965	RCCD1	15	9,1E+07	9,2E+07	1	398	30	319285	1	0,09888	protein_c	91433	RCCD1	1,63E-05	2	2	0,529	3	6,37E-15	0	eQTLGen_	+	No	NA	1,33E-10	rs2071382
544	UKBB_CAD	ENSG00000198901	PRC1	15	9,2E+07	9,2E+07	-1	508	33	320801	1	0,14276	protein_c	9055	PRC1	0,01598	0,727684006	2	0,529	0	NA	NA	NA	NA	No	NA	0,045466	rs2071382
545	UKBB_CAD	ENSG00000184056	VPS33B	15	9,2E+07	9,2E+07	-1	563	50	321455	0,97366	0,16511	protein_c	26276	VPS33B	2,91E-09	-0,472003543	0	0	1	1,03E-05	0,00708	BIOS_eQT	+	No	NA	5,45E-07	rs2071382
546	UKBB_CAD	ENSG00000140945	CDH13	16	8,3E+07	8,4E+07	1	7868	392	322200	2	0,01883	protein_c	1012	CDH13	NA	-0,348066372	299	18,38	1	1,44E-05	6,77E-10	GTEx_v7_	+	No	NA	5,14E-09	rs7500448
547	UKBB_CAD	ENSG00000170832	USP32	17	5,8E+07	5,8E+07	-1	293	13	326561	0,40517	0,34268	protein_c	84669	USP32	1	NA	1	0,411	0	NA	NA	NA	NA	Yes	IMR90:Me	1,07E-06	rs8068952
548	UKBB_CAD	ENSG00000141371	C17orf64	17	5,8E+07	5,9E+07	1	100	6	328720	0,36363	0,35807	protein_c	124773	C17orf64	0,0138	-0,401963721	1	0,411	0	NA	NA	NA	NA	Yes	hESC	0,00379	rs8068952
549	UKBB_CAD	ENSG00000062725	APPBP2	17	5,9E+07	5,9E+07	-1	127	15	321140	2	0,03717	protein_c	10513	APPBP2	0,99907	-0,109895205	0	0	0	NA	NA	NA	NA	Yes	IMR90	0,000161	rs8068952
550	UKBB_CAD	ENSG00000267772	RP11-15E	17	5,9E+07	5,9E+07	1	59	8	304383	3	0,00296	protein_c	388406	LINC0199	0,12016	NA	2	8	0	NA	NA	NA	NA	No	NA	NA	rs8068952
551	UKBB_CAD	ENSG00000170836	PPM1D	17	5,9E+07	5,9E+07	1	55	15	278449	3	0,00162	protein_c	8493	PPM1D	0,00117	-0,674472922	2	8	0	NA	NA	NA	NA	Yes	IMR90:Me	0,000161	rs8068952
552	UKBB_CAD	ENSG00000141376	BCAS3	17	5,9E+07	5,9E+07	1	1172	66	321699	5	3,03E-08	protein_c	54828	BCAS3	0,99885	-0,434508261	617	19,88	9	4,55E-14	0	BIOS_eQT	+	No	NA	1,41E-09	rs8068952
553	UKBB_CAD	ENSG00000121068	TBX2	17	5,9E+07	5,9E+07	1	256	36	317469	1	0,13172	protein_c	6909	TBX2	0,96402	NA	0	0	0	NA	NA	NA	NA	Yes	IMR90:Me	1,60E-07	rs8068952
554	UKBB_CAD	ENSG00000187013	C17orf82	17	5,9E+07	5,9E+07	1	237	38	318249	0,9013	0,18371	protein_c	388407	C17orf82	7,35E-05	-0,761191863	0	0	0	NA	NA	NA	NA	Yes	IMR90:Me	1,60E-07	rs8068952
555	UKBB_CAD	ENSG00000121075	TBX4	17	6E+07	6E+07	1	273	44	323378	-0,5996	0,72561	protein_c	9496	TBX4	0,40706	0,012927548	0	0	0	NA	NA	NA	NA	Yes	IMR90:Me	1,60E-07	rs8068952
556	UKBB_CAD	ENSG00000136492	BRIP1	17	6E+07	6E+07	-1	504	26	330127	0,21472	0,41499	protein_c	83990	BRIP1	5,38E-13	-0,711164143	0	0	0	NA	NA	NA	NA	Yes	IMR90:Me	1,60E-07	rs8068952
557	UKBB_CAD	ENSG00000108506	INTS2	17	6E+07	6E+07	-1	269	21	328874	-0,17132	0,56801	protein_c	57508	INTS2	0,99981	-0,307380687	0	0	0	NA	NA	NA	NA	Yes	Mesenchy	1,07E-06	rs8068952
558	UKBB_CAD	ENSG00000108510	MED13	17	6E+07	6E+07	-1	363	21	326426	0,43495	0,3318	protein_c	9969	MED13	1	-2	0	0	0	NA	NA	NA	NA	Yes	Mesenchy	1,60E-07	rs8068952
559	UKBB_CAD	ENSG00000090339	ICAM1	19	1E+07	1E+07	1	346	53	319120	1	0,07576	protein_c	3383	ICAM1	0,1178	-0,071896009	0	0	1	4,94E-06	0,0143	eQTLGen_	-	No	NA	3,69E-14	rs6511720
560	UKBB_CAD	ENSG00000105397	TYK2	19	1E+07	1E+07	-1	384	39	322173	1	0,11883	protein_c	7297	TYK2	0,00431	0,69112764	0	0	1	2,86E-06	0,00834	eQTLGen_	-	No	NA	5,34E-10	rs6511720
561	UKBB_CAD	ENSG00000079999	KEAP1	19	1,1E+07	1,1E+07	-1	356	42	324695	2	0,01206	protein_c	9817	KEAP1	0,25163	-0,049345802	12	18,43	0	NA	NA	NA	NA	No	NA	1,22E-05	rs6511720
562	UKBB_CAD	ENSG00000180739	S1PR5	19	1,1E+07	1,1E+07	-1	361	41	323280	3	0,00109	protein_c	53637	S1PR5	0,05741	-0,598100848	18	18,43	1	3,66E-18	0	BIOS_eQT	-	No	NA	1,22E-05	rs6511720
563	UKBB_CAD	ENSG00000130734	ATG4D	19	1,1E+07	1,1E+07	1	425	33	323751	3	0,00138	protein_c	84971	ATG4D	0,00032	NA	22	18,43	0	NA	NA	NA	NA	No	NA	1,22E-05	rs6511720
564	UKBB_CAD	ENSG00000129347	KRI1	19	1,1E+07	1,1E+07	-1	447	34	324242	3	0,00123	protein_c	65095	KRI1	8,40E-09	-0,684287675	25	18,43	0	NA	NA	NA	NA	No	NA	1,22E-05	rs6511720
565	UKBB_CAD	ENSG00000129355	CDKN2D	19	1,1E+07	1,1E+07	-1	411	26	324939	3	0,00154	protein_c	1032	CDKN2D	0,14729	NA	27	18,43	1	1,97E-20	0	eQTLGen_	-	No	NA	1,22E-05	rs6511720
566	UKBB_CAD	ENSG00000129354	AP1M2	19	1,1E+07	1,1E+07	-1	446	31	325032	4	0,00013	protein_c	10053	AP1M2	0,00135	-0,043748277	30	18,43	1	1,04E-31	0	BIOS_eQT	+	No	NA	1,22E-05	rs6511720
567	UKBB_CAD	ENSG00000129353	SLC44A2	19	1,1E+07	1,1E+07	1	433	35	324110	5	7,75E-07	protein_c	57153	SLC44A2	0,04146	-0,378860929	26	17,33	2	9,78E-35	0	eQTLGen_	+	No	NA	5,34E-10	rs6511720
568	UKBB_CAD	ENSG00000226104	AC011475	19	1,1E+07	1,1E+07	1	247	25	325330	4	8,52E-06	protein_c	NA	NA	NA	NA	14	17,33	0	NA	NA	NA	NA	No	NA	3,26E-05	rs6511720
569	UKBB_CAD	ENSG00000129351	ILF3	19	1,1E+07	1,1E+07	1	378	25	326520	4	5,12E-05	protein_c	3609	ILF3	1	-0,897292942	19	17,33	1	6,56E-07	0,00205	eQTLGen_	-	No	NA	5,34E-10	rs6511720

570	UKBB_CAD	ENSG00000213339	QTRT1	19	1,1E+07	1,1E+07	1	312	22	325629	3	0,00042	protein_c	81890	QTRT1	0,00022	-0,342791499	15	17,33	0	NA	NA	NA	NA	No	NA	3,26E-05	rs6511720
571	UKBB_CAD	ENSG00000079805	DNM2	19	1,1E+07	1,1E+07	1	533	37	324161	5	3,02E-08	protein_c	1785	DNM2	0,99997	-0,475523782	84	11,59	1	7,52E-10	1,32E-05	eQTLGen_	+	No	NA	1,23E-10	rs6511720
572	UKBB_CAD	ENSG00000099203	TMED1	19	1,1E+07	1,1E+07	-1	220	31	322293	6	1,01E-09	protein_c	11018	TMED1	0,00016	-0,379917355	68	11,66	0	NA	NA	NA	NA	No	NA	1,23E-10	rs6511720
573	UKBB_CAD	ENSG00000214212	C19orf38	19	1,1E+07	1,1E+07	1	274	25	322480	6	1,21E-10	protein_c	255809	C19orf38	NA	0,100777924	98	11,66	0	NA	NA	NA	NA	No	NA	1,23E-10	rs6511720
574	UKBB_CAD	ENSG00000142453	CARM1	19	1,1E+07	1,1E+07	1	335	27	322342	7	9,90E-13	protein_c	10498	CARM1	0,99936	NA	161	12,79	3	1,67E-57	0	BIOS_eQT	-	No	NA	1,23E-10	rs6511720
575	UKBB_CAD	ENSG00000130733	YIPF2	19	1,1E+07	1,1E+07	-1	256	21	323221	7	6,93E-12	protein_c	78992	YIPF2	0,0011	NA	127	12,79	1	1,01E-09	6,17E-06	BIOS_eQT	+	No	NA	1,23E-10	rs6511720
576	UKBB_CAD	ENSG00000142444	C19orf52	19	1,1E+07	1,1E+07	1	260	22	323030	7	4,69E-12	protein_c	90580	C19orf52	0,00199	-0,096033732	122	12,79	4	6,43E-51	0	eQTLGen_	+	No	NA	3,69E-14	rs6511720
577	UKBB_CAD	ENSG00000127616	SMARCA4	19	1,1E+07	1,1E+07	1	597	37	324118	9	1,37E-18	protein_c	6597	SMARCA4	1	NA	279	19,62	4	7,38E-67	0	BIOS_eQT	+	No	NA	7,88E-22	rs6511720
578	UKBB_CAD	ENSG00000130164	LDLR	19	1,1E+07	1,1E+07	1	578	52	325912	9	3,79E-19	protein_c	3949	LDLR	2,59E-16	0,415051182	151	19,62	0	NA	NA	NA	NA	No	NA	7,88E-22	rs6511720
579	UKBB_CAD	ENSG00000161888	SPC24	19	1,1E+07	1,1E+07	-1	509	46	325149	7	8,78E-13	protein_c	147841	SPC24	0,00628	4	48	13,8	0	NA	NA	NA	NA	No	NA	7,88E-22	rs6511720
580	UKBB_CAD	ENSG00000197256	KANK2	19	1,1E+07	1,1E+07	-1	550	53	324246	5	4,86E-07	protein_c	25959	KANK2	0,98151	1	7	8	0	NA	NA	NA	NA	No	NA	7,19E-07	rs6511720
581	UKBB_CAD	ENSG00000130158	DOCK6	19	1,1E+07	1,1E+07	-1	592	47	325274	3	0,00125	protein_c	57572	DOCK6	2,83E-19	NA	3	8	0	NA	NA	NA	NA	No	NA	7,19E-07	rs6511720
582	UKBB_CAD	ENSG00000256294	ZNF225	19	4,5E+07	4,5E+07	1	303	26	317548	-0,03782	0,51508	protein_c	7768	ZNF225	3,37E-08	0,547354183	0	0	0	NA	NA	NA	NA	Yes	IMR90	5,27E-10	rs56131196
583	UKBB_CAD	ENSG00000186567	CEACAM1	19	4,5E+07	4,5E+07	1	436	61	325473	3	0,00038	protein_c	56971	CEACAM1	5,78E-07	0,414157593	6	6	0	NA	NA	NA	NA	No	NA	2,86E-07	rs7412
584	UKBB_CAD	ENSG00000213892	CEACAM1	19	4,5E+07	4,5E+07	1	403	60	322014	4	0,00013	protein_c	388551	CEACAM1	0,02958	-0,586640098	16	13,1	0	NA	NA	NA	NA	No	NA	2,86E-07	rs7412;rs56131196
585	UKBB_CAD	ENSG00000069399	BCL3	19	4,5E+07	4,5E+07	1	403	45	319171	3	0,00334	protein_c	602	BCL3	0,86217	NA	32	18,08	0	NA	NA	NA	NA	No	NA	2,86E-07	rs7412;rs56131196
586	UKBB_CAD	ENSG00000142273	CBLC	19	4,5E+07	4,5E+07	1	430	46	318404	3	0,00041	protein_c	23624	CBLC	0,00018	-0,348046092	48	23,1	0	NA	NA	NA	NA	No	NA	1,44E-11	rs7412;rs56131196
587	UKBB_CAD	ENSG00000187244	BCAM	19	4,5E+07	4,5E+07	1	417	46	320920	4	9,47E-05	protein_c	4059	BCAM	0,03685	NA	40	23,1	0	NA	NA	NA	NA	No	NA	1,44E-11	rs56131196;rs7412
588	UKBB_CAD	ENSG00000130202	PVRL2	19	4,5E+07	4,5E+07	1	533	58	320206	7	1,62E-12	protein_c	5819	PVRL2	0,53194	-0,314456223	103	30	6	7,47E-45	0	BIOS_eQT	+	No	NA	2,17E-19	rs7412;rs56131196
589	UKBB_CAD	ENSG00000130204	TOMM40	19	4,5E+07	4,5E+07	1	427	55	321943	7	6,64E-12	protein_c	10452	TOMM40	0,96368	NA	85	30	0	NA	NA	NA	NA	No	NA	2,17E-19	rs7412;rs56131196
590	UKBB_CAD	ENSG00000130203	APOE	19	4,5E+07	4,5E+07	1	393	48	321521	7	2,70E-11	protein_c	348	APOE	0,03453	-0,237445173	76	30	0	NA	NA	NA	NA	No	NA	2,17E-19	rs56131196;rs7412
591	UKBB_CAD	ENSG00000130208	APOC1	19	4,5E+07	4,5E+07	1	370	50	320439	7	4,94E-12	protein_c	341	APOC1	0,05074	-0,105569458	73	30	0	NA	NA	NA	NA	No	NA	2,17E-19	rs7412;rs56131196
592	UKBB_CAD	ENSG00000224916	APOC4-AP	19	4,5E+07	4,5E+07	1	342	34	320074	6	1,76E-08	protein_c	1E+08	APOC4-AP	NA	NA	56	30	0	NA	NA	NA	NA	Yes	IMR90:Me	2,17E-19	rs56131196;rs7412
593	UKBB_CAD	ENSG00000267467	APOC4	19	4,5E+07	4,5E+07	1	342	34	320074	6	1,76E-08	protein_c	346	APOC4	0,00015	0,337274035	56	30	0	NA	NA	NA	NA	Yes	IMR90:Me	2,17E-19	rs56131196;rs7412
594	UKBB_CAD	ENSG00000234906	APOC2	19	4,5E+07	4,5E+07	1	321	31	320087	5	6,01E-07	protein_c	344	APOC2	0,0228	0,388732718	47	30	0	NA	NA	NA	NA	Yes	IMR90:Me	2,17E-19	rs7412;rs56131196
595	UKBB_CAD	ENSG00000267114	CTB-129P	19	4,5E+07	4,5E+07	-1	314	27	320285	5	2,58E-06	protein_c	NA	NA	NA	NA	46	30	0	NA	NA	NA	NA	No	NA	2,17E-19	rs56131196;rs7412
596	UKBB_CAD	ENSG00000104853	CLPTM1	19	4,5E+07	4,5E+07	1	376	33	317505	4	7,57E-05	protein_c	1209	CLPTM1	0,98048	NA	47	30	0	NA	NA	NA	NA	Yes	IMR90:Me	2,17E-19	rs7412;rs56131196
597	UKBB_CAD	ENSG00000104856	RELB	19	4,6E+07	4,6E+07	1	362	38	320551	1	0,09837	protein_c	5971	RELB	0,97356	NA	5	4,03	0	NA	NA	NA	NA	No	NA	0,001133	rs7412
598	UKBB_CAD	ENSG00000104859	CLASRP	19	4,6E+07	4,6E+07	1	348	42	320062	0,5385	0,29512	protein_c	11129	CLASRP	0,96497	-0,085202503	4	4,03	0	NA	NA	NA	NA	No	NA	0,006266	rs7412
599	UKBB_CAD	ENSG00000170684	ZNF296	19	4,6E+07	4,6E+07	-1	283	25	321522	-0,28073	0,61054	protein_c	162979	ZNF296	0,97329	NA	2	3	0	NA	NA	NA	NA	No	NA	0,006266	rs7412
600	UKBB_CAD	ENSG00000142252	GEMIN7	19	4,6E+07	4,6E+07	1	299	24	322821	-0,32307	0,62668	protein_c	79760	GEMIN7	0,3471	-0,101681128	2	3	0	NA	NA	NA	NA	No	NA	0,006266	rs7412;rs56131196
601	UKBB_CAD	ENSG00000007047	MARK4	19	4,6E+07	4,6E+07	1	1046	76	321399	2	0,02923	protein_c	57787	MARK4	0,99993	NA	5	3	0	NA	NA	NA	NA	No	NA	0,002982	rs7412;rs56131196
602	UKBB_CAD	ENSG00000104866	PPP1R37	19	4,6E+07	4,6E+07	1	454	35	321905	-0,21092	0,58353	protein_c	284352	PPP1R37	NA	0,143949947	4	2	0	NA	NA	NA	NA	No	NA	0,002982	rs56131196;rs7412
603	UKBB_CAD	ENSG00000179846	NKPD1	19	4,6E+07	4,6E+07	-1	334	35	319806	-0,39687	0,65427	protein_c	284353	NKPD1	1,89E-09	NA	4	2	0	NA	NA	NA	NA	No	NA	0,002982	rs56131196;rs7412
604	UKBB_CAD	ENSG00000007255	TRAPPC6A	19	4,6E+07	4,6E+07	-1	399	45	316125	-0,69463	0,75636	protein_c	79090	TRAPPC6A	0,00071	-0,133842318	4	2	0	NA	NA	NA	NA	No	NA	0,002982	rs56131196;rs7412
605	UKBB_CAD	ENSG00000189114	BLOC1S3	19	4,6E+07	4,6E+07	1	377	46	314935	-0,64025	0,73899	protein_c	388552	BLOC1S3	0,01843	-0,115773028	4	2	0	NA	NA	NA	NA	No	NA	0,002982	rs56131196;rs7412
606	UKBB_CAD	ENSG00000267545	AC005779	19	4,6E+07	4,6E+07	1	429	54	316549	0,88179	0,18895	protein_c	NA	NA	NA	NA	4	2	0	NA	NA	NA	NA	No	NA	0,002982	rs56131196;rs7412
607	UKBB_CAD	ENSG00000266958	AC006126	19	4,6E+07	4,6E+07	1	459	56	317595	1	0,06756	protein_c	NA	NA	NA	NA	4	2	0	NA	NA	NA	NA	No	NA	0,002982	rs56131196;rs7412
608	UKBB_CAD	ENSG00000130201	EXOC3L2	19	4,6E+07	4,6E+07	-1	447	53	318833	2	0,05843	protein_c	90332	EXOC3L2	0,92556	NA	2	2	0	NA	NA	NA	NA	No	NA	0,031584	rs7412;rs56131196
609	UKBB_CAD	ENSG00000104884	ERCC2	19	4,6E+07	4,6E+07	-1	383	48	324763	1	0,15075	protein_c	2068	ERCC2	3,14E-13	-0,123245341	1	0,308	0	NA	NA	NA	NA	No	NA	NA	rs7412
610	UKBB_CAD	ENSG00000104881	PPP1R13L	19	4,6E+07	4,6E+07	-1	370	46	324402	-0,43434	0,66798	protein_c	10848	PPP1R13L	0,00265	-0,293274669	1	0,308	0	NA	NA	NA	NA	No	NA	NA	rs7412
611	UKBB_CAD	ENSG00000117877	CD3EAP	19	4,6E+07	4,6E+07	1	293	37	323774	-0,44511	0,67188	protein_c	10849	CD3EAP	5,69E-05	2	1	0,308	0	NA	NA	NA	NA	No	NA	NA	rs7412
612	UKBB_CAD	ENSG00000012061	ERCC1	19	4,6E+07	4,6E+07	-1	523	55	324698	1	0,08929	protein_c	2067	ERCC1	0,00029	-0,011078743	2	2	0	NA	NA	NA	NA	No	NA	0,020591	rs7412
613	UKBB_CAD	ENSG00000125740	FOSB	19	4,6E+07	4,6E+07	1	342	42	323948	2	0,03597	protein_c	2354	FOSB	0,85292	-1	1	2	0	NA	NA	NA	NA	No	NA	0,020591	rs7412
614	UKBB_CAD	ENSG00000125744	RTN2	19	4,6E+07	4,6E+07	-1	375	44	324929	2	0,04855	protein_c	6253	RTN2	0,63618	NA	1	2	0	NA	NA	NA	NA	No	NA	0,020591	rs7412
615	UKBB_CAD	ENSG00000213889	PPM1N	19	4,6E+07	4,6E+07	1	386	45	325481	2	0,04843	protein_c	147699	PPM1N	0,0001	-0,142647997	1	2	0	NA	NA	NA	NA	No	NA	0,020591	rs7412
616	UKBB_CAD	ENSG00000125753	VASP	19	4,6E+07	4,6E+07	1	421	47	326027	2	0,04638	protein_c	7408	VASP	0,58367	-0,335330773	1	2	0	NA	NA	NA	NA	No	NA	0,020591	rs7412
617	UKBB_CAD	ENSG00000125741	OPA3	19	4,6E+07	4,6E+07	-1	540	64	323613	2	0,02991	protein_c	80207	OPA3	0,00412	-0,066661608	3	11,52	0	NA	NA	NA	NA	No	NA	0,020591	rs7412;rs56131196
618	UKBB_CAD	ENSG00000177464	GPR4	19	4,6E+07	4,6E+07	-1	338	49	322309	2	0,05609	protein_c	2828	GPR4	0,16599	-0,166011184	2	11,52	0	NA	NA	NA	NA	No	NA	NA	rs56131196
619	UKBB_CAD	ENSG00000125746	EML2	19	4,6E+07	4,6E+07	-1	473	55	324065	2	0,00649	protein_c	24139	EML2	7,58E-10	0,190257397	2	11,52	0	NA	NA	NA	NA	No	NA	NA	rs56131196
620	UKBB_CAD	ENSG00000267757	C19orf83	19	4,6E+07	4,6E+07	1	350	36	324509	3	0,00138	protein_c	1E+08	C19orf83	NA	NA	2	11,52	0	NA	NA	NA	NA	No	NA	NA	rs56131196
621	UKBB_CAD	ENSG00000010310	GIPR	19	4,6E+07	4,6E+07	1	368	33	325260	4	5,83E-05	protein_c	2696	GIPR	2,54E-22	-0,675671019	2	2	0	NA	NA	NA	NA	No	NA	NA	rs56131196
622	UKBB_CAD	ENSG00000125743	SNRPD2	19	4,6E+07	4,6E+07	-1	330	34	326266	4	5,94E-06	protein_c	6633	SNRPD2	0,74752	-0,475801412	1	2	0	NA	NA	NA	NA	No	NA	NA	rs56131196
623	UKBB_CAD	ENSG00000011478	QPCTL	19	4,6E+07	4,6E+07	1	335	35	326870	5	3,35E-06	protein_c	54814	QPCTL	0,00026	-0,638078993	1	2	0	NA	NA	NA	NA	No	NA	NA	rs56131196
624	UKBB_CAD	ENSG00000177051	FBXO46	19	4,6E+07	4,6E+07	-1	268	32	324855	4	3,86E-06	protein_c	23403	FBXO46	0,87644	0,090543664	1	2	0	NA	NA	NA	NA	No	NA	NA	rs56131196
625	UKBB_CAD	ENSG00000237452	AC074212	19	4,6E+07	4,6E+07	1	279	29	324168	5	9,57E-07	protein_c	388553	BHMG1	0,37632	NA	1	2	0	NA	NA	NA	NA	No	NA	NA	rs56131196
626	UKBB_CAD	ENSG00000101109	STK4	20	4,4E+07	4,4E+07	1	506	38	329666	-0,18888	0,57491	protein_c	6789	STK4	0,11831	-1	0	0	2	1,30E-11	0,00078	xQTLServe	NA	No	NA	0,002019	rs7623687
627	UKBB_CAD	ENSG00000243927	MRPS6	21	3,5E+07	3,6E+07	1	415	52	325289	2	0,04593	protein_c	64968	MRPS6	0,13624	NA	7	11,73	4	2,08E-58	0	BIOS_eQT	-	No	NA	2,62E-23	rs28451064;rs7282881
628	UKBB_CAD	ENSG00000272962	SLC5A3	21	3,5E+07	3,5E+07	1	298	35	323271	1	0,10226	protein_c	6526	SLC5A3	NA	-1	6	5	0	NA	NA	NA	NA	No	NA	0,000105	rs28451064;rs7282881
629	UKBB_CAD	ENSG00000198743	SLC5A3	21	3,5E+07	3,5E+07	1	298	35	323271	1	0,10226	protein_c	6526	SLC5A3	0,84946	-1	6	5	4	1,35E-09	6,14E-06	BIOS_eQT	-	No	NA	2,62E-23	rs28451064;rs7282881
630	UKBB_CAD	ENSG00000159197	KCNE2	21	3,6E+07	3,6E+07	1	349	43	324379	4	1,14E-05	protein_c	9992	KCNE2	0,00102	0,02614939	37	16,56	0	NA	NA	NA	NA	No	NA	1,21E-07	rs28451064;rs7282881
631	UKBB_CAD	ENSG00000205670	SMIM11	21	3,6E+07	3,6E+07	1	447	51	324903	3	0,00465	protein_c	54065	SMIM11	0,55668	-0,46579369	29	16,56	0	NA	NA	NA	NA	No	NA	1,24E-07	rs28451064;rs7282881
632	UKBB_CAD	ENSG00000222018	C21orf140	21	3,6E+07	3,6E+07	-1	327	32	323558	0,73823	0,23019	protein_c	1E+08	C21orf140	NA	NA	1	0,872	0	NA	NA	NA	NA	No	NA	0,021368	rs7282881
633	UKBB_CAD	ENSG00000184674	GSTT1	22	2,4E+07	2,4E+07	-1	170	22	277219	3	0,0036	protein_c	2952	GSTT1	0,00167	NA	3	4	0	NA	NA	NA	NA	No	NA	NA	rs180803
634	UKBB_CAD	ENSG00000099991	CABIN1	22	2,4E+07	2,5E+07	1	500	33	313179	3	0,00339	protein_c	23523	CABIN1	8,54E-08	-0,916075902	5	4	0	NA	NA	NA	NA	No	NA	NA	rs180803
635	UKBB_CAD	ENSG00000099994	SUSD2	22	2,5E+07	2,5E+07	1	297	26	321971	3	0,00428	protein_c	56241	SUSD2	1,33E-10	0,366055545	5	7	2	1,24E-08	6,42E-05	eQTLGen_	+	No	NA	4,94E-08	rs180803
636	UKBB_CAD	ENSG00000099998	GGT5	22	2,5E+07	2,5E+07	-1	364	38	318075	4	2,08E-05	protein_c	2687	GGT5	1,39E-05	-0,269738606	51	12,14	0	NA	NA	NA	NA	No	NA	7,09E-10	rs180803
637	UKBB_CAD	ENSG00000100014	SPECC1L	22	2,5E+07	2,5E+07	1	653	31	316735	3	0,00042	protein_c	23384	SPECC1L	0,9934	-1	110	17,37	0	NA	NA	NA	NA	No	NA	7,09E-10	rs180803
638	UKBB_CAD	ENSG00000258555	SPECC1L-A	22	2,5E+07	2,5E+07	1	729	32	317430	3	0,00025	protein_c	1E+08	SPECC1L-A	NA	NA	111	17,37	0	NA	NA	NA	NA	No	NA	7,09E-10	rs180803
639	UKBB_CAD	ENSG00000128271	ADORA2A	22	2,5E+07	2,5E+07	1	319	20	319944	2	0,01229	protein_c	135	ADORA2A	0,33162	-1	19	9	0	NA	NA	NA	NA	No	NA	3,07E-07	rs180803
640	UKBB_CAD	ENSG00000100024	UPB1	22	2,5E+07	2,5E+07	1	424	29	314030	2	0,02113	protein_c	51733	UPB1	1,85E-07	-0,057578259	22	12,49	0	NA	NA	NA	NA	No	NA	3,07E-07	rs180803
641	UKBB_CAD	ENSG00000138867	GUCD1	22	2,5E+07	2,5E+07	-1	307	27	309029	2	0,04929	protein_c	83606	GUCD1	0,01669	-0,251793531	20	12,49	0	NA	NA	NA	NA	No	NA	2,43E-06	rs180803
642	UKBB_CAD	ENSG00000100028	SNRPD3	22	2,5E+07	2,5E+07	1	311	27	298642	0,71526	0,23722	protein_c	6634	SNRPD3	0,87785	-0,828887166	20	12,49	0	NA	NA	NA	NA	No	NA	2,43E-06	rs180803
643	UKBB_CAD	ENSG00000100031	GGT1	22	2,5E+07	2,5E+07	1	311	32	292501	0,53957	0,29475	protein_c	2678	GGT1	0,67697	-0,203276478	17	12,49	0	NA	NA	NA	NA	No	NA	4,93E-05	rs180803
644	UKBB_CAD	ENSG00000178026	FAM211B	22	2,5E+07	2,5E+07	-1	228	29	295717	0,6741	0,25012	protein_c	388886	FAM211B	1,90E-11	NA	17	12,49	0	NA	NA	NA	NA	No	NA	4,93E-05	rs180803

Online Table III

Enrichment

**Description**	**genepattern1**	**genepattern2**	**genepattern3**	**genepattern4**	**genepattern5**	**genepattern6**	**genepattern7**	**genepattern8**	**genepattern9**	**genepattern10**	**genepattern11**	**genepattern12**	**genepattern13**	**genepattern14**	**genepattern15**
ArteryBlood.withMAGMA	0,651284768	1,838505663	2,462043496	1,773695388	1,778613946	0,53426414	1,223686658	2,354929784	1,195013448	1,454183524	1,846782699	1,227824132	0,558393019	2,509431	1,211169
p value	0,788373333	0,175973333	0,016506667	0,153746667	0,123733333	0,84664	0,388746667	0,006173333	0,407066667	0,23296	0,1076	0,444573333	0,835666667	0,015373333	0,45072

total enrichment p value 2,67E-05

Online Table III

Report

	total.FUMA.genes	possible.FUMA.genes	FUMA.genes.after.magma.filtering	FUMA.differentially.expressed
ArteryBlood.withMAGMA	644	612	317	74

Online Table III

GWAS_DEG

					DEG results											FUMA & MAGMA results							Known GWAS loci					
Index	Gene	ensg	entrezID	chr	start	end	strand	genepattern	enriched	IndSigSNPs	pLI	ncRVIS	maxCADD	RDB	eqtlMapSNPs	EqtlMapminP	eqtlDirection	eqtlMapts	ciMa	ciMapts	MAGMA_P	Lead SNP	Gene(s) at locus	ChrBP	Alleles	MAF	gwasP	func
1	*SWAP70*	ENSG00000133789	23075	11	9685624	9774538	+	1	no	rs10840293	0,35	-0,39	18,57	6	18	6,4098E-242	-	BIOS_eQTL_geneLevel:GTEx_v7_Artery_Tibial:eQTLGen_cis_	Yes	GM12878:IM	1,4297E-06	rs1084029	SWAP70	11:975119	A/G	0,4185	6,88E-09	intronic
2	*KCNH2*	ENSG00000055118	3757	7	150642049	150675403	-	2	no	rs3918226	1,00	NA	12,19	2b	0	NA	NA	NA	No	NA	0,0030527	rs3918226	NOS3	7:1506901	T/C	0,09841	1,58E-12	intronic
3	*SLC2A6*	ENSG00000160326	11182	9	136336217	136344259	-	2	no	rs507666	0,00	-0,03	10,16	4	1	6,93223E-05	-	BIOS_eQTL_geneLevel	No	NA	0,00091244	rs579459	ABO, SURF6, GBGT1	9:1361493	A/G	0,1859	1,34E-12	ncRNA_intronic
4	*BCL3*	ENSG00000069399	602	19	45250962	45263301	+	2	no	rs7412;rs56131196	0,86	NA	18,08	4	0	NA	NA	NA	No	NA	0,0033431	rs2075650	APOE, APOC1, TOMM40, PVRL2, COTL1	19:4541207	C/T	0,06262	2,17E-19	exonic
5	*FOSB*	ENSG00000125740	2354	19	45971253	45978437	+	2	no	rs7412;rs56131196	0,85	-1,21	2,07	4	0	NA	NA	NA	No	NA	0,03597	rs2075650	APOE, APOC1, TOMM40, PVRL2, COTL1	19:4541207	C/T	0,06262	2,17E-19	exonic
6	***SHE***	ENSG00000169291	126669	1	154442248	154474589	-	**3**	**yes**	rs6689306	0,72	-1,33	14,09	5	1	2,02966E-07	+	BIOS_eQTL_geneLevel:eQTLGen_cis_eQTLs	No	NA	2,1711E-06	**rs4845625**	**IL6R, AQP10, ATP8B2, CHTOP, UBAP2L**	1:1543959	A/G	0,4423	1,46E-09	intronic
7	***KCNN3***	ENSG00000143603	3782	1	154669931	154842756	-	**3**	**yes**	rs6689306	0,86	-0,63	3,70	5	0	NA	NA	NA	Yes	IMR90:Mese	0,014598	**rs4845625**	**IL6R, AQP10, ATP8B2, CHTOP, UBAP2L**	1:1543959	A/G	0,4423	1,46E-09	intronic
8	***VAMP5***	ENSG00000168899	10791	2	85811531	85820535	+	**3**	**yes**	rs7568458	0,05	0,55	20,90	1b	13	3.27170000000001E-310	-	BIOS_eQTL_geneLevel:GTEx_v7_Whole_Blood:eQTLGen_cis	Yes	IMR90:Mese	8,0479E-14	**rs1561198**	**VAMP5, VAMP8, GGCX**	2:8578817	A/T	0,4781	2,39E-13	intronic
9	***SEMA3F***	ENSG00000001617	6405	3	50192478	50226508	+	**3**	**yes**	rs7623687	1,00	NA	4,39	4	0	NA	NA	NA	No	NA	0,0047131	**rs7623687**	**RHOA, AMT, TCTA, CDHRA, KLHDC8B, and oth**	3:4944856	A/C	0,1491	3,72E-09	intronic
10	***HDAC9***	ENSG00000048052	9734	7	18126572	19042039	+	**3**	**yes**	rs2107595	1,00	-0,12	19,01	2b	0	NA	NA	NA	No	NA	0,027634	**rs2023938**	**HDAC9**	7:1904938	A/G	0,168	3,41E-13	intergenic
11	***GIMAP1***	ENSG00000213203	170575	7	150413645	150421372	+	**3**	**yes**	rs3918226	0,84	0,63	8,80	2b	0	NA	NA	NA	No	NA	0,036739	**rs3918226**	**NOS3**	7:1506901	T/C	0,09841	1,58E-12	intronic
12	***NOS3***	ENSG00000164867	4846	7	150688083	150711676	+	**3**	**yes**	rs3918226	0,00	0,13	12,19	2b	0	NA	NA	NA	No	NA	0,016994	**rs3918226**	**NOS3**	7:1506901	T/C	0,09841	1,58E-12	intronic
13	***DOCK6***	ENSG00000130158	57572	19	11309971	11373157	-	**3**	**yes**	rs6511720	0,00	NA	8,34	4	0	NA	NA	NA	No	NA	0,0012527	**rs1122608**	**LDLR, SMARCA4**	19:1120230	G/T	0,1103	7,88E-22	intronic
14	*NOP58*	ENSG00000055044	51602	2	203130439	203168389	+	4	no	rs114123510	0,51	0,12	13,15	7	17	8,0771E-27	-	eQTLGen_cis_eQTLs	Yes	IMR90:Mese	0,035498	rs6725887	WDR12, CARF, FAM117B, ICA1L, NBEAL1	2:2038312	A/T	0,1302	2,88E-19	intronic
15	*UBA7*	ENSG00000182179	7318	3	49842640	49851379	-	4	no	rs7623687	0,00	-0,32	16,53	4	20	3.27170000000001E-310	-	BIOS_eQTL_geneLevel:GTEx_v7_Whole_Blood:GTEx_v7_Art	No	NA	0,000083035	rs7623687	RHOA, AMT, TCTA, CDHRA, KLHDC8B, and othe	3:4944856	A/C	0,1491	3,72E-09	intronic
16	*ERP29*	ENSG00000089248	10961	12	112451120	112461255	+	4	no	rs10774625	0,47	-0,62	16,24	5	22	1,9341E-53	+	BIOS_eQTL_geneLevel:eQTLGen_cis_eQTLs	No	NA	0,000022903	rs3184504	SH2B3, FLJ21127, ATXN2, and others	12:1119102	A/G	0,4771	9,22E-14	intronic
17	*ILF3*	ENSG00000129351	3609	19	10764937	10803093	+	4	no	rs6511720	1,00	-0,90	17,33	4	1	6,5585E-07	-	eQTLGen_cis_eQTLs	No	NA	0,000051171	rs1122608	LDLR, SMARCA4	19:1120230	G/T	0,1103	7,88E-22	intronic
18	*SNRPD2*	ENSG00000125743	6633	19	46190712	46195827	-	4	no	rs7412;rs56131196	0,75	-0,48	1,68	4	0	NA	NA	NA	No	NA	5,9392E-06	rs2075650	APOE, APOC1, TOMM40, PVRL2, COTL1	19:4541207	C/T	0,06262	2,17E-19	exonic
19	*SFTPB*	ENSG00000168878	6439	2	85884437	85895864	-	5	no	rs7568458	0,04	-0,14	9,87	1b	0	NA	NA	NA	No	NA	0,00019003	rs1561198	VAMP5, VAMP8, GGCX	2:8578817	A/T	0,4781	2,39E-13	intronic
20	*CARF*	ENSG00000138380	79800	2	203776937	203851786	+	5	no	rs114123510	0,00	-1,33	18,32	7	23	7,31663E-12	-	BIOS_eQTL_geneLevel:GTEx_v7_Whole_Blood:GTEx_v7_Art	No	NA	3,2155E-16	rs6725887	WDR12, CARF, FAM117B, ICA1L, NBEAL1	2:2038312	A/T	0,1302	2,88E-19	intronic
21	*CYP20A1*	ENSG00000119004	57404	2	204103663	204163009	+	5	no	rs114123510	0,00	-0,36	22,90	7	0	NA	NA	NA	No	NA	2,6319E-06	rs6725887	WDR12, CARF, FAM117B, ICA1L, NBEAL1	2:2038312	A/T	0,1302	2,88E-19	intronic
22	*NICN1*	ENSG00000145029	84276	3	49460379	49466759	-	5	no	rs7623687	0,26	-1,09	17,41	4	21	2,5934E-116	-	GTEx_v7_Artery_Aorta:GTEx_v7_Artery_Tibial:eQTLGen_cis_	No	NA	1,0106E-06	rs7623687	RHOA, AMT, TCTA, CDHRA, KLHDC8B, and othe	3:4944856	A/C	0,1491	3,72E-09	intronic
23	*OPA3*	ENSG00000125741	80207	19	46030685	46105470	-	5	no	rs7412;rs56131196	0,00	-0,07	11,52	4	0	NA	NA	NA	No	NA	0,02991	rs2075650	APOE, APOC1, TOMM40, PVRL2, COTL1	19:4541207	C/T	0,06262	2,17E-19	exonic
24	*QPCTL*	ENSG00000011478	54814	19	46195741	46207247	+	5	no	rs7412;rs56131196	0,00	-0,64	1,68	4	0	NA	NA	NA	No	NA	0,000003346	rs2075650	APOE, APOC1, TOMM40, PVRL2, COTL1	19:4541207	C/T	0,06262	2,17E-19	exonic
25	*MORF4L1*	ENSG00000185787	10933	15	79102829	79190475	+	6	no	rs4887085;rs7164479	0,80	0,19	17,42	5	4	1,55161E-20	+	BIOS_eQTL_geneLevel:GTEx_v7_Artery_Tibial	No	NA	2,5617E-18	rs3825807	ADAMTS7	15:791230	T/C	0,4235	6,38E-18	intronic
26	*ADAR*	ENSG00000160710	103	1	154554538	154600475	-	7	no	rs6689306	0,91	-0,57	14,09	5	0	NA	NA	NA	Yes	Mesenchym	0,00061689	rs4845625	IL6R, AQP10, ATP8B2, CHTOP, UBAP2L	1:1543959	A/G	0,4423	1,46E-09	intronic
27	*GNLY*	ENSG00000115523	10578	2	85912298	85925977	+	7	no	rs7568458	0,00	1,07	9,87	1b	12	7,6147E-102	+	BIOS_eQTL_geneLevel:eQTLGen_cis_eQTLs	Yes	GM12878:IM	0,0070772	rs1561198	VAMP5, VAMP8, GGCX	2:8578817	A/T	0,4781	2,39E-13	intronic
28	*IGF2R*	ENSG00000197081	3482	6	160390131	160534539	+	7	no	rs624249;rs10455872;rs9365196;rs9457995;rs186696265	1,00	-0,31	13,65	6	0	NA	NA	NA	No	NA	3,4558E-10	rs4252120	PLG, LPAL2	6:1610101	G/A	0,07356	1,71E-49	intronic
29	*S1PR5*	ENSG00000180739	53637	19	10623623	10628607	-	7	no	rs6511720	0,06	-0,60	18,43	4	1	3,6629E-18	-	BIOS_eQTL_geneLevel:eQTLGen_cis_eQTLs	No	NA	0,0010933	rs1122608	LDLR, SMARCA4	19:1120230	G/T	0,1103	7,88E-22	intronic
30	*CABIN1*	ENSG00000099991	23523	22	24407642	24574596	+	7	no	rs180803	0,00	-0,92	4,39	NA	0	NA	NA	NA	No	NA	0,0033948	rs180803	ADORA2A	22:246588	G/T	0,01392	7,09E-10	ncRNA_intronic
31	***AMPD2***	ENSG00000116337	271	1	110158726	110174673	+	**8**	**yes**	rs7528419	0,01	-0,43	8,03	3a	0	NA	NA	NA	No	NA	0,003094	**rs599839**	**SORT1, PSCR1, CELSR2**	1:1098171	A/G	0,2127	3,77E-27	UTR3
32	***CTSS***	ENSG00000163131	1520	1	150702672	150738433	-	**8**	**yes**	rs11810571	0,63	-0,39	0,00	3a	3	5,8532E-07	-	eQTLGen_cis_eQTLs	No	NA	0,0002812	**rs1181057**	**TDRKH, RP11-98D18.9**	1:1517623	G/C	0,1402	2,21E-08	intronic
33	***IL6R***	ENSG00000160712	3570	1	154377669	154441926	+	**8**	**yes**	rs6689306	0,05	-0,79	13,41	5	2	3,681E-13	+	GTEx_v7_Whole_Blood:GTEx_v7_Artery_Tibial:eQTLGen_cis	No	NA	0,000001895	**rs4845625**	**IL6R, AQP10, ATP8B2, CHTOP, UBAP2L**	1:1543959	A/G	0,4423	1,46E-09	intronic
34	***CAPG***	ENSG00000042493	822	2	85621871	85645555	-	**8**	**yes**	rs7568458	0,00	-0,25	7,92	1b	4	7,6752E-22	+	BIOS_eQTL_geneLevel:eQTLGen_cis_eQTLs	Yes	IMR90:Mese	0,0046259	**rs1561198**	**VAMP5, VAMP8, GGCX**	2:8578817	A/T	0,4781	2,39E-13	intronic
35	***GPX1***	ENSG00000233276	2876	3	49394609	49396033	-	**8**	**yes**	rs7623687	0,01	-0,62	19,43	4	20	0	+	xQTLServer_eQTLs:BIOS_eQTL_geneLevel:eQTLGen_cis_eQT	No	NA	0,000029783	**rs7623687**	**RHOA, AMT, TCTA, CDHRA, KLHDC8B, and oth**	3:4944856	A/C	0,1491	3,72E-09	intronic
36	***GNAI2***	ENSG00000114353	2771	3	50263724	50296787	+	**8**	**yes**	rs7623687	0,86	-0,97	8,05	4	0	NA	NA	NA	No	NA	0,023877	**rs7623687**	**RHOA, AMT, TCTA, CDHRA, KLHDC8B, and oth**	3:4944856	A/C	0,1491	3,72E-09	intronic
37	***TRIB1***	ENSG00000173334	10221	8	126442563	126450647	+	**8**	**yes**	rs2954029	0,61	-0,46	18,82	7	0	NA	NA	NA	No	NA	4,5272E-06	**rs2954029**	**TRIB1**	8:1264909	A/T	0,4483	5,24E-13	ncRNA_intronic
38	***SH2B3***	ENSG00000111252	10019	12	111843752	111889427	+	**8**	**yes**	rs10774625	0,01	-0,24	17,18	5	31	7,3125E-124	+	BIOS_eQTL_geneLevel:eQTLGen_cis_eQTLs	No	NA	3,9734E-08	**rs3184504**	**SH2B3, FLJ21127, ATXN2, and others**	12:1119102	A/G	0,4771	9,22E-14	intronic
39	***CTSH***	ENSG00000103811	1512	15	79213400	79241916	-	**8**	**yes**	rs4887085;rs7164479	0,00	0,55	17,42	5	8	2,8975E-206	-	BIOS_eQTL_geneLevel:eQTLGen_cis_eQTLs	No	NA	0,00005596	**rs3825807**	**ADAMTS7**	15:791230	T/C	0,4235	6,38E-18	intronic
40	***FES***	ENSG00000182511	2242	15	91426925	91439006	+	**8**	**yes**	rs2071382	0,00	NA	16,83	5	5	3.27170000000001E-310	-	BIOS_eQTL_geneLevel:GTEx_v7_Whole_Blood:GTEx_v7_Art	No	NA	1,114E-13	**rs1751484**	**FURIN, FES**	15:9142819	T/C	0,4712	7,14E-13	intronic
41	***C19orf38***	ENSG00000214212	255809	19	10947251	10980466	+	**8**	**yes**	rs6511720	NA	0,10	11,66	4	0	NA	NA	NA	No	NA	1,213E-10	**rs1122608**	**LDLR, SMARCA4**	19:1120230	G/T	0,1103	7,88E-22	intronic
42	***VASP***	ENSG00000125753	7408	19	46009837	46030241	+	**8**	**yes**	rs7412;rs56131196	0,58	-0,34	2,07	4	0	NA	NA	NA	No	NA	0,04638	**rs2075650**	**APOE, APOC1, TOMM40, PVRL2, COTL1**	19:4541207	C/T	0,06262	2,17E-19	exonic
43	*GGCX*	ENSG00000115486	2677	2	85771846	85788670	-	9	no	rs7568458	0,00	-0,05	20,90	1b	13	3.27170000000001E-310	+	xQTLServer_eQTLs:BIOS_eQTL_geneLevel:GTEx_v7_Cells_EE	Yes	GM12878:IM	2,745E-13	rs1561198	VAMP5, VAMP8, GGCX	2:8578817	A/T	0,4781	2,39E-13	intronic
44	*ICA1L*	ENSG00000163596	130026	2	203640690	203736708	-	9	no	rs114123510	0,44	-0,76	18,32	7	24	2,4885E-163	+	xQTLServer_eQTLs:BIOS_eQTL_geneLevel:GTEx_v7_Artery_	No	NA	6,5238E-17	rs6725887	WDR12, CARF, FAM117B, ICA1L, NBEAL1	2:2038312	A/T	0,1302	2,88E-19	intronic
45	*ABI2*	ENSG00000138443	10152	2	204192942	204312446	+	9	no	rs114123510	0,95	-2,08	22,90	7	0	NA	NA	NA	No	NA	2,1348E-06	rs6725887	WDR12, CARF, FAM117B, ICA1L, NBEAL1	2:2038312	A/T	0,1302	2,88E-19	intronic
46	*FGF5*	ENSG00000138675	2250	4	81187753	81257834	+	9	no	rs10857147	0,00	0,71	20,50	5	0	NA	NA	NA	No	NA	0,00043691	rs1085714	PRDM8, FGF5	4:8118107	T/A	0,2644	8,96E-09	intergenic
47	*MTAP*	ENSG00000099810	4507	9	21802542	21931646	+	9	no	rs140458508;rs36228834;rs3217978;rs2891168	0,00	1,59	22,60	4	1	8,4803E-08	+	eQTLGen_cis_eQTLs	Yes	GM12878:IM	2,1716E-08	rs1333049	ANRIL, CDKN2B-AS	9:2209861	G/A	0,492	1,3E-101	ncRNA_intronic
48	*CTSK*	ENSG00000143387	1513	1	150768684	150780799	-	10	no	rs11810571	0,00	-0,59	0,00	3a	5	9,8816E-17	-	eQTLGen_cis_eQTLs	No	NA	0,00031591	rs1181057	TDRKH, RP11-98D18.9	1:1517623	G/C	0,1402	2,21E-08	intronic
49	*FN1*	ENSG00000115414	2335	2	216225163	216300895	-	10	no	rs1250229	0,06	0,19	14,47	5	2	1,3284E-16	+	BIOS_eQTL_geneLevel:eQTLGen_cis_eQTLs	No	NA	0,000059706	rs1250229	FN1, ATIC, LOC102724849, ABCA12, LINC00607	2:2163043	T/C	0,2316	1,85E-13	intergenic
50	*DAG1*	ENSG00000173402	1605	3	49506146	49573048	+	10	no	rs7623687	0,94	-0,84	19,64	4	18	4,4608E-26	-	BIOS_eQTL_geneLevel:GTEx_v7_Artery_Aorta:eQTLGen_cis_	No	NA	5,9207E-07	rs7623687	RHOA, AMT, TCTA, CDHRA, KLHDC8B, and othe	3:4944856	A/C	0,1491	3,72E-09	intronic
51	*MRAS*	ENSG00000158186	22808	3	138066539	138124375	+	10	no	rs139016349	0,86	-0,41	18,85	NA	18	4,97819E-46	+	BIOS_eQTL_geneLevel:GTEx_v7_Artery_Aorta:GTEx_v7_Arte	Yes	IMR90:Tropl	2,4978E-11	rs2306374	MRAS, CEP70	3:1380991	TTTC/T	0,1531	9,2E-12	intronic
52	*CDH13*	ENSG00000140945	1012	16	82660408	83830204	+	10	no	rs7500448	NA	-0,35	18,38	5	1	1,43908E-05	+	GTEx_v7_Artery_Aorta	No	NA	0,018831	rs7500448	CDH13	16:830457	A/G	0,2435	5,14E-09	intronic
53	*GSTT1*	ENSG00000184674	2952	22	24376133	24384680	-	10	no	rs180803	0,00	NA	4,39	NA	0	NA	NA	NA	No	NA	0,0035982	rs180803	ADORA2A	22:246588	G/T	0,01392	7,09E-10	ncRNA_intronic
54	*SORT1*	ENSG00000134243	6272	1	109852192	109940573	-	11	no	rs7528419	1,00	-1,35	25,50	3a	4	2,3725E-09	-	eQTLGen_cis_eQTLs	Yes	Aorta:GM12	7,2514E-14	rs599839	SORT1, PSCR1, CELSR2	1:1098171	A/G	0,2127	3,77E-27	UTR3
55	*RNF181*	ENSG00000168894	51255	2	85822848	85824736	+	11	no	rs7568458	0,00	-0,11	20,90	1b	6	4,38643E-11	-	BIOS_eQTL_geneLevel:GTEx_v7_Whole_Blood	Yes	IMR90:Mese	9,8474E-12	rs1561198	VAMP5, VAMP8, GGCX	2:8578817	A/T	0,4781	2,39E-13	intronic
56	*TRIM8*	ENSG00000171206	81603	10	104404253	104418164	+	11	no	rs11191416	0,99	-0,52	20,50	6	8	2,3966E-40	+	BIOS_eQTL_geneLevel:eQTLGen_cis_eQTLs	Yes	IMR90	0,018777	rs1241340	CYP17A1, CNNM2, NT5C2	10:1046049	T/G	0,09245	5,58E-09	ncRNA_intronic
57	*ALDH2*	ENSG00000111275	217	12	112204691	112247782	+	11	no	rs10774625	0,00	NA	16,28	5	23	6,4905E-118	-	xQTLServer_eQTLs:BIOS_eQTL_geneLevel:GTEx_v7_Artery_	Yes	IMR90:Mese	0,000041886	rs3184504	SH2B3, FLJ21127, ATXN2, and others	12:1119102	A/G	0,4771	9,22E-14	intronic
58	*APOE*	ENSG00000130203	348	19	45409011	45412650	+	11	no	rs7412;rs56131196	0,03	-0,24	30,00	4	0	NA	NA	NA	No	NA	2,7039E-11	rs2075650	APOE, APOC1, TOMM40, PVRL2, COTL1	19:4541207	C/T	0,06262	2,17E-19	exonic
59	*APOC1*	ENSG00000130208	341	19	45417504	45422606	+	11	no	rs7412;rs56131196	0,05	-0,11	30,00	4	0	NA	NA	NA	No	NA	4,9393E-12	rs2075650	APOE, APOC1, TOMM40, PVRL2, COTL1	19:4541207	C/T	0,06262	2,17E-19	exonic
60	*ZFP36L2*	ENSG00000152518	678	2	43449541	43453748	-	12	no	rs4299376;rs76866386	0,46	-0,44	11,39	7	0	NA	NA	NA	No	NA	4,2748E-06	rs6544713	ABCG5, ABCG8	2:4407257	G/T	0,3072	5,65E-10	intronic
61	*RHOA*	ENSG00000067560	387	3	49396578	49450431	-	12	no	rs7623687	0,80	-0,43	19,43	4	4	9,07444E-05	+	GTEx_v7_Whole_Blood	No	NA	7,4027E-06	rs7623687	RHOA, AMT, TCTA, CDHRA, KLHDC8B, and othe	3:4944856	A/C	0,1491	3,72E-09	intronic
62	*ACTR1A*	ENSG00000138107	10121	10	104238986	104262482	-	12	no	rs11191416	0,94	-0,27	16,96	6	11	1,2182E-24	-	eQTLGen_cis_eQTLs	Yes	GM12878:IM	0,042623	rs1241340	CYP17A1, CNNM2, NT5C2	10:1046049	T/G	0,09245	5,58E-09	ncRNA_intronic
63	*SLC44A2*	ENSG00000129353	57153	19	10713133	10755235	+	13	no	rs6511720	0,04	-0,38	17,33	4	2	9,7798E-35	+	eQTLGen_cis_eQTLs	No	NA	7,7469E-07	rs1122608	LDLR, SMARCA4	19:1120230	G/T	0,1103	7,88E-22	intronic
64	***ITGB5***	ENSG00000082781	3693	3	124480795	124620265	-	**14**	**yes**	rs62265630	0,00	1,25	20,70	6	1	8,4594E-25	-	eQTLGen_cis_eQTLs	No	NA	0,01193	**rs6226563**	**.**	3:1244752	G/T	0,1511	1E-08	intergenic
65	***ARHGEF2***	ENSG00000114790	26084	3	153838792	153975616	+	**14**	**yes**	rs12493885	0,00	-0,08	24,10	4	10	1,02487E-18	+	BIOS_eQTL_geneLevel:GTEx_v7_Artery_Aorta:GTEx_v7_Arte	No	NA	0,000010909	**rs1249388**	**ARHGEF26**	3:1538398	C/G	0,1262	3,29E-08	exonic
66	***CXCL12***	ENSG00000107562	6387	10	44793038	44881941	-	**14**	**yes**	rs1870634;rs1632484	0,12	NA	21,30	NA	0	NA	NA	NA	Yes	IMR90:Mese	6,316E-10	**rs1746048**	**CXCL12**	10:444808	G/T	0,334	5,51E-13	intergenic
67	***PTPN11***	ENSG00000179295	5781	12	112856155	112947717	+	**14**	**yes**	rs10774625	1,00	-2,25	15,46	5	19	4,2821E-25	-	BIOS_eQTL_geneLevel:eQTLGen_cis_eQTLs	No	NA	0,00032806	**rs3184504**	**SH2B3, FLJ21127, ATXN2, and others**	12:1119102	A/G	0,4771	9,22E-14	intronic
68	***COL4A1***	ENSG00000187498	1282	13	110801318	110959496	-	**14**	**yes**	rs11617955;rs4773141;rs9515203	1,00	-0,46	11,57	5	0	NA	NA	NA	No	NA	2,6152E-14	**rs4773144**	**COL4A1, COL4A2**	13:110818	T/A	0,1233	4,14E-10	intronic
69	***COL4A2***	ENSG00000134871	1284	13	110958159	111165374	+	**14**	**yes**	rs11617955;rs4773141;rs9515203	0,00	NA	12,36	5	0	NA	NA	NA	No	NA	2,7301E-10	**rs4773144**	**COL4A1, COL4A2**	13:110818	T/A	0,1233	4,14E-10	intronic
70	***KANK2***	ENSG00000197256	25959	19	11274943	11308467	-	**14**	**yes**	rs6511720	0,98	1,11	8,34	4	0	NA	NA	NA	No	NA	4,8566E-07	**rs1122608**	**LDLR, SMARCA4**	19:1120230	G/T	0,1103	7,88E-22	intronic
71	***GGT5***	ENSG00000099998	2687	22	24615622	24641110	-	**14**	**yes**	rs180803	0,00	-0,27	12,14	NA	0	NA	NA	NA	No	NA	0,000020803	**rs180803**	**ADORA2A**	22:246588	G/T	0,01392	7,09E-10	ncRNA_intronic
72	*VAMP8*	ENSG00000118640	8673	2	85788685	85809154	+	15	no	rs7568458	0,27	-0,06	20,90	1b	12	3.27170000000001E-310	-	eQTLGen_cis_eQTLs	Yes	GM12878:IM	3,6791E-13	rs1561198	VAMP5, VAMP8, GGCX	2:8578817	A/T	0,4781	2,39E-13	intronic
73	*MAPKAPK*	ENSG00000114738	7867	3	50648951	50686720	+	15	no	rs7623687	0,00	0,08	0,00	4	8	7,7103E-30	-	eQTLGen_cis_eQTLs	No	NA	0,027934	rs7623687	RHOA, AMT, TCTA, CDHRA, KLHDC8B, and othe	3:4944856	A/C	0,1491	3,72E-09	intronic
74	*ATP2B1*	ENSG00000070961	490	12	89981828	90103077	-	15	no	rs2681472	1,00	-1,82	20,80	NA	6	2,55141E-12	+	GTEx_v7_Artery_Tibial	No	NA	1,422E-07	rs2681472	ATP2B1	12:900089	G/A	0,1451	7,63E-11	intronic

Table S3

Enriched GWAS DEG

DEG information			FUMA & MAGMA information							GWAS loci information									
**Index**	**Gene**	**EnsemblID**	**eQTL**	**ciMAP**	**MAGMA p**	**Indep. Signals**	**Position**	**R^2**	**Dist. (bp)**	**Lead SNP**	**Position**	**Gene(s) at locus**	**EAF**	**OR**	**HTN**	**LIPIDS**	**Evidence for respective gene(s)**	**Most likely causal gene function**	**Source**
9	*AMPD2*	ENSG00000116337			3,09E-03	rs7528419	chr1:10,9817,192	0,94	4974	rs599839	chr1:109,822,166	*SORT1, PSCR1, CELSR2*	A (0.78)	1,11		+	mQTL, eQTL, functional data	regulation of apoB secretion and LDL catab	Erdmann J et al. Cardiovasc Res 2018, Teslovich TM et al. Nat 2010;Schunkert H et al., Nat Genet 2011;Samani NJ et al., NEJM 2007
10	*CTSS*	ENSG00000163131	eQTLGen_cis_eQTLs		2,81E-04	rs11810571				rs11810571	chr1:151,762,308	*TDRKH, RP11-98D18.9*	G (0.79)	1,07			eQTL (GTEx)	primary piRNA biogenesis	Erdmann J et al. Cardiovasc Res 2018, Nelson CP et al., Nat Genet 2017;Verweij N et al. Sci Rep 2017
1	*SHE*	ENSG00000169291	BIOS_eQTL_geneLevel; eQTLGen_cis_eQTLs		2,17E-06	rs6689306	chr1:154,395,946	0,90	26121	rs4845625	chr1:154,422,067	*IL6R, AQP10, ATP8B2, CHTOP, UBAP2L*	T (0.47)	1,06			eQTL, functional/regulatory data, MR stuc	IL-6 receptor, immune response	Erdmann J et al. Cardiovasc Res 2018, CARDIoGRAMplusC4D et al., Nat Genet 2013
2	*KCNN3*	ENSG00000143603		IMR90; Mesenchymal_Stem_Cell	0.015	rs6689306	chr1:154,395,946	0.90	26121	rs4845625	chr1:154,422,067	*IL6R, AQP10, ATP8B2, CHTOP, UBAP2L*	T (0.47)	1,06			eQTL, functional/regulatory data, MR stuc	IL-6 receptor, immune response	Erdmann J et al. Cardiovasc Res 2018, CARDIoGRAMplusC4D et al., Nat Genet 2013
11	*IL6R*	ENSG00000160712	GTEx_v7_Whole_Blood; GTEx_v7_Artery_Tibial; eQTLGen_cis_eQTLs		1,90E-06	rs6689306	chr1:154,395,946	0,90	26121	rs4845625	chr1:154,422,067	*IL6R, AQP10, ATP8B2, CHTOP, UBAP2L*	T (0.47)	1,06			eQTL, functional/regulatory data, MR stuc	IL-6 receptor, immune response	Erdmann J et al. Cardiovasc Res 2018, CARDIoGRAMplusC4D et al., Nat Genet 2013
23	*CXCL12*	ENSG00000107562		IMR90; Mesenchymal_Stem_Cell; Trophoblast-like_Cell; hESC	6,32E-10	rs1870634; rs1632484	chr10:44,480,811; chr10:44,773,984	0,94	1840	rs1746048	chr10:44,775,824	*CXCL12*	C (0.87)	1,09			gene function	endothelial regeneration; neutrophil migra	Erdmann J et al. Cardiovasc Res 2018, Schunkert H et al., Nat Genet 2011;Samani NJ et al., NEJM 2007
16	*SH2B3*	ENSG00000111252	BIOS_eQTL_geneLevel; eQTLGen_cis_eQTLs		3,97E-08	rs10774625	chr12:111,910,219	0.95	-25611	rs3184504	chr12:111,884,608	*SH2B3, FLJ21127, ATXN2, and others*	T (0.44)	1,07	+	+	coding, eQTL, pQTL, mQTL	negative regulator of cytokine signaling	Erdmann J et al. Cardiovasc Res 2018, Teslovich TM et al. Nat 2010;Schunkert H et al., Nat Genet 2011;Levy D et al., Nat Genet 2009;Newton-Cheh C et al., Nat Genet 2009;Gudbjartsson DF et al., Nat Genet 2009
24	*PTPN11*	ENSG00000179295	BIOS_eQTL_geneLevel; eQTLGen_cis_eQTLs		3,28E-04	rs10774625	chr12:111,910,219	0.95	-25611	rs3184504	chr12:111,884,608	*SH2B3, FLJ21127, ATXN2, and others*	T (0.44)	1,07	+	+	coding, eQTL, pQTL, mQTL	negative regulator of cytokine signaling	Erdmann J et al. Cardiovasc Res 2018, Teslovich TM et al. Nat 2010;Schunkert H et al., Nat Genet 2011;Levy D et al., Nat Genet 2009;Newton-Cheh C et al., Nat Genet 2009;Gudbjartsson DF et al., Nat Genet 2009
25	*COL4A1*	ENSG00000187498			2,62E-14	rs11617955; rs4773141; rs9515203	chr13:110,818,102; chr13:110,954,353; chr13:111,049,623	0,63	6359	rs4773144	chr13:110,960,712	*COL4A1, COL4A2*	G (0.44)	1,07			functional data, cross phenotype associati	Type IV collagen chain of basement memb	Erdmann J et al. Cardiovasc Res 2018, Schunkert H et al., Nat Genet 2011
26	*COL4A2*	ENSG00000134871			2,73E-10	rs4773141; rs9515203	chr13:110,954,353; chr13:111,049,623	0,63	6359	rs4773144	chr13:110,960,712	*COL4A1, COL4A2*	G (0.44)	1,07			functional data, cross phenotype associati	Type IV collagen chain of basement memb	Erdmann J et al. Cardiovasc Res 2018, Schunkert H et al., Nat Genet 2011
19	*C19ORF38*	ENSG00000214212			1,21E-10	rs6511720	chr19:11,202,306	n/a	n/a	rs3832966	chr14:75,147,552	*TMED10, ZC2HC1C, RPS6KL1, NEK9, EIF2B2e, ACYP1*	I (0.46)	1,05			nearest	negative regulator of TGF-β signaling	Erdmann J et al. Cardiovasc Res 2018, Verweij N et al. Sci Rep 2017
17	*CTSH*	ENSG00000103811	BIOS_eQTL_geneLevel; eQTLGen_cis_eQTLs		5,60E-05	rs7164479; rs4887085	chr15:79,123,054; chr15:79,025,683	0.58; 0.13	-33943; 63428	rs3825807	chr15:79,089,111	*ADAMTS7*	A (0.57)	1,08			coding, functional data, eQTL, mice studie	proliferative response to vascular injury	Erdmann J et al. Cardiovasc Res 2018, Schunkert H et al., Nat Genet 2011;C4D Consortium Nat Genet 2011;Reilly MP et al., Lancet 2011
18	*FES*	ENSG00000182511	BIOS_eQTL_geneLevel; GTEx_v7_Whole_Blood; GTEx_v7_Artery_Aorta; GTEx_v7_Artery_Tibial; eQTLGen_cis_eQTLs		1,11E-13	rs2071382	chr15:91,428,197	0,88	-11647	rs17514846	chr15:91,416,550	*FURIN, FES*	A (0.44)	1,07	+		eQTL	Furin: TGF-β1 precursor and type I MMP; F	Erdmann J et al. Cardiovasc Res 2018, CARDIoGRAMplusC4D et al., Nat Genet 2013;ICBP et al., Nat 2011
8	*DOCK6*	ENSG00000130158			1,25E-03	rs6511720	chr19:11,202,306	0.21	-38705	rs1122608	chr19:11,163,601	*LDLR, SMARCA4*	G (0.77)	1,14		+	rare variants in FH, eQTL data	LDLR: LDL clearance; SMARCA4: functions	Erdmann J et al. Cardiovasc Res 2018, Teslovich TM et al. Nat 2010;MI Genetics Consortium et ak., Nat Genet 2009;Schunkert H et al., Nat Genet 2011;Do R et al., Nat 2015
27	*KANK2*	ENSG00000197256			4,86E-07	rs6511720	chr19:11,202,306	0.21	-38705	rs1122608	chr19:11,163,601	*LDLR, SMARCA4*	G (0.77)	1,14		+	rare variants in FH, eQTL data	LDLR: LDL clearance; SMARCA4: functions	Erdmann J et al. Cardiovasc Res 2018, Teslovich TM et al. Nat 2010;MI Genetics Consortium et ak., Nat Genet 2009;Schunkert H et al., Nat Genet 2011;Do R et al., Nat 2015
20	*VASP*	ENSG00000125753			0.046	rs7412	chr19:45,412,079	no LD	no LD	rs2075650	chr19:45,395,619	*APOE, APOC1, TOMM40, PVRL2, COTL1*	G (0.14)	1,14		+	eQTL, pQTL, functional data, mice studies	APOE: LDL and VLDL clearance, APOC1: ma	Erdmann J et al. Cardiovasc Res 2018, Teslovich TM et al. Nat 2010;IKC Consortium PLoS Genetics 2011
3	*VAMP5*	ENSG00000168899	BIOS_eQTL_geneLevel; GTEx_v7_Whole_Blood; eQTLGen_cis_eQTLs	IMR90; Mesenchymal_Stem_Cell; Trophoblast-like_Cell	8,05E-14	rs7568458	chr2:85,788,175	0,91	21814	rs1561198	chr2:85,809,989	*VAMP5, VAMP8, GGCX*	T (0.45)	1,06			pseudoxanthoma elasticum-like disorder v	VAMP5/8: Intracellular vesicle trafficking, (	Erdmann J et al. Cardiovasc Res 2018, CARDIoGRAMplusC4D et al., Nat Genet 2013
12	*CAPG*	ENSG00000042493	BIOS_eQTL_geneLevel; eQTLGen_cis_eQTLs	IMR90; Mesenchymal_Stem_Cell	4,63E-03	rs7568458	chr2:85,788,175	0.91	21814	rs1561198	chr2:85,809,989	*VAMP5, VAMP8, GGCX*	T (0.45)	1,06			pseudoxanthoma elasticum-like disorder v	VAMP5/8: Intracellular vesicle trafficking, (	Erdmann J et al. Cardiovasc Res 2018, CARDIoGRAMplusC4D et al., Nat Genet 2013
28	*GGT5*	ENSG00000099998			2,08E-05	rs180803				rs180803	chr22:24,262,640	*ADORA2A*	G (0.97)	1,20			nearest	infarct-sparing effects	Erdmann J et al. Cardiovasc Res 2018, Nikpay M et al., Nat Genet 2015
21	*ITGB5*	ENSG00000082781	eQTLGen_cis_eQTLs		0.012	rs62265630	alias			rs142695226	chr3:124,475,201	*UMPS, ITGB5*	G (0.14)	1,08			nearest, gene function, mice studies	cell adhesion and integrin-	Erdmann J et al. Cardiovasc Res 2018, Nelson CP et al., Nat Genet 2017;Verweij N et al. Sci Rep 2017;Klarin D et al., Nat Genet 2017
22	*ARHGEF26*	ENSG00000114790	BIOS_eQTL_geneLevel; GTEx_v7_Artery_Aorta; GTEx_v7_Artery_Tibial		1,09E-05	rs12493885				rs12493885	chr3:153,839,866	*ARHGEF26*	C (0.85)	1,07			mice study, coding, functional data	involved in trans-endothelial migration of l	Erdmann J et al. Cardiovasc Res 2018, Nelson CP et al., Nat Genet 2017;Verweij N et al. Sci Rep 2017;Klarin D et al., Nat Genet 2017
4	*SEMA3F*	ENSG00000001617			4,71E-03	rs7623687				rs7623687	chr3:49,448,566	*RHOA, AMT, TCTA, CDHRA, KLHDC8B, and others*	A (0.86)	1,07			nearest	Small GTPase, involved in cytoskeleton reg	Erdmann J et al. Cardiovasc Res 2018, Nelson CP et al., Nat Genet 2017;Verweij N et al. Sci Rep 2017;Howson JMM et al., Nat Genet 2017;Klarin D et al., Nat Genet 2017
13	*GPX1*	ENSG00000233276	xQTLServer_eQTLs; BIOS_eQTL_geneLevel; eQTLGen_cis_eQTLs		2,98E-05	rs7623687				rs7623687	chr3:49,448,566	*RHOA, AMT, TCTA, CDHRA, KLHDC8B, and others*	A (0.86)	1,07			nearest	Small GTPase, involved in cytoskeleton reg	Erdmann J et al. Cardiovasc Res 2018, Nelson CP et al., Nat Genet 2017;Verweij N et al. Sci Rep 2017;Howson JMM et al., Nat Genet 2017;Klarin D et al., Nat Genet 2017
14	*GNAI2*	ENSG00000114353			0.024	rs7623687				rs7623687	chr3:49,448,566	*RHOA, AMT, TCTA, CDHRA, KLHDC8B, and others*	A (0.86)	1,07			nearest	Small GTPase, involved in cytoskeleton reg	Erdmann J et al. Cardiovasc Res 2018, Nelson CP et al., Nat Genet 2017;Verweij N et al. Sci Rep 2017;Howson JMM et al., Nat Genet 2017;Klarin D et al., Nat Genet 2017
6	*GIMAP1*	ENSG00000213203			0.037	rs3918226				rs3918226	chr7:150,690,176	*NOS3*	T (0.06)	1,14	+		nearest, gene function, mice studies	production of nitric oxide in endothelial ce	Erdmann J et al. Cardiovasc Res 2018, Nikpay M et al., Nat Genet 2015
7	*NOS3*	ENSG00000164867			0.017	rs3918226				rs3918226	chr7:150,690,176	*NOS3*	T (0.06)	1,14	+		nearest, gene function, mice studies	production of nitric oxide in endothelial ce	Erdmann J et al. Cardiovasc Res 2018, Nikpay M et al., Nat Genet 2015
5	*HDAC9*	ENSG00000048052			0.028	rs2107595	chr7:19,049,388	0.48	-12613	rs2023938	chr7:19,036,775	*HDAC9*	C (0.10)	1,08			cross phenotype association, functional da	represses MEF2 activity/beige adipogenesis	Erdmann J et al. Cardiovasc Res 2018, CARDIoGRAMplusC4D et al., Nat Genet 2013
15	*TRIB1*	ENSG00000173334			4,53E-06	rs2954029				rs2954029	chr8:126,490,972	*TRIB1*	A (0.55)	1,06		+	nearest, function, intermediate phenotype	TG, MAPK signalling, SMC proliferation	Erdmann J et al. Cardiovasc Res 2018, Teslovich TM et al. Nat 2010;CARDIoGRAMplusC4D et al., Nat Genet 2013;IKC Consortium PLoS Genetics 2011

Online Table III

Erdmann *et al* 2018

Gene_at_locus	Lead_SNP	Chr	BP	Notes	DEG_pattern	Prioritized	Role
ARHGEF26	rs12493885	3	1,54E+08		14	yes	unknown role
CDH13	rs7500448	16	83045790		10	yes	unknown role
SNRPD2	rs1964272	19	46190268		4	yes	unknown role
CAMSAP2	rs6700559	1	2,01E+08		10		unknown role
LMOD1	rs2820315	1	2,02E+08		14		unknown role
DDX59	rs6700559	1	2,01E+08				unknown role
HHAT	rs60154123	1	2,1E+08				unknown role
FNDC3B	rs12897	3	1,72E+08		10		unknown role
PALLD	rs7696431	4	1,7E+08		14		unknown role
ARHGAP26	rs246600	5	1,43E+08		8		unknown role
CDKN1A	rs1321309	6	36638636		11		unknown role
RAC1	rs7797644	7	6486067		11		unknown role
BCAP29	rs10953541	7	1,07E+08		10		unknown role
HSD17B12	rs7116641	11	43696917		10		unknown role
SIPA1	rs12801636	11	65391317	unclear, many	7		unknown role
NDUFA12	rs7306455	12	95355541				unknown role
MCF2L	rs1317507	13	1,14E+08		3		unknown role
PSMA3	rs2145598	14	58794001		12		unknown role
SERPINA1	rs112635299	14	94838142		8		unknown role
MFGE8	rs8042271	15	89574218		14		unknown role
CFDP1	rs3851738	16	75387533		6		unknown role
BCAR1	rs3851738	16	75387533		10		unknown role
NCK1	rs667920	3	1,36E+08				unknown role
DDX5	rs1867624	17	62387091		4		unknown role
HNRNPUL1	rs8108632	19	41854534		4		unknown role
PROCR	rs867186	20	33764554	many other ge	3		unknown role
EIF6	rs867186	20	33764554	many other ge	12		unknown role
PLCG1	rs6102343	20	39924279		13		unknown role
UNC5C	rs3775058	4	96117371				unknown role
PRDM16	rs2493298	1	3325912				unknown role
MC4R	rs663129	18	57838401				unknown role
ATP1B1	rs1892094	1	1,69E+08				unknown role
NAT2	rs6997340	8	18286997				unknown role
NME7	rs1892094	1	1,69E+08				unknown role
PRIM2	rs9367716	6	57160572				unknown role
PLEKHG1	rs17080091	6	1,51E+08				unknown role
DNAJC13	rs10512861	3	1,32E+08				unknown role
TEX41	rs2252641	2	1,46E+08				unknown role
FIGN	rs12999907	2	1,65E+08				unknown role
PPP2R3A	rs667920	3	1,36E+08				unknown role
KLHDC10	rs11556924	7	1,3E+08				unknown role
PARP12	rs10237377	7	1,4E+08				unknown role
NBEAL1	rs6725887	2	2,04E+08				unknown role
ANKRD13B	rs13723	17	27941886				unknown role
IRS1	rs2972146	2	2,27E+08				unknown role
KCNJ13	rs1801251	2	2,34E+08				unknown role
ALS2CL	rs7633770	3	46688562				unknown role
RTP3	rs7633770	3	46688562				unknown role
SLC22A4	rs273909	5	1,32E+08				unknown role
HDGFL1	rs35541991	6	22583878				unknown role
TIPARP	rs4266144	3	1,57E+08				unknown role
SHROOM3	rs12500824	4	77416627				unknown role

	Counts	
**Lead_SNP**	375	
**Gene_at_locus**	374	*LPAL2* is present twice
**DEG_pattern**	109	
**Prioritized**	26	
**Role**	71	

Gene_at_locus	Lead_SNP	Chr	BP	Notes	DEG_pattern	Prioritized	Role
ARHGEF26	rs1249388	3	1,54E+08		14	yes	unknown role
CDH13	rs7500448	16	83045790		10	yes	unknown role
SNRPD2	rs1964272	19	46190268		4	yes	unknown role

**unknown role**	
Prioritized + DEG_pattern	3
DEG_pattern	24

PRDM8	rs10857147	4	81181072				unknown role
ZNF827	rs35879803	4	1,47E+08				unknown role
TRIP4	rs6494488	15	65024204	unclear, many other genes could be linked			unknown role
UBE2Z	rs46522	17	46988597				unknown role
SMG6	rs216172	17	2126504				unknown role
HHIPL1	rs2895811	14	1E+08				unknown role
ADORA2A	rs180803	22	24262640				unknown role
GOSR2	rs17608766	17	45013271	many other genes could be linked			unknown role
MAP3K7CL	rs2832227	21	30533076				unknown role
GPR22	rs10953541	7	1,07E+08				unknown role
ZNF507	rs12976411	19	32882020				unknown role
CORO6	rs13723	17	27941886				unknown role
FCHO1	rs73015714	19	17855763				unknown role
HP	rs1050362	16	72096666				unknown role
TEX2	rs1867624	17	62387091				unknown role
CCDC92	rs11057401	12	1,24E+08				unknown role
HOXC4	rs11170820	12	54513915				unknown role
KCNE2	rs9982601	21	35599128				unknown role
GIP	rs46522	17	46988597				unknown role
SORT1	rs599839	1	1,1E+08		11	yes	
IL6R	rs4845625	1	1,54E+08		8	yes	
VAMP5	rs1561198	2	85809989		3	yes	
VAMP8	rs1561198	2	85809989		15	yes	
GGCX	rs1561198	2	85809989		9	yes	
CARF	rs6725887	2	2,04E+08		5	yes	
ICA1L	rs6725887	2	2,04E+08		9	yes	
FN1	rs1250229	2	2,16E+08		10	yes	
RHOA	rs7623687	3	49448566	many other ge	12	yes	
ITGB5	rs142695226	3	1,24E+08		14	yes	
MRAS	rs2306374	3	1,38E+08		10	yes	
PLTP	rs3827066	20	44586023		11		
FGF5	rs10857147	4	81181072		9	yes	
HDAC9	rs2023938	7	19036775		3	yes	
NOS3	rs3918226	7	1,51E+08		3	yes	
TRIB1	rs2954029	8	1,26E+08		8	yes	
CXCL12	rs1746048	10	44775824		14	yes	
SWAP70	rs10840293	11	9751196		1	yes	
SH2B3	rs3184504	12	1,12E+08	many other ge	8	yes	
COL4A1	rs4773144	13	1,11E+08		14	yes	
COL4A2	rs4773144	13	1,11E+08		14	yes	
FES	rs17514846	15	91416550		8	yes	
SF3A3	rs61776719	1	38461319				
APOE	rs2075650	19	45395619		11	yes	
APOC1	rs2075650	19	45395619		11	yes	
PCSK9	rs11206510	1	55496039				
SKI	rs36096196	1	2252205		6		
MANEAL	rs61776719	1	38461319		5		
PSCR1	rs599839	1	1,1E+08				
CELSR2	rs599839	1	1,1E+08				
ATP8B2	rs4845625	1	1,54E+08		13		
BLZF1	rs1892094	1	1,69E+08		5		
F5	rs1892094	1	1,69E+08		9		
SELP	rs1892094	1	1,69E+08		3		
PDS5B	rs9591012	13	33058333				
KIF14	rs6700559	1	2,01E+08		5		
WDR12	rs6725887	2	2,04E+08				
IPO9	rs2820315	1	2,02E+08		9		

NAV1	rs2820315	1	2,02E+08		10		
AGT	rs699	1	2,31E+08		14		
ARID4A	rs2145598	14	58794001				
CCDC181	rs1892094	1	1,69E+08				
KCNK5	rs10947789	6	39174922				
ZEB2	rs2252641	2	1,46E+08		8		
CALCRL	rs840616	2	1,88E+08		3		
TFPI	rs840616	2	1,88E+08		10		
PCNX3	rs12801636	11	65391317	unclear, many other genes could be linked			
RAB23	rs9367716	6	57160572				
SERPINA2	rs112635299	14	94838142				
TNS1	rs2571445	2	2,19E+08		14		
COL6A3	rs11677932	2	2,38E+08		14		
FGD5	rs748431	3	14928077		3		
MAP4	rs7617773	3	48193515		6		
TIMM17A	rs2820315	1	2,02E+08				
EYA4–AS1	rs12190287	6	1,34E+08				
SERTAD4	rs60154123	1	2,1E+08				
DIEXF	rs60154123	1	2,1E+08				
MIA3	rs17465637	1	2,23E+08				
AIDA	rs17465637	1	2,23E+08				
HNRNPD	rs11099493	4	82587050		12		
PDE5A	rs11723436	4	1,21E+08		14		
GUCY1A1	rs7692387	4	1,57E+08		14		
GNPAT	rs699	1	2,31E+08				
SEMA5A	rs1508798	5	9556694		14		
MAP3K1	rs3936511	5	55860781		1		
ABCG8	rs6544713	2	44073881				
FOXC1	rs9501744	6	1617143		10		
EDN1	rs12526453	6	12927544		3		
C2	rs3130683	6	31888367	many other ge	8		
CYP17A1	rs12413409	10	1,05E+08				
VEGFA	rs6905288	6	43758873		8		
DST	rs9367716	6	57160572		10		
IYD	rs17080091	6	1,51E+08		9		
LPAL2	rs4252120	6	1,61E+08		9		
LPAL2	rs3798220	6	1,61E+08		9		
CFTR	rs975722	7	1,17E+08				
KDELR2	rs7797644	7	6486067		10		
THSD7A	rs11509880	7	12261911		3		
FAM117B	rs6725887	2	2,04E+08				
MYO1G	rs2107732	7	45077978		15		
KCNH4	rs2074158	17	40257163				
TBXAS1	rs10237377	7	1,4E+08		8		
ATIC	rs1250229	2	2,16E+08				
DOK2	rs6984210	8	22033615		15		
ABCA12	rs1250229	2	2,16E+08				
KLF4	rs944172	9	1,11E+08		11		
DAB2IP	rs885150	9	1,24E+08		10		
OPTN	rs61848342	10	12303813		13		
RUFY4	rs2571445	2	2,19E+08				
TSPAN14	rs17680741	10	82251514		7		
HTRA1	rs4752700	10	1,24E+08		10		
PLEKHA1	rs4752700	10	1,24E+08		13		
TRIM22	rs11601507	11	5701074		4		
GIGYF2	rs1801251	2	2,34E+08				
GHDC	rs2074158	17	40257163				

SVEP1	rs111245230	9	1,13E+08				
SERPINH1	rs590121	11	75274150		10		
PDGFD	rs974819	11	1,04E+08		10		
C1S	rs11838267	12	7175872		10		
LRP1	rs11172113	12	57527283		11		
CDC123	rs61848342	10	12303813				
FLT1	rs9319428	13	28973621		3		
BCAS3	rs7212798	17	59013488				
AMT	rs7623687	3	49448566	many other genes could be linked			
TCTA	rs7623687	3	49448566	many other genes could be linked			
CDHRA	rs7623687	3	49448566	many other genes could be linked			
TMED10	rs3832966	14	75147552		6		
UMPS	rs142695226	3	1,24E+08				
OAZ2	rs6494488	15	65024204	unclear, many	11		
OASL	rs2244608	12	1,21E+08	many other genes could be linked			
NPHP3	rs10512861	3	1,32E+08				
RAD50	rs2706399	5	1,32E+08				
UBA5	rs10512861	3	1,32E+08				
PLCG2	rs7199941	16	81906423		1		
CENPN	rs7199941	16	81906423		5		
SRR	rs216172	17	2126504				
COPRS	rs76954792	17	30033514		10		
RAB11FIP4	rs76954792	17	30033514		5		
KAT2A	rs2074158	17	40257163		3		
PECAM1	rs1867624	17	62387091		3		
CCNL1	rs4266144	3	1,57E+08				
COLGALT1	rs73015714	19	17855763		8		
MRVI1	rs11042937	11	10745394				
TGFB1	rs8108632	19	41854534		15		
RGS12	rs16844401	4	3449652				
MSANTD1	rs16844401	4	3449652				
COTL1	rs2075650	19	45395619		15		
NOA1	rs17087335	4	57838583				
ACYP1	rs3832966	14	75147552				
sep-11	rs12500824	4	77416627				
ZHX3	rs6102343	20	39924279		9		
STBD1	rs12500824	4	77416627				
CETP	rs1800775	16	56961074				
MMP9	rs3827066	20	44586023		11		
BACH1	rs2832227	21	30533076		8		
MORN1	rs36096196	1	2252205				
APOA5	rs964184	11	1,17E+08				
MAD2L1	rs11723436	4	1,21E+08				
PEX10	rs2493298	1	3325912				
PLCH2	rs2493298	1	3325912				
RER1	rs2493298	1	3325912				
FHL3	rs61776719	1	38461319				
UTP11	rs61776719	1	38461319				
DDX60L	rs7696431	4	1,7E+08				
SLC5A3	rs9982601	21	35599128				
INPP5B	rs61776719	1	38461319				
ATXN2	rs3184504	12	1,12E+08	many other genes could be linked			
PPAP2B	rs17114036	1	56962821				
LOX	rs1800449	5	1,21E+08				
LIPA	rs1412444	10	91002927				
IL5	rs2706399	5	1,32E+08				
NGF	rs11806316	1	1,16E+08				

CASQ2	rs11806316	1	1,16E+08				
TDRKH	rs11810571	1	1,52E+08				
RP11-98D18.9	rs11810571	1	1,52E+08				
Ral1	rs12936587	17	17543722				
AQP10	rs4845625	1	1,54E+08				
ASZ1	rs975722	7	1,17E+08				
CHTOP	rs4845625	1	1,54E+08				
UBAP2L	rs4845625	1	1,54E+08				
UHRF1BP1	rs17609940	6	35034800				
BLMH	rs13723	17	27941886				
PI16	rs1321309	6	36638636				
APOA4	rs964184	11	1,17E+08				
ZC2HC1C	rs3832966	14	75147552				
MRPL14	rs6905288	6	43758873				
SLC19A2	rs1892094	1	1,69E+08				
DMTN	rs6984210	8	22033615				
CXCR2	rs2571445	2	2,19E+08				
RELA	rs12801636	11	65391317	unclear, many other genes could be linked			
BEND6	rs9367716	6	57160572				
FAM46A	rs4613862	6	82612271				
CENPW	rs1591805	6	1,27E+08				
SHISA4	rs2820315	1	2,02E+08				
TARID	rs12190287	6	1,34E+08				
KSR2	rs11830157	12	1,18E+08				
RBPMS2	rs6494488	15	65024204	unclear, many other genes could be linked			
ADAMTS7	rs3825807	15	79089111				
LPA	rs3798220	6	1,61E+08				
SLC22A3	rs3798220	6	1,61E+08				
C1orf58	rs17465637	1	2,23E+08				
PLG	rs4252120	6	1,61E+08				
CAPN9	rs699	1	2,31E+08				
MAD1L1	rs10267593	7	1937261				
APOB	rs515135	2	21286057				
ABCG5	rs6544713	2	44073881				
FAM220A	rs7797644	7	6486067				
PRKCE	rs582384	2	45896437				
TMEM247	rs582384	2	45896437				
HNF1A	rs2244608	12	1,21E+08	many other genes could be linked			
PMAIP1	rs663129	18	57838401				
CCM2	rs2107732	7	45077978				
ACAD11	rs10512861	3	1,32E+08				
STN1	rs4918072	10	1,06E+08				
STAG1	rs667920	3	1,36E+08				
CTTNBP2	rs975722	7	1,17E+08				
LOC400684	rs12976411	19	32882020				
TRIM5	rs11601507	11	5701074				
ZC3HC1	rs11556924	7	1,3E+08				
TRIM6	rs11601507	11	5701074				
OR52N1	rs11601507	11	5701074				
GIT1	rs13723	17	27941886				
RPL17	rs9964304	18	47229717				
TOMM40	rs2075650	19	45395619				
LOC102724849	rs1250229	2	2,16E+08				
BMP1	rs6984210	8	22033615				
LINC00607	rs1250229	2	2,16E+08				
REST	rs17087335	4	57838583				
ZNF831	rs260020	20	57714025				

DHX58	rs2074158	17	40257163				
LOC646736	rs2972146	2	2,27E+08				
ZFPM2	rs10093110	8	1,07E+08				
MIR5702	rs2972146	2	2,27E+08				
ANRIL	rs1333049	9	22125503				
CDKN2B-AS	rs1333049	9	22125503				
APOC3	rs964184	11	1,17E+08				
RASGEF1B	rs11099493	4	82587050				
ZNF335	rs3827066	20	44586023				
CTR9	rs11042937	11	10745394				
CDC25A	rs7617773	3	48193515				
SPINK8	rs7617773	3	48193515				
ATP5G1	rs46522	17	46988597				
ZNF589	rs7617773	3	48193515				
PCID2	rs1317507	13	1,14E+08				
FGD6	rs7306455	12	95355541				
DAGLB	rs7797644	7	6486067				
ACAA2	rs9964304	18	47229717				
KLHDC8B	rs7623687	3	49448566	many other genes could be linked			
CUL4A	rs1317507	13	1,14E+08				
TMEM106B	rs11509880	7	12261911				
TOM1L2	rs12936587	17	17543722				
ANGTPL4	rs116843064	19	8429323				
CNNM2	rs12413409	10	1,05E+08				
SMARCA4	rs1122608	19	11163601				
TMEM63B	rs6905288	6	43758873				
MSL2	rs667920	3	1,36E+08				
SMAD3	rs56062135	15	67455630				
ASIP	rs867186	20	33764554	many other genes could be linked			
CCDC97	rs8108632	19	41854534				
CEP70	rs2306374	3	1,38E+08				
ANKS1A	rs17609940	6	35034800				
FLJ21127	rs3184504	12	1,12E+08	many other genes could be linked			
LDLR	rs1122608	19	11163601				
PVRL2	rs2075650	19	45395619				
HGFAC	rs16844401	4	3449652				
ARNTL	rs1351525	11	13301548				
EFCAB5	rs13723	17	27941886				
SURF6	rs579459	9	1,36E+08				
NEK9	rs3832966	14	75147552				
PHYHIP	rs6984210	8	22033615				
MRPS6	rs9982601	21	35599128				
FAM47E	rs12500824	4	77416627				
RAB5	rs2074158	17	40257163				
YY1	rs2895811	14	1E+08				
APOA1	rs964184	11	1,17E+08				
MAT1A	rs17680741	10	82251514				
FAM213A	rs17680741	10	82251514				
TOP1	rs6102343	20	39924279				
MYL4	rs17608766	17	45013271	many other genes could be linked			
RP11-664H17.1	rs10841443	12	20220033				
SCARB1	rs11057830	12	1,25E+08				
EDNRA	rs1878406	4	1,47E+08				
STAT6	rs11172113	12	57527283				
NUDT5	rs61848342	10	12303813				
PEMT	rs12936587	17	17543722				
KIAA1462	rs2505083	10	30335122				

TAS2R1	rs1508798	5	9556694				
DNAJC7	rs2074158	17	40257163				
MIER3	rs3936511	5	55860781				
OR52B6	rs11601507	11	5701074				
B9D2	rs8108632	19	41854534				
HCRT	rs2074158	17	40257163				
C12orf43	rs2244608	12	1,21E+08	many other genes could be linked			
RPS6KL1	rs3832966	14	75147552				
SFTPC	rs6984210	8	22033615				
PHACTR1	rs12526453	6	12927544				
PCIF1	rs3827066	20	44586023				
EIF2B2e	rs3832966	14	75147552				
XPO7	rs6984210	8	22033615				
C4A	rs3130683	6	31888367	many other genes could be linked			
DHX38	rs1050362	16	72096666				
TCF21	rs12190287	6	1,34E+08				
DHODH	rs1050362	16	72096666				
ARL17A	rs17608766	17	45013271	many other genes could be linked			
ITGB4BP	rs867186	20	33764554	many other genes could be linked			
LPL	rs264	8	19813180				
RP11-326A19.4	rs8042271	15	89574218				
N4BP2L2	rs9591012	13	33058333				
NKIRAS2	rs2074158	17	40257163				
SSH2	rs13723	17	27941886				
POLA2	rs12801636	11	65391317	unclear, many other genes could be linked			
ARHGAP42	rs7947761	11	1,01E+08				
NT5C2	rs12413409	10	1,05E+08				
SMCR3	rs12936587	17	17543722				
RASD1	rs12936587	17	17543722				
SH3PXD2A	rs4918072	10	1,06E+08				
ABHD2	rs8042271	15	89574218				
FURIN	rs17514846	15	91416550				
GBGT1	rs579459	9	1,36E+08				
genedesert	rs17581137	15	96146414				
GIPR	rs1964272	19	46190268				
NEURL2	rs3827066	20	44586023				
ABO	rs579459	9	1,36E+08				
NCOA6	rs867186	20	33764554	many other genes could be linked			

Online Table IV

Extracellular and intracellular antibodies used for flow cytometry.

Antibody	Fluorochrome	Clone	Company	Cat. No.
Fixable Viability Dye	eFluor 780	-	eBioscience	65-0865-18
CD45	PE Cy7	2D1	ebioscience	25-9459-42
CD3	BV421	OKT3	Biolegend	317343
CD4	PETR	S3.5	Life Technologies	MHC50417
CD28	BV650	CD28.2	Biolegend	302946
Granzyme B	PE	GB11	ebioscience	12-8899-41
TruStain FcX	-		Biolegend	422302
